# Transmission networks of SARS-CoV-2 in Coastal Kenya during the first two waves: A retrospective genomic study

**DOI:** 10.7554/eLife.71703

**Published:** 2022-06-14

**Authors:** Charles N Agoti, Lynette Isabella Ochola-Oyier, Simon Dellicour, Khadija Said Mohammed, Arnold W Lambisia, Zaydah R de Laurent, John M Morobe, Maureen W Mburu, Donwilliams O Omuoyo, Edidah M Ongera, Leonard Ndwiga, Eric Maitha, Benson Kitole, Thani Suleiman, Mohamed Mwakinangu, John K Nyambu, John Otieno, Barke Salim, Jennifer Musyoki, Nickson Murunga, Edward Otieno, John N Kiiru, Kadondi Kasera, Patrick Amoth, Mercy Mwangangi, Rashid Aman, Samson Kinyanjui, George Warimwe, My Phan, Ambrose Agweyu, Matthew Cotten, Edwine Barasa, Benjamin Tsofa, D James Nokes, Philip Bejon, George Githinji

**Affiliations:** 1 Kenya Medical Research Institute (KEMRI)-Wellcome Trust Research Programme Kilifi Kenya; 2 Pwani University Kilifi Kenya; 3 Spatial Epidemiology Lab (SpELL), Université Libre de Bruxelles Bruxelles Belgium; 4 Department of Microbiology, Immunology and Transplantation, Rega Institute, Laboratory for Clinical and Epidemiological Virology, KU Leuven, University of Leuven Leuven Belgium; 5 Ministry of Health Nairobi Kenya; 6 Nuffield Department of Medicine, University of Oxford Oxford United Kingdom; 7 Medical Research Centre (MRC)/ Uganda Virus Research Institute Entebbe Uganda; 8 MRC-University of Glasgow Centre for Virus Research Glasgow United Kingdom; 9 University of Warwick Coventry United Kingdom; Johns Hopkins University United States; Radboud University Medical Centre Netherlands

**Keywords:** SARS-CoV-2, transmission, genomics, epidemiology, spread, Africa, Viruses

## Abstract

**Background:**

Detailed understanding of severe acute respiratory syndrome coronavirus 2 (SARS-CoV-2) regional transmission networks within sub-Saharan Africa is key for guiding local public health interventions against the pandemic.

**Methods:**

Here, we analysed 1139 SARS-CoV-2 genomes from positive samples collected between March 2020 and February 2021 across six counties of Coastal Kenya (Mombasa, Kilifi, Taita Taveta, Kwale, Tana River, and Lamu) to infer virus introductions and local transmission patterns during the first two waves of infections. Virus importations were inferred using ancestral state reconstruction, and virus dispersal between counties was estimated using discrete phylogeographic analysis.

**Results:**

During Wave 1, 23 distinct Pango lineages were detected across the six counties, while during Wave 2, 29 lineages were detected; 9 of which occurred in both waves and 4 seemed to be Kenya specific (B.1.530, B.1.549, B.1.596.1, and N.8). Most of the sequenced infections belonged to lineage B.1 (n = 723, 63%), which predominated in both Wave 1 (73%, followed by lineages N.8 [6%] and B.1.1 [6%]) and Wave 2 (56%, followed by lineages B.1.549 [21%] and B.1.530 [5%]). Over the study period, we estimated 280 SARS-CoV-2 virus importations into Coastal Kenya. Mombasa City, a vital tourist and commercial centre for the region, was a major route for virus imports, most of which occurred during Wave 1, when many Coronavirus Disease 2019 (COVID-19) government restrictions were still in force. In Wave 2, inter-county transmission predominated, resulting in the emergence of local transmission chains and diversity.

**Conclusions:**

Our analysis supports moving COVID-19 control strategies in the region from a focus on international travel to strategies that will reduce local transmission.

**Funding:**

This work was funded by The Wellcome (grant numbers: 220985, 203077/Z/16/Z, 220977/Z/20/Z, and 222574/Z/21/Z) and the National Institute for Health and Care Research (NIHR), project references: 17/63/and 16/136/33 using UK Aid from the UK government to support global health research, The UK Foreign, Commonwealth and Development Office. The views expressed in this publication are those of the author(s) and not necessarily those of the funding agencies.

## Introduction

Coronavirus Disease 2019 (COVID-19), caused by the severe acute respiratory syndrome coronavirus 2 (SARS-CoV-2), was declared a pandemic on March 11, 2020 (Hu et al., 2021). By February 28, 2021, there had been at least 114 million confirmed cases of COVID-19 and more than 2.6 million deaths worldwide (https://covid19.who.int/). By the same date, Kenya, an East Africa country with a population of around 50 million people, had reported a total of 105,648 COVID-19 cases and 1856 associated deaths, most of which were associated with two distinct waves of infections (MOH, 2021).

Kenya reported its first COVID-19 case on March 13, 2020. In response, the government outlined a series of countermeasures to minimize the effects of a pandemic locally (Brand et al., 2021). For instance, international travel was restricted, international borders closed, public gatherings prohibited, meetings with over 15 participants forbidden, travel from hotspot counties restricted, places of worship, bars, schools, and other learning institutions closed, and a nationwide dusk-to-dawn curfew enforced (Wambua et al., 2022). Despite these measures, the COVID-19 case numbers consistently grew and serological surveys in June 2020 indicated the local epidemic had progressed more than it could be discerned from the limited laboratory testing (Etyang et al., 2021; Uyoga et al., 2021a).

An analysis of blood donor samples collected in the first quarter of 2021 found that anti-SARS-CoV-2 IgG prevalence in Kenya was 48.5% (Adetifa et al., 2021; Uyoga et al., 2021b). Despite this progression of the local epidemic, understanding of local SARS-CoV-2 spread patterns remains limited (Githinji et al., 2021; Wilkinson et al., 2021). During the first two waves, documented cases were concentrated in the major cities, with Nairobi, the capital, accounting for a cumulative total of ~42% of the cases by February 2021 and Mombasa, a coastal city, accounting for ~8% of the cases (Brand et al., 2021). Here, we focused on the latter and its environs.

Throughout the COVID-19 pandemic period, genomic analysis has been crucial for tracking the spread of SARS-CoV-2 and investigating its transmission pathways (Bugembe et al., 2020; Geoghegan et al., 2020; Oude Munnink et al., 2020; Worobey et al., 2020). Previously, we analysed 311 SARS-CoV-2 early genomes collected in Coastal Kenya during Wave 1 (Githinji et al., 2021). In that study, we showed that several Pango lineages had been introduced into Coastal Kenya, but most of them did not take off, except for lineage B.1 (Githinji et al., 2021).

**Figure 1.**
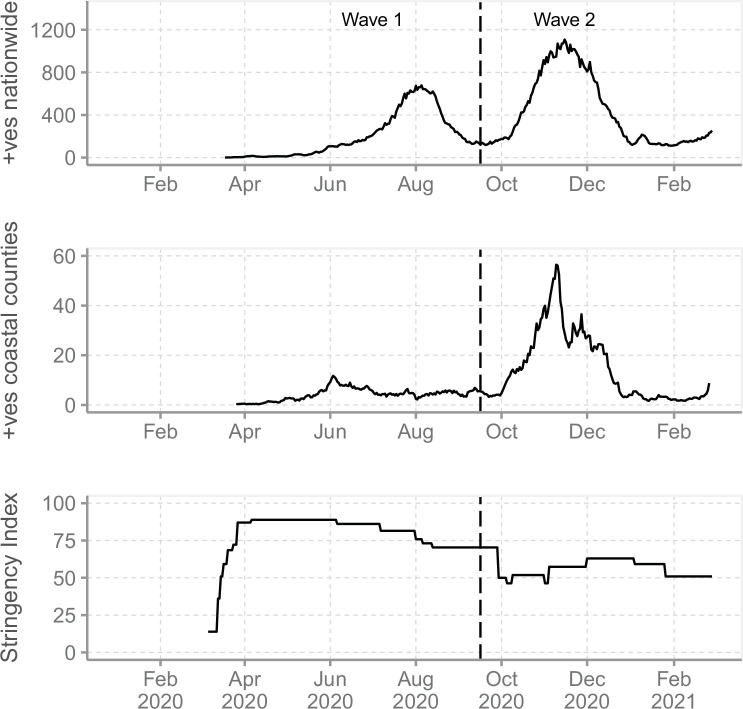
The severe acute respiratory syndrome coronavirus 2 (SARS-CoV-2) epidemic in Kenya and government response. (**A**) The reported daily new cases in Kenya from March 2020 to February 2021 shown as 7-day-rolling average demonstrating the first two national SARS-CoV-2 waves of infections. (**B**) The total reported daily cases for Coastal Kenya counties during the study period shown as 7-day-rolling average per million people. (**C**) The Kenya government COVID-19 intervention level during the study period as summarized by the Oxford Stringency Index (SI) (Hale et al., 2021). Figure 1—source data 1.Number of daily new cases of severe acute respiratory syndrome coronavirus 2 (SARS-CoV-2) in Kenya up to February 26, 2021, and the corresponding 7-day-rolling average. Figure 1—source data 2.Number of daily positive tests per million people for the Coastal Kenya region (all six counties combined). Figure 1—source data 3.Kenya government Coronavirus Disease 2019 (COVID-19) restrictions stringency index during the study period.

The second SARS-CoV-2 wave of infections in Kenya began in mid-September 2020 (Figure 1A), and a mathematical modelling study suggested that this wave was primarily driven by the easing of government restrictions (Brand et al., 2021). Here, we utilized a large set of genome sequences from Coastal Kenya to rule out that a new more transmissible or immune evasive variant was not involved in the second wave and investigate patterns of virus importations, lineage temporal dynamics, and local spread patterns within and between the six counties of Coastal Kenya during the first two epidemic waves of SARS-CoV-2 infections in Kenya.

## Methods

### Study design and population

We analysed SARS-CoV-2 genomic sequences from nasopharyngeal/oropharyngeal (NP/OP) swab samples collected across the six coastal counties of Kenya (Mombasa, Kilifi, Kwale, Taita Taveta, Tana River, and Lamu) between March 17, 2020, and February 26, 2021. Of the six, Mombasa is the most densely populated and has a seaport, an international airport, and an island (Table 1). Kwale and Taita Taveta counties share a border with Tanzania while Lamu includes several islands in the Indian Ocean. Based on the observed nationwide peaks in SARS-CoV-2 infections, we divided the study period into (a) Wave 1, which was the period between March 17 and September 15, 2020, and (b) Wave 2, the period between September 16, 2020, and February 26, 2021 (Figure 1A and B). Wave 2 period began when the number of national daily positive cases started to show a renewed consistent rise after the peak of Wave 1.

**Table 1.** Number of severe acute respiratory syndrome coronavirus 2 (SARS-CoV-2) positives reported by the Ministry of Health in Kenya by February 26, 2021, and breakdown of those conducted at KEMRI-Wellcome Trust Research Programme (KWTRP), including status of sequencing.

County	Total Population size^*^ (%)	Population density^†^	Ministry of Health reported positves ^‡^ (%)	RT-PCR tests (KWTRP, %)	Positives (KWTRP, %)	No. of whole genomes sequenced (%)^§^
Mombasa	1,208,333 (27.9)	5,495	8450 (66.8)	46,143 (55.8)	3139 (49.6)	468 (41.1)
Kilifi	1,453,787 (33.6)	116	2458 (19.4)	12,908 (15.6)	1443 (22.8)	294 (25.8)
Kwale	866,820 (20.0)	105	436 (3.4)	5491 (6.6)	436 (6.9)	102 (9.0)
Taita Taveta	340,671 (7.9)	20	855 (6.7)	14,543 (17.6)	855 (13.5)	196 (13.5)
Tana River	315,943 (7.3)	8	106 (0.8)	877 (1.1)	106 (1.7)	16 (1.7)
Lamu	143,920 (3.3)	23	350 (2.7)	2754 (3.3)	350 (5.5)	63 (5.5)
Overall	4,329,474 (100.0)	52	12,655 (100.0)	82,716 (100.0)	6329 (100.0)	1139 (100.0)

* Number of residents as per the 2019 national population census.

† Units here are number of persons per square kilometre.

‡ The Ministry of Health reports compiled results from all testing centres across the country including KWTRP.

§ The numbers in brackets represents the proportion sequenced of those detected following RT-PCR at the KWTRP.

### Ethical statement

The study protocol was reviewed and approved by the Scientific and Ethics Review Committee (SERU) at Kenya Medical Research Institute (KEMRI), Nairobi, Kenya (SERU protocol #4035). The committee did not require individual patient consent for studies using residual diagnostic material to investigate the SARS-CoV-2 genomic epidemiology for improved public health response.

### Samples analysed

The study used residue NP/OP swab samples collected by the Ministry of Health (MoH) County Department of Health rapid response teams (RRTs) for SARS-CoV-2 diagnostic testing (Agoti et al., 2020; Nyagwange et al., 2022). The RRTs delivered the NP/OP swabs to the KEMRI-Wellcome Trust Research Programme (KWTRP) laboratories within 48 hr in cool boxes with ice packs. The samples were from persons of any age collected following the MoH eligibility criteria that were periodically revised. Participants included persons with (1) acute respiratory illness symptoms, (2) returning travellers from early COVID-19 hotspot countries (i.e. China, Italy, and Iran), (3) persons seeking entry into Kenya at international border points, (4) contacts of confirmed cases, and (5) persons randomly approached as part of the ‘mass’ testing effort to understand the extent of infection spread in the communities.

### SARS-CoV-2 testing and genome sequencing at KWTRP

To purify nucleic acids (NA) in the NP/OP samples, a variety of commercial kits were used, namely, QIAamp Viral RNA Mini Kit, RNeasy QIAcube HT Kit, QIASYMPHONY RNA Kit, TIANamp Virus RNA Kit, Da An Gene Nucleic acid Isolation and Purification Kit, SPIN X Extraction, and RADI COVID-19 detection Kit. The NA extracts were tested for SARS-CoV-2 genetic material using one of the following kits/protocols: (1) the Berlin (Charité) primer-probe set (targeting envelope [E] gene, nucleocapsid [N] or RNA-dependent RNA-polymerase [RdRp]), (2) European Virus Archive – GLOBAL (EVA-g) (targeting E or RdRp genes), (3) Da An Gene Co. detection Kit (targeting N or ORF1ab), (4) BGI RT-PCR kit (targeting ORF1ab), (5) Sansure Biotech Novel Coronavirus (2019-nCoV) Nucleic Acid Diagnostic real-time RT-PCR kit or (6) Standard M kit (targeting E and ORF1ab), and (7) TIB MOLBIOL kit (targeting E gene). Kit/protocol-determined cycle threshold cut-offs were used to define positives (Mohammed et al., 2020).

Though we initially intended to sequence every positive case diagnosed at KWTRP, eventually we settled on sequencing a subset of cases once the epidemic had established (Githinji et al., 2021). Samples sequenced were those with RT-PCR cycle threshold values of <30 with spatial (at county level) and temporal (by month) representation (Figure 2—figure supplement 1). We re-extracted NA from samples selected for sequencing using QIAamp Viral RNA Mini kit following the manufacturer’s instructions and reverse-transcribed the RNA using LunaScript RT SuperMix Kit. The cDNA was amplified using Q5 Hot Start High-Fidelity 2x Mastermix along with the ARTIC nCoV-2019 version 3 primers. The PCR products were run on a 1.5% agarose gel, and for samples whose SARS-CoV-2 amplification was considered successful (amplicons visible) were purified using Agencourt AMPure XP beads and taken forward for library preparation. Sequencing libraries were constructed using Oxford Nanopore Technologies (ONT) ligation sequencing kit and the ONT Native Barcoding Expansion kit as described in the ARTIC protocol (Tyson et al., 2020). Every MinION (Mk1B) run comprised 23 samples and 1 negative (no-template) control.

### Genome assembly and lineage assignment

Following MinION sequencing, the FAST5 files were base-called and demultiplexed using the ONT’s software Guppy v3.5–4.2. Consensus SARS-CoV-2 sequences were derived from the reads using the ARTIC bioinformatics pipeline (https://artic.network/ncov-2019/ncov2019-bioinformatics-sop.html; last accessed August 3, 2021). A threshold of ×20 read depth was required for a base to be included in the consensus genome; otherwise, it was masked with an N (Githinji et al., 2021). Only complete or near-complete genomes with N count <5980 (i.e. >80% coverage) were further analysed.

The consensus genomes were assigned into Pango lineages as described by Rambaut et al., 2020 using Pangolin v3.1.16 (command line version) with Pango v1.2.101 and PangoLEARN model v2021-11-25 (O’Toole et al., 2021). Contextual information about lineages was obtained from the Pango lineage description list available at https://cov-lineages.org/lineage_list.html (last accessed December 21, 2021). Variants of concern (VOC) and variants of interest (VOI) were designated based on the WHO framework as of May 31, 2021 (https://www.who.int/en/activities/tracking-SARS-CoV-2-variants/). Amino acid sequence changes in the Coastal Kenya genomes were investigated using the Nextclade tool v0.14.2 (Hadfield et al., 2018): https://clades.nextstrain.org/ (last accessed August 3, 2021). Mutations in the Kenyan lineages were visualized using the Stanford University CORONAVIRUS ANTIVIRAL & RESISTANCE Database tool on webpage: https://covdb.stanford.edu/page/mutation-viewer/ (last accessed August 3, 2021).

### Global contextual sequences

The global contextual sequences were obtained from GISAID (https://www.gisaid.org/) using the inclusion criteria: (1) presence of the full sample collection date (year–month–day), (2) host recorded as ‘Human’, (3) sample collected between March 1, 2020, and February 28, 2021, and (4) absence of >5980 ambiguous (N) nucleotides. Three analysis datasets were prepared as shown in Figure 5—figure supplement 1.

- Set 1 was for investigating the global context and temporal dynamics of the Pango lineages detected in Coastal Kenya. All data available on GISAID assigned Pango lineages detected in Coastal Kenya were included (n = 420,492).
- Set 2 was for investigating lineage temporal dynamics across widening scales of observation (Coastal Kenya, across Kenya, Eastern Africa, Africa, and globally). These included all eligible African genomes (n = 21,150) and a subset of non-African genomes selected randomly from ‘master dataset’ using the R randomization command: *sample_n*(). A maximum of 30 genomes were selected from each country by year and month. The Eastern Africa subset comprised of 5275 genomes from 10 countries, namely, Ethiopia, Uganda, Rwanda, Malawi, Zimbabwe, Zambia, Mozambique, Madagascar, Reunion (a France overseas territory), and The Comoros.
- Set 3 was for investigating global phylogenetic relationships. It included genomes from the global subset of lineages detected in Coastal Kenya and then randomly split into two subsamples for tractable subsequent phylogenetic analysis (Figure 5—figure supplement 1).

### Phylogenetic analysis

Multiple sequence alignments were prepared in Nextalign v0.1.6 software using the initial Wuhan sequence (Accession number: NC_045512) as the reference with the command:

nextalign−rNC_045512.fasta−iinput.fasta

The alignment was manually inspected in AliView v1.21 to spot any obvious problems/misalignments. Quick non-bootstrapped neighbour-joining trees were created in SEAVIEW v4.6.4 to identify any aberrant sequences which were henceforth discarded. Maximum likelihood (ML) phylogenies were reconstructed using IQTREE v2.1.3 under the GTR (general time-reversible) model of evolution using the command:

./iqtree2−sinput.aligned.fasta−nt4−mGTR

The ML tree was linked to the various metadata (lineage, county, source, etc.) in R programming software v4.0.2 and visualized using the R package ‘ggtree’ v2.4.2. The ML phylogenetic tree was subsequently time-calibrated with the program TreeTime, assuming a constant genomic evolutionary rate of SARS-CoV-2 of 8.4 × 10^–4^ nucleotide substitutions per site per year (Sagulenko et al., 2018), and using the command.

treetime−−treinput.aligned.fasta.treefile−−alninput.aligned.fasta−−clock−rate0.00084–−datesdates.csv

Outlier sequences deviating from the molecular clock were identified by TreeTime and excluded using the R package ‘treeio’. TempEst v1.5.3 was then used to assess the consistency of nucleotide evolution of the analysed data with a molecular clock. A linear regression of root-to-tip genetic distances against sampling dates was plotted in RStudio and the coefficient of determination (R^2^) assessed. The resulting trees were visualized using the R package ‘ggtree’ v2.4.2.

### Import/export analysis

We estimated the number of viral importation/exportation events between Coastal Kenya and the rest of the world by ancestral state reconstruction from the global ML tree using methods similar to those described by Tegally et al., 2021; Wilkinson et al., 2021. This was achieved using the date and location annotated tree topology to count the number of transitions between Coastal Kenya counties and the rest of the world (‘non-coastal Kenya’) using the Python script developed by the KwaZuluNatal Research Innovation & Sequencing Platform team (KRISP, https://github.com/krisp-kwazulu-natal/SARSCoV2_South_Africa_major_lineages/tree/main/Phylogenetics; last accessed August 4, 2021). The results were plotted in R using the package ‘ggplot2’ v3.3.3. This analysis was repeated with a further two subsamples of the global background data and with also a downsampled set of the Coastal Kenya genomes that were normalized spatially and temporally (Supplementary file 5).

### Phylogeographic analyses

We used a discrete phylogeographic approach (Lemey et al., 2009) to investigate the dispersal history of SARS-CoV-2 lineages among coastal counties while trying to mitigate the potential impact of sampling bias by subsampling Kenyan counties according to their relative epidemiological importance during the study period. For this purpose, we implemented a subsampling procedure similar to the one described by Dellicour and colleagues to analyse the circulation of SARS-CoV-2 among New York City boroughs during the first phase of the American epidemic (Dellicour et al., 2021b). Specifically, we performed replicated discrete phylogeographic analyses based on random subset of genomic sequences. Each subset was obtained by subsampling available Kenyan genomic sequences according to the COVID-19 incidence recorded in each sampled county during the study period (Mombasa: 699 cases/100,000 people; Kilifi: 169; Kwale: 50; Taita Taveta: 251; Tana River: 34; and Lamu: 243; Table 1). Because Lamu was the proportionally least sampled county when comparing available number of sequences to local incidence, the sampling intensity of this county (63 genomic sequences sampled for a recorded incidence of 243 cases per 100,000 people) served as reference for downsampling the available number of sequences from the other counties. The resulting downsampled data sets comprised the following number of sequences: n = 181 (Mombasa), 44 (Kilifi), 13 (Kwale), 65 (Taita Taveta), 9 (Tana River), and 63 (Lamu). To investigate the impact of the stochastic subsampling procedure, we performed 10 replicated analyses each based on a distinct subsampling.

Discrete phylogeographic inferences were all performed using the discrete diffusion model (Lemey et al., 2009) implemented in the software package BEAST 1.10 (Suchard et al., 2018). In a first time and following a previously described analytical pipeline (Dellicour et al., 2021a), a preliminary discrete phylogeographic reconstruction was performed to delineate clades corresponding to distinct introduction events of SARS-CoV-2 lineages into Kenya. For this initial phylogeographic analysis, we only considered two possible ancestral locations: ‘Kenya’ and ‘other location’. We conducted Bayesian inference through Markov chain Monte Carlo (MCMC) for 10^6^ iterations and sampled every 10^3^ iterations. To ensure that effective sample size (ESS) values associated with estimated parameters were all >200, we inspected MCMC convergence and mixing properties using the program Tracer 1.7 (Rambaut et al., 2018). We then generated a maximum clade credibility (MCC) tree using the program TreeAnnotator 1.10 (Suchard et al., 2018) after having discarded 10% of sampled trees as burn-in. Finally, we used the resulting MCC tree to delineate phylogenetic clades corresponding to independent introduction events into Kenya.

In a second time, each replicated phylogeographic analysis was conducted along the overall time-scaled phylogenetic tree previously obtained with TreeTime (see the ‘Phylogenetic analysis’ subsection), within which Kenyan clades were delineated in the previous step (preliminary discrete phylogeographic inference), and whose Kenyan tips were subsampled with the function ‘drop.tip’ from the R package ‘ape’ (Paradis and Schliep, 2019) according to the above-described subsampling procedure. In order to identify the best-supported lineage transitions events between sampled coastal counties, we here used the Bayesian stochastic search variable selection (BSSVS) approach (Lemey et al., 2009) implemented in BEAST 1.10 (Suchard et al., 2018). Each MCMC was run for 10^8^ iterations and sampled every 10^4^ iterations. As described above, MCMC convergence and mixing properties were again inspected with Tracer. Statistical supports associated with transition events connecting each pair of sampled counties were obtained by computing adjusted Bayes factor (BF) supports, that is, BF supports that consider the relative abundance of samples by location (Dellicour et al., 2021b; Vrancken et al., 2021).

### Epidemiological data

The Kenya daily case data between March 2020 and February 2021 was downloaded from Our World in Data (https://ourworldindata.org/coronavirus/country/kenya; last accessedAugust 4 2021). The daily number of confirmed cases in each county during the study period was obtained from the Kenya Ministry of Health website, which provided the breakdown by county. Metadata for the Coastal Kenya samples was gathered from Ministry of Health case investigation forms delivered together with the samples to KWTRP.

### Kenya COVID-19 response

We derived the overall status of Kenya government COVID-19 interventions using the Oxford Stringency Index (SI) available from Our World in Data database (https://ourworldindata.org/coronavirus/country/kenya, last accessed on January 18, 2022; Figure 1C). Oxford SI is based on nine response indicators rescaled to values of 0–100, with 100 being strictest (Hale et al., 2021). The nine response indicators used to form the SI are (1) school closures, (2) workplace closures, (3) cancellation of public events, (4) restrictions on public gatherings, (5) closures of public transport, (6) stay-at-home requirements, (7) public information campaigns, (8) restrictions on internal movements, and (9) international travel controls. The various government COVID-19 measures and the dates they took effect or when they were lifted are provided in Supplementary file 1 and are also reviewed in detail in Brand et al., 2021; Wambua et al., 2022.

### Statistical analysis

Statistical data analyses were performed in R v4.0.5. Summary statistics (proportions, means, median, and ranges) were inferred where applicable. The ‘lm’ function in R was used to fit a linear regression model evaluating the relationship between sampling dates and root-to-tip genetic distance in the ML phylogeny. The goodness of fit was inferred from the correlation coefficient. Proportions were compared using chi-square test or Fisher’s exact test as appropriate.

## Results

### COVID-19 waves in Coastal Kenya and sequencing at KWTRP

**Figure 2.**
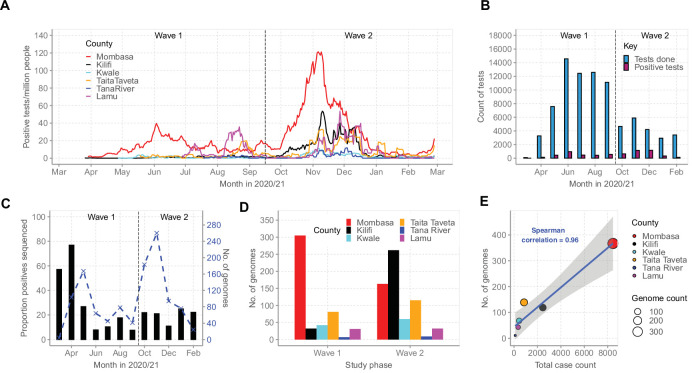
Severe acute respiratory syndrome coronavirus 2 (SARS-CoV-2) cases on the Kenyan Coast. (**A**) The epidemic curves for each of the six Coastal Kenya counties derived from the daily positive case numbers, 7-day-rolling average, as reported by the Ministry of Health. (**B**) The monthly count of SARS-CoV-2 RT-PCR tests undertaken at the KEMRI-Wellcome Trust Research Programme (KWTRP) and those positive during the study period. (**C**) The monthly proportion (black bars, primary y-axis) and number (dashed blue line, secondary y-axis) of samples sequenced from total SARS-CoV-2 positives detected at KWTRP. (**D**) County distribution of the sequenced 1139 samples by wave number. (**E**) Linear regression fit of the number of Ministry of Health-reported Coronavirus Disease 2019 (COVID-19) cases in the six Coastal Kenya counties as of February 26, 2021, against the number of SARS-CoV-2 genome sequences obtained at KWTRP during the period. Figure 2—source data 1.Number of daily positive tests per million people for each of the six Coastal Kenya counties. Figure 2—source data 2.Total monthly severe acute respiratory syndrome coronavirus 2 (SARS-CoV-2) tests at KEMRI-Wellcome Trust Research Programme (KWTRP) and identified positives. Figure 2—source data 3.Monthly proportion of positive samples whole genome sequenced from the positive tests at KEMRI-Wellcome Trust Research Programme (KWTRP). Figure 2—source data 4.Number of genomes available across the six coastal counties during the two national waves of infections. Figure 2—source data 5.Total case count and number genomes available from the six coastal counties.

By February 2021, Mombasa, Lamu, and Taita Taveta counties had experienced at least two waves of SARS-CoV-2 infections while Kilifi, Kwale, and Tana River had experienced only a single wave of infections (Figure 2A). Up to February 26, 2021, the MoH had reported a cumulative total of 12,655 cases for all the six coastal counties, a majority from Mombasa County (n = 8450, 67%; Table 1). Over the same period, KWTRP tested an aggregate of 82,716 NP/OP swabs from the six coastal counties, 6329 (8%) were positive, distributed by month as shown in Figure 2B. The majority of the KWTRP positives were from Mombasa County (n = 3139, 50%).

**Figure 2—figure supplement 1.**
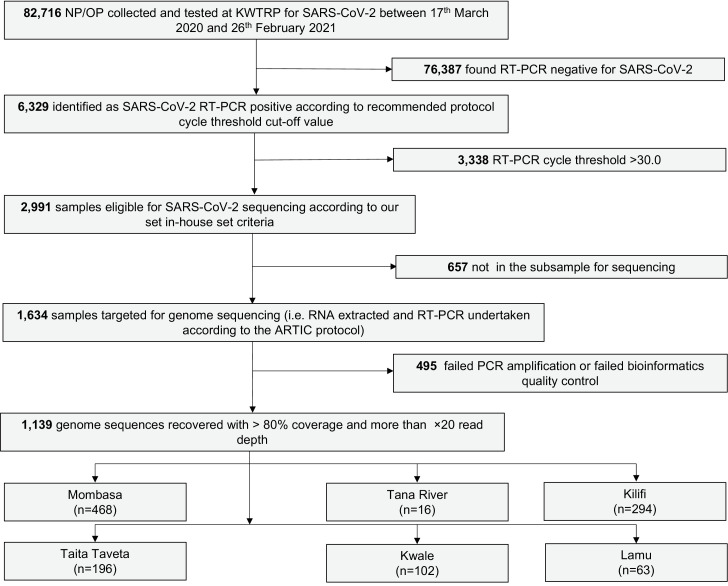
Laboratory flow of samples analysed in this study.

Among the positive cases, we sequenced 1139 cases (18%) distributed by county as reported in Table 1. The sample flow is summarized in Figure 2—figure supplement 1. The sequenced samples were spread across Wave 1 (n = 499, 44%) and Wave 2 (n = 640, 56%; Figure 2C and D) and corresponded to approximately one sequence for every 11 confirmed cases in the region. A high correlation was observed between the MoH case count and the number of samples sequenced for each county (R^2^ = 0.9216, Figure 2E).

### Demographic characteristics of the sequenced sample

The demographic details of the SARS-CoV-2-positive participants identified at KWTRP are presented in Table 2. Compared to Wave 1, Wave 2 identified slightly older individuals as positive (median age, 34 vs. 35 years); females were identified as positive more often (26% vs. 32%), Kenyans were identified as positive more often (80% vs. 88%), and fewer individuals with international travel histories were identified as positive (12% vs. 4%). Tanzania ranked second in terms of the number of individuals providing sequenced samples (n = 34, 4%). A total of 119 samples (15%) were sequenced from people who had recently travelled internationally (within 14 days). Travel history information was missing for 613 (54%) sequenced cases (Table 2).

**Table 2.** Demographic characteristics of the positive cases identified at KEMRI-Wellcome Trust Research Programme (KWTRP) in Coastal Kenya by sequencing status and wave period.

Characteristic	Total positives	Overall sequencing status			Total positives by wave period			Total sequenced by wave period		
	(n = 6329)(%)	Sequenced(n = 1139)(%)	Non-sequenced(n = 5190)(%)	p-Value^†^	Wave 1 (n = 2849)(%)	Wave 2 (n = 3480)(%)	p-Value^†^	Wave 1 (n = 499)(%)	Wave 2 (n = 640)(%)	p-Value^†^
*Age category (years)*				<0.001			0.0149			0.0419
0–9	178 (2.8)	22 (1.9)	156 (3.0)		94 (3.3)	84 (2.4)		11 (2.2)	11 (1.7)	
10–19	472 (7.5)	85 (7.5)	387 (7.5)		185 (6.5)	287 (8.2)		21 (4.2)	64 (10.0)	
20–29	1682 (26.6)	234 (20.5)	1,448 (27.9)		769 (27.2)	913 (26.1)		94 (18.9)	140 (21.8)	
30–39	1653 (26.1)	290 (25.5)	1,363 (26.3)		764 (27.0)	889 (25.4)		123 (24.7)	167 (26.1)	
40–49	1140 (18.0)	218 (19.1)	922 (17.8)		488 (17.2)	652 (18.6)		88 (17.7)	130 (20.3)	
50–59	605 (9.6)	122 (10.7)	483 (9.3)		247 (8.7)	358 (10.2)		57 (11.4)	65 (10.1)	
60–69	187 (2.9)	46 (4.0)	141 (2.7)		78 (2.8)	109 (3.1)		23 (4.6)	23 (3.6)	
70–79	74 (1.1)	17 (1.5)	57 (1.1)		33 (1.2)	41 (1.2)		7 (1.4)	10 (1.6)	
80+	13 (0.2)	4 (0.4)	9 (0.2)		7 (0.2)	6 (0.2)		3 (0.6)	1 (0.2)	
Missing	325 (3.25)	101 (8.9)	224 (4.3)		167 (5.9)	158 (4.5)		71 (14.3)	30 (4.7)	
*Gender*				0.554			<0.001			0.1979
Female	1896 (29.9)	333 (29.2)	1563 (30.1)		763 (26.9)	1,133 (32.4)		125 (25.1)	208 (32.4)	
Male	4058 (64.1)	686 (60.2)	3372 (65.0)		1860 (65.7)	2198 (62.9)		288 (57.8)	398 (62.1)	
Missing	375 (5.9)	120 (10.5)	255 (4.9)		209 (7.4)	166 (4.7)		85 (17.1)	85 (5.5)	
*Nationality*				<0.001			<0.001			<0.001
Kenyan	5356 (84.6)	870 (76.4)	4486 (86.4)		2270 (80.2)	3086 (88.2)		316 (63.5)	554 (86.4)	
Tanzania	131 (2.1)	34 (3.0)	97 (1.9)		81 (2.9)	50 (1.4)		25 (5.0)	9 (1.4)	
Uganda	16 (0.3)	1 (0.1)	15 (0.3)		10 (0.4)	6 (0.2)		0 (0.2)	4 (0.0)	
*Ethiopia*	14 (0.2)	4 (0.4)	10 (0.2)		0 (0.0)	14 (0.4)		1 (0.2)	0 (0.0)	
Other†	117 (1.84)	24 (2.1)	93 (1.8)		46 (1.6)	71 (2.0)		6 (1.2)	18 (2.8)	
Missing	695 (10.9)	206 (18.1)	489 (9.4)		425 (15.0)	270 (7.7)		150 (30.1)	56 (8.7)	
*Travel history* *				<0.001			<0.001			<0.001
Yes	485 (7.7)	119 (10.4)	366 (7.1)		340 (12.0)	145 (4.1)		83 (16.7)	36 (5.6)	
No	2562 (40.7)	407 (35.7)	2155 (41.5)		1372 (48.4)	1190 (34.0)		189 (38.0)	218 (34.0)	
Missing	3282 (51.9)	613 (53.8)	2669 (51.4)		1120 (39.5)	2162 (61.8)		226 (45.4)	387 (60.4)	

* Defined as having moved into Kenya in the previous 14 days or sampled at a point of entry (POE) into Kenya.

† p-value calculated using a Pearson’s chi-squared test, for variables where some cells in the table had <5 observations, Fishers' exact test was applied.

### Viral lineages circulating in Coastal Kenya

**Figure 3.**
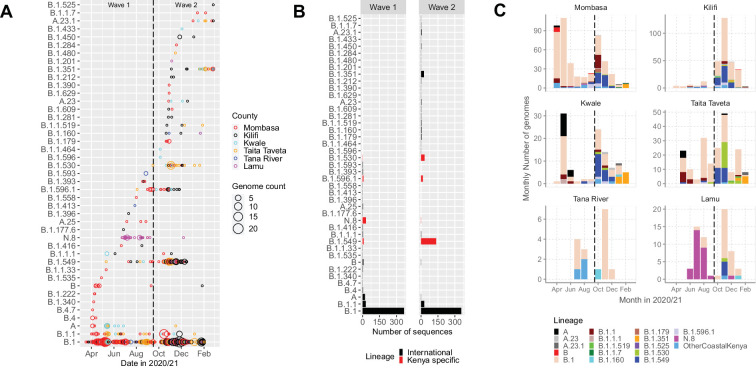
Lineage introductions and temporal dynamics in Coastal Kenya. (**A**) Timing of detections of severe acute respiratory syndrome coronavirus 2 (SARS-CoV-2) Pango lineages in the sequenced 1139 Coastal Kenya samples. The circle size scaled by number of daily detections. The vertical dashed line demarcates the date of transition from Wave 1 to Wave 2. (**B**) Cumulative detections by Pango lineage detections by wave number. The bars are coloured by known information about the lineages; Kenya specific (B.1.530, B.1.549, B.1.596.1, and N.8, red bars) or international lineages (black bars). (**C**) Monthly distribution of the common lineages identified across the six counties presented as raw counts of the sequenced infections. Lineages detected in less than four cases or not considered a variant of concern (VOC) or variant of interest (VOI) were put together and referred to as ‘other Coastal Kenya lineages’. This group comprises 26 lineages, namely, A.25, B.1.1.33, B.1.1.464, B.1.177.6, B.1.201, B.1.212, B.1.222, B.1.281, B.1.284, B.1.340, B.1.390, B.1.393, B.1.396, B.1.413, B.1.416, B.1.433, B.1.450, B.1.480, B.1.535, B.1.558, B.1.593, B.1.596, B.1.609, B.1.629, B.4, and B.4.7. Figure 3—source data 1.The total daily number of sequenced cases for each identified lineage across each of the six coastal counties. Figure 3—source data 2.Total cases sequenced for each 43 identified lineages in the two waves of infection in Kenya. Figure 3—source data 3.The monthly number of cases for each lineage across the two waves of infection in Kenya.

The 1139 Coastal Kenya genomes were classified into 43 Pango lineages, including 4 first identified in Kenya (N.8, B.1.530, B.1.549, and B.1.596.1) and 2 global variants of concern (VOC); B.1.1.7 (Alpha) and B.1.351 (Beta; Table 3). A total of 23 and 29 lineages were observed during Wave 1 and Wave 2, respectively, with 9 lineages detected in both waves (Figure 3A and B). Nineteen lineages were identified in three or more samples with the top six lineages accounting for 89% of the sequenced infections, namely, B.1 (n = 723, 63%), B.1.549 (n = 143, 13%), B.1.1 (n = 57, 5%), B.1.530 (n = 32, 3%), N.8 (n = 31, 3%), and B.1.351 (n = 26, 2%; Table 3). Many of the lineages were first detected in Mombasa (n = 21, 49%) before observation in other counties (Supplementary file 3). The temporal pattern of detection for the lineages across six counties is shown in Figure 3C.

**Table 3.** Lineages observed in Coastal Kenya, their county distribution, global history, and variants of concern (VOC)/variants of interest (VOI) status.

Lineage	Frequency (%)	Mombasa	Kilifi	Kwale	Taita Taveta	Tana River	Lamu	Earliest date	Number assigned	Description
A	22 (0.3)	3	-	13	6	-	-	Decmber 30, 2019	2224	Root of the pandemic lies within lineage A Predominantly found in China
A.23	4 (0.1)	1	1	2	-	-	-	August 14, 2020	92	Predominantly found in Uganda
A.23.1	6 (0.1)	2	1	1	2	-	-	September 21, 2020	1191	International lineage
A.25	3 (0.0)	3	-	-	-	-	-	June 8, 2020	47	Predominantly found in Uganda
B	9 (0.1)	8	1	-	-	-	-	December 24, 2019	7358	Second major haplotype (and first to be discovered)
B.1	723 (11.4)	328	192	44	119	12	28	January 1, 2020	88,731	Predominantly found in Europe, origin corresponds to the Northern Italian outbreak early in 2020
B.1.1	57 (0.9)	33	6	5	13	-	-	January 8, 2020	49,562	Predominantly found in Europe
B.1.1.1	5 (0.1)	1	2	2	-	-	-	March 2, 2020	2827	Predominantly found in England
B.1.1.33	1 (0.0)	1	-	-	-	-	-	March 1, 2020	2117	Predominantly found in Brazil
B.1.1.464	1 (0.0)	-	-	1	-	-	-	April 1, 2020	666	Predominantly found in USA
B.1.1.519	4 (0.1)	-	2	-	2	-	-	July 30, 2020	23,815	Predominantly found in USA/ Mexico
B.1.1.7	2 (0.0)	2	-	-	-	-	-	September 3, 2020	1,062,326	Alpha variant of concern
B.1.160	5 (0.1)	-	2	1	-	1	1	February 2, 2020	28,128	Predominantly found in Europe
B.1.177.6	1 (0.0)	-	1	-	-	-	-	May 29, 2020	949	Predominantly found in Wales
B.1.179	5 (0.1)	5	-	-	-	-	-	March 9, 2020	242	Predominantly found in Denmark
B.1.201	1 (0.0)	-	-	-	-	-	1	March 6, 2020	173	Predominantly found in the UK
B.1.212	2 (0.0)	-	2	-	-	-	-	March 3, 2020	59	Predominantly found in South America
B.1.222	2 (0.0)	2	-	-	-	-	-	February 24, 2020	568	Predominantly found in Scotland
B.1.281	2 (0.0)	-	2	-	-	-	-	April 8, 2020	41	Predominantly found in Bahrain
B.1.284	1 (0.0)	1	-	-	-	-	-	March 9, 2020	85	Predominantly found in TX,USA
B.1.340	1 (0.0)	1	-	-	-	-	-	March 13, 2020	221	Predominantly found in USA
B.1.351	26 (0.4)	6	5	8	7	-	-	September 1, 2020	29,720	Beta variant of concern
B.1.390	1 (0.0)	1	-	-	-	-	-	March 25, 2020	91	Predominantly found in USA
B.1.393	3 (0.0)	2	1	-	-	-	-	May 29, 2020	34	Predominantly found in Uganda
B.1.396	1 (0.0)	-	1	-	-	-	-	April 6, 2020	1375	Predominantly found in USA
B.1.413	1 (0.0)	-	-	-	-	1	-	March 12, 2020	195	Predominantly found in USA
B.1.416	2 (0.0)	1	1	-	-	-	-	April 11, 2020	594	Predominantly found in Senegal/ Gambia, reassigned from B.1.5.12
B.1.433	1 (0.0)	-	-	1	-	-	-	August 3, 2020	314	Predominantly found in TX, USA
B.1.450	3 (0.0)	-	3	-	-	-	-	March 14, 2020	86	Predominantly found in TX, USA
B.1.480	1 (0.0)	-	-	-	1	-	-	July 3, 2020	386	Predominantly found in England, Australia, Sweden, Norway
B.1.525	1 (0.0)	-	1	-	-	-	-	March 28, 2020	8012	Eta variant of interest
B.1.530	32 (0.5)	3	4	2	22	-	1	October 1, 2020	111	Predominantly found in Kenya
B.1.535	1 (0.0)	1	-	-	-	-	-	March 22, 2020	29	Predominantly found in Australia
B.1.549	143 (2.3)	42	56	18	23	-	4	May 11, 2020	171	Predominantly found in Kenya and England
B.1.558	1 (0.0)	1	-	-	-	-	-	April 6, 2020	211	Predominantly found in USA/ Mexico
B.1.593	2 (0.0)	-	-	-	-	2	-	July 3, 2020	99	Predominantly found in USA
B.1.596	1 (0.0)	-	-	1	-	-	-	April 11, 2020	9968	Predominantly found in USA
B.1.596.1	24 (0.4)	12	8	3	1	-	-	September 7, 2020	83	Predominantly found in Kenya
B.1.609	2 (0.0)	1	1	-	-	-	-	March 10, 2020	1879	Predominantly found in USA/ Mexico
B.1.629	1 (0.0)	1	-	-	-	-	-	July 12, 2020	231	Lineage circulating in several countries
B.4	3 (0.0)	3	-	-	-	-	-	January 18, 2020	386	Predominantly found in Iran
B.4.7	1 (0.0)	1	-	-	-	-	-	March 14, 2020	68	Predominantly found in Africa and UAE
N.8	31 (0.5)	2	1	-	-	-	28	June 23, 2020	15	Alias of B.1.1.33.8, predominantly found in Kenya

**Figure 4.**
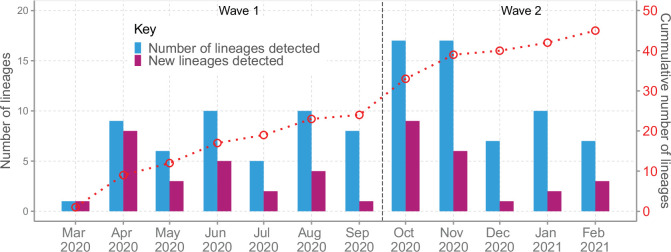
Lineage detection patterns in Coastal Kenya showing monthly count of total detected lineages, detected new lineages, and commutative total of detected lineages in Coastal Kenya across the study period (secondary axis). Figure 4—source data 1.New, total circulating and cumulative Pango lineage counts by month in Coastal Kenya. Figure 4—source data 2.Distribution of the detected Pango lineages by travel history information in Coastal Kenya.

We detected an average of eight Pango lineages in circulation per month during the study period; the lowest (n = 1) in March 2020 and the highest (n = 17) in November 2020 (Figure 4). The earliest sequences for 7 lineages (16%) came from individuals who reported recent international travel while earliest sequences for 16 lineages (37%) came from individuals who had no history of recent travel, and the earliest sequences for 20 lineages (47%) came from individuals who had no information about travel history (Figure 4—figure supplement 1). Among the individuals with recent travel history, the top five lineages were B.1, A, B.1.1, B.1.549, and B.1.351 (Figure 4—figure supplement 2). Most of the lineages detected in Coastal Kenya were first detected in Mombasa County (n = 14, 58%; Supplementary file 3).

**Figure 4—figure supplement 1.**
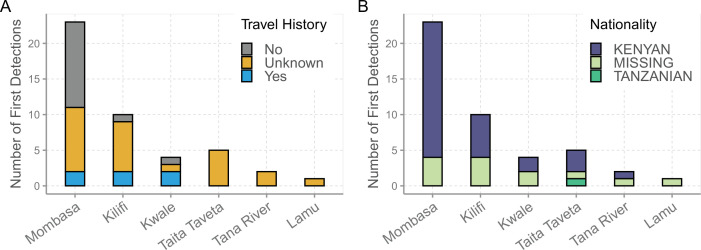
Demographic characteristics of the sequenced index cases of the 43 lineages identified in Coastal Kenya. (**A**) The travel history distribution.and (**B**) the nationality distribution.

**Figure 4—figure supplement 2.**
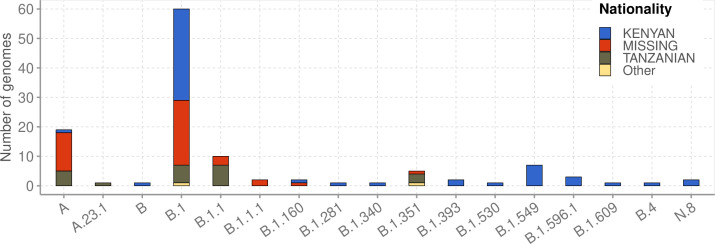
Lineages detected among individuals who reported a recent international travel history (n = 119) and their distribution by nationality.

### SARS-CoV-2 lineage dynamics beyond Coastal Kenya

**Figure 5.**
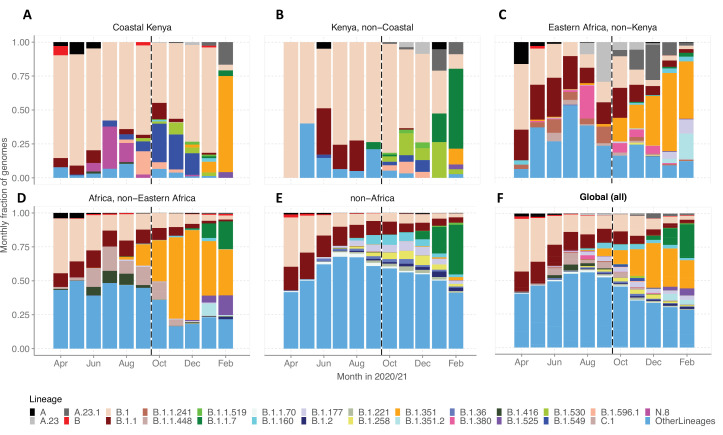
Investigation of lineage spatial temporal dynamics at widening scales of observation. (**A**) Monthly prevalence of detected lineages in Coastal Kenya from the sequenced 1139 genomes. (**B**) Monthly prevalence of detected lineages in Kenya (outside coastal counties) from 605 contemporaneous genomes data is available in GISAID. (**C**) Monthly prevalence of detected lineages in Eastern Africa from 3531 contemporaneous genomes from 10 countries whose contemporaneous data are available in GISAID. The included countries were Comoros, Ethiopia, Madagascar, Malawi, Mozambique, Reunion, Rwanda, Uganda, Zambia, and Zimbabwe. (**D**), Monthly distribution of detected lineages in African countries (excluding Eastern Africa). A total of 14,874 contemporaneous genomes from 37 countries that were available in GISAID are included in the analysis. (**E**) Monthly prevalence of detected lineages in a global subsample of 19,993 contemporaneous genomes from 147 countries that were compiled from GISAID (see detail in ‘Methods’ section). Genomes from African samples are excluded in this panel. (**F**) Includes all genomes analysed from the scales (**A–E**). Lineages not among the top 10 in at least one of the five scales of observation investigated have been lumped together as ‘Other lineages’. Figure 5—source data 1.Monthly counts for the top lineages observed at the different scales of observation analysed.

We evaluated various scales of observation to illustrate the spatial-temporal lineage dynamics during our study period (Figure 5). The genome set was carefully selected to minimize sampling bias (Figure 5—figure supplement 1). A total of 33 Pango lineages were identified for the Kenya sample, 125 lineages for Eastern Africa, 337 lineages for Africa, and 950 lineages globally (Supplementary file 4). The number of lineages detected for the different scales was consistent with the widening scope except for across Kenya where a relatively small number of genomes were available. The top 10 Pango lineages observed at each scale of observation is provided in Supplementary file 5.

**Figure 5—figure supplement 1.**
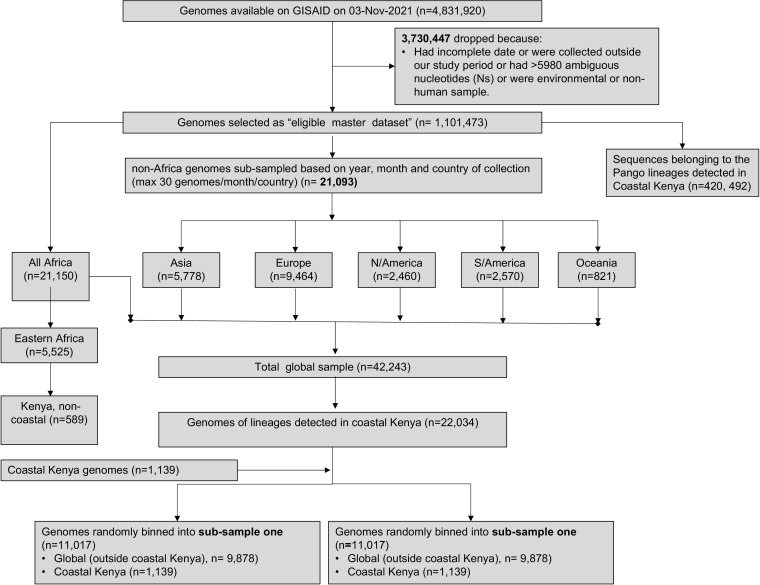
Flow of the genomes retrieved from GISAID and used in the comparative genomic epidemiology for lineage dynamics analysis and global context of the Coastal Kenya genomes.

**Figure 5—figure supplement 2.**
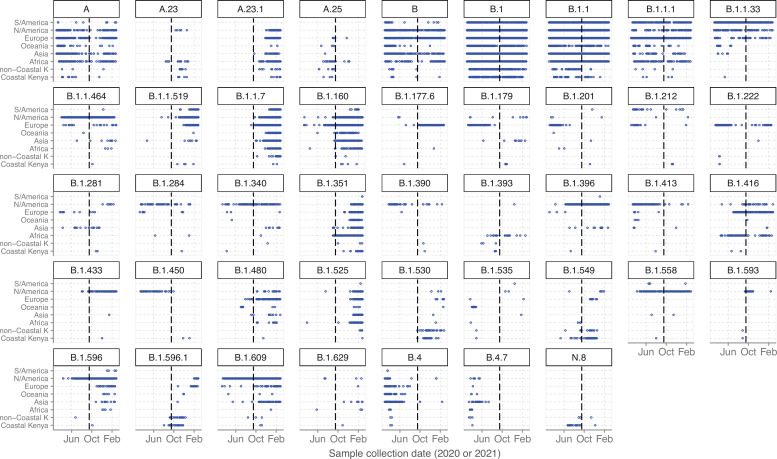
Global context and temporal dynamics of the Pango lineages detected in Coastal Kenya. The plot was derived from a combination of 420,492 global genomes available on GISAID sampled between March 1, 2020, and February 28, 2021, and the 1139 Coastal Kenya genomes generated in this study. The GISAID data was stratified by continent and those from Kenya (and sampled in non-coastal counties) separated from those from the rest of Africa. The dashed vertical black line demarcates the Wave 1 and Wave 2 period inferred from the Kenya epidemic.

By January 2021, the lineages B.1.1.7 and B.1.351 were already widely spread across Eastern Africa and Africa but there were only sporadic detections in Coastal Kenya (Figure 5A–D). Waves 1 and 2 Coastal Kenya predominant lineage B.1 occurred in substantial proportions across the different scales early in the pandemic (Wave 1), but its prevalence elsewhere outside Kenya diminished faster overtime compared to the Kenya sample. Greater than 95% (909/950) of the lineages comprising infections in the global subsample (March 1, 2020, and February 28, 2021) were not seen in the Coastal Kenya samples (Supplementary file 5). The global pattern of detection of the 43 locally detected lineages is shown in Figure 5—figure supplement 2. Only two lineages in the Coastal Kenya sampling were not in the global subsample; lineage N.8 and lineage B.1.593 (Figure 5—figure supplement 2).

### SARS-CoV-2 genetic diversity in Coastal Kenya

**Figure 6.**
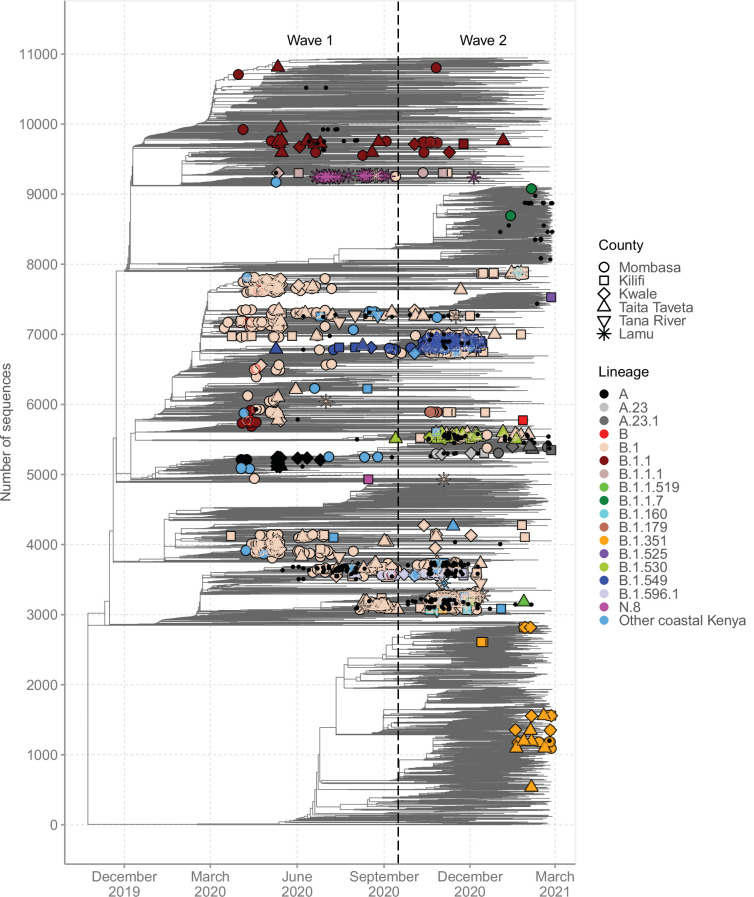
Global context of severe acute respiratory syndrome coronavirus 2 (SARS-CoV-2) diversity observed in Coastal Kenya. A time-resolved global phylogeny that combined 1139 Coastal Kenya SARS-CoV-2 genomes and 9906 global reference sequences. Distinct shapes are used to identify the different Coastal Kenya counties and distinct colours to identify the different lineages. Lineages detected in less than four cases were put together and referred to as ‘other Coastal Kenya lineages’. This group comprises 26 lineages, namely, A.25, B.1.1.33, B.1.1.464, B.1.177.6, B.1.201, B.1.212, B.1.222, B.1.281, B.1.284, B.1.340, B.1.390, B.1.393, B.1.396, B.1.413, B.1.416, B.1.433, B.1.450, B.1.480, B.1.535, B.1.558, B.1.593, B.1.596, B.1.609, B.1.629, B.4, and B.4.7. Sequences not fitting clock-like molecular evolution were removed using TreeTime program (Sagulenko et al., 2018). The analysis included 292 genomes obtained from samples collected in Kenya but outside coastal counties and these are shown as a small, solid black circles.

A time-resolved ML phylogeny for the Coastal Kenya genomes with global subsample in the background is provided in Figure 6. This phylogeny showed that (1) the Coastal Kenya genomes were represented across several but not all of the major phylogenetic clusters, (2) some of the Coastal Kenya clusters mapped into known Pango lineages, some of which appeared to expand after introduction, and (3) all six coastal counties appeared to have each had multiple virus introductions with some of the clusters comprising genomes detected across multiple counties (Figure 6). Many of the lineages identified in Coastal Kenya formed monophyletic groups (e.g. A, B.1.549, B.1.530, and N.8) with a few exceptions like lineage B.1, B.1.1, and B.1.351 which occurred on the phylogeny as multiple clusters. The data we analysed showed considerable correlation between the root-to-tip genetic distance and the sampling dates of the genomes (R^2^ = 0.604; Figure 6—figure supplement 1).

**Figure 6—figure supplement 1.**
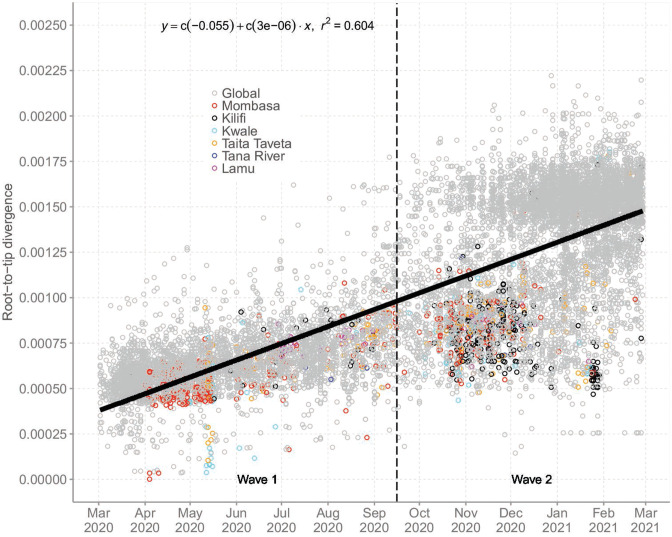
Root-to-tip regression analysis of Coastal Kenya genomes combined with global sequences.

**Figure 6—figure supplement 2.**
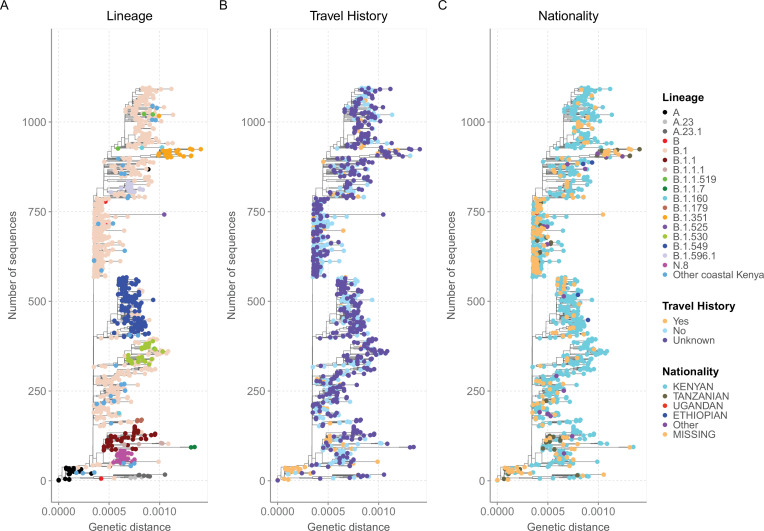
Mutation-resolved maximum likelihood (ML) phylogeny of the 1139 Coastal Kenya genomes annotated with available epidemiological information. (**A**) The phylogenetic relationships of the sequenced genomes with the taxa colored by the identified lineages. (**B**) The phylogenetic relationships of the sequenced genomes with the taxa colured by the travel history of the cases. (**C**) The phylogenetic relationships of the sequenced genomes with the taxa colored by the nationality of the cases.

We found that sequences from individuals reporting recent travel (n = 119) occurred throughout the local phylogeny based on the clustering of the Coastal Kenya genomes (Figure 6—figure supplement 2). Recent travellers infected with lineage B.1 (n = 60, 8%) were spread throughout the phylogeny and were captured in all the six counties of Coastal Kenyan counties. Contrastingly, individuals reporting recent travel and infected with lineage A (n = 19, 86%) and some of the lineage B.1.1 (n = 10, 18%)-infected cases clustered, suggesting a potential common infection source/origin for these lineages. Viral sequences from Kenyan nationals were spread across the tree structure. One striking exception was lineage A-infected cases whose nationality was frequently recorded as missing, but majority were travellers.

**Figure 7.**
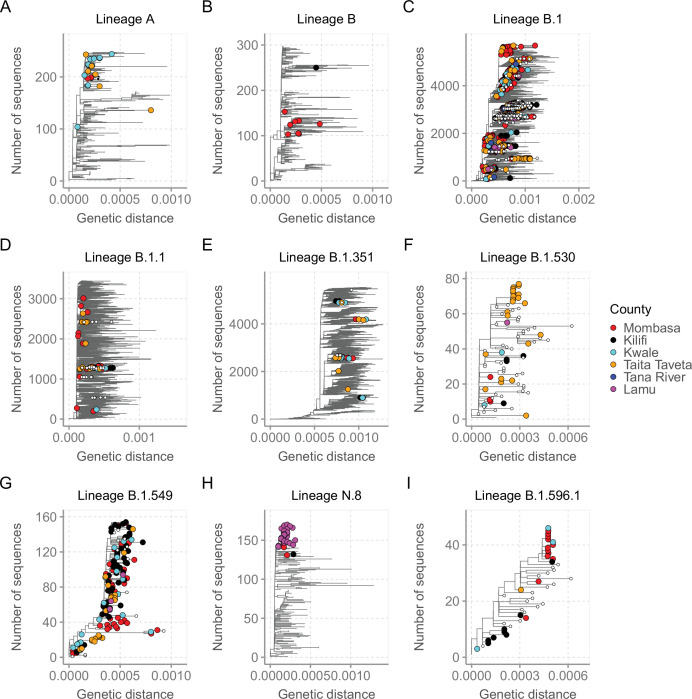
Mutation-resolved lineage-specific phylogenies for the top nine lineages detected in Coastal Kenya. The Coastal Kenya genomes are indicated with filled different shapes for the different counties. Genomes from other locations within Kenya are indicated with small solid black circles. (**A**) Phylogeny of the 22 lineage A Coastal Kenya genome combined 240 global lineage A sequences. (**B**) Phylogeny of the lineage B that combined 9 Coastal Kenya genomes and 291 global lineage B sequences. (**C**) Phylogeny for lineage B.1 that combined 723 Coastal Kenya genomes and 5136 global lineage B.1 sequences. (**D**) Phylogeny for lineage B.1.1 that combined 57 Coastal Kenya genomes and 3451 global lineage B.1.1sequences. (**E**) Phylogeny for lineage B.1.351 that combined 26 Coastal Kenya genomes and 5613 global lineage B.1.351 sequences. (**F**) Phylogeny for lineage B.1.530 that combined 32 Coastal Kenya genomes and 45 global lineage B.1.530 sequences. (**G**) Phylogeny for lineage B.1.549 that combined 143 Coastal Kenya genomes and 14 lineage B.1. 549 sequences from other locations. (**H**) Phylogeny for lineage N.8 that combined 31 Coastal Kenya genomes of lineage N.8, a single Coastal Kenya genomes of lineage B.1.1.33 and 139 lineage B.1.1.33 global sequences. (**I**) Phylogeny for lineage B.1.596.1 that combined 24 Coastal Kenya genomes and 22 lineage B.1.596.1 global sequences.

For detailed investigation into the local SARS-CoV-2 genetic diversity, we reconstructed mutation-resolved phylogenies for the top nine lineages in Coastal Kenya (Figure 7, and corresponding time-resolved phylogenies presented in Figure 7—figure supplement 1). We observed (1) considerable within-lineage diversity (highest in the predominant lineage B.1), (2) formation of multiple subclusters within these lineages, with some of clusters being county-specific (e.g. cluster of Taita Taveta sequences observed in lineage B.1.530; Figure 7F), and (3) scenarios of local sequences interspersed with global comparison genomes from the same lineage implying multiple import events of these lineages into Kenya, for example, for lineages A, B, B.1, B.1.1, and B.1.351 (Figure 7A–E). Of the four lineages that appeared to be Kenya specific, three (B.1.530, B.1.549, and B.1.596.1) had representation in other parts of Kenya outside of the coastal counties with formation of multiple genetic subclusters (Figure 7F and I). However, lineage N.8, which was mainly detected in Lamu, formed a single monophyletic group (Figure 7H) when co-analysed with its precursor lineage B.1.1.33.

**Figure 7—figure supplement 1.**
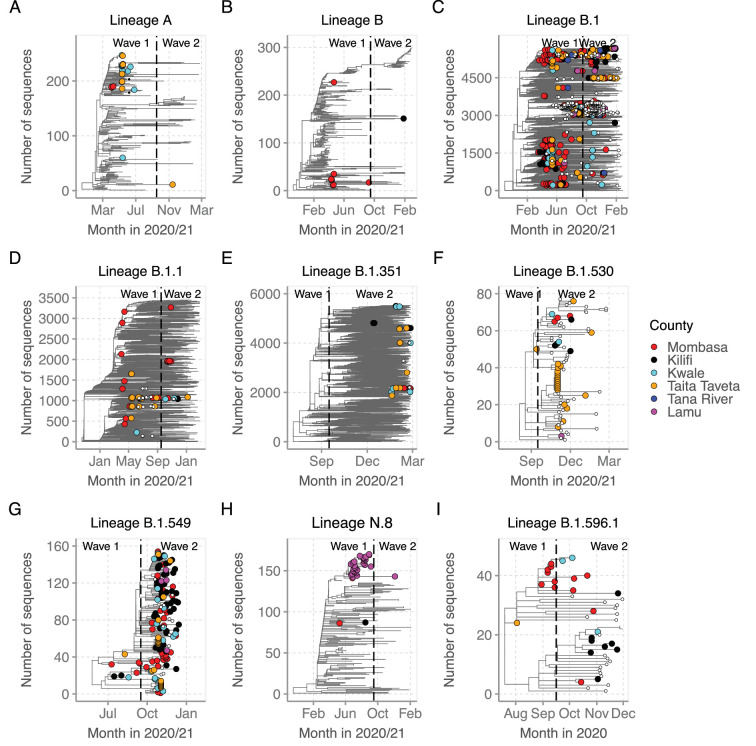
Time-resolved lineage-specific phylogenetic trees for top nine lineages detected in Coastal Kenya. (**A**) Phylogeny for lineage A that combined 22 Coastal Kenya genomes and 240 global sequences belonging also to lineage A. (**B**) Phyogeny for lineage B that combined 9 Coastal Kenya genomes and 291 global sequences also belonging to lineage B. (**C**) Phylogeny for lineage B.1 that combined 723 Coastal Kenya genomes and 5136 global sequences beloging also to lineage B.1. (**D**) Phylogeny for lineage B.1.1 that cimbined 57 coastal enya genomes and 3451 glonal sequences also belonging to lineage B.1.1. (**E**) Phylogeny for lineage B.1.351 that combined the 26 Coastal Kenya genomes and 5613 global sequences beloging to also lineage B.1.351. (**F**) Phylogeny for lineage B.1.530 that combined 32 Coastal Kenya genomes and 45 global sequences belonging to also lineage B.1.530. (**G**) Phylogeny of lineage B.1.549 that combined the 143 Coastal Kenya genomes and 14 global sequences from other locations belonging to also lineage B.1.549. (**H**) Phylogeny of lineage N.8 that combined 31 Coastal Kenya genomes of lineage N.8, 1 Coastal Kenya genome of lineage B.1.1.33 and 139 global genome sequences belonging to lineage B.1.1.33. (**I**) Phylogeny for lineage B.1.596.1 that combined 24 Coastal Kenya genomes and 22 global sequences that also belonged to lineage B.1.596.1.

**Figure 7—figure supplement 2.**
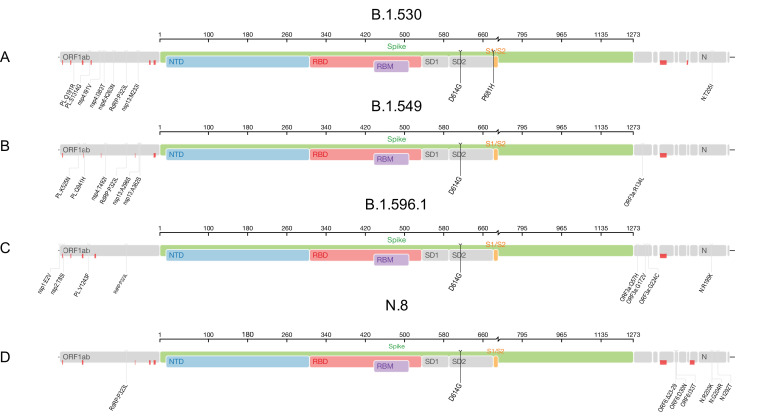
Genome maps of the ‘Kenya’ lineages where the spike region is shown in detail and in colour and the rest of the genome is shown in grey colour. (**A**) Pango lineage B.1.530. (**B**) Pango lineage B.1.549. (**C**) Pango lineage B.1.596.1. (**D**) Pango lineage N.8. Red marks throughout the four panels indicate where sequencing of Kenya strains resulted in ambiguous nucleotides. Plots generated using tool available from Stanford University Web page Coronavirus antiviral and resistance database https://covdb.stanford.edu/page/mutation-viewer/: last accessed on August 3, 2021.

### Imports and exports from Coastal Kenya

**Figure 8.**
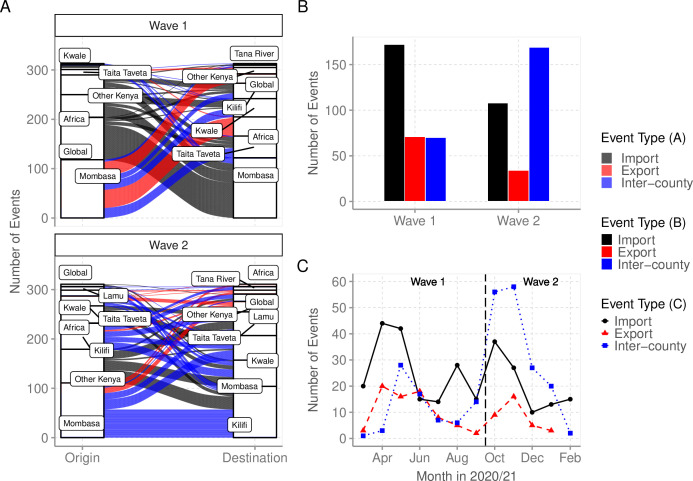
Virus importations and exportations from Coastal Kenya. (**A**) Alluvium plots stratified by wave number showing the estimated number and flow of importations into and exportations from Coastal Kenya. ‘Global’ refer to origins or destinations outside Kenya while ‘Other Kenya’ refer to origins or destinations within Kenya but outside the Coastal Counties. (**B**) The raw counts bar plot of location transition events observed within and between Coastal Kenya outside world shown as either virus exportations, importations, or inter-county transmission, these stratified by wave number. (**C**) Monthly trends of the observed transition events stratified by type. The findings presented in this figure are based on subsample 1. Figure 8—source data 1.The number of importation and exportation events by county and wave period. Figure 8—source data 2.The number of importations, inter-county transmission, and exportation events by month.

We used ancestral location state reconstruction of the dated phylogeny (Figure 6) to infer virus import and export (Sagulenko et al., 2018). By this approach, a total of 280 and 105 virus importation and virus exportation events were detected, respectively (Table 4), and distributed between the waves as summarized in Figure 8A and B. Virus importations and exportations into the region occurred predominantly through Mombasa (n = 140, 50%) and (n = 85, 81%), respectively. However, relative to its population size, Mombasa was second to Taita Taveta in importation rate per 100,000 people (Table 4). The majority of the international importation events we detected occurred during Wave 1 (Figure 8B). For the detected 105 virus exportations, 71 (68%) occurred during Wave 1 and 34 (32%) during Wave 2 (Figure 8A and B). We repeated the analysis using the second global subsample with a normalized subsample of the Coastal Kenya genomes accounting for total reported infections per county. The reanalysis found closely aligned results to those revealed by subsample1 (Supplementary file 6).

**Figure 8—figure supplement 1.**
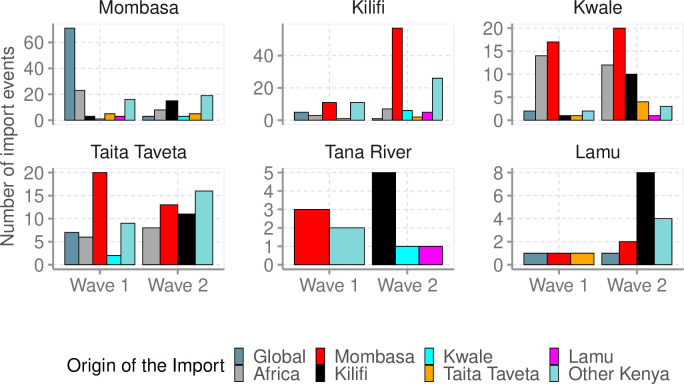
The number of import events, stratified by wave period into the individual coastal counties and their estimated origins.

**Table 4.** Summary of import and export events and rates into coastal counties populations.

County	Virus import (%)	Import rate (per 100,000)*	Virus export (%)	Export rate (per 100,000)*
Mombasa	140 (50)	11.6	85 (81)	7.0
Kilifi	53 (19)	3.6	4 (4)	0.3
Kwale	33 (12)	3.8	4 (4)	0.5
Taita Taveta	46 (16)	13.5	12 (11)	3.5
Tana River	2 (<1)	0.6	-	-
Lamu	6 (2)	4.1	-	-
Overall	280	6.7	105	2.4

* Denominator population as per the 2019 national census (see Table 1).

### Viral circulation between counties of Coastal Kenya

**Figure 9.**
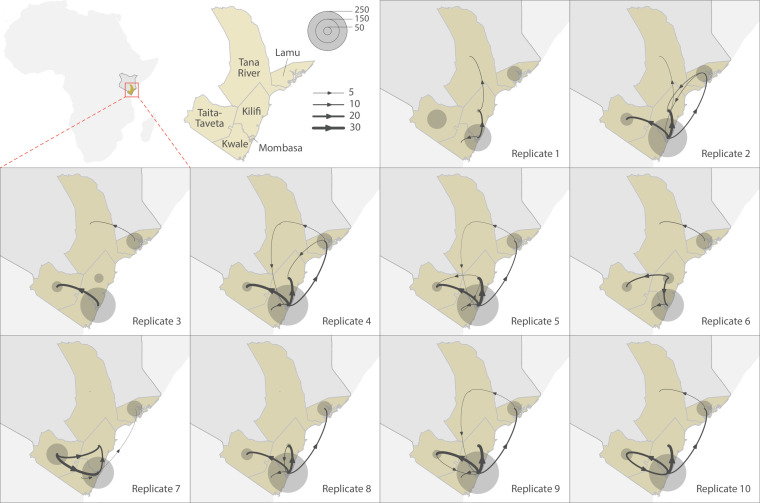
Replicated discrete phylogeographic reconstructions of the circulation of severe acute respiratory syndrome coronavirus 2 (SARS-CoV-2) lineages within and among counties of Coastal Kenya. Each replicated analysis was based on a random subset of genomic sequences subsampled according to local incidence (see the ‘Methods’ section for further detail). We here report the number of lineage dispersal events inferred among (arrows) and within (transparent grey circles) counties, both measures being averaged over posterior trees sampled from each posterior distribution. We here only report among-counties transition events supported by adjusted Bayes factor (BF) values >20, which corresponds to a strong support according to the scale of BF values interpretation of Kass and Raftery, 1995.

To explore the pattern of viral circulation within and among counties of Coastal Kenya, we conducted replicated discrete phylogeographic analyses based on random subsets of genomic sequences subsampled according to local incidence (Figure 9). We observe notable differences among the reconstructions of viral lineage dispersal history obtained from the 10 replicated analyses, meaning that the phylogeographic outcome is quite sensitive to the sampling pattern. However, if we look at the similarities among those replicated phylogeographic reconstruction, we can observe that Mombasa tended to act as an important hub associated with relatively important viral circulation and at the origin of numbers of viral dispersal events toward surrounding counties.

## Discussion

We report patterns of SARS-CoV-2 introduction and spread in Coastal Kenya during Waves 1 and 2, and estimate approximately 300 independent virus introductions occurred, many in the first six months of the pandemic. Given the limited diagnostic testing capacity and the relatively small number of samples sequenced, it is likely that there were more introductions than calculated here.

Multiple virus introductions occurred even at the county level, with inter-county spread predominating Wave 2. A lockdown was put in place for Mombasa, Kilifi, and Kwale in April 2020 and was later lifted on June 7, 2020, allowing mixing of the population and potential virus spread. It is notable that most imports into and exports from the Coastal Region probably passed through Mombasa, a major commercial, industrial, and tourist destination. This observation highlights the need for continuous and systematic surveillance of lineages circulating in Mombasa timely knowledge of variants entering or circulating within Coastal Kenya.

During Wave 1, we detected 23 Pango lineages in Coastal Kenya with lineage B.1 accounting for 73% of the sequenced infections. B.1 was detected in all counties of Coastal Kenya and was considerably diverse. Lineage B.1 dominance may have been in part driven by the possession of the D614G change in the spike protein, which has been found to enhance viral fitness (Baric, 2020). The strict quarantine and isolation of confirmed cases in the early period may have prevented some of the other lineages introduced from widely spreading, for example, lineage A was limited to travellers.

Lineage N.8 was specific to Lamu County with only three cases recorded elsewhere in Coastal Kenya and three cases elsewhere in Kenya. Lineage N.8 precursor (lineage B.1.1.33) was observed earlier in Brazil. The occurrence of lineage N.8 in Lamu may have arisen from its direct introduction from outside Kenya or introduction as B.1.1.33 followed by local evolution. Determining the exact origin of this lineage is complicated by the sparse genomic surveillance elsewhere Kenya during the study period and indeed for many regions across the world. The N.8 lineage has seven characteristic lineage defining mutations including S: D614G and N: R203K, N: G204R, and N: I292T (Figure 7—figure supplement 1).

During Wave 2, Kilifi, Tana River, and Kwale observed their first major wave of infections. This wave started when most of the government COVID-19 restriction measures had been lowered or removed. For instance, international flights resumed on August 1, 2020, the operation of bars had resumed in September 2020, phased reopening of schools started in October 2020, and the curfew hours were moved to from 11 pm to 4 am. A total of 29 lineages were detected in Coastal Kenya during Wave 2, 9 of these had also been earlier detected during Wave 1.

Genomic data on GISAID database indicated that lineages B.1.530, B.1.549, and B.1.596.1 were predominantly detected in Kenya. The first sequenced cases of all these three lineages were identified in Taita Taveta County but the travel history of these individuals was indicated as ‘unknown’. These lineages may have arisen in Kenya or another East Africa location that had limited genomic surveillance, for example, in Tanzania. Lineage B.1.530 has six characteristic mutations including spike P681H change adjacent to the biologically important furin cleavage site, lineage B.1.549 has seven characteristic mutations, five occurring in the ORF1a or ORF1b while lineage B.1.596.1 has eight lineage defining mutation 3 in ORF6 and three in N protein (Figure 7—figure supplement 1).

Three of the four Kenya-specific lineages were later observed in other countries albeit in small numbers. Lineage B.1.530 was detected in seven countries, namely, Germany (n = 3), the USA (n = 3), Rwanda (n = 1), Australia (n = 1), Japan (n = 1), and the Netherlands (n = 1). Lineage B.1.549 was detected in four countries, namely, England (n = 20), the USA (n = 4), Madagascar (n = 3), and Canada (n = 1). Lineage B.1.596.1 was detected in six countries, namely, the USA (n = 21), Sweden (n = 12), Australia (n = 2), Fiji (n = 1), Finland (n = 1), and India (n = 1). Note that the ancestral location state reconstruction analysis detected up to 105 virus exportation events from the Coastal Kenya counties to the rest of the world.

Lineage B.1.351 was first detected in Kilifi in November 2020 in a local with no travel history and later in two asymptomatic international travellers of South Africa nationality. Lineage B.1.1.7 was detected in a local who presented to a Mombasa clinic in the second week of January 2021 and in the subsequent weeks up to the end of the period covered by this analysis (February 2021), only one additional B.1.1.7 case was detected unlike lineage B.1.351, which continued to be detected sporadically in January and February 2021. Overall, only a minor increase in cases was observed in January–February 2021, despite the arrival of these VOCs before they subsequently resulted in the third national wave of infection recorded March–April 2021.

Despite the very large number of lineages detected globally (>900) during our study period, only a small fraction (n = 41, <5%) of these were documented in Coastal Kenya (O’Toole et al., 2021). Notably, two VOC lineages were already extensively spread across Eastern Africa (B.1.351), Africa (B.1.351), and worldwide (B.1.1.7) in the last quarter of 2020 unlike for Coastal Kenya. Thus, it is interesting that whereas in some countries (e.g. South Africa) the second wave appeared to be majorly driven by emergence of new variants, in Coastal Kenya, this may not have been the case. A lag was observed in the VOC large-scale spread in Coastal Kenya perhaps due to its remoteness and public health measures in place during the period.

Our study contributes to improved understanding on SARS-CoV-2 introduction and transmission patterns in sub-Saharan Africa countries (Bugembe et al., 2020; Butera et al., 2021; Githinji et al., 2021; Mashe et al., 2021; Wilkinson et al., 2021). This knowledge has potential to inform the application of future mitigation strategies especially in light of the growing evidence that SARS-CoV-2 will be endemic in human populations (Planas et al., 2021). Our analysis reveals lineage prevalence patterns and routes of entry into Coastal Kenya. New variants were frequently introduced via Mombasa County, thus surveillance in the city may provide an early warning system of new variant introductions into the region. We also provide evidence that the first two waves of infection in Coastal Kenya were not driven by VOCs, indicating the presence of other important factors impacting and driving SARS-CoV-2 waves of infection.

Sampling bias is a limitation as (1) sequenced and non-sequenced samples differed significantly in the demographic characteristics, (2) only a small proportion of confirmed cases (<10%) were sequenced, prioritizing samples with a Ct value of <30.0, (3) the MoH case identification protocols were repeatedly altered as the pandemic progressed (Githinji et al., 2021), and (4) sampling intensity across the six coastal counties due to accessibility differences. This may have skewed the observed lineage and phylogenetic patterns. There was considerable missingness in metadata (e.g. travel history, nationality, Table 2), which made it hard to integrate genomic and epidemiological data in an analysis. Due to amplicon drop-off, some of the analysed genomes were incomplete impacting the overall phylogenetic signal.

The accuracy of the inferred patterns of virus movement into and from Coastal Kenya is dependent on both the representativeness of our sequenced samples for Coastal Kenya and the comprehensiveness of the comparison data from outside Coastal Kenya. Our sequenced sample was proportional to the number of positive cases reported in the respective Coastal Kenya counties. Also, we carefully selected comparison data to optimize chances of observing introductions occurring into the coastal region (e.g. by using all Africa data). But still there remained some important gaps, for example, non-coastal Kenya genomic data was limited (n = 605). Despite this, we think the results from ancestral state reconstruction indicate that Mombasa is a major gateway for variants entering Coastal Kenya is consistent with (1) the county showing the highest number lineages circulating during the study period compared to the other five remaining coastal counties Kenya, (2) approximately half of the detected lineages in Coastal Kenya had their first case identified in Mombasa, (3) Mombasa had an early wave of infections compared to the other coastal counties, and (4) Mombasa is the most well-connected county in the region to the rest of the world (large international seaport and airport and major railway terminus and several bus terminus).

In conclusion, we show that the first two SARS-CoV-2 waves in Coastal Kenya observed transmission of both newly introduced and potentially locally evolved lineages, many of them being non-VOCs. Approximately 50% of lineage introductions into the region occurred through Mombasa City. Our findings are consistent with mathematical modelling conclusion that it is more likely that relaxation or removal of some of the government COVID-19 countermeasures could have facilitated the second wave of SARS-CoV-2 infections in Kenya (Brand et al., 2021). Based on our observations of local distinctive phylogenies and the predominance of inter-county transmission, we suggest focusing COVID-19 control strategies on local transmission rather than international travel.

## Data Availability

(1) Sequence data have been deposited in GISAID database under accession numbers provided in Supplement File 2. (2) Source Data files have been provided for Figures 1-2 and 4-10. (3) Source Code associated with the figures has been uploaded (Source Code File 1) and also been made available through Harvard Dataverse. The following dataset was generated: AgotiCN 2021Replication Data for: Genomic surveillance reveals the spread patterns of SARS-CoV-2 in coastal Kenya during the first two wavesHarvard Dataverse10.7910/DVN/4ZZYIM The following previously published dataset was used: GithinjiG 2021Genomic epidemiology of SARS-CoV-2 in coastal Kenya (March - July 2020)Github8402936

## Additional files

Supplementary file 1. Kenya government public health response and intervention to the COVID-19 pandemic.

Supplementary file 2. Details on GISAID accession IDs, county of sampling, date of sample collection, assigned lineage, assigned clade, and the nucleotide sequences of the presented 1139 Coastal Kenya severe acute respiratory syndrome coronavirus 2 (SARS-CoV-2) genomes.

Supplementary file 3. History of lineages detected in Coastal Kenya during the study period.

Supplementary file 4. Patterns of Pango lineage detection at the various scales of observation analysed.

Supplementary file 5. A summary of the top 10 detected Pango lineages detected in the different scales of observation investigated.

Supplementary file 6. Summary output from separate runs of the import/export ancestral state reconstruction (ASR) analysis.

Supplementary file 7. Acknowledgement of investigators and laboratories that have deposited genomic data into GISAID database that we used to place the Coastal Kenya genomes into the global context.

Transparent reporting form

Reporting standard 1. STROBE Checklist.

Source code 1. The R scripts used in the generation of the main text figures presented in the article.
