# Supplementary material for: Transmission networks of SARS-CoV-2 in Coastal Kenya during the first two waves: A retrospective genomic study
Source: eLife. 2022 Jun 14;11:e71703. doi: 10.7554/eLife.71703 (PMC9282859; doi:10.7554/eLife.71703)
Supplement: Supplementary file 1. [file elife-71703-supp1.docx]

**Supplementary File 1.** Kenya Government Response

| Date of effect | Intervention type | Intervention |
| --- | --- | --- |
| 2020-03-15 | Restriction | Restriction of travel from countries with COVID-19 cases; all private and public sector workers directed to work from home wherever possible. |
| 2020-03-20 | Restriction | Schools and higher learning institutions closed. |
| 2020-03-22 | Restriction | All bars closed; restaurants allowed to remain open for take-away services only; public transportation capacity reduced. |
| 2020-03-25 | Restriction | 7 p.m to 5 a.m. national curfew announced; international flights suspended with the exception of cargo flights |
| 2020-04-06 | Restriction | Cessation of movement in Nairobi, Mambasa, Kilifi and Kwale county except for food supplies and other cargo. |
| 2020-04-19 | Public health measure | Facemasks mandated in public spaces. |
| 2020-05-06 | Restriction | Cessation of movement in and out of Old town (Mvita) in Mombasa and Eastleigh area in Nairobi. |
| 2020-06-07 | Relaxation | Lockdown lifted in Mombasa, Mandera and Nairobi. |
| 2020-06-10 | Public health measure | Government launches home-based care for patients. |
| 2020-07-15 | Relaxation | Reopening local air travel within territory. |
| 2020-08-01 | Relaxation | International flights resumed. |
| 2020-09-27 | Relaxation | Bars and restaurants reopened with capacity limits. |
| 2020-10-05 | Relaxation | Phased reopening of learning institutions; grade 4, class 8 and form 4’s reported back to school. |
| 2020-11-04 | Restriction | Bars and restaurants close by 9pm, public rallies suspended. |
| 2020-12-06 | Other occurrences | Health workers strike starts. |
| 2021-01-04 | Relaxation | All schools reopened. |
