# Supplementary material for: Transmission networks of SARS-CoV-2 in Coastal Kenya during the first two waves: A retrospective genomic study
Source: eLife. 2022 Jun 14;11:e71703. doi: 10.7554/eLife.71703 (PMC9282859; doi:10.7554/eLife.71703)
Supplement: Supplementary file 3. [file elife-71703-supp3.docx]

**Supplementary File 2:** History of lineages detected in Coastal Kenya during the study period.

| Lineage | County first detected | First-identified Case Travel History | Study Phase | Date of First Detection |
| --- | --- | --- | --- | --- |
| B.1 | Mombasa | Yes | Wave 1 | 2020-03-17 |
| B.1.1 | Mombasa | No | Wave 1 | 2020-04-01 |
| A | Mombasa | No | Wave 1 | 2020-04-04 |
| B.4 | Mombasa | Unknown | Wave 1 | 2020-04-04 |
| B.4.7 | Mombasa | Unknown | Wave 1 | 2020-04-04 |
| B.1.340 | Mombasa | Yes | Wave 1 | 2020-04-07 |
| B.1.222 | Mombasa | Unknown | Wave 1 | 2020-04-09 |
| B | Mombasa | No | Wave 1 | 2020-04-11 |
| B.1.535 | Mombasa | No | Wave 1 | 2020-04-28 |
| B.1.1.33 | Mombasa | No | Wave 1 | 2020-05-11 |
| B.1.549 | Taita Taveta | Unknown | Wave 1 | 2020-05-11 |
| B.1.1.1 | Kwale | Yes | Wave 1 | 2020-05-13 |
| B.1.416 | Mombasa | No | Wave 1 | 2020-06-21 |
| N.8 | Lamu | Unknown | Wave 1 | 2020-06-23 |
| B.1.177.6 | Kilifi | Unknown | Wave 1 | 2020-06-27 |
| A.25 | Mombasa | No | Wave 1 | 2020-07-06 |
| B.1.396 | Kilifi | No | Wave 1 | 2020-07-11 |
| B.1.413 | Tana River | Unknown | Wave 1 | 2020-07-28 |
| B.1.558 | Mombasa | No | Wave 1 | 2020-08-01 |
| B.1.596.1 | Taita Taveta | Unknown | Wave 1 | 2020-08-03 |
| B.1.393 | Mombasa | Yes | Wave 1 | 2020-08-18 |
| B.1.593 | Tana River | Unknown | Wave 1 | 2020-08-27 |
| B.1.530 | Taita Taveta | Unknown | Wave 1 | 2020-09-15 |
| B.1.1.464 | Kwale | No | Wave 2 | 2020-10-05 |
| B.1.596 | Kwale | Unknown | Wave 2 | 2020-10-05 |
| B.1.160 | Kilifi | Yes | Wave 2 | 2020-10-21 |
| B.1.179 | Mombasa | Unknown | Wave 2 | 2020-10-21 |
| B.1.1.519 | Kilifi | No | Wave 2 | 2020-10-23 |
| B.1.281 | Kilifi | Yes | Wave 2 | 2020-10-27 |
| A.23 | Mombasa | No | Wave 2 | 2020-10-28 |
| B.1.609 | Kilifi | Yes | Wave 2 | 2020-10-28 |
| B.1.390 | Mombasa | Unknown | Wave 2 | 2020-10-29 |
| B.1.629 | Mombasa | No | Wave 2 | 2020-10-29 |
| B.1.212 | Kilifi | No | Wave 2 | 2020-11-04 |
| B.1.351 | Kilifi | No | Wave 2 | 2020-11-04 |
| B.1.201 | Lamu | Unknown | Wave 2 | 2020-11-05 |
| B.1.480 | Taita Taveta | Unknown | Wave 2 | 2020-11-15 |
| B.1.284 | Mombasa | Unknown | Wave 2 | 2020-11-18 |
| B.1.450 | Kilifi | Unknown | Wave 2 | 2020-11-27 |
| B.1.433 | Kwale | Unknown | Wave 2 | 2020-12-03 |
| A.23.1 | Mombasa | No | Wave 2 | 2021-01-02 |
| B.1.1.7 | Mombasa | Unknown | Wave 2 | 2021-01-14 |
| B.1.525 | Kilifi | Unknown | Wave 2 | 2021-02-26 |
