# Supplementary material for: Transmission networks of SARS-CoV-2 in Coastal Kenya during the first two waves: A retrospective genomic study
Source: eLife. 2022 Jun 14;11:e71703. doi: 10.7554/eLife.71703 (PMC9282859; doi:10.7554/eLife.71703)
Supplement: Supplementary file 4. [file elife-71703-supp4.docx]

**Supplementary File 3.** Patterns of Pango lineage detection at the various scales of observation analysed.

| Scale of observation | # Total genomes ANALYSED (n) | Total Pango lineages IDENTIFIED | Number of Pango Lineages SHARED with Coastal Kenya | Number of Pango Lineages NOT in Coastal Kenya | Number of Pango Lineages in Coastal Kenya ONLY |
| --- | --- | --- | --- | --- | --- |
| Coastal Kenya alone | 1,139 | 43 | - | - | - |
| Kenya, without coastal Kenya | 605 | 33 | 19 | 14 | 24 |
| Eastern Africa, without coastal Kenya | 4,136 | 125 | 24 | 101 | 19 |
| Africa, without coastal Kenya | 19,010 | 337 | 30 | 307 | 13 |
| Global, without Africa | 19,993 | 843 | 34 | 809 | 9 |
| Global, without coastal Kenya | 39,003 | 950 | 41 | 909 | 2 |
