# Supplementary material for: Transmission networks of SARS-CoV-2 in Coastal Kenya during the first two waves: A retrospective genomic study
Source: eLife. 2022 Jun 14;11:e71703. doi: 10.7554/eLife.71703 (PMC9282859; doi:10.7554/eLife.71703)
Supplement: Supplementary file 5. [file elife-71703-supp5.docx]

**Supplementary File 4.** A summary of the top 10 detected Pango lineages detected in the different scales of observation investigated

| Lineage | Coastal Kenya | Kenya, non-Coastal | Eastern Africa, non-Kenya | Africa, non-Eastern Africa | non-Africa | Total |
| --- | --- | --- | --- | --- | --- | --- |
| A | 22 | 3 | 21 | 134 | 82 | 262 |
| A.23 | 4 | 9 | 74 | 2 | - | 89 |
| A.23.1 | 6 | 17 | 252 | 27 | 30 | 332 |
| B | 9 | - | 7 | 92 | 192 | 300 |
| B.1 | 723 | 329 | 283 | 1850 | 2683 | 5868 |
| B.1.1 | 57 | 39 | 341 | 947 | 2076 | 3460 |
| B.1.1.519 | 4 | 7 | - | 1 | 60 | 72 |
| B.1.1.7 | 2 | 72 | 39 | 751 | 1395 | 2259 |
| B.1.160 | 5 | - | 67 | 112 | 564 | 748 |
| B.1.351 | 26 | 13 | 1122 | 4389 | 89 | 5639 |
| B.1.525 | 1 | 4 | 9 | 447 | 7 | 468 |
| B.1.530 | 32 | 44 | 1 | - | - | 77 |
| B.1.549 | 143 | 14 | - | - | - | 157 |
| B.1.596.1 | 24 | 20 | - | - | 2 | 46 |
| N.8 | 31 | - | - | - | - | 31 |
| B.1.177 | - | - | 146 | 66 | 640 | 852 |
| B.1.351.2 | - | - | 201 | 332 | 3 | 536 |
| B.1.380 | - | - | 126 | - | - | 126 |
| B.1.1.241 | - | - | 83 | 3 | 14 | 100 |
| B.1.1.448 | - | - | 18 | 420 | 1 | 439 |
| C.1 | - | - | 16 | 353 | - | 369 |
| B.1.416 | 2 | - | - | 301 | 12 | 315 |
| B.1.258 | - | - | 5 | 10 | 512 | 527 |
| B.1.36 | - | - | 2 | 6 | 290 | 298 |
| B.1.2 | - | - | 2 | 43 | 277 | 322 |
| B.1.221 | - | - | 1 | 4 | 262 | 267 |
| B.1.1.70 | - | - | - | 7 | 243 | 250 |
| Other Lineages | 48 | 34 | 715 | 4577 | 10559 | 15933 |
| Total | 1139 | 605 | 3531 | 14874 | 19993 | 40142 |
