# Supplementary material for: Transmission networks of SARS-CoV-2 in Coastal Kenya during the first two waves: A retrospective genomic study
Source: eLife. 2022 Jun 14;11:e71703. doi: 10.7554/eLife.71703 (PMC9282859; doi:10.7554/eLife.71703)
Supplement: Supplementary file 6. [file elife-71703-supp6.docx]

**Supplementary File 5.** Summary output from separate runs of the import/export ancestral state reconstruction (ASR) analysis

| Characteristic | Run 1^π^ | Run 2^∑^ | Run 3^#^ | Run 4^£^ | Run 5^$^ |
| --- | --- | --- | --- | --- | --- |
| Coastal Kenya analysed  (Global) | 1,139 (9,906) | 1,139 (9906) | 732 (3,958) | 728 (3,968) | 338 (3,979) |
| Total Location transition events | 624 | 605 | 357 | 341 | 217 |
| Virus imports |  |  |  |  |  |
| Total | 280 | 243 | 69 | 65 | 79 |
| Thr’ Mombasa | 140 | 121 | 40 | 38 | 36 |
| Through Kwale | 33 | 28 | 9 | 5 | 17 |
| Thr’ Taita Taveta | 46 | 46 | 13 | 13 | 20 |
| Thr’ Kilifi | 53 | 42 | 5 | 5 | 17 |
| Thr’ Lamu | 6 | 5 | 1 | 1 | 1 |
| Thr’ Tana River | 2 | 1 | - | - | - |
|  |  |  |  |  |  |
| Virus exports |  |  |  |  |  |
| Total | **105** | 118 | 93 | 87 | 25 |
| Thr’ Mombasa | 85 | 93 | 86 | 81 | 22 |
| Thr’ Kwale | 4 | 4 | 6 | 3 | 1 |
| Thr’ Taita Taveta | 12 | 15 | 1 | 3 | 1 |
| Thr’ Kilifi | 4 | - | - | - | 1 |
| Inter-county transmission events | 239 | 244 | 193 | 189 | 113 |

^π^ Import/export analysis results from ASR combining 1,139 genome sequences from coastal Kenya and the comparison dataset from sub-sample one.

^∑^ Import/export analysis results from ASR combining 1,139 genome sequences from coastal Kenya and the comparison dataset from sub-sample two.

^#^Import/export analysis results from ASR combining 732 genome sequences from coastal Kenya and a random 3,958 global (outside Kenya) genomes.

^£^Import/export analysis results from ASR combining 728 genomes from coastal Kenya and 3,968 global (outside Kenya) genomes.

^$^ Import/export analysis results from ASR combining 338 genome sequences from coastal Kenya and a random 3,979 global (outside Kenya) genomes. The coastal Kenya sub-sample used for this run 5 was randomly selected aiming to be spatial-temporally representative with up to 15 genomes per county per month over the study period.
