# Supplementary material for: Transmission networks of SARS-CoV-2 in Coastal Kenya during the first two waves: A retrospective genomic study
Source: eLife. 2022 Jun 14;11:e71703. doi: 10.7554/eLife.71703 (PMC9282859; doi:10.7554/eLife.71703)
Supplement: Supplementary file 7. [file elife-71703-supp7.pdf]

We gratefully acknowledge the following Authors from the Originating laboratories responsible for obtaining the specimens, as well as the Submitting laboratories where the genome data were generated and shared via GISAID, on which this research is based.

All Submitters of data may be contacted directly via www.gisaid.org

Authors are sorted alphabetically.

Accession ID	Originating Laboratory	Submitting Laboratory	Authors
EPI_ISL_632310	1-Laboratory of Microbiology, National Reference Lab, Charles Nicolle Hospital; 2-University of Tunis ElManar, Faculty of Medicine of Tunis, LR99ES09, Tunis, Tunisia	1-Clinical and Experimental Pharmacology Lab, LR16SP02, National Center of Pharmacovigilance, University of Tunis El Manar, Tunis, Tunisia. 2-Neurodegenerative diseases and psychiatric troubles, LR18SP03, Razi Hospital, University of Tunis El Manar, Tunis, Tunisia. 3- Ministry of Health, National Observatory of New and Emerging Diseases, 1006, Tunis, Tunisia	Alia Ben Kahla; Gaies Emna; Ilhem Boutiba-Ben Boubaker; Imen Kacem; Imen Mkada; Jalila Ben Khelil; Maher Kharrat; Mouna Ben Sassi; Mouna Safer; Nissaf Ben Alaya; Riadh Daghfous; Riadh Gouider.; Salma Abid; Sameh Trabelsi; Sana Ferjani; Soumaya Rammeh
EPI_ISL_634977, EPI_ISL_635059, EPI_ISL_635060, EPI_ISL_635061, EPI_ISL_635062, EPI_ISL_683329, EPI_ISL_699655, EPI_ISL_699656, EPI_ISL_699657, EPI_ISL_707697, EPI_ISL_707698, EPI_ISL_707699, EPI_ISL_707700, EPI_ISL_707791, EPI_ISL_707792, EPI_ISL_707793, EPI_ISL_733499, EPI_ISL_733500, EPI_ISL_763065, EPI_ISL_763067, EPI_ISL_794735, EPI_ISL_794736, EPI_ISL_794737, EPI_ISL_794738, EPI_ISL_1208403			
see above	1-Laboratory of Microbiology, National Reference Lab, Charles Nicolle Hospital; 2-University of Tunis ElManar, Faculty of Medicine of Tunis, LR99ES09, Tunis, Tunisia	1-Clinical and Experimental Pharmacology Lab, LR16SP02, National Center of Pharmacovigilance, University of Tunis El Manar, Tunis, Tunisia. 2-Neurodegenerative diseases and psychiatric troubles, LR18SP03, Razi Hospital, University of Tunis El Manar, Tunis, Tunisia. 3- Ministry of Health, National Observatory of New and Emerging Diseases, 1006, Tunis, Tunisia	Alia Ben Kahla; Alia BenKahla; Asma Ferjani; Awatef El MOussi; Awatef El Moussi; Gaies Emna; Guedi Ali Barreh; Guedi Berrabeh; Habiba Ben Romdhane; Hafsia Ladhari; Hanen El Jebari; Hanen ElJebari; Houssem Guedouar; Ilhem Boutiba-Ben Boubaker; Ilhem Boutiba-Ben Boubaker.; Imen Kacem; Imen Mkada; Ines Mdini; Jalila Ben Khelil; Maher Kharrat; Manel Ben Sassi; Mouna Ben Sassi; Mouna Safer; Nissaf Ben Alaya; Riadh Daghfous; Riadh Gouider; Riadh Gouider.; Roua Ben Othman; Rouaa Ben Othman; Salma Abid; Salwa Mrabet; Sameh Trabelsi; Sana Ferjani; Sara Chammam; Sarra Chamman; Souissi Amira; Soumaya Rammeh; Zaineb Hamzaoui
EPI_ISL_640056, EPI_ISL_640078, EPI_ISL_640089, EPI_ISL_640107, EPI_ISL_640115, EPI_ISL_640117, EPI_ISL_640127, EPI_ISL_700446, EPI_ISL_700555, EPI_ISL_1040736, EPI_ISL_1040737, EPI_ISL_1040741, EPI_ISL_1040743, EPI_ISL_1040744, EPI_ISL_1040769, EPI_ISL_1040770, EPI_ISL_1040771, EPI_ISL_1040772, EPI_ISL_1040773, EPI_ISL_1040790, EPI_ISL_1040791, EPI_ISL_1040792			
see above	2 Military Hospital wc MAA	NHLS/UCT	Arash Iranzadeh; Bruna Galvao; Carolyn Williamson; Deelan Doolabh; Diana Hardie; Innocent Mudau; Kruger Marais; Lynn Tyers; Marvin Hsiao; Stephen Korsman
EPI_ISL_960130, EPI_ISL_960151	2 Military Hospital wc MAA	National Health Laboratory Service/UCT	Arash Iranzadeh; Bruna Galvao; Carolyn Williamson; Deelan Doolabh; Diana Hardie; Innocent Mudau; Kruger Marais; Lynn Tyers; Marvin Hsiao; Stephen Korsman
EPI_ISL_602622, EPI_ISL_602623, EPI_ISL_602624, EPI_ISL_602625, EPI_ISL_602626, EPI_ISL_602627, EPI_ISL_602628, EPI_ISL_602629, EPI_ISL_602630, EPI_ISL_602631, EPI_ISL_605784			
see above	AHRI-Sigal	KRISP, KZN Research Innovation and Sequencing Platform	Cele S; Gazy I; Giandhari J; Karim F; Pillay S; Sigal A; Sigl A; Sigla; Tegally H; Wilkinson E; de Oliveira T
EPI_ISL_1229367, EPI_ISL_1229368	AHRI-Sigal	KRISP, KZn Research Innovation and Sequencing Platform	Cele S; Gazy I; Giandhari J; Karim F; Pillay S; Sigal A; Tegally H; Wilkinson E; de Oliveira T
EPI_ISL_467432, EPI_ISL_467433, EPI_ISL_467434, EPI_ISL_467435, EPI_ISL_467449, EPI_ISL_467450, EPI_ISL_467451, EPI_ISL_467452, EPI_ISL_467453, EPI_ISL_467454, EPI_ISL_467455, EPI_ISL_467456, EPI_ISL_467457, EPI_ISL_467458, EPI_ISL_467459, EPI_ISL_467460, EPI_ISL_467461, EPI_ISL_467462, EPI_ISL_467463, EPI_ISL_467464, EPI_ISL_467465, EPI_ISL_467466, EPI_ISL_467467, EPI_ISL_467468, EPI_ISL_467469, EPI_ISL_467470, EPI_ISL_467471, EPI_ISL_467472, EPI_ISL_467473, EPI_ISL_467474			
see above	AMPATH-DBN	KRISP, KZN Research Innovation and Sequencing Platform	Chimukangara B; Giandhari J; Khan S; Lessells R; Mdlalose K; Pillay S; Tegally H; Wilkinson E; York D; de Oliveira T
EPI_ISL_458150	ANOUAL	ANOUAL	Azami Nawfel; Benhida Rachid; Chenaoui Mohamed; El Aliani Aissam; El Ansari Fatima Zahra; Fekkak Jamal; Jouali Farah; Kasmi Yassine; Kitane Driss Lahlou; Loukman Salma; Marchoudi Nabila
EPI_ISL_700475, EPI_ISL_700476, EPI_ISL_700521, EPI_ISL_700548, EPI_ISL_1040749	Alma CDC wc AHC	NHLS/UCT	Arash Iranzadeh; Bruna Galvao; Carolyn Williamson; Deelan Doolabh; Diana Hardie; Houriiyah Tegally; Innocent Mudau; Kruger Marais; Lynn Tyers; Marvin Hsiao; Stephen Korsman
EPI_ISL_660121, EPI_ISL_660122, EPI_ISL_660123, EPI_ISL_660124, EPI_ISL_660125, EPI_ISL_660126, EPI_ISL_660127, EPI_ISL_660128, EPI_ISL_660129			
see above	Ampath	National Health Laboratory Service (NHLS), Tygerberg	Bronwyn Kleinhans; Davis M-A; Draper C; Eduan Wilkindon; Gert van Zyl; Houriiyah Tegally; Hsiao M; Kayla Delaney; Siegfried N; Susan Engelbrecht; Tulio de Oliveira; Williamson C; Wolfgang Preiser
EPI_ISL_860553, EPI_ISL_860631	Ampath-Netcare	KRISP, KZn Research Innovation and Sequencing Platform	Giandhari J; Khan S; Lessells R; Mdlalose K; Pillay S; Tegally H; Wilkinson E; York D; de Oliveira T
EPI_ISL_700553	Beaufort West CDC wc BWC	NHLS/UCT	Arash Iranzadeh; Bruna Galvao; Carolyn Williamson; Deelan Doolabh; Diana Hardie; Innocent Mudau; Kruger Marais; Lynn Tyers; Marvin Hsiao; Stephen Korsman
EPI_ISL_640031, EPI_ISL_640047, EPI_ISL_700453	Beaufort West Hospital wc BWH	NHLS/UCT	Arash Iranzadeh; Bruna Galvao; Carolyn Williamson; Deelan Doolabh; Diana Hardie; Innocent Mudau; Kruger Marais; Lynn Tyers; Marvin Hsiao; Stephen Korsman
EPI_ISL_960141	Beaufort West Hospital wc BWH	National Health Laboratory Service/UCT	Arash Iranzadeh; Bruna Galvao; Carolyn Williamson; Deelan Doolabh; Diana Hardie; Innocent Mudau; Kruger Marais; Lynn Tyers; Marvin Hsiao; Stephen Korsman
EPI_ISL_527007	Biological Prevention, Army	Biological Prevention, Army	A.E.; A.F.; A.M.; Ageez; B.E.; Elhoseiny; Gad; Harty; M.D.; M.F.; M.G.; Seadawy; Shabaan; Shamel
EPI_ISL_526975, EPI_ISL_526976, EPI_ISL_526977, EPI_ISL_526978, EPI_ISL_526979, EPI_ISL_526980, EPI_ISL_526981, EPI_ISL_526982, EPI_ISL_526983, EPI_ISL_526984, EPI_ISL_526985, EPI_ISL_526986, EPI_ISL_526987, EPI_ISL_526988, EPI_ISL_526989, EPI_ISL_526990, EPI_ISL_526991, EPI_ISL_526992, EPI_ISL_526993, EPI_ISL_526994, EPI_ISL_526995, EPI_ISL_526996, EPI_ISL_526997, EPI_ISL_526998, EPI_ISL_526999, EPI_ISL_527000, EPI_ISL_527001, EPI_ISL_527002, EPI_ISL_527003, EPI_ISL_527004, EPI_ISL_527005, EPI_ISL_527006			
see above	Biological prevention, army	Biological prevention, army	A.E.; A.F.; A.M.; Ageez; B.E.; Elhoseiny; Elhosieny; Gad; Harty; M.D.; M.F.; M.G.; Seadawy; Shabaan; Shamel
EPI_ISL_510526, EPI_ISL_510532	Biological prevention, army	Biological prevention, army	A.A.; A.F.; A.M. and Soliman; Ali; Amer; B.E.; B.S.; ELnabrawy; ElGohary; Elhoseiny; Elhoseny; Elnagdy; Elnakeeb; Gad; H.A.; Harty; Hassan; Kandeil; Karam; M.A.; M.D.; M.F.; M.G.; M.M. and Gad; Raouf; Seadawy; Shamel; T.A.; W.A.; Y.A.; k.E.
EPI_ISL_907076, EPI_ISL_907077, EPI_ISL_907078, EPI_ISL_907079, EPI_ISL_907080, EPI_ISL_907081, EPI_ISL_907082, EPI_ISL_907083, EPI_ISL_907084, EPI_ISL_907085			
see above	Biology, MCL	Biology, MCL	A.F.; A.N.; Elhoseiny; Gad; M.D.; M.F.; M.G.; Seadawy; Shamel; Zekri
EPI_ISL_794634	Biology, MCL	Biology, MCL	A.F.; A.S.; B.S. and EL-Safty; EL-harty; EL-hosieny; Gad; M.D.; M.F.; M.G.; Seadawy; Shamel
EPI_ISL_985058, EPI_ISL_985059, EPI_ISL_985060, EPI_ISL_985061, EPI_ISL_985062, EPI_ISL_985063, EPI_ISL_985064, EPI_ISL_985065, EPI_ISL_985066, EPI_ISL_985067, EPI_ISL_985068, EPI_ISL_985069, EPI_ISL_985070, EPI_ISL_985071, EPI_ISL_985072, EPI_ISL_985073, EPI_ISL_985074, EPI_ISL_985075, EPI_ISL_985076, EPI_ISL_985077, EPI_ISL_985078, EPI_ISL_985079, EPI_ISL_985080, EPI_ISL_985081, EPI_ISL_985082, EPI_ISL_985083, EPI_ISL_985084, EPI_ISL_985085, EPI_ISL_985086, EPI_ISL_985087, EPI_ISL_985088, EPI_ISL_985089, EPI_ISL_985090, EPI_ISL_985091, EPI_ISL_985092, EPI_ISL_985093, EPI_ISL_985094, EPI_ISL_985095, EPI_ISL_985096, EPI_ISL_985097, EPI_ISL_985098, EPI_ISL_985099, EPI_ISL_985100, EPI_ISL_985101, EPI_ISL_985102, EPI_ISL_985103, EPI_ISL_985104, EPI_ISL_985105, EPI_ISL_985106, EPI_ISL_985107, EPI_ISL_985108, EPI_ISL_985109, EPI_ISL_985110, EPI_ISL_985111, EPI_ISL_985112, EPI_ISL_985113, EPI_ISL_985114, EPI_ISL_985115, EPI_ISL_985116, EPI_ISL_985117, EPI_ISL_985118, EPI_ISL_985119, EPI_ISL_985120, EPI_ISL_985121, EPI_ISL_985122, EPI_ISL_985123, EPI_ISL_985124, EPI_ISL_985125, EPI_ISL_985126, EPI_ISL_985127, EPI_ISL_985128, EPI_ISL_985129, EPI_ISL_985237, EPI_ISL_985238			
see above	Biorepository and Clinical Virology Laboratory	Ozer Lab	Adeola A. Fowotade; Babafemi O. Taiwo; Egon A. Ozer; Ewean C. Omoruyi; Johnson A. Adeniji; Judd F. Hultquist; Lacy M. Simons; Olubusuyi M. Adewumi; Ramon Lorenzo-Redondo
EPI_ISL_458287	Biosafety Department PCL3	Biosafety Department PCL3	A. and El Kabbaj, S.; Lemriss, S.; Souiri
EPI_ISL_745118, EPI_ISL_745134, EPI_ISL_745158, EPI_ISL_745159, EPI_ISL_745168, EPI_ISL_745169	Bishop Lavis CDC wc BLP	National Health Laboratory Service (NHLS), Tygerberg	Bronwyn Kleinhans; Eduan Wilkindon; Gert van Zyl; Houriiyah Tegally; Kayla Delaney; Susan Engelbrecht; Tulio de Oliveira; Wolfgang Preiser
EPI_ISL_700589	Bongolethu Clinic wc BLC	NHLS/UCT	Arash Iranzadeh; Bruna Galvao; Carolyn Williamson; Deelan Doolabh; Diana Hardie; Houriiyah Tegally; Innocent Mudau; Kruger Marais; Lynn Tyers; Marvin Hsiao; Stephen Korsman
EPI_ISL_640075	Bothasig CDC wc BLD	NHLS/UCT	Arash Iranzadeh; Bruna Galvao; Carolyn Williamson; Deelan Doolabh; Diana Hardie; Innocent Mudau; Kruger Marais; Lynn Tyers; Marvin Hsiao; Stephen Korsman
EPI_ISL_965182	Botswana Harvard AIDS Institute Partnership	Botswana Harvard AIDS Institute Partnership	Boitumelo Zuze; Botshelo Radibe; David Lawrence; Dorcas Maruapula; Joseph Makhema; Mosepele Mosepele; Roger Shapiro; Shahin Lockman; Sikhulile Moyo; Simani Gaseitsiwe; Wonderful T. Choga
EPI_ISL_965178	Botswana Harvard HIV Reference Laboratory	Botswana Harvard AIDS Institute Partnership	Boitumelo Zuze; Botshelo Radibe; David Lawrence; Dorcas Maruapula; Joseph Makhema; Mosepele Mosepele; Roger Shapiro; Shahin Lockman; Sikhulile Moyo; Simani Gaseitsiwe; Wonderful T. Choga
EPI_ISL_871872, EPI_ISL_933724, EPI_ISL_935040, EPI_ISL_935042, EPI_ISL_935045, EPI_ISL_940762, EPI_ISL_940765, EPI_ISL_940768, EPI_ISL_940776, EPI_ISL_940844, EPI_ISL_943992, EPI_ISL_944590, EPI_ISL_944756, EPI_ISL_944757, EPI_ISL_944760, EPI_ISL_944761, EPI_ISL_944767, EPI_ISL_944782, EPI_ISL_944790, EPI_ISL_949068, EPI_ISL_955102, EPI_ISL_960226, EPI_ISL_960303, EPI_ISL_960312, EPI_ISL_962880, EPI_ISL_962882, EPI_ISL_962886, EPI_ISL_965181, EPI_ISL_965264, EPI_ISL_965277, EPI_ISL_965909, EPI_ISL_968214, EPI_ISL_968234, EPI_ISL_968252, EPI_ISL_968857, EPI_ISL_969034, EPI_ISL_977499, EPI_ISL_981384, EPI_ISL_981386, EPI_ISL_981548, EPI_ISL_982860			
see above	Botswana Harvard HIV Reference Laboratory	Botswana Harvard HIV Reference Laboratory	Boitumelo Zuze; Botshelo Radibe; David Lawrence; Dorcas Maruapula; Joseph; Joseph Makhema; Legodile T. Kooepile David Lawrence; Makhema; Mosepele Mosepele; Roger L. Shapiro; Roger Shapiro; Shahin Lockman; Sikhulile Moyo; Simani Gaseitsiwe; Wonderful Choga; Wonderful T. Choga
EPI_ISL_745170, EPI_ISL_745190	Brackengate Field Hospital COVID-19 wc BRG	National Health Laboratory Service (NHLS), Tygerberg	Bronwyn Kleinhans; Eduan Wilkindon; Gert van Zyl; Houriiyah Tegally; Kayla Delaney; Susan Engelbrecht; Tulio de Oliveira; Wolfgang Preiser
EPI_ISL_1040762	Bredasdorp clinic	NHLS/UCT	Arash Iranzadeh; Bruna Galvao; Carolyn Williamson; Deelan Doolabh; Diana Hardie; Innocent Mudau; Kruger Marais; Lynn Tyers; Marvin Hsiao; Stephen Korsman
EPI_ISL_640036	Bridgeton CDC wc BTC	NHLS/UCT	Arash Iranzadeh; Bruna Galvao; Carolyn Williamson; Deelan Doolabh; Diana Hardie; Innocent Mudau; Kruger Marais; Lynn Tyers; Marvin Hsiao; Stephen Korsman
EPI_ISL_467516	CAPRISA	KRISP, KZN Research Innovation and Sequencing Platform	Chimukangara B; Giandhari J; Khan S; Lessells R; Mdlalose K; Pillay S; Tegally H; Wilkinson E; York D; de Oliveira T
EPI_ISL_605780, EPI_ISL_605781, EPI_ISL_605782	CEIRS Data Processing and Coordinating Center, St. Jude Center of Excellence for Influenza Research and Surveillance (CEIRS)	CEIRS Data Processing and Coordinating Center, St. Jude Center of Excellence for Influenza Research and Surveillance (CEIRS)	A.E.; Ali; El-Guindy; El-Sayes, M.; El-Shesheny, R.; El-Taweel, A.; Gomaa, M.; Kamel; Kandeil, A.; Kayali, G.; Kayed; Kutkat, O.; M.A.; M.N.; Mahmoud; Mahrous, N.; Moatasim, Y.; Mostafa, A.; N.M.; Naguib, A.; Roshdy; S.H.; Shehata, M.; Showky, S.; W.H.; Webby, R.
EPI_ISL_1013099, EPI_ISL_1013100, EPI_ISL_1013101, EPI_ISL_1013102, EPI_ISL_1013103, EPI_ISL_1013104, EPI_ISL_1013105, EPI_ISL_1013106, EPI_ISL_1013107, EPI_ISL_1013108, EPI_ISL_1013109, EPI_ISL_1013110, EPI_ISL_1013111, EPI_ISL_1013112, EPI_ISL_1013113, EPI_ISL_1013114, EPI_ISL_1013115, EPI_ISL_1013116, EPI_ISL_1013117, EPI_ISL_1013118, EPI_ISL_1013119, EPI_ISL_1013120, EPI_ISL_1013121, EPI_ISL_1013122, EPI_ISL_1013123, EPI_ISL_1013124, EPI_ISL_1013125, EPI_ISL_1013126, EPI_ISL_1013127, EPI_ISL_1013128, EPI_ISL_1013129, EPI_ISL_1013130, EPI_ISL_1013131, EPI_ISL_1013132, EPI_ISL_1013133, EPI_ISL_1013134, EPI_ISL_1013135, EPI_ISL_1013136, EPI_ISL_1013137, EPI_ISL_1013138, EPI_ISL_1013139, EPI_ISL_1013140, EPI_ISL_1013141, EPI_ISL_1013142, EPI_ISL_1013143, EPI_ISL_1013144, EPI_ISL_1013145, EPI_ISL_1013146, EPI_ISL_1013147, EPI_ISL_1013148, EPI_ISL_1013149, EPI_ISL_1013150, EPI_ISL_1013151, EPI_ISL_1013152, EPI_ISL_1013153, EPI_ISL_1013154, EPI_ISL_1013155, EPI_ISL_1013156, EPI_ISL_1013157, EPI_ISL_1013158, EPI_ISL_1013159, EPI_ISL_1013160, EPI_ISL_1013161, EPI_ISL_1013162, EPI_ISL_1013163, EPI_ISL_1013164, EPI_ISL_1013165, EPI_ISL_1013166, EPI_ISL_1013167, EPI_ISL_1013168, EPI_ISL_1013169, EPI_ISL_1013170, EPI_ISL_1013171, EPI_ISL_1013172, EPI_ISL_1013173, EPI_ISL_1013174, EPI_ISL_1013175, EPI_ISL_1013176, EPI_ISL_1013177, EPI_ISL_1013178, EPI_ISL_1013179, EPI_ISL_1013180, EPI_ISL_1013181, EPI_ISL_1013182, EPI_ISL_1013183, EPI_ISL_1013184, EPI_ISL_1013185, EPI_ISL_1013186, EPI_ISL_1013187, EPI_ISL_1013188, EPI_ISL_1013189, EPI_ISL_1013190, EPI_ISL_1013191, EPI_ISL_1013192, EPI_ISL_1013193, EPI_ISL_1013194, EPI_ISL_1013195, EPI_ISL_1013196, EPI_ISL_1013197, EPI_ISL_1013198, EPI_ISL_1013199, EPI_ISL_1013200, EPI_ISL_1013201, EPI_ISL_1013202, EPI_ISL_1013203, EPI_ISL_1013204, EPI_ISL_1013205, EPI_ISL_1013206, EPI_ISL_1013207, EPI_ISL_1013208, EPI_ISL_1013209, EPI_ISL_1013224, EPI_ISL_1013225, EPI_ISL_1013226, EPI_ISL_1013227, EPI_ISL_1013228, EPI_ISL_1013229, EPI_ISL_1013230, EPI_ISL_1013231, EPI_ISL_1013232, EPI_ISL_1013233, EPI_ISL_1013234, EPI_ISL_1013237, EPI_ISL_1013238, EPI_ISL_1013239, EPI_ISL_1013240, EPI_ISL_1013242, EPI_ISL_1013243, EPI_ISL_1013244, EPI_ISL_1013245, EPI_ISL_1013246, EPI_ISL_1013247,			

EPI_ISL_1013248, EPI_ISL_1013249, EPI_ISL_1013250, EPI_ISL_1013251, EPI_ISL_1013252, EPI_ISL_1013253, EPI_ISL_1013254, EPI_ISL_1013255, EPI_ISL_1013256, EPI_ISL_1013257, EPI_ISL_1013258, EPI_ISL_1013259, EPI_ISL_1013260, EPI_ISL_1013261, EPI_ISL_1013262, EPI_ISL_1013263, EPI_ISL_1013264, EPI_ISL_1013265, EPI_ISL_1013266, EPI_ISL_1013267, EPI_ISL_1013268, EPI_ISL_1013269, EPI_ISL_1013270, EPI_ISL_1013271, EPI_ISL_1013272, EPI_ISL_1013273, EPI_ISL_1013274, EPI_ISL_1013275, EPI_ISL_1013276, EPI_ISL_1013277, EPI_ISL_1013278, EPI_ISL_1013279, EPI_ISL_1013280, EPI_ISL_1013281, EPI_ISL_1013282, EPI_ISL_1013283, EPI_ISL_1013284, EPI_ISL_1013285, EPI_ISL_1013286, EPI_ISL_1013287, EPI_ISL_1013288, EPI_ISL_1013289, EPI_ISL_1013290, EPI_ISL_1013291, EPI_ISL_1013292, EPI_ISL_1013293, EPI_ISL_1013294, EPI_ISL_1013295, EPI_ISL_1013296, EPI_ISL_1013297, EPI_ISL_1013298, EPI_ISL_1013299, EPI_ISL_1013300, EPI_ISL_1013301, EPI_ISL_1013302, EPI_ISL_1013303, EPI_ISL_1013304, EPI_ISL_1013305, EPI_ISL_1013306, EPI_ISL_1013307, EPI_ISL_1013308, EPI_ISL_1013309, EPI_ISL_1013310, EPI_ISL_1013311, EPI_ISL_1013312, EPI_ISL_1013313, EPI_ISL_1013314, EPI_ISL_1013315, EPI_ISL_1013316, EPI_ISL_1013317, EPI_ISL_1013318, EPI_ISL_1013319, EPI_ISL_1013320, EPI_ISL_1013321, EPI_ISL_1013322, EPI_ISL_1013323, EPI_ISL_1013324, EPI_ISL_1013325, EPI_ISL_1013326, EPI_ISL_1013327, EPI_ISL_1013328, EPI_ISL_1013329, EPI_ISL_1013330, EPI_ISL_1013331, EPI_ISL_1013332, EPI_ISL_1013333, EPI_ISL_1013334, EPI_ISL_1013335, EPI_ISL_1013336, EPI_ISL_1013337, EPI_ISL_1013338, EPI_ISL_1013339, EPI_ISL_1013340, EPI_ISL_1013341, EPI_ISL_1013342, EPI_ISL_1013343, EPI_ISL_1013344, EPI_ISL_1013345, EPI_ISL_1013346, EPI_ISL_1013347, EPI_ISL_1013348, EPI_ISL_1013349, EPI_ISL_1013350, EPI_ISL_1013351, EPI_ISL_1013352, EPI_ISL_1013353, EPI_ISL_1013354, EPI_ISL_1013355, EPI_ISL_1013356, EPI_ISL_1013357, EPI_ISL_1013358, EPI_ISL_1013359, EPI_ISL_1013360, EPI_ISL_1013361, EPI_ISL_1013362, EPI_ISL_1013363, EPI_ISL_1013364, EPI_ISL_1013365, EPI_ISL_1013366, EPI_ISL_1013367, EPI_ISL_1013368, EPI_ISL_1013369, EPI_ISL_1013370, EPI_ISL_1013371, EPI_ISL_1013372, EPI_ISL_1013373, EPI_ISL_1013374, EPI_ISL_1013375, EPI_ISL_1013376, EPI_ISL_1013377, EPI_ISL_1013378, EPI_ISL_1013379, EPI_ISL_1013380, EPI_ISL_1013381, EPI_ISL_1013382, EPI_ISL_1013383, EPI_ISL_1013384, EPI_ISL_1013385, EPI_ISL_1013386, EPI_ISL_1013387, EPI_ISL_1013388, EPI_ISL_1013389, EPI_ISL_1013390, EPI_ISL_1013391, EPI_ISL_1013392, EPI_ISL_1013393, EPI_ISL_1013394, EPI_ISL_1013395, EPI_ISL_1013396, EPI_ISL_1013397, EPI_ISL_1013398, EPI_ISL_1013399, EPI_ISL_1013400, EPI_ISL_1013401, EPI_ISL_1013402, EPI_ISL_1013403, EPI_ISL_1013404, EPI_ISL_1013405, EPI_ISL_1013406, EPI_ISL_1013407			
see above	CH de Mayotte	National Reference Center for Viruses of Respiratory Infections, Institut Pasteur, Paris	Angela Brisebarre; Camille Capel; Combe Patrice; Etienne Simon-Lorière; Marion Barbet; Maud Vanpeene; Méline Bizard; Sylvie Behillil; Sylvie van der Werf; Vincent Enouf
EPI_ISL_894220, EPI_ISL_894221, EPI_ISL_894222, EPI_ISL_894224, EPI_ISL_894225, EPI_ISL_894226, EPI_ISL_894227, EPI_ISL_894228, EPI_ISL_894229, EPI_ISL_894230, EPI_ISL_894231, EPI_ISL_909674, EPI_ISL_909687, EPI_ISL_909688, EPI_ISL_909689, EPI_ISL_909690, EPI_ISL_909691, EPI_ISL_909692, EPI_ISL_909693, EPI_ISL_909694, EPI_ISL_909695, EPI_ISL_909696, EPI_ISL_909697, EPI_ISL_909698, EPI_ISL_909699, EPI_ISL_909700, EPI_ISL_909701, EPI_ISL_909708, EPI_ISL_909709, EPI_ISL_909710, EPI_ISL_909711, EPI_ISL_909712, EPI_ISL_909713, EPI_ISL_909714, EPI_ISL_909715, EPI_ISL_909716, EPI_ISL_909717, EPI_ISL_909718, EPI_ISL_909719, EPI_ISL_909720, EPI_ISL_909721, EPI_ISL_909722, EPI_ISL_909723, EPI_ISL_912402, EPI_ISL_912403			
see above	CH de Mayotte - Laboratoire de Biologie	National Reference Center for Viruses of Respiratory Infections, Institut Pasteur, Paris	Angela Brisebarre; Camille Capel; Combe Patrice; Etienne Simon-Lorière; Marion Barbet; Maud Vanpeene; Méline Bizard; Sylvie Behillil; Sylvie van der Werf; Vincent Enouf
EPI_ISL_1190785	CHR LA REUNION FELIX GUYON	CNR Virus des Infections Respiratoires - France SUD	Antonin Bal; Bruno Lina; Bruno Simon; Gregory Destras; Gwendolyne Burfin; Hadrien Regue; Laurence Josset; Martine Valette; Quentin Semanas
EPI_ISL_683835	CICM	Malaria Research and Training Center (MRTC-Parasito)	Abdoulaye Djimde; Antoine Dara
EPI_ISL_487446, EPI_ISL_487447, EPI_ISL_487448, EPI_ISL_487449, EPI_ISL_487450, EPI_ISL_487451, EPI_ISL_487452, EPI_ISL_487453, EPI_ISL_487454, EPI_ISL_487455, EPI_ISL_487456, EPI_ISL_487457, EPI_ISL_487458, EPI_ISL_487459, EPI_ISL_487460, EPI_ISL_487461, EPI_ISL_487462, EPI_ISL_487463, EPI_ISL_487464, EPI_ISL_487465, EPI_ISL_487466			
see above	CICM-Mali	Bundeswehr Institut of Microbiology	Antwerpen; Bestehorn-Willmann; Dürr; Heitzer; Kouriba; Maiga; Quedraogo; Rehn; Sangaré; Sogodogo; Traoré; Walter; Wölfel; Zimmermann
EPI_ISL_1190749, EPI_ISL_1190750, EPI_ISL_1190751, EPI_ISL_1190752, EPI_ISL_1190753, EPI_ISL_1190754, EPI_ISL_1190755, EPI_ISL_1190756, EPI_ISL_1190757, EPI_ISL_1190758, EPI_ISL_1190759, EPI_ISL_1190760, EPI_ISL_1190761, EPI_ISL_1190762, EPI_ISL_1190763, EPI_ISL_1190764, EPI_ISL_1190765, EPI_ISL_1190766, EPI_ISL_1190767, EPI_ISL_1190768			
see above	CREMER(Centre de Rechercherches sur les Maladies Emergentes et Ré-émergentes)	TransVIHMI(Recherches Translationnelles sur le VIH et les Maladies Infectieuses)	Ahidjo Ayouba; Celestin Godwe; Christelle Butel; Dowbiss Meta Djomsi; Eitel Mpoudi Ngole; Eric Delaporte; Esemu Livo; Laetitia Serrano; Marcel Tongo; Marie Amougou; Martin Maidadi Foudi; Martine Peeters; Nicole Vidal; Rodrigue Kamga
EPI_ISL_636980	CS Xai Xai	KRISP, KZN Research Innovation and Sequencing Platform	Giandhari J; Ismael N; Nadia Sitoe; Nedio Mabunda; Paulo Arnaldo; Pillay S; Tegally H; Wilkinson E; de Oliveira T
EPI_ISL_745121	Calvinia Hospital	National Health Laboratory Service (NHLS), Tygerberg	Bronwyn Kleinhans; Eduan Wilkindon; Gert van Zyl; Houriiyah Tegally; Kayla Delaney; Susan Engelbrecht; Tulio de Oliveira; Wolfgang Preiser
EPI_ISL_576371, EPI_ISL_576372, EPI_ISL_576373, EPI_ISL_857315, EPI_ISL_857316, EPI_ISL_857317, EPI_ISL_857318, EPI_ISL_857319, EPI_ISL_857320, EPI_ISL_857321, EPI_ISL_857322, EPI_ISL_857323, EPI_ISL_857324, EPI_ISL_857325, EPI_ISL_857326, EPI_ISL_857327, EPI_ISL_857328, EPI_ISL_857329, EPI_ISL_857330, EPI_ISL_857331, EPI_ISL_857332, EPI_ISL_857333, EPI_ISL_857334, EPI_ISL_857335, EPI_ISL_857336, EPI_ISL_857337, EPI_ISL_857338, EPI_ISL_857339, EPI_ISL_857340, EPI_ISL_857341, EPI_ISL_857342, EPI_ISL_857343, EPI_ISL_857344, EPI_ISL_857345, EPI_ISL_857346, EPI_ISL_857347, EPI_ISL_862783, EPI_ISL_862784, EPI_ISL_862785, EPI_ISL_862786, EPI_ISL_862787, EPI_ISL_862788, EPI_ISL_862789, EPI_ISL_862790, EPI_ISL_862791, EPI_ISL_862792, EPI_ISL_862793, EPI_ISL_862794, EPI_ISL_862795, EPI_ISL_862796, EPI_ISL_862797, EPI_ISL_862798, EPI_ISL_862799, EPI_ISL_862800, EPI_ISL_862801, EPI_ISL_862802, EPI_ISL_862803, EPI_ISL_862804, EPI_ISL_862805, EPI_ISL_862806, EPI_ISL_862807, EPI_ISL_862808, EPI_ISL_862809, EPI_ISL_862810, EPI_ISL_862811, EPI_ISL_862812, EPI_ISL_862813, EPI_ISL_890195, EPI_ISL_890196, EPI_ISL_890197, EPI_ISL_890198, EPI_ISL_890199, EPI_ISL_890200, EPI_ISL_890201, EPI_ISL_890202, EPI_ISL_890203, EPI_ISL_890204, EPI_ISL_890205, EPI_ISL_890206, EPI_ISL_890207, EPI_ISL_890208, EPI_ISL_890209, EPI_ISL_890210, EPI_ISL_890211, EPI_ISL_890212, EPI_ISL_890213, EPI_ISL_890214, EPI_ISL_890215, EPI_ISL_890216, EPI_ISL_890217, EPI_ISL_890218, EPI_ISL_890219, EPI_ISL_890220, EPI_ISL_890221, EPI_ISL_890222, EPI_ISL_890223, EPI_ISL_890224, EPI_ISL_890225, EPI_ISL_890226, EPI_ISL_890227, EPI_ISL_890228, EPI_ISL_890229, EPI_ISL_890230, EPI_ISL_890231, EPI_ISL_907086, EPI_ISL_907087, EPI_ISL_907088, EPI_ISL_907089, EPI_ISL_907090, EPI_ISL_907091, EPI_ISL_907092, EPI_ISL_907093, EPI_ISL_907094, EPI_ISL_907095, EPI_ISL_907096, EPI_ISL_907097, EPI_ISL_907098, EPI_ISL_907099, EPI_ISL_907100, EPI_ISL_907101, EPI_ISL_907102, EPI_ISL_907103, EPI_ISL_907104, EPI_ISL_907105, EPI_ISL_907106, EPI_ISL_907107, EPI_ISL_907108, EPI_ISL_907109, EPI_ISL_907110, EPI_ISL_907111, EPI_ISL_907112, EPI_ISL_907113, EPI_ISL_907114, EPI_ISL_907115, EPI_ISL_907116, EPI_ISL_907117, EPI_ISL_907118			
see above	Cancer Biology Department, National Cancer Institute	Cancer Biology Department, National Cancer Institute	A.A.; A.E.; A.N.; Abouelhoda, M.; Ahmed; Bahnassy; Elhosieny; F.W.; Gad; H.K.; Hafez; Hamdy; M.G.; M.M.; M.S.; O.S.; Sedawy; Soliman; Soliman, L.; Zekri
EPI_ISL_1040916	Cancer Biology Department, National Cancer Institute	Cancer Biology Department, National Cancer Institute	A.A.; A.E.; A.N.; Ahmed; Bahnassy; Elhosieny; F.W.; Gad; H.K.; Hafez; Hamdy; L. and Abouelhoda, M.; M.G.; M.M.; M.S.; O.S.; Sedawy; Soliman; Zekri
EPI_ISL_971451	Cell culture Unit at CV-MIT belonging to HIMMV	Functional Genomic Platform UATRS-biology, CNRST	Abderrazzak Rfak; Amine Idriss Lahlou; Elmostafa EL FAHIME; Hicham Elannaz; Khalid ENNIBI; Marouane MELLOUL; Mly Abdelaziz ELALAOUI; Mostafa ELOUENNASS; Mouhssine Hemlali; Nadia Touil; Sanaa ALAOUI-Amine
EPI_ISL_430820	Center of Scientific Excellence for Influenza Viruses, National Research Centre (NRC), Egypt.	Center of Scientific Excellence for Influenza Viruses, National Research Centre (NRC), Egypt.	Abo Shama; Ahmed E Kayed; Ahmed El-Taweel; Ahmed Kandeil; Ahmed Mostafa; Amal Naguib; M Noura; Mahmoud Shehata; Mina Kamel; Mohamed Ahmed Ali; Mohamed El Sayes; Mokhtar Gomaa; Nancy M. El Guindy; Omnia Kutkat; Rabeh El-Shesheny; Sara Mahmoud; Shymaa Showky Ahmed; Wael Roshdy; Yassmin Moatasim
EPI_ISL_430819	Center of Scientific Excellence for Influenza Viruses,National Research Centre (NRC), Egypt.	Center of Scientific Excellence for Influenza Viruses,National Research Centre (NRC), Egypt.	Abo Shama; Ahmed E Kayed; Ahmed El-Taweel; Ahmed Kandeil; Ahmed Mostafa; Amal Naguib; M Noura; Mahmoud Shehata; Mina Kamel; Mohamed Ahmed Ali; Mohamed El Sayes; Mokhtar Gomaa; Nancy M. El Guindy; Omnia Kutkat; Rabeh El-Shesheny; Sara Mahmoud; Shymaa Showky Ahmed; Wael Roshdy; Yassmin Moatasim
EPI_ISL_815255	Centogene	Centogene	Krishna Kumar Kandaswamy; Peter Bauer; Vivi Hue-Trang Lieu
EPI_ISL_794593, EPI_ISL_794594, EPI_ISL_794595, EPI_ISL_794596, EPI_ISL_794597, EPI_ISL_794598, EPI_ISL_794599, EPI_ISL_794600, EPI_ISL_794601, EPI_ISL_794602, EPI_ISL_794603, EPI_ISL_794604			
see above	Central Laboratories, Egyptian Ministry of Health and Population	Central Laboratories, Egyptian Ministry of Health and Population	A.E.; Ali; El Guindy; El Sayes; El Taweel, A.; El-Shesheny, R.; Gomaa, M.; Kamel; Kandeil, A.; Kayali, G.; Kayed; Khalifa; Kutkat, O.; M.A.; M.K.; M.N.; Mahmoud; Mahrous, N.; Moatasim, Y.; Mostafa, A.; N.M.; Naguib, A.; Roshdy; S.H.; Saleh, M.; Shawky, S.; Shehata, M.; W.H.
EPI_ISL_529032	Central Molecular Microbiology Laboratory and Next Generation Sequencing Reference Laboratory, Clinical and Chemical Pathology Department, Faculty of Medicine, CAIRO UNIVERSITY	Next Generation Sequencing Reference Laboratory, Faculty of Medicine, CAIRO UNIVERSITY and The Center for Genome and Microbiome Research, Faculty of Pharmacy, CAIRO UNIVERSITY	May Abdelfattah; May Sherif Soliman; Ramy Karam Aziz
EPI_ISL_529031	Central Molecular Microbiology Laboratory, Clinical and Chemical Pathology Department, Faculty of Medicine, CAIRO UNIVERSITY	Next Generation Sequencing Reference Laboratory, Faculty of Medicine, Cairo University and The Center for Genome and Microbiome Research, Faculty of Pharmacy, CAIRO UNIVERSITY	May Abdelfattah; May Sherif Soliman; Ramy Karam Aziz
EPI_ISL_794605, EPI_ISL_794606, EPI_ISL_794607, EPI_ISL_794608	Central Public Health Laboratories, Egyptian Ministry of Health and Population	Central Public Health Laboratories, Egyptian Ministry of Health and Population	A.E.; Ali; El Guindy; El Sayes; El Taweel, A.; El-Shesheny, R.; Gomaa, M.; Kamel; Kandeil, A.; Kayali, G.; Kayed; Khalifa; Kutkat, O.; M.A.; M.K.; M.N.; Mahmoud; Mahrous, N.; Moatasim, Y.; Mostafa, A.; N.M.; Naguib, A.; Roshdy; S.H.; Saleh, M.; Shawky, S.; Shehata, M.; W.H.
EPI_ISL_457999, EPI_ISL_458000	Centre For Biotechnology Research and Development	Centre For Biotechnology Research and Development	C.N. and Michuki; D.K.; G.N.; J.O.; Kimotho, J.; Matoke-Muhia; Matoke-Muhia, D.; Muuo; Ochwoto, M.; S.L.; S.N.; Symeker; Waruhiu; Zablon
EPI_ISL_660442	Centre Muraz	Project group Epidemology of Highly Pathogenic Microorganisms, Robert Koch Institut	Abdoul-Salam Ouedraogo; Arsène Zongo; Essia Belarbi; Fabian Leendertz; Grit Schubert; Soumeya Ouangraoua; Yacouba Sawadogo
EPI_ISL_1001042, EPI_ISL_1001043, EPI_ISL_1001044, EPI_ISL_1001045, EPI_ISL_1001046, EPI_ISL_1001047, EPI_ISL_1001048, EPI_ISL_1001049, EPI_ISL_1001050, EPI_ISL_1001051, EPI_ISL_1001052, EPI_ISL_1001053, EPI_ISL_1001054, EPI_ISL_1001055, EPI_ISL_1001056, EPI_ISL_1001057, EPI_ISL_1001058, EPI_ISL_1001059, EPI_ISL_1001060, EPI_ISL_1001061, EPI_ISL_1001062, EPI_ISL_1001063, EPI_ISL_1001064, EPI_ISL_1001065, EPI_ISL_1001066, EPI_ISL_1001067, EPI_ISL_1001068			
see above	Centre Pasteur du Cameroun	Institut Pasteur de Dakar	Njouom Richard
EPI_ISL_512873	Centre Pasteur of Cameroun	Virology Service, Centre Pasteur of Cameroun	Richard Njouom and Serge Alain SADEUH-Mba
EPI_ISL_788933, EPI_ISL_788934, EPI_ISL_788935, EPI_ISL_788936, EPI_ISL_788937, EPI_ISL_788938	Centre de Recherche et de Formation en Infectiologie Guinée	TransVIHMI, IRD/INSERM/Monpellier University	Abdoul Karim SOUMAH; Abdoulaye TOURE; Ahidjo AYOUBA; Alimou CAMARA; Alpha Kabinet KEITA; Bouna; Bouna Yatassaye; Christelle BUTEL; Eric DELAPORTE; Jean-louis MONEMOU; Joel KOIVOGUI; Kaba KOUROUMA; Laetitia SERRANO; Mamadou Bhoye KEITA; Mamadou Saliou BAH; Mamadou Saliou SOW; Mandiou DIAKITE; Martine PEETERS; Moriba POVOGUI; Penda Malhado DIALLO; Sakoba KEITA
EPI_ISL_539573, EPI_ISL_539574, EPI_ISL_539575, EPI_ISL_539576	Centre de Recherches Medicales de Lambarene (CERMEL)	Department of Emerging Infectious Diseases, Institute of Tropical Medicine, Nagasaki University	Akim A. Adegnika; Bertrand Lell; Haruka Abe; Jiro Yasuda; Rodrigue Bikangui; Yuri Ushijima
EPI_ISL_978534, EPI_ISL_978535, EPI_ISL_978536, EPI_ISL_978537, EPI_ISL_978538, EPI_ISL_978539, EPI_ISL_978540, EPI_ISL_978541, EPI_ISL_978542, EPI_ISL_978543, EPI_ISL_978544, EPI_ISL_978545, EPI_ISL_978546, EPI_ISL_978547, EPI_ISL_978548, EPI_ISL_978549, EPI_ISL_978550, EPI_ISL_978551, EPI_ISL_978552			
see above	Centre de Virologie des Maladies infectueuses Tropicales	Functional Genomic Platform UATRS-biology, CNRST	; Abdelilah LARAQUI; Abderrazzak Rfaki; Elmostafa BENAISSA; Elmostafa EL FAHIME; Elmostafa EL FAHIME.; Farida HILALI; Hemlali Mouhssine; Khalid ENNIBI; Khalid ENNIBI.; Marouane MELLOUL; Mly Abdelaziz ELALAOUI; Mostafa ELOUENNASS; Nadia Touil; Safaa GHOULAM; Safae Ghoulam; Sanaa ALAOUI-Amine; Taha Chouati; Tahar BAJJOU; Yassine SEKHSOK; Yassine SEKHSOKH
EPI_ISL_471456, EPI_ISL_471457, EPI_ISL_471458, EPI_ISL_471459, EPI_ISL_471460	Centre de Virologie des Maladies Tropicales	Functional Genomic Platform/Service Analyses Biologique/UATRS/ Centre National Pour la Recherche Scientifique Et Technique (CNRST)	Abdelillah LARAQUI; Abdelkader LAATIRIS; Ahmed REGGAD; Elmostafa EL FAHIME; Farida HILALI; Hicham EL ANNAZ; Idriss-Amine LAHLOU; Khalid ENNIBI; Marouane MELLOUL; Mly Abdelaziz ELALAOUI; Mostafa ELOUENNASS; Nadia TOUIL; Rachid ABI; Rida TAGAJDID; Safae ELKOCHRI; Sanaa ALAOUI-Amine; Tahar BAJJOU; Yassine SEKHSOKH; Youssef AKHOUAD; Zohour KASMY
EPI_ISL_654794	Centre for Human Virology & Genomics, Nigerian Institute of Medical Research	Centre for Human Virology & Genomics, Nigerian Institute of Medical Research	Shaibu, J.
EPI_ISL_940075, EPI_ISL_940076, EPI_ISL_940077, EPI_ISL_940078, EPI_ISL_940079, EPI_ISL_940080, EPI_ISL_940081, EPI_ISL_940082, EPI_ISL_940083, EPI_ISL_940084, EPI_ISL_940085, EPI_ISL_940086, EPI_ISL_940087, EPI_ISL_940088, EPI_ISL_940089, EPI_ISL_940090, EPI_ISL_940091, EPI_ISL_940092, EPI_ISL_940093, EPI_ISL_940094, EPI_ISL_940095, EPI_ISL_940096, EPI_ISL_940097, EPI_ISL_940098, EPI_ISL_940099, EPI_ISL_940100, EPI_ISL_940101, EPI_ISL_940102, EPI_ISL_940103, EPI_ISL_940104, EPI_ISL_940106, EPI_ISL_940107, EPI_ISL_940109, EPI_ISL_940110, EPI_ISL_940111, EPI_ISL_940112, EPI_ISL_940113, EPI_ISL_940114, EPI_ISL_940115, EPI_ISL_940116, EPI_ISL_940117, EPI_ISL_940118, EPI_ISL_940119, EPI_ISL_940120, EPI_ISL_940121, EPI_ISL_940122, EPI_ISL_940123, EPI_ISL_940124, EPI_ISL_940126, EPI_ISL_940127, EPI_ISL_940128, EPI_ISL_940129, EPI_ISL_940130, EPI_ISL_940131, EPI_ISL_940132, EPI_ISL_940133, EPI_ISL_940134, EPI_ISL_940135, EPI_ISL_940136, EPI_ISL_940137, EPI_ISL_1239267, EPI_ISL_1239268, EPI_ISL_1239269, EPI_ISL_1239270, EPI_ISL_1239271, EPI_ISL_1239272, EPI_ISL_1239273, EPI_ISL_1239274, EPI_ISL_1239275, EPI_ISL_1239276, EPI_ISL_1239277, EPI_ISL_1239278, EPI_ISL_1239279, EPI_ISL_1239280, EPI_ISL_1239281, EPI_ISL_1239282, EPI_ISL_1239283, EPI_ISL_1239284, EPI_ISL_1239285, EPI_ISL_1239287, EPI_ISL_1239288, EPI_ISL_1239289, EPI_ISL_1239290, EPI_ISL_1239291, EPI_ISL_1239292, EPI_ISL_1239293, EPI_ISL_1239294, EPI_ISL_1239295, EPI_ISL_1239296, EPI_ISL_1239297, EPI_ISL_1239298, EPI_ISL_1239299, EPI_ISL_1239300, EPI_ISL_1239301, EPI_ISL_1239302, EPI_ISL_1239303, EPI_ISL_1239304, EPI_ISL_1239305, EPI_ISL_1239306, EPI_ISL_1239307, EPI_ISL_1239308, EPI_ISL_1239309, EPI_ISL_1239310, EPI_ISL_1239311, EPI_ISL_1239312, EPI_ISL_1239313, EPI_ISL_1239314, EPI_ISL_1239315, EPI_ISL_1239316, EPI_ISL_1239317, EPI_ISL_1239318, EPI_ISL_1239319, EPI_ISL_1239320, EPI_ISL_1239321, EPI_ISL_1239322, EPI_ISL_1239323, EPI_ISL_1239324, EPI_ISL_1239325, EPI_ISL_1239326, EPI_ISL_1239328, EPI_ISL_1239329			
see above	Charlotte Maxeke Johannesburg Academic Hospital, National Health Laboratory Services, Gauteng, South Africa	National Institute for Communicable Diseases of the National Health Laboratory Service	Allam M; Amoako DG; Bhiman JN; Ismail A; Mahlangu B; Mohale T; Ntuli N; Scheepers C
EPI_ISL_1239330, EPI_ISL_1239331, EPI_ISL_1239332, EPI_ISL_1239333, EPI_ISL_1239334, EPI_ISL_1239335, EPI_ISL_1239336, EPI_ISL_1239337, EPI_ISL_1239338, EPI_ISL_1239339, EPI_ISL_1239340, EPI_ISL_1239341, EPI_ISL_1239342, EPI_ISL_1239343, EPI_ISL_1239344, EPI_ISL_1239345, EPI_ISL_1239346, EPI_ISL_1239347, EPI_ISL_1239348, EPI_ISL_1239349, EPI_ISL_1239350, EPI_ISL_1239351, EPI_ISL_1239352, EPI_ISL_1239353, EPI_ISL_1239354, EPI_ISL_1239355, EPI_ISL_1239356, EPI_ISL_1239357, EPI_ISL_1239358, EPI_ISL_1239360			
see above	Chris Hani Baragwanath Hospital, National Health Laboratory Services, Gauteng, South Africa	National Institute for Communicable Diseases of the National Health Laboratory Service	Amoako DG; Bhiman JN; Ismail A; Mahlangu B; Mohale T; Ntuli N; Scheepers C
EPI_ISL_636981	City of Chimoio	KRISP, KZN Research Innovation and Sequencing Platform	Giandhari J; Ismael N; Nadia Sitoe; Nedio Mabunda; Paulo Arnaldo; Pillay S; Tegally H; Wilkinson E; de Oliveira T
EPI_ISL_640068, EPI_ISL_700432,	Clinic-in-Asla	NHLS/UCT	Arash Iranzadeh; Bruna Galvao; Carolyn Williamson; Deelan Doolabh; Diana Hardie; Houriiyah Tegally; Innocent Mudau; Kruger Marais; Lynn Tyers; Marvin Hsiao; Stephen Korsman

EPI_ISL_700519			
EPI_ISL_745128, EPI_ISL_745153	CoVid EC Nelson Mandela Bay Metro	National Health Laboratory Service (NHLS), Tygerberg	Bronwyn Kleinhans; Eduan Wilkindon; Gert van Zyl; Houriiyah Tegally; Kayla Delaney; Susan Engelbrecht; Tulio de Oliveira; Wolfgang Preiser
EPI_ISL_640062, EPI_ISL_640063, EPI_ISL_1040819, EPI_ISL_1040820, EPI_ISL_1040821	CoVid WC Cape Town Metro	NHLS/UCT	Arash Iranzadeh; Bruna Galvao; Carolyn Williamson; Deelan Doolabh; Diana Hardie; Innocent Mudau; Kruger Marais; Lynn Tyers; Marvin Hsiao; Stephen Korsman
EPI_ISL_640015, EPI_ISL_640118, EPI_ISL_700414, EPI_ISL_700441, EPI_ISL_700467, EPI_ISL_700488, EPI_ISL_700492, EPI_ISL_700496, EPI_ISL_700504, EPI_ISL_700506, EPI_ISL_700533, EPI_ISL_700536, EPI_ISL_700538, EPI_ISL_700539, EPI_ISL_700554, EPI_ISL_700556, EPI_ISL_700557, EPI_ISL_700563, EPI_ISL_700581, EPI_ISL_700587, EPI_ISL_700590, EPI_ISL_700599			
see above	Conville CDC wc CVC	NHLS/UCT	Arash Iranzadeh; Bruna Galvao; Carolyn Williamson; Deelan Doolabh; Diana Hardie; Houriiyah Tegally; Innocent Mudau; Kruger Marais; Lynn Tyers; Marvin Hsiao; Stephen Korsman
EPI_ISL_960119, EPI_ISL_960122, EPI_ISL_960127, EPI_ISL_960134	Conville CDC wc CVC	National Health Laboratory Service/UCT	Arash Iranzadeh; Bruna Galvao; Carolyn Williamson; Deelan Doolabh; Diana Hardie; Innocent Mudau; Kruger Marais; Lynn Tyers; Marvin Hsiao; Stephen Korsman
EPI_ISL_696472, EPI_ISL_696492, EPI_ISL_696508, EPI_ISL_696509, EPI_ISL_696512	Conville CDC wc CVC & NHLS/UCT	KRISP, KZN Research Innovation and Sequencing Platform	Arash Iranzadeh; Bruna Galvao; Carolyn Williamson; Deelan Doolabh; Diana Hardie; Emanuel James San; Houriiyah Tegally; Innocent Mudau; Jennifer Giandhari; Kruger Marais; Lynn Tyers; Marvin Hsiao; Stephen Korsman; Sureshnee Pillay; Tulio de Oliveira
EPI_ISL_700443	Crags Clinic wc CRG	NHLS/UCT	Arash Iranzadeh; Bruna Galvao; Carolyn Williamson; Deelan Doolabh; Diana Hardie; Houriiyah Tegally; Innocent Mudau; Kruger Marais; Lynn Tyers; Marvin Hsiao; Stephen Korsman
EPI_ISL_696480	Crags Clinic wc CRG & NHLS/UCT	KRISP, KZN Research Innovation and Sequencing Platform	Arash Iranzadeh; Bruna Galvao; Carolyn Williamson; Deelan Doolabh; Diana Hardie; Emanuel James San; Houriiyah Tegally; Innocent Mudau; Jennifer Giandhari; Kruger Marais; Lynn Tyers; Marvin Hsiao; Stephen Korsman; Sureshnee Pillay; Tulio de Oliveira
EPI_ISL_640029, EPI_ISL_640048, EPI_ISL_700420, EPI_ISL_700427, EPI_ISL_700448, EPI_ISL_700470, EPI_ISL_700564, EPI_ISL_700569			
see above	D'Almeida Clinic wc DAL	NHLS/UCT	Arash Iranzadeh; Bruna Galvao; Carolyn Williamson; Deelan Doolabh; Diana Hardie; Houriiyah Tegally; Innocent Mudau; Kruger Marais; Lynn Tyers; Marvin Hsiao; Stephen Korsman
EPI_ISL_745171, EPI_ISL_745172	Delft CHC wc DFP	National Health Laboratory Service (NHLS), Tygerberg	Bronwyn Kleinhans; Eduan Wilkindon; Gert van Zyl; Houriiyah Tegally; Kayla Delaney; Susan Engelbrecht; Tulio de Oliveira; Wolfgang Preiser
EPI_ISL_515082, EPI_ISL_515083, EPI_ISL_515084, EPI_ISL_515085, EPI_ISL_515086, EPI_ISL_515087, EPI_ISL_515088, EPI_ISL_515089, EPI_ISL_515090, EPI_ISL_515091, EPI_ISL_515092, EPI_ISL_515093, EPI_ISL_515094, EPI_ISL_515095, EPI_ISL_515096, EPI_ISL_515097, EPI_ISL_515098, EPI_ISL_515099, EPI_ISL_515100, EPI_ISL_515101, EPI_ISL_515102, EPI_ISL_515103, EPI_ISL_515104, EPI_ISL_515105, EPI_ISL_515106, EPI_ISL_515107, EPI_ISL_515108, EPI_ISL_515109, EPI_ISL_515110, EPI_ISL_515111, EPI_ISL_515112			
see above	Department of Biochemistry, Cell and Molecular Biology	WACCBIP, University of Ghana	A.K.; Adu, B.; Amenga-Etego; Ampofo, W.; Amuzu; Anang; Arjarquah, A.; Asante, I.; Awandare; Bediako, Y.; Boatemaa, L.; Bonney, E.; Bonney, K.; C.M.; D.S.; Eshun, M.; G.A.; G.B.; J.K.; J.M.; Kotey, E.; Kumordjie, S.; Kyei; L.N.; Magnussen, V.; Morang'a; Mutungi; Ngoi; Quashie, P.; Tei-Maya, F.
EPI_ISL_884826, EPI_ISL_884827, EPI_ISL_884828, EPI_ISL_884829, EPI_ISL_884830, EPI_ISL_884831, EPI_ISL_884832, EPI_ISL_884833, EPI_ISL_884834, EPI_ISL_884835, EPI_ISL_884836, EPI_ISL_884837, EPI_ISL_884838, EPI_ISL_884839, EPI_ISL_884840, EPI_ISL_884841, EPI_ISL_884842, EPI_ISL_884843, EPI_ISL_884844, EPI_ISL_884845, EPI_ISL_884846, EPI_ISL_884847, EPI_ISL_884848, EPI_ISL_884849, EPI_ISL_884850, EPI_ISL_884851, EPI_ISL_884852, EPI_ISL_884853, EPI_ISL_884854, EPI_ISL_884855, EPI_ISL_884856, EPI_ISL_944646, EPI_ISL_944647, EPI_ISL_944648, EPI_ISL_944649, EPI_ISL_944650, EPI_ISL_944651, EPI_ISL_944652, EPI_ISL_944653, EPI_ISL_944654, EPI_ISL_944655, EPI_ISL_944656, EPI_ISL_944657, EPI_ISL_944658, EPI_ISL_944659, EPI_ISL_944660, EPI_ISL_944661, EPI_ISL_944662, EPI_ISL_944663, EPI_ISL_944664, EPI_ISL_944665, EPI_ISL_944666, EPI_ISL_944667, EPI_ISL_944668, EPI_ISL_944669, EPI_ISL_944670, EPI_ISL_944671, EPI_ISL_944672, EPI_ISL_944673, EPI_ISL_944674, EPI_ISL_944675, EPI_ISL_944676, EPI_ISL_944677, EPI_ISL_944678, EPI_ISL_944679, EPI_ISL_944680, EPI_ISL_944681, EPI_ISL_944682, EPI_ISL_944683, EPI_ISL_944684, EPI_ISL_944685, EPI_ISL_944686, EPI_ISL_944687, EPI_ISL_944688, EPI_ISL_944689, EPI_ISL_944690, EPI_ISL_944691, EPI_ISL_944692, EPI_ISL_944693, EPI_ISL_944694, EPI_ISL_944695, EPI_ISL_944696, EPI_ISL_944697, EPI_ISL_944698, EPI_ISL_944699, EPI_ISL_944700, EPI_ISL_944701, EPI_ISL_944702, EPI_ISL_944703, EPI_ISL_944704, EPI_ISL_944705, EPI_ISL_944706, EPI_ISL_944707, EPI_ISL_944708, EPI_ISL_944709, EPI_ISL_944710, EPI_ISL_944711, EPI_ISL_944712, EPI_ISL_944713, EPI_ISL_944714, EPI_ISL_944715, EPI_ISL_944716, EPI_ISL_944717, EPI_ISL_944718, EPI_ISL_944719, EPI_ISL_944720, EPI_ISL_944721, EPI_ISL_944722, EPI_ISL_944723, EPI_ISL_944724, EPI_ISL_944725, EPI_ISL_944726, EPI_ISL_944727, EPI_ISL_944728, EPI_ISL_944729, EPI_ISL_944730, EPI_ISL_944731, EPI_ISL_944732, EPI_ISL_944733, EPI_ISL_944734, EPI_ISL_944735			
see above	Department of Biochemistry, Cell and Molecular Biology, West African Centre for Cell Biology of Infectious Pathogens (WACCBIP), University of Ghana	Department of Biochemistry, Cell and Molecular Biology, West African Centre for Cell Biology of Infectious Pathogens (WACCBIP), University of Ghana	A.-K.; A.B.; Abass; Adusei-Poku, M.; Akoriyea; Amenga-Etego; Amoako, E.; Ampofo; Amuzu; Asante, I.; Awandare; Bediako, Y.; Boakye; Bonney; Bonney, E.; C.M.; D.S.; Diallo; E.B.; G.A.; J.H.; J.K.; J.M.; Kibinge, N.; Kumi-Ansah, F.; L.N.; Magnussen, V.; Mohammed, A.; Morang'a; N.T.; Ndam; Ngoi; O.D.; Odoom; Odoom, T.; Ofori-Boadu, L.; Quansah; Quashie, P.; S.K.; Saiid, S.; Tapela, K.; Tei-Maya, F.; W.K.
EPI_ISL_1273103	Department of Microbial Biotechnology, Genetic Engineering Division, National Research Centre	Department of Microbial Biotechnology, Genetic Engineering Division, National Research Centre	A.E. and Shemis, M.; A.G.; Dawood; Farghaly, A.; Fikry; Khalifa; M.K.; R.M.; Taha
EPI_ISL_855557, EPI_ISL_855558, EPI_ISL_855559, EPI_ISL_855560, EPI_ISL_855561, EPI_ISL_855562, EPI_ISL_855563, EPI_ISL_855564, EPI_ISL_855565, EPI_ISL_855566, EPI_ISL_855567, EPI_ISL_855568, EPI_ISL_855569, EPI_ISL_855570, EPI_ISL_855571, EPI_ISL_855572			
see above	Department of Virology, Principal Military Hospital of Instruction of Tunis	Bundeswehr Institute of Microbiology	Habiba Naija; Kilian Stoecker; Malena Bestehorn-Willmann; Markus H. Antwerpen; Mathias C. Walter; Roman Wölfel & Mohamed Ben Moussa; Simone Eckstein; Susann Handrick
EPI_ISL_640044	District 6 CDC wc DSI	NHLS/UCT	Arash Iranzadeh; Bruna Galvao; Carolyn Williamson; Deelan Doolabh; Diana Hardie; Innocent Mudau; Kruger Marais; Lynn Tyers; Marvin Hsiao; Stephen Korsman
EPI_ISL_640100, EPI_ISL_700513, EPI_ISL_700517, EPI_ISL_700525, EPI_ISL_700547, EPI_ISL_700552, EPI_ISL_700567, EPI_ISL_700568, EPI_ISL_700570, EPI_ISL_700582, EPI_ISL_700591, EPI_ISL_700592			
see above	Dr Abdurahman CDC wc DAC	NHLS/UCT	Arash Iranzadeh; Bruna Galvao; Carolyn Williamson; Deelan Doolabh; Diana Hardie; Innocent Mudau; Kruger Marais; Lynn Tyers; Marvin Hsiao; Stephen Korsman
EPI_ISL_960160, EPI_ISL_960161, EPI_ISL_960171, EPI_ISL_960172	Dr Abdurahman CDC wc DAC	National Health Laboratory Service/UCT	Arash Iranzadeh; Bruna Galvao; Carolyn Williamson; Deelan Doolabh; Diana Hardie; Innocent Mudau; Kruger Marais; Lynn Tyers; Marvin Hsiao; Stephen Korsman
EPI_ISL_745113, EPI_ISL_745132, EPI_ISL_745154	Dr Ivan Toms Clinic wc IVT	National Health Laboratory Service (NHLS), Tygerberg	Bronwyn Kleinhans; Eduan Wilkindon; Gert van Zyl; Houriiyah Tegally; Kayla Delaney; Susan Engelbrecht; Tulio de Oliveira; Wolfgang Preiser
EPI_ISL_745112, EPI_ISL_745138, EPI_ISL_745139	Dr Van Niekerk Hospital	National Health Laboratory Service (NHLS), Tygerberg	Bronwyn Kleinhans; Eduan Wilkindon; Gert van Zyl; Houriiyah Tegally; Kayla Delaney; Susan Engelbrecht; Tulio de Oliveira; Wolfgang Preiser
EPI_ISL_640057, EPI_ISL_640059, EPI_ISL_640091, EPI_ISL_640094, EPI_ISL_640095	Du Noon CDC wc DNC	NHLS/UCT	Arash Iranzadeh; Bruna Galvao; Carolyn Williamson; Deelan Doolabh; Diana Hardie; Innocent Mudau; Kruger Marais; Lynn Tyers; Marvin Hsiao; Stephen Korsman
EPI_ISL_640024, EPI_ISL_700520	Dysselsdorp Clinic wc DDC	NHLS/UCT	Arash Iranzadeh; Bruna Galvao; Carolyn Williamson; Deelan Doolabh; Diana Hardie; Houriiyah Tegally; Innocent Mudau; Kruger Marais; Lynn Tyers; Marvin Hsiao; Stephen Korsman
EPI_ISL_960096	Dysselsdorp Clinic wc DDC	National Health Laboratory Service/UCT	Arash Iranzadeh; Bruna Galvao; Carolyn Williamson; Deelan Doolabh; Diana Hardie; Innocent Mudau; Kruger Marais; Lynn Tyers; Marvin Hsiao; Stephen Korsman
EPI_ISL_1137609	Département de Maladies Infectieuses, CHU Farhat Hached Sousse, Tunisie	Laboratoire des Procédés de Criblage Moléculaire et Cellulaire-Centre de Biotechnologie de Sfax	A. and Masmoudi, S.; Abdelmoulah, F.; Abid, M.; Abid, N.; Ajili, F.; Aouni, M.; Ben Ayed, I.; Bensaid, M.; Chawech, H.; Chtourou, A.; Elargoubi, A.; Fki-berrajah, L.; Gaaloul, I.; Gargouri, S.; Hammami, A.; Kamoun, S.; Karray Hakim, H.; Kharat, N.; Letaief, A.; Mastouri, M.; Mhalla, S.; Nabli, A.; Rebai; Smeti, I.; Souissi, A.; Stambouli, N.; Turki, M.
EPI_ISL_712077, EPI_ISL_712078, EPI_ISL_712092	East London NHLS Laboratory, Eastern Cape, South Africa	National Institute for Communicable Diseases of the National Health Laboratory Service	Allam M; Bhiman JN; Ismail A; Mahlangu B; Mohale T; Ntuli N
EPI_ISL_745137, EPI_ISL_745151	Edameni Clinic	National Health Laboratory Service (NHLS), Tygerberg	Bronwyn Kleinhans; Eduan Wilkindon; Gert van Zyl; Houriiyah Tegally; Kayla Delaney; Susan Engelbrecht; Tulio de Oliveira; Wolfgang Preiser
EPI_ISL_745119, EPI_ISL_745173, EPI_ISL_745174, EPI_ISL_745175, EPI_ISL_745176, EPI_ISL_745177, EPI_ISL_745178			
see above	Eerste River Hospital wc ERH	National Health Laboratory Service (NHLS), Tygerberg	Bronwyn Kleinhans; Eduan Wilkindon; Gert van Zyl; Houriiyah Tegally; Kayla Delaney; Susan Engelbrecht; Tulio de Oliveira; Wolfgang Preiser
EPI_ISL_468044, EPI_ISL_468045, EPI_ISL_468046, EPI_ISL_468047, EPI_ISL_468048, EPI_ISL_468049, EPI_ISL_468050, EPI_ISL_468051, EPI_ISL_468052, EPI_ISL_468053, EPI_ISL_468054, EPI_ISL_468055, EPI_ISL_468056, EPI_ISL_468057, EPI_ISL_468058, EPI_ISL_468059, EPI_ISL_468060, EPI_ISL_468061, EPI_ISL_468062, EPI_ISL_475722, EPI_ISL_475723, EPI_ISL_475724, EPI_ISL_477161, EPI_ISL_478672, EPI_ISL_479686, EPI_ISL_479687, EPI_ISL_479688, EPI_ISL_479689, EPI_ISL_479690, EPI_ISL_479691, EPI_ISL_479692, EPI_ISL_479693, EPI_ISL_479694, EPI_ISL_479695, EPI_ISL_479696, EPI_ISL_479697, EPI_ISL_479698, EPI_ISL_479699, EPI_ISL_479700, EPI_ISL_479701, EPI_ISL_479702, EPI_ISL_479703, EPI_ISL_479704, EPI_ISL_479705, EPI_ISL_479706, EPI_ISL_479707, EPI_ISL_479708, EPI_ISL_479709, EPI_ISL_479710, EPI_ISL_479711, EPI_ISL_479712, EPI_ISL_479713, EPI_ISL_479714, EPI_ISL_479715, EPI_ISL_479716, EPI_ISL_479717, EPI_ISL_479718, EPI_ISL_479719, EPI_ISL_479720, EPI_ISL_479721, EPI_ISL_479722, EPI_ISL_479723, EPI_ISL_479724, EPI_ISL_479725, EPI_ISL_479726, EPI_ISL_479727, EPI_ISL_479728, EPI_ISL_479729, EPI_ISL_479730, EPI_ISL_479731, EPI_ISL_479732, EPI_ISL_479733, EPI_ISL_479734, EPI_ISL_479735, EPI_ISL_524426, EPI_ISL_524427, EPI_ISL_529141, EPI_ISL_529142, EPI_ISL_529143, EPI_ISL_529144, EPI_ISL_529145			
see above	Egyptian National Cancer Institute (ENCI)	Egyptian National Cancer Institute (ENCI)	A.A.; A.N.; Abdel Rahman N; Abdel Rahman N.; Abdelhamid, W.; Abouelhoda; Abouelhoda, M.; Ahmed; Ali; Ali, M.; Amer; Bahnassy; Elkhateeb; Elsissy; Ezzelarab; Gad, A.; H.K.; Hafez; Hamdy; Hassan; Hassan, W.; K.E.; Khattab; M.A.; M.H.; M.M.; M.S.; Mahmoud; Mohamed; O.S.; Raouf, A.; S.M.; Samir, M.; Soliman; W.A.; Zekri
EPI_ISL_469275	Egyptian National Cancer Institute (ENCI)	Human Genome Center	A.A.; Abdel Rahman N; Abdelhamid, W.; Abouelhoda; Ahmed; Ali, M.; Amer; Bahnassy; Elkhateeb; Elsissy; Ezzelarab; Gad, A.; H.K.; Hafez; Hamdy; Hassan, W.; K.E.; M.H.; M.M.; M.S.; Mohamed; O.S.; Raouf, A.; S.M.; Samir, M.; Soliman; Zekri
EPI_ISL_745110, EPI_ISL_745125, EPI_ISL_745162	Elsies River CHC wc ERP	National Health Laboratory Service (NHLS), Tygerberg	Bronwyn Kleinhans; Eduan Wilkindon; Gert van Zyl; Houriiyah Tegally; Kayla Delaney; Susan Engelbrecht; Tulio de Oliveira; Wolfgang Preiser
EPI_ISL_605924, EPI_ISL_605925, EPI_ISL_605926, EPI_ISL_605927, EPI_ISL_605928	Emergence des Maladies Virales, Centre Interdisciplinaire de Recherches Medicales de Franceville	Emergence des Maladies Virales, Centre Interdisciplinaire de Recherches Medicales de Franceville	A.M.; Andeko; J.-B.; J.C.; Kandet Yattara, M.; Lekana-Douki; Mbongo kama, E.; Mintsa Ndong, A.; N'dilimabaka, N.; Ngonga Dikongo; S.E.; Zong Minko, O.
EPI_ISL_640064	Ethembeni Clinic [Prieska]	NHLS/UCT	Arash Iranzadeh; Bruna Galvao; Carolyn Williamson; Deelan Doolabh; Diana Hardie; Innocent Mudau; Kruger Marais; Lynn Tyers; Marvin Hsiao; Stephen Korsman
EPI_ISL_640033, EPI_ISL_640050, EPI_ISL_640088, EPI_ISL_640090, EPI_ISL_640116, EPI_ISL_640121, EPI_ISL_640123, EPI_ISL_640124, EPI_ISL_640125, EPI_ISL_1040681, EPI_ISL_1040682, EPI_ISL_1040739, EPI_ISL_1040740, EPI_ISL_1040775, EPI_ISL_1040777, EPI_ISL_1040779, EPI_ISL_1040780			

see above	False Bay Hospital wc FBH	NHLS/UCT	Arash Iranzadeh; Bruna Galvao; Carolyn Williamson; Deelan Doolabh; Diana Hardie; Innocent Mudau; Kruger Marais; Lynn Tyers; Marvin Hsiao; Stephen Korsman
EPI_ISL_960105, EPI_ISL_960110, EPI_ISL_960112	False Bay Hospital wc FBH	National Health Laboratory Service/UCT	Arash Iranzadeh; Bruna Galvao; Carolyn Williamson; Deelan Doolabh; Diana Hardie; Innocent Mudau; Kruger Marais; Lynn Tyers; Marvin Hsiao; Stephen Korsman
EPI_ISL_581455, EPI_ISL_581462, EPI_ISL_581472, EPI_ISL_581486, EPI_ISL_581487, EPI_ISL_581488, EPI_ISL_581489, EPI_ISL_581490, EPI_ISL_581491, EPI_ISL_581492, EPI_ISL_581493			
see above	Fondation Congolaise pour la recherche medicale (FCRM)	NGS Competence Center Tübingen, Institut für Medizinische Mikrobiologie und Hygiene, Universitätsklinikum Tübingen	Angel Angelov
EPI_ISL_912353, EPI_ISL_912354, EPI_ISL_912355, EPI_ISL_912356, EPI_ISL_912357, EPI_ISL_912358, EPI_ISL_912359, EPI_ISL_912360, EPI_ISL_912361, EPI_ISL_912362, EPI_ISL_912363, EPI_ISL_912364, EPI_ISL_912365, EPI_ISL_912366, EPI_ISL_912367, EPI_ISL_912368, EPI_ISL_912369, EPI_ISL_912370, EPI_ISL_912371, EPI_ISL_912372, EPI_ISL_912373, EPI_ISL_912374, EPI_ISL_912375, EPI_ISL_912376, EPI_ISL_912377, EPI_ISL_912378, EPI_ISL_912379, EPI_ISL_912380, EPI_ISL_912381, EPI_ISL_912382, EPI_ISL_912383, EPI_ISL_912384, EPI_ISL_912385, EPI_ISL_912386, EPI_ISL_912387, EPI_ISL_912388, EPI_ISL_912389, EPI_ISL_912390, EPI_ISL_912391, EPI_ISL_912392, EPI_ISL_912393, EPI_ISL_912394			
see above	Fondation Congolaise pour la recherche medicale (FCRM), Francine Ntoumi	NGS Competence Center Tuebingen, Institut für Medizinische Mikrobiologie und Hygiene, Universitaetsklinikum Tübingen	Angel Angelov
EPI_ISL_522549, EPI_ISL_522550	Félix Guyon Hospital	UMR PIMIT Université de La Réunion	Camille Lebarbenchon; David Wilkinson; Patrick Mavingui
EPI_ISL_812783, EPI_ISL_812784, EPI_ISL_812785, EPI_ISL_812786, EPI_ISL_812787, EPI_ISL_812788, EPI_ISL_812789, EPI_ISL_812790, EPI_ISL_812791, EPI_ISL_812792, EPI_ISL_812793, EPI_ISL_812794, EPI_ISL_812795, EPI_ISL_812796, EPI_ISL_812797, EPI_ISL_812798, EPI_ISL_812799, EPI_ISL_812800, EPI_ISL_812801, EPI_ISL_812802, EPI_ISL_812803, EPI_ISL_812804, EPI_ISL_812805, EPI_ISL_812806, EPI_ISL_812807, EPI_ISL_812808, EPI_ISL_812809, EPI_ISL_812810, EPI_ISL_812811, EPI_ISL_812812, EPI_ISL_812813, EPI_ISL_812814, EPI_ISL_812815, EPI_ISL_812816, EPI_ISL_812817, EPI_ISL_812818, EPI_ISL_812819, EPI_ISL_812820, EPI_ISL_812821, EPI_ISL_812822, EPI_ISL_812823, EPI_ISL_812824, EPI_ISL_812825, EPI_ISL_812826, EPI_ISL_812827, EPI_ISL_812828, EPI_ISL_812829, EPI_ISL_812830, EPI_ISL_812831, EPI_ISL_812832, EPI_ISL_812833, EPI_ISL_812834, EPI_ISL_812835, EPI_ISL_812836, EPI_ISL_812837, EPI_ISL_812838, EPI_ISL_812839, EPI_ISL_812840, EPI_ISL_812841, EPI_ISL_812842, EPI_ISL_812843, EPI_ISL_812844, EPI_ISL_812845, EPI_ISL_812846, EPI_ISL_812847, EPI_ISL_812848, EPI_ISL_812849, EPI_ISL_812850, EPI_ISL_812851, EPI_ISL_812852, EPI_ISL_812853, EPI_ISL_812854, EPI_ISL_812855, EPI_ISL_812856, EPI_ISL_812857, EPI_ISL_812858, EPI_ISL_812859, EPI_ISL_812860, EPI_ISL_812861, EPI_ISL_812862, EPI_ISL_812863, EPI_ISL_812864, EPI_ISL_812865, EPI_ISL_812866, EPI_ISL_812867, EPI_ISL_812868, EPI_ISL_812869, EPI_ISL_812870, EPI_ISL_812871, EPI_ISL_812872			
see above	Genomics Program, Children Cancer Hospital	Genomics Program, Children Cancer Hospital	Abdo, I.; Abouelnaga, S.; Amer, K.; Bakry, U.; Diab, A.; El-Shaqnqery, H.; El-Zayat, M.; Farawyla, H.; Gomaa, C.; Hadad, A.; Halafawy, A.; Hammad, M.; Hassan, R.; Hassan, W.; Hatem, A.; Hussein, S.; Jalal, D.; Magdeldin, S.; Mansour, T.; Monuir, G.; Salah, H.; Samir, O.; Sayed, A.; Soliman, M.; Soliman, S.; shalaby, L.
EPI_ISL_640016, EPI_ISL_640027, EPI_ISL_700497	George Hospital wc GRH	NHLS/UCT	Arash Iranzadeh; Bruna Galvao; Carolyn Williamson; Deelan Doolabh; Diana Hardie; Innocent Mudau; Kruger Marais; Lynn Tyers; Marvin Hsiao; Stephen Korsman
EPI_ISL_960118	George Hospital wc GRH	National Health Laboratory Service/UCT	Arash Iranzadeh; Bruna Galvao; Carolyn Williamson; Deelan Doolabh; Diana Hardie; Innocent Mudau; Kruger Marais; Lynn Tyers; Marvin Hsiao; Stephen Korsman
EPI_ISL_696458, EPI_ISL_696471, EPI_ISL_696473, EPI_ISL_696481, EPI_ISL_696503	George Hospital wc GRH & NHLS/UCT	KRISP, KZN Research Innovation and Sequencing Platform	Arash Iranzadeh; Bruna Galvao; Carolyn Williamson; Deelan Doolabh; Diana Hardie; Emanuel James San; Houriiyah Tegally; Innocent Mudau; Jennifer Giandhari; Kruger Marais; Lynn Tyers; Marvin Hsiao; Stephen Korsman; Sureshnee Pillay; Tulio de Oliveira
EPI_ISL_640049	George Road Sat Clinic wc GWM	NHLS/UCT	Arash Iranzadeh; Bruna Galvao; Carolyn Williamson; Deelan Doolabh; Diana Hardie; Innocent Mudau; Kruger Marais; Lynn Tyers; Marvin Hsiao; Stephen Korsman
EPI_ISL_700543, EPI_ISL_700550	Great Brak River Clinic wc GBC	NHLS/UCT	Arash Iranzadeh; Bruna Galvao; Carolyn Williamson; Deelan Doolabh; Diana Hardie; Houriiyah Tegally; Innocent Mudau; Kruger Marais; Lynn Tyers; Marvin Hsiao; Stephen Korsman
EPI_ISL_960149	Great Brak River Clinic wc GBC	National Health Laboratory Service/UCT	Arash Iranzadeh; Bruna Galvao; Carolyn Williamson; Deelan Doolabh; Diana Hardie; Innocent Mudau; Kruger Marais; Lynn Tyers; Marvin Hsiao; Stephen Korsman
EPI_ISL_696466, EPI_ISL_696497, EPI_ISL_696501, EPI_ISL_696507	Great Brak River Clinic wc GBC & NHLS/UCT	KRISP, KZN Research Innovation and Sequencing Platform	Arash Iranzadeh; Bruna Galvao; Carolyn Williamson; Deelan Doolabh; Diana Hardie; Emanuel James San; Houriiyah Tegally; Innocent Mudau; Jennifer Giandhari; Kruger Marais; Lynn Tyers; Marvin Hsiao; Stephen Korsman; Sureshnee Pillay; Tulio de Oliveira
EPI_ISL_640041	Green Point CDC wc GPH	NHLS/UCT	Arash Iranzadeh; Bruna Galvao; Carolyn Williamson; Deelan Doolabh; Diana Hardie; Innocent Mudau; Kruger Marais; Lynn Tyers; Marvin Hsiao; Stephen Korsman
EPI_ISL_640040, EPI_ISL_640043, EPI_ISL_640045, EPI_ISL_640051, EPI_ISL_640052, EPI_ISL_640053, EPI_ISL_640054, EPI_ISL_640055, EPI_ISL_640058, EPI_ISL_640060, EPI_ISL_640061, EPI_ISL_640066, EPI_ISL_640067, EPI_ISL_640084, EPI_ISL_640111, EPI_ISL_640112, EPI_ISL_640119, EPI_ISL_640120, EPI_ISL_640122, EPI_ISL_640128, EPI_ISL_640129, EPI_ISL_640130, EPI_ISL_640131, EPI_ISL_640132, EPI_ISL_640133, EPI_ISL_640134, EPI_ISL_640135, EPI_ISL_640137, EPI_ISL_640138, EPI_ISL_640139, EPI_ISL_640140, EPI_ISL_700493, EPI_ISL_700507, EPI_ISL_700534, EPI_ISL_700577, EPI_ISL_1040644, EPI_ISL_1040645, EPI_ISL_1040646, EPI_ISL_1040648, EPI_ISL_1040649, EPI_ISL_1040650, EPI_ISL_1040651, EPI_ISL_1040652, EPI_ISL_1040653, EPI_ISL_1040654, EPI_ISL_1040656, EPI_ISL_1040657, EPI_ISL_1040658, EPI_ISL_1040659, EPI_ISL_1040660, EPI_ISL_1040661, EPI_ISL_1040663, EPI_ISL_1040668, EPI_ISL_1040672, EPI_ISL_1040673, EPI_ISL_1040676, EPI_ISL_1040683, EPI_ISL_1040684, EPI_ISL_1040685, EPI_ISL_1040686, EPI_ISL_1040687, EPI_ISL_1040689, EPI_ISL_1040690, EPI_ISL_1040691, EPI_ISL_1040692, EPI_ISL_1040693, EPI_ISL_1040696, EPI_ISL_1040697, EPI_ISL_1040698, EPI_ISL_1040699, EPI_ISL_1040700, EPI_ISL_1040704, EPI_ISL_1040705, EPI_ISL_1040706, EPI_ISL_1040707, EPI_ISL_1040708, EPI_ISL_1040711, EPI_ISL_1040714, EPI_ISL_1040716, EPI_ISL_1040720, EPI_ISL_1040722, EPI_ISL_1040723, EPI_ISL_1040725, EPI_ISL_1040726, EPI_ISL_1040727, EPI_ISL_1040728, EPI_ISL_1040729, EPI_ISL_1040731, EPI_ISL_1040733, EPI_ISL_1040735, EPI_ISL_1040738, EPI_ISL_1040745, EPI_ISL_1040747, EPI_ISL_1040748, EPI_ISL_1040750, EPI_ISL_1040751, EPI_ISL_1040752, EPI_ISL_1040753, EPI_ISL_1040754, EPI_ISL_1040755, EPI_ISL_1040756, EPI_ISL_1040758, EPI_ISL_1040759, EPI_ISL_1040763, EPI_ISL_1040765, EPI_ISL_1040767, EPI_ISL_1040768, EPI_ISL_1040781, EPI_ISL_1040782, EPI_ISL_1040783, EPI_ISL_1040785, EPI_ISL_1040786, EPI_ISL_1040787, EPI_ISL_1040789, EPI_ISL_1040795, EPI_ISL_1040796, EPI_ISL_1040800, EPI_ISL_1040801, EPI_ISL_1040802, EPI_ISL_1040804, EPI_ISL_1040807, EPI_ISL_1040808, EPI_ISL_1040811, EPI_ISL_1040812, EPI_ISL_1040813, EPI_ISL_1040815, EPI_ISL_1040816, EPI_ISL_1040817, EPI_ISL_1040818, EPI_ISL_1040822			
see above	Groote Schuur Hospital wc GSH	NHLS/UCT	Arash Iranzadeh; Bruna Galvao; Carolyn Williamson; Deelan Doolabh; Diana Hardie; Innocent Mudau; Kruger Marais; Lynn Tyers; Marvin Hsiao; Stephen Korsman
EPI_ISL_960102, EPI_ISL_960103, EPI_ISL_960107, EPI_ISL_960109, EPI_ISL_960117, EPI_ISL_960126, EPI_ISL_960132, EPI_ISL_960133, EPI_ISL_960135, EPI_ISL_960136, EPI_ISL_960140, EPI_ISL_960142, EPI_ISL_960143, EPI_ISL_960145			
see above	Groote Schuur Hospital wc GSH	National Health Laboratory Service/UCT	Arash Iranzadeh; Bruna Galvao; Carolyn Williamson; Deelan Doolabh; Diana Hardie; Innocent Mudau; Kruger Marais; Lynn Tyers; Marvin Hsiao; Stephen Korsman
EPI_ISL_696464	Groote Schuur Hospital wc GSH & NHLS/UCT	KRISP, KZN Research Innovation and Sequencing Platform	Arash Iranzadeh; Bruna Galvao; Carolyn Williamson; Deelan Doolabh; Diana Hardie; Emanuel James San; Houriiyah Tegally; Innocent Mudau; Jennifer Giandhari; Kruger Marais; Lynn Tyers; Marvin Hsiao; Stephen Korsman; Sureshnee Pillay; Tulio de Oliveira
EPI_ISL_1040761	Guguletu CHC	NHLS/UCT	Arash Iranzadeh; Bruna Galvao; Carolyn Williamson; Deelan Doolabh; Diana Hardie; Innocent Mudau; Kruger Marais; Lynn Tyers; Marvin Hsiao; Stephen Korsman
EPI_ISL_640069, EPI_ISL_640070, EPI_ISL_640071, EPI_ISL_640104, EPI_ISL_700415, EPI_ISL_700455, EPI_ISL_700481, EPI_ISL_700491, EPI_ISL_700503, EPI_ISL_700512, EPI_ISL_700514, EPI_ISL_700530, EPI_ISL_700576, EPI_ISL_700578, EPI_ISL_1040666, EPI_ISL_1040667, EPI_ISL_1040669, EPI_ISL_1040671, EPI_ISL_1040675, EPI_ISL_1040678, EPI_ISL_1040710, EPI_ISL_1040713, EPI_ISL_1040715, EPI_ISL_1040718, EPI_ISL_1040721, EPI_ISL_1040724, EPI_ISL_1040732, EPI_ISL_1040734, EPI_ISL_1040793, EPI_ISL_1040805			
see above	Guguletu CHC wc GDH	NHLS/UCT	Arash Iranzadeh; Bruna Galvao; Carolyn Williamson; Deelan Doolabh; Diana Hardie; Innocent Mudau; Kruger Marais; Lynn Tyers; Marvin Hsiao; Stephen Korsman
EPI_ISL_960154, EPI_ISL_960155, EPI_ISL_960156, EPI_ISL_960157, EPI_ISL_960158, EPI_ISL_960163, EPI_ISL_960167, EPI_ISL_960174			
see above	Guguletu CHC wc GDH	National Health Laboratory Service/UCT	Arash Iranzadeh; Bruna Galvao; Carolyn Williamson; Deelan Doolabh; Diana Hardie; Innocent Mudau; Kruger Marais; Lynn Tyers; Marvin Hsiao; Stephen Korsman
EPI_ISL_1170956	HIV Molecular Lab, Ethiopian Public Health Institute	HIV Molecular Lab	A.G.; Abichu, G.; Ahyong, V.; D. and Tato, C.; Damena, D.; Detweiler, A.; Fahsbender, E.; Gutema, G.; Kalantar, K.; Kidane, E.; Leta; Neff, N.; S.K.; Tan, M.; Tessema; Vanaerschot, M.; Weldemariam
EPI_ISL_1169919	HIV Molecular Laboratory, Ethiopian Public Health Institute	HIV Molecular Laboratory, Ethiopian Public Health Institute	A.G.; Abichu, G.; Ahyong, V.; D. and Tato, C.; Damena, D.; Detweiler, A.; Fahsbender, E.; Gutema, G.; Kalantar, K.; Kidane, E.; Leta; Neff, N.; S.K.; Tan, M.; Tessema; Vanaerschot, M.; Weldemariam
EPI_ISL_1170958	HIV Molecular Laboratory, Ethiopian Public Health Institute, Ethiopia	HIV molecular lab, Ethiopian Public Health Institute, Ethiopian	A.G.; Abichu, G.; Ahyong, V.; D. and Tato, C.; Damena, D.; Detweiler, A.; Fahsbender, E.; Gutema, G.; Kalantar, K.; Kidane, E.; Leta; Neff, N.; S.K.; Tan, M.; Tessema; Vanaerschot, M.; Weldemariam
EPI_ISL_1170957	HIV Molecular laboratory, Ethiopian Public Health Institute	HIV Molecular Laboratory	A.G.; Abichu, G.; Ahyong, V.; D. and Tato, C.; Damena, D.; Detweiler, A.; Fahsbender, E.; Gutema, G.; Kalantar, K.; Kidane, E.; Leta; Neff, N.; S.K.; Tan, M.; Tessema; Vanaerschot, M.; Weldemariam
EPI_ISL_636975, EPI_ISL_636976, EPI_ISL_636977, EPI_ISL_636978, EPI_ISL_636979	HP Pemba	KRISP, KZN Research Innovation and Sequencing Platform	Giandhari J; Ismael N; Nadia Sitoe; Nedio Mabunda; Paulo Arnaldo; Pillay S; Tegally H; Wilkinson E; de Oliveira T
EPI_ISL_660137, EPI_ISL_660138, EPI_ISL_660139, EPI_ISL_660141, EPI_ISL_660142	Hamadi	National Health Laboratory Service (NHLS), Tygerberg	Bronwyn Kleinhans; Davis M-A; Draper C; Eduan Wilkindon; Gert van Zyl; Houriiyah Tegally; Hsiao M; Kayla Delaney; Siegfried N; Susan Engelbrecht; Tulio de Oliveira; Williamson C; Wolfgang Preiser
EPI_ISL_700411, EPI_ISL_700418, EPI_ISL_700457, EPI_ISL_700463, EPI_ISL_700477, EPI_ISL_700480, EPI_ISL_700494, EPI_ISL_700515, EPI_ISL_700516, EPI_ISL_700561, EPI_ISL_700562, EPI_ISL_700566, EPI_ISL_700573, EPI_ISL_700585, EPI_ISL_700593, EPI_ISL_1040694, EPI_ISL_1040695, EPI_ISL_1040717, EPI_ISL_1040730, EPI_ISL_1040764, EPI_ISL_1040797, EPI_ISL_1040809			
see above	Hanover Park CHC wc HPH	NHLS/UCT	Arash Iranzadeh; Bruna Galvao; Carolyn Williamson; Deelan Doolabh; Diana Hardie; Innocent Mudau; Kruger Marais; Lynn Tyers; Marvin Hsiao; Stephen Korsman
EPI_ISL_960165	Hanover Park CHC wc HPH	National Health Laboratory Service/UCT	Arash Iranzadeh; Bruna Galvao; Carolyn Williamson; Deelan Doolabh; Diana Hardie; Innocent Mudau; Kruger Marais; Lynn Tyers; Marvin Hsiao; Stephen Korsman
EPI_ISL_640018	Heidelberg Clinic wc HBC	NHLS/UCT	Arash Iranzadeh; Bruna Galvao; Carolyn Williamson; Deelan Doolabh; Diana Hardie; Innocent Mudau; Kruger Marais; Lynn Tyers; Marvin Hsiao; Stephen Korsman
EPI_ISL_640101, EPI_ISL_640103, EPI_ISL_640106, EPI_ISL_700412, EPI_ISL_700454, EPI_ISL_700459, EPI_ISL_700473, EPI_ISL_700499, EPI_ISL_700508, EPI_ISL_700522, EPI_ISL_700549, EPI_ISL_1040719			
see above	Heideveld CDC wc HVP	NHLS/UCT	Arash Iranzadeh; Bruna Galvao; Carolyn Williamson; Deelan Doolabh; Diana Hardie; Innocent Mudau; Kruger Marais; Lynn Tyers; Marvin Hsiao; Stephen Korsman
EPI_ISL_960164, EPI_ISL_960168, EPI_ISL_960169, EPI_ISL_960170, EPI_ISL_960173	Heideveld CDC wc HVP	National Health Laboratory Service/UCT	Arash Iranzadeh; Bruna Galvao; Carolyn Williamson; Deelan Doolabh; Diana Hardie; Innocent Mudau; Kruger Marais; Lynn Tyers; Marvin Hsiao; Stephen Korsman
EPI_ISL_640082, EPI_ISL_700445, EPI_ISL_700545, EPI_ISL_700559, EPI_ISL_700574, EPI_ISL_700584, EPI_ISL_700595, EPI_ISL_700598, EPI_ISL_1040662, EPI_ISL_1040665, EPI_ISL_1040670, EPI_ISL_1040680, EPI_ISL_1040688, EPI_ISL_1040757, EPI_ISL_1040798, EPI_ISL_1040803, EPI_ISL_1040806, EPI_ISL_1040810			
see above	Heideveld Emergency Centre	NHLS/UCT	Arash Iranzadeh; Bruna Galvao; Carolyn Williamson; Deelan Doolabh; Diana Hardie; Houriiyah Tegally; Innocent Mudau; Kruger Marais; Lynn Tyers; Marvin Hsiao; Stephen Korsman
EPI_ISL_960124, EPI_ISL_960153, EPI_ISL_960162, EPI_ISL_960166	Heideveld Emergency Centre	National Health Laboratory Service/UCT	Arash Iranzadeh; Bruna Galvao; Carolyn Williamson; Deelan Doolabh; Diana Hardie; Innocent Mudau; Kruger Marais; Lynn Tyers; Marvin Hsiao; Stephen Korsman
EPI_ISL_700565, EPI_ISL_700571	Herbertsdale Sat Clinic wc HBD	NHLS/UCT	Arash Iranzadeh; Bruna Galvao; Carolyn Williamson; Deelan Doolabh; Diana Hardie; Innocent Mudau; Kruger Marais; Lynn Tyers; Marvin Hsiao; Stephen Korsman
EPI_ISL_1196013	Hlathikhulu Government Hospital	National Institute for Communicable Diseases of the National Health Laboratory Service	Amoako DG; Bhiman JN; Ismail A; Mahlangu B; Maphalala GP; Mohale T; Ntuli N; Scheepers C

EPI_ISL_1196003, EPI_ISL_1196011, EPI_ISL_1196012, EPI_ISL_1196014	Homecare	National Institute for Communicable Diseases of the National Health Laboratory Service	Amoako DG; Bhiman JN; Ismail A; Mahlangu B; Maphalala GP; Mohale T; Ntuli N; Scheepers C
EPI_ISL_977664, EPI_ISL_1166891, EPI_ISL_1166892, EPI_ISL_1166893, EPI_ISL_1166894, EPI_ISL_1166895, EPI_ISL_1166896, EPI_ISL_1166897, EPI_ISL_1166898, EPI_ISL_1166899, EPI_ISL_1166900, EPI_ISL_1166901, EPI_ISL_1166902, EPI_ISL_1166903, EPI_ISL_1166904, EPI_ISL_1166905, EPI_ISL_1166906, EPI_ISL_1166907, EPI_ISL_1166908, EPI_ISL_1166909, EPI_ISL_1166910, EPI_ISL_1166911, EPI_ISL_1166912, EPI_ISL_1166913, EPI_ISL_1166914, EPI_ISL_1166915, EPI_ISL_1166916, EPI_ISL_1166917, EPI_ISL_1166919, EPI_ISL_1166920, EPI_ISL_1166921, EPI_ISL_1166922, EPI_ISL_1166923, EPI_ISL_1166924, EPI_ISL_1166925, EPI_ISL_1166926, EPI_ISL_1166927, EPI_ISL_1166928, EPI_ISL_1166929, EPI_ISL_1166930, EPI_ISL_1166931, EPI_ISL_1166932, EPI_ISL_1166933, EPI_ISL_1166934, EPI_ISL_1166935, EPI_ISL_1166936, EPI_ISL_1166937, EPI_ISL_1166938, EPI_ISL_1166939, EPI_ISL_1166940, EPI_ISL_1166941, EPI_ISL_1166942, EPI_ISL_1166943, EPI_ISL_1166944, EPI_ISL_1166945, EPI_ISL_1166946, EPI_ISL_1166947, EPI_ISL_1166948, EPI_ISL_1166949, EPI_ISL_1166950, EPI_ISL_1166951, EPI_ISL_1166952, EPI_ISL_1166953, EPI_ISL_1166954, EPI_ISL_1166955, EPI_ISL_1166956, EPI_ISL_1166957, EPI_ISL_1166958, EPI_ISL_1166959, EPI_ISL_1166960, EPI_ISL_1166961, EPI_ISL_1166962, EPI_ISL_1166963, EPI_ISL_1166964, EPI_ISL_1166965, EPI_ISL_1166966, EPI_ISL_1166967, EPI_ISL_1166968, EPI_ISL_1166969, EPI_ISL_1166970, EPI_ISL_1166971, EPI_ISL_1166972, EPI_ISL_1166973, EPI_ISL_1166974, EPI_ISL_1166975, EPI_ISL_1166976, EPI_ISL_1166977, EPI_ISL_1166978, EPI_ISL_1166979, EPI_ISL_1166980, EPI_ISL_1166981, EPI_ISL_1166982, EPI_ISL_1166983, EPI_ISL_1166984, EPI_ISL_1166985, EPI_ISL_1166986, EPI_ISL_1166987, EPI_ISL_1166988, EPI_ISL_1166989, EPI_ISL_1166990, EPI_ISL_1166991, EPI_ISL_1166992, EPI_ISL_1166993, EPI_ISL_1166994, EPI_ISL_1166995, EPI_ISL_1166996, EPI_ISL_1166997, EPI_ISL_1166998, EPI_ISL_1166999, EPI_ISL_1167000, EPI_ISL_1167001, EPI_ISL_1167002, EPI_ISL_1167003, EPI_ISL_1167004, EPI_ISL_1167005, EPI_ISL_1167006, EPI_ISL_1167007, EPI_ISL_1167008, EPI_ISL_1167009, EPI_ISL_1167010, EPI_ISL_1167011, EPI_ISL_1167012, EPI_ISL_1167013, EPI_ISL_1167014, EPI_ISL_1167015, EPI_ISL_1167016, EPI_ISL_1167017, EPI_ISL_1167018, EPI_ISL_1167019, EPI_ISL_1167020, EPI_ISL_1167021			
see above	Hopital	National Reference Center for Viruses of Respiratory Infections, Institut Pasteur, Paris	Angela Brisebarre; Camille Capel; Combe Patrice; Etienne Simon-Lorière; Marion Barbet; Maud Vanpeene; Méline Bizard; Sylvie Behillil; Sylvie van der Werf; Vincent Enouf
EPI_ISL_700458, EPI_ISL_700524, EPI_ISL_700558	Hornlee Clinic wc HLC	NHLS/UCT	Arash Iranzadeh; Bruna Galvao; Carolyn Williamson; Deelan Doolabh; Diana Hardie; Houriiyah Tegally; Innocent Mudau; Kruger Marais; Lynn Tyers; Marvin Hsiao; Stephen Korsman
EPI_ISL_696457, EPI_ISL_696479, EPI_ISL_696483, EPI_ISL_696486, EPI_ISL_696502	Hornlee Clinic wc HLC & NHLS/UCT	KRISP, KZN Research Innovation and Sequencing Platform	Arash Iranzadeh; Bruna Galvao; Carolyn Williamson; Deelan Doolabh; Diana Hardie; Emanuel James San; Houriiyah Tegally; Innocent Mudau; Jennifer Giandhari; Kruger Marais; Lynn Tyers; Marvin Hsiao; Stephen Korsman; Sureshnee Pillay; Tulio de Oliveira
EPI_ISL_855360, EPI_ISL_855361, EPI_ISL_855362, EPI_ISL_855363, EPI_ISL_855364, EPI_ISL_855365, EPI_ISL_855366, EPI_ISL_855367, EPI_ISL_855368, EPI_ISL_855369, EPI_ISL_855370, EPI_ISL_855371, EPI_ISL_855372, EPI_ISL_855373, EPI_ISL_855374, EPI_ISL_855375, EPI_ISL_855376, EPI_ISL_855377, EPI_ISL_855378, EPI_ISL_855379, EPI_ISL_855380, EPI_ISL_890355, EPI_ISL_890356, EPI_ISL_890357, EPI_ISL_890358			
see above	Hospital	National Reference Center for Viruses of Respiratory Infections, Institut Pasteur, Paris	Angela Brisebarre; Camille Capel; Combe Patrice; Etienne Simon-Lorière; Marion Barbet; Maud Vanpeene; Méline Bizard; Sylvie Behillil; Sylvie van der Werf; Vincent Enouf
EPI_ISL_710532, EPI_ISL_710534, EPI_ISL_710537, EPI_ISL_710540, EPI_ISL_710541, EPI_ISL_710575, EPI_ISL_711057, EPI_ISL_712060			
see above	Hôpital Fattouma-Bourguiba de Monastir	Laboratoire des Procédés de Criblage Moléculaire et Cellulaire-Centre de Biotechnologie de Sfax	A. and Masmoudi, S.; Abdelmoulah, F.; Abid, N.; Ajili, F.; Aouni, M.; Ben Ayed, I.; Bensaid, M.; Chtourou, A.; Elargoubi, A.; Fki-berrajah, L.; Gaaloul, I.; Gargouri, S.; Hammami, A.; Kamoun, S.; Karray Hakim, H.; Kharat, N.; Mastouri, M.; Mhalla, S.; Nabli, A.; Rebai; Smeti, I.; Souissi, A.; Stambouli, N.; Turki, M.
EPI_ISL_1027628	IRESSEF GENOMICS LAB	IRESSEF GENOMICS LAB	Abdoulie Kanteh; Dr Abdou PADANE; Dr Abdul Karim Sesay; Dr Aminata MBOUP; Dr Badara CISSE; Dr Birahim Pierre NDIAYE; Dr Moustapha MBOW; Dr Papa Alassane DIAW; Professor Ndéye Coumba Touré KANE; Professor Souleymane MBOUP
EPI_ISL_1018071, EPI_ISL_1018072, EPI_ISL_1018073, EPI_ISL_1018074, EPI_ISL_1018075, EPI_ISL_1018076, EPI_ISL_1018077, EPI_ISL_1018078, EPI_ISL_1018079, EPI_ISL_1018080, EPI_ISL_1018081, EPI_ISL_1018082, EPI_ISL_1018083, EPI_ISL_1018084, EPI_ISL_1018085, EPI_ISL_1018086, EPI_ISL_1018087, EPI_ISL_1018088, EPI_ISL_1018089, EPI_ISL_1018090, EPI_ISL_1018091, EPI_ISL_1018092, EPI_ISL_1018093, EPI_ISL_1018094, EPI_ISL_1018095, EPI_ISL_1018096, EPI_ISL_1018097, EPI_ISL_1018098, EPI_ISL_1018099, EPI_ISL_1018100, EPI_ISL_1018101			
see above	Immunology, Noguchi Memorial Institute for Medical Research	Immunology, Noguchi Memorial Institute for Medical Research	Adu, B.; Adusei-Poku; Agbodzi, B.; Ampofo; Appiah-Kubi, J.; Asare; Bonney; Egyir, B.; J.K.; K.M.; Kumordjie, S.; M.A.; Mohktar, Q.; Odoom; Oteng, F.; Owusu-Nyantakyi, C.; W.K.; Yeboah, C.
EPI_ISL_1240719, EPI_ISL_1240720, EPI_ISL_1240721, EPI_ISL_1240722, EPI_ISL_1240723	Influenza and respiratory viruses	Human virology Department	A.Bensalem; A.Hachid; F.Derrar; F.Khardine; M.A.Beloufa
EPI_ISL_1093429	Influenza and respiratory viruses Lab. Pasteur Institute in Algeria	Human Virology Department	A.Hachid; F.A.Khardine; F.Derrar; M.A.Beloufa
EPI_ISL_1093427	Influenza and respiratory viruses lab. Pasteur Institute in Algeria	Human virology Department	A.Hachid; F.A.Khardine; F.Derrar; M.A. Beloufa
EPI_ISL_1093428	Influenza and respiratory viruses. Pasteur Institute in Algeria	Human virology deprtment	A.Hachid; F.A.Khardine; F.Derrar
EPI_ISL_1093430	Influenza and respiratory viurses Lab. Pasteur Institute in Algeria	Human Virology Department	A.Hachid; F.A.Khardine; F.Derrar; M.A.Beloufa
EPI_ISL_418215	Instirut Pasteur Dakar	Institut Pasteur de Dakar	Amadou Alpha Sall; Ndongo Dia; Ousmane Faye
EPI_ISL_485712	Institut Pasteur	Institut Pasteur de Dakar	Amadou Alpha Sall.; Mamadou Malado Jallow; Mamadou diop; Marie Henriette Dior Ndione; Moussa Moise Diagne; Ndongo Dia; Ousmane Faye; Safietou Sanke
EPI_ISL_418207, EPI_ISL_418208, EPI_ISL_418209, EPI_ISL_418210, EPI_ISL_418211, EPI_ISL_418212, EPI_ISL_418213, EPI_ISL_418214, EPI_ISL_418216, EPI_ISL_418217, EPI_ISL_420069, EPI_ISL_420070, EPI_ISL_420071, EPI_ISL_420072, EPI_ISL_420073, EPI_ISL_420074, EPI_ISL_420076, EPI_ISL_420077, EPI_ISL_420078, EPI_ISL_420079, EPI_ISL_476148, EPI_ISL_476149, EPI_ISL_476150, EPI_ISL_476151, EPI_ISL_476491, EPI_ISL_476492, EPI_ISL_476493, EPI_ISL_476494, EPI_ISL_476495, EPI_ISL_476497, EPI_ISL_476514, EPI_ISL_476515, EPI_ISL_476516, EPI_ISL_476558, EPI_ISL_476560, EPI_ISL_476562, EPI_ISL_476564, EPI_ISL_476569, EPI_ISL_476570, EPI_ISL_476572, EPI_ISL_476574, EPI_ISL_477141, EPI_ISL_477142, EPI_ISL_477143, EPI_ISL_477144, EPI_ISL_477145, EPI_ISL_477146, EPI_ISL_477147, EPI_ISL_477148, EPI_ISL_477149, EPI_ISL_477150, EPI_ISL_477151, EPI_ISL_477152, EPI_ISL_477153, EPI_ISL_477154, EPI_ISL_477155, EPI_ISL_477156, EPI_ISL_477157, EPI_ISL_477158, EPI_ISL_477159, EPI_ISL_480554, EPI_ISL_480555, EPI_ISL_480556, EPI_ISL_480782, EPI_ISL_480783, EPI_ISL_480786, EPI_ISL_480787, EPI_ISL_480788, EPI_ISL_480789, EPI_ISL_481220, EPI_ISL_481234, EPI_ISL_481235, EPI_ISL_481236, EPI_ISL_481237, EPI_ISL_481238, EPI_ISL_481239, EPI_ISL_481240, EPI_ISL_481243, EPI_ISL_482873, EPI_ISL_482874, EPI_ISL_482875, EPI_ISL_482876, EPI_ISL_482877, EPI_ISL_482878, EPI_ISL_485635, EPI_ISL_485707, EPI_ISL_485708, EPI_ISL_485710, EPI_ISL_485711, EPI_ISL_485713, EPI_ISL_485714, EPI_ISL_485715, EPI_ISL_485716, EPI_ISL_485717, EPI_ISL_485718, EPI_ISL_486857, EPI_ISL_486858, EPI_ISL_486859, EPI_ISL_486860, EPI_ISL_486861, EPI_ISL_486862, EPI_ISL_486863, EPI_ISL_486864, EPI_ISL_486865, EPI_ISL_486866, EPI_ISL_486867, EPI_ISL_486868, EPI_ISL_486869, EPI_ISL_486870, EPI_ISL_486871, EPI_ISL_486872, EPI_ISL_486873			
see above	Institut Pasteur Dakar	Institut Pasteur de Dakar	Amadou Alpha Sall; Amadou Alpha Sall.; Amadou Alpha sall; Amadou alpha Sall; Mamadou Diop; Mamadou Malado Jallow; Mamadou diop; Mamadou malado Jallow; Marie Henriette Dior Ndione; Moussa Moise; Moussa Moise Diagne; Ndongo Dia; Ousmane Faye; Ousmane faye; Safietou Sanke; Safietou Sankhe; mamadou Diop
EPI_ISL_498229, EPI_ISL_498230, EPI_ISL_498231, EPI_ISL_498232, EPI_ISL_498233, EPI_ISL_498234, EPI_ISL_498235, EPI_ISL_498236, EPI_ISL_498237, EPI_ISL_498238, EPI_ISL_498239, EPI_ISL_498240, EPI_ISL_498241, EPI_ISL_498242, EPI_ISL_498243, EPI_ISL_498244, EPI_ISL_498245, EPI_ISL_498246, EPI_ISL_498247, EPI_ISL_498248, EPI_ISL_498249, EPI_ISL_498250, EPI_ISL_498251, EPI_ISL_498252			
see above	Institut Pasteur de Dakar	Institut Pasteur de Dakar	Amadou Alpha Sall.; Mamadou Diop; Mamadou Malado Jallow; Marie Henriette Dior Ndione; Moussa Moise Diagne; Ndongo Dia; Ousmane Faye; Safietou Sankhe Mbengue
EPI_ISL_999030, EPI_ISL_999032	Institut Pasteur de Guinée	Institut Pasteur de Dakar	Dia Ndongo; Diagne Moussa Moïse; Diallo Amadou; Diop Mamadou; Faye Ousmane; Grayo Solene; Loucoubar Cheikh; Mbengue Safietou Sankhe; Ndiaye Ndack; Sall Amadou Alpha; Tordo Noel
EPI_ISL_459965, EPI_ISL_459966, EPI_ISL_459967, EPI_ISL_459968, EPI_ISL_459969, EPI_ISL_459970, EPI_ISL_459971, EPI_ISL_459972, EPI_ISL_459973, EPI_ISL_459974, EPI_ISL_459975, EPI_ISL_459976, EPI_ISL_459977, EPI_ISL_459978, EPI_ISL_459979, EPI_ISL_459980, EPI_ISL_459981, EPI_ISL_459982, EPI_ISL_459983, EPI_ISL_459984			
see above	Institut Pasteur du Maroc	Institut Pasteur du Maroc	Abdellah Faouzi; Anass Abbad; Anderrahmane Maaroufi; Angela Brisebarre; Camille Capel; Etienne Simon-Lorière; Jalal Nourlil; Latifa Anga; Marion Barbet; Maud Vanpeene; Mjid Eloualid; Méline Bizard; Sylvie Behillil; Sylvie van der Werf; Vincent Enouf
EPI_ISL_420075, EPI_ISL_476566	Institut pasteur Dakar	Institut Pasteur de Dakar	Amadou Alpha Sall; Mamadou Diop; Moussa Moise Diagne; Ndongo Dia; Ousmane Faye
EPI_ISL_887419, EPI_ISL_887420, EPI_ISL_887421, EPI_ISL_887422, EPI_ISL_887423, EPI_ISL_887424, EPI_ISL_887425, EPI_ISL_887426, EPI_ISL_887427, EPI_ISL_887428, EPI_ISL_887429, EPI_ISL_887430, EPI_ISL_887431, EPI_ISL_887432, EPI_ISL_887433, EPI_ISL_887434, EPI_ISL_887435, EPI_ISL_887436, EPI_ISL_887437, EPI_ISL_887438, EPI_ISL_887439, EPI_ISL_887440, EPI_ISL_887441, EPI_ISL_887442, EPI_ISL_887443, EPI_ISL_887444, EPI_ISL_887445, EPI_ISL_887446, EPI_ISL_887447, EPI_ISL_887448, EPI_ISL_887449, EPI_ISL_887450, EPI_ISL_887451, EPI_ISL_887452, EPI_ISL_887453, EPI_ISL_887454, EPI_ISL_887455, EPI_ISL_887456, EPI_ISL_887457, EPI_ISL_887458, EPI_ISL_887459, EPI_ISL_887460, EPI_ISL_887461, EPI_ISL_887462, EPI_ISL_887463, EPI_ISL_887464, EPI_ISL_887465, EPI_ISL_887466, EPI_ISL_887467, EPI_ISL_887468, EPI_ISL_887469, EPI_ISL_887470, EPI_ISL_887471, EPI_ISL_887472, EPI_ISL_887473, EPI_ISL_887474, EPI_ISL_887475, EPI_ISL_887476, EPI_ISL_887477, EPI_ISL_887478, EPI_ISL_887479, EPI_ISL_887480, EPI_ISL_887481, EPI_ISL_887482, EPI_ISL_887483, EPI_ISL_887484, EPI_ISL_887485, EPI_ISL_887486, EPI_ISL_887487, EPI_ISL_887488, EPI_ISL_887489, EPI_ISL_887490, EPI_ISL_887491, EPI_ISL_887492, EPI_ISL_887493, EPI_ISL_887494, EPI_ISL_887495, EPI_ISL_887496, EPI_ISL_887497, EPI_ISL_887498, EPI_ISL_887499, EPI_ISL_887500, EPI_ISL_887501, EPI_ISL_887502, EPI_ISL_887503, EPI_ISL_887504, EPI_ISL_887505, EPI_ISL_964918, EPI_ISL_964919, EPI_ISL_964920, EPI_ISL_964921, EPI_ISL_964922, EPI_ISL_964923, EPI_ISL_964924, EPI_ISL_964925, EPI_ISL_964926, EPI_ISL_964927, EPI_ISL_964928, EPI_ISL_964929, EPI_ISL_964930, EPI_ISL_964931, EPI_ISL_964932, EPI_ISL_964933, EPI_ISL_964934, EPI_ISL_964935, EPI_ISL_964936, EPI_ISL_964937, EPI_ISL_964938, EPI_ISL_964939, EPI_ISL_964940, EPI_ISL_964941, EPI_ISL_964942, EPI_ISL_964943, EPI_ISL_964944, EPI_ISL_964945, EPI_ISL_964946, EPI_ISL_964947, EPI_ISL_964948, EPI_ISL_1132818, EPI_ISL_1132819, EPI_ISL_1132820, EPI_ISL_1132821, EPI_ISL_1132822, EPI_ISL_1132823, EPI_ISL_1132824, EPI_ISL_1132825, EPI_ISL_1132826, EPI_ISL_1132827, EPI_ISL_1132828, EPI_ISL_1132829, EPI_ISL_1132830, EPI_ISL_1132831, EPI_ISL_1132832, EPI_ISL_1132833, EPI_ISL_1132834, EPI_ISL_1132835, EPI_ISL_1132836, EPI_ISL_1132837, EPI_ISL_1132838, EPI_ISL_1132839, EPI_ISL_1132840, EPI_ISL_1132841, EPI_ISL_1132842, EPI_ISL_1132843, EPI_ISL_1132844			
see above	Instituto Nacional de Saude (INS), Mozambique	KRISP, KZN Research Innovation and Sequencing Platform	Emmanuel S; Giandhari J; Nadia Sitoe; Nalia Ismael; Nedio Mabunda; Paulo Arnaldo; Pillay S; Tegally H; Wilkinson E; de Oliveira T
EPI_ISL_1167125, EPI_ISL_1167126, EPI_ISL_1167127, EPI_ISL_1167128, EPI_ISL_1167129, EPI_ISL_1167130, EPI_ISL_1167131, EPI_ISL_1167132, EPI_ISL_1167133, EPI_ISL_1167134, EPI_ISL_1167135, EPI_ISL_1167136, EPI_ISL_1167137, EPI_ISL_1167138, EPI_ISL_1167139, EPI_ISL_1167140, EPI_ISL_1167141, EPI_ISL_1167142, EPI_ISL_1167143, EPI_ISL_1167144, EPI_ISL_1167145, EPI_ISL_1167146, EPI_ISL_1167147, EPI_ISL_1167148, EPI_ISL_1167149, EPI_ISL_1167150, EPI_ISL_1167151, EPI_ISL_1167152, EPI_ISL_1167153, EPI_ISL_1167154, EPI_ISL_1167155, EPI_ISL_1167156, EPI_ISL_1167157, EPI_ISL_1167158, EPI_ISL_1167159, EPI_ISL_1167160, EPI_ISL_1167161, EPI_ISL_1167162, EPI_ISL_1167163, EPI_ISL_1167164, EPI_ISL_1167165, EPI_ISL_1167166, EPI_ISL_1167167, EPI_ISL_1167168, EPI_ISL_1167169, EPI_ISL_1167170, EPI_ISL_1167171, EPI_ISL_1167172, EPI_ISL_1167173, EPI_ISL_1167174, EPI_ISL_1167175, EPI_ISL_1167176, EPI_ISL_1167177, EPI_ISL_1167178, EPI_ISL_1167179, EPI_ISL_1167180, EPI_ISL_1167181, EPI_ISL_1167182, EPI_ISL_1167183, EPI_ISL_1167184			
see above	Iressef Genomics lab	L'institut de Recherche en Santé, de Surveillance Épidémiologique et de Formation (IRESSEF)	Abdou PADANE; Abdoulie KANTEH; Abdul Karim SESAY; Ambroise AHOUIDI; Aminata DIA; Aminata MBOUP; Astou Gaye GAYE; Barada CISSE; Birahim Piere NDIAYE; Gora LO; Khadim GUEYE; Moustapha MBOW; Nafisatou LEYE; Ndeye Coumba Toure KANE; Papa Alassane DIAW; Souleymane MBOUP; Yacine DIA
EPI_ISL_745135, EPI_ISL_745144	Joe Slovo Hospital	National Health Laboratory Service (NHLS), Tygerberg	Bronwyn Kleinhans; Eduan Wilkindon; Gert van Zyl; Houriiyah Tegally; Kayla Delaney; Susan Engelbrecht; Tulio de Oliveira; Wolfgang Preiser
EPI_ISL_457845, EPI_ISL_457846, EPI_ISL_457847, EPI_ISL_457848, EPI_ISL_457849, EPI_ISL_457850, EPI_ISL_457851, EPI_ISL_457852, EPI_ISL_457853, EPI_ISL_457854, EPI_ISL_457855, EPI_ISL_457856, EPI_ISL_457857, EPI_ISL_457858, EPI_ISL_457859, EPI_ISL_457860, EPI_ISL_457861, EPI_ISL_457862, EPI_ISL_457863, EPI_ISL_457864, EPI_ISL_457865, EPI_ISL_457866, EPI_ISL_457867, EPI_ISL_457868, EPI_ISL_457869, EPI_ISL_457870, EPI_ISL_457871, EPI_ISL_457872, EPI_ISL_457873, EPI_ISL_457874, EPI_ISL_457875, EPI_ISL_457876, EPI_ISL_457877, EPI_ISL_457878, EPI_ISL_457879, EPI_ISL_457880, EPI_ISL_457881, EPI_ISL_457882, EPI_ISL_457883, EPI_ISL_457884, EPI_ISL_457885, EPI_ISL_457886, EPI_ISL_457887, EPI_ISL_457888, EPI_ISL_457889, EPI_ISL_457890, EPI_ISL_457891, EPI_ISL_457892, EPI_ISL_457893, EPI_ISL_457894, EPI_ISL_457895, EPI_ISL_457896, EPI_ISL_457897, EPI_ISL_457898, EPI_ISL_457899, EPI_ISL_457900, EPI_ISL_457901, EPI_ISL_457902, EPI_ISL_457903, EPI_ISL_457904, EPI_ISL_457905, EPI_ISL_457906, EPI_ISL_457907, EPI_ISL_457908, EPI_ISL_457909, EPI_ISL_457910, EPI_ISL_457911, EPI_ISL_457912, EPI_ISL_457913, EPI_ISL_457914, EPI_ISL_457915, EPI_ISL_457916, EPI_ISL_457917, EPI_ISL_457918, EPI_ISL_457919, EPI_ISL_457920, EPI_ISL_457921, EPI_ISL_457922, EPI_ISL_457923, EPI_ISL_457924, EPI_ISL_457925, EPI_ISL_457926, EPI_ISL_457927, EPI_ISL_457928, EPI_ISL_457929, EPI_ISL_457930, EPI_ISL_457931			
see above	KEMRI-CGMR-C	KEMRI-Wellcome Trust Research Programme/KEMRI-CGMR-C Kilifi	Githinji G. et al 2020
EPI_ISL_457932, EPI_ISL_457933, EPI_ISL_457934, EPI_ISL_457935, EPI_ISL_457936	KEMRI-Centre for Virus Research	KEMRI-Wellcome Trust Research Programme/KEMRI-CGMR-C Kilifi	Githinji G. et al 2020
EPI_ISL_568695, EPI_ISL_568696, EPI_ISL_568697, EPI_ISL_568698, EPI_ISL_568699, EPI_ISL_568700, EPI_ISL_568701, EPI_ISL_568702, EPI_ISL_568703, EPI_ISL_568704, EPI_ISL_568705, EPI_ISL_568706, EPI_ISL_568707, EPI_ISL_568708, EPI_ISL_568709, EPI_ISL_568710, EPI_ISL_568711, EPI_ISL_568712, EPI_ISL_568713, EPI_ISL_568714, EPI_ISL_568715, EPI_ISL_568716, EPI_ISL_568717, EPI_ISL_568718, EPI_ISL_568719, EPI_ISL_568720, EPI_ISL_568721, EPI_ISL_568722, EPI_ISL_568723, EPI_ISL_568724, EPI_ISL_568725, EPI_ISL_568726, EPI_ISL_568727, EPI_ISL_568728, EPI_ISL_568729, EPI_ISL_568730, EPI_ISL_568731, EPI_ISL_568732, EPI_ISL_568733, EPI_ISL_568734, EPI_ISL_568735, EPI_ISL_568736, EPI_ISL_568737, EPI_ISL_568738, EPI_ISL_568739, EPI_ISL_568740, EPI_ISL_568741, EPI_ISL_568742, EPI_ISL_568743, EPI_ISL_568744, EPI_ISL_568745, EPI_ISL_568746, EPI_ISL_568747, EPI_ISL_568748, EPI_ISL_568749, EPI_ISL_568750, EPI_ISL_568751, EPI_ISL_568752, EPI_ISL_568753, EPI_ISL_568754, EPI_ISL_568755, EPI_ISL_568756, EPI_ISL_568757, EPI_ISL_568758, EPI_ISL_568759, EPI_ISL_568760, EPI_ISL_568761, EPI_ISL_568762, EPI_ISL_568763, EPI_ISL_568764, EPI_ISL_568765, EPI_ISL_568766, EPI_ISL_568767, EPI_ISL_568768, EPI_ISL_568769, EPI_ISL_568770, EPI_ISL_568771, EPI_ISL_568772, EPI_ISL_568773, EPI_ISL_568774, EPI_ISL_568775, EPI_ISL_568776, EPI_ISL_568777, EPI_ISL_568778, EPI_ISL_568779, EPI_ISL_568780, EPI_ISL_568781, EPI_ISL_568782, EPI_ISL_568783, EPI_ISL_568784, EPI_ISL_568785, EPI_ISL_568786, EPI_ISL_568787, EPI_ISL_568788, EPI_ISL_568789, EPI_ISL_568790, EPI_ISL_568791, EPI_ISL_568792, EPI_ISL_568793, EPI_ISL_568794, EPI_ISL_568795, EPI_ISL_568796, EPI_ISL_568797, EPI_ISL_568798, EPI_ISL_568799, EPI_ISL_568800, EPI_ISL_568801, EPI_ISL_568802, EPI_ISL_568803, EPI_ISL_568804, EPI_ISL_568805, EPI_ISL_568806, EPI_ISL_568807, EPI_ISL_568808, EPI_ISL_568809, EPI_ISL_568810, EPI_ISL_568811, EPI_ISL_568812, EPI_ISL_568813, EPI_ISL_568814, EPI_ISL_568815, EPI_ISL_568816, EPI_ISL_568817, EPI_ISL_568818, EPI_ISL_568819, EPI_ISL_568820, EPI_ISL_568821, EPI_ISL_568822, EPI_ISL_568823, EPI_ISL_568824, EPI_ISL_568825, EPI_ISL_568826, EPI_ISL_568827, EPI_ISL_568828, EPI_ISL_568829, EPI_ISL_568830, EPI_ISL_568831, EPI_ISL_568832, EPI_ISL_568833, EPI_ISL_568834, EPI_ISL_568835, EPI_ISL_568836, EPI_ISL_568837, EPI_ISL_568838, EPI_ISL_568839, EPI_ISL_568840, EPI_ISL_568841, EPI_ISL_568842, EPI_ISL_568843, EPI_ISL_568844, EPI_ISL_568845, EPI_ISL_568846, EPI_ISL_568847, EPI_ISL_568848, EPI_ISL_568849, EPI_ISL_568850, EPI_ISL_568851, EPI_ISL_568852, EPI_ISL_568853, EPI_ISL_568854, EPI_ISL_568855, EPI_ISL_568856, EPI_ISL_568857, EPI_ISL_568858, EPI_ISL_568859, EPI_ISL_568860, EPI_ISL_568861, EPI_ISL_568862,			

EPI_ISL_568863, EPI_ISL_568864, EPI_ISL_568865, EPI_ISL_568866, EPI_ISL_568867, EPI_ISL_568868, EPI_ISL_568869, EPI_ISL_568870, EPI_ISL_568871, EPI_ISL_568872, EPI_ISL_806548, EPI_ISL_806549, EPI_ISL_806550, EPI_ISL_806551, EPI_ISL_806552, EPI_ISL_806553, EPI_ISL_806554, EPI_ISL_806555, EPI_ISL_806556, EPI_ISL_806557, EPI_ISL_806558, EPI_ISL_806559, EPI_ISL_806560, EPI_ISL_806561, EPI_ISL_806562, EPI_ISL_806563, EPI_ISL_806564, EPI_ISL_806565, EPI_ISL_806566, EPI_ISL_806567, EPI_ISL_806568, EPI_ISL_806569, EPI_ISL_806570, EPI_ISL_806571, EPI_ISL_806572, EPI_ISL_806573, EPI_ISL_806574, EPI_ISL_806575, EPI_ISL_806576, EPI_ISL_806577, EPI_ISL_806578, EPI_ISL_806579, EPI_ISL_806580, EPI_ISL_806581, EPI_ISL_806582, EPI_ISL_806583, EPI_ISL_806584, EPI_ISL_806585, EPI_ISL_806586, EPI_ISL_806587, EPI_ISL_806588, EPI_ISL_806589, EPI_ISL_806590, EPI_ISL_806591, EPI_ISL_806592, EPI_ISL_806593, EPI_ISL_806594, EPI_ISL_806595, EPI_ISL_806596, EPI_ISL_806597, EPI_ISL_806598, EPI_ISL_806599, EPI_ISL_806600, EPI_ISL_806601, EPI_ISL_806602, EPI_ISL_806603, EPI_ISL_806604, EPI_ISL_806605, EPI_ISL_806606, EPI_ISL_806607, EPI_ISL_806608, EPI_ISL_806609, EPI_ISL_806610, EPI_ISL_806611, EPI_ISL_806612, EPI_ISL_806613, EPI_ISL_806614, EPI_ISL_806615, EPI_ISL_806616, EPI_ISL_806617, EPI_ISL_806618, EPI_ISL_806619, EPI_ISL_806620, EPI_ISL_806621, EPI_ISL_806623, EPI_ISL_806624, EPI_ISL_806625, EPI_ISL_806626, EPI_ISL_806627, EPI_ISL_806628, EPI_ISL_806629, EPI_ISL_806630, EPI_ISL_806631, EPI_ISL_806632, EPI_ISL_806633, EPI_ISL_806634, EPI_ISL_806635, EPI_ISL_806636, EPI_ISL_806637, EPI_ISL_806638, EPI_ISL_806639, EPI_ISL_806640, EPI_ISL_806641, EPI_ISL_806642, EPI_ISL_806643, EPI_ISL_806644, EPI_ISL_806645, EPI_ISL_806646, EPI_ISL_806647, EPI_ISL_806648, EPI_ISL_806649, EPI_ISL_806650, EPI_ISL_806651, EPI_ISL_806652, EPI_ISL_806653, EPI_ISL_806654, EPI_ISL_806655, EPI_ISL_806656, EPI_ISL_806657, EPI_ISL_806658, EPI_ISL_806659, EPI_ISL_806660, EPI_ISL_806661, EPI_ISL_806662, EPI_ISL_806663, EPI_ISL_806664, EPI_ISL_806665, EPI_ISL_806666, EPI_ISL_806667, EPI_ISL_806668, EPI_ISL_806669, EPI_ISL_806670, EPI_ISL_806671, EPI_ISL_806672, EPI_ISL_806673, EPI_ISL_806674, EPI_ISL_806675, EPI_ISL_806676, EPI_ISL_806677, EPI_ISL_806678, EPI_ISL_806679, EPI_ISL_806680, EPI_ISL_806681, EPI_ISL_806682, EPI_ISL_806683, EPI_ISL_806684, EPI_ISL_806685, EPI_ISL_806686, EPI_ISL_806687, EPI_ISL_806688, EPI_ISL_806689, EPI_ISL_806690, EPI_ISL_806691, EPI_ISL_806692, EPI_ISL_806693, EPI_ISL_806694, EPI_ISL_806695, EPI_ISL_806696, EPI_ISL_806697, EPI_ISL_806698, EPI_ISL_806699, EPI_ISL_806700, EPI_ISL_806701, EPI_ISL_806702, EPI_ISL_806703, EPI_ISL_806704, EPI_ISL_806705, EPI_ISL_806706, EPI_ISL_806707, EPI_ISL_806708, EPI_ISL_806709, EPI_ISL_806710, EPI_ISL_806711, EPI_ISL_806712, EPI_ISL_806713, EPI_ISL_806714, EPI_ISL_806715, EPI_ISL_806716, EPI_ISL_806717, EPI_ISL_855494, EPI_ISL_855495, EPI_ISL_855496, EPI_ISL_855497, EPI_ISL_855498, EPI_ISL_855499, EPI_ISL_855500, EPI_ISL_855501, EPI_ISL_855502, EPI_ISL_855503, EPI_ISL_855504, EPI_ISL_855505, EPI_ISL_855506, EPI_ISL_855507, EPI_ISL_855508, EPI_ISL_855509, EPI_ISL_855510, EPI_ISL_855511, EPI_ISL_855512, EPI_ISL_855513, EPI_ISL_855514, EPI_ISL_855515, EPI_ISL_855516, EPI_ISL_855517, EPI_ISL_855518, EPI_ISL_855519, EPI_ISL_855520, EPI_ISL_855521, EPI_ISL_855522, EPI_ISL_855523, EPI_ISL_855524, EPI_ISL_855525, EPI_ISL_855526, EPI_ISL_855527, EPI_ISL_855528, EPI_ISL_855529, EPI_ISL_855530, EPI_ISL_855531, EPI_ISL_855533, EPI_ISL_855534, EPI_ISL_855535, EPI_ISL_855536, EPI_ISL_855537, EPI_ISL_855538, EPI_ISL_855539, EPI_ISL_855540, EPI_ISL_855541, EPI_ISL_855542, EPI_ISL_855543, EPI_ISL_855544, EPI_ISL_855545, EPI_ISL_855546, EPI_ISL_855547, EPI_ISL_855548, EPI_ISL_968807, EPI_ISL_968808, EPI_ISL_968810, EPI_ISL_968813, EPI_ISL_968815, EPI_ISL_968817, EPI_ISL_968819, EPI_ISL_968820, EPI_ISL_968821, EPI_ISL_968822, EPI_ISL_968825, EPI_ISL_968826, EPI_ISL_968827, EPI_ISL_968828, EPI_ISL_968829, EPI_ISL_968831, EPI_ISL_968832, EPI_ISL_968834, EPI_ISL_968835, EPI_ISL_968837, EPI_ISL_968838, EPI_ISL_968839, EPI_ISL_968841, EPI_ISL_968842, EPI_ISL_968843, EPI_ISL_968844, EPI_ISL_968846, EPI_ISL_968847, EPI_ISL_968849, EPI_ISL_968850, EPI_ISL_968851, EPI_ISL_968853, EPI_ISL_968854, EPI_ISL_968855, EPI_ISL_968856, EPI_ISL_968858, EPI_ISL_968860, EPI_ISL_968861, EPI_ISL_968863, EPI_ISL_968864, EPI_ISL_968865, EPI_ISL_968868, EPI_ISL_968869, EPI_ISL_968871, EPI_ISL_968873, EPI_ISL_968877, EPI_ISL_968878, EPI_ISL_968881, EPI_ISL_968882, EPI_ISL_968883, EPI_ISL_968884, EPI_ISL_968885, EPI_ISL_968887, EPI_ISL_968889, EPI_ISL_968890, EPI_ISL_968891, EPI_ISL_968892, EPI_ISL_968894, EPI_ISL_968896, EPI_ISL_968897, EPI_ISL_968898, EPI_ISL_968899, EPI_ISL_968901, EPI_ISL_968902, EPI_ISL_968903, EPI_ISL_968904, EPI_ISL_968905, EPI_ISL_968907, EPI_ISL_968908, EPI_ISL_968909, EPI_ISL_968910, EPI_ISL_968911, EPI_ISL_968913, EPI_ISL_968914, EPI_ISL_968916, EPI_ISL_968919, EPI_ISL_968920, EPI_ISL_968922, EPI_ISL_968924, EPI_ISL_968925, EPI_ISL_968926, EPI_ISL_968927, EPI_ISL_968929, EPI_ISL_968930, EPI_ISL_968941, EPI_ISL_968942, EPI_ISL_968943, EPI_ISL_968955, EPI_ISL_968956, EPI_ISL_968958, EPI_ISL_968959, EPI_ISL_968961, EPI_ISL_968972, EPI_ISL_968984, EPI_ISL_968985, EPI_ISL_968986, EPI_ISL_968987, EPI_ISL_968988, EPI_ISL_968989, EPI_ISL_968990, EPI_ISL_968992, EPI_ISL_968993, EPI_ISL_968996, EPI_ISL_968997, EPI_ISL_968998, EPI_ISL_968999, EPI_ISL_969001, EPI_ISL_969002, EPI_ISL_969003, EPI_ISL_969004, EPI_ISL_969005, EPI_ISL_969007, EPI_ISL_969008, EPI_ISL_969009, EPI_ISL_969010, EPI_ISL_969011, EPI_ISL_969012, EPI_ISL_969013, EPI_ISL_969014, EPI_ISL_969016, EPI_ISL_969017, EPI_ISL_969018, EPI_ISL_969019, EPI_ISL_969020, EPI_ISL_969021, EPI_ISL_969023, EPI_ISL_969024, EPI_ISL_969025, EPI_ISL_969026, EPI_ISL_969027, EPI_ISL_969028, EPI_ISL_969029, EPI_ISL_969030, EPI_ISL_969031, EPI_ISL_969041, EPI_ISL_969042, EPI_ISL_969043, EPI_ISL_969044, EPI_ISL_969046, EPI_ISL_969047, EPI_ISL_969048, EPI_ISL_969049, EPI_ISL_969050, EPI_ISL_969051, EPI_ISL_969052, EPI_ISL_969053, EPI_ISL_969054, EPI_ISL_969056, EPI_ISL_969057, EPI_ISL_969058, EPI_ISL_969059, EPI_ISL_969060, EPI_ISL_969061, EPI_ISL_969063, EPI_ISL_969064, EPI_ISL_969065, EPI_ISL_969066, EPI_ISL_969067, EPI_ISL_969068, EPI_ISL_969069, EPI_ISL_969071, EPI_ISL_969072, EPI_ISL_969073, EPI_ISL_969074, EPI_ISL_969245, EPI_ISL_1039223, EPI_ISL_1039224, EPI_ISL_1039225, EPI_ISL_1039226, EPI_ISL_1039227, EPI_ISL_1039228, EPI_ISL_1039229			
see above	KEMRI-Wellcome Trust Research Programme/KEMRI-CGMR-C Kilifi	KEMRI-Wellcome Trust Research Programme/KEMRI-CGMR-C Kilifi	Githinji et al; Githinji et al 2020
EPI_ISL_436684, EPI_ISL_436685, EPI_ISL_436686, EPI_ISL_436687, EPI_ISL_455629, EPI_ISL_455630, EPI_ISL_455631, EPI_ISL_455632, EPI_ISL_455633, EPI_ISL_455634, EPI_ISL_455635, EPI_ISL_455636, EPI_ISL_455637, EPI_ISL_455638, EPI_ISL_455639, EPI_ISL_660221, EPI_ISL_660222, EPI_ISL_660228, EPI_ISL_660229, EPI_ISL_660230, EPI_ISL_660231, EPI_ISL_660232, EPI_ISL_660233, EPI_ISL_660256, EPI_ISL_660257, EPI_ISL_660258, EPI_ISL_660664			
see above	KRISP, KZN Research Innovation and Sequencing Platform	KRISP, KZN Research Innovation and Sequencing Platform	Chimukangara B; Deforche K; Giandhari J; Khan S; Lessells R; Mdlalose K; Pillay S; Tegally H; Wilkinson E; York D; de Oliveira T
EPI_ISL_1073037, EPI_ISL_1075644	Kasane Primary Hospital Laboratory	Botswana Harvard HIV Reference Laboratory	Boitumelo Zuze; Botshelo Radibe; David Lawrence; Dorcas Maruapula; Joseph; Joseph Makhema; Legodile Kooepile; Makhema; Mosepele Mosepele; Roger Shapiro; Shahin Lockman; Sikhulile Moyo; Simani Gaseitsiwe; Wonderful T. Choga
EPI_ISL_512811, EPI_ISL_512812, EPI_ISL_512813, EPI_ISL_512815, EPI_ISL_512816, EPI_ISL_512817, EPI_ISL_512818, EPI_ISL_512819, EPI_ISL_512820, EPI_ISL_512821			
see above	Kenema Government Hospital, Ministry of Health and Sanitation	Kenema Government Hospital, Ministry of Health and Sanitation	Andersen, K.; Garry, R.; Goba, A.; Grant, D.; Happi, C.; Jalloh, S.; Mehta, S.; Momoh, M.; Olawoye, I.; Oluniyi, P.; Park, D.; Sandi, J.; Siddle, K.; Tomkins-Tinch, C.
EPI_ISL_1040760	Kensington CDC	NHLS/UCT	Arash Iranzadeh; Bruna Galvao; Carolyn Williamson; Deelan Doolabh; Diana Hardie; Innocent Mudau; Kruger Marais; Lynn Tyers; Marvin Hsiao; Stephen Korsman
EPI_ISL_640092, EPI_ISL_640093	Kensington CDC wc KSC	NHLS/UCT	Arash Iranzadeh; Bruna Galvao; Carolyn Williamson; Deelan Doolabh; Diana Hardie; Innocent Mudau; Kruger Marais; Lynn Tyers; Marvin Hsiao; Stephen Korsman
EPI_ISL_696511	Keurhoek Clinic wc KTT & NHLS/UCT	KRISP, KZN Research Innovation and Sequencing Platform	Arash Iranzadeh; Bruna Galvao; Carolyn Williamson; Deelan Doolabh; Diana Hardie; Emanuel James San; Houriiyah Tegally; Innocent Mudau; Jennifer Giandhari; Kruger Marais; Lynn Tyers; Marvin Hsiao; Stephen Korsman; Sureshnee Pillay; Tulio de Oliveira
EPI_ISL_700423, EPI_ISL_700464, EPI_ISL_700468, EPI_ISL_700485, EPI_ISL_700505, EPI_ISL_700540	Khayelethu Clinic wc KLC	NHLS/UCT	Arash Iranzadeh; Bruna Galvao; Carolyn Williamson; Deelan Doolabh; Diana Hardie; Houriiyah Tegally; Innocent Mudau; Kruger Marais; Lynn Tyers; Marvin Hsiao; Stephen Korsman
EPI_ISL_696484, EPI_ISL_696505	Khayelethu Clinic wc KLC & NHLS/UCT	KRISP, KZN Research Innovation and Sequencing Platform	Arash Iranzadeh; Bruna Galvao; Carolyn Williamson; Deelan Doolabh; Diana Hardie; Emanuel James San; Houriiyah Tegally; Innocent Mudau; Jennifer Giandhari; Kruger Marais; Lynn Tyers; Marvin Hsiao; Stephen Korsman; Sureshnee Pillay; Tulio de Oliveira
EPI_ISL_640083	Khayelitsha (Site B) CHC wc KHC	NHLS/UCT	Arash Iranzadeh; Bruna Galvao; Carolyn Williamson; Deelan Doolabh; Diana Hardie; Innocent Mudau; Kruger Marais; Lynn Tyers; Marvin Hsiao; Stephen Korsman
EPI_ISL_745111, EPI_ISL_745127, EPI_ISL_745163	Khayelitsha (Site B) CHC wc KHC	National Health Laboratory Service (NHLS), Tygerberg	Bronwyn Kleinhans; Eduan Wilkindon; Gert van Zyl; Houriiyah Tegally; Kayla Delaney; Susan Engelbrecht; Tulio de Oliveira; Wolfgang Preiser
EPI_ISL_745114, EPI_ISL_745115, EPI_ISL_745116, EPI_ISL_745120, EPI_ISL_745122, EPI_ISL_745123, EPI_ISL_745145, EPI_ISL_745166			
see above	Khayelitsha Hospital wc KHA	National Health Laboratory Service (NHLS), Tygerberg	Bronwyn Kleinhans; Eduan Wilkindon; Gert van Zyl; Houriiyah Tegally; Kayla Delaney; Susan Engelbrecht; Tulio de Oliveira; Wolfgang Preiser
EPI_ISL_1239225, EPI_ISL_1239226, EPI_ISL_1239227, EPI_ISL_1239228, EPI_ISL_1239229, EPI_ISL_1239230, EPI_ISL_1239231, EPI_ISL_1239232, EPI_ISL_1239233, EPI_ISL_1239234, EPI_ISL_1239235, EPI_ISL_1239236, EPI_ISL_1239237, EPI_ISL_1239238, EPI_ISL_1239239, EPI_ISL_1239240, EPI_ISL_1239241, EPI_ISL_1239242, EPI_ISL_1239243, EPI_ISL_1239244, EPI_ISL_1239245, EPI_ISL_1239246, EPI_ISL_1239247, EPI_ISL_1239248, EPI_ISL_1239249, EPI_ISL_1239250, EPI_ISL_1239251, EPI_ISL_1239252, EPI_ISL_1239253, EPI_ISL_1239254, EPI_ISL_1239255, EPI_ISL_1239256, EPI_ISL_1239257, EPI_ISL_1239258, EPI_ISL_1239259, EPI_ISL_1239260, EPI_ISL_1239261, EPI_ISL_1239262, EPI_ISL_1239263, EPI_ISL_1239264, EPI_ISL_1239265, EPI_ISL_1239266, EPI_ISL_1239458, EPI_ISL_1239460, EPI_ISL_1239461, EPI_ISL_1239463, EPI_ISL_1239466, EPI_ISL_1239467, EPI_ISL_1239468, EPI_ISL_1239471, EPI_ISL_1239474, EPI_ISL_1239475, EPI_ISL_1239476, EPI_ISL_1239477, EPI_ISL_1239478, EPI_ISL_1239480, EPI_ISL_1239481, EPI_ISL_1239483, EPI_ISL_1239485, EPI_ISL_1239486, EPI_ISL_1239488, EPI_ISL_1239489, EPI_ISL_1239508, EPI_ISL_1239512, EPI_ISL_1239518, EPI_ISL_1239519, EPI_ISL_1239520, EPI_ISL_1239522, EPI_ISL_1239527, EPI_ISL_1239529, EPI_ISL_1239531			
see above	Kimberley Hospital, National Health Laboratory Services, Northern Cape, South Africa	National Institute for Communicable Diseases of the National Health Laboratory Service	Amoako DG; Bhiman JN; Ismail A; Mahlangu B; Mohale T; Ntuli N; Scheepers C
EPI_ISL_700433, EPI_ISL_700436, EPI_ISL_700440, EPI_ISL_700450, EPI_ISL_700469, EPI_ISL_700474, EPI_ISL_700526, EPI_ISL_700529, EPI_ISL_700531, EPI_ISL_700542			
see above	Knysna CDC wc WLC	NHLS/UCT	Arash Iranzadeh; Bruna Galvao; Carolyn Williamson; Deelan Doolabh; Diana Hardie; Houriiyah Tegally; Innocent Mudau; Kruger Marais; Lynn Tyers; Marvin Hsiao; Stephen Korsman
EPI_ISL_696467, EPI_ISL_696495, EPI_ISL_696506	Knysna CDC wc WLC & NHLS/UCT	KRISP, KZN Research Innovation and Sequencing Platform	Arash Iranzadeh; Bruna Galvao; Carolyn Williamson; Deelan Doolabh; Diana Hardie; Emanuel James San; Houriiyah Tegally; Innocent Mudau; Jennifer Giandhari; Kruger Marais; Lynn Tyers; Marvin Hsiao; Stephen Korsman; Sureshnee Pillay; Tulio de Oliveira
EPI_ISL_640022, EPI_ISL_640038, EPI_ISL_640076, EPI_ISL_700419, EPI_ISL_700422, EPI_ISL_700426, EPI_ISL_700429, EPI_ISL_700456, EPI_ISL_700484, EPI_ISL_700501, EPI_ISL_700509, EPI_ISL_700510, EPI_ISL_700583			
see above	Knysna Hospital wc KNY	NHLS/UCT	Arash Iranzadeh; Bruna Galvao; Carolyn Williamson; Deelan Doolabh; Diana Hardie; Houriiyah Tegally; Innocent Mudau; Kruger Marais; Lynn Tyers; Marvin Hsiao; Stephen Korsman
EPI_ISL_960120	Knysna Hospital wc KNY	National Health Laboratory Service/UCT	Arash Iranzadeh; Bruna Galvao; Carolyn Williamson; Deelan Doolabh; Diana Hardie; Innocent Mudau; Kruger Marais; Lynn Tyers; Marvin Hsiao; Stephen Korsman
EPI_ISL_696463, EPI_ISL_696469, EPI_ISL_696477, EPI_ISL_696478, EPI_ISL_696482, EPI_ISL_696491	Knysna Hospital wc KNY & NHLS/UCT	KRISP, KZN Research Innovation and Sequencing Platform	Arash Iranzadeh; Bruna Galvao; Carolyn Williamson; Deelan Doolabh; Diana Hardie; Emanuel James San; Houriiyah Tegally; Innocent Mudau; Jennifer Giandhari; Kruger Marais; Lynn Tyers; Marvin Hsiao; Stephen Korsman; Sureshnee Pillay; Tulio de Oliveira
EPI_ISL_745109, EPI_ISL_745131, EPI_ISL_745133, EPI_ISL_745136, EPI_ISL_745164, EPI_ISL_745179	Kraaifontein CHC wc KFP	National Health Laboratory Service (NHLS), Tygerberg	Bronwyn Kleinhans; Eduan Wilkindon; Gert van Zyl; Houriiyah Tegally; Kayla Delaney; Susan Engelbrecht; Tulio de Oliveira; Wolfgang Preiser
EPI_ISL_696460, EPI_ISL_696498	Kranshoek Clinic wc KSH & NHLS/UCT	KRISP, KZN Research Innovation and Sequencing Platform	Arash Iranzadeh; Bruna Galvao; Carolyn Williamson; Deelan Doolabh; Diana Hardie; Emanuel James San; Houriiyah Tegally; Innocent Mudau; Jennifer Giandhari; Kruger Marais; Lynn Tyers; Marvin Hsiao; Stephen Korsman; Sureshnee Pillay; Tulio de Oliveira
EPI_ISL_515181, EPI_ISL_515182, EPI_ISL_515183, EPI_ISL_515184, EPI_ISL_515247, EPI_ISL_515248, EPI_ISL_515249			
see above	Kumasi Centre for Collaborative Research in Tropical Medicine, Kumasi.	Institute of Virology, Charité – Universitätsmedizin Berlin	Augustina Sylverken; Christian Drosten; Eric Adu; Jesse Addo Asamoah; Julia Schneider; Jörn Beheim-Schwarzbach; Michael Owusu; Philip El-Duah; Richard Phillips.; Richmond Gorman; Richmond Yeboah; Sherihane Aryeetey; Victor Max Corman
EPI_ISL_640030, EPI_ISL_700532, EPI_ISL_1040674	Kwamandlenkosi Clinic wc KMN	NHLS/UCT	Arash Iranzadeh; Bruna Galvao; Carolyn Williamson; Deelan Doolabh; Diana Hardie; Innocent Mudau; Kruger Marais; Lynn Tyers; Marvin Hsiao; Stephen Korsman
EPI_ISL_700437, EPI_ISL_700495, EPI_ISL_700535, EPI_ISL_700537, EPI_ISL_700541, EPI_ISL_700579	Kwanokuthula CDC wc KWA	NHLS/UCT	Arash Iranzadeh; Bruna Galvao; Carolyn Williamson; Deelan Doolabh; Diana Hardie; Houriiyah Tegally; Innocent Mudau; Kruger Marais; Lynn Tyers; Marvin Hsiao; Stephen Korsman
EPI_ISL_960123	Kwanokuthula CDC wc KWA	National Health Laboratory Service/UCT	Arash Iranzadeh; Bruna Galvao; Carolyn Williamson; Deelan Doolabh; Diana Hardie; Innocent Mudau; Kruger Marais; Lynn Tyers; Marvin Hsiao; Stephen Korsman
EPI_ISL_696465	Kwanokuthula CDC wc KWA & NHLS/UCT	KRISP, KZN Research Innovation and Sequencing Platform	Arash Iranzadeh; Bruna Galvao; Carolyn Williamson; Deelan Doolabh; Diana Hardie; Emanuel James San; Houriiyah Tegally; Innocent Mudau; Jennifer Giandhari; Kruger Marais; Lynn Tyers; Marvin Hsiao; Stephen Korsman; Sureshnee Pillay; Tulio de Oliveira
EPI_ISL_469017, EPI_ISL_469049, EPI_ISL_469051, EPI_ISL_469052, EPI_ISL_469053, EPI_ISL_469054, EPI_ISL_482732, EPI_ISL_482733, EPI_ISL_482734, EPI_ISL_482735, EPI_ISL_482736, EPI_ISL_482737, EPI_ISL_482738, EPI_ISL_482739, EPI_ISL_482740, EPI_ISL_728221, EPI_ISL_728222, EPI_ISL_728223, EPI_ISL_728224, EPI_ISL_728235, EPI_ISL_728236, EPI_ISL_728237			
see above	LNR National Reference Laboratory, Mohammed VI University of Health Sciences	Medical Biotechnology Laboratory, Rabat Medical and Pharmacy School, Mohammed The Vth University in Rabat	Adib Ghassan; Chakib NEJJARI; Houda BENRAHMA; Housna ARROUCHI; Idrissa DIAWARA; Idrissa Diawara; Imane SMYEJ; Jalil El ATAR; Jalil El Atar; Jalila RAHOUI; Lahcen BELYAMANI and Azeddine IBRAHIMI; Laila SBABOU; Laila SBABOU; Loubna ALLAM; M.W. CHEMAO-ELFIHRI; Meriem LAAMARTI; Mouna OUADGHIRI; Mouneem Essabbar; Nabila Soara; Rachid EL JAOUDI; Rachid MENTAG; Rokaia LAAMRTI; Rokia LAAMARTI; Saaid AMZAZI; Souad KARTTI; Tarek Aanniz

EPI_ISL_723469, EPI_ISL_728219, EPI_ISL_728272, EPI_ISL_728273, EPI_ISL_728274, EPI_ISL_728275, EPI_ISL_728276, EPI_ISL_728277, EPI_ISL_728289, EPI_ISL_728290, EPI_ISL_728291, EPI_ISL_728295, EPI_ISL_728297, EPI_ISL_728301, EPI_ISL_728322, EPI_ISL_728332, EPI_ISL_728334, EPI_ISL_728336, EPI_ISL_728339, EPI_ISL_728340, EPI_ISL_728342, EPI_ISL_728344, EPI_ISL_728347, EPI_ISL_728349, EPI_ISL_728352, EPI_ISL_728353, EPI_ISL_728355, EPI_ISL_728359, EPI_ISL_728360, EPI_ISL_728363, EPI_ISL_728366, EPI_ISL_728367, EPI_ISL_768831, EPI_ISL_768832, EPI_ISL_768834, EPI_ISL_768835, EPI_ISL_768836, EPI_ISL_768837, EPI_ISL_768839, EPI_ISL_768840, EPI_ISL_769863, EPI_ISL_775219, EPI_ISL_775220, EPI_ISL_775221, EPI_ISL_775222, EPI_ISL_775223, EPI_ISL_775224, EPI_ISL_775225, EPI_ISL_775226, EPI_ISL_775247, EPI_ISL_775249, EPI_ISL_775250, EPI_ISL_775252, EPI_ISL_775254, EPI_ISL_775256, EPI_ISL_775258, EPI_ISL_775260, EPI_ISL_775262, EPI_ISL_775263, EPI_ISL_775264, EPI_ISL_775265			
see above	Laboratoire Biolife	Laboratoire de Biotechnologie	Afaf Alaoui; Amina Benouda; Bouchra Belfquih; Hanae Dakka; Lahcen Belyamani; Lahcen belyamani; Lahecn Belyamani; Mohamed Chenaoui; Mohammed Walid Chemao Elfihri; Mouna Ouadghiri; Otmane Touzani; Saaid Amzazi and Azeddine Ibrahim; Saaid Amzazi and Azeddine Ibrahimi; Tarik Aanniz
EPI_ISL_1103576, EPI_ISL_1107189, EPI_ISL_1109622, EPI_ISL_1109623, EPI_ISL_1116465, EPI_ISL_1116466, EPI_ISL_1116470, EPI_ISL_1116555, EPI_ISL_1116592, EPI_ISL_1117289, EPI_ISL_1120732, EPI_ISL_1137621, EPI_ISL_1159697, EPI_ISL_1159698, EPI_ISL_1159699, EPI_ISL_1159700			
see above	Laboratoire central de Virologie	Laboratoire de Biotechnologie	Abdelomunim Essabbar; Amal Zouaki; Ghizlane EL Amin; Hakima Kabbaj; Lahcen Belyamani and Azeddine Ibrahim; Lahcen Belyamani and Azeddine Ibrahimi; Mouna Ouadghiri; Myriam Seffar; Saaïd Amzazi; Tarik Aanniz
EPI_ISL_1103584	Laboratoire central de Virologie, Centre hospitalo-universitaire Ibn Sina, Hôpital des spécialités	Laboratoire de Biotechnologie	Abdelomunim Essabbar; Amal Zouaki; Ghizlane EL Amin; Hakima Kabbaj; Lahcen Belyamani and Azeddine Ibrahimi; Mouna Ouadghiri; Myriam Seffar; Saaïd Amzazi; Tarik Aanniz
EPI_ISL_1105946	Laboratoire central de Virologie, Centre hospitalo-universitaire Ibn Sina Hôpital des spécialités	Laboratoire de Biotechnologie	Abdelomunim Essabbar; Amal Zouaki; Ghizlane EL Amin; Hakima Kabbaj; Lahcen Belyamani and Azeddine Ibrahimi; Mouna Ouadghiri; Myriam Seffar; Saaïd Amzazi; Tarik Aanniz
EPI_ISL_1103579	Laboratoire central de Virologie, Centre hospitalo-universitaire Ibn Sina, Hôpital des spécialités	Laboratoire de Biotechnologie	Abdelomunim Essabbar; Amal Zouaki; Ghizlane EL Amin; Hakima Kabbaj; Lahcen Belyamani and Azeddine Ibrahimi; Mouna Ouadghiri; Myriam Seffar; Saaïd Amzazi; Tarik Aanniz
EPI_ISL_1103569	Laboratoire central de Virologie, Centre hospitalo-universitaire Ibn Sina, Hopital des spécialités, Rabat	Laboratoire de Biotechnologie	Abdelomunim Essabbar; Amal Zouaki; Ghizlane EL Amin; Hakima Kabbaj; Lahcen Belyamani and Azeddine Ibrahimi; Mouna Ouadghiri; Myriam Seffar; Saaïd Amzazi; Tarik Aanniz
EPI_ISL_660445, EPI_ISL_660446, EPI_ISL_660447, EPI_ISL_660448, EPI_ISL_660449, EPI_ISL_660450, EPI_ISL_660451, EPI_ISL_660452, EPI_ISL_660453, EPI_ISL_660454, EPI_ISL_660455, EPI_ISL_660456, EPI_ISL_660457, EPI_ISL_660458, EPI_ISL_660459, EPI_ISL_660460, EPI_ISL_660461, EPI_ISL_660462, EPI_ISL_660463, EPI_ISL_660464, EPI_ISL_660465, EPI_ISL_660466, EPI_ISL_660467, EPI_ISL_660468, EPI_ISL_660469, EPI_ISL_660470, EPI_ISL_660471, EPI_ISL_660472, EPI_ISL_660473, EPI_ISL_660474, EPI_ISL_660475, EPI_ISL_660476, EPI_ISL_660477, EPI_ISL_660478, EPI_ISL_660479, EPI_ISL_660480, EPI_ISL_660481, EPI_ISL_660482, EPI_ISL_660483, EPI_ISL_660484, EPI_ISL_660485, EPI_ISL_660486, EPI_ISL_660487, EPI_ISL_660488, EPI_ISL_660489, EPI_ISL_660490, EPI_ISL_660491, EPI_ISL_660492, EPI_ISL_660493, EPI_ISL_660494, EPI_ISL_660495, EPI_ISL_660496, EPI_ISL_660497, EPI_ISL_660498, EPI_ISL_660499, EPI_ISL_660500, EPI_ISL_660501, EPI_ISL_660502, EPI_ISL_660503, EPI_ISL_660504, EPI_ISL_660505, EPI_ISL_660506, EPI_ISL_660507, EPI_ISL_660508, EPI_ISL_660509, EPI_ISL_660510, EPI_ISL_660511, EPI_ISL_660512, EPI_ISL_660513, EPI_ISL_660514, EPI_ISL_660515, EPI_ISL_660516, EPI_ISL_660517, EPI_ISL_660518, EPI_ISL_660519, EPI_ISL_660520, EPI_ISL_660521, EPI_ISL_660522, EPI_ISL_660523, EPI_ISL_660524, EPI_ISL_660525, EPI_ISL_660526, EPI_ISL_660527, EPI_ISL_660528			
see above	Laboratoire de Microbiologie CHU Souro Sanou	Centre Muraz	Abdoul-Salam Ouedraogo; Arsène Zongo; Essia Belarbi; Fabian Leendertz; Grit Schubert; Halidou Tinto; Lassana Sangaré; Soumeya Ouangraoua; Yacouba Sawadogo; Zekiba Tarnagda
EPI_ISL_1116464, EPI_ISL_1116467, EPI_ISL_1116468, EPI_ISL_1116469, EPI_ISL_1118675, EPI_ISL_1118884, EPI_ISL_1118923, EPI_ISL_1118926, EPI_ISL_1138747			
see above	Laboratoire de Microbiologie- CHU Habib Bourguiba – Sfax	Laboratoire des Procédés de Criblage Moléculaire et Cellulaire-Centre de Biotechnologie de Sfax	A. and Masmoudi, S.; Abdelmoulah, F.; Abid, N.; Ajili, F.; Aouni, M.; Ben Ayed, I.; Bensaid, M.; Chtourou, A.; Elargoubi, A.; Fki-berrajah, L.; Gaaloul, I.; Gargouri, S.; Hammami, A.; Kamoun, S.; Karray Hakim, H.; Kharat, N.; Mastouri, M.; Mhalla, S.; Nabli, A.; Rebai; Smeti, I.; Souissi, A.; Stambouli, N.; Turki, M.
EPI_ISL_712062, EPI_ISL_712063, EPI_ISL_712064, EPI_ISL_712065, EPI_ISL_712066, EPI_ISL_712067, EPI_ISL_712068, EPI_ISL_712069, EPI_ISL_712568			
see above	Laboratoire de Microbiologie- CHU Habib Bourguiba – Sfax adresse	Laboratoire des Procédés de Criblage Moléculaire et Cellulaire-Centre de Biotechnologie de Sfax	A. and Masmoudi, S.; Abdelmoulah, F.; Abid, N.; Ajili, F.; Aouni, M.; Ben Ayed, I.; Bensaid, M.; Chtourou, A.; Elargoubi, A.; Fki-berrajah, L.; Gaaloul, I.; Gargouri, S.; Hammami, A.; Kamoun, S.; Karray Hakim, H.; Kharat, N.; Mastouri, M.; Mhalla, S.; Nabli, A.; Rebai; Smeti, I.; Souissi, A.; Stambouli, N.; Turki, M.
EPI_ISL_451400	Laboratoire de Recherche et d'Analyse Médicale de la Gendarmerie Royale	Laboratoire de Recherche et d'Analyse Médicale de la Gendarmerie Royale	Amal SOUIRI; Sanaâ LEMRISS; Saâd EL KABBAJ
EPI_ISL_933715, EPI_ISL_933719	Laboratoire de Recherche et d'Analyses Medicales de la Gendarmerie Royale	Laboratoire de Recherche et d'Analyses Medicales de la Gendarmerie Royale	Amal Souiri; Mohammed Labioui; Momammed Labioui; Nabil Lemzaoui; S. El kabbaj; Sanaâ Lemriss; Saâd El kabbaj; elmostafa El Fahime
EPI_ISL_476024, EPI_ISL_476025, EPI_ISL_476026	Laboratoire de Recherche et d'Analyses Médicales de la Gendarmerie Royale	Laboratoire de Recherche et d'Analyses Médicales de la Gendarmerie Royale	Amal SOUIRI; Amal Souiri; Ayoub Jibjibe; Mahmoud Yartaoui; Marouane El Rhouila; Mohamed Chahmi; Mohamed Labioui; Nabil Lemzaoui; Nabil Ouaariba; Nadia Kandoussi; Omar Mestoui; Samiha Sellak; Sanaâ LEMRISS; Sanaâ Lemriss; Saâd EL KABBAJ; Saâd El Kabbaj.
EPI_ISL_1197037, EPI_ISL_1207287, EPI_ISL_1208160, EPI_ISL_1208398, EPI_ISL_1208399	Laboratoire de virologie clinique - Institut Pasteur de Tunis	1-Laboratory of Microbiology, National Reference Lab, Charles Nicolle Hospital; 2-University of Tunis ElManar, Faculty of Medicine of Tunis, LR99ES09, Tunis, Tunisia	Alia BenKahla; Anissa Chouikha; Fares Wasfi; Henda Triki; Ilhem Boutiba-Ben Boubaker.; Imen Kacem; Ines Mdini; Jalila Ben Khelil; Maher Kharrat; Manel Ben Sassi; Mariem Gdoura; Mouna Ben Sassi; Mouna Safer; Nissaf Ben Alaya; Riadh Daghfous; Riadh Gouider; Roua Ben Othman; Salma Abid; Sameh Trabelsi; Sana Ferjani; Sara Chammam; Sondes Haddad
EPI_ISL_476822, EPI_ISL_476823, EPI_ISL_476824, EPI_ISL_476825, EPI_ISL_476826, EPI_ISL_476827, EPI_ISL_476828, EPI_ISL_476829, EPI_ISL_476830, EPI_ISL_476831, EPI_ISL_476833, EPI_ISL_476834			
see above	Laboratoire des Fièvres Hémorragiques Virales du Benin	Charité-Universitätsmedizin Berlin	Anges; Drexler; Jan Felix; Moreira-Soto Andres; Sander Anna-Lena; Yadouleton
EPI_ISL_953402, EPI_ISL_953403, EPI_ISL_953404, EPI_ISL_953405, EPI_ISL_953406, EPI_ISL_953407, EPI_ISL_953408, EPI_ISL_953409, EPI_ISL_953410, EPI_ISL_953411, EPI_ISL_953412, EPI_ISL_953413, EPI_ISL_953414, EPI_ISL_953415, EPI_ISL_953416, EPI_ISL_953417, EPI_ISL_953418, EPI_ISL_953419, EPI_ISL_953420, EPI_ISL_953421, EPI_ISL_953422, EPI_ISL_953423, EPI_ISL_953424, EPI_ISL_953425			
see above	Laboratorio de Investigaciones de Baney	"Swiss Tropical and Public Health Institute"	"Carlos Cortes; Bonifacio Manguire Nlavo; Claudia Daubenberger; Diosdado Odjama Nseng Ada; Elizabeth Nyakarungu; Guillermo Garcia; Maximilian Mpina; Mitoha Ondo O Ayekaba; Philip Wonder Phiri"; Salome Hosch; Tobias Schindler
EPI_ISL_648303, EPI_ISL_648304, EPI_ISL_648305, EPI_ISL_648306, EPI_ISL_648307, EPI_ISL_648308, EPI_ISL_648309, EPI_ISL_648310, EPI_ISL_648311, EPI_ISL_648312, EPI_ISL_648313, EPI_ISL_648314, EPI_ISL_648315, EPI_ISL_648316, EPI_ISL_648317, EPI_ISL_648318, EPI_ISL_648319, EPI_ISL_648320, EPI_ISL_648321, EPI_ISL_648322, EPI_ISL_648323, EPI_ISL_648324, EPI_ISL_648325, EPI_ISL_648326, EPI_ISL_648327, EPI_ISL_648328, EPI_ISL_648329, EPI_ISL_648330, EPI_ISL_648331, EPI_ISL_648332, EPI_ISL_648333, EPI_ISL_648334, EPI_ISL_648335, EPI_ISL_648336, EPI_ISL_648337, EPI_ISL_648338, EPI_ISL_648339, EPI_ISL_648340, EPI_ISL_648341, EPI_ISL_648342, EPI_ISL_648343, EPI_ISL_648344, EPI_ISL_648345, EPI_ISL_648346, EPI_ISL_648347, EPI_ISL_648348, EPI_ISL_648349, EPI_ISL_648350, EPI_ISL_648351, EPI_ISL_648352, EPI_ISL_648353, EPI_ISL_648354, EPI_ISL_648355, EPI_ISL_648356, EPI_ISL_648357, EPI_ISL_648358, EPI_ISL_648359, EPI_ISL_648360, EPI_ISL_648361, EPI_ISL_648362, EPI_ISL_648363, EPI_ISL_648364, EPI_ISL_648365, EPI_ISL_648366, EPI_ISL_648367, EPI_ISL_648368, EPI_ISL_648369, EPI_ISL_648370, EPI_ISL_648371, EPI_ISL_648372, EPI_ISL_648373, EPI_ISL_648374, EPI_ISL_648375, EPI_ISL_648376, EPI_ISL_648377, EPI_ISL_648378, EPI_ISL_648379, EPI_ISL_649155, EPI_ISL_649156, EPI_ISL_649157, EPI_ISL_649158, EPI_ISL_649159, EPI_ISL_649160, EPI_ISL_649161, EPI_ISL_649162, EPI_ISL_649163, EPI_ISL_649164, EPI_ISL_649165, EPI_ISL_649166, EPI_ISL_649167, EPI_ISL_649168, EPI_ISL_649169, EPI_ISL_649170, EPI_ISL_649171, EPI_ISL_649172			
see above	Laboratorio de Investigaciones de Baney	University Hospital Basel, Clinical Bacteriology	Adrian Egli; Alfredo Mari; Bonifacio Manguire Nlavo; Carlos Cortes; Claudia Daubenberger; Diosdado Odjama Nseng Ada; Elizabeth Nyakarungu; Guillermo Garcia; Helena Seth-Smith; Madlen Stange; Maximilian Mpina; Mitoha Ondo O Ayekaba; Philip Wonder Phiri; Salome Hosch; Tim Roloff; Tobias Schindler
EPI_ISL_654016, EPI_ISL_654017, EPI_ISL_654018, EPI_ISL_654019, EPI_ISL_654020	Laboratory of Microbiology, National Reference Lab, Charles Nicolle Hospital; 2-University of Tunis ElManar, Faculty of Medicine of Tunis, LR99ES09, Tunis, Tunisia	1-Clinical and Experimental Pharmacology Lab, LR16SP02, National Center of Pharmacovigilance, University of Tunis El Manar, Tunis, Tunisia. 2-Neurodegenerative diseases and psychiatric troubles, LR18SP03, Razi Hospital, University of Tunis El Manar, Tunis, Tunisia. 3- Ministry of Health, National Observatory of New and Emerging Diseases, 1006, Tunis, Tunisia	Alia Ben Kahla; Gaies Emna; Ilhem Boutiba-Ben Boubaker; Imen Kacem; Imen Mkada; Jalila Ben Khelil; Maher Kharrat; Mouna Ben Sassi; Mouna Safer; Nissaf Ben Alaya; Riadh Daghfous; Riadh Gouider.; Salma Abid; Sameh Trabelsi; Sana Ferjani; Soumaya Rammeh
EPI_ISL_796782, EPI_ISL_803119, EPI_ISL_803120, EPI_ISL_803430, EPI_ISL_803817, EPI_ISL_803851, EPI_ISL_811145			
see above	Laboratory of Microbiology, National Reference Lab, Charles Nicolle Hospital; 2-University of Tunis ElManar, Faculty of Medicine of Tunis, LR99ES09, Tunis, Tunisia	Clinical and Experimental Pharmacology Lab, LR16SP02, National Center of Pharmacovigilance, University of Tunis El Manar, Tunis, Tunisia. 2-Neurodegenerative diseases and psychiatric troubles, LR18SP03, Razi Hospital, University of Tunis El Manar, Tunis, Tunisia. 3- Ministry of Health, National Observatory of New and Emerging Diseases, 1006, Tunis, Tunisia	Ahmed Fakhfakh; Alia Ben Kahla; Alia BenKahla; Asma Ferjani; Awatef El Moussi; Gaies Emna; Guedi Ali Barreh; Habiba Ben Romdhane; Hanen El Jebari; Ilhem Boutiba-Ben Boubaker; Ines Mdini; Jalila Ben Khelil; Maher Kharrat; Mouna Ben Sassi; Mouna Safer; Nissaf Ben Alaya; Riadh Daghfous; Riadh Gouider.; Salma Abid; Sameh Trabelsi; Sana Ferjani; Sarra Chamman; Souissi Amira; Zaineb Hamzaoui
EPI_ISL_700466	Ladismith (Nissenville) Clinic wc LAF	NHLS/UCT	Arash Iranzadeh; Bruna Galvao; Carolyn Williamson; Deelan Doolabh; Diana Hardie; Innocent Mudau; Kruger Marais; Lynn Tyers; Marvin Hsiao; Stephen Korsman
EPI_ISL_745124, EPI_ISL_745152	Laetitia Bam CHC	National Health Laboratory Service (NHLS), Tygerberg	Bronwyn Kleinhans; Eduan Wilkindon; Gert van Zyl; Houriiyah Tegally; Kayla Delaney; Susan Engelbrecht; Tulio de Oliveira; Wolfgang Preiser
EPI_ISL_660130, EPI_ISL_660131, EPI_ISL_660132, EPI_ISL_660133, EPI_ISL_660134, EPI_ISL_660135, EPI_ISL_660136			
see above	Lancet	National Health Laboratory Service (NHLS), Tygerberg	Bronwyn Kleinhans; Davis M-A; Draper C; Eduan Wilkindon; Gert van Zyl; Houriiyah Tegally; Hsiao M; Kayla Delaney; Siegfried N; Susan Engelbrecht; Tulio de Oliveira; Williamson C; Wolfgang Preiser
EPI_ISL_622943, EPI_ISL_622946, EPI_ISL_622947, EPI_ISL_622948, EPI_ISL_622949, EPI_ISL_622950, EPI_ISL_622951, EPI_ISL_622952, EPI_ISL_622953, EPI_ISL_622955, EPI_ISL_622957, EPI_ISL_622959, EPI_ISL_622960, EPI_ISL_622961, EPI_ISL_622962, EPI_ISL_622963, EPI_ISL_622964, EPI_ISL_622965, EPI_ISL_622966, EPI_ISL_622967, EPI_ISL_622968, EPI_ISL_622969, EPI_ISL_622978, EPI_ISL_622983, EPI_ISL_622985, EPI_ISL_622986, EPI_ISL_622988, EPI_ISL_622990, EPI_ISL_622994, EPI_ISL_623064, EPI_ISL_623068, EPI_ISL_623073			
see above	Lancet Laboratories	National Institute for Communicable Diseases of the National Health Laboratory Service	Allam M; Bhiman JN; Ismail A; Khumalo Z; Kwenda S; Mnyameni F; Mohale T; Mtshali P; Subramoney K
EPI_ISL_745130, EPI_ISL_745155, EPI_ISL_745160	Langebaan Clinic wc LBC	National Health Laboratory Service (NHLS), Tygerberg	Bronwyn Kleinhans; Eduan Wilkindon; Gert van Zyl; Houriiyah Tegally; Kayla Delaney; Susan Engelbrecht; Tulio de Oliveira; Wolfgang Preiser
EPI_ISL_1040702, EPI_ISL_1040709	Lentegeur Hospital wc LGH	NHLS/UCT	Arash Iranzadeh; Bruna Galvao; Carolyn Williamson; Deelan Doolabh; Diana Hardie; Innocent Mudau; Kruger Marais; Lynn Tyers; Marvin Hsiao; Stephen Korsman
EPI_ISL_736927, EPI_ISL_736930, EPI_ISL_736966, EPI_ISL_736967, EPI_ISL_736968, EPI_ISL_736995	MDS	KRISP, KZN Research Innovation and Sequencing Platform	ChimukangaraB; Giandhari J; Khan S; Lessells R; Mdlalose K; Pillay S; Tegally H; Wilkinson E; York D; de Oliveira T
EPI_ISL_954226, EPI_ISL_954227, EPI_ISL_954228, EPI_ISL_954229, EPI_ISL_954230, EPI_ISL_954231, EPI_ISL_954232, EPI_ISL_954233, EPI_ISL_954234, EPI_ISL_954235, EPI_ISL_954236, EPI_ISL_954237, EPI_ISL_954238, EPI_ISL_954239, EPI_ISL_954240, EPI_ISL_954241, EPI_ISL_954242, EPI_ISL_954243, EPI_ISL_954244, EPI_ISL_954245, EPI_ISL_954246, EPI_ISL_954247, EPI_ISL_954248, EPI_ISL_954249, EPI_ISL_954250, EPI_ISL_954251, EPI_ISL_954252, EPI_ISL_954253, EPI_ISL_954254, EPI_ISL_954255, EPI_ISL_954256, EPI_ISL_954257, EPI_ISL_954258, EPI_ISL_954259, EPI_ISL_954260, EPI_ISL_954261, EPI_ISL_954262, EPI_ISL_954263, EPI_ISL_954264, EPI_ISL_954266, EPI_ISL_954267, EPI_ISL_954268, EPI_ISL_954269, EPI_ISL_954270, EPI_ISL_954271, EPI_ISL_954272, EPI_ISL_954273, EPI_ISL_954274, EPI_ISL_954275, EPI_ISL_954276, EPI_ISL_954277, EPI_ISL_954278, EPI_ISL_954279, EPI_ISL_954280, EPI_ISL_954281, EPI_ISL_954282, EPI_ISL_954283, EPI_ISL_954284, EPI_ISL_954285, EPI_ISL_954286, EPI_ISL_954287, EPI_ISL_954288, EPI_ISL_954289, EPI_ISL_954290, EPI_ISL_954291, EPI_ISL_954292, EPI_ISL_954293, EPI_ISL_954294, EPI_ISL_954295, EPI_ISL_954296, EPI_ISL_954297, EPI_ISL_954298, EPI_ISL_954299, EPI_ISL_954300, EPI_ISL_955136			
see above	MRC/UVRI & LSHTM Uganda Research Unit	Where sequence data have been generated and submitted to GISAID	Dan Lule Bugembe; Isaac Sseeewanyana; Matthew Cotten; My V.T. Phan; Patrick Semanda; Pontiano Kaleebu; Susan Nabadda
EPI_ISL_455795	MRCG at LSHTM Genomics Lab	MRCG at LSHTM Genomics Lab	Sesay et al
EPI_ISL_428856	MRCG at LSHTM Genomics Lab	MRCG at LSHTM Genomics lab	Sesay et al
EPI_ISL_428857, EPI_ISL_471158, EPI_ISL_471159, EPI_ISL_471160, EPI_ISL_471161, EPI_ISL_471162, EPI_ISL_471163, EPI_ISL_471164, EPI_ISL_471165, EPI_ISL_471166, EPI_ISL_471167, EPI_ISL_471168, EPI_ISL_471169, EPI_ISL_471170, EPI_ISL_471171, EPI_ISL_560992, EPI_ISL_560993, EPI_ISL_560994, EPI_ISL_560995, EPI_ISL_560996, EPI_ISL_560997, EPI_ISL_560998, EPI_ISL_560999, EPI_ISL_561000, EPI_ISL_561001, EPI_ISL_561002, EPI_ISL_561003, EPI_ISL_561004, EPI_ISL_561005, EPI_ISL_561006, EPI_ISL_561007, EPI_ISL_561008, EPI_ISL_561009, EPI_ISL_561010, EPI_ISL_561011, EPI_ISL_561012, EPI_ISL_561013, EPI_ISL_561014, EPI_ISL_561015, EPI_ISL_561016, EPI_ISL_561017, EPI_ISL_561018, EPI_ISL_561019, EPI_ISL_561020, EPI_ISL_561021, EPI_ISL_561022, EPI_ISL_561023, EPI_ISL_561024, EPI_ISL_561025, EPI_ISL_561026, EPI_ISL_561027, EPI_ISL_561028, EPI_ISL_561029, EPI_ISL_561030, EPI_ISL_561031, EPI_ISL_561032, EPI_ISL_561033, EPI_ISL_561034, EPI_ISL_561035, EPI_ISL_561036, EPI_ISL_561037, EPI_ISL_561038, EPI_ISL_561039, EPI_ISL_561040, EPI_ISL_561041, EPI_ISL_561042, EPI_ISL_561043, EPI_ISL_561044, EPI_ISL_561045, EPI_ISL_561046, EPI_ISL_561047, EPI_ISL_561048, EPI_ISL_561049, EPI_ISL_561050, EPI_ISL_561051, EPI_ISL_561052, EPI_ISL_561053, EPI_ISL_561054, EPI_ISL_561055, EPI_ISL_561056, EPI_ISL_561057, EPI_ISL_561058, EPI_ISL_561059, EPI_ISL_561060, EPI_ISL_561061, EPI_ISL_561062, EPI_ISL_561063, EPI_ISL_561064, EPI_ISL_561065, EPI_ISL_561066, EPI_ISL_561067, EPI_ISL_561068, EPI_ISL_561069, EPI_ISL_561070, EPI_ISL_561071, EPI_ISL_561072, EPI_ISL_561073, EPI_ISL_561074, EPI_ISL_561075, EPI_ISL_561076, EPI_ISL_561077, EPI_ISL_561078, EPI_ISL_561079, EPI_ISL_561080, EPI_ISL_561081, EPI_ISL_561082, EPI_ISL_561083, EPI_ISL_561084, EPI_ISL_561085, EPI_ISL_561086, EPI_ISL_561087, EPI_ISL_561088, EPI_ISL_561089, EPI_ISL_561090, EPI_ISL_561091, EPI_ISL_561092, EPI_ISL_561093, EPI_ISL_561094, EPI_ISL_561095, EPI_ISL_561096, EPI_ISL_561097, EPI_ISL_561098, EPI_ISL_561099, EPI_ISL_561100, EPI_ISL_561101, EPI_ISL_561102, EPI_ISL_561103, EPI_ISL_561104, EPI_ISL_561105, EPI_ISL_561106, EPI_ISL_561107, EPI_ISL_561108, EPI_ISL_561109, EPI_ISL_561110, EPI_ISL_561111, EPI_ISL_561112, EPI_ISL_561113, EPI_ISL_561114, EPI_ISL_561115, EPI_ISL_561116, EPI_ISL_561117, EPI_ISL_561118, EPI_ISL_561119, EPI_ISL_561120,			

EPI_ISL_561121, EPI_ISL_561122, EPI_ISL_561123, EPI_ISL_561124, EPI_ISL_561125, EPI_ISL_561126, EPI_ISL_561127, EPI_ISL_561128, EPI_ISL_561129, EPI_ISL_561130, EPI_ISL_561131, EPI_ISL_561132, EPI_ISL_561133, EPI_ISL_561134, EPI_ISL_561135, EPI_ISL_561136, EPI_ISL_561137, EPI_ISL_561138, EPI_ISL_561139, EPI_ISL_561140, EPI_ISL_561141, EPI_ISL_561142, EPI_ISL_561143, EPI_ISL_561144, EPI_ISL_561145, EPI_ISL_561146, EPI_ISL_561147, EPI_ISL_561148, EPI_ISL_561149, EPI_ISL_561150, EPI_ISL_561151, EPI_ISL_561152, EPI_ISL_561153, EPI_ISL_561154, EPI_ISL_561155, EPI_ISL_561156, EPI_ISL_561157, EPI_ISL_561158, EPI_ISL_561159, EPI_ISL_561160, EPI_ISL_561161, EPI_ISL_561162, EPI_ISL_561163, EPI_ISL_561164, EPI_ISL_561165, EPI_ISL_561166, EPI_ISL_561167, EPI_ISL_561168, EPI_ISL_561169, EPI_ISL_561170, EPI_ISL_561171, EPI_ISL_561172, EPI_ISL_561173, EPI_ISL_561174, EPI_ISL_561175, EPI_ISL_561176, EPI_ISL_561177, EPI_ISL_561178, EPI_ISL_561179, EPI_ISL_561180, EPI_ISL_561181, EPI_ISL_561182, EPI_ISL_561183, EPI_ISL_561184, EPI_ISL_561185, EPI_ISL_561186, EPI_ISL_561187, EPI_ISL_561188, EPI_ISL_561189, EPI_ISL_561190, EPI_ISL_561191, EPI_ISL_561192, EPI_ISL_561193, EPI_ISL_561194, EPI_ISL_561195, EPI_ISL_561196, EPI_ISL_561197, EPI_ISL_561198, EPI_ISL_561199, EPI_ISL_561200, EPI_ISL_561201, EPI_ISL_561202, EPI_ISL_561203, EPI_ISL_561204, EPI_ISL_561207, EPI_ISL_561209, EPI_ISL_561210, EPI_ISL_561211, EPI_ISL_561212, EPI_ISL_561213, EPI_ISL_561214, EPI_ISL_561215, EPI_ISL_561216, EPI_ISL_561217, EPI_ISL_561218, EPI_ISL_561219, EPI_ISL_561220, EPI_ISL_561221, EPI_ISL_561222, EPI_ISL_561223, EPI_ISL_561224, EPI_ISL_561225, EPI_ISL_561226, EPI_ISL_561227, EPI_ISL_561228, EPI_ISL_561229, EPI_ISL_561230, EPI_ISL_561234, EPI_ISL_561235, EPI_ISL_561236, EPI_ISL_561237, EPI_ISL_561238, EPI_ISL_561240, EPI_ISL_561241, EPI_ISL_561249, EPI_ISL_561252, EPI_ISL_561253, EPI_ISL_561255, EPI_ISL_561256, EPI_ISL_561258, EPI_ISL_561259, EPI_ISL_561260, EPI_ISL_561261, EPI_ISL_561262, EPI_ISL_561263, EPI_ISL_561264, EPI_ISL_561265, EPI_ISL_561266, EPI_ISL_561267, EPI_ISL_561268, EPI_ISL_561269, EPI_ISL_561272, EPI_ISL_561274, EPI_ISL_561276, EPI_ISL_561281, EPI_ISL_561283, EPI_ISL_561285, EPI_ISL_561288, EPI_ISL_561289, EPI_ISL_561290, EPI_ISL_561291, EPI_ISL_561292, EPI_ISL_561293, EPI_ISL_561294, EPI_ISL_561295, EPI_ISL_561297, EPI_ISL_561302, EPI_ISL_561304, EPI_ISL_561309, EPI_ISL_561310, EPI_ISL_561312, EPI_ISL_561315, EPI_ISL_561316, EPI_ISL_561319, EPI_ISL_561322, EPI_ISL_561325, EPI_ISL_561328, EPI_ISL_810971, EPI_ISL_810972, EPI_ISL_810973, EPI_ISL_810974, EPI_ISL_810975, EPI_ISL_810976, EPI_ISL_810977, EPI_ISL_810978, EPI_ISL_810979, EPI_ISL_810980, EPI_ISL_810981, EPI_ISL_810982, EPI_ISL_810983, EPI_ISL_810984, EPI_ISL_810985, EPI_ISL_810986, EPI_ISL_810987, EPI_ISL_810988, EPI_ISL_810989, EPI_ISL_810990, EPI_ISL_810991, EPI_ISL_810992, EPI_ISL_810993, EPI_ISL_810994, EPI_ISL_810995, EPI_ISL_810996, EPI_ISL_810997, EPI_ISL_810998, EPI_ISL_810999, EPI_ISL_811000, EPI_ISL_811001, EPI_ISL_811002, EPI_ISL_811003, EPI_ISL_811004, EPI_ISL_811005, EPI_ISL_811006, EPI_ISL_811007, EPI_ISL_811008, EPI_ISL_811009, EPI_ISL_811010, EPI_ISL_811011, EPI_ISL_811012, EPI_ISL_811013, EPI_ISL_811014, EPI_ISL_811015, EPI_ISL_811016, EPI_ISL_811017, EPI_ISL_811018, EPI_ISL_811019, EPI_ISL_811020, EPI_ISL_811021, EPI_ISL_811022, EPI_ISL_811023, EPI_ISL_811024, EPI_ISL_811025, EPI_ISL_811026, EPI_ISL_811027, EPI_ISL_811028, EPI_ISL_811029, EPI_ISL_811030, EPI_ISL_811031, EPI_ISL_811032, EPI_ISL_811033, EPI_ISL_811034, EPI_ISL_811035, EPI_ISL_811036, EPI_ISL_811037, EPI_ISL_914970, EPI_ISL_915192, EPI_ISL_915421, EPI_ISL_1216070, EPI_ISL_1216072, EPI_ISL_1216074, EPI_ISL_1216076, EPI_ISL_1216077, EPI_ISL_1216079, EPI_ISL_1216081, EPI_ISL_1216083, EPI_ISL_1216085, EPI_ISL_1216087, EPI_ISL_1216088, EPI_ISL_1216090, EPI_ISL_1216092, EPI_ISL_1216093, EPI_ISL_1216095, EPI_ISL_1216097, EPI_ISL_1216099, EPI_ISL_1216100, EPI_ISL_1216104, EPI_ISL_1216106, EPI_ISL_1216107, EPI_ISL_1216109, EPI_ISL_1216110, EPI_ISL_1216112, EPI_ISL_1216114, EPI_ISL_1216116, EPI_ISL_1216118, EPI_ISL_1216119, EPI_ISL_1216121, EPI_ISL_1216123, EPI_ISL_1216124, EPI_ISL_1216127, EPI_ISL_1216128, EPI_ISL_1216130, EPI_ISL_1216132, EPI_ISL_1216133, EPI_ISL_1234525, EPI_ISL_1234526, EPI_ISL_1234527, EPI_ISL_1234528, EPI_ISL_1234529, EPI_ISL_1234530, EPI_ISL_1234531, EPI_ISL_1234532, EPI_ISL_1234533, EPI_ISL_1234534, EPI_ISL_1234535, EPI_ISL_1234536			
see above	MRCG at LSHTM Genomics lab	MRCG at LSHTM Genomics lab	Abdoulie Kanteh; Abdul Karim sesay; Bakary Sanyang; Jarra Manneh; Mariama Kujabi; Sesay et al
EPI_ISL_455794	MRCG at LSHTM Genomics lab	MRCG at LSHTM Genomics laboratory	Sesay et al
EPI_ISL_428855	MRCG at LSHTM Geomics lab	MRCG at LSHTM Genomics lab	Sesay et al
EPI_ISL_640074	Mamre CDC wc MRC	NHLS/UCT	Arash Iranzadeh; Bruna Galvao; Carolyn Williamson; Deelan Doolabh; Diana Hardie; Innocent Mudau; Kruger Marais; Lynn Tyers; Marvin Hsiao; Stephen Korsman
EPI_ISL_1196017, EPI_ISL_1196018	Manzini RRT	National Institute for Communicable Diseases of the National Health Laboratory Service	Amoako DG; Bhiman JN; Ismail A; Mahlangu B; Maphalala GP; Mohale T; Ntuli N; Scheepers C
EPI_ISL_700544	Masincedane Clinic wc MAS	NHLS/UCT	Arash Iranzadeh; Bruna Galvao; Carolyn Williamson; Deelan Doolabh; Diana Hardie; Innocent Mudau; Kruger Marais; Lynn Tyers; Marvin Hsiao; Stephen Korsman
EPI_ISL_1196000	Mbabane Clinic	National Institute for Communicable Diseases of the National Health Laboratory Service	Amoako DG; Bhiman JN; Ismail A; Mahlangu B; Maphalala GP; Mohale T; Ntuli N; Scheepers C
EPI_ISL_1196001, EPI_ISL_1196007	Mbabane Gov Hospital	National Institute for Communicable Diseases of the National Health Laboratory Service	Amoako DG; Bhiman JN; Ismail A; Mahlangu B; Maphalala GP; Mohale T; Ntuli N; Scheepers C
EPI_ISL_1196004, EPI_ISL_1196015, EPI_ISL_1196016	Mbabane PHU	National Institute for Communicable Diseases of the National Health Laboratory Service	Amoako DG; Bhiman JN; Ismail A; Mahlangu B; Maphalala GP; Mohale T; Ntuli N; Scheepers C
EPI_ISL_475745, EPI_ISL_475746, EPI_ISL_475747, EPI_ISL_475748, EPI_ISL_475749, EPI_ISL_475750, EPI_ISL_475751, EPI_ISL_475752, EPI_ISL_475753, EPI_ISL_482759, EPI_ISL_482760, EPI_ISL_482761, EPI_ISL_482762, EPI_ISL_482763, EPI_ISL_482764, EPI_ISL_482765, EPI_ISL_482766, EPI_ISL_482767, EPI_ISL_482768, EPI_ISL_482769, EPI_ISL_482770, EPI_ISL_482771, EPI_ISL_482772, EPI_ISL_482773, EPI_ISL_482774, EPI_ISL_482775, EPI_ISL_483035, EPI_ISL_483036, EPI_ISL_483037, EPI_ISL_483038, EPI_ISL_1097025, EPI_ISL_1097026, EPI_ISL_1097027, EPI_ISL_1097028, EPI_ISL_1097029, EPI_ISL_1098839, EPI_ISL_1109484, EPI_ISL_1109485, EPI_ISL_1109486, EPI_ISL_1109624, EPI_ISL_1109625, EPI_ISL_1109626, EPI_ISL_1109627, EPI_ISL_1109628, EPI_ISL_1109629, EPI_ISL_1109630, EPI_ISL_1141525, EPI_ISL_1165078, EPI_ISL_1165079, EPI_ISL_1165080, EPI_ISL_1165081, EPI_ISL_1165082, EPI_ISL_1165083, EPI_ISL_1165084, EPI_ISL_1165085, EPI_ISL_1165086, EPI_ISL_1165087, EPI_ISL_1167186, EPI_ISL_1167187, EPI_ISL_1167188, EPI_ISL_1167189, EPI_ISL_1167190, EPI_ISL_1167191, EPI_ISL_1167192, EPI_ISL_1167193, EPI_ISL_1167194, EPI_ISL_1167195, EPI_ISL_1167196, EPI_ISL_1167197			
see above	Medical Ain Shams Research Institute (MASRI), Ain Shams University	Medical Ain Shams Research Institute (MASRI), Ain Shams University	Ahmad Moustafa; Ashraf Omar; Aya Mohamed; Fatma Ebied; Ghada Ismael; Hagar Elshora; Hala Hafez; Hesham Elghazaly; Hoda Abd Elsatar; Hoda Ezz Elarab; Iman Foda; Mahmoud Elmeitini; Mahmoud Elmeteini; Manal Hamdy Elsaid; Mohamed Elhadidi; Osama Mansour; Osama Mansour.; Reham Kassab; Reham Mamdouh; Samia Abdo; Samia Abdou Girgis; Sara Elnakeep; Sara Hassan Agwa; Shaimaa Moustafa; Shimaa Moustafa; Shimaa moustafa
EPI_ISL_495535, EPI_ISL_495536, EPI_ISL_495537, EPI_ISL_495538, EPI_ISL_495539, EPI_ISL_495540, EPI_ISL_495541, EPI_ISL_495542, EPI_ISL_515800, EPI_ISL_515801, EPI_ISL_515802, EPI_ISL_515803, EPI_ISL_515804, EPI_ISL_515805, EPI_ISL_515806, EPI_ISL_515807, EPI_ISL_515808, EPI_ISL_515809, EPI_ISL_515810, EPI_ISL_515811, EPI_ISL_515812, EPI_ISL_515813, EPI_ISL_515814, EPI_ISL_515815, EPI_ISL_515816, EPI_ISL_515817, EPI_ISL_515818, EPI_ISL_515819, EPI_ISL_515820, EPI_ISL_515821, EPI_ISL_515822, EPI_ISL_515823, EPI_ISL_515824, EPI_ISL_515825, EPI_ISL_515826, EPI_ISL_515827, EPI_ISL_515828, EPI_ISL_515829, EPI_ISL_515830, EPI_ISL_515831, EPI_ISL_515832, EPI_ISL_515833, EPI_ISL_515834, EPI_ISL_515835, EPI_ISL_515836, EPI_ISL_515837, EPI_ISL_515838, EPI_ISL_515839, EPI_ISL_515840, EPI_ISL_515841, EPI_ISL_515842, EPI_ISL_515843, EPI_ISL_515844, EPI_ISL_515845, EPI_ISL_515846, EPI_ISL_515847, EPI_ISL_515848, EPI_ISL_515849, EPI_ISL_515850, EPI_ISL_515851, EPI_ISL_515852, EPI_ISL_515853, EPI_ISL_515854, EPI_ISL_515855, EPI_ISL_515856, EPI_ISL_515857, EPI_ISL_515858, EPI_ISL_515859, EPI_ISL_515860, EPI_ISL_515861, EPI_ISL_515862, EPI_ISL_515863, EPI_ISL_515864, EPI_ISL_515865, EPI_ISL_515866, EPI_ISL_515867, EPI_ISL_515868, EPI_ISL_515869, EPI_ISL_515870, EPI_ISL_515871, EPI_ISL_515872, EPI_ISL_515873, EPI_ISL_515874, EPI_ISL_515875, EPI_ISL_515876, EPI_ISL_515877, EPI_ISL_515878, EPI_ISL_515879, EPI_ISL_515880, EPI_ISL_515881, EPI_ISL_515882, EPI_ISL_515883, EPI_ISL_515884, EPI_ISL_515885, EPI_ISL_515886, EPI_ISL_515887, EPI_ISL_515888, EPI_ISL_515889, EPI_ISL_515890, EPI_ISL_515891, EPI_ISL_515892, EPI_ISL_515893			
see above	Medical Disagnostics Services (MDS)	KRISP, KZN Research Innovation and Sequencing Platform	Chimukangara B; ChimukangaraB; Giandhari J; Khan S; Lessells R; Mdlalose K; Pillay S; Tegally H; Wilkinson E; York D; de Oliveira T
EPI_ISL_640080	Mfuleni CDC wc MFU	NHLS/UCT	Arash Iranzadeh; Bruna Galvao; Carolyn Williamson; Deelan Doolabh; Diana Hardie; Innocent Mudau; Kruger Marais; Lynn Tyers; Marvin Hsiao; Stephen Korsman
EPI_ISL_640025	Mitchells Plain CHC wc MHC	NHLS/UCT	Arash Iranzadeh; Bruna Galvao; Carolyn Williamson; Deelan Doolabh; Diana Hardie; Innocent Mudau; Kruger Marais; Lynn Tyers; Marvin Hsiao; Stephen Korsman
EPI_ISL_640020, EPI_ISL_640028, EPI_ISL_640065, EPI_ISL_700489, EPI_ISL_700572, EPI_ISL_700597, EPI_ISL_1040766, EPI_ISL_1040794			
see above	Mitchells Plain Hospital wc MPH	NHLS/UCT	Arash Iranzadeh; Bruna Galvao; Carolyn Williamson; Deelan Doolabh; Diana Hardie; Innocent Mudau; Kruger Marais; Lynn Tyers; Marvin Hsiao; Stephen Korsman
EPI_ISL_960095, EPI_ISL_960097, EPI_ISL_960098, EPI_ISL_960100, EPI_ISL_960121, EPI_ISL_960125, EPI_ISL_960128, EPI_ISL_960129, EPI_ISL_960137, EPI_ISL_960148			
see above	Mitchells Plain Hospital wc MPH	National Health Laboratory Service/UCT	Arash Iranzadeh; Bruna Galvao; Carolyn Williamson; Deelan Doolabh; Diana Hardie; Innocent Mudau; Kruger Marais; Lynn Tyers; Marvin Hsiao; Stephen Korsman
EPI_ISL_660259, EPI_ISL_660260, EPI_ISL_660261, EPI_ISL_660262, EPI_ISL_660263	Molecualr Diagnostic Services (MDS)	KRISP, KZN Research Innovation and Sequencing Platform	Giandhari J; Khan S; Lessells R; Mdlalose K; Pillay S; Tegally H; Wilkinson E; York D; de Oliveira T
EPI_ISL_421573, EPI_ISL_421574, EPI_ISL_421575, EPI_ISL_421576	Molecular Diagnostic Services	KRISP, KZN Research Innovation and Sequencing Platform	Chimukangara B; Deforche K; Giandhari J; Lessells R; Ngcapu S; Pillay S; Samsunder N; Tegally H; Wilkinson E; de Oliveira T
EPI_ISL_421572	Molecular Diagnostic Services and FLowpath	KRISP, KZN Research Innovation and Sequencing Platform	Chimukangara B; Deforche K; Giandhari J; Lessells R; Ngcapu S; Pillay S; Samsunder N; Tegally H; Wilkinson E; de Oliveira T
EPI_ISL_467431, EPI_ISL_467444, EPI_ISL_467445, EPI_ISL_467446, EPI_ISL_467447, EPI_ISL_467448, EPI_ISL_467475, EPI_ISL_467476, EPI_ISL_467477, EPI_ISL_467478, EPI_ISL_467479, EPI_ISL_467480, EPI_ISL_467481, EPI_ISL_467482, EPI_ISL_467483, EPI_ISL_467484, EPI_ISL_467485, EPI_ISL_467486, EPI_ISL_467487, EPI_ISL_467488, EPI_ISL_467489, EPI_ISL_467490, EPI_ISL_467491, EPI_ISL_467494, EPI_ISL_467495, EPI_ISL_467496, EPI_ISL_467497, EPI_ISL_467498, EPI_ISL_467499, EPI_ISL_467500, EPI_ISL_467501, EPI_ISL_467502, EPI_ISL_467503, EPI_ISL_467504, EPI_ISL_467505, EPI_ISL_467506, EPI_ISL_482702, EPI_ISL_482703, EPI_ISL_482704, EPI_ISL_482705, EPI_ISL_482706, EPI_ISL_482707, EPI_ISL_482708, EPI_ISL_482709, EPI_ISL_482714, EPI_ISL_482715, EPI_ISL_482716, EPI_ISL_482717, EPI_ISL_482718, EPI_ISL_482719, EPI_ISL_482720, EPI_ISL_482721, EPI_ISL_482722, EPI_ISL_482723, EPI_ISL_482851, EPI_ISL_482852, EPI_ISL_482853, EPI_ISL_482854, EPI_ISL_482855, EPI_ISL_482856, EPI_ISL_482857, EPI_ISL_482858, EPI_ISL_482859, EPI_ISL_482860, EPI_ISL_482861, EPI_ISL_482862, EPI_ISL_482863, EPI_ISL_482864, EPI_ISL_482865, EPI_ISL_482866, EPI_ISL_482867, EPI_ISL_482868, EPI_ISL_482869, EPI_ISL_482870, EPI_ISL_482871, EPI_ISL_482872, EPI_ISL_487329, EPI_ISL_487330, EPI_ISL_487331, EPI_ISL_487332, EPI_ISL_487333, EPI_ISL_487334, EPI_ISL_487335, EPI_ISL_487336, EPI_ISL_487337, EPI_ISL_487338, EPI_ISL_487339, EPI_ISL_487340, EPI_ISL_487341, EPI_ISL_1250507, EPI_ISL_1250509, EPI_ISL_1250512, EPI_ISL_1250514, EPI_ISL_1250515, EPI_ISL_1250517, EPI_ISL_1250518, EPI_ISL_1250520, EPI_ISL_1250522, EPI_ISL_1250523, EPI_ISL_1250525, EPI_ISL_1250526, EPI_ISL_1250528, EPI_ISL_1250529, EPI_ISL_1250531, EPI_ISL_1250533, EPI_ISL_1250535, EPI_ISL_1250537, EPI_ISL_1250538, EPI_ISL_1250540, EPI_ISL_1250542, EPI_ISL_1250543, EPI_ISL_1250545, EPI_ISL_1250547, EPI_ISL_1250548, EPI_ISL_1250550, EPI_ISL_1250551, EPI_ISL_1250552			
see above	Molecular Diagnostics Services (MDS)	KRISP, KZN Research Innovation and Sequencing Platform	Chimukangara B; Emmanuel SJ; Giandhari J; Khan S; Lessells R; Maslo C; Mdlalose K; Pillay S; Sitharam L; Tegally H; Wilkinson E; York D; de Oliveira T
EPI_ISL_614347, EPI_ISL_614348, EPI_ISL_614349, EPI_ISL_614350, EPI_ISL_614351, EPI_ISL_614352, EPI_ISL_614353, EPI_ISL_614354, EPI_ISL_614355, EPI_ISL_614356, EPI_ISL_614357, EPI_ISL_614358, EPI_ISL_614359, EPI_ISL_614360, EPI_ISL_614361, EPI_ISL_614362, EPI_ISL_614363, EPI_ISL_614364, EPI_ISL_614365, EPI_ISL_614366, EPI_ISL_614367, EPI_ISL_614368, EPI_ISL_614369, EPI_ISL_614370, EPI_ISL_614371, EPI_ISL_614372, EPI_ISL_614373, EPI_ISL_614374, EPI_ISL_614375, EPI_ISL_614376, EPI_ISL_614377, EPI_ISL_614378, EPI_ISL_614379, EPI_ISL_614380, EPI_ISL_614381, EPI_ISL_614382, EPI_ISL_614383, EPI_ISL_614384, EPI_ISL_614385, EPI_ISL_614386, EPI_ISL_614387, EPI_ISL_614388, EPI_ISL_614389, EPI_ISL_614390, EPI_ISL_614391, EPI_ISL_614392, EPI_ISL_614393, EPI_ISL_614394, EPI_ISL_614395, EPI_ISL_681829, EPI_ISL_681830, EPI_ISL_681831, EPI_ISL_681832, EPI_ISL_681833, EPI_ISL_681834, EPI_ISL_681835, EPI_ISL_681836, EPI_ISL_681837, EPI_ISL_681838, EPI_ISL_681839, EPI_ISL_681840, EPI_ISL_681841, EPI_ISL_681842, EPI_ISL_682058, EPI_ISL_682059			
see above	Molecular diagnostic unit for viral haemorrhagic fevers and emerging viruses, Bouaké CHU Laboratory	Project group Epidemology of Highly Pathogenic Microorganisms, Robert Koch-Institute	Adjaratou Traoré; Bamba Fatoumata Touré; Chantal Akoua-Koffi; Coulibaly Mbegnan; Diané Bamourou; Essia Belarbi; Etilé Anoh; Fabian Leendertz; Grit Schubert; Kra Ouffoué; Monemo Pacome; Safiatou Karidioula; Soundélé Maïté
EPI_ISL_640039	Mossel Bay Hospital wc MBY	NHLS/UCT	Arash Iranzadeh; Bruna Galvao; Carolyn Williamson; Deelan Doolabh; Diana Hardie; Innocent Mudau; Kruger Marais; Lynn Tyers; Marvin Hsiao; Stephen Korsman
EPI_ISL_700479, EPI_ISL_1040701, EPI_ISL_1040712	Mowbray Maternity Hospital wc MMH	NHLS/UCT	Arash Iranzadeh; Bruna Galvao; Carolyn Williamson; Deelan Doolabh; Diana Hardie; Innocent Mudau; Kruger Marais; Lynn Tyers; Marvin Hsiao; Stephen Korsman
EPI_ISL_960104	Mowbray Maternity Hospital wc MMH	National Health Laboratory Service/UCT	Arash Iranzadeh; Bruna Galvao; Carolyn Williamson; Deelan Doolabh; Diana Hardie; Innocent Mudau; Kruger Marais; Lynn Tyers; Marvin Hsiao; Stephen Korsman
EPI_ISL_682323, EPI_ISL_682324, EPI_ISL_682325, EPI_ISL_682326, EPI_ISL_682327, EPI_ISL_682328, EPI_ISL_682329, EPI_ISL_682330, EPI_ISL_682331, EPI_ISL_682332, EPI_ISL_682333, EPI_ISL_682334, EPI_ISL_682335, EPI_ISL_682336, EPI_ISL_682337, EPI_ISL_682338, EPI_ISL_682339, EPI_ISL_682340, EPI_ISL_682341, EPI_ISL_682342, EPI_ISL_682343, EPI_ISL_682344, EPI_ISL_682345, EPI_ISL_682346, EPI_ISL_682347, EPI_ISL_682348, EPI_ISL_682349, EPI_ISL_682350, EPI_ISL_682351, EPI_ISL_682352, EPI_ISL_684047, EPI_ISL_912456, EPI_ISL_912457, EPI_ISL_912458, EPI_ISL_912459, EPI_ISL_912460, EPI_ISL_912461, EPI_ISL_912462, EPI_ISL_912463, EPI_ISL_912464, EPI_ISL_912465, EPI_ISL_912466, EPI_ISL_912467, EPI_ISL_912468, EPI_ISL_912469, EPI_ISL_912470, EPI_ISL_912471, EPI_ISL_912472, EPI_ISL_912473, EPI_ISL_912474, EPI_ISL_912475, EPI_ISL_912476, EPI_ISL_912477, EPI_ISL_912478, EPI_ISL_912479, EPI_ISL_912480, EPI_ISL_912481, EPI_ISL_912482, EPI_ISL_912483, EPI_ISL_912484, EPI_ISL_912485, EPI_ISL_912486, EPI_ISL_912487, EPI_ISL_912488, EPI_ISL_912489, EPI_ISL_912490, EPI_ISL_912491, EPI_ISL_912492, EPI_ISL_912493, EPI_ISL_912494, EPI_ISL_912495, EPI_ISL_912496, EPI_ISL_912497, EPI_ISL_912498, EPI_ISL_912499, EPI_ISL_912500, EPI_ISL_912501, EPI_ISL_912502, EPI_ISL_912503, EPI_ISL_912504, EPI_ISL_912505, EPI_ISL_912506, EPI_ISL_912507, EPI_ISL_912508, EPI_ISL_912509, EPI_ISL_912510, EPI_ISL_912511, EPI_ISL_912512, EPI_ISL_912513, EPI_ISL_912514, EPI_ISL_912515, EPI_ISL_912516, EPI_ISL_912517, EPI_ISL_912518, EPI_ISL_912519, EPI_ISL_912520, EPI_ISL_912521, EPI_ISL_912522, EPI_ISL_912523, EPI_ISL_912524, EPI_ISL_912525, EPI_ISL_912526, EPI_ISL_912527, EPI_ISL_912528, EPI_ISL_912529, EPI_ISL_912530, EPI_ISL_912531, EPI_ISL_912532, EPI_ISL_912533, EPI_ISL_912534, EPI_ISL_912535, EPI_ISL_912536, EPI_ISL_912537, EPI_ISL_912538, EPI_ISL_940147, EPI_ISL_940148, EPI_ISL_940149, EPI_ISL_940150, EPI_ISL_940151, EPI_ISL_940152, EPI_ISL_940153, EPI_ISL_940154, EPI_ISL_940155, EPI_ISL_940156, EPI_ISL_949618, EPI_ISL_949620, EPI_ISL_949622, EPI_ISL_949624, EPI_ISL_949626, EPI_ISL_949628, EPI_ISL_949630, EPI_ISL_949632, EPI_ISL_949634, EPI_ISL_1007984, EPI_ISL_1007985, EPI_ISL_1007986, EPI_ISL_1007987, EPI_ISL_1007988, EPI_ISL_1007989, EPI_ISL_1007990, EPI_ISL_1007991			
see above	NHLS Universitas Academic	UFS Virology	D Goedhals; Emmanuel Ogunbayo; MM Nyaga; MT Mogotsi; P Nthiga; PA Bester; T de Oliveira
EPI_ISL_467436, EPI_ISL_467437, EPI_ISL_467438, EPI_ISL_467439, EPI_ISL_467440, EPI_ISL_467441, EPI_ISL_467442, EPI_ISL_467443, EPI_ISL_467492, EPI_ISL_467493, EPI_ISL_467507, EPI_ISL_467508, EPI_ISL_467509, EPI_ISL_467510, EPI_ISL_467511, EPI_ISL_467512, EPI_ISL_467513, EPI_ISL_467514, EPI_ISL_467515, EPI_ISL_467517, EPI_ISL_467518, EPI_ISL_467519, EPI_ISL_467520, EPI_ISL_467521, EPI_ISL_467522, EPI_ISL_467523, EPI_ISL_467524, EPI_ISL_482710, EPI_ISL_482711, EPI_ISL_482712, EPI_ISL_482713, EPI_ISL_482724, EPI_ISL_482725, EPI_ISL_482726, EPI_ISL_482727, EPI_ISL_482728, EPI_ISL_482729, EPI_ISL_482730, EPI_ISL_482731, EPI_ISL_482848, EPI_ISL_482849, EPI_ISL_482850, EPI_ISL_487277, EPI_ISL_487278, EPI_ISL_487279, EPI_ISL_487280, EPI_ISL_487281, EPI_ISL_487282, EPI_ISL_487283, EPI_ISL_487284, EPI_ISL_487285, EPI_ISL_487286, EPI_ISL_487287, EPI_ISL_487288, EPI_ISL_487289, EPI_ISL_487290, EPI_ISL_487291, EPI_ISL_487292, EPI_ISL_487293, EPI_ISL_487294, EPI_ISL_487295, EPI_ISL_487296, EPI_ISL_487297, EPI_ISL_487298, EPI_ISL_487299, EPI_ISL_487300, EPI_ISL_487301, EPI_ISL_487302, EPI_ISL_487303, EPI_ISL_487304, EPI_ISL_487305, EPI_ISL_487306, EPI_ISL_487307, EPI_ISL_487308, EPI_ISL_487309, EPI_ISL_487310, EPI_ISL_487311, EPI_ISL_487312, EPI_ISL_487313, EPI_ISL_487314, EPI_ISL_487315, EPI_ISL_487316, EPI_ISL_487317, EPI_ISL_487318, EPI_ISL_487319, EPI_ISL_487320, EPI_ISL_487321, EPI_ISL_487322, EPI_ISL_487323, EPI_ISL_487324, EPI_ISL_487325, EPI_ISL_487326, EPI_ISL_487327, EPI_ISL_487328, EPI_ISL_487348, EPI_ISL_495516, EPI_ISL_495517, EPI_ISL_495518, EPI_ISL_495519, EPI_ISL_495520, EPI_ISL_495521, EPI_ISL_495522, EPI_ISL_495523, EPI_ISL_495524, EPI_ISL_495525, EPI_ISL_495526, EPI_ISL_495527, EPI_ISL_495528, EPI_ISL_495529, EPI_ISL_495530, EPI_ISL_495531, EPI_ISL_495532, EPI_ISL_495533, EPI_ISL_495534, EPI_ISL_495543, EPI_ISL_495544, EPI_ISL_495545, EPI_ISL_495546, EPI_ISL_495547, EPI_ISL_495548, EPI_ISL_495549, EPI_ISL_495550, EPI_ISL_495551, EPI_ISL_495552, EPI_ISL_495553, EPI_ISL_495554, EPI_ISL_495555, EPI_ISL_495556, EPI_ISL_495557, EPI_ISL_495558, EPI_ISL_495559, EPI_ISL_495560, EPI_ISL_495561, EPI_ISL_495562, EPI_ISL_498054, EPI_ISL_498055, EPI_ISL_498056, EPI_ISL_498057, EPI_ISL_498058, EPI_ISL_498059, EPI_ISL_498060, EPI_ISL_498061, EPI_ISL_498062, EPI_ISL_498063, EPI_ISL_498064, EPI_ISL_498065, EPI_ISL_498066, EPI_ISL_498067, EPI_ISL_498068, EPI_ISL_498069, EPI_ISL_498070, EPI_ISL_498071, EPI_ISL_498072, EPI_ISL_498073, EPI_ISL_498074, EPI_ISL_498075, EPI_ISL_498076, EPI_ISL_498077, EPI_ISL_498078, EPI_ISL_498079, EPI_ISL_498080, EPI_ISL_498081, EPI_ISL_498082, EPI_ISL_498083, EPI_ISL_498084, EPI_ISL_498085, EPI_ISL_498086, EPI_ISL_498087, EPI_ISL_498088, EPI_ISL_498089, EPI_ISL_498090, EPI_ISL_498091, EPI_ISL_498092, EPI_ISL_498093, EPI_ISL_498094, EPI_ISL_498095, EPI_ISL_498096, EPI_ISL_498097, EPI_ISL_498098, EPI_ISL_498099, EPI_ISL_498100, EPI_ISL_498101, EPI_ISL_498102, EPI_ISL_498103, EPI_ISL_498104, EPI_ISL_498105, EPI_ISL_498106, EPI_ISL_498107, EPI_ISL_498108, EPI_ISL_498109, EPI_ISL_498110, EPI_ISL_498111, EPI_ISL_498112, EPI_ISL_498113, EPI_ISL_498114, EPI_ISL_498115, EPI_ISL_498116, EPI_ISL_498117, EPI_ISL_498118, EPI_ISL_498119, EPI_ISL_498120, EPI_ISL_498121, EPI_ISL_498122, EPI_ISL_498123, EPI_ISL_498124, EPI_ISL_498125, EPI_ISL_498126, EPI_ISL_509223, EPI_ISL_509224, EPI_ISL_509225, EPI_ISL_509226, EPI_ISL_509227, EPI_ISL_509228, EPI_ISL_509229, EPI_ISL_509230, EPI_ISL_509231, EPI_ISL_509232, EPI_ISL_509233, EPI_ISL_509234, EPI_ISL_509235, EPI_ISL_509236, EPI_ISL_509237, EPI_ISL_509238, EPI_ISL_509239, EPI_ISL_509240, EPI_ISL_509241, EPI_ISL_509242, EPI_ISL_509243, EPI_ISL_509244, EPI_ISL_509245, EPI_ISL_509246, EPI_ISL_509247, EPI_ISL_509248, EPI_ISL_509249, EPI_ISL_509250, EPI_ISL_509251, EPI_ISL_509252, EPI_ISL_509253, EPI_ISL_509254, EPI_ISL_509255, EPI_ISL_509256, EPI_ISL_509257, EPI_ISL_509258, EPI_ISL_509259, EPI_ISL_509260, EPI_ISL_509261, EPI_ISL_509262, EPI_ISL_509263, EPI_ISL_509264, EPI_ISL_509265, EPI_ISL_509266, EPI_ISL_509267, EPI_ISL_509268, EPI_ISL_509269, EPI_ISL_509270, EPI_ISL_509271, EPI_ISL_509272, EPI_ISL_509273, EPI_ISL_509274, EPI_ISL_509275, EPI_ISL_509276, EPI_ISL_509277, EPI_ISL_509278, EPI_ISL_509279, EPI_ISL_509280, EPI_ISL_509281, EPI_ISL_509282, EPI_ISL_509283, EPI_ISL_509284, EPI_ISL_509285, EPI_ISL_509286, EPI_ISL_509287, EPI_ISL_509288, EPI_ISL_509289, EPI_ISL_509290, EPI_ISL_509291, EPI_ISL_509292, EPI_ISL_509293, EPI_ISL_509294, EPI_ISL_509295, EPI_ISL_509296, EPI_ISL_509297, EPI_ISL_509298, EPI_ISL_509299, EPI_ISL_509300, EPI_ISL_509301, EPI_ISL_509302, EPI_ISL_509303, EPI_ISL_509304, EPI_ISL_509305, EPI_ISL_509306, EPI_ISL_509307, EPI_ISL_509308, EPI_ISL_509309, EPI_ISL_509310, EPI_ISL_509311, EPI_ISL_509312, EPI_ISL_509313, EPI_ISL_509314, EPI_ISL_509315, EPI_ISL_509316, EPI_ISL_509317, EPI_ISL_509318, EPI_ISL_509319, EPI_ISL_509320, EPI_ISL_509321, EPI_ISL_509322, EPI_ISL_509323, EPI_ISL_509324, EPI_ISL_509325, EPI_ISL_509326, EPI_ISL_509327, EPI_ISL_509328, EPI_ISL_509329, EPI_ISL_509330, EPI_ISL_509331, EPI_ISL_509332, EPI_ISL_509333, EPI_ISL_509334, EPI_ISL_509335, EPI_ISL_509336, EPI_ISL_509337, EPI_ISL_509338, EPI_ISL_509339, EPI_ISL_509340, EPI_ISL_509341, EPI_ISL_509342, EPI_ISL_509343, EPI_ISL_509344, EPI_ISL_509345, EPI_ISL_509346, EPI_ISL_509347, EPI_ISL_509348, EPI_ISL_509349, EPI_ISL_509350, EPI_ISL_509351, EPI_ISL_509352, EPI_ISL_509353, EPI_ISL_509354, EPI_ISL_509355, EPI_ISL_509356, EPI_ISL_509357, EPI_ISL_509358, EPI_ISL_509359, EPI_ISL_509360, EPI_ISL_509361, EPI_ISL_509362, EPI_ISL_509363, EPI_ISL_509364, EPI_ISL_509365, EPI_ISL_509366, EPI_ISL_509367, EPI_ISL_509368, EPI_ISL_509369, EPI_ISL_509370, EPI_ISL_509371, EPI_ISL_515568, EPI_ISL_515569, EPI_ISL_515570, EPI_ISL_515571, EPI_ISL_515572, EPI_ISL_515573, EPI_ISL_515574, EPI_ISL_515575, EPI_ISL_515576, EPI_ISL_515577, EPI_ISL_515578, EPI_ISL_515579, EPI_ISL_515580, EPI_ISL_515581, EPI_ISL_515582, EPI_ISL_515583, EPI_ISL_515584, EPI_ISL_515585, EPI_ISL_515586, EPI_ISL_515587, EPI_ISL_515588, EPI_ISL_515589, EPI_ISL_515590, EPI_ISL_515591, EPI_ISL_515592, EPI_ISL_515593, EPI_ISL_515594, EPI_ISL_515595, EPI_ISL_515596, EPI_ISL_515597, EPI_ISL_515598, EPI_ISL_515599, EPI_ISL_515600, EPI_ISL_515601, EPI_ISL_515602, EPI_ISL_515603, EPI_ISL_515604, EPI_ISL_515605, EPI_ISL_515606, EPI_ISL_515607, EPI_ISL_515608, EPI_ISL_515609, EPI_ISL_515610, EPI_ISL_515611, EPI_ISL_515612, EPI_ISL_515613, EPI_ISL_515614, EPI_ISL_515615, EPI_ISL_515616, EPI_ISL_515617, EPI_ISL_515618, EPI_ISL_515619, EPI_ISL_515620, EPI_ISL_515621, EPI_ISL_515622, EPI_ISL_515623, EPI_ISL_515624, EPI_ISL_515625, EPI_ISL_515626, EPI_ISL_515627, EPI_ISL_515628, EPI_ISL_515629, EPI_ISL_515630, EPI_ISL_515631, EPI_ISL_515632, EPI_ISL_515633, EPI_ISL_515634, EPI_ISL_515635, EPI_ISL_515636, EPI_ISL_515637, EPI_ISL_515638, EPI_ISL_515639, EPI_ISL_515640, EPI_ISL_515641, EPI_ISL_515642, EPI_ISL_515643,			

EPI_ISL_515644, EPI_ISL_515645, EPI_ISL_515646, EPI_ISL_515647, EPI_ISL_515648, EPI_ISL_515649, EPI_ISL_515650, EPI_ISL_515651, EPI_ISL_515652, EPI_ISL_515653, EPI_ISL_515654, EPI_ISL_515655, EPI_ISL_515656, EPI_ISL_515657, EPI_ISL_515658, EPI_ISL_515659, EPI_ISL_515660, EPI_ISL_515661, EPI_ISL_515662, EPI_ISL_515663, EPI_ISL_515664, EPI_ISL_515665, EPI_ISL_515666, EPI_ISL_515667, EPI_ISL_515668, EPI_ISL_515669, EPI_ISL_515670, EPI_ISL_515671, EPI_ISL_515672, EPI_ISL_515673, EPI_ISL_515674, EPI_ISL_515675, EPI_ISL_515676, EPI_ISL_515677, EPI_ISL_515678, EPI_ISL_515679, EPI_ISL_515680, EPI_ISL_515681, EPI_ISL_515682, EPI_ISL_515683, EPI_ISL_515684, EPI_ISL_515685, EPI_ISL_515686, EPI_ISL_515687, EPI_ISL_515688, EPI_ISL_515689, EPI_ISL_515690, EPI_ISL_515691, EPI_ISL_515692, EPI_ISL_515693, EPI_ISL_515694, EPI_ISL_515695, EPI_ISL_515696, EPI_ISL_515697, EPI_ISL_515698, EPI_ISL_515699, EPI_ISL_515700, EPI_ISL_515701, EPI_ISL_515702, EPI_ISL_515703, EPI_ISL_515704, EPI_ISL_515705, EPI_ISL_515706, EPI_ISL_515707, EPI_ISL_515708, EPI_ISL_515709, EPI_ISL_515710, EPI_ISL_515711, EPI_ISL_515712, EPI_ISL_515713, EPI_ISL_515714, EPI_ISL_515715, EPI_ISL_515716, EPI_ISL_515717, EPI_ISL_515718, EPI_ISL_515719, EPI_ISL_515720, EPI_ISL_515721, EPI_ISL_515722, EPI_ISL_515723, EPI_ISL_515724, EPI_ISL_515725, EPI_ISL_515726, EPI_ISL_515727, EPI_ISL_515728, EPI_ISL_515729, EPI_ISL_515730, EPI_ISL_515731, EPI_ISL_515732, EPI_ISL_515733, EPI_ISL_515734, EPI_ISL_515735, EPI_ISL_515736, EPI_ISL_515737, EPI_ISL_515738, EPI_ISL_515739, EPI_ISL_515740, EPI_ISL_515741, EPI_ISL_515742, EPI_ISL_515743, EPI_ISL_515744, EPI_ISL_515745, EPI_ISL_515746, EPI_ISL_515747, EPI_ISL_515748, EPI_ISL_515749, EPI_ISL_515750, EPI_ISL_515751, EPI_ISL_515752, EPI_ISL_515753, EPI_ISL_515754, EPI_ISL_515755, EPI_ISL_515756, EPI_ISL_515757, EPI_ISL_515758, EPI_ISL_515759, EPI_ISL_515760, EPI_ISL_515761, EPI_ISL_515762, EPI_ISL_515763, EPI_ISL_515764, EPI_ISL_515765, EPI_ISL_515766, EPI_ISL_515767, EPI_ISL_515768, EPI_ISL_515769, EPI_ISL_515770, EPI_ISL_515771, EPI_ISL_515772, EPI_ISL_515773, EPI_ISL_515774, EPI_ISL_515775, EPI_ISL_515776, EPI_ISL_515777, EPI_ISL_515778, EPI_ISL_515779, EPI_ISL_515780, EPI_ISL_515781, EPI_ISL_515782, EPI_ISL_515783, EPI_ISL_515784, EPI_ISL_515785, EPI_ISL_515786, EPI_ISL_515787, EPI_ISL_515788, EPI_ISL_515789, EPI_ISL_515790, EPI_ISL_515791, EPI_ISL_515792, EPI_ISL_515793, EPI_ISL_515794, EPI_ISL_515795, EPI_ISL_515796, EPI_ISL_515797, EPI_ISL_515798, EPI_ISL_515799, EPI_ISL_518033, EPI_ISL_518034, EPI_ISL_518035, EPI_ISL_518036, EPI_ISL_518037, EPI_ISL_518038, EPI_ISL_518039, EPI_ISL_518040, EPI_ISL_518041, EPI_ISL_518042, EPI_ISL_518043, EPI_ISL_518044, EPI_ISL_518045, EPI_ISL_518046, EPI_ISL_518047, EPI_ISL_518048, EPI_ISL_518049, EPI_ISL_518050, EPI_ISL_518051, EPI_ISL_518052, EPI_ISL_518053, EPI_ISL_529720, EPI_ISL_529721, EPI_ISL_529722, EPI_ISL_529723, EPI_ISL_529724, EPI_ISL_529725, EPI_ISL_529726, EPI_ISL_529727, EPI_ISL_529728, EPI_ISL_529729, EPI_ISL_529730, EPI_ISL_529731, EPI_ISL_529732, EPI_ISL_529733, EPI_ISL_529734, EPI_ISL_529735, EPI_ISL_529736, EPI_ISL_529737, EPI_ISL_529738, EPI_ISL_529739, EPI_ISL_529740, EPI_ISL_529741, EPI_ISL_529742, EPI_ISL_529743, EPI_ISL_529744, EPI_ISL_529745, EPI_ISL_529746, EPI_ISL_529747, EPI_ISL_529748, EPI_ISL_529749, EPI_ISL_529750, EPI_ISL_529751, EPI_ISL_529752, EPI_ISL_529753, EPI_ISL_529754, EPI_ISL_529755, EPI_ISL_529756, EPI_ISL_529757, EPI_ISL_529758, EPI_ISL_529759, EPI_ISL_529760, EPI_ISL_529761, EPI_ISL_529762, EPI_ISL_529763, EPI_ISL_529764, EPI_ISL_529765, EPI_ISL_529766, EPI_ISL_529767, EPI_ISL_529768, EPI_ISL_529769, EPI_ISL_529770, EPI_ISL_529771, EPI_ISL_529772, EPI_ISL_529773, EPI_ISL_529774, EPI_ISL_529775, EPI_ISL_529776, EPI_ISL_529777, EPI_ISL_529778, EPI_ISL_529779, EPI_ISL_529780, EPI_ISL_529781, EPI_ISL_529782, EPI_ISL_529783, EPI_ISL_529784, EPI_ISL_529785, EPI_ISL_529786, EPI_ISL_529787, EPI_ISL_529788, EPI_ISL_529789, EPI_ISL_529790, EPI_ISL_529791, EPI_ISL_529792, EPI_ISL_529793, EPI_ISL_529794, EPI_ISL_529795, EPI_ISL_529796, EPI_ISL_529797, EPI_ISL_529798, EPI_ISL_529799, EPI_ISL_529800, EPI_ISL_529801, EPI_ISL_529802, EPI_ISL_529803, EPI_ISL_535390, EPI_ISL_535391, EPI_ISL_535392, EPI_ISL_535393, EPI_ISL_535394, EPI_ISL_535395, EPI_ISL_535396, EPI_ISL_535397, EPI_ISL_535398, EPI_ISL_535399, EPI_ISL_535400, EPI_ISL_535401, EPI_ISL_535402, EPI_ISL_535403, EPI_ISL_535404, EPI_ISL_535405, EPI_ISL_535406, EPI_ISL_535407, EPI_ISL_535408, EPI_ISL_535409, EPI_ISL_535410, EPI_ISL_535411, EPI_ISL_535412, EPI_ISL_535413, EPI_ISL_535414, EPI_ISL_535415, EPI_ISL_535416, EPI_ISL_535417, EPI_ISL_535418, EPI_ISL_535419, EPI_ISL_535420, EPI_ISL_535421, EPI_ISL_535422, EPI_ISL_535423, EPI_ISL_535424, EPI_ISL_535425, EPI_ISL_535426, EPI_ISL_535427, EPI_ISL_535428, EPI_ISL_535429, EPI_ISL_535430, EPI_ISL_535431, EPI_ISL_535432, EPI_ISL_535433, EPI_ISL_535434, EPI_ISL_535435, EPI_ISL_535436, EPI_ISL_535437, EPI_ISL_535438, EPI_ISL_535439, EPI_ISL_535440, EPI_ISL_535441, EPI_ISL_535442, EPI_ISL_535443, EPI_ISL_535444, EPI_ISL_535445, EPI_ISL_535446, EPI_ISL_535447, EPI_ISL_535448, EPI_ISL_535449, EPI_ISL_535450, EPI_ISL_535451, EPI_ISL_535452, EPI_ISL_535453, EPI_ISL_535454, EPI_ISL_535455, EPI_ISL_535456, EPI_ISL_535457, EPI_ISL_535458, EPI_ISL_535459, EPI_ISL_535460, EPI_ISL_535461, EPI_ISL_535462, EPI_ISL_535463, EPI_ISL_535464, EPI_ISL_535465, EPI_ISL_535466, EPI_ISL_535467, EPI_ISL_535468, EPI_ISL_535469, EPI_ISL_535470, EPI_ISL_535471, EPI_ISL_535472, EPI_ISL_535473, EPI_ISL_535474, EPI_ISL_535475, EPI_ISL_535476, EPI_ISL_535477, EPI_ISL_535478, EPI_ISL_535479, EPI_ISL_535480, EPI_ISL_535481, EPI_ISL_535482, EPI_ISL_535483, EPI_ISL_535484, EPI_ISL_535485, EPI_ISL_535486, EPI_ISL_535487, EPI_ISL_535488, EPI_ISL_535496, EPI_ISL_535497, EPI_ISL_535498, EPI_ISL_535499, EPI_ISL_535500, EPI_ISL_535501, EPI_ISL_535502, EPI_ISL_535503, EPI_ISL_535504, EPI_ISL_535505, EPI_ISL_535506, EPI_ISL_535507, EPI_ISL_535508, EPI_ISL_535509, EPI_ISL_535510, EPI_ISL_535511, EPI_ISL_535512, EPI_ISL_535513, EPI_ISL_535514, EPI_ISL_535515, EPI_ISL_535516, EPI_ISL_535517, EPI_ISL_535518, EPI_ISL_535519, EPI_ISL_535520, EPI_ISL_535521, EPI_ISL_535522, EPI_ISL_535523, EPI_ISL_535524, EPI_ISL_535525, EPI_ISL_535526, EPI_ISL_535527, EPI_ISL_535528, EPI_ISL_535529, EPI_ISL_535530, EPI_ISL_535531, EPI_ISL_535532, EPI_ISL_535533, EPI_ISL_535534, EPI_ISL_535535, EPI_ISL_535536, EPI_ISL_535537, EPI_ISL_535538, EPI_ISL_535539, EPI_ISL_535540, EPI_ISL_535541, EPI_ISL_535542, EPI_ISL_535543, EPI_ISL_535544, EPI_ISL_535545, EPI_ISL_535546, EPI_ISL_535547, EPI_ISL_535548, EPI_ISL_535549, EPI_ISL_535550, EPI_ISL_535551, EPI_ISL_535552, EPI_ISL_535553, EPI_ISL_535554, EPI_ISL_535555, EPI_ISL_535556, EPI_ISL_535557, EPI_ISL_535558, EPI_ISL_535559, EPI_ISL_535560, EPI_ISL_535561, EPI_ISL_535562, EPI_ISL_535563, EPI_ISL_535564, EPI_ISL_535565, EPI_ISL_535566, EPI_ISL_535567, EPI_ISL_535568, EPI_ISL_535569, EPI_ISL_535570, EPI_ISL_535571, EPI_ISL_535572, EPI_ISL_602632, EPI_ISL_602633, EPI_ISL_602634, EPI_ISL_602635, EPI_ISL_602636, EPI_ISL_602637, EPI_ISL_602638, EPI_ISL_602639, EPI_ISL_602640, EPI_ISL_602641, EPI_ISL_602642, EPI_ISL_602643, EPI_ISL_602644, EPI_ISL_602645, EPI_ISL_602646, EPI_ISL_602647, EPI_ISL_602648, EPI_ISL_602649, EPI_ISL_602650, EPI_ISL_602651, EPI_ISL_602652, EPI_ISL_602653, EPI_ISL_602654, EPI_ISL_602655, EPI_ISL_602656, EPI_ISL_602657, EPI_ISL_602658, EPI_ISL_602659, EPI_ISL_602660, EPI_ISL_602661, EPI_ISL_602662, EPI_ISL_602663, EPI_ISL_602664, EPI_ISL_602665, EPI_ISL_602666, EPI_ISL_602667, EPI_ISL_602668, EPI_ISL_602669, EPI_ISL_602670, EPI_ISL_602671, EPI_ISL_602672, EPI_ISL_602673, EPI_ISL_602674, EPI_ISL_602675, EPI_ISL_602676, EPI_ISL_602677, EPI_ISL_602678, EPI_ISL_602679, EPI_ISL_602680, EPI_ISL_602681, EPI_ISL_602682, EPI_ISL_602683, EPI_ISL_602684, EPI_ISL_602685, EPI_ISL_602686, EPI_ISL_602687, EPI_ISL_602688, EPI_ISL_602689, EPI_ISL_602690, EPI_ISL_602691, EPI_ISL_602692, EPI_ISL_602693, EPI_ISL_602694, EPI_ISL_602695, EPI_ISL_602696, EPI_ISL_602697, EPI_ISL_602698, EPI_ISL_602699, EPI_ISL_602700, EPI_ISL_602701, EPI_ISL_602702, EPI_ISL_602703, EPI_ISL_602704, EPI_ISL_602705, EPI_ISL_602706, EPI_ISL_602707, EPI_ISL_602708, EPI_ISL_602709, EPI_ISL_602710, EPI_ISL_602711, EPI_ISL_602712, EPI_ISL_602713, EPI_ISL_602714, EPI_ISL_602715, EPI_ISL_602716, EPI_ISL_602717, EPI_ISL_602718, EPI_ISL_602719, EPI_ISL_602720, EPI_ISL_602721, EPI_ISL_602722, EPI_ISL_602723, EPI_ISL_602724, EPI_ISL_602725, EPI_ISL_602726, EPI_ISL_602727, EPI_ISL_602728, EPI_ISL_602729, EPI_ISL_602730, EPI_ISL_602731, EPI_ISL_602732, EPI_ISL_602733, EPI_ISL_602734, EPI_ISL_602735, EPI_ISL_602736, EPI_ISL_602737, EPI_ISL_602738, EPI_ISL_602739, EPI_ISL_602740, EPI_ISL_602741, EPI_ISL_602742, EPI_ISL_602743, EPI_ISL_602744, EPI_ISL_602745, EPI_ISL_602746, EPI_ISL_602747, EPI_ISL_602748, EPI_ISL_602749, EPI_ISL_602750, EPI_ISL_602751, EPI_ISL_602752, EPI_ISL_602753, EPI_ISL_602754, EPI_ISL_602755, EPI_ISL_602756, EPI_ISL_602757, EPI_ISL_602758, EPI_ISL_602759, EPI_ISL_602760, EPI_ISL_602761, EPI_ISL_602762, EPI_ISL_602763, EPI_ISL_602764, EPI_ISL_602765, EPI_ISL_602766, EPI_ISL_602767, EPI_ISL_602768, EPI_ISL_602769, EPI_ISL_602770, EPI_ISL_602771, EPI_ISL_602772, EPI_ISL_602773, EPI_ISL_602774, EPI_ISL_602775, EPI_ISL_602776, EPI_ISL_602777, EPI_ISL_602778, EPI_ISL_602779, EPI_ISL_602780, EPI_ISL_602781, EPI_ISL_602782, EPI_ISL_602783, EPI_ISL_602784, EPI_ISL_602785, EPI_ISL_602786, EPI_ISL_602787, EPI_ISL_602788, EPI_ISL_602789, EPI_ISL_602790, EPI_ISL_602791, EPI_ISL_602792, EPI_ISL_602793, EPI_ISL_602794, EPI_ISL_602795, EPI_ISL_602796, EPI_ISL_602797, EPI_ISL_602798, EPI_ISL_602799, EPI_ISL_602800, EPI_ISL_602801, EPI_ISL_602802, EPI_ISL_602803, EPI_ISL_602804, EPI_ISL_602805, EPI_ISL_602806, EPI_ISL_602807, EPI_ISL_602808, EPI_ISL_602809, EPI_ISL_602810, EPI_ISL_602811, EPI_ISL_602812, EPI_ISL_602813, EPI_ISL_602814, EPI_ISL_602815, EPI_ISL_602816, EPI_ISL_602817, EPI_ISL_602818, EPI_ISL_602819, EPI_ISL_602820, EPI_ISL_602821, EPI_ISL_602822, EPI_ISL_602823, EPI_ISL_602824, EPI_ISL_602825, EPI_ISL_602826, EPI_ISL_602827, EPI_ISL_602828, EPI_ISL_602829, EPI_ISL_602830, EPI_ISL_602831, EPI_ISL_602832, EPI_ISL_602833, EPI_ISL_602834, EPI_ISL_602835, EPI_ISL_602836, EPI_ISL_602837, EPI_ISL_602838, EPI_ISL_602839, EPI_ISL_602840, EPI_ISL_602841, EPI_ISL_602842, EPI_ISL_602843, EPI_ISL_602844, EPI_ISL_602845, EPI_ISL_602846, EPI_ISL_602847, EPI_ISL_602848, EPI_ISL_602849, EPI_ISL_602850, EPI_ISL_602851, EPI_ISL_602852, EPI_ISL_602853, EPI_ISL_602854, EPI_ISL_602855, EPI_ISL_602856, EPI_ISL_602857, EPI_ISL_602858, EPI_ISL_602859, EPI_ISL_602860, EPI_ISL_602861, EPI_ISL_602862, EPI_ISL_602863, EPI_ISL_602864, EPI_ISL_602865, EPI_ISL_602866, EPI_ISL_602867, EPI_ISL_602868, EPI_ISL_602869, EPI_ISL_602870, EPI_ISL_602871, EPI_ISL_602872, EPI_ISL_602873, EPI_ISL_602874, EPI_ISL_602875, EPI_ISL_602876, EPI_ISL_602877, EPI_ISL_602878, EPI_ISL_602879, EPI_ISL_602880, EPI_ISL_602881, EPI_ISL_602882, EPI_ISL_602883, EPI_ISL_602884, EPI_ISL_602885, EPI_ISL_602886, EPI_ISL_602887, EPI_ISL_602888, EPI_ISL_602889, EPI_ISL_602890, EPI_ISL_602891, EPI_ISL_602892, EPI_ISL_602893, EPI_ISL_602894, EPI_ISL_602895, EPI_ISL_602896, EPI_ISL_602897, EPI_ISL_602898, EPI_ISL_602899, EPI_ISL_602900, EPI_ISL_602901, EPI_ISL_602902, EPI_ISL_602903, EPI_ISL_602904, EPI_ISL_602905, EPI_ISL_602906, EPI_ISL_602907, EPI_ISL_602908, EPI_ISL_602909, EPI_ISL_602910, EPI_ISL_602911, EPI_ISL_602912, EPI_ISL_602913, EPI_ISL_602914, EPI_ISL_602915, EPI_ISL_602916, EPI_ISL_602917, EPI_ISL_602918, EPI_ISL_602919, EPI_ISL_602920, EPI_ISL_602921, EPI_ISL_602922, EPI_ISL_602923, EPI_ISL_602924, EPI_ISL_602925, EPI_ISL_602926, EPI_ISL_602927, EPI_ISL_602928, EPI_ISL_602929, EPI_ISL_602930, EPI_ISL_605785, EPI_ISL_605786, EPI_ISL_605787, EPI_ISL_605788, EPI_ISL_605789, EPI_ISL_605790, EPI_ISL_660159, EPI_ISL_660160, EPI_ISL_660161, EPI_ISL_660162, EPI_ISL_660163, EPI_ISL_660164, EPI_ISL_660165, EPI_ISL_660166, EPI_ISL_660167, EPI_ISL_660168, EPI_ISL_660169, EPI_ISL_660170, EPI_ISL_660171, EPI_ISL_660172, EPI_ISL_660173, EPI_ISL_660174, EPI_ISL_660175, EPI_ISL_660176, EPI_ISL_660177, EPI_ISL_660178, EPI_ISL_660179, EPI_ISL_660180, EPI_ISL_660181, EPI_ISL_660182, EPI_ISL_660183, EPI_ISL_660184, EPI_ISL_660185, EPI_ISL_660186, EPI_ISL_660187, EPI_ISL_660188, EPI_ISL_660189, EPI_ISL_660190, EPI_ISL_660191, EPI_ISL_660192, EPI_ISL_660193, EPI_ISL_660194, EPI_ISL_660195, EPI_ISL_660196, EPI_ISL_660197, EPI_ISL_660198, EPI_ISL_660199, EPI_ISL_660200, EPI_ISL_660201, EPI_ISL_660202, EPI_ISL_660203, EPI_ISL_660204, EPI_ISL_660205, EPI_ISL_660206, EPI_ISL_660207, EPI_ISL_660208, EPI_ISL_660209, EPI_ISL_660210, EPI_ISL_660211, EPI_ISL_660212, EPI_ISL_660213, EPI_ISL_660214, EPI_ISL_660215, EPI_ISL_660216, EPI_ISL_660217, EPI_ISL_660218, EPI_ISL_660219, EPI_ISL_660220, EPI_ISL_660223, EPI_ISL_660224, EPI_ISL_660225, EPI_ISL_660226, EPI_ISL_660227, EPI_ISL_660234, EPI_ISL_660235, EPI_ISL_660236, EPI_ISL_660237, EPI_ISL_660238, EPI_ISL_660239, EPI_ISL_660240, EPI_ISL_660241, EPI_ISL_660242, EPI_ISL_660243, EPI_ISL_660244, EPI_ISL_660245, EPI_ISL_660246, EPI_ISL_660247, EPI_ISL_660248, EPI_ISL_660249, EPI_ISL_660250, EPI_ISL_660251, EPI_ISL_660252, EPI_ISL_660253, EPI_ISL_660254, EPI_ISL_660255, EPI_ISL_660605, EPI_ISL_660606, EPI_ISL_660607, EPI_ISL_660608, EPI_ISL_660609, EPI_ISL_660610, EPI_ISL_660611, EPI_ISL_660612, EPI_ISL_660613, EPI_ISL_660614, EPI_ISL_660615, EPI_ISL_660616, EPI_ISL_660617, EPI_ISL_660618, EPI_ISL_660619, EPI_ISL_660620, EPI_ISL_660621, EPI_ISL_660622, EPI_ISL_660623, EPI_ISL_660624, EPI_ISL_660625, EPI_ISL_660626, EPI_ISL_660627, EPI_ISL_660628, EPI_ISL_660629, EPI_ISL_660630, EPI_ISL_660631, EPI_ISL_660632, EPI_ISL_660633, EPI_ISL_660634, EPI_ISL_660635, EPI_ISL_660636, EPI_ISL_660637, EPI_ISL_660638, EPI_ISL_660639, EPI_ISL_660640, EPI_ISL_660641, EPI_ISL_660642, EPI_ISL_660643, EPI_ISL_660644, EPI_ISL_660645, EPI_ISL_660646, EPI_ISL_660647, EPI_ISL_660648, EPI_ISL_660649, EPI_ISL_660650, EPI_ISL_660651, EPI_ISL_660652, EPI_ISL_660653, EPI_ISL_660654, EPI_ISL_660655, EPI_ISL_660656, EPI_ISL_660657, EPI_ISL_660658, EPI_ISL_660659, EPI_ISL_660660, EPI_ISL_660661, EPI_ISL_660662, EPI_ISL_660663, EPI_ISL_678566, EPI_ISL_678569, EPI_ISL_678570, EPI_ISL_678574, EPI_ISL_678575, EPI_ISL_678598, EPI_ISL_678599, EPI_ISL_678600, EPI_ISL_678601, EPI_ISL_678602, EPI_ISL_678603, EPI_ISL_678604, EPI_ISL_678605, EPI_ISL_678606, EPI_ISL_678607, EPI_ISL_678608, EPI_ISL_678609, EPI_ISL_678610, EPI_ISL_678611, EPI_ISL_678612, EPI_ISL_678613, EPI_ISL_678614, EPI_ISL_678615, EPI_ISL_678616, EPI_ISL_678617, EPI_ISL_678618, EPI_ISL_678619, EPI_ISL_678620, EPI_ISL_678621, EPI_ISL_678622, EPI_ISL_678623, EPI_ISL_678624, EPI_ISL_678625, EPI_ISL_678626, EPI_ISL_678627, EPI_ISL_678628, EPI_ISL_678629, EPI_ISL_678630, EPI_ISL_678631, EPI_ISL_678632, EPI_ISL_678633, EPI_ISL_678634, EPI_ISL_678635, EPI_ISL_678636, EPI_ISL_678637, EPI_ISL_678638, EPI_ISL_678639, EPI_ISL_678640, EPI_ISL_678641, EPI_ISL_678642, EPI_ISL_678643, EPI_ISL_736925, EPI_ISL_736926, EPI_ISL_736931, EPI_ISL_736932, EPI_ISL_736933, EPI_ISL_736934, EPI_ISL_736935, EPI_ISL_736938, EPI_ISL_736940, EPI_ISL_736941, EPI_ISL_736942, EPI_ISL_736943, EPI_ISL_736944, EPI_ISL_736945, EPI_ISL_736946, EPI_ISL_736947, EPI_ISL_736948, EPI_ISL_736949, EPI_ISL_736950, EPI_ISL_736951, EPI_ISL_736952, EPI_ISL_736953, EPI_ISL_736954, EPI_ISL_736955, EPI_ISL_736956, EPI_ISL_736957, EPI_ISL_736958, EPI_ISL_736959, EPI_ISL_736960, EPI_ISL_736969, EPI_ISL_736970, EPI_ISL_736971, EPI_ISL_736972, EPI_ISL_736973, EPI_ISL_736974, EPI_ISL_736975, EPI_ISL_736976, EPI_ISL_736977, EPI_ISL_736978, EPI_ISL_736979, EPI_ISL_736980, EPI_ISL_736984, EPI_ISL_736985, EPI_ISL_736986, EPI_ISL_736987, EPI_ISL_736988, EPI_ISL_736989, EPI_ISL_736990, EPI_ISL_736991, EPI_ISL_736992, EPI_ISL_736993, EPI_ISL_736994, EPI_ISL_825090, EPI_ISL_825091, EPI_ISL_825092, EPI_ISL_825093, EPI_ISL_825094, EPI_ISL_825095, EPI_ISL_825096, EPI_ISL_825097, EPI_ISL_825098, EPI_ISL_825099, EPI_ISL_825100, EPI_ISL_825101, EPI_ISL_825102, EPI_ISL_825103, EPI_ISL_825104, EPI_ISL_825105, EPI_ISL_825106, EPI_ISL_825107, EPI_ISL_825108, EPI_ISL_825109, EPI_ISL_825110, EPI_ISL_825111, EPI_ISL_825112, EPI_ISL_825113, EPI_ISL_825114, EPI_ISL_825115, EPI_ISL_825116, EPI_ISL_825117, EPI_ISL_825118, EPI_ISL_825119, EPI_ISL_825120, EPI_ISL_825121, EPI_ISL_825122, EPI_ISL_825123, EPI_ISL_825124, EPI_ISL_825125, EPI_ISL_825126, EPI_ISL_825127, EPI_ISL_825128, EPI_ISL_825129, EPI_ISL_825130, EPI_ISL_825131, EPI_ISL_825132, EPI_ISL_825133, EPI_ISL_825134, EPI_ISL_825135, EPI_ISL_825136, EPI_ISL_825137, EPI_ISL_825138, EPI_ISL_825139, EPI_ISL_825438, EPI_ISL_825439, EPI_ISL_825440, EPI_ISL_825441, EPI_ISL_825442, EPI_ISL_825443, EPI_ISL_825444, EPI_ISL_825445, EPI_ISL_825446, EPI_ISL_825447, EPI_ISL_825448, EPI_ISL_825449, EPI_ISL_825450, EPI_ISL_825451, EPI_ISL_825452, EPI_ISL_825453, EPI_ISL_825454, EPI_ISL_825455, EPI_ISL_825456, EPI_ISL_825457, EPI_ISL_825458, EPI_ISL_825459, EPI_ISL_825460, EPI_ISL_825461, EPI_ISL_825462, EPI_ISL_825463, EPI_ISL_825464, EPI_ISL_825465, EPI_ISL_825466, EPI_ISL_825467, EPI_ISL_825468, EPI_ISL_825469, EPI_ISL_825470, EPI_ISL_825471, EPI_ISL_825472, EPI_ISL_825473, EPI_ISL_825474, EPI_ISL_825475, EPI_ISL_825476, EPI_ISL_825477, EPI_ISL_825478, EPI_ISL_825479, EPI_ISL_825480, EPI_ISL_825481, EPI_ISL_825482, EPI_ISL_825483, EPI_ISL_825484, EPI_ISL_825485, EPI_ISL_825486, EPI_ISL_825487, EPI_ISL_825488, EPI_ISL_825489			
see above	NHLS-IALCH	KRISP, KZN Research Innovation and Sequencing Platform	Cele S; Chimukangara B; ChimukangaraB; Gazy I; Giandhari J; Karim F; Khan S; Lessells R; Mdlalose K; Pillay S; Sigal A; Tegally H; Wilkinson E; York D; de Oliveira T
EPI_ISL_860554, EPI_ISL_860555, EPI_ISL_860556, EPI_ISL_860557, EPI_ISL_860558, EPI_ISL_860559, EPI_ISL_860560, EPI_ISL_860561, EPI_ISL_860562, EPI_ISL_860563, EPI_ISL_860564, EPI_ISL_860565, EPI_ISL_860566, EPI_ISL_860567, EPI_ISL_860568, EPI_ISL_860569, EPI_ISL_860570, EPI_ISL_860571, EPI_ISL_860572, EPI_ISL_860573, EPI_ISL_860574, EPI_ISL_860575, EPI_ISL_860576, EPI_ISL_860577, EPI_ISL_860578, EPI_ISL_860579, EPI_ISL_860580, EPI_ISL_860581, EPI_ISL_860582, EPI_ISL_860583, EPI_ISL_860584, EPI_ISL_860585, EPI_ISL_860586, EPI_ISL_860587, EPI_ISL_860588, EPI_ISL_860589, EPI_ISL_860590, EPI_ISL_860591, EPI_ISL_860592, EPI_ISL_860593, EPI_ISL_860594, EPI_ISL_860595, EPI_ISL_860596, EPI_ISL_860597, EPI_ISL_860598, EPI_ISL_860599, EPI_ISL_860600, EPI_ISL_860601, EPI_ISL_860602, EPI_ISL_860603, EPI_ISL_860604, EPI_ISL_860605, EPI_ISL_860606, EPI_ISL_860607, EPI_ISL_860608, EPI_ISL_860609, EPI_ISL_860610, EPI_ISL_860611, EPI_ISL_860612, EPI_ISL_860613, EPI_ISL_860614, EPI_ISL_860615, EPI_ISL_860616, EPI_ISL_860617, EPI_ISL_860618, EPI_ISL_860619, EPI_ISL_860620, EPI_ISL_860621, EPI_ISL_860622, EPI_ISL_860623, EPI_ISL_860624, EPI_ISL_860625, EPI_ISL_860626, EPI_ISL_860627, EPI_ISL_860628, EPI_ISL_860629, EPI_ISL_860630			
see above	NHLS-IALCH	KRISP, KZn Research Innovation and Sequencing Platform	Giandhari J; Khan S; Lessells R; Mdlalose K; Pillay S; Tegally H; Wilkinson E; York D; de Oliveira T
EPI_ISL_736928, EPI_ISL_736929, EPI_ISL_736939, EPI_ISL_736981, EPI_ISL_736982, EPI_ISL_736983	NHLS-UCT	KRISP, KZN Research Innovation and Sequencing Platform	Arash Iranzadeh; Bruna Galvao; Carolyn Williamson; Deelan Doolabh; Diana Hardie; Emanuel James San; Houriiyah Tegally; Innocent Mudau; Jennifer Giandhari; Kruger Marais; Lynn Tyers; Marvin Hsiao; Stephen Korsman; Sureshnee Pillay; Tulio de Oliveira
EPI_ISL_418241, EPI_ISL_418242, EPI_ISL_420037, EPI_ISL_766861, EPI_ISL_766862, EPI_ISL_766863, EPI_ISL_766864, EPI_ISL_766865, EPI_ISL_766866, EPI_ISL_766867, EPI_ISL_766868, EPI_ISL_766869, EPI_ISL_766870, EPI_ISL_766871, EPI_ISL_766872, EPI_ISL_766873, EPI_ISL_766874, EPI_ISL_766875			
see above	NIC Viral Respiratory Unit - Institut Pasteur of Algeria	National Reference Center for Viruses of Respiratory Infections, Institut Pasteur, Paris	Angela Brisebarre; Etienne Simon-Lorière; Fawzi Derrar; Flora Donati; Marion Barbet; Maud Vanpeene; Mélanie Albert; Méline Bizard; Sylvie Behillil; Sylvie van der Werf; Vincent Enouf
EPI_ISL_422382, EPI_ISL_422384, EPI_ISL_422387, EPI_ISL_422390, EPI_ISL_422394, EPI_ISL_422397, EPI_ISL_422398, EPI_ISL_422399, EPI_ISL_422400, EPI_ISL_422401, EPI_ISL_422402, EPI_ISL_422403, EPI_ISL_422404, EPI_ISL_422405, EPI_ISL_422406			
see above	NMIMR, Department of Virology	WACCBIP, University of Ghana	Abraham Kwabena Anang; Augustina Arjaquah; Augustina Arjarquah; Bright Adu; Collins M. Misita; Collins M. Morang'a; Dominic S. Y. Amuzu; Erasmus Kotey; Evelyn Bonney; Fred Tei-Maya; George B. Kyei; Gordon A. Awandare; Ivy Asante; Joyce M. Ngoi; Kofi Bonney; Linda Boatemaa; Miriam Eshun; Peter Quashie; Selassie Kumordjie; Vanessa Magnussen; William Ampofo
EPI_ISL_745188	Nababeep Hospital	National Health Laboratory Service (NHLS), Tygerberg	Bronwyn Kleinhans; Eduan Wilkindon; Gert van Zyl; Houriiyah Tegally; Kayla Delaney; Susan Engelbrecht; Tulio de Oliveira; Wolfgang Preiser
EPI_ISL_1208956, EPI_ISL_1208957, EPI_ISL_1208958, EPI_ISL_1208959, EPI_ISL_1208960, EPI_ISL_1208961, EPI_ISL_1208963, EPI_ISL_1208964, EPI_ISL_1208965, EPI_ISL_1208966, EPI_ISL_1208969, EPI_ISL_1208970, EPI_ISL_1208971, EPI_ISL_1208972, EPI_ISL_1208973, EPI_ISL_1208974, EPI_ISL_1208975, EPI_ISL_1208976, EPI_ISL_1208977, EPI_ISL_1208978, EPI_ISL_1208979, EPI_ISL_1208980, EPI_ISL_1208981, EPI_ISL_1208982, EPI_ISL_1208983, EPI_ISL_1208984, EPI_ISL_1208985, EPI_ISL_1208986, EPI_ISL_1208987, EPI_ISL_1208988, EPI_ISL_1208989, EPI_ISL_1208990, EPI_ISL_1208991, EPI_ISL_1208992, EPI_ISL_1208993, EPI_ISL_1208994, EPI_ISL_1208995, EPI_ISL_1208996, EPI_ISL_1208997, EPI_ISL_1208998, EPI_ISL_1208999, EPI_ISL_1209004, EPI_ISL_1209005, EPI_ISL_1209006, EPI_ISL_1209007, EPI_ISL_1209008, EPI_ISL_1209009, EPI_ISL_1209010			
see above	Namibia Institute of Pathology (NIP)	National Institute for Communicable Diseases of the National Health Laboratory Service	Amoako DG; Bhiman JN; Ismail A; Konstantinus I; Mahlangu B; Mohale T; Ntuli N; Scheepers C; Van Rooyen G
EPI_ISL_560385, EPI_ISL_560387, EPI_ISL_560388, EPI_ISL_560389, EPI_ISL_560390	National Health Laboratory	Botswana Institute for Technology Research and Innovation	Dineo Emang Tshiamo; Dineo Emang Tshiamo. Gape Nyepetsi; Gape Nyepetsi; Kefentse Arnold Tumedi; Madisa Mine; Maitshwarelo Ignatius Matsheka; Thongbotho Mphoyakgosi
EPI_ISL_560386	National Health Laboratory	Botswana Institute for Technology Research and innovation	Dineo Emang Tshiamo. Gape Nyepetsi; Kefentse Arnold Tumedi; Madisa Mine; Maitshwarelo Ignatius Matsheka; Thongbotho Mphoyakgosi
EPI_ISL_622942, EPI_ISL_622944, EPI_ISL_622945, EPI_ISL_622954, EPI_ISL_622958, EPI_ISL_622970, EPI_ISL_622971, EPI_ISL_622972, EPI_ISL_622973, EPI_ISL_622974, EPI_ISL_622975, EPI_ISL_622976, EPI_ISL_622977, EPI_ISL_622979, EPI_ISL_622980, EPI_ISL_622981, EPI_ISL_622982, EPI_ISL_622984, EPI_ISL_622987, EPI_ISL_622989, EPI_ISL_622991, EPI_ISL_622992, EPI_ISL_622993, EPI_ISL_622995, EPI_ISL_622996, EPI_ISL_622997, EPI_ISL_622998, EPI_ISL_622999, EPI_ISL_623000, EPI_ISL_623001, EPI_ISL_623002, EPI_ISL_623003, EPI_ISL_623004, EPI_ISL_623005, EPI_ISL_623006, EPI_ISL_623007, EPI_ISL_623008, EPI_ISL_623009, EPI_ISL_623010, EPI_ISL_623011, EPI_ISL_623012, EPI_ISL_623013, EPI_ISL_623014, EPI_ISL_623015, EPI_ISL_623016, EPI_ISL_623017, EPI_ISL_623018, EPI_ISL_623019, EPI_ISL_623020, EPI_ISL_623021, EPI_ISL_623022, EPI_ISL_623023, EPI_ISL_623024, EPI_ISL_623026, EPI_ISL_623027, EPI_ISL_623029, EPI_ISL_623030, EPI_ISL_623031, EPI_ISL_623032, EPI_ISL_623033, EPI_ISL_623034, EPI_ISL_623035, EPI_ISL_623036, EPI_ISL_623037, EPI_ISL_623038, EPI_ISL_623039, EPI_ISL_623040, EPI_ISL_623041, EPI_ISL_623042, EPI_ISL_623043, EPI_ISL_623044, EPI_ISL_623045, EPI_ISL_623046, EPI_ISL_623047, EPI_ISL_623048, EPI_ISL_623049, EPI_ISL_623050, EPI_ISL_623051, EPI_ISL_623052, EPI_ISL_623053, EPI_ISL_623054, EPI_ISL_623055, EPI_ISL_623056, EPI_ISL_623057, EPI_ISL_623058, EPI_ISL_623059, EPI_ISL_623060, EPI_ISL_623061, EPI_ISL_623062, EPI_ISL_623063, EPI_ISL_623065, EPI_ISL_623066, EPI_ISL_623067, EPI_ISL_623069, EPI_ISL_623070, EPI_ISL_623071, EPI_ISL_623072			
see above	National Health Laboratory Service	National Institute for Communicable Diseases of the National Health Laboratory Service	Allam M; Bhiman JN; Ismail A; Khumalo Z; Kwenda S; Mnyameni F; Mohale T; Mtshali P; Subramoney K
EPI_ISL_464112, EPI_ISL_464113, EPI_ISL_464114, EPI_ISL_464115, EPI_ISL_464116, EPI_ISL_464117, EPI_ISL_464118, EPI_ISL_464119, EPI_ISL_464120, EPI_ISL_464121, EPI_ISL_464122, EPI_ISL_464123, EPI_ISL_464124, EPI_ISL_464125, EPI_ISL_464126, EPI_ISL_464127, EPI_ISL_464128, EPI_ISL_464129, EPI_ISL_464130, EPI_ISL_464131, EPI_ISL_464132, EPI_ISL_464133, EPI_ISL_464135, EPI_ISL_464136, EPI_ISL_464137, EPI_ISL_464138, EPI_ISL_464139, EPI_ISL_464140, EPI_ISL_464141, EPI_ISL_464142, EPI_ISL_464143, EPI_ISL_464144, EPI_ISL_464145, EPI_ISL_464146, EPI_ISL_464147, EPI_ISL_464148, EPI_ISL_464149, EPI_ISL_464150, EPI_ISL_464151, EPI_ISL_464152, EPI_ISL_464153, EPI_ISL_464154, EPI_ISL_464155, EPI_ISL_464156, EPI_ISL_464157, EPI_ISL_464158			
see above	National Health Laboratory Service (NHLS), Tygerberg	Division of Medical Virology, Stellenbosch University and National Health Laboratory Service (NHLS)	Bronwyn Kleinhans; Eduan Wilkindon; Gert van Zyl; Houriiyah Tegally; Kayla Delaney; Susan Engelbrecht; Tulio de Oliveira; Wolfgang Preiser
EPI_ISL_464134	National Health Laboratory Service (NHLS), Tygerberg	Stellenbosch University and NHLS	Bronwyn Kleinhans; Eduan Wilkindon; Gert van Zyl; Houriiyah Tegally; Kayla Delaney; Susan Engelbrecht; Tulio de Oliveira; Wolfgang Preiser
EPI_ISL_634978, EPI_ISL_634979, EPI_ISL_634980, EPI_ISL_634981, EPI_ISL_634982, EPI_ISL_634983, EPI_ISL_634984, EPI_ISL_634985, EPI_ISL_634986, EPI_ISL_634987, EPI_ISL_634988, EPI_ISL_634989, EPI_ISL_634990, EPI_ISL_634991, EPI_ISL_634992, EPI_ISL_634993, EPI_ISL_634994, EPI_ISL_634995, EPI_ISL_634996, EPI_ISL_634997, EPI_ISL_634998, EPI_ISL_634999, EPI_ISL_635000, EPI_ISL_635001, EPI_ISL_635002, EPI_ISL_635003, EPI_ISL_635004, EPI_ISL_635005, EPI_ISL_635006, EPI_ISL_635007, EPI_ISL_635008, EPI_ISL_635009, EPI_ISL_635010, EPI_ISL_635011, EPI_ISL_635012, EPI_ISL_635013, EPI_ISL_635014, EPI_ISL_635015, EPI_ISL_635016, EPI_ISL_635017, EPI_ISL_635018, EPI_ISL_635019, EPI_ISL_635020, EPI_ISL_635021, EPI_ISL_635022, EPI_ISL_635023, EPI_ISL_635024, EPI_ISL_635025, EPI_ISL_635026, EPI_ISL_635027, EPI_ISL_635028, EPI_ISL_635029, EPI_ISL_635030, EPI_ISL_635031, EPI_ISL_635032, EPI_ISL_635033, EPI_ISL_635034, EPI_ISL_635035, EPI_ISL_635036, EPI_ISL_635037, EPI_ISL_635038, EPI_ISL_635039, EPI_ISL_635040, EPI_ISL_635041, EPI_ISL_635042, EPI_ISL_635043, EPI_ISL_635044, EPI_ISL_635045, EPI_ISL_635046, EPI_ISL_635047, EPI_ISL_635048, EPI_ISL_635049, EPI_ISL_635050, EPI_ISL_635051, EPI_ISL_635052, EPI_ISL_635053, EPI_ISL_635054, EPI_ISL_635055, EPI_ISL_635056, EPI_ISL_635057, EPI_ISL_635058			
see above	National Health Laboratory Service - Inkosi Albert Luthuli Central Hospital (NHLS-IALCH)	KRISP, KZN Research Innovation and Sequencing Platform	Giandhari J; Khan S; Lessells R; Mdlalose K; Pillay S; Tegally H; Wilkinson E; York D; de Oliveira T
EPI_ISL_944124, EPI_ISL_944125, EPI_ISL_944126, EPI_ISL_944127, EPI_ISL_944128, EPI_ISL_944129, EPI_ISL_944130, EPI_ISL_944131, EPI_ISL_944132, EPI_ISL_944133, EPI_ISL_944134, EPI_ISL_944135, EPI_ISL_944136, EPI_ISL_944137, EPI_ISL_944138, EPI_ISL_944139, EPI_ISL_944140, EPI_ISL_944141, EPI_ISL_944142, EPI_ISL_944143, EPI_ISL_944144, EPI_ISL_944145, EPI_ISL_944146, EPI_ISL_944147, EPI_ISL_944148, EPI_ISL_944149, EPI_ISL_944150, EPI_ISL_944151, EPI_ISL_944152, EPI_ISL_944153, EPI_ISL_944154, EPI_ISL_944155, EPI_ISL_944156, EPI_ISL_944157, EPI_ISL_944158, EPI_ISL_944159, EPI_ISL_944160, EPI_ISL_944161, EPI_ISL_944162, EPI_ISL_944163, EPI_ISL_944164, EPI_ISL_944165, EPI_ISL_944166, EPI_ISL_944167, EPI_ISL_944168, EPI_ISL_944169, EPI_ISL_944170, EPI_ISL_944171, EPI_ISL_944172, EPI_ISL_944173, EPI_ISL_944174, EPI_ISL_944175, EPI_ISL_944176, EPI_ISL_944177, EPI_ISL_1250424, EPI_ISL_1250425, EPI_ISL_1250427, EPI_ISL_1250428, EPI_ISL_1250430, EPI_ISL_1250431, EPI_ISL_1250432, EPI_ISL_1250434, EPI_ISL_1250436, EPI_ISL_1250437, EPI_ISL_1250439, EPI_ISL_1250441, EPI_ISL_1250442, EPI_ISL_1250444, EPI_ISL_1250446, EPI_ISL_1250447, EPI_ISL_1250448, EPI_ISL_1250450, EPI_ISL_1250452, EPI_ISL_1250453, EPI_ISL_1250455, EPI_ISL_1250456, EPI_ISL_1250458, EPI_ISL_1250460, EPI_ISL_1250462, EPI_ISL_1250463, EPI_ISL_1250465, EPI_ISL_1250466, EPI_ISL_1250468, EPI_ISL_1250469, EPI_ISL_1250472, EPI_ISL_1250474, EPI_ISL_1250476, EPI_ISL_1250477, EPI_ISL_1250479, EPI_ISL_1250480, EPI_ISL_1250482, EPI_ISL_1250483, EPI_ISL_1250485, EPI_ISL_1250486, EPI_ISL_1250488, EPI_ISL_1250490, EPI_ISL_1250491, EPI_ISL_1250493, EPI_ISL_1250494, EPI_ISL_1250496, EPI_ISL_1250497, EPI_ISL_1250499, EPI_ISL_1250501, EPI_ISL_1250502, EPI_ISL_1250504, EPI_ISL_1250505			
see above	National Health Laboratory Service, South Africa	KRISP, KZN Research Innovation and Sequencing Platform	Emmanuel SJ; Giandhari J; Khan S; Laguda-Akingba O; Lessells R; Maslo C; Mdlalose K; Pillay S; Sitharam L; Tegally H; Wilkinson E; York D; de Oliveira T
EPI_ISL_1048431, EPI_ISL_1048432, EPI_ISL_1048433, EPI_ISL_1048434, EPI_ISL_1048435, EPI_ISL_1048436, EPI_ISL_1048437, EPI_ISL_1048438, EPI_ISL_1048439, EPI_ISL_1048440, EPI_ISL_1048441, EPI_ISL_1048442, EPI_ISL_1048443, EPI_ISL_1048444, EPI_ISL_1048445, EPI_ISL_1048446, EPI_ISL_1048447, EPI_ISL_1048448, EPI_ISL_1048449, EPI_ISL_1048450, EPI_ISL_1048451, EPI_ISL_1048452, EPI_ISL_1048453, EPI_ISL_1048454, EPI_ISL_1048455, EPI_ISL_1048456, EPI_ISL_1048457, EPI_ISL_1048458, EPI_ISL_1048459, EPI_ISL_1048460, EPI_ISL_1048461, EPI_ISL_1048462, EPI_ISL_1048463, EPI_ISL_1048464, EPI_ISL_1048465, EPI_ISL_1048466, EPI_ISL_1048467, EPI_ISL_1048468, EPI_ISL_1048469, EPI_ISL_1048470, EPI_ISL_1048471, EPI_ISL_1048472, EPI_ISL_1048473, EPI_ISL_1048474, EPI_ISL_1048475, EPI_ISL_1048476, EPI_ISL_1048477, EPI_ISL_1048478, EPI_ISL_1048479, EPI_ISL_1048480, EPI_ISL_1048481, EPI_ISL_1048482, EPI_ISL_1048483, EPI_ISL_1048484, EPI_ISL_1048485, EPI_ISL_1048486, EPI_ISL_1048487, EPI_ISL_1048488, EPI_ISL_1048489, EPI_ISL_1048490, EPI_ISL_1048491, EPI_ISL_1048492, EPI_ISL_1048493, EPI_ISL_1048494, EPI_ISL_1048495, EPI_ISL_1048496,			

EPI_ISL_1048497, EPI_ISL_1048498, EPI_ISL_1048499, EPI_ISL_1048500, EPI_ISL_1048501, EPI_ISL_1048502, EPI_ISL_1048503, EPI_ISL_1048504, EPI_ISL_1048505, EPI_ISL_1048506, EPI_ISL_1048507, EPI_ISL_1048508, EPI_ISL_1048509, EPI_ISL_1048510, EPI_ISL_1048511, EPI_ISL_1048512, EPI_ISL_1048513, EPI_ISL_1048514, EPI_ISL_1048515, EPI_ISL_1048516, EPI_ISL_1048517, EPI_ISL_1048518, EPI_ISL_1048519, EPI_ISL_1048520, EPI_ISL_1048521, EPI_ISL_1048522, EPI_ISL_1048523, EPI_ISL_1048524, EPI_ISL_1048525, EPI_ISL_1048526, EPI_ISL_1048527, EPI_ISL_1048528, EPI_ISL_1048529, EPI_ISL_1048530, EPI_ISL_1048531, EPI_ISL_1048532, EPI_ISL_1048533, EPI_ISL_1048534, EPI_ISL_1048535, EPI_ISL_1048536, EPI_ISL_1048537, EPI_ISL_1048538, EPI_ISL_1048539, EPI_ISL_1048540, EPI_ISL_1048541, EPI_ISL_1048542, EPI_ISL_1048543, EPI_ISL_1048544, EPI_ISL_1048545, EPI_ISL_1048546, EPI_ISL_1048547, EPI_ISL_1048548, EPI_ISL_1048549, EPI_ISL_1048550, EPI_ISL_1048551, EPI_ISL_1048552, EPI_ISL_1048553, EPI_ISL_1048554, EPI_ISL_1048555, EPI_ISL_1048556, EPI_ISL_1048557, EPI_ISL_1048558, EPI_ISL_1048559, EPI_ISL_1048560, EPI_ISL_1048561, EPI_ISL_1048562, EPI_ISL_1048563, EPI_ISL_1048564, EPI_ISL_1048565, EPI_ISL_1048566, EPI_ISL_1048567, EPI_ISL_1048568			
see above	National Health Laboratory Service, South Africa	KRISP, KZn Research Innovation and Sequencing Platform	Emmanuel SJ; Giandhari J; Khan S; Laguda-Akingba O; Lessells R; Mdlalose K; Pillay S; Tegally H; Wilkinson E; York D; de Oliveira T
EPI_ISL_1080932, EPI_ISL_1080933, EPI_ISL_1080963, EPI_ISL_1080966, EPI_ISL_1080969, EPI_ISL_1080970, EPI_ISL_1080974			
see above	National Health Laboratory Services	National Health Laboratory Services	Kathleen Subramoney; Mushal Ali; Thabo Mohale
EPI_ISL_1069202, EPI_ISL_1069394, EPI_ISL_1070401	National Health Laboratory Services	National Health Laboratory Services, Virology	Kathleen Subramoney; Mushal Ali; Thabo Mohale
EPI_ISL_770470, EPI_ISL_770471, EPI_ISL_770472, EPI_ISL_770473, EPI_ISL_770474	National Health laboratory	Botswana Institute for Technology Research and Innovation	Dineo Emang Tshiamo. Gape Nyepetsi; Kefentse Arnold Tumedi; Madisa Mine; Maitshwarelo Ignatius Matsheka; Malebogo Kebabonye; Thongbotho Mphoyakgosi
EPI_ISL_450482	National Influenza and other Respiratory Viruses Centre-Tunisia, Virology Unit, Microbiology Laboratory, Charles Nicolle Hospital	National Influenza and other Respiratory Viruses Centre-Tunisia, Virology Unit, Microbiology Laboratory, Charles Nicolle Hospital	Abid, S.; Ben Nasr, M.; Charaa, L.; D. and Boutiba, I.; El Moussi, A.; Enigrou; Landolsi, I.
EPI_ISL_417186, EPI_ISL_430297, EPI_ISL_435058, EPI_ISL_435059, EPI_ISL_450296, EPI_ISL_450297, EPI_ISL_450298, EPI_ISL_450299, EPI_ISL_450300, EPI_ISL_450301, EPI_ISL_450495, EPI_ISL_470878, EPI_ISL_470879, EPI_ISL_470880, EPI_ISL_490255, EPI_ISL_490256, EPI_ISL_490257, EPI_ISL_490258, EPI_ISL_490259, EPI_ISL_490260, EPI_ISL_490261, EPI_ISL_490262, EPI_ISL_490263, EPI_ISL_490264, EPI_ISL_490265, EPI_ISL_490266, EPI_ISL_490267, EPI_ISL_490268, EPI_ISL_490269, EPI_ISL_490270, EPI_ISL_490271, EPI_ISL_490272, EPI_ISL_490273, EPI_ISL_490274, EPI_ISL_490275, EPI_ISL_490276, EPI_ISL_490277, EPI_ISL_490278, EPI_ISL_490279, EPI_ISL_490280, EPI_ISL_490281, EPI_ISL_490282, EPI_ISL_490283, EPI_ISL_490284, EPI_ISL_490285, EPI_ISL_490286, EPI_ISL_490287, EPI_ISL_490288, EPI_ISL_490289, EPI_ISL_490290, EPI_ISL_490291, EPI_ISL_490292, EPI_ISL_490293, EPI_ISL_490294, EPI_ISL_490295, EPI_ISL_490296, EPI_ISL_490297, EPI_ISL_490298, EPI_ISL_490299, EPI_ISL_490300, EPI_ISL_490301, EPI_ISL_490302, EPI_ISL_490303, EPI_ISL_490304, EPI_ISL_490305, EPI_ISL_490306, EPI_ISL_490307, EPI_ISL_490308, EPI_ISL_490309, EPI_ISL_490310, EPI_ISL_490311, EPI_ISL_490312, EPI_ISL_490313, EPI_ISL_490314, EPI_ISL_504186, EPI_ISL_504187, EPI_ISL_504188, EPI_ISL_504189, EPI_ISL_504190, EPI_ISL_504191, EPI_ISL_504192, EPI_ISL_504193, EPI_ISL_504194, EPI_ISL_504195, EPI_ISL_504196, EPI_ISL_504197, EPI_ISL_504198, EPI_ISL_504199, EPI_ISL_504200, EPI_ISL_504201, EPI_ISL_504202, EPI_ISL_504203, EPI_ISL_504204, EPI_ISL_504205, EPI_ISL_504206, EPI_ISL_504207, EPI_ISL_504208, EPI_ISL_504209, EPI_ISL_504210, EPI_ISL_504211, EPI_ISL_504212, EPI_ISL_504213, EPI_ISL_504214, EPI_ISL_504215, EPI_ISL_504216, EPI_ISL_504217, EPI_ISL_504218, EPI_ISL_504219, EPI_ISL_504220, EPI_ISL_504221, EPI_ISL_504222, EPI_ISL_504223, EPI_ISL_504224, EPI_ISL_504225, EPI_ISL_504226, EPI_ISL_504227, EPI_ISL_504228, EPI_ISL_504229, EPI_ISL_504230, EPI_ISL_504231, EPI_ISL_504232, EPI_ISL_504233, EPI_ISL_504234, EPI_ISL_504235, EPI_ISL_504236, EPI_ISL_504237, EPI_ISL_504238, EPI_ISL_504239, EPI_ISL_504240, EPI_ISL_504241, EPI_ISL_504242, EPI_ISL_504243, EPI_ISL_504244, EPI_ISL_514355, EPI_ISL_514356, EPI_ISL_514357, EPI_ISL_514358, EPI_ISL_514359, EPI_ISL_514360, EPI_ISL_514361, EPI_ISL_514362, EPI_ISL_514363, EPI_ISL_514364, EPI_ISL_514365, EPI_ISL_514366, EPI_ISL_514367, EPI_ISL_514368, EPI_ISL_514369, EPI_ISL_514370, EPI_ISL_514371, EPI_ISL_514372, EPI_ISL_514373, EPI_ISL_514374, EPI_ISL_514375, EPI_ISL_514376, EPI_ISL_514377, EPI_ISL_514378, EPI_ISL_514379, EPI_ISL_514380, EPI_ISL_514381, EPI_ISL_514382, EPI_ISL_514383, EPI_ISL_514384, EPI_ISL_514385, EPI_ISL_514386, EPI_ISL_514387, EPI_ISL_514388, EPI_ISL_514389, EPI_ISL_514390, EPI_ISL_514391, EPI_ISL_514392, EPI_ISL_514393, EPI_ISL_514394, EPI_ISL_514395, EPI_ISL_514396, EPI_ISL_514397, EPI_ISL_514398, EPI_ISL_514399, EPI_ISL_514400, EPI_ISL_514401, EPI_ISL_514402, EPI_ISL_514403, EPI_ISL_514404, EPI_ISL_514405, EPI_ISL_514406, EPI_ISL_514407, EPI_ISL_514408, EPI_ISL_514409, EPI_ISL_514410, EPI_ISL_514411, EPI_ISL_514412, EPI_ISL_514413, EPI_ISL_514414, EPI_ISL_514415, EPI_ISL_514416, EPI_ISL_514417, EPI_ISL_514418, EPI_ISL_514419, EPI_ISL_514420, EPI_ISL_514421, EPI_ISL_514422, EPI_ISL_514423, EPI_ISL_514424, EPI_ISL_515133, EPI_ISL_515134, EPI_ISL_515135, EPI_ISL_515136, EPI_ISL_515137, EPI_ISL_515138, EPI_ISL_515139, EPI_ISL_515140, EPI_ISL_515141, EPI_ISL_515142, EPI_ISL_515143, EPI_ISL_515144, EPI_ISL_515145, EPI_ISL_515146, EPI_ISL_515147, EPI_ISL_515148, EPI_ISL_515149, EPI_ISL_515150, EPI_ISL_515151, EPI_ISL_515152, EPI_ISL_515153, EPI_ISL_515154, EPI_ISL_515155, EPI_ISL_515156, EPI_ISL_515157, EPI_ISL_515158, EPI_ISL_515159, EPI_ISL_515160, EPI_ISL_515161, EPI_ISL_515162, EPI_ISL_515163, EPI_ISL_515164, EPI_ISL_515165, EPI_ISL_515166, EPI_ISL_515167, EPI_ISL_515168, EPI_ISL_515169, EPI_ISL_515170, EPI_ISL_515171, EPI_ISL_515172, EPI_ISL_515173, EPI_ISL_515174, EPI_ISL_515175, EPI_ISL_515176, EPI_ISL_515177, EPI_ISL_515178, EPI_ISL_515179, EPI_ISL_515180, EPI_ISL_622894, EPI_ISL_622895, EPI_ISL_622896, EPI_ISL_622897, EPI_ISL_622898, EPI_ISL_622899, EPI_ISL_622900, EPI_ISL_622901, EPI_ISL_622902, EPI_ISL_622903, EPI_ISL_622904, EPI_ISL_622905, EPI_ISL_622906, EPI_ISL_622907, EPI_ISL_622908, EPI_ISL_622909, EPI_ISL_622910, EPI_ISL_622911, EPI_ISL_622912, EPI_ISL_622913, EPI_ISL_622914, EPI_ISL_622915, EPI_ISL_622916, EPI_ISL_622917, EPI_ISL_622918, EPI_ISL_622919, EPI_ISL_622920, EPI_ISL_622921, EPI_ISL_622922, EPI_ISL_622923, EPI_ISL_622924, EPI_ISL_622925, EPI_ISL_622926, EPI_ISL_622927, EPI_ISL_622928, EPI_ISL_622929, EPI_ISL_622930, EPI_ISL_622931, EPI_ISL_622932, EPI_ISL_622933, EPI_ISL_622934, EPI_ISL_622935, EPI_ISL_622936, EPI_ISL_622937, EPI_ISL_622938, EPI_ISL_622939, EPI_ISL_622940, EPI_ISL_622941			
see above	National Institute for Communicable Diseases of the National Health Laboratory Service	National Institute for Communicable Diseases of the National Health Laboratory Service	A; Allam M; Bhiman JN; Ismail A; Khumalo Z; Kwenda S; Mnyameni F; Mohale T; Mtshali P; Subramoney K; van Heusden P; von Gottberg
EPI_ISL_939656, EPI_ISL_939657, EPI_ISL_939658, EPI_ISL_939659, EPI_ISL_940067, EPI_ISL_1239850, EPI_ISL_1239851, EPI_ISL_1239852, EPI_ISL_1239853, EPI_ISL_1239854, EPI_ISL_1239855, EPI_ISL_1239856, EPI_ISL_1239857, EPI_ISL_1239858, EPI_ISL_1239859, EPI_ISL_1239860, EPI_ISL_1239861, EPI_ISL_1239862, EPI_ISL_1239863, EPI_ISL_1239864, EPI_ISL_1239865, EPI_ISL_1239866, EPI_ISL_1239867, EPI_ISL_1239868, EPI_ISL_1239869, EPI_ISL_1239870, EPI_ISL_1239871, EPI_ISL_1239872, EPI_ISL_1239873, EPI_ISL_1239874, EPI_ISL_1239875, EPI_ISL_1239876, EPI_ISL_1239877, EPI_ISL_1239878, EPI_ISL_1239879, EPI_ISL_1239880, EPI_ISL_1239881, EPI_ISL_1239882			
see above	National Institute for Communicable Diseases,National Health Laboratory Services, Gauteng, South Africa	National Institute for Communicable Diseases of the National Health Laboratory Service	Allam M; Amoako DG; Bhiman JN; Ismail A; Mahlangu B; Mohale T; Ntuli N; Scheepers C
EPI_ISL_644738, EPI_ISL_644739, EPI_ISL_644740, EPI_ISL_644741, EPI_ISL_644742, EPI_ISL_644743, EPI_ISL_644744, EPI_ISL_644745, EPI_ISL_644746, EPI_ISL_644747, EPI_ISL_644748, EPI_ISL_644749, EPI_ISL_644750, EPI_ISL_644751, EPI_ISL_644752, EPI_ISL_644753, EPI_ISL_644754, EPI_ISL_644755, EPI_ISL_644756, EPI_ISL_644757, EPI_ISL_644758, EPI_ISL_644759, EPI_ISL_644760, EPI_ISL_644761, EPI_ISL_644762, EPI_ISL_644763, EPI_ISL_644764, EPI_ISL_644765, EPI_ISL_644766, EPI_ISL_644767, EPI_ISL_644768, EPI_ISL_644769, EPI_ISL_644770, EPI_ISL_644771, EPI_ISL_644772, EPI_ISL_644773, EPI_ISL_644774, EPI_ISL_644775, EPI_ISL_644776, EPI_ISL_644777, EPI_ISL_644778, EPI_ISL_644779, EPI_ISL_644780, EPI_ISL_644781, EPI_ISL_644782, EPI_ISL_644783, EPI_ISL_644784, EPI_ISL_644785, EPI_ISL_644786, EPI_ISL_644787, EPI_ISL_644788, EPI_ISL_644789, EPI_ISL_644791, EPI_ISL_644792, EPI_ISL_644793, EPI_ISL_644794, EPI_ISL_644795, EPI_ISL_644796, EPI_ISL_644797, EPI_ISL_644798, EPI_ISL_644800, EPI_ISL_644801, EPI_ISL_644802, EPI_ISL_644803, EPI_ISL_644804, EPI_ISL_644805, EPI_ISL_644806, EPI_ISL_644807, EPI_ISL_644808, EPI_ISL_644809, EPI_ISL_644810, EPI_ISL_644811, EPI_ISL_644812, EPI_ISL_644813, EPI_ISL_644814, EPI_ISL_644815, EPI_ISL_644816, EPI_ISL_644817, EPI_ISL_644818, EPI_ISL_644819, EPI_ISL_644820, EPI_ISL_644821, EPI_ISL_644822, EPI_ISL_647970, EPI_ISL_647971, EPI_ISL_647972, EPI_ISL_647973, EPI_ISL_647974, EPI_ISL_647975, EPI_ISL_647976, EPI_ISL_647977, EPI_ISL_647978, EPI_ISL_647979, EPI_ISL_647980, EPI_ISL_647981, EPI_ISL_647982, EPI_ISL_647983, EPI_ISL_647984, EPI_ISL_1191782, EPI_ISL_1191783, EPI_ISL_1191784, EPI_ISL_1191785, EPI_ISL_1191786, EPI_ISL_1191787, EPI_ISL_1191788, EPI_ISL_1191789, EPI_ISL_1191790, EPI_ISL_1191791, EPI_ISL_1191792, EPI_ISL_1191793, EPI_ISL_1191794, EPI_ISL_1191795, EPI_ISL_1191796, EPI_ISL_1191797, EPI_ISL_1191798, EPI_ISL_1191799, EPI_ISL_1191800, EPI_ISL_1191801, EPI_ISL_1191802, EPI_ISL_1191803, EPI_ISL_1191804, EPI_ISL_1191805, EPI_ISL_1191806, EPI_ISL_1191807, EPI_ISL_1191808, EPI_ISL_1191809, EPI_ISL_1191810, EPI_ISL_1191811, EPI_ISL_1191812, EPI_ISL_1191813, EPI_ISL_1191814, EPI_ISL_1191815, EPI_ISL_1191816, EPI_ISL_1191817, EPI_ISL_1191818, EPI_ISL_1191819, EPI_ISL_1191820, EPI_ISL_1191821, EPI_ISL_1191822, EPI_ISL_1191823, EPI_ISL_1191824, EPI_ISL_1191825, EPI_ISL_1191826, EPI_ISL_1191827, EPI_ISL_1191828, EPI_ISL_1191829, EPI_ISL_1191830, EPI_ISL_1191831, EPI_ISL_1191832, EPI_ISL_1191833, EPI_ISL_1191834, EPI_ISL_1191835, EPI_ISL_1191836, EPI_ISL_1191837, EPI_ISL_1191838, EPI_ISL_1191839, EPI_ISL_1191840, EPI_ISL_1191841, EPI_ISL_1191842, EPI_ISL_1191843, EPI_ISL_1191844, EPI_ISL_1191845, EPI_ISL_1191846, EPI_ISL_1191847, EPI_ISL_1191848, EPI_ISL_1191849, EPI_ISL_1191850, EPI_ISL_1191851, EPI_ISL_1191852, EPI_ISL_1191853, EPI_ISL_1191854, EPI_ISL_1191855, EPI_ISL_1191856, EPI_ISL_1191857, EPI_ISL_1191858, EPI_ISL_1191859, EPI_ISL_1191860, EPI_ISL_1191861, EPI_ISL_1191862, EPI_ISL_1191863, EPI_ISL_1191864, EPI_ISL_1191865, EPI_ISL_1191866, EPI_ISL_1191867, EPI_ISL_1191868, EPI_ISL_1191869, EPI_ISL_1191870, EPI_ISL_1191871, EPI_ISL_1191872, EPI_ISL_1191873, EPI_ISL_1191874, EPI_ISL_1191875, EPI_ISL_1191876, EPI_ISL_1191877, EPI_ISL_1191878, EPI_ISL_1191879, EPI_ISL_1191880, EPI_ISL_1191881, EPI_ISL_1191882, EPI_ISL_1191883, EPI_ISL_1191884, EPI_ISL_1191885, EPI_ISL_1191886, EPI_ISL_1191887, EPI_ISL_1191888, EPI_ISL_1191889, EPI_ISL_1191890, EPI_ISL_1191891, EPI_ISL_1191892, EPI_ISL_1191893, EPI_ISL_1191894, EPI_ISL_1191895, EPI_ISL_1191896, EPI_ISL_1191897, EPI_ISL_1191898, EPI_ISL_1191899, EPI_ISL_1191900, EPI_ISL_1191901, EPI_ISL_1191902, EPI_ISL_1191903, EPI_ISL_1191904, EPI_ISL_1191905, EPI_ISL_1191906, EPI_ISL_1191907, EPI_ISL_1191908, EPI_ISL_1191909, EPI_ISL_1191910, EPI_ISL_1191911, EPI_ISL_1191912, EPI_ISL_1191913, EPI_ISL_1191914, EPI_ISL_1191915, EPI_ISL_1191916, EPI_ISL_1191917, EPI_ISL_1191918, EPI_ISL_1191919, EPI_ISL_1191920, EPI_ISL_1191921, EPI_ISL_1191922, EPI_ISL_1191923, EPI_ISL_1191924, EPI_ISL_1191925, EPI_ISL_1191926, EPI_ISL_1191927, EPI_ISL_1191928, EPI_ISL_1191929, EPI_ISL_1191930, EPI_ISL_1191931, EPI_ISL_1191932, EPI_ISL_1191933, EPI_ISL_1191934, EPI_ISL_1191935, EPI_ISL_1191936, EPI_ISL_1191937, EPI_ISL_1191938, EPI_ISL_1191939, EPI_ISL_1191940, EPI_ISL_1191941, EPI_ISL_1191942, EPI_ISL_1191943, EPI_ISL_1191944, EPI_ISL_1191945, EPI_ISL_1191946, EPI_ISL_1191947, EPI_ISL_1191948, EPI_ISL_1191949, EPI_ISL_1191950, EPI_ISL_1191951, EPI_ISL_1191952, EPI_ISL_1191953, EPI_ISL_1191954, EPI_ISL_1191955, EPI_ISL_1191956, EPI_ISL_1191957, EPI_ISL_1191958, EPI_ISL_1191959, EPI_ISL_1191960, EPI_ISL_1191961, EPI_ISL_1191962, EPI_ISL_1191963, EPI_ISL_1191964, EPI_ISL_1191965, EPI_ISL_1191966, EPI_ISL_1191967, EPI_ISL_1191968, EPI_ISL_1191969, EPI_ISL_1191970, EPI_ISL_1191971, EPI_ISL_1191972, EPI_ISL_1191973, EPI_ISL_1191974, EPI_ISL_1191975, EPI_ISL_1191976, EPI_ISL_1191977, EPI_ISL_1191978, EPI_ISL_1191979, EPI_ISL_1191980, EPI_ISL_1191981, EPI_ISL_1191982, EPI_ISL_1191983, EPI_ISL_1191984, EPI_ISL_1191985, EPI_ISL_1191986, EPI_ISL_1191987, EPI_ISL_1191988, EPI_ISL_1191989, EPI_ISL_1191990, EPI_ISL_1191991, EPI_ISL_1191992, EPI_ISL_1191993, EPI_ISL_1191994, EPI_ISL_1191995, EPI_ISL_1191996, EPI_ISL_1191997, EPI_ISL_1191998, EPI_ISL_1191999, EPI_ISL_1192000, EPI_ISL_1192001, EPI_ISL_1192002, EPI_ISL_1192003, EPI_ISL_1192004, EPI_ISL_1192005, EPI_ISL_1192006, EPI_ISL_1192007, EPI_ISL_1192008, EPI_ISL_1192009, EPI_ISL_1192010, EPI_ISL_1192011, EPI_ISL_1192012, EPI_ISL_1192013, EPI_ISL_1192014, EPI_ISL_1192015, EPI_ISL_1192016, EPI_ISL_1192017, EPI_ISL_1192018, EPI_ISL_1192019, EPI_ISL_1192020, EPI_ISL_1192021, EPI_ISL_1192022, EPI_ISL_1192023, EPI_ISL_1192024, EPI_ISL_1192025, EPI_ISL_1192026, EPI_ISL_1192027, EPI_ISL_1192028, EPI_ISL_1192029, EPI_ISL_1192030, EPI_ISL_1192031, EPI_ISL_1192032, EPI_ISL_1192033, EPI_ISL_1192034, EPI_ISL_1192035, EPI_ISL_1192036, EPI_ISL_1192037, EPI_ISL_1192038, EPI_ISL_1192039, EPI_ISL_1192040, EPI_ISL_1192041, EPI_ISL_1192042, EPI_ISL_1192043, EPI_ISL_1192044, EPI_ISL_1192045, EPI_ISL_1192046, EPI_ISL_1192047, EPI_ISL_1192048, EPI_ISL_1192049, EPI_ISL_1192050, EPI_ISL_1192051, EPI_ISL_1192052, EPI_ISL_1192053, EPI_ISL_1192054, EPI_ISL_1192055, EPI_ISL_1192056, EPI_ISL_1192057, EPI_ISL_1192058, EPI_ISL_1192059, EPI_ISL_1192060, EPI_ISL_1192061, EPI_ISL_1192062, EPI_ISL_1192063, EPI_ISL_1192064, EPI_ISL_1192065, EPI_ISL_1192066, EPI_ISL_1192067, EPI_ISL_1192068, EPI_ISL_1192069, EPI_ISL_1192070, EPI_ISL_1192071, EPI_ISL_1192072, EPI_ISL_1192073, EPI_ISL_1192074, EPI_ISL_1192075, EPI_ISL_1195295, EPI_ISL_1195296, EPI_ISL_1195297, EPI_ISL_1195298, EPI_ISL_1195299, EPI_ISL_1195300, EPI_ISL_1195301			
see above	National Microbiology Reference Laboratory	Quadram Institute Bioscience	Agnes Juru; Alexander Goredema; Ana-Victoria Gutierrez; Andrew J. Page; Andrew Tarupiwa; Barbra Murwira; Beuty Makamure; Charles Nyagupe; David Baker; Faustinos T Takawira; Gaetan Thilliez; Gemma Kay; Gibson Mhlanga; Hlanai Gumbo; Isaac Phiri; Justin O'Grady; Kenneth K Maeka; Leonardo de Oliveira Martins; Muchaneta Mugabe; Portia Manangazira; Raiva Simbi; Robert Kingsley; Sekesai Zinyowera; Tapfumanei Mashe; Tatenda Takawira; Thanh Le Viet
EPI_ISL_457827, EPI_ISL_457828, EPI_ISL_457829, EPI_ISL_457830, EPI_ISL_457831, EPI_ISL_457832, EPI_ISL_457833, EPI_ISL_457834, EPI_ISL_457835, EPI_ISL_457836, EPI_ISL_457837, EPI_ISL_457838, EPI_ISL_457839, EPI_ISL_457840, EPI_ISL_457841, EPI_ISL_457842, EPI_ISL_457843, EPI_ISL_457844			
see above	National Public Health Laboratory	KEMRI-Wellcome Trust Research Programme/KEMRI-CGMR-C Kilifi	Githinji G. et al 2020
EPI_ISL_845546, EPI_ISL_845547, EPI_ISL_845548, EPI_ISL_845549, EPI_ISL_845550, EPI_ISL_845551, EPI_ISL_845552, EPI_ISL_845553, EPI_ISL_845554, EPI_ISL_845555, EPI_ISL_845556, EPI_ISL_845557, EPI_ISL_845558, EPI_ISL_845559, EPI_ISL_845560, EPI_ISL_845561, EPI_ISL_845562, EPI_ISL_845563, EPI_ISL_845564, EPI_ISL_845565			
see above	National Public Health Laboratory, Cameroon	African Centre of Excellence for Genomics of Infectious Diseases (ACEGID), Redeemer's University	Oluniyi P.E. et al
EPI_ISL_1082297	National Reference Laboratory, Nigeria Centre for Disease Control.	National Reference Laboratory, Nigeria Centre for Disease Control, Gaduwa, Abuja, Nigeria	Anthony Ahumibe; Catherine Okoi; Chimaobi Chukwu; Dr Chikwe Ihekweazu; Dr Ndodo Nnaemeka; Dr Omoare Adesuyi; Grace Esebanmen; Naidoo Dhamari; Nwando Mba; Olusola Anuoluwapo Akanbi
EPI_ISL_737200, EPI_ISL_737201, EPI_ISL_737205, EPI_ISL_737206, EPI_ISL_737207, EPI_ISL_1073135	National Reference Laboratory, Nigeria Centre for Disease Control.	National Reference Laboratory, Nigeria Centre for Disease Control, Gaduwa,Abuja, Nigeria	(JHU); Abdulmajid Musa; Adama Ahmad; Akinpelu Afolabi; Anthony Ahumibe; Bamidele Olorunfemi; Catherine Okoi; Celestina Obiekea; Chimaobi Chukwu; Dr Adesuyi Omoare; Dr Chikwe Ihekweazu; Dr Chikwe Ihekweazu.; Dr Chinwe Ochu; Dr Ndodo Nnaemeka; Dr Omoare Adesuyi; Dr Sikiru Badaru; Esebanmen Grace; Grace Esebanmen; Kingsley Madubuike; Kingsley Njoku; Michael Popoola; Naidoo Dhamari; Nneamaka Uba; Nwando Mba; Olusola Akanbi; Olusola Anuoluwapo Akanbi; Peter Van Heusden (SANBI); Shirlee Wohl; oluwaseyi Ajegbe
EPI_ISL_770475	National health Laboratory	Botswana Institute for Technology Research and Innovation	Dineo Emang Tshiamo. Gape Nyepetsi; Kefentse Arnold Tumedi; Madisa Mine; Maitshwarelo Ignatius Matsheka; Malebogo Kebabonye; Thongbotho Mphoyakgosi
EPI_ISL_678548, EPI_ISL_678549, EPI_ISL_678550, EPI_ISL_678551, EPI_ISL_678552, EPI_ISL_678553, EPI_ISL_678554, EPI_ISL_678555, EPI_ISL_678556, EPI_ISL_678557, EPI_ISL_678558, EPI_ISL_678559, EPI_ISL_678560, EPI_ISL_678561, EPI_ISL_678562, EPI_ISL_678563, EPI_ISL_678564, EPI_ISL_678565, EPI_ISL_678567, EPI_ISL_678568, EPI_ISL_678571, EPI_ISL_678572, EPI_ISL_678573, EPI_ISL_678576, EPI_ISL_678577, EPI_ISL_678578, EPI_ISL_678579, EPI_ISL_678580, EPI_ISL_678581, EPI_ISL_678582, EPI_ISL_678583, EPI_ISL_678584, EPI_ISL_678585, EPI_ISL_678586, EPI_ISL_678587, EPI_ISL_678588, EPI_ISL_678589, EPI_ISL_678590, EPI_ISL_678591, EPI_ISL_678592, EPI_ISL_678593, EPI_ISL_678594, EPI_ISL_678595, EPI_ISL_678596, EPI_ISL_678597, EPI_ISL_736936, EPI_ISL_736937, EPI_ISL_736961, EPI_ISL_736962, EPI_ISL_736963, EPI_ISL_736964, EPI_ISL_736965			
see above	Netcare	KRISP, KZN Research Innovation and Sequencing Platform	ChimukangaraB; Giandhari J; Khan S; Lessells R; Mdlalose K; Pillay S; Tegally H; Wilkinson E; York D; de Oliveira T
EPI_ISL_1085951, EPI_ISL_1085952, EPI_ISL_1085953, EPI_ISL_1085954, EPI_ISL_1085955, EPI_ISL_1085956, EPI_ISL_1085957, EPI_ISL_1085958, EPI_ISL_1085959, EPI_ISL_1085960, EPI_ISL_1085961, EPI_ISL_1085962, EPI_ISL_1085963, EPI_ISL_1085964, EPI_ISL_1085965, EPI_ISL_1085966, EPI_ISL_1085967, EPI_ISL_1085968, EPI_ISL_1085969, EPI_ISL_1085970, EPI_ISL_1085971, EPI_ISL_1085972, EPI_ISL_1085973, EPI_ISL_1085974, EPI_ISL_1085975, EPI_ISL_1085976, EPI_ISL_1085977, EPI_ISL_1085978, EPI_ISL_1085979, EPI_ISL_1085980, EPI_ISL_1085981, EPI_ISL_1085982, EPI_ISL_1085983, EPI_ISL_1085984, EPI_ISL_1085985, EPI_ISL_1085986, EPI_ISL_1085987, EPI_ISL_1085988, EPI_ISL_1085989, EPI_ISL_1085990, EPI_ISL_1085991, EPI_ISL_1085992, EPI_ISL_1085993, EPI_ISL_1085994, EPI_ISL_1085995, EPI_ISL_1085996, EPI_ISL_1085997, EPI_ISL_1085998, EPI_ISL_1085999, EPI_ISL_1086000, EPI_ISL_1086001, EPI_ISL_1086002, EPI_ISL_1086003, EPI_ISL_1086004, EPI_ISL_1086005, EPI_ISL_1086006, EPI_ISL_1086007, EPI_ISL_1086008, EPI_ISL_1086009, EPI_ISL_1086010, EPI_ISL_1086011, EPI_ISL_1086012, EPI_ISL_1086013, EPI_ISL_1086014, EPI_ISL_1086015, EPI_ISL_1086016, EPI_ISL_1086017, EPI_ISL_1086018, EPI_ISL_1086019, EPI_ISL_1086020, EPI_ISL_1086021, EPI_ISL_1086022, EPI_ISL_1086023, EPI_ISL_1086024, EPI_ISL_1086025, EPI_ISL_1086026, EPI_ISL_1086027, EPI_ISL_1086028, EPI_ISL_1086029, EPI_ISL_1086030, EPI_ISL_1086031, EPI_ISL_1086032, EPI_ISL_1086033, EPI_ISL_1250356, EPI_ISL_1250358, EPI_ISL_1250359, EPI_ISL_1250361, EPI_ISL_1250362, EPI_ISL_1250364, EPI_ISL_1250366, EPI_ISL_1250367, EPI_ISL_1250369, EPI_ISL_1250370, EPI_ISL_1250371, EPI_ISL_1250373, EPI_ISL_1250375, EPI_ISL_1250376, EPI_ISL_1250378, EPI_ISL_1250379, EPI_ISL_1250381, EPI_ISL_1250383, EPI_ISL_1250385, EPI_ISL_1250386, EPI_ISL_1250388, EPI_ISL_1250390, EPI_ISL_1250392, EPI_ISL_1250393, EPI_ISL_1250394, EPI_ISL_1250396, EPI_ISL_1250397, EPI_ISL_1250399, EPI_ISL_1250401, EPI_ISL_1250402, EPI_ISL_1250404, EPI_ISL_1250405, EPI_ISL_1250407, EPI_ISL_1250409, EPI_ISL_1250410, EPI_ISL_1250412, EPI_ISL_1250413, EPI_ISL_1250414, EPI_ISL_1250416, EPI_ISL_1250417, EPI_ISL_1250419, EPI_ISL_1250420, EPI_ISL_1250422			
see above	Netcare/AMPATH	KRISP, KZN Research Innovation and Sequencing Platform	Emmanuel SJ; Giandhari J; Khan S; Lessells R; Maslo C; Mdlalose K; Pillay S; Sitharam L; Tegally H; Wilkinson E; York D; de Oliveira T
EPI_ISL_700442, EPI_ISL_700444	New Horizon Clinic wc NZC	NHLS/UCT	Arash Iranzadeh; Bruna Galvao; Carolyn Williamson; Deelan Doolabh; Diana Hardie; Houriiyah Tegally; Innocent Mudau; Kruger Marais; Lynn Tyers; Marvin Hsiao; Stephen Korsman
EPI_ISL_960116	New Horizon Clinic wc NZC	National Health Laboratory Service/UCT	Arash Iranzadeh; Bruna Galvao; Carolyn Williamson; Deelan Doolabh; Diana Hardie; Innocent Mudau; Kruger Marais; Lynn Tyers; Marvin Hsiao; Stephen Korsman
EPI_ISL_696468, EPI_ISL_696489	New Horizon Clinic wc NZC & NHLS/UCT	KRISP, KZN Research Innovation and Sequencing Platform	Arash Iranzadeh; Bruna Galvao; Carolyn Williamson; Deelan Doolabh; Diana Hardie; Emanuel James San; Houriiyah Tegally; Innocent Mudau; Jennifer Giandhari; Kruger Marais; Lynn Tyers; Marvin Hsiao; Stephen Korsman; Sureshnee Pillay; Tulio de Oliveira
EPI_ISL_960175	New Somerset Hospital wc NSH	National Health Laboratory Service/UCT	Arash Iranzadeh; Bruna Galvao; Carolyn Williamson; Deelan Doolabh; Diana Hardie; Innocent Mudau; Kruger Marais; Lynn Tyers; Marvin Hsiao; Stephen Korsman
EPI_ISL_1196008, EPI_ISL_1196009, EPI_ISL_1196010, EPI_ISL_1196020, EPI_ISL_1196021	Nhlangano Health Centre	National Institute for Communicable Diseases of the National Health Laboratory Service	Amoako DG; Bhiman JN; Ismail A; Mahlangu B; Maphalala GP; Mohale T; Ntuli N; Scheepers C
EPI_ISL_1040703	Nieuveldpark Clinic wc NVP	NHLS/UCT	Arash Iranzadeh; Bruna Galvao; Carolyn Williamson; Deelan Doolabh; Diana Hardie; Innocent Mudau; Kruger Marais; Lynn Tyers; Marvin Hsiao; Stephen Korsman
EPI_ISL_1197041	Nigeria Centre For Disease Control	National Reference Laboratory, NCDC, Gaduwa, Abuja	Anthony Ahumibe; Catherine Okoi; Chimaobi Chukwu; Dr Chikwe Ihekweazu; Dr Ndodo Nnaemeka; Dr Omoare Adesuyi; Naidoo Dhamari; Nwando Mba; Olusola Anuoluwapo Akanbi
EPI_ISL_825385, EPI_ISL_825437, EPI_ISL_825617, EPI_ISL_825632, EPI_ISL_825633, EPI_ISL_825713, EPI_ISL_825714, EPI_ISL_825807, EPI_ISL_1073137, EPI_ISL_1073139, EPI_ISL_1073638, EPI_ISL_1073639, EPI_ISL_1073640, EPI_ISL_1197062, EPI_ISL_1197063, EPI_ISL_1197064, EPI_ISL_1197065, EPI_ISL_1197066, EPI_ISL_1197129, EPI_ISL_1197130, EPI_ISL_1197150, EPI_ISL_1197933, EPI_ISL_1198006			

see above	Nigeria Centre For Disease Control	National reference Laboratory, NCDC, Gaduwa, Abuja	Anthony Ahumibe; Cahterine Okoi; Catherine Okoi; Celestina Obiekea; Chimaobi Chukwu; Chinwe Ochu; Dr Chikwe Ihekweazu; Dr Ndodo Nnaemeka; Dr Omoare Adesuyi; Esebanmen Grace; Grace Esebanmen; Kingsley Madubuike; Kingsley Njoku; Naidoo Dhamari; Nwando Mba; Olusola Anuoluwapo Akanbi
EPI_ISL_766051	Nigeria Centre For Disease Control,	National reference Laboratory, NCDC, Gaduwa, Abuja	Anthony Ahumibe; Chimaobi Chukwu; Dr Chikwe Ihekweazu; Dr Ndodo Nnaemeka; Dr Omoare Adesuyi; Kingsley Madubuike; Naidoo Dhamari; Nwando Mba; Olusola Akanbi
EPI_ISL_455422, EPI_ISL_455426	Nigeria Centre for Disease Control	African Centre of Excellence for Genomics of Infectious Diseases (ACEGID), Redeemer's University, Ede, Osun State, Nigeria	Ajogbasile F.V.; Folarin O.A.; Happi C.T.; Ihekweazu C.; Kayode A.; Oguzie J.; Olawoye I.; Olumade T.; Oluniyi P.E.; Uwanibe J.
EPI_ISL_1173207, EPI_ISL_1173208, EPI_ISL_1173209, EPI_ISL_1173210, EPI_ISL_1173211, EPI_ISL_1173212, EPI_ISL_1173213, EPI_ISL_1173214, EPI_ISL_1173215, EPI_ISL_1173216, EPI_ISL_1173217, EPI_ISL_1173218, EPI_ISL_1173219, EPI_ISL_1173220, EPI_ISL_1173221, EPI_ISL_1173222, EPI_ISL_1173223, EPI_ISL_1173224, EPI_ISL_1173225, EPI_ISL_1173226, EPI_ISL_1173227, EPI_ISL_1173228, EPI_ISL_1173229, EPI_ISL_1173230, EPI_ISL_1173231, EPI_ISL_1173232, EPI_ISL_1173233, EPI_ISL_1173234, EPI_ISL_1173235, EPI_ISL_1173236, EPI_ISL_1173237, EPI_ISL_1173238, EPI_ISL_1173239, EPI_ISL_1173240, EPI_ISL_1173241, EPI_ISL_1173242, EPI_ISL_1173243, EPI_ISL_1173244, EPI_ISL_1173245, EPI_ISL_1173246			
see above	Nigeria Centre for Disease Control (NCDC)	African Centre for Excellence for Genomics of Infectious Diseases (ACEGID), Redeemer's University	I.B.; Olawoye; et al
EPI_ISL_872601, EPI_ISL_872602, EPI_ISL_872603, EPI_ISL_872604, EPI_ISL_872605, EPI_ISL_872606, EPI_ISL_872607, EPI_ISL_872608, EPI_ISL_872609, EPI_ISL_872610, EPI_ISL_872611, EPI_ISL_872612, EPI_ISL_872613, EPI_ISL_872614, EPI_ISL_872615, EPI_ISL_872616, EPI_ISL_872617, EPI_ISL_872618, EPI_ISL_872619, EPI_ISL_872620, EPI_ISL_872621, EPI_ISL_872622, EPI_ISL_872623, EPI_ISL_872624, EPI_ISL_872625, EPI_ISL_872626, EPI_ISL_872627, EPI_ISL_906275, EPI_ISL_906276, EPI_ISL_906277, EPI_ISL_906278, EPI_ISL_906279, EPI_ISL_906280, EPI_ISL_906281, EPI_ISL_906282, EPI_ISL_906283, EPI_ISL_906284, EPI_ISL_906285, EPI_ISL_906286, EPI_ISL_906287, EPI_ISL_906288, EPI_ISL_906289, EPI_ISL_906290, EPI_ISL_906291, EPI_ISL_906292, EPI_ISL_906293, EPI_ISL_906294, EPI_ISL_906295, EPI_ISL_906296, EPI_ISL_906297, EPI_ISL_906298, EPI_ISL_906299, EPI_ISL_906300, EPI_ISL_906301, EPI_ISL_906302, EPI_ISL_906303, EPI_ISL_906304, EPI_ISL_906305, EPI_ISL_941275, EPI_ISL_941276, EPI_ISL_941277, EPI_ISL_941278, EPI_ISL_941279, EPI_ISL_941280, EPI_ISL_941281, EPI_ISL_941282, EPI_ISL_941283, EPI_ISL_941284, EPI_ISL_941285, EPI_ISL_941286, EPI_ISL_941287, EPI_ISL_941288, EPI_ISL_941289, EPI_ISL_941290, EPI_ISL_941291, EPI_ISL_941292, EPI_ISL_941293, EPI_ISL_941294, EPI_ISL_941295, EPI_ISL_941296, EPI_ISL_941297, EPI_ISL_941925, EPI_ISL_941926, EPI_ISL_1242009, EPI_ISL_1242010, EPI_ISL_1242011, EPI_ISL_1242012, EPI_ISL_1242013, EPI_ISL_1242014, EPI_ISL_1242015, EPI_ISL_1242016, EPI_ISL_1242017, EPI_ISL_1242018, EPI_ISL_1242019, EPI_ISL_1242020, EPI_ISL_1242021, EPI_ISL_1242022, EPI_ISL_1242023, EPI_ISL_1242024, EPI_ISL_1242025, EPI_ISL_1242026, EPI_ISL_1242027, EPI_ISL_1242028			
see above	Nigeria Centre for Disease Control (NCDC)	African Centre of Excellence for Genomics of Infectious Diseases (ACEGID), Redeemer's University	Oluniyi P.E. et al
EPI_ISL_455362, EPI_ISL_455412, EPI_ISL_455413, EPI_ISL_455414, EPI_ISL_455415, EPI_ISL_455418, EPI_ISL_455419, EPI_ISL_455423, EPI_ISL_455424, EPI_ISL_455425, EPI_ISL_455427, EPI_ISL_455428, EPI_ISL_455429, EPI_ISL_455430, EPI_ISL_455431, EPI_ISL_487087, EPI_ISL_487089, EPI_ISL_487090, EPI_ISL_487091, EPI_ISL_487092, EPI_ISL_487095, EPI_ISL_487096, EPI_ISL_487098, EPI_ISL_487099, EPI_ISL_487101, EPI_ISL_487102, EPI_ISL_487103, EPI_ISL_487104, EPI_ISL_487105, EPI_ISL_487106, EPI_ISL_487107, EPI_ISL_487108, EPI_ISL_487109, EPI_ISL_487110, EPI_ISL_487111, EPI_ISL_487112, EPI_ISL_527872, EPI_ISL_527874, EPI_ISL_527875, EPI_ISL_527876, EPI_ISL_527877, EPI_ISL_527878, EPI_ISL_527879, EPI_ISL_527880, EPI_ISL_527881, EPI_ISL_527882, EPI_ISL_527883, EPI_ISL_527884, EPI_ISL_527885, EPI_ISL_527886, EPI_ISL_527887, EPI_ISL_527888, EPI_ISL_527889, EPI_ISL_527890, EPI_ISL_527891, EPI_ISL_527892, EPI_ISL_527893, EPI_ISL_527894, EPI_ISL_527895, EPI_ISL_527896, EPI_ISL_527897, EPI_ISL_527898, EPI_ISL_527899, EPI_ISL_527900, EPI_ISL_527901, EPI_ISL_527902, EPI_ISL_527903, EPI_ISL_527904, EPI_ISL_527905, EPI_ISL_527906, EPI_ISL_527907, EPI_ISL_527908, EPI_ISL_527909, EPI_ISL_527910, EPI_ISL_527911, EPI_ISL_527912, EPI_ISL_527913, EPI_ISL_527914, EPI_ISL_527915, EPI_ISL_729920, EPI_ISL_729921, EPI_ISL_729922, EPI_ISL_729923, EPI_ISL_729924, EPI_ISL_729925, EPI_ISL_729926, EPI_ISL_729927, EPI_ISL_729928, EPI_ISL_729929, EPI_ISL_729930, EPI_ISL_729931, EPI_ISL_729932, EPI_ISL_729933, EPI_ISL_729934, EPI_ISL_729935, EPI_ISL_729936, EPI_ISL_729937, EPI_ISL_729938, EPI_ISL_729939, EPI_ISL_729940, EPI_ISL_729941, EPI_ISL_729942, EPI_ISL_729943, EPI_ISL_729944, EPI_ISL_729945, EPI_ISL_729946, EPI_ISL_729947, EPI_ISL_729948, EPI_ISL_729949, EPI_ISL_729950, EPI_ISL_729951, EPI_ISL_729952, EPI_ISL_729953, EPI_ISL_729954, EPI_ISL_729955, EPI_ISL_729956, EPI_ISL_729957, EPI_ISL_729958, EPI_ISL_729959, EPI_ISL_729960, EPI_ISL_729961, EPI_ISL_729962, EPI_ISL_729963, EPI_ISL_729964, EPI_ISL_729965, EPI_ISL_729966, EPI_ISL_729967, EPI_ISL_729968, EPI_ISL_729969, EPI_ISL_729970, EPI_ISL_729971, EPI_ISL_729972, EPI_ISL_729973, EPI_ISL_729974, EPI_ISL_729975, EPI_ISL_729976, EPI_ISL_729977, EPI_ISL_729978, EPI_ISL_729979, EPI_ISL_729980, EPI_ISL_729981, EPI_ISL_729982, EPI_ISL_729983, EPI_ISL_729984, EPI_ISL_729985, EPI_ISL_729986, EPI_ISL_729987, EPI_ISL_729988, EPI_ISL_729989, EPI_ISL_729990, EPI_ISL_729991, EPI_ISL_729992, EPI_ISL_729993, EPI_ISL_729994, EPI_ISL_729995, EPI_ISL_729996, EPI_ISL_729997, EPI_ISL_729998, EPI_ISL_729999, EPI_ISL_730000, EPI_ISL_730001, EPI_ISL_730002, EPI_ISL_730003, EPI_ISL_730004, EPI_ISL_730005, EPI_ISL_730006, EPI_ISL_730007, EPI_ISL_730008, EPI_ISL_730009, EPI_ISL_730010, EPI_ISL_730011, EPI_ISL_730012, EPI_ISL_730013, EPI_ISL_730014, EPI_ISL_730015, EPI_ISL_730016, EPI_ISL_730017, EPI_ISL_730018, EPI_ISL_730019, EPI_ISL_730020, EPI_ISL_730021, EPI_ISL_730022, EPI_ISL_730023, EPI_ISL_730024, EPI_ISL_730025, EPI_ISL_730026, EPI_ISL_730027, EPI_ISL_730028, EPI_ISL_730029, EPI_ISL_730030, EPI_ISL_730031, EPI_ISL_730032, EPI_ISL_730033, EPI_ISL_730034, EPI_ISL_730035, EPI_ISL_730036, EPI_ISL_730037, EPI_ISL_730038, EPI_ISL_730039, EPI_ISL_730040, EPI_ISL_730041, EPI_ISL_730042, EPI_ISL_730043, EPI_ISL_730044, EPI_ISL_730045, EPI_ISL_730046, EPI_ISL_730047, EPI_ISL_730048			
see above	Nigeria Centre for Disease Control (NCDC)	African Centre of Excellence for Genomics of Infectious Diseases (ACEGID), Redeemer's University, Ede, Osun State, Nigeria	Ajogbasile F.V.; Folarin O.A.; Happi C.T.; Ihekweazu C.; Kayode A.; Oguzie J.; Olawoye I.; Olumade T.; Oluniyi P.E.; Oluniyi P.E. et al; Uwanibe J.
EPI_ISL_487113	Nigeria Centre for Disease Control (NCDC)	Redeemer's University, ACEGID	Ajogbasile F.V.; Folarin O.A.; Happi C.T.; Ihekweazu C.; Kayode A.; Oguzie J.; Olawoye I.; Olumade T.; Oluniyi P.E.; Uwanibe J.
EPI_ISL_766049	Nigeria Centre for Disease Control (NCDC), National Reference Laboratory (NRL)	National Reference Laboratory, NCDC, Abuja	Chimaobi Chukwu; Dr Chikwe Ihekweazu; Dr Ndodo Nnaemeka; Dr Omoare Adesuyi; Mrs Nwando Mba; Olusola Akanbi
EPI_ISL_977540, EPI_ISL_977541, EPI_ISL_977542, EPI_ISL_977543, EPI_ISL_977544, EPI_ISL_977545, EPI_ISL_977546, EPI_ISL_977547, EPI_ISL_977548, EPI_ISL_977549, EPI_ISL_977550, EPI_ISL_977551, EPI_ISL_977553, EPI_ISL_977554, EPI_ISL_977555, EPI_ISL_977556, EPI_ISL_977557, EPI_ISL_977558, EPI_ISL_977559, EPI_ISL_977560, EPI_ISL_977561, EPI_ISL_977562, EPI_ISL_977564, EPI_ISL_977565			
see above	Nigeria Centre of Disease Control (NCDC)	African Centre of Excellence for Genomics of Infectious Diseases (ACEGID), Redeemer's University	Olawoye I. B. et al
EPI_ISL_1093431, EPI_ISL_1093432, EPI_ISL_1093433, EPI_ISL_1093434, EPI_ISL_1093435, EPI_ISL_1093436, EPI_ISL_1093437, EPI_ISL_1093438, EPI_ISL_1093439, EPI_ISL_1093440, EPI_ISL_1093441, EPI_ISL_1093442, EPI_ISL_1093443, EPI_ISL_1093444, EPI_ISL_1093445, EPI_ISL_1093446, EPI_ISL_1093447, EPI_ISL_1093448, EPI_ISL_1093449, EPI_ISL_1093450, EPI_ISL_1093451, EPI_ISL_1093452, EPI_ISL_1093453, EPI_ISL_1093454, EPI_ISL_1093455, EPI_ISL_1093456, EPI_ISL_1093457, EPI_ISL_1093458, EPI_ISL_1093459, EPI_ISL_1093460, EPI_ISL_1093461, EPI_ISL_1093462, EPI_ISL_1093463, EPI_ISL_1093464, EPI_ISL_1093466, EPI_ISL_1093467, EPI_ISL_1093468, EPI_ISL_1093469, EPI_ISL_1093470, EPI_ISL_1093471, EPI_ISL_1093472, EPI_ISL_1093473, EPI_ISL_1093474, EPI_ISL_1093475, EPI_ISL_1093476, EPI_ISL_1235642, EPI_ISL_1235643, EPI_ISL_1235644, EPI_ISL_1235645, EPI_ISL_1235646, EPI_ISL_1235647, EPI_ISL_1235648, EPI_ISL_1235649, EPI_ISL_1235650, EPI_ISL_1235651, EPI_ISL_1235652, EPI_ISL_1235653, EPI_ISL_1235654, EPI_ISL_1235655, EPI_ISL_1235656, EPI_ISL_1235657, EPI_ISL_1235658, EPI_ISL_1235659, EPI_ISL_1235660, EPI_ISL_1235661, EPI_ISL_1235662, EPI_ISL_1235663, EPI_ISL_1235664, EPI_ISL_1235665, EPI_ISL_1235666, EPI_ISL_1235667			
see above	Nigerian Centre for Disease Control (NCDC)	African Centre of Excellence for Genomics of Infectious Diseases (ACEGID), Redeemer's University	I.B.; Olawoye; et al; et.al
EPI_ISL_1035810, EPI_ISL_1035811, EPI_ISL_1035812, EPI_ISL_1035813, EPI_ISL_1035814, EPI_ISL_1035815, EPI_ISL_1035816, EPI_ISL_1035817, EPI_ISL_1035818, EPI_ISL_1035819, EPI_ISL_1035820, EPI_ISL_1035821, EPI_ISL_1035822, EPI_ISL_1035823, EPI_ISL_1035824, EPI_ISL_1035825, EPI_ISL_1035826, EPI_ISL_1035827			
see above	Nigerian Centre for Disease Control (NCDC)	African Centre of Excellence for Genomics of Infectious Diseases (ACEGID), Redeemer's University, Ede	I.B.; Olawoye; et al
EPI_ISL_450507, EPI_ISL_462992	Nigerian Institute of Medical Research	Nigerian Institute of Medical Research	A.B.; A.O.; A.P.; Abosede, O.; Adegbola, R.; Adesegun, A.; Ahmed; Ahmed, R.; Amoo; B.L. and Audu; C.K.; F.A.; Ige; J.O.; James; L.C.; Liboro, G.; O.B.; O.S.; Odewale, E.; Okoli; Okwuraiwe; Omilabu; Onwuamah; Oyefolu; Oyefolu, A.; R.A.; S. and Audu, R.; S.A.; Saibu; Salako; Salako, B.; Salu; Shaibu; Sokei, J.
EPI_ISL_745142, EPI_ISL_745143	Nomangesi Jayiya Clinic	National Health Laboratory Service (NHLS), Tygerberg	Bronwyn Kleinhans; Eduan Wilkindon; Gert van Zyl; Houriiyah Tegally; Kayla Delaney; Susan Engelbrecht; Tulio de Oliveira; Wolfgang Preiser
EPI_ISL_735438	Nuclei Acid Testing - Rwanda National Reference Laboratory	GIGA Medical Genomics	Bouchra Boujemla; Esperence Umumararungu; Jacob Souopgui; Keith Durkin; Léon Mutesa; Maria Artesi; Marie-Pierre Hayette; Patrick Tuyisenge; Robert Rutayisire; Sabin Nsanzimana; Swaibu Gatare; Sébastien Bontems; Vincent Bours; Yvan Butera
EPI_ISL_735436, EPI_ISL_735437, EPI_ISL_735444, EPI_ISL_735446, EPI_ISL_735447, EPI_ISL_735448	Nucleic Acid Testing - Rwanda National Reference Laboratory	GIGA Medical Genomics	Bouchra Boujemla; Esperence Umumararungu; Jacob Souopgui; Keith Durkin; Léon Mutesa; Maria Artesi; Marie-Pierre Hayette; Patrick Tuyisenge; Robert Rutayisire; Sabin Nsanzimana; Swaibu Gatare; Sébastien Bontems; Vincent Bours; Yvan Butera
EPI_ISL_925847, EPI_ISL_925848, EPI_ISL_925849, EPI_ISL_925850, EPI_ISL_925851, EPI_ISL_925852, EPI_ISL_925853, EPI_ISL_925854, EPI_ISL_925855, EPI_ISL_925856, EPI_ISL_925857, EPI_ISL_925858, EPI_ISL_925859, EPI_ISL_925860, EPI_ISL_925861, EPI_ISL_925862, EPI_ISL_925863, EPI_ISL_925864, EPI_ISL_925865, EPI_ISL_925866, EPI_ISL_925867, EPI_ISL_925868, EPI_ISL_925869, EPI_ISL_925870, EPI_ISL_925871, EPI_ISL_925872, EPI_ISL_925873, EPI_ISL_925874, EPI_ISL_925875, EPI_ISL_925876, EPI_ISL_925877, EPI_ISL_925878, EPI_ISL_925879, EPI_ISL_925880, EPI_ISL_925881, EPI_ISL_925882, EPI_ISL_925883, EPI_ISL_925884, EPI_ISL_925885, EPI_ISL_925886, EPI_ISL_925887, EPI_ISL_925888, EPI_ISL_925889, EPI_ISL_925890, EPI_ISL_925891, EPI_ISL_925892, EPI_ISL_925893, EPI_ISL_925894, EPI_ISL_925895, EPI_ISL_925896, EPI_ISL_925897, EPI_ISL_925898, EPI_ISL_925899, EPI_ISL_925900, EPI_ISL_925901, EPI_ISL_925902, EPI_ISL_925903, EPI_ISL_925904, EPI_ISL_925905, EPI_ISL_925906, EPI_ISL_925907, EPI_ISL_925908, EPI_ISL_925909, EPI_ISL_925910, EPI_ISL_925911, EPI_ISL_925912, EPI_ISL_925913, EPI_ISL_925914, EPI_ISL_925915, EPI_ISL_930567, EPI_ISL_930634, EPI_ISL_930853, EPI_ISL_960227, EPI_ISL_960228, EPI_ISL_960229, EPI_ISL_960230, EPI_ISL_960231, EPI_ISL_960232, EPI_ISL_960233, EPI_ISL_960234, EPI_ISL_960235, EPI_ISL_960236, EPI_ISL_960237, EPI_ISL_960238, EPI_ISL_960239, EPI_ISL_960240, EPI_ISL_960241, EPI_ISL_960242, EPI_ISL_960243, EPI_ISL_960244, EPI_ISL_960245, EPI_ISL_960246, EPI_ISL_960247, EPI_ISL_960248, EPI_ISL_960249, EPI_ISL_960250, EPI_ISL_960251, EPI_ISL_960252, EPI_ISL_960253, EPI_ISL_960254, EPI_ISL_960255, EPI_ISL_960256, EPI_ISL_960257, EPI_ISL_960258, EPI_ISL_960259, EPI_ISL_960260, EPI_ISL_960261, EPI_ISL_960262, EPI_ISL_960263, EPI_ISL_960264, EPI_ISL_960265, EPI_ISL_960266, EPI_ISL_960267, EPI_ISL_960268, EPI_ISL_960269, EPI_ISL_960270, EPI_ISL_960271, EPI_ISL_960272, EPI_ISL_960273, EPI_ISL_960274, EPI_ISL_960275, EPI_ISL_960276, EPI_ISL_960277, EPI_ISL_960278, EPI_ISL_960279, EPI_ISL_960280, EPI_ISL_960281, EPI_ISL_960282, EPI_ISL_960283, EPI_ISL_960284, EPI_ISL_960285, EPI_ISL_960286, EPI_ISL_960287, EPI_ISL_960288, EPI_ISL_960289, EPI_ISL_960290, EPI_ISL_960291, EPI_ISL_960292, EPI_ISL_960293, EPI_ISL_960294, EPI_ISL_960295, EPI_ISL_960296, EPI_ISL_960297, EPI_ISL_960298, EPI_ISL_960299, EPI_ISL_960300, EPI_ISL_960301, EPI_ISL_960302, EPI_ISL_1020288			
see above	Nucleic Acid Testing, National Reference Laboratory	GIGA Medical Genomics	Bouchra Boujemla; Corinne Fasquelle; Esperence Umumararungu; Jacob Souopgui; Keith Durkin; Léon Mutesa; Maria Artesi; Marie-Pierre Hayette; Nathalie Renotte; Patrick Tuyisenge; Robert Rutayisire; Sabin Nsanzimana; Swaibu Gatare; Sébastien Bontems; Vincent Bours; Yvan Butera
EPI_ISL_640105, EPI_ISL_700471, EPI_ISL_700472, EPI_ISL_700486, EPI_ISL_700511, EPI_ISL_700523, EPI_ISL_700580			
see above	Nyanga CDC wc NGC	NHLS/UCT	Arash Iranzadeh; Bruna Galvao; Carolyn Williamson; Deelan Doolabh; Diana Hardie; Innocent Mudau; Kruger Marais; Lynn Tyers; Marvin Hsiao; Stephen Korsman
EPI_ISL_1074743, EPI_ISL_1076023, EPI_ISL_1076306	Nyangabwe Hospital HIV Reference Laboratory	Botswana Harvard HIV Reference Laboratory	Boitumelo Zuze; Botshelo Radibe; David Lawrence; Dorcas Maruapula; Joseph; Joseph Makhema; Legodile Kooepile; Makhema; Mosepele Mosepele; Roger Shapiro; Shahin Lockman; Sikhulile Moyo; Simani Gaseitsiwe; Wonderful T. Choga
EPI_ISL_1074301	Nyangabwe Hospital HIV Reference Laboratory	Botswana Harvard HIV Reference Laboratory,	Boitumelo Zuze; Botshelo Radibe; David Lawrence; Dorcas Maruapula; Joseph Makhema; Mosepele Mosepele; Roger Shapiro; Shahin Lockman; Sikhulile Moyo; Simani Gaseitsiwe; Wonderful T. Choga
EPI_ISL_745126	Okiep CHC	National Health Laboratory Service (NHLS), Tygerberg	Bronwyn Kleinhans; Eduan Wilkindon; Gert van Zyl; Houriiyah Tegally; Kayla Delaney; Susan Engelbrecht; Tulio de Oliveira; Wolfgang Preiser
EPI_ISL_640017, EPI_ISL_640023, EPI_ISL_640077, EPI_ISL_700417, EPI_ISL_700447, EPI_ISL_700575, EPI_ISL_700594			
see above	Oudtshoorn Hospital wc OUD	NHLS/UCT	Arash Iranzadeh; Bruna Galvao; Carolyn Williamson; Deelan Doolabh; Diana Hardie; Innocent Mudau; Kruger Marais; Lynn Tyers; Marvin Hsiao; Stephen Korsman
EPI_ISL_960144, EPI_ISL_960146, EPI_ISL_960147	Oudtshoorn Hospital wc OUD	National Health Laboratory Service/UCT	Arash Iranzadeh; Bruna Galvao; Carolyn Williamson; Deelan Doolabh; Diana Hardie; Innocent Mudau; Kruger Marais; Lynn Tyers; Marvin Hsiao; Stephen Korsman
EPI_ISL_1035923, EPI_ISL_1035931, EPI_ISL_1035932, EPI_ISL_1035933, EPI_ISL_1035934, EPI_ISL_1035935, EPI_ISL_1035936, EPI_ISL_1035937, EPI_ISL_1035938, EPI_ISL_1035939, EPI_ISL_1035940, EPI_ISL_1035941, EPI_ISL_1035942, EPI_ISL_1035943, EPI_ISL_1035944, EPI_ISL_1035945, EPI_ISL_1035946, EPI_ISL_1035947, EPI_ISL_1035948, EPI_ISL_1035959			
see above	Outre Mer CH de Mayotte	National Reference Center for Viruses of Respiratory Infections, Institut Pasteur, Paris	Angela Brisebarre; Camille Capel; Combe Patrice; Etienne Simon-Lorière; Marion Barbet; Maud Vanpeene; Méline Bizard; Sylvie Behillil; Sylvie van der Werf; Vincent Enouf
EPI_ISL_995331, EPI_ISL_995332, EPI_ISL_995333, EPI_ISL_995334, EPI_ISL_995335, EPI_ISL_995336, EPI_ISL_995337, EPI_ISL_995338, EPI_ISL_995339, EPI_ISL_995340, EPI_ISL_995341, EPI_ISL_995342, EPI_ISL_995343, EPI_ISL_995344, EPI_ISL_995345, EPI_ISL_995346, EPI_ISL_995347, EPI_ISL_995348, EPI_ISL_995349, EPI_ISL_995350, EPI_ISL_995351, EPI_ISL_995352, EPI_ISL_995353, EPI_ISL_995354, EPI_ISL_995355, EPI_ISL_995356, EPI_ISL_995357, EPI_ISL_995358, EPI_ISL_995359, EPI_ISL_995360, EPI_ISL_995361, EPI_ISL_995362, EPI_ISL_995363, EPI_ISL_995364, EPI_ISL_995365, EPI_ISL_995366, EPI_ISL_995367, EPI_ISL_995368, EPI_ISL_995369, EPI_ISL_995370, EPI_ISL_995371, EPI_ISL_995372, EPI_ISL_995373, EPI_ISL_995374, EPI_ISL_995375, EPI_ISL_995376, EPI_ISL_995377, EPI_ISL_995378, EPI_ISL_995379, EPI_ISL_995380, EPI_ISL_995381, EPI_ISL_995382, EPI_ISL_995383, EPI_ISL_995384, EPI_ISL_995385, EPI_ISL_995386, EPI_ISL_995387, EPI_ISL_995388, EPI_ISL_995389, EPI_ISL_995390, EPI_ISL_995391, EPI_ISL_995392, EPI_ISL_995393, EPI_ISL_995394, EPI_ISL_995395, EPI_ISL_995396, EPI_ISL_995397, EPI_ISL_995398, EPI_ISL_995399, EPI_ISL_995400, EPI_ISL_995401, EPI_ISL_995402, EPI_ISL_995403, EPI_ISL_995404, EPI_ISL_995405, EPI_ISL_995406, EPI_ISL_995407, EPI_ISL_995408, EPI_ISL_995409, EPI_ISL_995410, EPI_ISL_995411, EPI_ISL_995412, EPI_ISL_995413, EPI_ISL_995414, EPI_ISL_995415, EPI_ISL_995416, EPI_ISL_995417, EPI_ISL_995418, EPI_ISL_995419, EPI_ISL_995420, EPI_ISL_995421, EPI_ISL_995422, EPI_ISL_995423, EPI_ISL_995424, EPI_ISL_995425, EPI_ISL_995426, EPI_ISL_995427, EPI_ISL_995428, EPI_ISL_995429, EPI_ISL_995430, EPI_ISL_995431, EPI_ISL_995432, EPI_ISL_995433, EPI_ISL_995434, EPI_ISL_995435, EPI_ISL_995436, EPI_ISL_995437, EPI_ISL_995438, EPI_ISL_995439, EPI_ISL_995440, EPI_ISL_995441, EPI_ISL_995442, EPI_ISL_995443, EPI_ISL_995444, EPI_ISL_995445, EPI_ISL_1201511, EPI_ISL_1201512, EPI_ISL_1201513, EPI_ISL_1201514, EPI_ISL_1201515, EPI_ISL_1201516, EPI_ISL_1201517, EPI_ISL_1201518, EPI_ISL_1201519			
see above	Outre mer	National Reference Center for Viruses of Respiratory Infections, Institut Pasteur, Paris	Angela Brisebarre; Camille Capel; Combe Patrice; Etienne Simon-Lorière; Marion Barbet; Maud Vanpeene; Méline Bizard; Sylvie Behillil; Sylvie van der Werf; Vincent Enouf
EPI_ISL_700435, EPI_ISL_700439, EPI_ISL_700449, EPI_ISL_700451, EPI_ISL_700452, EPI_ISL_700478, EPI_ISL_700551, EPI_ISL_700586, EPI_ISL_1040655, EPI_ISL_1040679, EPI_ISL_1040799			
see above	Pacaltsdorp Clinic wc PAC	NHLS/UCT	Arash Iranzadeh; Bruna Galvao; Carolyn Williamson; Deelan Doolabh; Diana Hardie; Houriiyah Tegally; Innocent Mudau; Kruger Marais; Lynn Tyers; Marvin Hsiao; Stephen Korsman
EPI_ISL_696453, EPI_ISL_696456, EPI_ISL_696459, EPI_ISL_696470, EPI_ISL_696474, EPI_ISL_696504, EPI_ISL_696513			
see above	Pacaltsdorp Clinic wc PAC & NHLS/UCT	KRISP, KZN Research Innovation and Sequencing Platform	Arash Iranzadeh; Bruna Galvao; Carolyn Williamson; Deelan Doolabh; Diana Hardie; Emanuel James San; Houriiyah Tegally; Innocent Mudau; Jennifer Giandhari; Kruger Marais; Lynn Tyers; Marvin Hsiao; Stephen Korsman; Sureshnee Pillay; Tulio de Oliveira
EPI_ISL_1064198, EPI_ISL_1075350	Palapye Primary Hospital Laboratory	Botswana Harvard HIV Reference Laboratory	Boitumelo Zuze; Botshelo Radibe; David Lawrence; Dorcas Maruapula; Joseph; Joseph Makhema; Legodile Kooepile; Makhema; Mosepele Mosepele; Roger Shapiro; Shahin Lockman; Sikhulile Moyo; Simani Gaseitsiwe; Wonderful T. Choga
EPI_ISL_660143, EPI_ISL_660144, EPI_ISL_660145, EPI_ISL_660146, EPI_ISL_660147, EPI_ISL_660148, EPI_ISL_660149, EPI_ISL_660150, EPI_ISL_660151, EPI_ISL_660152, EPI_ISL_660153, EPI_ISL_660154, EPI_ISL_660155, EPI_ISL_660156, EPI_ISL_660157, EPI_ISL_660158, EPI_ISL_912401			
see above	PathCare	National Health Laboratory Service (NHLS), Tygerberg	Bronwyn Kleinhans; Davis M-A; Draper C; Eduan Wilkindon; Gert van Zyl; Houriiyah Tegally; Hsiao M; Jean Maritz; Kayla Delaney; Siegfried N; Susan Engelbrecht; Tulio de Oliveira; Williamson C; Wolfgang Preiser
EPI_ISL_1209000,	Pathcare	National Institute for Communicable Diseases of the National Health Laboratory Service	Amoako DG; Bhiman JN; Ismail A; Konstantinus I; Mahlangu B; Mohale T; Ntuli N; Scheepers C; Van Rooyen G

EPI_ISL_1209001, EPI_ISL_1209002, EPI_ISL_1209003			
EPI_ISL_640042	Pelican Park CDC wc PAX	NHLS/UCT	Arash Iranzadeh; Bruna Galvao; Carolyn Williamson; Deelan Doolabh; Diana Hardie; Innocent Mudau; Kruger Marais; Lynn Tyers; Marvin Hsiao; Stephen Korsman
EPI_ISL_960111	Pelican Park CDC wc PAX	National Health Laboratory Service/UCT	Arash Iranzadeh; Bruna Galvao; Carolyn Williamson; Deelan Doolabh; Diana Hardie; Innocent Mudau; Kruger Marais; Lynn Tyers; Marvin Hsiao; Stephen Korsman
EPI_ISL_522547, EPI_ISL_522548	Platforme CYROI	UMR PIMIT Université de La Réunion	Camille Lebarbenchon; David Wilkinson; Patrick Mavingui
EPI_ISL_700430, EPI_ISL_700434, EPI_ISL_700500, EPI_ISL_700518, EPI_ISL_700528, EPI_ISL_700560	Plettenberg Bay Clinic wc PLC	NHLS/UCT	Arash Iranzadeh; Bruna Galvao; Carolyn Williamson; Deelan Doolabh; Diana Hardie; Houriiyah Tegally; Innocent Mudau; Kruger Marais; Lynn Tyers; Marvin Hsiao; Stephen Korsman
EPI_ISL_960115	Plettenberg Bay Clinic wc PLC	National Health Laboratory Service/UCT	Arash Iranzadeh; Bruna Galvao; Carolyn Williamson; Deelan Doolabh; Diana Hardie; Innocent Mudau; Kruger Marais; Lynn Tyers; Marvin Hsiao; Stephen Korsman
EPI_ISL_696454, EPI_ISL_696476	Plettenberg Bay Clinic wc PLC & NHLS/UCT	KRISP, KZN Research Innovation and Sequencing Platform	Arash Iranzadeh; Bruna Galvao; Carolyn Williamson; Deelan Doolabh; Diana Hardie; Emanuel James San; Houriiyah Tegally; Innocent Mudau; Jennifer Giandhari; Kruger Marais; Lynn Tyers; Marvin Hsiao; Stephen Korsman; Sureshnee Pillay; Tulio de Oliveira
EPI_ISL_712070, EPI_ISL_712071, EPI_ISL_712072, EPI_ISL_712073, EPI_ISL_712074, EPI_ISL_712075, EPI_ISL_712076, EPI_ISL_712079, EPI_ISL_712080, EPI_ISL_712081, EPI_ISL_712082, EPI_ISL_712083, EPI_ISL_712084, EPI_ISL_712085, EPI_ISL_712086, EPI_ISL_712087, EPI_ISL_712088, EPI_ISL_712089, EPI_ISL_712090, EPI_ISL_712091, EPI_ISL_712093, EPI_ISL_712094, EPI_ISL_712095, EPI_ISL_712096, EPI_ISL_723468			
see above	Port Elizabeth Provincial Hospital, National Health Laboratory Services, Eastern Cape, South Africa	National Institute for Communicable Diseases of the National Health Laboratory Service	Allam M; Bhiman JN; Ismail A; Mahlangu B; Mohale T; Ntuli N
EPI_ISL_745129, EPI_ISL_745187	Port Nolloth Hospital	National Health Laboratory Service (NHLS), Tygerberg	Bronwyn Kleinhans; Eduan Wilkindon; Gert van Zyl; Houriiyah Tegally; Kayla Delaney; Susan Engelbrecht; Tulio de Oliveira; Wolfgang Preiser
EPI_ISL_1196005, EPI_ISL_1196019	RSP Clinic	National Institute for Communicable Diseases of the National Health Laboratory Service	Amoako DG; Bhiman JN; Ismail A; Mahlangu B; Maphalala GP; Mohale T; Ntuli N; Scheepers C
EPI_ISL_640086, EPI_ISL_640136, EPI_ISL_1040742	Red Cross Children's Hospital wc RXH	NHLS/UCT	Arash Iranzadeh; Bruna Galvao; Carolyn Williamson; Deelan Doolabh; Diana Hardie; Innocent Mudau; Kruger Marais; Lynn Tyers; Marvin Hsiao; Stephen Korsman
EPI_ISL_960106	Red Cross Children's Hospital wc RXH	National Health Laboratory Service/UCT	Arash Iranzadeh; Bruna Galvao; Carolyn Williamson; Deelan Doolabh; Diana Hardie; Innocent Mudau; Kruger Marais; Lynn Tyers; Marvin Hsiao; Stephen Korsman
EPI_ISL_467299	Research and Medical Analysis Laboratory of Gendarmerie Royale	Research and Medical Analysis Laboratory of Gendarmerie Royale	Sanaâ LEMRISS Amal SOUIRI Hicham EL OSSMANI Saâd EL Kabbaj
EPI_ISL_1040778	Retreat CHC wc RHC	NHLS/UCT	Arash Iranzadeh; Bruna Galvao; Carolyn Williamson; Deelan Doolabh; Diana Hardie; Innocent Mudau; Kruger Marais; Lynn Tyers; Marvin Hsiao; Stephen Korsman
EPI_ISL_640035, EPI_ISL_700465	Riversdale Clinic wc RAV	NHLS/UCT	Arash Iranzadeh; Bruna Galvao; Carolyn Williamson; Deelan Doolabh; Diana Hardie; Innocent Mudau; Kruger Marais; Lynn Tyers; Marvin Hsiao; Stephen Korsman
EPI_ISL_1239479, EPI_ISL_1239484, EPI_ISL_1239487, EPI_ISL_1239490, EPI_ISL_1239493, EPI_ISL_1239502, EPI_ISL_1239509, EPI_ISL_1239515, EPI_ISL_1239524, EPI_ISL_1239528, EPI_ISL_1239530			
see above	Rob Ferreira Hospital, National Health Laboratory Services, Mpumalanga, South Africa	National Institute for Communicable Diseases of the National Health Laboratory Service	Amoako DG; Bhiman JN; Ismail A; Mahlangu B; Mohale T; Ntuli N; Scheepers C
EPI_ISL_735445	Rwanda National Reference Laboratory	GIGA Medical Genomics	Bouchra Boujemla; Esperence Umumararungu; Jacob Souopgui; Keith Durkin; Léon Mutesa; Maria Artesi; Marie-Pierre Hayette; Patrick Tuyisenge; Robert Rutayisire; Sabin Nsanzimana; Swaibu Gatare; Sébastien Bontems; Vincent Bours; Yvan Butera
EPI_ISL_615069, EPI_ISL_1064171	Rwanda National Reference Laboratory	Rwanda National Laboratory	Aine O'Toole; Enatha Mukantwari; Mukantwari Enatha; Stefan Rooke; UMuringa Jeanne d'Arc; Umuringa Jeanne d'Arc
EPI_ISL_614763, EPI_ISL_614891, EPI_ISL_614892, EPI_ISL_614980, EPI_ISL_615063, EPI_ISL_615064, EPI_ISL_615066, EPI_ISL_615067, EPI_ISL_615071, EPI_ISL_615074, EPI_ISL_615075, EPI_ISL_707711, EPI_ISL_707712, EPI_ISL_707713, EPI_ISL_707771, EPI_ISL_707772, EPI_ISL_707773, EPI_ISL_707774, EPI_ISL_707776, EPI_ISL_707777, EPI_ISL_707779, EPI_ISL_707780, EPI_ISL_707783, EPI_ISL_707787, EPI_ISL_707788, EPI_ISL_707789, EPI_ISL_707790, EPI_ISL_1063900, EPI_ISL_1063901, EPI_ISL_1063905, EPI_ISL_1063915, EPI_ISL_1063994, EPI_ISL_1064022, EPI_ISL_1064147, EPI_ISL_1064148, EPI_ISL_1064149, EPI_ISL_1064152, EPI_ISL_1064153, EPI_ISL_1064154, EPI_ISL_1064163, EPI_ISL_1064164, EPI_ISL_1064165, EPI_ISL_1064166, EPI_ISL_1064168, EPI_ISL_1064170			
see above	Rwanda National Reference Laboratory	Rwanda National Reference Laboratory	Aine O'Toole; Aine O'Toole; ENatha MUkantwari; ENatha Mukantwari; Enatha MUkantwari; Enatha Mukantwari; Jeanne d'Arc; Jeanne d'Arc Mukantwari; Jeanne d'Arc UMuringa; Jeanne d'Arc Umuringa; MUkanntwari Eanatha; MUkantwari Enatha; Mukantwari Enatha; Stefan Rook; Stefan Rooke; UMuringa Jeanne d'Arc; Umuringa Jeanne d'Arc; Umurunga Jeanne d'Arc
EPI_ISL_745156	SAS Saldanha	National Health Laboratory Service (NHLS), Tygerberg	Bronwyn Kleinhans; Eduan Wilkindon; Gert van Zyl; Houriiyah Tegally; Kayla Delaney; Susan Engelbrecht; Tulio de Oliveira; Wolfgang Preiser
EPI_ISL_745157, EPI_ISL_745161	SAS Saldanha VPA	National Health Laboratory Service (NHLS), Tygerberg	Bronwyn Kleinhans; Eduan Wilkindon; Gert van Zyl; Houriiyah Tegally; Kayla Delaney; Susan Engelbrecht; Tulio de Oliveira; Wolfgang Preiser
EPI_ISL_510529	School of Veterinary Medicine, Disease Control	School of Veterinary Medicine, Disease Control	A.L.; A.N.; Bates, M.; C. and Zumla, A.; Chambaro, H.; Chanda, D.; Changula, K.; Chilufya; Chipimo; Chitanga, S.; Fwoloshi, S.; K.S.; Kapata; Kapata, N.; Kapaya, F.; Kapin'a, M.; Kayeyi, N.; Liwewe; M.M.; Malama, K.; Masahiro, K.; Monze, M.; Morales; Mubemba, B.; Mukonka, V.; Mulenga, L.; Muleya, W.; Mupeta, F.; Musonda, K.; Nalubamba; Ngosa, W.; P.C.; P.J.; Saasa, N.; Sawa, H.; Shibemba; Simulundu, E.; Sinyange, N.; Takada, A.; Tembo, J.; Zulu, P.
EPI_ISL_2649891, EPI_ISL_2649892, EPI_ISL_2649893, EPI_ISL_2649894, EPI_ISL_2649895, EPI_ISL_2649896, EPI_ISL_2649897, EPI_ISL_2649898, EPI_ISL_2649899, EPI_ISL_2649900			
see above	Scientific Department, Division of Molecular Biology	Scientific Department, Division of Molecular Biology	A.B.; A.M.; Alaruusi; Alaruusi, A.; Annajr, B.; Bashein, A.; Belkhir; Bitrou; Bitrou, S.; D'Amore, N.; De Sanctis, R.; De Santis, R.; Elahmer, O.; Faggioni, G.; Fillo, S.; Giordani, F.; Hamdani; Hamdani, T.; Jumaa; K.A.; Lista, F.; Mad, W.; Madi; Monte, A.; Palomba, S.; S.M.; T.N.; Taloa; Taloa, K.; W.Y.; Zorgani, A.
EPI_ISL_640034, EPI_ISL_700588	Sedgefield Clinic wc SGE	NHLS/UCT	Arash Iranzadeh; Bruna Galvao; Carolyn Williamson; Deelan Doolabh; Diana Hardie; Innocent Mudau; Kruger Marais; Lynn Tyers; Marvin Hsiao; Stephen Korsman
EPI_ISL_696462, EPI_ISL_696475, EPI_ISL_696487, EPI_ISL_696488, EPI_ISL_696510, EPI_ISL_696514, EPI_ISL_696515, EPI_ISL_696516, EPI_ISL_696517, EPI_ISL_696518			
see above	Sedgefield Clinic wc SGE & NHLS/UCT	KRISP, KZN Research Innovation and Sequencing Platform	Arash Iranzadeh; Bruna Galvao; Carolyn Williamson; Deelan Doolabh; Diana Hardie; Emanuel James San; Houriiyah Tegally; Innocent Mudau; Jennifer Giandhari; Kruger Marais; Lynn Tyers; Marvin Hsiao; Stephen Korsman; Sureshnee Pillay; Tulio de Oliveira
EPI_ISL_640079	Stellenbosch Hospital wc STB	NHLS/UCT	Arash Iranzadeh; Bruna Galvao; Carolyn Williamson; Deelan Doolabh; Diana Hardie; Innocent Mudau; Kruger Marais; Lynn Tyers; Marvin Hsiao; Stephen Korsman
EPI_ISL_1040647	Swellendam PHC	NHLS/UCT	Arash Iranzadeh; Bruna Galvao; Carolyn Williamson; Deelan Doolabh; Diana Hardie; Innocent Mudau; Kruger Marais; Lynn Tyers; Marvin Hsiao; Stephen Korsman
EPI_ISL_1196006	TLC Clinic	National Institute for Communicable Diseases of the National Health Laboratory Service	Amoako DG; Bhiman JN; Ismail A; Mahlangu B; Maphalala GP; Mohale T; Ntuli N; Scheepers C
EPI_ISL_966940	Technical Support Units for Scientific Research (UATRS), National Centre for Scientific and Technical Research (CNRST)	Technical Support Units for Scientific Research (UATRS), National Centre for Scientific and Technical Research (CNRST)	Alaoui; Elalaoui; Elannaz, H.; Elouanass, M.; Ennibi; H. and El Fahime, E.; Hemlali, M.; Lahlou; M.A.; Melloul, M.; Rfaki, A.; S.A.; Touil, N.; a.l.
EPI_ISL_640019, EPI_ISL_640026, EPI_ISL_700424, EPI_ISL_700425, EPI_ISL_700428, EPI_ISL_700431, EPI_ISL_700438, EPI_ISL_700460, EPI_ISL_700461, EPI_ISL_700482, EPI_ISL_700483, EPI_ISL_700487, EPI_ISL_700490, EPI_ISL_700498, EPI_ISL_700502			
see above	Thembalethu CDC wc THC	NHLS/UCT	Arash Iranzadeh; Bruna Galvao; Carolyn Williamson; Deelan Doolabh; Diana Hardie; Houriiyah Tegally; Innocent Mudau; Kruger Marais; Lynn Tyers; Marvin Hsiao; Stephen Korsman
EPI_ISL_960139	Thembalethu CDC wc THC	National Health Laboratory Service/UCT	Arash Iranzadeh; Bruna Galvao; Carolyn Williamson; Deelan Doolabh; Diana Hardie; Innocent Mudau; Kruger Marais; Lynn Tyers; Marvin Hsiao; Stephen Korsman
EPI_ISL_696521	Thembalethu CDC wc THC & NHLS/UC	KRISP, KZN Research Innovation and Sequencing Platform	Arash Iranzadeh; Bruna Galvao; Carolyn Williamson; Deelan Doolabh; Diana Hardie; Emanuel James San; Houriiyah Tegally; Innocent Mudau; Jennifer Giandhari; Kruger Marais; Lynn Tyers; Marvin Hsiao; Stephen Korsman; Sureshnee Pillay; Tulio de Oliveira
EPI_ISL_696452, EPI_ISL_696455, EPI_ISL_696461, EPI_ISL_696493, EPI_ISL_696494, EPI_ISL_696496, EPI_ISL_696499, EPI_ISL_696500, EPI_ISL_696519, EPI_ISL_696520			
see above	Thembalethu CDC wc THC & NHLS/UCT	KRISP, KZN Research Innovation and Sequencing Platform	Arash Iranzadeh; Bruna Galvao; Carolyn Williamson; Deelan Doolabh; Diana Hardie; Emanuel James San; Houriiyah Tegally; Innocent Mudau; Jennifer Giandhari; Kruger Marais; Lynn Tyers; Marvin Hsiao; Stephen Korsman; Sureshnee Pillay; Tulio de Oliveira
EPI_ISL_696485, EPI_ISL_696490	Touwsranten Clinic wc TST & NHLS/UCT	KRISP, KZN Research Innovation and Sequencing Platform	Arash Iranzadeh; Bruna Galvao; Carolyn Williamson; Deelan Doolabh; Diana Hardie; Emanuel James San; Houriiyah Tegally; Innocent Mudau; Jennifer Giandhari; Kruger Marais; Lynn Tyers; Marvin Hsiao; Stephen Korsman; Sureshnee Pillay; Tulio de Oliveira
EPI_ISL_1239459, EPI_ISL_1239462, EPI_ISL_1239464, EPI_ISL_1239465, EPI_ISL_1239469, EPI_ISL_1239470, EPI_ISL_1239472, EPI_ISL_1239473, EPI_ISL_1239482, EPI_ISL_1239491, EPI_ISL_1239492, EPI_ISL_1239494, EPI_ISL_1239495, EPI_ISL_1239496, EPI_ISL_1239497, EPI_ISL_1239498, EPI_ISL_1239499, EPI_ISL_1239500, EPI_ISL_1239501, EPI_ISL_1239503, EPI_ISL_1239504, EPI_ISL_1239505, EPI_ISL_1239506, EPI_ISL_1239507, EPI_ISL_1239510, EPI_ISL_1239511, EPI_ISL_1239513, EPI_ISL_1239514, EPI_ISL_1239516, EPI_ISL_1239517, EPI_ISL_1239521, EPI_ISL_1239523, EPI_ISL_1239525, EPI_ISL_1239526			
see above	Tshepong Hospital, National Health Laboratory Services, North West, South Africa	National Institute for Communicable Diseases of the National Health Laboratory Service	Amoako DG; Bhiman JN; Ismail A; Mahlangu B; Mohale T; Ntuli N; Scheepers C
EPI_ISL_960152	Tshwaragano Hospital	National Health Laboratory Service/UCT	Arash Iranzadeh; Bruna Galvao; Carolyn Williamson; Deelan Doolabh; Diana Hardie; Innocent Mudau; Kruger Marais; Lynn Tyers; Marvin Hsiao; Stephen Korsman
EPI_ISL_745140, EPI_ISL_745141, EPI_ISL_745147, EPI_ISL_745148, EPI_ISL_745149, EPI_ISL_745150, EPI_ISL_745180, EPI_ISL_745181, EPI_ISL_745182, EPI_ISL_745183, EPI_ISL_745184, EPI_ISL_745185			
see above	Tygerberg Hospital wc TBH	National Health Laboratory Service (NHLS), Tygerberg	Bronwyn Kleinhans; Eduan Wilkindon; Gert van Zyl; Houriiyah Tegally; Kayla Delaney; Susan Engelbrecht; Tulio de Oliveira; Wolfgang Preiser
EPI_ISL_628750, EPI_ISL_628751, EPI_ISL_628752, EPI_ISL_628753, EPI_ISL_628754, EPI_ISL_628755, EPI_ISL_628756, EPI_ISL_628757, EPI_ISL_628758, EPI_ISL_628759, EPI_ISL_628760, EPI_ISL_628761, EPI_ISL_648124, EPI_ISL_648125, EPI_ISL_648126, EPI_ISL_648127, EPI_ISL_648128, EPI_ISL_861459, EPI_ISL_861460, EPI_ISL_861461, EPI_ISL_861462, EPI_ISL_861463, EPI_ISL_861464, EPI_ISL_861465, EPI_ISL_861466, EPI_ISL_861467, EPI_ISL_861468, EPI_ISL_861469, EPI_ISL_861470, EPI_ISL_861471			
see above	UHAS COVID-19 Lab	UHAS COVID-19 Lab	John O. Gyapong and the UHAS COVID-19 Lab Team; Jones Gyamfi; Kwabena O. Duedu; Reuben Ayivor-Djanie
EPI_ISL_737931, EPI_ISL_737932, EPI_ISL_737933, EPI_ISL_737934, EPI_ISL_737935, EPI_ISL_737936, EPI_ISL_737937, EPI_ISL_737938, EPI_ISL_737939, EPI_ISL_737940, EPI_ISL_737941, EPI_ISL_737942, EPI_ISL_737943, EPI_ISL_737944, EPI_ISL_737945, EPI_ISL_737946, EPI_ISL_737947, EPI_ISL_737948, EPI_ISL_737949, EPI_ISL_737950, EPI_ISL_737951, EPI_ISL_737952, EPI_ISL_737953, EPI_ISL_737954, EPI_ISL_737955, EPI_ISL_737956, EPI_ISL_737957, EPI_ISL_737958, EPI_ISL_737959, EPI_ISL_737960, EPI_ISL_737961, EPI_ISL_737962, EPI_ISL_737963, EPI_ISL_737964, EPI_ISL_737965, EPI_ISL_737966, EPI_ISL_737967, EPI_ISL_737968, EPI_ISL_737969, EPI_ISL_737970, EPI_ISL_737971, EPI_ISL_737972, EPI_ISL_737973, EPI_ISL_737974, EPI_ISL_737975, EPI_ISL_737976, EPI_ISL_737977, EPI_ISL_737978, EPI_ISL_737979, EPI_ISL_737980, EPI_ISL_737981, EPI_ISL_737982, EPI_ISL_737983, EPI_ISL_737985, EPI_ISL_737986, EPI_ISL_737987, EPI_ISL_737988, EPI_ISL_737989, EPI_ISL_737990, EPI_ISL_737991, EPI_ISL_737992, EPI_ISL_737993, EPI_ISL_737994, EPI_ISL_737995, EPI_ISL_737997, EPI_ISL_737998, EPI_ISL_737999, EPI_ISL_738000, EPI_ISL_738001, EPI_ISL_738002, EPI_ISL_738003, EPI_ISL_738004, EPI_ISL_738005, EPI_ISL_738006, EPI_ISL_738007, EPI_ISL_738008, EPI_ISL_738009, EPI_ISL_738010, EPI_ISL_738011, EPI_ISL_738012, EPI_ISL_738013, EPI_ISL_738014, EPI_ISL_738015, EPI_ISL_738016, EPI_ISL_738017, EPI_ISL_738018, EPI_ISL_738019, EPI_ISL_738020, EPI_ISL_738021, EPI_ISL_738022, EPI_ISL_738023, EPI_ISL_738025, EPI_ISL_738026, EPI_ISL_738027, EPI_ISL_738028, EPI_ISL_738029, EPI_ISL_738030, EPI_ISL_738031, EPI_ISL_738032, EPI_ISL_738033, EPI_ISL_738034, EPI_ISL_738035, EPI_ISL_738036, EPI_ISL_738037, EPI_ISL_738038, EPI_ISL_738039, EPI_ISL_738040, EPI_ISL_738041, EPI_ISL_738042, EPI_ISL_738043			

see above	Uganda Central Public Health Lab and Uganda Virus Research Institute	MRC/UVRI & LSHTM Uganda Research Unit	Dan Lule Bugembe; Matthew Cotten; My V.T. Phan; Pontiano Kaleebu et al.
EPI_ISL_451183, EPI_ISL_451184, EPI_ISL_451185, EPI_ISL_451186, EPI_ISL_451187, EPI_ISL_451188, EPI_ISL_451189, EPI_ISL_451190, EPI_ISL_451191, EPI_ISL_451192, EPI_ISL_451193, EPI_ISL_451194, EPI_ISL_451195, EPI_ISL_451196, EPI_ISL_451197, EPI_ISL_451198, EPI_ISL_451199, EPI_ISL_451200, EPI_ISL_451201, EPI_ISL_451202			
see above	Uganda Virus Research Institute	MRC/UVRI & LSHTM Uganda Research Unit	Beatrice Dhaala; Dan Lule Bugembe; Deogratius Ssemwanga; Henry Kyobe; Henry Mwebesa; Jane Aceng; John Kayiwa; Jonas Lexow; Julius Lutwama; Matthew Cotten; My V.T Phan; Phionah Tushabe; Pontiano Kaleebu; Stephen Balinandi
EPI_ISL_812966, EPI_ISL_812967	University Clinical Research Center, University of Sciences	University Clinical Research Center, University of Sciences	A.A.; Bane, S.; Dao, S.; Diakite, M.; Diarra, B.; Doumbia, S.; Guindo, I.; Iknane; Kone, A.
EPI_ISL_1208967, EPI_ISL_1208968, EPI_ISL_1209011, EPI_ISL_1209012	University of Namibia (UNAM LAB)	National Institute for Communicable Diseases of the National Health Laboratory Service	Amoako DG; Bhiman JN; Ismail A; Konstantinus I; Mahlangu B; Mohale T; Ntuli N; Scheepers C; Van Rooyen G
EPI_ISL_977251, EPI_ISL_977252, EPI_ISL_977253, EPI_ISL_977254, EPI_ISL_977255, EPI_ISL_977256, EPI_ISL_977257, EPI_ISL_977258, EPI_ISL_977259, EPI_ISL_977260, EPI_ISL_977261, EPI_ISL_977262, EPI_ISL_977263, EPI_ISL_977264, EPI_ISL_977265, EPI_ISL_977266, EPI_ISL_977267, EPI_ISL_977268, EPI_ISL_977269, EPI_ISL_977270, EPI_ISL_977271, EPI_ISL_977272, EPI_ISL_977273, EPI_ISL_977274, EPI_ISL_977275, EPI_ISL_977276, EPI_ISL_977277, EPI_ISL_977278, EPI_ISL_977279, EPI_ISL_977280, EPI_ISL_977281, EPI_ISL_977282, EPI_ISL_977283, EPI_ISL_977284, EPI_ISL_977285, EPI_ISL_977286, EPI_ISL_977287, EPI_ISL_977288, EPI_ISL_977289, EPI_ISL_977290, EPI_ISL_977291, EPI_ISL_977292, EPI_ISL_977293, EPI_ISL_977294, EPI_ISL_977295, EPI_ISL_977296, EPI_ISL_977297, EPI_ISL_977298, EPI_ISL_977299, EPI_ISL_977300, EPI_ISL_977301, EPI_ISL_977302, EPI_ISL_977303, EPI_ISL_977304, EPI_ISL_977305, EPI_ISL_977306, EPI_ISL_977307, EPI_ISL_977308, EPI_ISL_977309, EPI_ISL_977310, EPI_ISL_977311, EPI_ISL_977312, EPI_ISL_977313, EPI_ISL_977314, EPI_ISL_977315, EPI_ISL_977316, EPI_ISL_977317, EPI_ISL_977318, EPI_ISL_977319, EPI_ISL_977320, EPI_ISL_977321, EPI_ISL_977322, EPI_ISL_977323, EPI_ISL_977324, EPI_ISL_977325, EPI_ISL_977326, EPI_ISL_977327, EPI_ISL_977328, EPI_ISL_977329, EPI_ISL_977330, EPI_ISL_977331, EPI_ISL_977332, EPI_ISL_977333, EPI_ISL_977334, EPI_ISL_977335, EPI_ISL_977336, EPI_ISL_977337, EPI_ISL_977338, EPI_ISL_977339, EPI_ISL_977340, EPI_ISL_977341, EPI_ISL_977342, EPI_ISL_977343, EPI_ISL_977344, EPI_ISL_977345, EPI_ISL_977346, EPI_ISL_977347, EPI_ISL_977348, EPI_ISL_977349, EPI_ISL_977350, EPI_ISL_977351, EPI_ISL_977352, EPI_ISL_977353, EPI_ISL_977354, EPI_ISL_977355, EPI_ISL_977356, EPI_ISL_977357, EPI_ISL_977358, EPI_ISL_977359, EPI_ISL_977360, EPI_ISL_977361, EPI_ISL_977362, EPI_ISL_977363, EPI_ISL_977364, EPI_ISL_977365, EPI_ISL_977366, EPI_ISL_977367, EPI_ISL_977368, EPI_ISL_977369, EPI_ISL_977370, EPI_ISL_977371, EPI_ISL_977372, EPI_ISL_977373, EPI_ISL_977374, EPI_ISL_977375, EPI_ISL_977376, EPI_ISL_977377, EPI_ISL_977378, EPI_ISL_977379, EPI_ISL_977380, EPI_ISL_977381, EPI_ISL_977382, EPI_ISL_977383, EPI_ISL_977384, EPI_ISL_977385, EPI_ISL_977386, EPI_ISL_977387, EPI_ISL_977388, EPI_ISL_977389, EPI_ISL_977390, EPI_ISL_977391, EPI_ISL_977392, EPI_ISL_977393, EPI_ISL_977394, EPI_ISL_977395, EPI_ISL_977396, EPI_ISL_977397, EPI_ISL_977398, EPI_ISL_977399, EPI_ISL_977400, EPI_ISL_977401, EPI_ISL_977402, EPI_ISL_977403, EPI_ISL_977404, EPI_ISL_977405, EPI_ISL_977406, EPI_ISL_977407, EPI_ISL_977408, EPI_ISL_977409, EPI_ISL_977410, EPI_ISL_977411, EPI_ISL_977412, EPI_ISL_977413, EPI_ISL_977414, EPI_ISL_977415, EPI_ISL_977416, EPI_ISL_977418, EPI_ISL_977419, EPI_ISL_977420, EPI_ISL_977421, EPI_ISL_977422, EPI_ISL_977423, EPI_ISL_977424, EPI_ISL_977425, EPI_ISL_977426, EPI_ISL_977427, EPI_ISL_977428, EPI_ISL_977429, EPI_ISL_977430, EPI_ISL_977431, EPI_ISL_977432, EPI_ISL_977433, EPI_ISL_977434, EPI_ISL_977435, EPI_ISL_977436, EPI_ISL_977437, EPI_ISL_977438, EPI_ISL_977439, EPI_ISL_977440, EPI_ISL_977441, EPI_ISL_977442, EPI_ISL_977443, EPI_ISL_977444, EPI_ISL_977445, EPI_ISL_977446, EPI_ISL_977447, EPI_ISL_977448, EPI_ISL_977449, EPI_ISL_977450, EPI_ISL_977451, EPI_ISL_977452, EPI_ISL_977453, EPI_ISL_977454, EPI_ISL_977455, EPI_ISL_977456, EPI_ISL_977457, EPI_ISL_977458, EPI_ISL_977459, EPI_ISL_977460, EPI_ISL_977461, EPI_ISL_977462, EPI_ISL_977463, EPI_ISL_977464, EPI_ISL_977465, EPI_ISL_977466, EPI_ISL_977467, EPI_ISL_977468, EPI_ISL_977469, EPI_ISL_977470			
see above	University of Zambia, School of Veterinary Medicine	UNZAVET and PATH	Daniel Bridges; Mulenga Mwenda-Chimfwembe; Ngonda Saasa
EPI_ISL_856695	University of Zambia, School of Veterinary Medicine, Disease Control	University of Zambia, School of Veterinary Medicine, Disease Control	A.L.; A.N.; Bates, M.; C. and Zumla, A.; Chambaro, H.; Chanda, D.; Changula, K.; Chilufya; Chipimo; Chitanga, S.; Fwoloshi, S.; K.S.; Kapata; Kapata, N.; Kapaya, F.; Kapin'a, M.; Kayeyi, N.; Liwewe; M.M.; Malama, K.; Masahiro, K.; Monze, M.; Morales; Mubemba, B.; Mukonka, V.; Mulenga, L.; Muleya, W.; Mupeta, F.; Musonda, K.; Nalubamba; Ngosa, W.; P.C.; P.J.; Saasa, N.; Sawa, H.; Shibemba; Simulundu, E.; Sinyange, N.; Takada, A.; Tembo, J.; Zulu, P.
EPI_ISL_940850, EPI_ISL_940851, EPI_ISL_940852, EPI_ISL_940853, EPI_ISL_940854, EPI_ISL_940855, EPI_ISL_940856, EPI_ISL_940857, EPI_ISL_940858, EPI_ISL_940859, EPI_ISL_940861, EPI_ISL_940862, EPI_ISL_940863, EPI_ISL_940864, EPI_ISL_940865, EPI_ISL_940866, EPI_ISL_940867, EPI_ISL_940868, EPI_ISL_940869, EPI_ISL_940870, EPI_ISL_940871, EPI_ISL_940872, EPI_ISL_940873, EPI_ISL_940874, EPI_ISL_940875, EPI_ISL_940876, EPI_ISL_940877, EPI_ISL_940878, EPI_ISL_940879, EPI_ISL_940880, EPI_ISL_940881, EPI_ISL_940882, EPI_ISL_940883, EPI_ISL_940884, EPI_ISL_940885, EPI_ISL_940886, EPI_ISL_940887, EPI_ISL_940888, EPI_ISL_940889, EPI_ISL_940890, EPI_ISL_940891			
see above	Vaccines and Infectious Diseases Analytics Research Unit (VIDA)	KRISP, KZN Research Innovation and Sequencing Platform	Baillie Vicky; Giandhari Jennifer; Madhi Shabir; Naidoo Yeshnee; Pillay Sureshnee; Tegally Houriiyah; de Oliveira Tulio; du Plessis Jeanine
EPI_ISL_1132669, EPI_ISL_1132670, EPI_ISL_1132671, EPI_ISL_1132672, EPI_ISL_1132673, EPI_ISL_1132674, EPI_ISL_1132675, EPI_ISL_1132676, EPI_ISL_1132677, EPI_ISL_1132678, EPI_ISL_1132679, EPI_ISL_1132680, EPI_ISL_1132681, EPI_ISL_1132682, EPI_ISL_1132683, EPI_ISL_1132684, EPI_ISL_1132685, EPI_ISL_1132686, EPI_ISL_1132687, EPI_ISL_1132688, EPI_ISL_1132689, EPI_ISL_1132690, EPI_ISL_1132691, EPI_ISL_1132692, EPI_ISL_1132693, EPI_ISL_1132694, EPI_ISL_1132695, EPI_ISL_1132696, EPI_ISL_1132697, EPI_ISL_1132698, EPI_ISL_1132699, EPI_ISL_1132700, EPI_ISL_1132701, EPI_ISL_1132702, EPI_ISL_1132703, EPI_ISL_1132704, EPI_ISL_1132705, EPI_ISL_1132706, EPI_ISL_1132707, EPI_ISL_1132708, EPI_ISL_1132709, EPI_ISL_1132710, EPI_ISL_1132711, EPI_ISL_1132712, EPI_ISL_1132713, EPI_ISL_1132714, EPI_ISL_1132715, EPI_ISL_1132716, EPI_ISL_1132717, EPI_ISL_1132718, EPI_ISL_1132719, EPI_ISL_1132720, EPI_ISL_1132721, EPI_ISL_1132722, EPI_ISL_1132723, EPI_ISL_1132724, EPI_ISL_1132725, EPI_ISL_1132726, EPI_ISL_1132727, EPI_ISL_1132728			
see above	Vaccines and Infectious Diseases Analytics Research Unit (VIDA)	KRISP, KZn Research Innovation and Sequencing Platform	Baillie Vicky; Giandhari Jennifer; Madhi Shabir; Naidoo Yeshnee; Pillay Sureshnee; Tegally Houriiyah; de Oliveira Tulio; du Plessis Jeanine
EPI_ISL_640037, EPI_ISL_640046, EPI_ISL_640114	Valkenberg Hospital wc VBH	NHLS/UCT	Arash Iranzadeh; Bruna Galvao; Carolyn Williamson; Deelan Doolabh; Diana Hardie; Innocent Mudau; Kruger Marais; Lynn Tyers; Marvin Hsiao; Stephen Korsman
EPI_ISL_640072, EPI_ISL_640081, EPI_ISL_640096, EPI_ISL_640097, EPI_ISL_640098, EPI_ISL_640099, EPI_ISL_640102, EPI_ISL_700413, EPI_ISL_700462, EPI_ISL_700527, EPI_ISL_1040677			
see above	Vanguard CHC wc VGC	NHLS/UCT	Arash Iranzadeh; Bruna Galvao; Carolyn Williamson; Deelan Doolabh; Diana Hardie; Innocent Mudau; Kruger Marais; Lynn Tyers; Marvin Hsiao; Stephen Korsman
EPI_ISL_960159	Vanguard CHC wc VGC	National Health Laboratory Service/UCT	Arash Iranzadeh; Bruna Galvao; Carolyn Williamson; Deelan Doolabh; Diana Hardie; Innocent Mudau; Kruger Marais; Lynn Tyers; Marvin Hsiao; Stephen Korsman
EPI_ISL_640021, EPI_ISL_640032, EPI_ISL_640085, EPI_ISL_640087, EPI_ISL_640108, EPI_ISL_640109, EPI_ISL_640110, EPI_ISL_640113, EPI_ISL_640126, EPI_ISL_640141, EPI_ISL_700421, EPI_ISL_700546, EPI_ISL_700596, EPI_ISL_1040746, EPI_ISL_1040774, EPI_ISL_1040776, EPI_ISL_1040784, EPI_ISL_1040788, EPI_ISL_1040814			
see above	Victoria Hospital wc VHW	NHLS/UCT	Arash Iranzadeh; Bruna Galvao; Carolyn Williamson; Deelan Doolabh; Diana Hardie; Innocent Mudau; Kruger Marais; Lynn Tyers; Marvin Hsiao; Stephen Korsman
EPI_ISL_960094, EPI_ISL_960099, EPI_ISL_960101, EPI_ISL_960108, EPI_ISL_960113, EPI_ISL_960114, EPI_ISL_960131, EPI_ISL_960138, EPI_ISL_960150			
see above	Victoria Hospital wc VHW	National Health Laboratory Service/UCT	Arash Iranzadeh; Bruna Galvao; Carolyn Williamson; Deelan Doolabh; Diana Hardie; Innocent Mudau; Kruger Marais; Lynn Tyers; Marvin Hsiao; Stephen Korsman
EPI_ISL_414647, EPI_ISL_417433, EPI_ISL_417434, EPI_ISL_417435, EPI_ISL_417436, EPI_ISL_417437, EPI_ISL_417438, EPI_ISL_417439, EPI_ISL_417440, EPI_ISL_417441, EPI_ISL_417442, EPI_ISL_417941, EPI_ISL_417942, EPI_ISL_417944, EPI_ISL_417946, EPI_ISL_417947, EPI_ISL_417948, EPI_ISL_417950, EPI_ISL_417955, EPI_ISL_420030, EPI_ISL_420031, EPI_ISL_420032, EPI_ISL_420033, EPI_ISL_420034, EPI_ISL_420035, EPI_ISL_420838, EPI_ISL_420839, EPI_ISL_420840, EPI_ISL_420841, EPI_ISL_420842, EPI_ISL_420843, EPI_ISL_420844, EPI_ISL_420845, EPI_ISL_420846, EPI_ISL_420847, EPI_ISL_420848, EPI_ISL_420849, EPI_ISL_420850, EPI_ISL_420851, EPI_ISL_420852, EPI_ISL_420853, EPI_ISL_420854, EPI_ISL_429254, EPI_ISL_429255, EPI_ISL_429258, EPI_ISL_429259, EPI_ISL_431011, EPI_ISL_431012, EPI_ISL_434678, EPI_ISL_434679, EPI_ISL_434680, EPI_ISL_434681, EPI_ISL_434710, EPI_ISL_434711, EPI_ISL_435032, EPI_ISL_435033, EPI_ISL_435113, EPI_ISL_435114, EPI_ISL_435116, EPI_ISL_435117, EPI_ISL_435118, EPI_ISL_435156, EPI_ISL_435157, EPI_ISL_435158, EPI_ISL_435159, EPI_ISL_435160, EPI_ISL_435161, EPI_ISL_435162, EPI_ISL_435163, EPI_ISL_435164, EPI_ISL_435165, EPI_ISL_435166, EPI_ISL_435167, EPI_ISL_435168, EPI_ISL_436194, EPI_ISL_436412, EPI_ISL_437193, EPI_ISL_437194, EPI_ISL_437195, EPI_ISL_437196, EPI_ISL_437337, EPI_ISL_437338, EPI_ISL_437339, EPI_ISL_437340, EPI_ISL_437341, EPI_ISL_437342, EPI_ISL_437343, EPI_ISL_437344, EPI_ISL_437345, EPI_ISL_437346, EPI_ISL_437347, EPI_ISL_437348, EPI_ISL_437350, EPI_ISL_437351, EPI_ISL_437352, EPI_ISL_437353, EPI_ISL_437354, EPI_ISL_437355, EPI_ISL_437356, EPI_ISL_437357, EPI_ISL_437358, EPI_ISL_447230, EPI_ISL_447231, EPI_ISL_447232, EPI_ISL_447233, EPI_ISL_447234, EPI_ISL_447235, EPI_ISL_447236, EPI_ISL_447237, EPI_ISL_447238, EPI_ISL_447239, EPI_ISL_447240, EPI_ISL_447241, EPI_ISL_447242, EPI_ISL_447243, EPI_ISL_447244, EPI_ISL_447245, EPI_ISL_447246, EPI_ISL_447247, EPI_ISL_447248, EPI_ISL_447249, EPI_ISL_447596, EPI_ISL_447597, EPI_ISL_447598, EPI_ISL_447599, EPI_ISL_447600, EPI_ISL_447601, EPI_ISL_447602, EPI_ISL_447603, EPI_ISL_447604, EPI_ISL_447605, EPI_ISL_447606, EPI_ISL_447607, EPI_ISL_471396, EPI_ISL_471397, EPI_ISL_471398, EPI_ISL_471399, EPI_ISL_471400, EPI_ISL_471401, EPI_ISL_471402, EPI_ISL_471403, EPI_ISL_471404, EPI_ISL_471405, EPI_ISL_471406, EPI_ISL_471407, EPI_ISL_471408, EPI_ISL_471409, EPI_ISL_471410, EPI_ISL_471411, EPI_ISL_471412, EPI_ISL_471413, EPI_ISL_471414, EPI_ISL_471415, EPI_ISL_487365, EPI_ISL_487369, EPI_ISL_495629, EPI_ISL_495630, EPI_ISL_495631, EPI_ISL_495632, EPI_ISL_495633, EPI_ISL_495634, EPI_ISL_495635, EPI_ISL_495636, EPI_ISL_495637, EPI_ISL_495638, EPI_ISL_495639, EPI_ISL_495640, EPI_ISL_495641, EPI_ISL_496491, EPI_ISL_496492, EPI_ISL_496493, EPI_ISL_496494, EPI_ISL_496495, EPI_ISL_496496, EPI_ISL_496497, EPI_ISL_496498, EPI_ISL_496499, EPI_ISL_496500, EPI_ISL_496501, EPI_ISL_496502, EPI_ISL_496503, EPI_ISL_496504, EPI_ISL_496505, EPI_ISL_496506, EPI_ISL_496507, EPI_ISL_496508, EPI_ISL_496509, EPI_ISL_496510, EPI_ISL_496511, EPI_ISL_496512, EPI_ISL_496513, EPI_ISL_496514, EPI_ISL_496515, EPI_ISL_496516, EPI_ISL_496517, EPI_ISL_513589, EPI_ISL_513590, EPI_ISL_513591, EPI_ISL_513592, EPI_ISL_513593, EPI_ISL_513594, EPI_ISL_513595, EPI_ISL_513596, EPI_ISL_513597, EPI_ISL_513598, EPI_ISL_513599, EPI_ISL_513600, EPI_ISL_513601, EPI_ISL_513602, EPI_ISL_513603, EPI_ISL_513604, EPI_ISL_513605, EPI_ISL_513606, EPI_ISL_513607, EPI_ISL_513608, EPI_ISL_513609, EPI_ISL_513610, EPI_ISL_513611, EPI_ISL_513612, EPI_ISL_513613, EPI_ISL_513614, EPI_ISL_513615, EPI_ISL_513616, EPI_ISL_513617, EPI_ISL_513618, EPI_ISL_513619, EPI_ISL_513620, EPI_ISL_513621, EPI_ISL_513622, EPI_ISL_513623, EPI_ISL_513624, EPI_ISL_513625, EPI_ISL_513626, EPI_ISL_513627, EPI_ISL_513628, EPI_ISL_513629, EPI_ISL_513630, EPI_ISL_513631, EPI_ISL_514103, EPI_ISL_514104, EPI_ISL_514105, EPI_ISL_514106, EPI_ISL_514107, EPI_ISL_514108, EPI_ISL_514109, EPI_ISL_514110, EPI_ISL_514111, EPI_ISL_514112, EPI_ISL_514113, EPI_ISL_514114, EPI_ISL_514115, EPI_ISL_514116, EPI_ISL_514117, EPI_ISL_514118, EPI_ISL_514119, EPI_ISL_514120, EPI_ISL_514121, EPI_ISL_514122, EPI_ISL_514123, EPI_ISL_514124, EPI_ISL_514125, EPI_ISL_527489, EPI_ISL_527490, EPI_ISL_527491, EPI_ISL_527492, EPI_ISL_527493, EPI_ISL_527494, EPI_ISL_527495, EPI_ISL_527496, EPI_ISL_527497, EPI_ISL_527498, EPI_ISL_527499, EPI_ISL_527500, EPI_ISL_527501, EPI_ISL_527502, EPI_ISL_527503, EPI_ISL_527504, EPI_ISL_527505, EPI_ISL_527506, EPI_ISL_527507, EPI_ISL_527508, EPI_ISL_527509, EPI_ISL_527510, EPI_ISL_527511, EPI_ISL_527512, EPI_ISL_527513, EPI_ISL_527514, EPI_ISL_527515, EPI_ISL_527516, EPI_ISL_527517, EPI_ISL_527518, EPI_ISL_527519, EPI_ISL_527520, EPI_ISL_527521, EPI_ISL_527522, EPI_ISL_527523, EPI_ISL_527524, EPI_ISL_527525, EPI_ISL_527526, EPI_ISL_527527, EPI_ISL_527528, EPI_ISL_527529, EPI_ISL_527530, EPI_ISL_527531, EPI_ISL_527532, EPI_ISL_527533, EPI_ISL_527534, EPI_ISL_527535, EPI_ISL_527536, EPI_ISL_527537, EPI_ISL_527538, EPI_ISL_527539, EPI_ISL_527540, EPI_ISL_527541, EPI_ISL_527542, EPI_ISL_527543, EPI_ISL_527544, EPI_ISL_527545, EPI_ISL_527546, EPI_ISL_527547, EPI_ISL_527548, EPI_ISL_527549, EPI_ISL_527550, EPI_ISL_527551, EPI_ISL_527552, EPI_ISL_527553, EPI_ISL_527554, EPI_ISL_527555, EPI_ISL_527556, EPI_ISL_527557, EPI_ISL_527558, EPI_ISL_527559, EPI_ISL_527560, EPI_ISL_527561, EPI_ISL_527562, EPI_ISL_527563, EPI_ISL_527564, EPI_ISL_527565, EPI_ISL_527566, EPI_ISL_527567, EPI_ISL_527568, EPI_ISL_527569, EPI_ISL_527570, EPI_ISL_527571, EPI_ISL_527572, EPI_ISL_591083, EPI_ISL_591084, EPI_ISL_591085, EPI_ISL_591086, EPI_ISL_591087, EPI_ISL_591088, EPI_ISL_591089, EPI_ISL_961010, EPI_ISL_961011, EPI_ISL_961012, EPI_ISL_961013, EPI_ISL_961014, EPI_ISL_961015, EPI_ISL_961016, EPI_ISL_961759, EPI_ISL_965122, EPI_ISL_965123, EPI_ISL_965124			
see above	Viral Respiratory Lab, National Institute for Biomedical Research (INRB)	Pathogen Sequencing Lab, National Institute for Biomedical Research (INRB)	Adrienne Amuri Aziza; Allison Black; Amuri Aziza; Andrew Rambault; Andrew Rambaut; Catherine Pratt; Eddy Kinganda-Lusamaki; Edith Nkwembe; Emmanuel Lokilo Lofiko; Emmanuel Lokilo-Lofiko; Francisca Muyembe Mawete; Francisca Muyembe-Mawete; Ian Goodfellow; James Hadfield; Jean Claude Makangara; Jean-Claude Makangara Cigolo; Jean-Jacques Muyembe Tamfum; Josh Quick; Kristian Andersen; Matthias Pauthner; Michael Wiley; Nick Loman; Placide Mbala-Kingebeni; Steve Ahuka-Mundeke; Trevor Bedford
EPI_ISL_487192	Viral Respiratory Lab, National institute for Biomedical Research (INRB)	Pathogen Sequencing Lab, National Institute for Biomedical Research (INRB)	Allison Black; Amuri Aziza; Andrew Rambault; Catherine Pratt; Eddy Kinganda-Lusamaki; Edith Nkwembe; Emmanuel Lokilo-Lofiko; Francisca Muyembe-Mawete; Ian Goodfellow; James Hadfield; Jean-Jacques Muyembe Tamfum.; Josh Quick; Kristian Andersen; Matthias Pauthner; Michael Wiley; Nick Loman; Placide Mbala-Kingebeni; Steve Ahuka-Mundeke; Trevor Bedford
EPI_ISL_528386	Viral vaccines, VSVRI- Veterinary serum and vaccine research institute	Viral vaccines, VSVRI- Veterinary serum and vaccine research institute	A.A.; M.A.; Saad; Saleh
EPI_ISL_1191597, EPI_ISL_1191598, EPI_ISL_1191599, EPI_ISL_1191600, EPI_ISL_1191601, EPI_ISL_1191602, EPI_ISL_1191603, EPI_ISL_1191604, EPI_ISL_1191605, EPI_ISL_1191606, EPI_ISL_1191607, EPI_ISL_1191608			
see above	Virology Department, Victoria Hospital, Plaine-Wilhems, Mauritius	National Institute for Communicable Diseases of the National Health Laboratory Service	Amoako DG; Baboo SB; Bhiman JN; Ismail A; Mahlangu B; Manraj SS; Mohale T; Ntuli N; Ramuth M; Scheepers C; Sonoo J
EPI_ISL_636982, EPI_ISL_636983, EPI_ISL_636984, EPI_ISL_636985, EPI_ISL_636986, EPI_ISL_636987, EPI_ISL_636988			
see above	Virology Lab, National Institute for Biomedical Research (INRB)	Project group Epidemiology of Highly Pathogenic Microorganisms, Robert Koch-Institute	Eddy Kinganda-Lusamaki; Essia Belarbi; Fabian Leendertz; Gabriel Mbunsu; Grit Schubert; Jasmin Schlotterbeck; Jean-Jacques Muyembe Tamfum; Sheila Makiala; Steve Ahuka-Mundeke
EPI_ISL_1123270, EPI_ISL_1123271, EPI_ISL_1123272, EPI_ISL_1123273, EPI_ISL_1123274, EPI_ISL_1123275, EPI_ISL_1123276, EPI_ISL_1123277, EPI_ISL_1123278, EPI_ISL_1123279, EPI_ISL_1123280, EPI_ISL_1123281, EPI_ISL_1123282			
see above	Virology Laboratory of Praia	Institut Pasteur de Dakar	Dia Ndongo; Diagne Moussa Moïse; Diallo Amadou; Diop Mamadou; Faye Ousmane; Kevin Sanders; Loucoubar Cheikh; Mbengue Safietou Sankhe; Ndiaye Ndack; Sall Amadou Alpha; Tordo Noel
EPI_ISL_1123283, EPI_ISL_1123284, EPI_ISL_1123285, EPI_ISL_1123286, EPI_ISL_1123287	Virology Laboratory of São Vicente	Institut Pasteur de Dakar	Dia Ndongo; Diagne Moussa Moïse; Diallo Amadou; Diop Mamadou; Faye Ousmane; Kevin Sanders; Loucoubar Cheikh; Mbengue Safietou Sankhe; Nadia Rodrigues; Ndiaye Ndack; Sall Amadou Alpha; Tordo Noel
EPI_ISL_1123288	Virology Laboratory of São Vicente	Instituto Nacional de Saúde Pública (INSP)	Dia Ndongo; Diagne Moussa Moïse; Diallo Amadou; Diop Mamadou; Faye Ousmane; Kevin Sanders; Loucoubar Cheikh; Mbengue Safietou Sankhe; Nadia Rodrigues; Ndiaye Ndack; Sall Amadou Alpha; Tordo Noel
EPI_ISL_508862, EPI_ISL_508863, EPI_ISL_625456, EPI_ISL_677634, EPI_ISL_677635, EPI_ISL_677636	Virology Unit, Institut Pasteur de Madagascar	Virology Unit, Institut Pasteur de Madagascar	Cara Brook; Cara E. Brook; Christian Ranaivoson; Cristina M. Tato; Helisoa Razafimanjato; Jean-Michel Heraud; Joseph L. DeRisi; Michelle Tan; Norosoa Razanajatovo; Philippe Dussart; Soa Fy Andriamandimby; Tsiry Randriambolamanantsoa; Vida Ahyong; Vololoniaina Raharinosy
EPI_ISL_745117, EPI_ISL_745165, EPI_ISL_745167, EPI_ISL_745189	Vredenburg Hospital wc VBG	National Health Laboratory Service (NHLS), Tygerberg	Bronwyn Kleinhans; Eduan Wilkindon; Gert van Zyl; Houriiyah Tegally; Kayla Delaney; Susan Engelbrecht; Tulio de Oliveira; Wolfgang Preiser
EPI_ISL_745146	Vredendal Hospital wc VRE	National Health Laboratory Service (NHLS), Tygerberg	Bronwyn Kleinhans; Eduan Wilkindon; Gert van Zyl; Houriiyah Tegally; Kayla Delaney; Susan Engelbrecht; Tulio de Oliveira; Wolfgang Preiser
EPI_ISL_745186	Wallacedene Clinic wc WAL	National Health Laboratory Service (NHLS), Tygerberg	Bronwyn Kleinhans; Eduan Wilkindon; Gert van Zyl; Houriiyah Tegally; Kayla Delaney; Susan Engelbrecht; Tulio de Oliveira; Wolfgang Preiser

EPI_ISL_1196002	Women correctional	National Institute for Communicable Diseases of the National Health Laboratory Service	Amoako DG; Bhiman JN; Ismail A; Mahlangu B; Maphalala GP; Mohale T; Ntuli N; Scheepers C
EPI_ISL_1040664	Worcester Hospital	NHLS/UCT	Arash Iranzadeh; Bruna Galvao; Carolyn Williamson; Deelan Doolabh; Diana Hardie; Innocent Mudau; Kruger Marais; Lynn Tyers; Marvin Hsiao; Stephen Korsman
EPI_ISL_700416	Zoar Clinic wc ZOA	NHLS/UCT	Arash Iranzadeh; Bruna Galvao; Carolyn Williamson; Deelan Doolabh; Diana Hardie; Innocent Mudau; Kruger Marais; Lynn Tyers; Marvin Hsiao; Stephen Korsman
EPI_ISL_872197, EPI_ISL_872200, EPI_ISL_872202, EPI_ISL_872221, EPI_ISL_872222, EPI_ISL_872223, EPI_ISL_872224, EPI_ISL_872225, EPI_ISL_872226, EPI_ISL_872232, EPI_ISL_872240, EPI_ISL_872241, EPI_ISL_872242, EPI_ISL_872243, EPI_ISL_872244, EPI_ISL_872245, EPI_ISL_872246, EPI_ISL_872247, EPI_ISL_872248, EPI_ISL_872271, EPI_ISL_872272, EPI_ISL_872273, EPI_ISL_872326, EPI_ISL_872327, EPI_ISL_872328, EPI_ISL_872329, EPI_ISL_872330, EPI_ISL_872331, EPI_ISL_872332, EPI_ISL_872333, EPI_ISL_872334, EPI_ISL_872335, EPI_ISL_872336, EPI_ISL_872337, EPI_ISL_872338, EPI_ISL_884006, EPI_ISL_884007			
see above	hopital	National Reference Center for Viruses of Respiratory Infections, Institut Pasteur, Paris	Angela Brisebarre; Camille Capel; Combe Patrice; Etienne Simon-Lorière; Marion Barbet; Maud Vanpeene; Méline Bizard; Sylvie Behillil; Sylvie van der Werf; Vincent Enouf
EPI_ISL_458285, EPI_ISL_458286	unknown	Bundeswehr Institute of Microbiology	Antwerpen; Bestehorn-Willmann; Eckstein, S.; Handrick, S.; M.C.; M.H.; M.S.; Naija, H.; R. and Ben Moussa, M.; Rehn, A.; Stoecker, K.; Walter; Woelfel
EPI_ISL_463001, EPI_ISL_463002, EPI_ISL_463003, EPI_ISL_463004, EPI_ISL_463005, EPI_ISL_463006	unknown	Clinical virology	Fares, W.; Triki, H.
EPI_ISL_640073	unknown	NHLS/UCT	Arash Iranzadeh; Bruna Galvao; Carolyn Williamson; Deelan Doolabh; Diana Hardie; Innocent Mudau; Kruger Marais; Lynn Tyers; Marvin Hsiao; Stephen Korsman
EPI_ISL_450490, EPI_ISL_450491, EPI_ISL_450492, EPI_ISL_450493, EPI_ISL_450494	unknown	National Influenza and other Respiratory Viruses Centre-Tunisia	Abid, S.; Arab Ennigrou, D.; Ben Nasr, M.; Boutiba, I.; Charaa, L.; El Moussi, A.; Ferjeni, A.; Landolsi, I.

We gratefully acknowledge the following Authors from the Originating laboratories responsible for obtaining the specimens, as well as the Submitting laboratories where the genome data were generated and shared via GISAID, on which this research is based.

All Submitters of data may be contacted directly via www.gisaid.org

Authors are sorted alphabetically.

Accession ID	Originating Laboratory	Submitting Laboratory	Authors
EPI_ISL_1534290, EPI_ISL_1534296, EPI_ISL_1534327, EPI_ISL_1534328, EPI_ISL_1534329, EPI_ISL_1534330, EPI_ISL_1534374, EPI_ISL_1534401, EPI_ISL_1534418, EPI_ISL_1534429, EPI_ISL_2140653, EPI_ISL_2140666, EPI_ISL_2140674, EPI_ISL_2375948, EPI_ISL_2621099, EPI_ISL_2621100, EPI_ISL_2621103, EPI_ISL_2802148			
see above	2 Military Hospital wc MAA	NHLS/UCT	Arash Iranzadeh; Bruna Galvao; Carolyn Williamson; Deelan Doolabh; Diana Hardie; Emmanuel SJ; Gert Marais; Innocent Mudau; Kruger Marais; Lynn Tyers; Marvin Hsiao; Stephen Korsman; Tegally H; de Oliveira T
EPI_ISL_2444137, EPI_ISL_2444138, EPI_ISL_2444139, EPI_ISL_2444140, EPI_ISL_2444141, EPI_ISL_2444142, EPI_ISL_2444143, EPI_ISL_2444144, EPI_ISL_2444145, EPI_ISL_2444146, EPI_ISL_2444147, EPI_ISL_2444148, EPI_ISL_2444149, EPI_ISL_2444150, EPI_ISL_2444151, EPI_ISL_2444152			
see above	AAMPATH LABORATORIES	National Institute for Communicable Diseases of the National Health Laboratory Service	Amoako DG; Bhiman JN; Ismail A; Mahlangu B; Mohale T; Ntuli N; Scheepers C
EPI_ISL_2397308, EPI_ISL_2397309, EPI_ISL_2397310, EPI_ISL_2397311, EPI_ISL_2397312	AHRI	KRISP, KZN Research Innovation and Sequencing Platform	Bernstein M; COMMIT-KZN Team; Cele S; Giandhari J; Gosnell BI; Hanekom W; Karim F; Khan K; Lessells RJ; Manickchund N; Mlisana K; Moosa MYS; Msomi N; Pillay S; Ramphal U; San JE; Sigal A; Singh L; Tegally H; Wilkinson E; de Oliveira T
EPI_ISL_2086984, EPI_ISL_2086985, EPI_ISL_2086986, EPI_ISL_2086987, EPI_ISL_2086988, EPI_ISL_2086989, EPI_ISL_2086990, EPI_ISL_2086991, EPI_ISL_2086992, EPI_ISL_2086993, EPI_ISL_2086994, EPI_ISL_2086995, EPI_ISL_2086996, EPI_ISL_2086997, EPI_ISL_2086998, EPI_ISL_2086999, EPI_ISL_2087000, EPI_ISL_2087001			
see above	AMPATH LABORATORIES	National Institute for Communicable Diseases of the National Health Laboratory Service	Amoako DG; Bhiman JN; Ismail A; Mahlangu B; Mohale T; Ntuli N; Scheepers C
EPI_ISL_2617037, EPI_ISL_2617050, EPI_ISL_2617059, EPI_ISL_2617060, EPI_ISL_2617064, EPI_ISL_2617068, EPI_ISL_2617076, EPI_ISL_2617086			
see above	Africa_CDC - Malawi P4 S2	CERI, Centre for Epidemic Response and Innvoation, Stellenbosch University and KRISP, KZN Research Innovation and Sequencing Platform, UKZN.	Auld A; Chilima B; Chiwaula M; Emmanuel SJ; Giandhari J; Kaba M; Kampira E; Kasambara W; Kim L; Lessells R; Maida A; Mvula B; Mwangomba W; Naidoo Y; Panja L; Pillay S; Tegally H; Wadonda N; Wilkinson E; de Oliveira T
EPI_ISL_2617035, EPI_ISL_2617041, EPI_ISL_2617044, EPI_ISL_2617047, EPI_ISL_2617051, EPI_ISL_2617052, EPI_ISL_2617053, EPI_ISL_2617057, EPI_ISL_2617058, EPI_ISL_2617065, EPI_ISL_2617069, EPI_ISL_2617074, EPI_ISL_2617080, EPI_ISL_2617082, EPI_ISL_2617087, EPI_ISL_2617088, EPI_ISL_2617089, EPI_ISL_2617091			
see above	Africa_CDC - Malawi P6 S2	CERI, Centre for Epidemic Response and Innvoation, Stellenbosch University and KRISP, KZN Research Innovation and Sequencing Platform, UKZN.	Auld A; Chilima B; Chiwaula M; Emmanuel SJ; Giandhari J; Kaba M; Kampira E; Kasambara W; Kim L; Lessells R; Maida A; Mvula B; Mwangomba W; Naidoo Y; Panja L; Pillay S; Tegally H; Wadonda N; Wilkinson E; de Oliveira T
EPI_ISL_2272010, EPI_ISL_2272015, EPI_ISL_2272016, EPI_ISL_2272023	Agincourt Clinic	National Institute for Communicable Diseases of the National Health Laboratory Service	Amoako DG; Bhiman JN; Ismail A; Mahlangu B; Mohale T; Ntuli N; Scheepers C
EPI_ISL_1534336	Albertinia Clinic wc AAP	NHLS/UCT	Arash Iranzadeh; Bruna Galvao; Carolyn Williamson; Deelan Doolabh; Diana Hardie; Emmanuel SJ; Innocent Mudau; Kruger Marais; Lynn Tyers; Marvin Hsiao; Stephen Korsman; Tegally H; de Oliveira T
EPI_ISL_1534288, EPI_ISL_1534371, EPI_ISL_1534384, EPI_ISL_1534454, EPI_ISL_2802128	Alma CDC wc AHC	NHLS/UCT	Arash Iranzadeh; Bruna Galvao; Carolyn Williamson; Deelan Doolabh; Diana Hardie; Emmanuel SJ; Gert Marais; Innocent Mudau; Kruger Marais; Lynn Tyers; Marvin Hsiao; Stephen Korsman; Tegally H; de Oliveira T
EPI_ISL_1534405	Amalienstein Clinic wc AMS	NHLS/UCT	Arash Iranzadeh; Bruna Galvao; Carolyn Williamson; Deelan Doolabh; Diana Hardie; Emmanuel SJ; Innocent Mudau; Kruger Marais; Lynn Tyers; Marvin Hsiao; Stephen Korsman; Tegally H; de Oliveira T
EPI_ISL_1706551, EPI_ISL_1706552	Ampath Cape Town	NHLS/UCT	Arash Iranzadeh; Bruna Galvao; Carolyn Williamson; Deelan Doolabh; Diana Hardie; Emmanuel SJ; Innocent Mudau; Jacques Marx; Kruger Marais; Lynn Tyers; Marvin Hsiao; Stephen Korsman; Tegally H; de Oliveira T
EPI_ISL_2771497, EPI_ISL_2771498	Arizona State University	Arizona State University	Adam K. Khan; Efrem S. Lim; LaRinda A. Holland; Peter T. Skidmore; Rabia Maqsood
EPI_ISL_3071135, EPI_ISL_3071136, EPI_ISL_3071137	Armauer Hansen Research Institute	Armauer Hansen Research Institute	Dr. Abebe Genetu Bayih; Dr. Abel Abera Negash; Dr. Adane Mihret; Dr. Alemseged Abdissa; Dr. Andargachew Mulu; Dr. Getachew Tesfaye Beyene; Dr. Markos Markos Abebe; Dr. Tesfaye Gelanew; Mr. Dawit Hailu Alemayehu; Mr. Dessalegn Abeje Tefera; Mr. Fekadu Alemu; Mr. Tamrayehu Seyoum; Mrs. Bethlehem Adnew
EPI_ISL_1315065	Armed Forces Medical Research Laboratories and Blood Bank, Egypt	Department of Microbial Biotechnology, Genetic Engineering Division, National Research Centre	Ahmed Elsayed; Ahmed Taha; Ayman Farghaly; Mohamed Khalifa; Mohamed Shemis; Reham Dawood
EPI_ISL_1315064	Armed Forces Medical Research Laboratories and Blood Bank, Egypt	Department of Microbial Biotechnology, Genetic Engineering Division, National Research Centre,	Ahmed Elsayed; Ahmed Taha; Ayman Farghaly; Mohamed Khalifa; Mohamed Shemis; Reham Dawood
EPI_ISL_2779390, EPI_ISL_2779391, EPI_ISL_2779392, EPI_ISL_2779393, EPI_ISL_2779394, EPI_ISL_2779395, EPI_ISL_2779400, EPI_ISL_2779401, EPI_ISL_2779402, EPI_ISL_2779403, EPI_ISL_2779404, EPI_ISL_2779405, EPI_ISL_2779406, EPI_ISL_2779407, EPI_ISL_2779408, EPI_ISL_2779409, EPI_ISL_2779410, EPI_ISL_2779411, EPI_ISL_2779412, EPI_ISL_2779413, EPI_ISL_2779414, EPI_ISL_2779415, EPI_ISL_2779416, EPI_ISL_2779417, EPI_ISL_2779509, EPI_ISL_2779510, EPI_ISL_2779511, EPI_ISL_2779512, EPI_ISL_2779513, EPI_ISL_2779514, EPI_ISL_2779515, EPI_ISL_2779516, EPI_ISL_2779517, EPI_ISL_2779518, EPI_ISL_2779519, EPI_ISL_2779520, EPI_ISL_2779521			
see above	BATUK	USAMRD-A, Basic Science Laboratory	Alan Lemtudo; Beth Mutai; Brian Andika; Carol Kifude; Clement Masakwe; Eric Muthanje; Esther Omuseni; Faith Sigei; Gathii Kimita; George Awinda; John Waitumbi; Josphat Nyataya; Rachel Githii; Rehema Liyai; Stephen Ochola
EPI_ISL_1628766, EPI_ISL_1628769, EPI_ISL_1628775, EPI_ISL_1628777, EPI_ISL_1628783, EPI_ISL_1628784, EPI_ISL_1628785, EPI_ISL_1628786, EPI_ISL_1628787, EPI_ISL_1628788, EPI_ISL_1628789, EPI_ISL_1628790, EPI_ISL_1628791, EPI_ISL_1628792, EPI_ISL_1628793, EPI_ISL_1628794, EPI_ISL_1628795, EPI_ISL_1628796, EPI_ISL_1628797, EPI_ISL_1628798, EPI_ISL_1628799, EPI_ISL_1628800, EPI_ISL_1628801, EPI_ISL_1628802, EPI_ISL_1629413, EPI_ISL_1629414, EPI_ISL_1629415, EPI_ISL_1629416, EPI_ISL_1629417, EPI_ISL_1629418, EPI_ISL_1629419, EPI_ISL_1629420, EPI_ISL_3153872			
see above	BIO AUSTRAL	UMR PIMIT	Dr Camille Lebarbenchon; Dr David A Wilkinson; Dr Patrick Mavingui; Magali Turpin; Marie-Alice Simbi
EPI_ISL_2970353, EPI_ISL_2970354, EPI_ISL_2970355	Bacterial and Viral Diseases & Biotechnology and Bioinformatics, Armauer Hansen Research Institute	Bacterial and Viral Diseases & Biotechnology and Bioinformatics, Armauer Hansen Research Institute	A.A.; A.G.; Abdissa, A.; Abebe, M.; Adnew, B.; Alemayehu; Alemu, F.; Bayih; Beyene; D.A.; D.H.; G.T.; Gelanew, T.; Mihrete, A.; Mulu, A.; Negash; Seyoum, T.; Tefera
EPI_ISL_1534276, EPI_ISL_1534449, EPI_ISL_2621085	Bongolethu Clinic wc BLC	NHLS/UCT	Arash Iranzadeh; Bruna Galvao; Carolyn Williamson; Deelan Doolabh; Diana Hardie; Emmanuel SJ; Gert Marais; Innocent Mudau; Kruger Marais; Lynn Tyers; Marvin Hsiao; Stephen Korsman; Tegally H; de Oliveira T
EPI_ISL_1516827, EPI_ISL_1516828, EPI_ISL_1516829, EPI_ISL_1516830, EPI_ISL_1516831, EPI_ISL_1516832, EPI_ISL_1516833, EPI_ISL_1516834, EPI_ISL_1516835, EPI_ISL_1516836, EPI_ISL_1516837, EPI_ISL_1516838, EPI_ISL_1516839, EPI_ISL_1516840, EPI_ISL_1516842, EPI_ISL_1516843, EPI_ISL_1516844, EPI_ISL_1516845, EPI_ISL_1516846, EPI_ISL_1516847, EPI_ISL_1516848, EPI_ISL_1516849, EPI_ISL_1516850, EPI_ISL_1516851, EPI_ISL_1516852, EPI_ISL_1516853, EPI_ISL_1516854, EPI_ISL_1516855, EPI_ISL_1516856, EPI_ISL_1516857, EPI_ISL_1516858, EPI_ISL_1516863, EPI_ISL_1516864, EPI_ISL_1516865, EPI_ISL_1516866, EPI_ISL_1516867, EPI_ISL_1516868, EPI_ISL_1516869, EPI_ISL_1516870, EPI_ISL_1516871, EPI_ISL_1516873, EPI_ISL_1516874, EPI_ISL_1516876, EPI_ISL_1516877, EPI_ISL_1516879, EPI_ISL_1516880, EPI_ISL_1516881, EPI_ISL_2233454, EPI_ISL_2233455, EPI_ISL_2233456, EPI_ISL_2233457, EPI_ISL_2233458, EPI_ISL_2233459, EPI_ISL_2233460, EPI_ISL_2233461, EPI_ISL_2233462, EPI_ISL_2233463, EPI_ISL_2233464, EPI_ISL_2233465, EPI_ISL_2233466, EPI_ISL_2372292			
see above	Botswana Harvard HIV Reference Laboratory	Botswana Harvard HIV Reference Laboratory	Boitumelo Zuze; Botshelo Radibe; David Lawrence; Dorcas Maruapula; Joseph Makhema; Keoratile Ntshambiwa; Kgomotso Moruisi; Kwana Lechiile; Legodile Kooepile; Madisa Mine; Mosepele Mosepele; Ontlametse T. Bareng; Pamela Smith-Lawrence; Roger Shapiro; Shahin Lockman; Sikhulile Dorcas Maruapula; Sikhulile Moyo; Sikhulile Wonderful T. Choga; Simani Gaseitsiwe; Thongbotho Mphoyakgosi; Wonderful T. Choga
EPI_ISL_1534363	Bredasdorp Clinic wc BDC	NHLS/UCT	Arash Iranzadeh; Bruna Galvao; Carolyn Williamson; Deelan Doolabh; Diana Hardie; Emmanuel SJ; Innocent Mudau; Kruger Marais; Lynn Tyers; Marvin Hsiao; Stephen Korsman; Tegally H; de Oliveira T
EPI_ISL_1534396	Brooklyn Chest Hospital wc BCH	NHLS/UCT	Arash Iranzadeh; Bruna Galvao; Carolyn Williamson; Deelan Doolabh; Diana Hardie; Emmanuel SJ; Innocent Mudau; Kruger Marais; Lynn Tyers; Marvin Hsiao; Stephen Korsman; Tegally H; de Oliveira T
EPI_ISL_2779387, EPI_ISL_2779396, EPI_ISL_2779397	Busia County Referral Hospital	USAMRD-A, Basic Science Laboratory	Alan Lemtudo; Beth Mutai; Brian Andika; Carol Kifude; Clement Masakwe; Eric Muthanje; Esther Omuseni; Faith Sigei; Gathii Kimita; George Awinda; John Waitumbi; Josphat Nyataya; Rachel Githii; Rehema Liyai; Stephen Ochola
EPI_ISL_2779289, EPI_ISL_2779290, EPI_ISL_2779291	Busia border point	USAMRD-A, Basic Science Laboratory	Alan Lemtudo; Beth Mutai; Brian Andika; Carol Kifude; Clement Masakwe; Eric Muthanje; Esther Omuseni; Faith Sigei; Gathii Kimita; George Awinda; John Waitumbi; Josphat Nyataya; Rachel Githii; Rehema Liyai; Stephen Ochola
EPI_ISL_2955501, EPI_ISL_2955502, EPI_ISL_2955503, EPI_ISL_2955504, EPI_ISL_2955505, EPI_ISL_2955506, EPI_ISL_2955507, EPI_ISL_2955508, EPI_ISL_2955509, EPI_ISL_2955510, EPI_ISL_2955511			
see above	CAPRISA	KRISP, KZn Research Innovation and Sequencing Platform	Emmanuel SJ; Giandhari J; Lessells R; Naidoo Y; Ngcapu S; Pillay S; Ramphal U; Samsunder N; Sivro A; Tegally H; Wilkinson E; de Oliveira T
EPI_ISL_1628686, EPI_ISL_1628687, EPI_ISL_1628688, EPI_ISL_1628689, EPI_ISL_1628690, EPI_ISL_1628691, EPI_ISL_1628692, EPI_ISL_1628693, EPI_ISL_1628694, EPI_ISL_1628695, EPI_ISL_1628696, EPI_ISL_1628697, EPI_ISL_1628698, EPI_ISL_1628699, EPI_ISL_1628709, EPI_ISL_1628710, EPI_ISL_1628711, EPI_ISL_1628712, EPI_ISL_1628713, EPI_ISL_1628714, EPI_ISL_1628715, EPI_ISL_1628716, EPI_ISL_1628717, EPI_ISL_1628718, EPI_ISL_1628719, EPI_ISL_1628720, EPI_ISL_1628721, EPI_ISL_1628722, EPI_ISL_1628740, EPI_ISL_1628741, EPI_ISL_1628742, EPI_ISL_1628743, EPI_ISL_1628753, EPI_ISL_1628754, EPI_ISL_1628755, EPI_ISL_1628770, EPI_ISL_1628771, EPI_ISL_1628772, EPI_ISL_1628773, EPI_ISL_1628776, EPI_ISL_1628812, EPI_ISL_1628813, EPI_ISL_1628814, EPI_ISL_1628815, EPI_ISL_1628818, EPI_ISL_1628819, EPI_ISL_1628820, EPI_ISL_1628821, EPI_ISL_1628822, EPI_ISL_1628823, EPI_ISL_1628824, EPI_ISL_1628834, EPI_ISL_1628835, EPI_ISL_1628836, EPI_ISL_1628837, EPI_ISL_1628838, EPI_ISL_1628839, EPI_ISL_1628840, EPI_ISL_1628841, EPI_ISL_1628842, EPI_ISL_1628851, EPI_ISL_1628852, EPI_ISL_1628853, EPI_ISL_1628872, EPI_ISL_1628873, EPI_ISL_1628874, EPI_ISL_1628875, EPI_ISL_1628876, EPI_ISL_1628877, EPI_ISL_1628878, EPI_ISL_1628879, EPI_ISL_1628880, EPI_ISL_1628881, EPI_ISL_1628882, EPI_ISL_1628883, EPI_ISL_1628884, EPI_ISL_1628885, EPI_ISL_1628886, EPI_ISL_1628887, EPI_ISL_1628888, EPI_ISL_1628913, EPI_ISL_1628914, EPI_ISL_1628952, EPI_ISL_1628953, EPI_ISL_1628954, EPI_ISL_1628955, EPI_ISL_1628956, EPI_ISL_1628957, EPI_ISL_1628958, EPI_ISL_1628959, EPI_ISL_1628960, EPI_ISL_1628961, EPI_ISL_1628962, EPI_ISL_1628963, EPI_ISL_1628964, EPI_ISL_1628965, EPI_ISL_1628966, EPI_ISL_1628967, EPI_ISL_1628968, EPI_ISL_1628969, EPI_ISL_1628970, EPI_ISL_1628971, EPI_ISL_1628972, EPI_ISL_1628973, EPI_ISL_1628974, EPI_ISL_1628975, EPI_ISL_1628976, EPI_ISL_1628979, EPI_ISL_1628980, EPI_ISL_1628981, EPI_ISL_1628982, EPI_ISL_1628983, EPI_ISL_1628984, EPI_ISL_1628985, EPI_ISL_1628986, EPI_ISL_1628987, EPI_ISL_1628988, EPI_ISL_1628989, EPI_ISL_1628990, EPI_ISL_1628991, EPI_ISL_1628992, EPI_ISL_1628993, EPI_ISL_1628994, EPI_ISL_1628995, EPI_ISL_1628996, EPI_ISL_1628997, EPI_ISL_1628998, EPI_ISL_1628999, EPI_ISL_1629000, EPI_ISL_1629011, EPI_ISL_1629012, EPI_ISL_1629013, EPI_ISL_1629034, EPI_ISL_1629035, EPI_ISL_1629036, EPI_ISL_1629037, EPI_ISL_1629038, EPI_ISL_1629039, EPI_ISL_1629040, EPI_ISL_1629041, EPI_ISL_1629042, EPI_ISL_1629043, EPI_ISL_1629044, EPI_ISL_1629045, EPI_ISL_1629046, EPI_ISL_1629047, EPI_ISL_1629048, EPI_ISL_1629049, EPI_ISL_1629050, EPI_ISL_1629051, EPI_ISL_1629052, EPI_ISL_1629126, EPI_ISL_1629127, EPI_ISL_1629128, EPI_ISL_1629129, EPI_ISL_1629130, EPI_ISL_1629131, EPI_ISL_1629132, EPI_ISL_1629133, EPI_ISL_1629134, EPI_ISL_1629135, EPI_ISL_1629136, EPI_ISL_1629137, EPI_ISL_1629138, EPI_ISL_1629139, EPI_ISL_1629140, EPI_ISL_1629141, EPI_ISL_1629142, EPI_ISL_1629143, EPI_ISL_1629144, EPI_ISL_1629145, EPI_ISL_1629146, EPI_ISL_1629147, EPI_ISL_1629148, EPI_ISL_1629149, EPI_ISL_1629150, EPI_ISL_1629151, EPI_ISL_1629152, EPI_ISL_1629153, EPI_ISL_1629154, EPI_ISL_1629155, EPI_ISL_1629156, EPI_ISL_1629157, EPI_ISL_1629158, EPI_ISL_1629159, EPI_ISL_1629160, EPI_ISL_1629161, EPI_ISL_1629162, EPI_ISL_1629163, EPI_ISL_1629350, EPI_ISL_1629351, EPI_ISL_1629352, EPI_ISL_1629353, EPI_ISL_1629354, EPI_ISL_1629355, EPI_ISL_2090731, EPI_ISL_2090733, EPI_ISL_2090734, EPI_ISL_2090735, EPI_ISL_2090736, EPI_ISL_2090737, EPI_ISL_2090738, EPI_ISL_2090745, EPI_ISL_2090746, EPI_ISL_2090747, EPI_ISL_2090748, EPI_ISL_2090750, EPI_ISL_3154041, EPI_ISL_3154097			
see above	CERBALLIANCE	UMR PIMIT	Dr Camille Lebarbenchon; Dr David A Wilkinson; Dr Patrick Mavingui; Magali Turpin; Marie-Alice Simbi
EPI_ISL_2812957	CH Mayotte	Institut Pasteur	Angela Brisebarre; Camille Capel; Christophe Malabat; Combe Patrice; Corinne Maufrais; Emmanuelle Permal; Etienne Simon-Lorière; Frédéric Lemoine; Louise Lefrançois; Marion Barbet; Maud Vanpeene; Méline Bizard; Sylvie Behillil; Sylvie Van der Werf; Vincent Enouf
EPI_ISL_2086873, EPI_ISL_2086874, EPI_ISL_2086875, EPI_ISL_2086876, EPI_ISL_2086877, EPI_ISL_2086878, EPI_ISL_2086879, EPI_ISL_2086880, EPI_ISL_2086881, EPI_ISL_2086882, EPI_ISL_2086883, EPI_ISL_2086884, EPI_ISL_2086885, EPI_ISL_2086886, EPI_ISL_2086887, EPI_ISL_2086888, EPI_ISL_2086889, EPI_ISL_2086890, EPI_ISL_2086891, EPI_ISL_2086892, EPI_ISL_2086893, EPI_ISL_2086894, EPI_ISL_2086895, EPI_ISL_2086896, EPI_ISL_2086897, EPI_ISL_2086898, EPI_ISL_2086899, EPI_ISL_2086900, EPI_ISL_2086901, EPI_ISL_2086902, EPI_ISL_2086903, EPI_ISL_2285233, EPI_ISL_2285234, EPI_ISL_2285235, EPI_ISL_2285236, EPI_ISL_2285237, EPI_ISL_2285238, EPI_ISL_2285239, EPI_ISL_2285327, EPI_ISL_2285328, EPI_ISL_2285329, EPI_ISL_2285330, EPI_ISL_2285331, EPI_ISL_2285332, EPI_ISL_2285333, EPI_ISL_2285334, EPI_ISL_2285337, EPI_ISL_2285338			
see above	CHARLOTTE MAXEKE JOHANNESBURG ACADEMIC HOSPITAL	National Institute for Communicable Diseases of the National Health Laboratory Service	Amoako DG; Bhiman JN; Ismail A; Mahlangu B; Mohale T; Ntuli N; Scheepers C
EPI_ISL_1628739, EPI_ISL_1628860, EPI_ISL_1628977, EPI_ISL_1628978, EPI_ISL_1629032, EPI_ISL_1629033, EPI_ISL_1629053, EPI_ISL_2090742			
see above	CHOR	UMR PIMIT	Dr Camille Lebarbenchon; Dr David A Wilkinson; Dr Patrick Mavingui; Magali Turpin
EPI_ISL_1336529, EPI_ISL_1336530, EPI_ISL_1336531, EPI_ISL_1336532, EPI_ISL_1336574, EPI_ISL_1336575, EPI_ISL_1336576, EPI_ISL_1336577, EPI_ISL_1336578, EPI_ISL_1336580, EPI_ISL_1336581, EPI_ISL_1336582, EPI_ISL_1336583, EPI_ISL_1336584, EPI_ISL_1336585, EPI_ISL_1336586, EPI_ISL_1336587, EPI_ISL_1336588, EPI_ISL_1336589, EPI_ISL_1371761			
see above	CHR LA REUNION FELIX GUYON	CNR Virus des Infections Respiratoires - France SUD	Antonin Bal; Bruno Lina; Bruno Simon; Gregory Destras; Gwendolyne Burfin; Hadrien Regue; Laurence Josset; Martine Valette; Quentin Semanas

EPI_ISL_2086904, EPI_ISL_2086905, EPI_ISL_2086906, EPI_ISL_2086907, EPI_ISL_2086908, EPI_ISL_2086910, EPI_ISL_2086911, EPI_ISL_2086912, EPI_ISL_2086913, EPI_ISL_2086915, EPI_ISL_2086921, EPI_ISL_2086922, EPI_ISL_2086923, EPI_ISL_2086924, EPI_ISL_2153516, EPI_ISL_2153517, EPI_ISL_2153518, EPI_ISL_2153519, EPI_ISL_2153520, EPI_ISL_2153521, EPI_ISL_2153522, EPI_ISL_2285249, EPI_ISL_2285250, EPI_ISL_2442366, EPI_ISL_2442367, EPI_ISL_2442368, EPI_ISL_2442369, EPI_ISL_2662468, EPI_ISL_2662469, EPI_ISL_2662470, EPI_ISL_2662471, EPI_ISL_2662472, EPI_ISL_2662473, EPI_ISL_2662474, EPI_ISL_2662492, EPI_ISL_2662493, EPI_ISL_2662494, EPI_ISL_2662495, EPI_ISL_2662496, EPI_ISL_2662497, EPI_ISL_2662498, EPI_ISL_2662499, EPI_ISL_2662500, EPI_ISL_2662501, EPI_ISL_2662502, EPI_ISL_2662503, EPI_ISL_2662504, EPI_ISL_2662505, EPI_ISL_2662506, EPI_ISL_2662507, EPI_ISL_2662508, EPI_ISL_2662509, EPI_ISL_2662510, EPI_ISL_2662554, EPI_ISL_2662555			
see above	CHRIS HANI BARAGWANATH LABORATORY	National Institute for Communicable Diseases of the National Health Laboratory Service	Amoako DG; Bhiman JN; Everatt J; Ismail A; Mahlangu B; Mnguni A; Mohale T; Ntuli N; Scheepers C
EPI_ISL_1628723, EPI_ISL_1628724, EPI_ISL_1628736, EPI_ISL_1628737, EPI_ISL_1628738, EPI_ISL_1628803, EPI_ISL_1628804, EPI_ISL_1628805, EPI_ISL_1628806, EPI_ISL_1628807, EPI_ISL_1628808, EPI_ISL_1628809, EPI_ISL_1628810, EPI_ISL_1628811, EPI_ISL_1628826, EPI_ISL_1628827, EPI_ISL_1628828, EPI_ISL_1628829, EPI_ISL_1628830, EPI_ISL_1628831, EPI_ISL_1628832, EPI_ISL_1628833, EPI_ISL_1628843, EPI_ISL_1628854, EPI_ISL_1628889, EPI_ISL_1628890, EPI_ISL_1628891, EPI_ISL_1628919, EPI_ISL_1628920, EPI_ISL_1628921, EPI_ISL_1628922, EPI_ISL_1628923, EPI_ISL_1628924, EPI_ISL_1628925, EPI_ISL_1629001, EPI_ISL_1629002, EPI_ISL_1629003, EPI_ISL_1629004, EPI_ISL_1629005, EPI_ISL_1629006, EPI_ISL_1629007, EPI_ISL_1629008, EPI_ISL_1629009, EPI_ISL_1629010, EPI_ISL_1629021, EPI_ISL_1629022, EPI_ISL_1629023, EPI_ISL_1629024, EPI_ISL_1629025, EPI_ISL_1629026, EPI_ISL_1629027, EPI_ISL_1629028, EPI_ISL_1629054, EPI_ISL_1629055, EPI_ISL_1629056, EPI_ISL_1629057, EPI_ISL_1629058, EPI_ISL_1629059, EPI_ISL_1629060, EPI_ISL_1629061, EPI_ISL_1629062, EPI_ISL_1629063, EPI_ISL_1629064, EPI_ISL_1629065, EPI_ISL_1629066, EPI_ISL_1629067, EPI_ISL_1629068, EPI_ISL_1629069, EPI_ISL_1629070, EPI_ISL_1629071, EPI_ISL_1629072, EPI_ISL_1629073, EPI_ISL_1629074, EPI_ISL_1629102, EPI_ISL_1629103, EPI_ISL_1629104, EPI_ISL_1629105, EPI_ISL_1629106, EPI_ISL_1629107, EPI_ISL_1629174, EPI_ISL_1629175, EPI_ISL_1629176, EPI_ISL_1629177, EPI_ISL_1629183, EPI_ISL_1629184, EPI_ISL_1629187, EPI_ISL_1629188, EPI_ISL_1629381, EPI_ISL_1629382, EPI_ISL_1629383, EPI_ISL_1629384, EPI_ISL_1629385, EPI_ISL_1629386, EPI_ISL_1629387, EPI_ISL_1629388, EPI_ISL_1629389, EPI_ISL_1629390, EPI_ISL_1629391, EPI_ISL_1629392, EPI_ISL_1629393, EPI_ISL_1629394, EPI_ISL_1629395, EPI_ISL_1629396, EPI_ISL_1629397, EPI_ISL_1629472			
see above	CHU	UMR PIMIT	Dr Camille Lebarbenchon; Dr David A Wilkinson; Dr Patrick Mavingui; Magali Turpin
EPI_ISL_1628747, EPI_ISL_1628748, EPI_ISL_1628749, EPI_ISL_1628750, EPI_ISL_1628751, EPI_ISL_1628756, EPI_ISL_1628757, EPI_ISL_1628758, EPI_ISL_1628759, EPI_ISL_1628760, EPI_ISL_1628761, EPI_ISL_1628762, EPI_ISL_1628763, EPI_ISL_1629260, EPI_ISL_1629263, EPI_ISL_2090729, EPI_ISL_2090730, EPI_ISL_2090740, EPI_ISL_2090741, EPI_ISL_2090749, EPI_ISL_2090751, EPI_ISL_2090752, EPI_ISL_2090753, EPI_ISL_3154370, EPI_ISL_3154371			
see above	CHU NORD	UMR PIMIT	Dr Camille Lebarbenchon; Dr David A Wilkinson; Dr Patrick Mavingui; Magali Turpin; Marie-Alice Simbi
EPI_ISL_1265825, EPI_ISL_1265826, EPI_ISL_1265834, EPI_ISL_1265839, EPI_ISL_1265840, EPI_ISL_1265841, EPI_ISL_1265851, EPI_ISL_1265852			
see above	CHU REUNION	CNR Virus des Infections Respiratoires - France SUD	Antonin Bal; Bruno Lina; Bruno Simon; Gregory Destras; Gwendolyne Burfin; Hadrien Regue; Laurence Josset; Martine Valette; Quentin Semanas
EPI_ISL_2681640, EPI_ISL_2682597, EPI_ISL_2683495, EPI_ISL_2683497, EPI_ISL_2683873	CICM, Bamako	Malaria Research and Training Center-Bamako	Abdoul Karim Sangare; Abdoulaye Djimde; Amadou Daou; Antoine Dara; Bourema Kouriba
EPI_ISL_2672214, EPI_ISL_2672215	CICM, Bamako	Malaria Research and Training Center-Parasito	Abdoul Karim Sangare; Abdoulaye Djimde; Amadou Daou; Antoine Dara; Bourema Kouriba
EPI_ISL_1299497, EPI_ISL_1299498, EPI_ISL_1591099, EPI_ISL_1591100	CMA Dano	Centre Muraz	Abdoul-Salam Ouedraogo; Ange Badjo; Armel Poda; Arsène Somé; Arsène Zongo; Essia Belarbi; Fabian Leendertz; Firmin Kaboré; Grit Schubert; Jasmin Schlotterbeck; Soumeya Ouangraoua; Thérèse Kagone; Yacouba Sawadogo
EPI_ISL_1534294, EPI_ISL_1534383, EPI_ISL_1534404	Calitzdorp (Bergsig) Clinic wc CDC	NHLS/UCT	Arash Iranzadeh; Bruna Galvao; Carolyn Williamson; Deelan Doolabh; Diana Hardie; Emmanuel SJ; Innocent Mudau; Kruger Marais; Lynn Tyers; Marvin Hsiao; Stephen Korsman; Tegally H; de Oliveira T
EPI_ISL_1817676, EPI_ISL_1817720, EPI_ISL_1817723, EPI_ISL_1817725, EPI_ISL_1817728, EPI_ISL_1817732	Calvinia RSC Clinic	NHLS/UCT	Arash Iranzadeh; Bruna Galvao; Carolyn Williamson; Deelan Doolabh; Diana Hardie; Innocent Mudau; Kruger Marais; Lynn Tyers; Marvin Hsiao; Stephen Korsman
EPI_ISL_2835622, EPI_ISL_2835623, EPI_ISL_2835624, EPI_ISL_2835625, EPI_ISL_2835627, EPI_ISL_2835628, EPI_ISL_2835629, EPI_ISL_2835630, EPI_ISL_2835631, EPI_ISL_2835632, EPI_ISL_2835633, EPI_ISL_2835634, EPI_ISL_2835635, EPI_ISL_2835636, EPI_ISL_2835637, EPI_ISL_2835638, EPI_ISL_2835639, EPI_ISL_2835640			
see above	Center for Medical and Sanitary Research (CERMES)	CDC Atlanta	Adamou Lagare; Dhwani Batra; Justin Lee
EPI_ISL_2227344, EPI_ISL_2232252, EPI_ISL_2232253, EPI_ISL_2232254, EPI_ISL_2232255, EPI_ISL_2232256, EPI_ISL_2232257, EPI_ISL_2232258, EPI_ISL_2232259, EPI_ISL_2232260, EPI_ISL_2232261, EPI_ISL_2232262, EPI_ISL_2232263, EPI_ISL_2232264, EPI_ISL_2232265, EPI_ISL_2232266, EPI_ISL_2232267, EPI_ISL_2232268, EPI_ISL_2232269, EPI_ISL_2232270, EPI_ISL_2232271, EPI_ISL_2232272, EPI_ISL_2232273, EPI_ISL_2232274, EPI_ISL_2232275, EPI_ISL_2232276, EPI_ISL_2232277, EPI_ISL_2232278, EPI_ISL_2232279, EPI_ISL_2232280, EPI_ISL_2232281, EPI_ISL_2232282, EPI_ISL_2232283, EPI_ISL_2232284, EPI_ISL_2232285, EPI_ISL_2232286, EPI_ISL_2232287, EPI_ISL_2232288, EPI_ISL_2232289, EPI_ISL_2232290, EPI_ISL_2232291, EPI_ISL_2232292, EPI_ISL_2232293, EPI_ISL_2232294, EPI_ISL_2232295, EPI_ISL_2232296, EPI_ISL_2232297, EPI_ISL_2232298, EPI_ISL_2232299, EPI_ISL_2232300, EPI_ISL_2232301, EPI_ISL_2232302, EPI_ISL_2232303, EPI_ISL_2232304, EPI_ISL_2232305, EPI_ISL_2232306, EPI_ISL_2232307, EPI_ISL_2232308, EPI_ISL_2232309, EPI_ISL_2232310, EPI_ISL_2232311, EPI_ISL_2232312, EPI_ISL_2232313, EPI_ISL_2232314, EPI_ISL_2232315, EPI_ISL_2232316, EPI_ISL_2232317, EPI_ISL_2232318, EPI_ISL_2232319, EPI_ISL_2232320, EPI_ISL_2232321, EPI_ISL_2232322, EPI_ISL_2232323, EPI_ISL_2232324, EPI_ISL_2232325, EPI_ISL_2232326, EPI_ISL_2232327, EPI_ISL_2232328, EPI_ISL_2232329, EPI_ISL_2232330, EPI_ISL_2232331, EPI_ISL_2232332, EPI_ISL_2232333, EPI_ISL_2232334, EPI_ISL_2232335, EPI_ISL_2232336, EPI_ISL_2232337, EPI_ISL_2232338, EPI_ISL_2232339, EPI_ISL_2232340, EPI_ISL_2232341, EPI_ISL_2232342, EPI_ISL_2232343, EPI_ISL_2232344, EPI_ISL_2232345, EPI_ISL_2232346, EPI_ISL_2232347, EPI_ISL_2232348, EPI_ISL_2232349, EPI_ISL_2232350, EPI_ISL_2232351, EPI_ISL_2232352, EPI_ISL_2232353, EPI_ISL_2232354, EPI_ISL_2232355, EPI_ISL_2232356, EPI_ISL_2232357, EPI_ISL_2232358, EPI_ISL_2232359, EPI_ISL_2232360, EPI_ISL_2232361, EPI_ISL_2232362, EPI_ISL_2232363, EPI_ISL_2232364, EPI_ISL_2232365, EPI_ISL_2232366, EPI_ISL_2232367, EPI_ISL_2232368, EPI_ISL_2232369, EPI_ISL_2232370, EPI_ISL_2232371, EPI_ISL_2232372, EPI_ISL_2232373, EPI_ISL_2232374, EPI_ISL_2232375, EPI_ISL_2232376, EPI_ISL_2232377, EPI_ISL_2232378, EPI_ISL_2232379, EPI_ISL_2232380, EPI_ISL_2232381, EPI_ISL_2232382, EPI_ISL_2232383, EPI_ISL_2232384, EPI_ISL_2232385, EPI_ISL_2232386, EPI_ISL_2232387, EPI_ISL_2232388, EPI_ISL_2232389, EPI_ISL_2232390, EPI_ISL_2232391, EPI_ISL_2232392, EPI_ISL_2232393, EPI_ISL_2232394, EPI_ISL_2232395, EPI_ISL_2232396, EPI_ISL_2232397, EPI_ISL_2232398, EPI_ISL_2232399, EPI_ISL_2232400, EPI_ISL_2232401, EPI_ISL_2232402, EPI_ISL_2232403, EPI_ISL_2232404, EPI_ISL_2232405, EPI_ISL_2232406, EPI_ISL_2232407, EPI_ISL_2380050, EPI_ISL_2380051, EPI_ISL_2380052, EPI_ISL_2380053, EPI_ISL_2380054, EPI_ISL_2380055, EPI_ISL_2380056, EPI_ISL_2380057, EPI_ISL_2380058, EPI_ISL_2380059, EPI_ISL_2380060, EPI_ISL_2380061, EPI_ISL_2380062, EPI_ISL_2380063, EPI_ISL_2380064, EPI_ISL_2380065, EPI_ISL_2380066, EPI_ISL_2380067, EPI_ISL_2380068, EPI_ISL_2380069, EPI_ISL_2380070, EPI_ISL_2380071, EPI_ISL_2380072, EPI_ISL_2380073, EPI_ISL_2380074, EPI_ISL_2380075, EPI_ISL_2380076, EPI_ISL_2380077, EPI_ISL_2380078, EPI_ISL_2380079, EPI_ISL_2380080, EPI_ISL_2380081, EPI_ISL_2380082, EPI_ISL_2380083, EPI_ISL_2380084, EPI_ISL_2380085, EPI_ISL_2380086, EPI_ISL_2380087, EPI_ISL_2380088, EPI_ISL_2380089, EPI_ISL_2380090, EPI_ISL_2380091, EPI_ISL_2380092, EPI_ISL_2380093, EPI_ISL_2380094, EPI_ISL_2380095, EPI_ISL_2380096, EPI_ISL_2380097, EPI_ISL_2380098			
see above	Center of Scientific Excellence for Influenza Viruses (CSEIV), National Research Centre	Center of Scientific Excellence for Influenza Viruses (CSEIV), National Research Centre	Ahmed E Kayed; Ahmed El-Taweel; Ahmed Kandeil; Ahmed Mostafa; Amal Naguib; Ghazi Kayali; M K Khalifa; Mahmoud Shehata; Mina Kamel; Mohamed Ahmed Ali; Mohamed El Sayes; Mohamed Hassany; Mokhtar Gomaa; Nancy M. El Guindy; Noura M Abo Shama; Omnia Kutkat; Rabeh El-Shesheny; Richard Webby; Sara Mahmoud; Shymaa S Ahmed; Wael Roshdy; Yassmin Moatasim
EPI_ISL_2285162, EPI_ISL_2285163, EPI_ISL_2285164, EPI_ISL_2285165, EPI_ISL_2285166, EPI_ISL_2285167	Central Health Laboratory	National Institute for Communicable Diseases of the National Health Laboratory Service	Allam M; Amoako DG; Baboo SB; Bhiman JN; Ismail A; Mahlangu B; Manraj SS; Mohale T; Ntuli N; Ramuth M; Sonoo J
EPI_ISL_1357599, EPI_ISL_1357601, EPI_ISL_1357603, EPI_ISL_1357604	Centre For Biotechnology Research And Development	THE AFRICA GENOMICS CENTRE AND CONSULTANCY	Adede Hawi; Cecilia Waruhiu; Damaris Matoke Muhia; Damaris Matoke-Muhia; George Michuki; John Njuguna; Lilian Kanjau; Ravena Mubichi
EPI_ISL_1357597	Centre For Biotechnology Research And Development	THE AFRICA GENOMICS CENTRE AND CONSULTANCY LIMITED	Adede Hawi; Cecilia Waruhiu; Damaris Matoke-Muhia; George Michuki; John Njuguna; Lilian Kanjau; Ravena Mubichi
EPI_ISL_1819026, EPI_ISL_1819027, EPI_ISL_1819028, EPI_ISL_1819029, EPI_ISL_1819030, EPI_ISL_1819031, EPI_ISL_1819032, EPI_ISL_1819033, EPI_ISL_1819034, EPI_ISL_1819035, EPI_ISL_1819036, EPI_ISL_1819037, EPI_ISL_1819038, EPI_ISL_1819039, EPI_ISL_1819040, EPI_ISL_1819041, EPI_ISL_1819042, EPI_ISL_1819043, EPI_ISL_1819044			
see above	Centre Hospitalier Universitaire Mère-Enfant Fondation Jeanne Ebori (CHUMEFJE)	Centre de recherches médicales de Lambaréné (CERMEL)	Ayola A. Adegnika; Ayong Moure; Bertrand Lell; Bénédicte Ndeboko; Emilio Skarwan; Georgelin Nguema Ondo; Gédéon P. Manouana; Haruka Abe; Jiro Yasuda; Joel Fleury Djoba Siawaya; Rodrigue Bikangui; Rotimi Myrabelle Avome Houechenou; Samira Zoa-Assoumou; Yuri Ushijima
EPI_ISL_1760556	Centre Hospitalier Universitaire Mère-Enfant, Fondation Jeanne Ebori (CHUMEFJE)	Centre de recherches médicales de Lambaréné (CERMEL)	Ayola A. Adegnika; Ayong Moure; Bertrand Lell; Bénédicte Ndeboko; Emilio Skarwan; Georgelin Nguema Ondo; Gédéon P. Manouana; Haruka Abe; Jiro Yasuda; Joel Fleury Djoba Siawaya; Rodrigue Bikangui; Rotimi Myrabelle Avome Houechenou; Samira Zoa-Assoumou; Yuri Ushijima
EPI_ISL_1299491, EPI_ISL_1299492, EPI_ISL_1299493, EPI_ISL_1299494, EPI_ISL_1299495, EPI_ISL_1299496, EPI_ISL_1299499			
see above	Centre Hospitalo Universitaire Souro Sanou	Centre Muraz	Abdoul-Salam Ouedraogo; Ange Badjo; Armel Poda; Arsène Somé; Arsène Zongo; Essia Belarbi; Fabian Leendertz; Firmin Kaboré; Grit Schubert; Jasmin Schlotterbeck; Soumeya Ouangraoua; Thérèse Kagone; Yacouba Sawadogo
EPI_ISL_1790077, EPI_ISL_1790078, EPI_ISL_1790079, EPI_ISL_1790080, EPI_ISL_1790081, EPI_ISL_1790082, EPI_ISL_1790083, EPI_ISL_1790084, EPI_ISL_1790085, EPI_ISL_1790086, EPI_ISL_1790087, EPI_ISL_1790088, EPI_ISL_1790089, EPI_ISL_1790090, EPI_ISL_1790091, EPI_ISL_1790092, EPI_ISL_1790093, EPI_ISL_1790094, EPI_ISL_1790095, EPI_ISL_1790096, EPI_ISL_1790097, EPI_ISL_1790098, EPI_ISL_1790099, EPI_ISL_1790101, EPI_ISL_1790102, EPI_ISL_1790103			
see above	Centre Pasteur Annexe de Garoua	Institut Pasteur de Dakar	Carniel Elisabeth; Dia Ndongo; Diagne Moussa Moïse; Diallo Amadou; Diop Mamadou; Faye Ousmane; Loucoubar Cheikh; Ndiaye Ndack; Njouom Richard; Sall Amadou Alpha; Sankhe Safietou
EPI_ISL_1257874, EPI_ISL_1257875, EPI_ISL_1257876, EPI_ISL_1257877, EPI_ISL_1257878, EPI_ISL_1257879, EPI_ISL_1257880, EPI_ISL_1257881, EPI_ISL_1790104, EPI_ISL_1790105, EPI_ISL_1790106, EPI_ISL_1790107, EPI_ISL_1790108, EPI_ISL_1790109, EPI_ISL_1790110, EPI_ISL_1790111, EPI_ISL_1790112, EPI_ISL_1790113, EPI_ISL_1790114, EPI_ISL_1790115, EPI_ISL_1790116, EPI_ISL_1790117, EPI_ISL_1790118, EPI_ISL_1790119, EPI_ISL_1790120, EPI_ISL_1790121, EPI_ISL_1790122, EPI_ISL_1790123, EPI_ISL_1790124, EPI_ISL_1790125, EPI_ISL_1790126, EPI_ISL_1790127, EPI_ISL_1790128, EPI_ISL_1790129, EPI_ISL_1790130, EPI_ISL_1790131, EPI_ISL_1790132, EPI_ISL_1790133			
see above	Centre Pasteur du Cameroun	Institut Pasteur de Dakar	Carniel Elisabeth; Dia Ndongo; Diagne Moussa Moïse; Diallo Amadou; Diop Mamadou; Elisabeth Carniel; Faye Ousmane; Loucoubar Cheikh; Ndiaye Ndack; Njouom Richard; Sall Amadou Alpha; Sankhe Safietou
EPI_ISL_1970569	Centre de Recherche sur les Maladies Infectieuses	Centre de Recherche sur les Maladies Infectieuses	Mfoutou Mapanguy Claujeans Chastel and Batchi-Bouyou Armel Landry; Prof. Dr. Thirumalaisamy P. Velavan; Prof. Francine Ntoumi
EPI_ISL_1913035, EPI_ISL_1935059, EPI_ISL_2036198, EPI_ISL_2036199, EPI_ISL_2097223, EPI_ISL_2097224, EPI_ISL_2097225, EPI_ISL_2097226, EPI_ISL_2097227, EPI_ISL_2097228, EPI_ISL_2097229, EPI_ISL_2156806, EPI_ISL_2156807, EPI_ISL_2156809, EPI_ISL_2156822, EPI_ISL_2156825, EPI_ISL_2424136, EPI_ISL_2424137, EPI_ISL_2424138			
see above	Centre de Recherches Médicales de Lambaréné (CERMEL)	Centre de Recherches Médicales de Lambaréné (CERMEL)	Anicet Mouity Matoumba; Bertrand Lell and Ayola Akim Adegnika; Georgelin Nguema Ondo; Gédéon Prince Manouana; Jean Bernard Lekana-Douki; Joël-Fleury Djoba Siawaya; Michel Ngonga Dikongo; Moustapha Nzamba Maloum; Rodrigue Bikangui; Sam O'neilla Oye Bingono; Samira Zoa Assoumou; Srinivas reddy Pallerla; Steffen Borrmann; Thirumalaisamy P. Velavan
EPI_ISL_2968035, EPI_ISL_2968036, EPI_ISL_2968037, EPI_ISL_2968038, EPI_ISL_2968039, EPI_ISL_2968040, EPI_ISL_2968041, EPI_ISL_2968042, EPI_ISL_2968043, EPI_ISL_2968044, EPI_ISL_2968045, EPI_ISL_2968046, EPI_ISL_2968047, EPI_ISL_2968048, EPI_ISL_2968049, EPI_ISL_2968050, EPI_ISL_2968051, EPI_ISL_2968052, EPI_ISL_2968053, EPI_ISL_2968054, EPI_ISL_2968055, EPI_ISL_2968056, EPI_ISL_2968057, EPI_ISL_2968058, EPI_ISL_2968059, EPI_ISL_2968060, EPI_ISL_2968061, EPI_ISL_2968062, EPI_ISL_2968063, EPI_ISL_2968064, EPI_ISL_2968065, EPI_ISL_2968066, EPI_ISL_2968067, EPI_ISL_2968068, EPI_ISL_2968069			
see above	Centre de Virologie des Maladies infectueuses Tropicales	Functional Genomic Platform UATRS-biology, CNRST	; Ahmed REGGAD; Charifa DRISSI TOUZANI; ELOUENNASS; Elmostafa BENAISSA; Elmostafa EL FAHIME; Fatna BSSAIBIS; Hemlali Mouhssine; Hicham EL ANNAZ; Idriss-Amine LAHLOU; Ikram BNOUYAHIA; Khalid ENNIBI; Khalid ENNIBI Mostafa; Marouane MELLOUL; Mly Abdelaziz ELALAOUI; Mostafa ELOUENNASS; Nadia EL MRIMAR; Nadia TOUIL; Rachid ABI; Rida TAGAJDID; Safae ELKOCHRI; Sanaa ALAOUI-Amine; Youssef AKHOUAD; Zohour KASMY
EPI_ISL_2351932, EPI_ISL_2382421, EPI_ISL_2382422, EPI_ISL_2382423, EPI_ISL_2398288, EPI_ISL_2398290, EPI_ISL_2398296, EPI_ISL_2399459, EPI_ISL_2399460, EPI_ISL_2399461, EPI_ISL_2399462, EPI_ISL_2501079, EPI_ISL_2501080, EPI_ISL_2501081, EPI_ISL_2501082, EPI_ISL_2501083, EPI_ISL_2501085, EPI_ISL_2501086, EPI_ISL_2501088, EPI_ISL_2501089, EPI_ISL_2502018, EPI_ISL_2502019, EPI_ISL_2502020, EPI_ISL_2502021, EPI_ISL_2502022, EPI_ISL_2502025, EPI_ISL_2502026			
see above	Centre for Human Virology and Genomics, Microbiology Department, Nigerian Institute of Medical Research	Central Research Laboratory, Nigerian Institute of Medical Research	Ayorinde Babatunde James; Azuka Patrick Okwuraiwe; Babatunde Lawal Salako; Bamidele Iwalokun; Chika Kingsley Onwuamah; Grace Oni; Joseph Ojonugwa Shaibu; Josiah Ayoola Isong; Joy Ayoola; Muinah Adenike Fowora; Ngozi Mirabel Otuonye; Nyam Itse Yusuf; Olufemi Samuel Amoo; Phasha-Muchemenye Mmatshepho; Rahaman A. Ahmed; Rosemary Ajuma Audu; Sharon Abimbola; Sola Ajibaye; Uyi Emokpae; Yusuf Jimoh
EPI_ISL_2442084, EPI_ISL_2442088, EPI_ISL_2442089, EPI_ISL_2442090, EPI_ISL_2442092, EPI_ISL_2442093, EPI_ISL_2442094, EPI_ISL_2442095, EPI_ISL_2442096, EPI_ISL_2442097, EPI_ISL_2442098, EPI_ISL_2442099, EPI_ISL_2442100, EPI_ISL_2442101, EPI_ISL_2442102, EPI_ISL_2442103, EPI_ISL_2442104, EPI_ISL_2442105, EPI_ISL_2442106, EPI_ISL_2442107, EPI_ISL_2442108, EPI_ISL_2442109, EPI_ISL_2442110, EPI_ISL_2442111, EPI_ISL_2442112, EPI_ISL_2442113, EPI_ISL_2442114, EPI_ISL_2442115, EPI_ISL_2442116, EPI_ISL_2442117, EPI_ISL_2442118, EPI_ISL_2442119, EPI_ISL_2442120, EPI_ISL_2442121, EPI_ISL_2442122, EPI_ISL_2442123, EPI_ISL_2442124, EPI_ISL_2442125, EPI_ISL_2442126, EPI_ISL_2442127, EPI_ISL_2442128, EPI_ISL_2442129, EPI_ISL_2442130, EPI_ISL_2442131, EPI_ISL_2442132, EPI_ISL_2442133, EPI_ISL_2442134, EPI_ISL_2442135, EPI_ISL_2442136, EPI_ISL_2442137, EPI_ISL_2442138, EPI_ISL_2442139, EPI_ISL_2442140, EPI_ISL_2442141, EPI_ISL_2442142, EPI_ISL_2442143, EPI_ISL_2442144, EPI_ISL_2442145, EPI_ISL_2442146, EPI_ISL_2442147, EPI_ISL_2442148, EPI_ISL_2442149, EPI_ISL_2442150, EPI_ISL_2442151, EPI_ISL_2442152, EPI_ISL_2442153, EPI_ISL_2442154, EPI_ISL_2442155, EPI_ISL_2442156, EPI_ISL_2442157, EPI_ISL_2442158, EPI_ISL_2442159, EPI_ISL_2442160, EPI_ISL_2442161, EPI_ISL_2442162, EPI_ISL_2442163, EPI_ISL_2442164, EPI_ISL_2442165, EPI_ISL_2442166, EPI_ISL_2442167, EPI_ISL_2442168, EPI_ISL_2442169, EPI_ISL_2442170, EPI_ISL_2442171, EPI_ISL_2442172, EPI_ISL_2442173, EPI_ISL_2442174, EPI_ISL_2442175, EPI_ISL_2442176, EPI_ISL_2442177, EPI_ISL_2442178, EPI_ISL_2442179, EPI_ISL_2442180, EPI_ISL_2442181, EPI_ISL_2442182, EPI_ISL_2442183, EPI_ISL_2442184, EPI_ISL_2442185, EPI_ISL_2442186, EPI_ISL_2442187, EPI_ISL_2442188, EPI_ISL_2442189, EPI_ISL_2442190, EPI_ISL_2442191, EPI_ISL_2442192, EPI_ISL_2442193, EPI_ISL_2442194, EPI_ISL_2442195, EPI_ISL_2442196, EPI_ISL_2442197, EPI_ISL_2442198, EPI_ISL_2442199, EPI_ISL_2442200, EPI_ISL_2442201, EPI_ISL_2442202			
see above	Centro de Investigação em Saúde de Manhiça (CISM) & ISGlobal, Institut de Salut Global de Barcelona	Instituto de Biomedicina de València - CSIC	Alfredo Mayor; Arsenia Massinga; Inacio Mandomando; Irving Cancino; Iñaki Comas; Manoli Torres Puente; Santiago Jiménez-Serrano
EPI_ISL_2504130, EPI_ISL_2504131, EPI_ISL_2504132, EPI_ISL_2504133, EPI_ISL_2504134	Chantal Biya International Reference Centre (CIRCB)	International Centre for Genetic Engineering and Biotechnology (ICGEB) and ARGO Open Lab for Genome Sequencing	Alessandro Marcello; Alexis Ndjolo; Celine Nkenfou; Collins Chenwi; Danilo Licastro; Desire Takou; Emanuele Orsini; Grace Angong Beloumou; Joseph Fokam; Ndine Fainguem; Simeone Dal Monego
EPI_ISL_1534264, EPI_ISL_3207534	Clinic-in-Asla	NHLS/UCT	Arash Iranzadeh; Bruna Galvao; Carolyn Williamson; Deelan Doolabh; Diana Hardie; Gert Marais; Innocent Mudau; Kruger Marais; Lynn Tyers; Marvin Hsiao; Rageema Joseph; Stephen Korsman
EPI_ISL_2035560, EPI_ISL_2035563, EPI_ISL_2035734, EPI_ISL_2035752, EPI_ISL_2035753, EPI_ISL_2035940	Clinical Virology	Clinical Virology	Anissa Chouikha; Henda Triki; Kais Ghedira; Mariem Gdoura; Sondos Haddad; Wasfi Fares; Wasfi Fraes

EPI_ISL_2035720	Clinical virology	Clinical Virology	Anissa Chouikha; Henda Triki; Kais Ghedira; Mariem Gdoura; Sondos Haddad; Wasfi Fares
EPI_ISL_1534273, EPI_ISL_1534274, EPI_ISL_1534298, EPI_ISL_1534340, EPI_ISL_1534355, EPI_ISL_1534395	CoVid WC Garden Route	NHLS/UCT	Arash Iranzadeh; Bruna Galvao; Carolyn Williamson; Deelan Doolabh; Diana Hardie; Emmanuel SJ; Innocent Mudau; Kruger Marais; Lynn Tyers; Marvin Hsiao; Stephen Korsman; Tegally H; de Oliveira T
EPI_ISL_1534334, EPI_ISL_1534399, EPI_ISL_1817707, EPI_ISL_2140657, EPI_ISL_2140658, EPI_ISL_2140660, EPI_ISL_2140663, EPI_ISL_2375934, EPI_ISL_2375935, EPI_ISL_2621055, EPI_ISL_2621057, EPI_ISL_2621064, EPI_ISL_2621065, EPI_ISL_2621067, EPI_ISL_2621068, EPI_ISL_2621070, EPI_ISL_2621073, EPI_ISL_2621075, EPI_ISL_2621076, EPI_ISL_2621079, EPI_ISL_2621087, EPI_ISL_2621091, EPI_ISL_2621092, EPI_ISL_2621093, EPI_ISL_2802133, EPI_ISL_3207510, EPI_ISL_3207527, EPI_ISL_3207528, EPI_ISL_3207532			
see above	Conville CDC wc CVC	NHLS/UCT	Arash Iranzadeh; Bruna Galvao; Carolyn Williamson; Deelan Doolabh; Diana Hardie; Emmanuel SJ; Gert Marais; Innocent Mudau; Kruger Marais; Lynn Tyers; Marvin Hsiao; Rageema Joseph; Stephen Korsman; Tegally H; de Oliveira T
EPI_ISL_1534338, EPI_ISL_1534419	Crags Clinic wc CRG	NHLS/UCT	Arash Iranzadeh; Bruna Galvao; Carolyn Williamson; Deelan Doolabh; Diana Hardie; Emmanuel SJ; Innocent Mudau; Kruger Marais; Lynn Tyers; Marvin Hsiao; Stephen Korsman; Tegally H; de Oliveira T
EPI_ISL_1534270, EPI_ISL_1534284, EPI_ISL_1534285, EPI_ISL_1534297, EPI_ISL_1534436, EPI_ISL_1534439, EPI_ISL_1534440, EPI_ISL_1534460, EPI_ISL_1817688, EPI_ISL_1817706, EPI_ISL_3207535, EPI_ISL_3207536			
see above	D'Almeida Clinic wc DAL	NHLS/UCT	Arash Iranzadeh; Bruna Galvao; Carolyn Williamson; Deelan Doolabh; Diana Hardie; Emmanuel SJ; Gert Marais; Innocent Mudau; Kruger Marais; Lynn Tyers; Marvin Hsiao; Rageema Joseph; Stephen Korsman; Tegally H; de Oliveira T
EPI_ISL_2779352, EPI_ISL_2779371, EPI_ISL_2779372, EPI_ISL_2779431, EPI_ISL_2779432, EPI_ISL_2779433, EPI_ISL_2779449, EPI_ISL_2779474, EPI_ISL_2779483, EPI_ISL_2779484, EPI_ISL_2779485, EPI_ISL_2779487, EPI_ISL_2779524, EPI_ISL_2779542, EPI_ISL_2779543			
see above	DFMH	USAMRD-A, Basic Science Laboratory	Alan Lemtudo; Beth Mutai; Brian Andika; Carol Kifude; Clement Masakwe; Eric Muthanje; Esther Omuseni; Faith Sigei; Gathii Kimita; George Awinda; John Waitumbi; Josphat Nyataya; Rachel Githii; Rehema Liyai; Stephen Ochola
EPI_ISL_1534421	DP Marais Hospital wc DPM	NHLS/UCT	Arash Iranzadeh; Bruna Galvao; Carolyn Williamson; Deelan Doolabh; Diana Hardie; Emmanuel SJ; Innocent Mudau; Kruger Marais; Lynn Tyers; Marvin Hsiao; Stephen Korsman; Tegally H; de Oliveira T
EPI_ISL_2153483, EPI_ISL_2153484, EPI_ISL_2153485, EPI_ISL_2153486, EPI_ISL_2153487, EPI_ISL_2153488, EPI_ISL_2153489, EPI_ISL_2153490, EPI_ISL_2153491, EPI_ISL_2153492, EPI_ISL_2153493, EPI_ISL_2153494, EPI_ISL_2153495, EPI_ISL_2153496, EPI_ISL_2153497, EPI_ISL_2153498, EPI_ISL_2153499, EPI_ISL_2153500, EPI_ISL_2153501, EPI_ISL_2153502, EPI_ISL_2153503, EPI_ISL_2153504, EPI_ISL_2153505, EPI_ISL_2153506, EPI_ISL_2153507, EPI_ISL_2153508, EPI_ISL_2153509, EPI_ISL_2153510, EPI_ISL_2153511, EPI_ISL_2153512, EPI_ISL_2153513, EPI_ISL_2153514, EPI_ISL_2153515, EPI_ISL_2285251, EPI_ISL_2285252, EPI_ISL_2582809, EPI_ISL_2662511, EPI_ISL_2662512, EPI_ISL_2662513, EPI_ISL_2662514, EPI_ISL_2662515, EPI_ISL_2662516, EPI_ISL_2662517, EPI_ISL_2662518, EPI_ISL_2662519, EPI_ISL_2662520, EPI_ISL_2662521, EPI_ISL_2662522, EPI_ISL_2662523, EPI_ISL_2662524, EPI_ISL_2662525, EPI_ISL_2662526, EPI_ISL_2662527, EPI_ISL_2662528, EPI_ISL_2662538, EPI_ISL_2662539, EPI_ISL_2662540, EPI_ISL_2662541, EPI_ISL_2662542, EPI_ISL_2662543, EPI_ISL_2662544, EPI_ISL_2662545, EPI_ISL_2662546, EPI_ISL_2662547, EPI_ISL_2662548, EPI_ISL_2662549, EPI_ISL_2662550, EPI_ISL_2662551, EPI_ISL_2662552, EPI_ISL_2662553			
see above	DR GEORGE MUKHARI LABORATORY	National Institute for Communicable Diseases of the National Health Laboratory Service	Amoako DG; Bhiman JN; Everatt J; Ismail A; Mahlangu B; Mnguni A; Mohale T; Ntuli N; Scheepers C
EPI_ISL_1534365	De Rust (Blommenek) Clinic wc DRC	NHLS/UCT	Arash Iranzadeh; Bruna Galvao; Carolyn Williamson; Deelan Doolabh; Diana Hardie; Emmanuel SJ; Innocent Mudau; Kruger Marais; Lynn Tyers; Marvin Hsiao; Stephen Korsman; Tegally H; de Oliveira T
EPI_ISL_2372404, EPI_ISL_2372406, EPI_ISL_2372407, EPI_ISL_2648210, EPI_ISL_2648212	Debswana Orapa Mine Hospital Laboratory	Botswana Harvard HIV Reference Laboratory	Boitumelo Zuze; Botshelo Radibe; Dorcas Maruapula; Joseph Makhema; Keoratile Ntshambiwa; Kgomotso Moruisi; Koketso Maotwe; Legodile Kooepile; Lesedi Magama; Madisa Mine; Mosepele Mosepele; Mpo Molapisi; Ontlametse T. Bareng; Pamela Smith-Lawrence; Roger Shapiro; Shahin Lockman; Sikhulile Moyo; Simani Gaseitsiwe; Thongbotho Mphoyakgosi; Wonderful T. Choga
EPI_ISL_1534373	District 6 CDC	NHLS/UCT	Arash Iranzadeh; Bruna Galvao; Carolyn Williamson; Deelan Doolabh; Diana Hardie; Emmanuel SJ; Innocent Mudau; Kruger Marais; Lynn Tyers; Marvin Hsiao; Stephen Korsman; Tegally H; de Oliveira T
EPI_ISL_2695801, EPI_ISL_2695802, EPI_ISL_2695803, EPI_ISL_2695804, EPI_ISL_2695805, EPI_ISL_2695806, EPI_ISL_2695807, EPI_ISL_2695808, EPI_ISL_2695809, EPI_ISL_2695810, EPI_ISL_2876349, EPI_ISL_2876350, EPI_ISL_2876351			
see above	Division of Medical Virology, National Health Laboratory Service (NHLS), Tygerberg Hospital / Stellenbosch University	Division of Medical Virology, National Health Laboratory Service (NHLS), Tygerberg Hospital / Stellenbosch University	Bronwyn Kleinhans; Gert van Zyl; San Emmanual James; Susan Engelbrecht; Tania Stander; Tongai Maponga; Tulio de Oliveira; Wolfgang Preiser
EPI_ISL_1502129, EPI_ISL_1502130, EPI_ISL_1502131, EPI_ISL_1502132, EPI_ISL_1502133, EPI_ISL_1502134, EPI_ISL_1502135, EPI_ISL_1502136, EPI_ISL_1502137, EPI_ISL_1502138, EPI_ISL_1502139, EPI_ISL_1502140, EPI_ISL_1502141, EPI_ISL_1502142, EPI_ISL_1502143, EPI_ISL_1502144, EPI_ISL_1502145, EPI_ISL_1591352, EPI_ISL_1591353, EPI_ISL_1591354, EPI_ISL_1591355, EPI_ISL_1591356, EPI_ISL_1591357, EPI_ISL_1591358, EPI_ISL_1591359, EPI_ISL_1591360, EPI_ISL_1591361, EPI_ISL_1591362, EPI_ISL_1591363, EPI_ISL_1591364, EPI_ISL_1591365, EPI_ISL_1591366, EPI_ISL_1591367, EPI_ISL_1591368, EPI_ISL_1591369, EPI_ISL_1591370, EPI_ISL_1591371, EPI_ISL_1591372, EPI_ISL_1591373, EPI_ISL_1591374, EPI_ISL_1591375, EPI_ISL_1591376, EPI_ISL_1591377, EPI_ISL_1591378, EPI_ISL_1591379, EPI_ISL_1591380, EPI_ISL_1591381, EPI_ISL_1591382, EPI_ISL_1591383, EPI_ISL_1591384, EPI_ISL_1591385, EPI_ISL_1591386, EPI_ISL_1591387, EPI_ISL_1591388, EPI_ISL_1591389, EPI_ISL_1591390, EPI_ISL_1591391, EPI_ISL_1591392, EPI_ISL_1591393, EPI_ISL_1591394, EPI_ISL_1591395, EPI_ISL_1591396, EPI_ISL_1591397, EPI_ISL_1591398, EPI_ISL_1591399, EPI_ISL_1591400, EPI_ISL_1591401, EPI_ISL_1591402, EPI_ISL_1591403, EPI_ISL_1591404, EPI_ISL_1591405, EPI_ISL_1591406, EPI_ISL_1591407, EPI_ISL_1591408, EPI_ISL_1591409, EPI_ISL_1591410, EPI_ISL_1591411, EPI_ISL_1591412, EPI_ISL_1591413, EPI_ISL_1591414, EPI_ISL_1591415, EPI_ISL_1591416, EPI_ISL_1591417, EPI_ISL_1591418, EPI_ISL_1591419, EPI_ISL_1591420, EPI_ISL_1591421, EPI_ISL_1591422, EPI_ISL_1591423, EPI_ISL_1591424, EPI_ISL_1591425, EPI_ISL_1591426, EPI_ISL_1591427, EPI_ISL_1591428, EPI_ISL_1591429, EPI_ISL_1591430, EPI_ISL_1591431, EPI_ISL_1591432, EPI_ISL_1591433, EPI_ISL_1591434, EPI_ISL_1591435, EPI_ISL_1591436, EPI_ISL_1591437, EPI_ISL_1591438, EPI_ISL_1591439, EPI_ISL_1591440, EPI_ISL_1591441, EPI_ISL_1591442, EPI_ISL_1591443, EPI_ISL_1591444, EPI_ISL_1591445, EPI_ISL_1591446, EPI_ISL_1591447, EPI_ISL_1591448, EPI_ISL_1591449, EPI_ISL_1591450, EPI_ISL_1591451, EPI_ISL_1591452, EPI_ISL_1591453, EPI_ISL_1591454, EPI_ISL_1591455, EPI_ISL_1608099, EPI_ISL_1608100, EPI_ISL_1608101, EPI_ISL_1608102, EPI_ISL_3066385, EPI_ISL_3066386, EPI_ISL_3066387, EPI_ISL_3066388, EPI_ISL_3066389, EPI_ISL_3066390, EPI_ISL_3066391, EPI_ISL_3066392, EPI_ISL_3066393, EPI_ISL_3066394, EPI_ISL_3066451, EPI_ISL_3066452, EPI_ISL_3066453, EPI_ISL_3066454, EPI_ISL_3066455, EPI_ISL_3066456, EPI_ISL_3066457, EPI_ISL_3066458, EPI_ISL_3066459, EPI_ISL_3066460, EPI_ISL_3066461, EPI_ISL_3066462, EPI_ISL_3066463, EPI_ISL_3066464, EPI_ISL_3066465, EPI_ISL_3066466, EPI_ISL_3066467, EPI_ISL_3066468, EPI_ISL_3066469, EPI_ISL_3066470, EPI_ISL_3066471, EPI_ISL_3066472, EPI_ISL_3066473, EPI_ISL_3066474, EPI_ISL_3066475, EPI_ISL_3066476, EPI_ISL_3066477, EPI_ISL_3066478, EPI_ISL_3066479, EPI_ISL_3066480, EPI_ISL_3066481, EPI_ISL_3066482, EPI_ISL_3066483, EPI_ISL_3066484, EPI_ISL_3066485, EPI_ISL_3066487, EPI_ISL_3066488, EPI_ISL_3066489, EPI_ISL_3066490, EPI_ISL_3066491, EPI_ISL_3066492, EPI_ISL_3066493, EPI_ISL_3066494, EPI_ISL_3066495, EPI_ISL_3066496, EPI_ISL_3066497, EPI_ISL_3066498, EPI_ISL_3066499, EPI_ISL_3066500, EPI_ISL_3066501, EPI_ISL_3066502, EPI_ISL_3066503, EPI_ISL_3066504, EPI_ISL_3066505, EPI_ISL_3066506, EPI_ISL_3066507, EPI_ISL_3066508, EPI_ISL_3066509, EPI_ISL_3066510, EPI_ISL_3066511, EPI_ISL_3066512, EPI_ISL_3066513, EPI_ISL_3066514, EPI_ISL_3066515, EPI_ISL_3066516, EPI_ISL_3066517, EPI_ISL_3066518, EPI_ISL_3066519, EPI_ISL_3066520, EPI_ISL_3066521, EPI_ISL_3066522, EPI_ISL_3066523, EPI_ISL_3067983, EPI_ISL_3067984, EPI_ISL_3067985, EPI_ISL_3067986, EPI_ISL_3067987, EPI_ISL_3067988, EPI_ISL_3067989, EPI_ISL_3067990, EPI_ISL_3067991, EPI_ISL_3067992, EPI_ISL_3067993, EPI_ISL_3067994, EPI_ISL_3067995, EPI_ISL_3067996, EPI_ISL_3067997, EPI_ISL_3067998, EPI_ISL_3067999, EPI_ISL_3068000, EPI_ISL_3068001, EPI_ISL_3068002, EPI_ISL_3068003, EPI_ISL_3068004, EPI_ISL_3068005, EPI_ISL_3068006, EPI_ISL_3068007, EPI_ISL_3068008, EPI_ISL_3068009, EPI_ISL_3068010, EPI_ISL_3068011, EPI_ISL_3068012, EPI_ISL_3068013, EPI_ISL_3068014, EPI_ISL_3068015, EPI_ISL_3068016, EPI_ISL_3068017, EPI_ISL_3068018, EPI_ISL_3068019, EPI_ISL_3068020, EPI_ISL_3068021, EPI_ISL_3068022, EPI_ISL_3068023, EPI_ISL_3068024, EPI_ISL_3068025, EPI_ISL_3068026, EPI_ISL_3068027, EPI_ISL_3068028, EPI_ISL_3068029, EPI_ISL_3068030, EPI_ISL_3068031, EPI_ISL_3068032, EPI_ISL_3068033, EPI_ISL_3068034, EPI_ISL_3068035, EPI_ISL_3068036, EPI_ISL_3068037, EPI_ISL_3068038, EPI_ISL_3068039, EPI_ISL_3068040, EPI_ISL_3068041, EPI_ISL_3068042, EPI_ISL_3068043, EPI_ISL_3068044, EPI_ISL_3068045, EPI_ISL_3068046, EPI_ISL_3068047, EPI_ISL_3068048, EPI_ISL_3068049, EPI_ISL_3068050, EPI_ISL_3068051, EPI_ISL_3068052, EPI_ISL_3068053, EPI_ISL_3068054, EPI_ISL_3068055, EPI_ISL_3068056, EPI_ISL_3068057, EPI_ISL_3068058, EPI_ISL_3068059, EPI_ISL_3068060, EPI_ISL_3068061, EPI_ISL_3068062, EPI_ISL_3068063, EPI_ISL_3068064, EPI_ISL_3068065, EPI_ISL_3068066, EPI_ISL_3068067, EPI_ISL_3068068, EPI_ISL_3068069, EPI_ISL_3068070, EPI_ISL_3068071, EPI_ISL_3068072, EPI_ISL_3068073, EPI_ISL_3068074, EPI_ISL_3068075, EPI_ISL_3068076, EPI_ISL_3068077, EPI_ISL_3068078, EPI_ISL_3068079, EPI_ISL_3068080, EPI_ISL_3068081, EPI_ISL_3068082, EPI_ISL_3068083, EPI_ISL_3068084, EPI_ISL_3068085, EPI_ISL_3068086, EPI_ISL_3068087, EPI_ISL_3068088, EPI_ISL_3068089, EPI_ISL_3068090, EPI_ISL_3068091, EPI_ISL_3068092, EPI_ISL_3068093, EPI_ISL_3068094, EPI_ISL_3068095, EPI_ISL_3068096, EPI_ISL_3068097, EPI_ISL_3068098, EPI_ISL_3068099, EPI_ISL_3068100, EPI_ISL_3068101, EPI_ISL_3068102, EPI_ISL_3068103, EPI_ISL_3068104, EPI_ISL_3068105, EPI_ISL_3068106, EPI_ISL_3068107, EPI_ISL_3068108, EPI_ISL_3068109, EPI_ISL_3068110, EPI_ISL_3068111, EPI_ISL_3068112, EPI_ISL_3068113, EPI_ISL_3068114, EPI_ISL_3068115, EPI_ISL_3068116, EPI_ISL_3068117, EPI_ISL_3068118, EPI_ISL_3068119, EPI_ISL_3068120, EPI_ISL_3068121, EPI_ISL_3068122, EPI_ISL_3068123, EPI_ISL_3068124, EPI_ISL_3068125, EPI_ISL_3068126, EPI_ISL_3068127, EPI_ISL_3068128, EPI_ISL_3068129, EPI_ISL_3068130, EPI_ISL_3068131, EPI_ISL_3068132, EPI_ISL_3068133, EPI_ISL_3068134, EPI_ISL_3068135, EPI_ISL_3068136, EPI_ISL_3068137, EPI_ISL_3068138, EPI_ISL_3068139, EPI_ISL_3068140, EPI_ISL_3068141, EPI_ISL_3068142, EPI_ISL_3068143			
see above	Division of Medical Virology, National Health Laboratory Service (NHLS), Tygerberg Hospital / Stellenbosch University	Division of Medical Virology, Stellenbosch University and NHLS Tygerberg Hospital	Bronwyn Kleinhans; Gert van Zyl; Kayla Delaney; Menzi Nkosi; Susan Engelbrecht; Wolfgang Preiser
EPI_ISL_1534387, EPI_ISL_2621084, EPI_ISL_2621095	Dysselsdorp Clinic wc DDC	NHLS/UCT	Arash Iranzadeh; Bruna Galvao; Carolyn Williamson; Deelan Doolabh; Diana Hardie; Emmanuel SJ; Gert Marais; Innocent Mudau; Kruger Marais; Lynn Tyers; Marvin Hsiao; Stephen Korsman; Tegally H; de Oliveira T
EPI_ISL_1915786, EPI_ISL_1915789, EPI_ISL_1915790, EPI_ISL_1915791, EPI_ISL_1915792, EPI_ISL_1915797, EPI_ISL_1915799, EPI_ISL_1915800, EPI_ISL_1917489, EPI_ISL_1917490, EPI_ISL_1917491, EPI_ISL_1972357, EPI_ISL_1972358, EPI_ISL_1972359, EPI_ISL_1972360, EPI_ISL_1972361, EPI_ISL_1972362, EPI_ISL_1972364, EPI_ISL_2107111			
see above	EHA Clinics	National Reference Laboratory, Nigeria Centre for Disease Control	Anthony Ahumibe; Catherine Okoi; Chimaobi Chukwu; Dr Chikwe Ihekweazu; Dr Ndodo Nnaemeka; Dr Omoare Adesuyi; Grace Esebanmen; Naidoo Dhamari; Nwando Mba; Olusola Anuoluwapo Akanbi
EPI_ISL_2107113	EHA, Clinics	National Reference Laboratory, Nigeria Centre for Disease Control	Anthony Ahumibe; Catherine Okoi; Chimaobi Chukwu; Dr Chikwe Ihekweazu; Dr Ndodo Nnaemeka; Dr Omoare Adesuyi; Grace Esebanmen; Naidoo Dhamari; Nwando Mba; Olusola Anuoluwapo Akanbi
EPI_ISL_2442373	ERMELO LABORATORY	National Institute for Communicable Diseases of the National Health Laboratory Service	Amoako DG; Bhiman JN; Ismail A; Mahlangu B; Mohale T; Ntuli N; Scheepers C
EPI_ISL_2447778, EPI_ISL_2447779, EPI_ISL_2447780, EPI_ISL_2447781, EPI_ISL_2447782, EPI_ISL_2447783, EPI_ISL_2447784, EPI_ISL_2447785, EPI_ISL_2447786, EPI_ISL_2447787, EPI_ISL_2447788			
see above	East London Laboratory	National Institute for Communicable Diseases of the National Health Laboratory Service	Amoako DG; Bhiman JN; Ismail A; Mahlangu B; Mohale T; Ntuli N; Scheepers C
EPI_ISL_2272008	Eastridge Clinic	National Institute for Communicable Diseases of the National Health Laboratory Service	Amoako DG; Bhiman JN; Ismail A; Mahlangu B; Mohale T; Ntuli N; Scheepers C
EPI_ISL_2271972, EPI_ISL_2271982, EPI_ISL_2271990, EPI_ISL_2271991, EPI_ISL_2272019, EPI_ISL_2272020, EPI_ISL_2272021			
see above	Edendale Gateway Clinic	National Institute for Communicable Diseases of the National Health Laboratory Service	Amoako DG; Bhiman JN; Ismail A; Mahlangu B; Mohale T; Ntuli N; Scheepers C
EPI_ISL_2758401, EPI_ISL_2758402, EPI_ISL_2758403, EPI_ISL_2758404, EPI_ISL_2758405, EPI_ISL_2758406	Emergence des Maladies Virales, Centre Interdisciplinaire de Recherches Medicales de Franceville	Emergence des Maladies Virales, Centre Interdisciplinaire de Recherches Medicales de Franceville	A.A.; A.M.; Adegnika; Andeko; Diane, A.; Djoba Siawaya; J.-B.; J.C.; J.F.; Lekana-Douki; Mbongo-Kama, E.; Mintsa Ndong, A.; N'dilimabaka, N.; Ndeboko, B.; Ngonga Dikongo; S.E.; Zoa Assoumou, S.; Zong Minko, O.
EPI_ISL_2112529	Epidemiology of Microbial Diseases, Yale School of Public Health	Epidemiology of Microbial Diseases, Yale School of Public Health	A.E.; A.F.; Alpert, T.; Breban; Brito; C.B.; C.C.; Fauver; Grubaugh; I.M.; Iwasaki, A.; J.E.; J.R.; Kalinich; Landry; Lucas, C.; M.E.; M.I.; M.L.; N.D.; Ott; Petrone; Rothman; Vogels; Watkins
EPI_ISL_1897644, EPI_ISL_1897646, EPI_ISL_1897648, EPI_ISL_1897884, EPI_ISL_1898258, EPI_ISL_1898554, EPI_ISL_1898814, EPI_ISL_1899047, EPI_ISL_1899281, EPI_ISL_1899485, EPI_ISL_2241493, EPI_ISL_2241494, EPI_ISL_2241611, EPI_ISL_2241622			
see above	Ethiopian Biotechnology Institute (EBTI)	International Centre for Genetic Engineering and Biotechnology (ICGEB) and ARGO Open Lab for Genome Sequencing	Alessandro Marcello; Danilo Licastro; Emanuele Orsini; Getnet Hailu; Hailu Dadi; Kassahun Tesfaye; Keyru Tuki; Kominist Asmamaw; Molalegne Bitew; Simeone Dal Monego; Yakob Gebregziabher Tsegay
EPI_ISL_1627092	Everight Diagnostics	National reference Laboratory, NCDC, Gaduwa, Abuja, Nigeria	Akinpelu Afolabi; Anthony Ahumibe; Badaru Sikiru; Catherine Okoi; Celestina Obiekea; Chimaobi Chukwu; Dr Chikwe Ihekweazu; Dr Grace Esebanmen; Dr Ndodo Nnaemeka; Dr Omoare Adesuyi; Kingsley Njoku; Nwando Mba; Olusola Anuoluwapo Akanbi
EPI_ISL_1534260, EPI_ISL_1534262, EPI_ISL_1534266, EPI_ISL_1534278, EPI_ISL_1534307, EPI_ISL_1534331, EPI_ISL_1534380, EPI_ISL_1534400, EPI_ISL_1534442, EPI_ISL_1534461, EPI_ISL_1817684, EPI_ISL_1817714, EPI_ISL_2140652, EPI_ISL_2140671, EPI_ISL_2140672, EPI_ISL_2375947, EPI_ISL_2802147, EPI_ISL_2802160			
see above	False Bay Hospital wc FBH	NHLS/UCT	Arash Iranzadeh; Bruna Galvao; Carolyn Williamson; Deelan Doolabh; Diana Hardie; Emmanuel SJ; Gert Marais; Innocent Mudau; Kruger Marais; Lynn Tyers; Marvin Hsiao; Stephen Korsman; Tegally H; de Oliveira T
EPI_ISL_1654211, EPI_ISL_1654212, EPI_ISL_1654213, EPI_ISL_1654214, EPI_ISL_1654215, EPI_ISL_1654216, EPI_ISL_1654218, EPI_ISL_1671922, EPI_ISL_1671924, EPI_ISL_1671927, EPI_ISL_1671928, EPI_ISL_1671929, EPI_ISL_1671930, EPI_ISL_1671931, EPI_ISL_1840862, EPI_ISL_1840863, EPI_ISL_1840864, EPI_ISL_1840865, EPI_ISL_1854755, EPI_ISL_1854756, EPI_ISL_1854757, EPI_ISL_1854758, EPI_ISL_1854759, EPI_ISL_1854760, EPI_ISL_1854761, EPI_ISL_1854762, EPI_ISL_1854763, EPI_ISL_1854764, EPI_ISL_1854765, EPI_ISL_1854766, EPI_ISL_1854767, EPI_ISL_1854768, EPI_ISL_1854769, EPI_ISL_1854770, EPI_ISL_1854771, EPI_ISL_1854772, EPI_ISL_1854773, EPI_ISL_1854774, EPI_ISL_1854775, EPI_ISL_1854776, EPI_ISL_1854777, EPI_ISL_1854778, EPI_ISL_1854779, EPI_ISL_1854780, EPI_ISL_1854781, EPI_ISL_1854782, EPI_ISL_1854783, EPI_ISL_1854784, EPI_ISL_1854785, EPI_ISL_1854786, EPI_ISL_1854787, EPI_ISL_1854788, EPI_ISL_1854789, EPI_ISL_1854790, EPI_ISL_1854791, EPI_ISL_1854792, EPI_ISL_1854793, EPI_ISL_1857277, EPI_ISL_1857278, EPI_ISL_1857279, EPI_ISL_1857280, EPI_ISL_1857283, EPI_ISL_1857284, EPI_ISL_1857285, EPI_ISL_1857286			
see above	Fondation Congolaise pour la recherche medicale (FCRM), Francine Ntoumi	Institute of Tropical Medicine	Mfoutou Mapanguy Claujeans Chastel and Batchi-Bouyou Armel Landry; Prof. Dr. Thirumalaisamy P. Velavan; Prof. Dr. Thirumalaisamy P. Velavan and Prof. Francine Ntoumi; Prof. Francine Ntoumi; Prof. Francine Ntoumi and Prof. Dr. Thirumalaisamy P. Velavan
EPI_ISL_1265757, EPI_ISL_1265820, EPI_ISL_1265821, EPI_ISL_1265822, EPI_ISL_1265823, EPI_ISL_1265824, EPI_ISL_1265836, EPI_ISL_1265837, EPI_ISL_1265838, EPI_ISL_1265849, EPI_ISL_1265850			
see above	GHE REUNION	CNR Virus des Infections Respiratoires - France SUD	Antonin Bal; Bruno Lina; Bruno Simon; Gregory Destras; Gwendolyne Burfin; Hadrien Regue; Laurence Josset; Martine Valette; Quentin Semanas
EPI_ISL_1628844, EPI_ISL_1628845, EPI_ISL_1628846, EPI_ISL_1628847, EPI_ISL_1628848, EPI_ISL_1628855, EPI_ISL_1628856, EPI_ISL_1628857, EPI_ISL_1628858, EPI_ISL_1628859, EPI_ISL_1628915, EPI_ISL_1628916, EPI_ISL_1628917, EPI_ISL_1628926, EPI_ISL_1628927, EPI_ISL_1628928, EPI_ISL_1628929, EPI_ISL_1628930, EPI_ISL_1628931, EPI_ISL_1628932, EPI_ISL_1628933, EPI_ISL_1628934, EPI_ISL_1628935, EPI_ISL_1628936, EPI_ISL_1628937, EPI_ISL_1628938, EPI_ISL_1628939, EPI_ISL_1628940, EPI_ISL_1628941, EPI_ISL_1628942, EPI_ISL_1628943, EPI_ISL_1628944, EPI_ISL_1628945, EPI_ISL_1628946, EPI_ISL_1628947, EPI_ISL_1628948, EPI_ISL_1628949, EPI_ISL_1628951, EPI_ISL_1629014, EPI_ISL_1629015, EPI_ISL_1629016, EPI_ISL_1629017, EPI_ISL_1629018, EPI_ISL_1629019, EPI_ISL_1629020, EPI_ISL_1629029, EPI_ISL_1629030, EPI_ISL_1629031, EPI_ISL_1629075, EPI_ISL_1629076, EPI_ISL_1629077, EPI_ISL_1629078, EPI_ISL_1629079, EPI_ISL_1629080, EPI_ISL_1629081, EPI_ISL_1629082, EPI_ISL_1629083, EPI_ISL_1629084, EPI_ISL_1629085, EPI_ISL_1629086, EPI_ISL_1629087, EPI_ISL_1629088, EPI_ISL_1629089, EPI_ISL_1629090, EPI_ISL_1629091, EPI_ISL_1629092, EPI_ISL_1629093, EPI_ISL_1629094, EPI_ISL_1629095, EPI_ISL_1629096, EPI_ISL_1629097, EPI_ISL_1629098, EPI_ISL_1629099, EPI_ISL_1629100, EPI_ISL_1629101, EPI_ISL_1629108, EPI_ISL_1629109, EPI_ISL_1629110, EPI_ISL_1629111, EPI_ISL_1629112, EPI_ISL_1629113, EPI_ISL_1629114, EPI_ISL_1629116, EPI_ISL_1629117, EPI_ISL_1629118, EPI_ISL_1629119, EPI_ISL_1629120, EPI_ISL_1629121, EPI_ISL_1629122, EPI_ISL_1629123, EPI_ISL_1629124, EPI_ISL_1629165, EPI_ISL_1629166, EPI_ISL_1629167, EPI_ISL_1629168, EPI_ISL_1629169, EPI_ISL_1629172, EPI_ISL_1629173, EPI_ISL_1629180, EPI_ISL_1629181, EPI_ISL_1629182, EPI_ISL_3153612, EPI_ISL_3153622, EPI_ISL_3153688, EPI_ISL_3153692, EPI_ISL_3153698, EPI_ISL_3153793, EPI_ISL_3153794, EPI_ISL_3153796, EPI_ISL_3153824, EPI_ISL_3153825, EPI_ISL_3153826, EPI_ISL_3153827, EPI_ISL_3153874, EPI_ISL_3153875, EPI_ISL_3153876, EPI_ISL_3153877, EPI_ISL_3153878, EPI_ISL_3153993, EPI_ISL_3153994, EPI_ISL_3154092, EPI_ISL_3154093, EPI_ISL_3154094, EPI_ISL_3154095, EPI_ISL_3154096, EPI_ISL_3154373, EPI_ISL_3154374, EPI_ISL_3154375, EPI_ISL_3154376, EPI_ISL_3154377			

see above	GHER	UMR PIMIT	Dr Camille Lebarbenchon; Dr David A Wilkinson; Dr Patrick Mavingui; Magali Turpin; Marie-Alice Simbi
EPI_ISL_2779285, EPI_ISL_2779303, EPI_ISL_2779304, EPI_ISL_2779305, EPI_ISL_2779306, EPI_ISL_2779307, EPI_ISL_2779422, EPI_ISL_2779423			
see above	GK Prison Busia (Matayo)Matayos Prison	USAMRD-A, Basic Science Laboratory	Alan Lemtudo; Beth Mutai; Brian Andika; Carol Kifude; Clement Masakwe; Eric Muthanje; Esther Omuseni; Faith Sigei; Gathii Kimita; George Awinda; John Waitumbi; Josphat Nyataya; Rachel Githii; Rehema Liyai; Stephen Ochola
EPI_ISL_2779388, EPI_ISL_2779389	GK Prison Kiambu	USAMRD-A, Basic Science Laboratory	Alan Lemtudo; Beth Mutai; Brian Andika; Carol Kifude; Clement Masakwe; Eric Muthanje; Esther Omuseni; Faith Sigei; Gathii Kimita; George Awinda; John Waitumbi; Josphat Nyataya; Rachel Githii; Rehema Liyai; Stephen Ochola
EPI_ISL_2779488	GK Prison Kitengela	USAMRD-A, Basic Science Laboratory	Alan Lemtudo; Beth Mutai; Brian Andika; Carol Kifude; Clement Masakwe; Eric Muthanje; Esther Omuseni; Faith Sigei; Gathii Kimita; George Awinda; John Waitumbi; Josphat Nyataya; Rachel Githii; Rehema Liyai; Stephen Ochola
EPI_ISL_2864595, EPI_ISL_2864597, EPI_ISL_2864599, EPI_ISL_2864600, EPI_ISL_2864602, EPI_ISL_2864604, EPI_ISL_2864606, EPI_ISL_2864608, EPI_ISL_2864609, EPI_ISL_2864617, EPI_ISL_2864624, EPI_ISL_2864626, EPI_ISL_2864630, EPI_ISL_2864632, EPI_ISL_2864634, EPI_ISL_2864640, EPI_ISL_2864653, EPI_ISL_2864655, EPI_ISL_2864667, EPI_ISL_2864674, EPI_ISL_2864678, EPI_ISL_2864680, EPI_ISL_2864682			
see above	Ga East Hospital	UHAS COVID-19 Lab	John O. Gyapong and the UHAS COVID-19 Lab Team; Jones Gyamfi; Kwabena O. Duedu; Reuben Ayivor-Djanie
EPI_ISL_2932460, EPI_ISL_2932461, EPI_ISL_2932462, EPI_ISL_2932463, EPI_ISL_2932464, EPI_ISL_2932465	Genome Research Unit, Animal Health Research Institute	Genome Research Unit, Animal Health Research Institute	Ahmed, H.; El-Deeb, A.; Hagag, N.; Hamdy, M.; Shaheen, M.
EPI_ISL_1534341, EPI_ISL_1534423, EPI_ISL_1534431, EPI_ISL_1534433, EPI_ISL_1534444, EPI_ISL_2140655, EPI_ISL_2621066, EPI_ISL_2621072, EPI_ISL_2802110			
see above	George Hospital wc GRH	NHLS/UCT	Arash Iranzadeh; Bruna Galvao; Carolyn Williamson; Deelan Doolabh; Diana Hardie; Emmanuel SJ; Gert Marais; Innocent Mudau; Kruger Marais; Lynn Tyers; Marvin Hsiao; Stephen Korsman; Tegally H; de Oliveira T
EPI_ISL_2621056, EPI_ISL_2621086, EPI_ISL_3207529	Great Brak River Clinic wc GBC	NHLS/UCT	Arash Iranzadeh; Bruna Galvao; Carolyn Williamson; Deelan Doolabh; Diana Hardie; Gert Marais; Innocent Mudau; Lynn Tyers; Marvin Hsiao; Rageema Joseph; Stephen Korsman
EPI_ISL_1534247, EPI_ISL_1534248, EPI_ISL_1534249, EPI_ISL_1534250, EPI_ISL_1534251, EPI_ISL_1534252, EPI_ISL_1534253, EPI_ISL_1534254, EPI_ISL_1534255, EPI_ISL_1534256, EPI_ISL_1534257, EPI_ISL_1534258, EPI_ISL_1534259, EPI_ISL_1534268, EPI_ISL_1534283, EPI_ISL_1534291, EPI_ISL_1534302, EPI_ISL_1534304, EPI_ISL_1534306, EPI_ISL_1534310, EPI_ISL_1534313, EPI_ISL_1534315, EPI_ISL_1534316, EPI_ISL_1534317, EPI_ISL_1534318, EPI_ISL_1534319, EPI_ISL_1534320, EPI_ISL_1534322, EPI_ISL_1534323, EPI_ISL_1534324, EPI_ISL_1534325, EPI_ISL_1534326, EPI_ISL_1534345, EPI_ISL_1534346, EPI_ISL_1534347, EPI_ISL_1534348, EPI_ISL_1534349, EPI_ISL_1534350, EPI_ISL_1534351, EPI_ISL_1534352, EPI_ISL_1534362, EPI_ISL_1534364, EPI_ISL_1534366, EPI_ISL_1534369, EPI_ISL_1534376, EPI_ISL_1534377, EPI_ISL_1534382, EPI_ISL_1534389, EPI_ISL_1534390, EPI_ISL_1534391, EPI_ISL_1534392, EPI_ISL_1534393, EPI_ISL_1534394, EPI_ISL_1534406, EPI_ISL_1534407, EPI_ISL_1534408, EPI_ISL_1534411, EPI_ISL_1534412, EPI_ISL_1534413, EPI_ISL_1534414, EPI_ISL_1534415, EPI_ISL_1534416, EPI_ISL_1534450, EPI_ISL_1534451, EPI_ISL_1534452, EPI_ISL_1534453, EPI_ISL_1534455, EPI_ISL_1534457, EPI_ISL_1534459, EPI_ISL_1706553, EPI_ISL_1706554, EPI_ISL_1706555, EPI_ISL_1706556, EPI_ISL_1706557, EPI_ISL_1706558, EPI_ISL_1706559, EPI_ISL_1706560, EPI_ISL_1706561, EPI_ISL_1706562, EPI_ISL_1706563, EPI_ISL_1706564, EPI_ISL_1706565, EPI_ISL_1706566, EPI_ISL_1706567, EPI_ISL_1706568, EPI_ISL_1706569, EPI_ISL_1706570, EPI_ISL_1706571, EPI_ISL_1706572, EPI_ISL_1706573, EPI_ISL_1706574, EPI_ISL_1706575, EPI_ISL_1706576, EPI_ISL_1817557, EPI_ISL_1817665, EPI_ISL_1817668, EPI_ISL_1817683, EPI_ISL_1817686, EPI_ISL_1817694, EPI_ISL_1817750, EPI_ISL_1817751, EPI_ISL_1817753, EPI_ISL_1817754, EPI_ISL_2140675, EPI_ISL_2140676, EPI_ISL_2140677, EPI_ISL_2140678, EPI_ISL_2140679, EPI_ISL_2140680, EPI_ISL_2140681, EPI_ISL_2140683, EPI_ISL_2140684, EPI_ISL_2140685, EPI_ISL_2140686, EPI_ISL_2140687, EPI_ISL_2140688, EPI_ISL_2140689, EPI_ISL_2140690, EPI_ISL_2375952, EPI_ISL_2375953, EPI_ISL_2375954, EPI_ISL_2375955, EPI_ISL_2375956, EPI_ISL_2375957, EPI_ISL_2375958, EPI_ISL_2375960, EPI_ISL_2375961, EPI_ISL_2375962, EPI_ISL_2375963, EPI_ISL_2375964, EPI_ISL_2375965, EPI_ISL_2375966, EPI_ISL_2375967, EPI_ISL_2375968, EPI_ISL_2375969, EPI_ISL_2375970, EPI_ISL_2375971, EPI_ISL_2375972, EPI_ISL_2375973, EPI_ISL_2375974, EPI_ISL_2375975, EPI_ISL_2375979, EPI_ISL_2375980, EPI_ISL_2375984, EPI_ISL_2375985, EPI_ISL_2375986, EPI_ISL_2375987, EPI_ISL_2375988, EPI_ISL_2621058, EPI_ISL_2621098, EPI_ISL_2621102, EPI_ISL_2621105, EPI_ISL_2621108, EPI_ISL_2621109, EPI_ISL_2621110, EPI_ISL_2621111, EPI_ISL_2621112, EPI_ISL_2621113, EPI_ISL_2621114, EPI_ISL_2621115, EPI_ISL_2621116, EPI_ISL_2621117, EPI_ISL_2621118, EPI_ISL_2621119, EPI_ISL_2621120, EPI_ISL_2621122, EPI_ISL_2621123, EPI_ISL_2621124, EPI_ISL_2621125, EPI_ISL_2621126, EPI_ISL_2621127, EPI_ISL_2621128, EPI_ISL_2621129, EPI_ISL_2621130, EPI_ISL_2621131, EPI_ISL_2802111, EPI_ISL_2802131, EPI_ISL_2802132, EPI_ISL_2802134, EPI_ISL_2802135, EPI_ISL_2802136, EPI_ISL_2802137, EPI_ISL_2802138, EPI_ISL_2802139, EPI_ISL_2802140, EPI_ISL_2802141, EPI_ISL_2802142, EPI_ISL_2802150, EPI_ISL_2802151, EPI_ISL_2802152, EPI_ISL_2802153, EPI_ISL_2802154, EPI_ISL_2802155, EPI_ISL_2802156, EPI_ISL_2802157, EPI_ISL_2802158, EPI_ISL_2802164, EPI_ISL_2802165, EPI_ISL_2802166, EPI_ISL_2802167, EPI_ISL_2802169, EPI_ISL_2802170, EPI_ISL_2802171, EPI_ISL_2802172, EPI_ISL_2802173, EPI_ISL_2802174, EPI_ISL_2802175, EPI_ISL_2802176, EPI_ISL_2802177, EPI_ISL_2876939, EPI_ISL_3207531, EPI_ISL_3207533			
see above	Groote Schuur Hospital wc GSH	NHLS/UCT	Arash Iranzadeh; Bruna Galvao; Carolyn Williamson; Deelan Doolabh; Diana Hardie; Emmanuel SJ; Gert Marais; Innocent Mudau; Kruger Marais; Lynn Tyers; Marvin Hsiao; Rageema Joseph; Stephen Korsman; Tegally H; de Oliveira T
EPI_ISL_2547373, EPI_ISL_2547374, EPI_ISL_2547375, EPI_ISL_2547376, EPI_ISL_2547377, EPI_ISL_2547378, EPI_ISL_2547379, EPI_ISL_2547380, EPI_ISL_2547381, EPI_ISL_2547382, EPI_ISL_2547383, EPI_ISL_2547384, EPI_ISL_2547385, EPI_ISL_2547386, EPI_ISL_2547387, EPI_ISL_2547388, EPI_ISL_2547389, EPI_ISL_2547390, EPI_ISL_2547391, EPI_ISL_2547392, EPI_ISL_2547393, EPI_ISL_2547394, EPI_ISL_2547395, EPI_ISL_2547396, EPI_ISL_2547397, EPI_ISL_2547398, EPI_ISL_2547399, EPI_ISL_2547400, EPI_ISL_2547401, EPI_ISL_2547402, EPI_ISL_2547403, EPI_ISL_2547404, EPI_ISL_2547405, EPI_ISL_2547406, EPI_ISL_2547407, EPI_ISL_2547408			
see above	Groote Schuur Hospital wc GSH	National Health Laboratory Service/University of Cape Town (NHLS/UCT)	Arash Iranzadeh; Bruna Galvao; Carolyn Williamson; Deelan Doolabh; Diana Hardie; Gert Marais; Innocent Mudau; Lynn Tyers; Marvin Hsiao; Stephen Korsman
EPI_ISL_1534397, EPI_ISL_1534410, EPI_ISL_2375983	Guguletu CHC wc GDH	NHLS/UCT	Arash Iranzadeh; Bruna Galvao; Carolyn Williamson; Deelan Doolabh; Diana Hardie; Emmanuel SJ; Innocent Mudau; Kruger Marais; Lynn Tyers; Marvin Hsiao; Stephen Korsman; Tegally H; de Oliveira T
EPI_ISL_2827101	HELEN JOSEPH LABORATORY	National Institute for Communicable Diseases of the National Health Laboratory Service	Amoako DG; Bhiman JN; Everatt J; Ismail A; Mahlangu B; Mnguni A; Mohale T; Ntuli N; Scheepers C
EPI_ISL_1534314, EPI_ISL_1534437, EPI_ISL_2375982	Hanover Park CHC wc HPH	NHLS/UCT	Arash Iranzadeh; Bruna Galvao; Carolyn Williamson; Deelan Doolabh; Diana Hardie; Emmanuel SJ; Innocent Mudau; Kruger Marais; Lynn Tyers; Marvin Hsiao; Stephen Korsman; Tegally H; de Oliveira T
EPI_ISL_1534357, EPI_ISL_1534379, EPI_ISL_1534398	Heidelberg Clinic wc HBC	NHLS/UCT	Arash Iranzadeh; Bruna Galvao; Carolyn Williamson; Deelan Doolabh; Diana Hardie; Emmanuel SJ; Innocent Mudau; Kruger Marais; Lynn Tyers; Marvin Hsiao; Stephen Korsman; Tegally H; de Oliveira T
EPI_ISL_1534402	Heideveld CDC wc HVP	NHLS/UCT	Arash Iranzadeh; Bruna Galvao; Carolyn Williamson; Deelan Doolabh; Diana Hardie; Emmanuel SJ; Innocent Mudau; Kruger Marais; Lynn Tyers; Marvin Hsiao; Stephen Korsman; Tegally H; de Oliveira T
EPI_ISL_1534359, EPI_ISL_1534446, EPI_ISL_1534448, EPI_ISL_1817752, EPI_ISL_3207530	Heideveld Emergency Centre	NHLS/UCT	Arash Iranzadeh; Bruna Galvao; Carolyn Williamson; Deelan Doolabh; Diana Hardie; Emmanuel SJ; Gert Marais; Innocent Mudau; Kruger Marais; Lynn Tyers; Marvin Hsiao; Rageema Joseph; Stephen Korsman; Tegally H; de Oliveira T
EPI_ISL_2271973, EPI_ISL_2271974, EPI_ISL_2271983, EPI_ISL_2271995, EPI_ISL_2271996, EPI_ISL_2271997, EPI_ISL_2271998, EPI_ISL_2272001, EPI_ISL_2272002, EPI_ISL_2272003, EPI_ISL_2272005, EPI_ISL_2272006, EPI_ISL_2272017, EPI_ISL_2272022			
see above	Helen Joseph	National Institute for Communicable Diseases of the National Health Laboratory Service	Amoako DG; Bhiman JN; Ismail A; Mahlangu B; Mohale T; Ntuli N; Scheepers C
EPI_ISL_2625632, EPI_ISL_2638048, EPI_ISL_2678283, EPI_ISL_2678284, EPI_ISL_2678285, EPI_ISL_2678286, EPI_ISL_2678287, EPI_ISL_2678288, EPI_ISL_2678289, EPI_ISL_2678290, EPI_ISL_2678291, EPI_ISL_2678292, EPI_ISL_2678293, EPI_ISL_2678294, EPI_ISL_2678295, EPI_ISL_2678296, EPI_ISL_2678297, EPI_ISL_2678298, EPI_ISL_2678299, EPI_ISL_2678300, EPI_ISL_2678301, EPI_ISL_2678302, EPI_ISL_2678303, EPI_ISL_2678304, EPI_ISL_2678305, EPI_ISL_2678306, EPI_ISL_2678307, EPI_ISL_2678308, EPI_ISL_2678309, EPI_ISL_2678310, EPI_ISL_2678311, EPI_ISL_2678312, EPI_ISL_2678313, EPI_ISL_2678314, EPI_ISL_2678315, EPI_ISL_2678316, EPI_ISL_2678317, EPI_ISL_2678318			
see above	HerpeZ	Institute of Tropical Medicine	Alimuddin Zumla; Edgar Simulundu; Franklyn Egbe Nkongho; John Tembo; Kangwa Mulonga; Kwitaka Maluzi; Le Thi Kieu Linh; Lloyd Mulenga; Matthew Bates; Moses Chilufya; Nathan Kapata; Prof. Dr. Thirumalaisamy P Velavan; Sivaramakrishna Rachakonda; Sombo Fwoloshi; Srinivas Reddy Pallerla; Victor Mukonka
EPI_ISL_1534275, EPI_ISL_1534358	Hillside Clinic wc BFW	NHLS/UCT	Arash Iranzadeh; Bruna Galvao; Carolyn Williamson; Deelan Doolabh; Diana Hardie; Emmanuel SJ; Innocent Mudau; Kruger Marais; Lynn Tyers; Marvin Hsiao; Stephen Korsman; Tegally H; de Oliveira T
EPI_ISL_1259284, EPI_ISL_1259285, EPI_ISL_1259286, EPI_ISL_1259287, EPI_ISL_1259288, EPI_ISL_1259289, EPI_ISL_1259290, EPI_ISL_1259291, EPI_ISL_1259292, EPI_ISL_1259293, EPI_ISL_1259294, EPI_ISL_1259295			
see above	Hopital	National Reference Center for Viruses of Respiratory Infections, Institut Pasteur, Paris	Angela Brisebarre; Camille Capel; Combe Patrice; Etienne Simon-Lorière; Marion Barbet; Maud Vanpeene; Méline Bizard; Sylvie Behillil; Sylvie van der Werf; Vincent Enouf
EPI_ISL_1257882, EPI_ISL_1257883, EPI_ISL_1257884, EPI_ISL_1257885, EPI_ISL_1257886, EPI_ISL_1257887, EPI_ISL_1257888, EPI_ISL_1790068, EPI_ISL_1790069, EPI_ISL_1790070, EPI_ISL_1790071, EPI_ISL_1790072, EPI_ISL_1790073			
see above	Hopital Général de Douala	Institut Pasteur de Dakar	Carniel Elisabeth; Dia Ndongo; Diagne Moussa Moïse; Diallo Amadou; Diop Mamadou; Elisabeth Carniel; Faye Ousmane; Loucoubar Cheikh; Ndiaye Ndack; Njouom Richard; Sall Amadou Alpha; Sankhe Safietou
EPI_ISL_1257889, EPI_ISL_1257890, EPI_ISL_1257891, EPI_ISL_1257892, EPI_ISL_1790063, EPI_ISL_1790064, EPI_ISL_1790065, EPI_ISL_1790066, EPI_ISL_1790067			
see above	Hopital Laquintinie de Douala	Institut Pasteur de Dakar	Carniel Elisabeth; Dia Ndongo; Diagne Moussa Moïse; Diallo Amadou; Diop Mamadou; Elisabeth Carniel; Faye Ousmane; Loucoubar Cheikh; Ndiaye Ndack; Njouom Richard; Sall Amadou Alpha; Sankhe Safietou
EPI_ISL_2621096, EPI_ISL_2621107	Hornlee Clinic wc HLC	NHLS/UCT	Arash Iranzadeh; Bruna Galvao; Carolyn Williamson; Deelan Doolabh; Diana Hardie; Gert Marais; Innocent Mudau; Lynn Tyers; Marvin Hsiao; Stephen Korsman
EPI_ISL_1630255, EPI_ISL_1630256, EPI_ISL_1630257, EPI_ISL_1630258, EPI_ISL_1630259, EPI_ISL_1630260, EPI_ISL_1630261, EPI_ISL_1630262, EPI_ISL_1630263, EPI_ISL_1630264, EPI_ISL_1630265, EPI_ISL_1630266, EPI_ISL_1630267, EPI_ISL_1630268, EPI_ISL_1630269, EPI_ISL_1630270, EPI_ISL_1630271, EPI_ISL_1630272, EPI_ISL_1822607, EPI_ISL_1822608, EPI_ISL_1822609			
see above	IRESSEF	Abbott	Adbou Padane; Ambroise Ahouidi; Aminata Dia; Aminata Mboup; Ana Olivo; Anna julienne selbe Ndiaye; Barbara Harris; Cyrille Diedhiou; Gavin Cloherty; Mary Rodgers; Moustapha Mbow; Nafissatou Leye; Ndeye Diabou Diagne; Papa Alassane Diaw; Souleymane Mboup; Todd Meyer
EPI_ISL_2001061, EPI_ISL_2001062, EPI_ISL_2001063, EPI_ISL_2001064, EPI_ISL_2001065, EPI_ISL_2001066, EPI_ISL_2001067, EPI_ISL_2001068, EPI_ISL_2001080, EPI_ISL_2001081, EPI_ISL_2001082, EPI_ISL_2001083, EPI_ISL_2001084, EPI_ISL_2001085, EPI_ISL_2001086, EPI_ISL_2001087, EPI_ISL_2001088, EPI_ISL_2001089, EPI_ISL_2001090, EPI_ISL_2001091, EPI_ISL_2001092, EPI_ISL_2001093, EPI_ISL_2001094, EPI_ISL_2001095, EPI_ISL_2001096, EPI_ISL_2001097, EPI_ISL_2001098, EPI_ISL_2151338, EPI_ISL_2151339, EPI_ISL_2151340, EPI_ISL_2151341, EPI_ISL_2151342, EPI_ISL_2151343, EPI_ISL_2151344, EPI_ISL_2508370, EPI_ISL_2508371, EPI_ISL_2508380, EPI_ISL_2508381, EPI_ISL_2508382, EPI_ISL_2508383, EPI_ISL_2508384, EPI_ISL_2508387, EPI_ISL_2508388, EPI_ISL_2508389, EPI_ISL_2508390, EPI_ISL_2508391, EPI_ISL_2508392, EPI_ISL_2508393, EPI_ISL_2508394, EPI_ISL_2508395, EPI_ISL_2508396			
see above	Immunology, Noguchi Memorial Institute for Medical Research	Immunology, Noguchi Memorial Institute for Medical Research	A.W.; Adu, B.; Agbenyo; Agbodzi, B.; Appiah-Kubi, J.; Asare; Assane, H.; Attiku; Awunyo, S.; Bonney; Campbell, A.; Dagnran, A.; Dorkenoo, A.; Egyir, B.; Gevao, S.; Halatoko; Harding, D.; Issa, Z.; J.K.; K.J.; K.M.; K.O.; Kossi, K.; Kumordjie, S.; Lamboni, L.; Layibo, Y.; Maman, I.; Mohktar, Q.; Odoom; Opoku Frempong, H.; Oteng, F.; Owusu-Nyantakyi, C.; P.O.; Rogers, J.; S.B.; Sadji; Salah, D.; Salou, M.; Uche; Wurie, I.; Y.A.; Yeboah, C.
EPI_ISL_2281227, EPI_ISL_2281228, EPI_ISL_2281229, EPI_ISL_2281230, EPI_ISL_2281231, EPI_ISL_2281232, EPI_ISL_2281233, EPI_ISL_2281234, EPI_ISL_2281235, EPI_ISL_2281236, EPI_ISL_2281237, EPI_ISL_2281238, EPI_ISL_2281239, EPI_ISL_2281240, EPI_ISL_2281241, EPI_ISL_2281242, EPI_ISL_2281243, EPI_ISL_2281244, EPI_ISL_2281245, EPI_ISL_2281246, EPI_ISL_2281247, EPI_ISL_2281248, EPI_ISL_2281262			
see above	Insitut Pasteur Bangui	Institut Pasteur de Dakar	Amadou Alpha Sall; Amadou Diallo; Benjamin Selekon; Cheikh Loucoubar; Christian Malaka; Mamadou Diop; Marie-Astrid Vernet; Moussa Moïse Diagne; Ndack Ndiaye; Ndongo Dia; Ousmane Faye; Rivalyn Nakoune Yandoko; Safietou Sankhe; Sandra Garba Ouangole
EPI_ISL_1896670, EPI_ISL_1909253, EPI_ISL_1912790, EPI_ISL_1912936, EPI_ISL_1912937, EPI_ISL_1913009, EPI_ISL_1913014, EPI_ISL_1913018, EPI_ISL_1913024, EPI_ISL_1913026, EPI_ISL_1913028, EPI_ISL_1914289			
see above	Institut National d'Hygiène	Laboratoire de Biotechnologie	Abdelomunim Essabbar; Fatima El Falaki; Fatima ElFalaki; Hicham Oumzil; Lahcen Belyamani and Azeddine Ibrahimi; Lahcen Belyamani and Azeddine Ibrahimih; Mohamed Rhajaoui; Mouna Ouadghiri; Saaïd Amzazi; Tarik Aanniz
EPI_ISL_1404615, EPI_ISL_1433132, EPI_ISL_1434443, EPI_ISL_1435763, EPI_ISL_1436817, EPI_ISL_1437749, EPI_ISL_1438551, EPI_ISL_1439647, EPI_ISL_1440879			
see above	Institut National d'Hygiène	Unité Mixte Internationale TransVIHMI (UMI 233 IRD - U1175 INSERM - Université de Montpellier) IRD (Institut de recherche pour le développement)	Abla A. KONOU; Adodo SADJI; Ahidjo AYOUBA; Akoélé SILIADIN; Alassane OURO-MEDELI; Amivi EHLAN; Améyo DORKENOO; Anoumou DAGNRA; Christelle BUTEL; Déléma MABA; Eric DELAPORTE; Issaka Maman; Kokou TEGUENI; Laetitia SERRANO; Martine PEETERS; Messanh DOUFFAN; Mireille PRINCE-DAVID; Mounerou SALOU; Sidonie A.M.KAGNISSODE; Sika DOSSIM; Wembo A. HALATOKO
EPI_ISL_1415293, EPI_ISL_1415425, EPI_ISL_1415428, EPI_ISL_1416165, EPI_ISL_1416192, EPI_ISL_1416222, EPI_ISL_1442993, EPI_ISL_1443000, EPI_ISL_1443001, EPI_ISL_1443002, EPI_ISL_1443003			
see above	Institut National d'hygiène	"Unité Mixte Internationale TransVIHMI (UMI 233 IRD - U1175 INSERM - Université de Montpellier) IRD (Institut de recherche pour le développement)"	Abla A. KONOU; Adodo SADJI; Ahidjo AYOUBA; Akoélé SILIADIN; Alassane OURO-MEDELI; Amivi EHLAN; Améyo DORKENOO; Anoumou DAGNRA; Christelle BUTEL; Déléma MABA; Eric DELAPORTE; Issaka Maman; Kokou TEGUENI; Laetitia SERRANO; Martine PEETERS; Messanh DOUFFAN; Mireille PRINCE-DAVID; Mounerou SALOU; Sidonie A.M.KAGNISSODE; Sika DOSSIM; Wembo A. HALATOKO
EPI_ISL_1508953, EPI_ISL_1508958, EPI_ISL_1508960, EPI_ISL_1508988, EPI_ISL_1508998, EPI_ISL_1509002, EPI_ISL_1509095, EPI_ISL_1509097, EPI_ISL_1509099, EPI_ISL_1509101			
see above	Institut National d'hygiène	Unité Mixte Internationale TransVIHMI (UMI 233 IRD - U1175 INSERM - Université de Montpellier) IRD (Institut de recherche pour le développement)	Abla A. KONOU; Adodo SADJI; Ahidjo AYOUBA; Akoélé SILIADIN; Alassane OURO-MEDELI; Amivi EHLAN; Améyo DORKENOO; Anoumou DAGNRA; Christelle BUTEL; Déléma MABA; Eric DELAPORTE; Issaka Maman; Kokou TEGUENI; Koku AGBODEKA; Laetitia SERRANO; Martine PEETERS; Messanh DOUFFAN; Mireille PRINCE-DAVID; Mounerou SALOU; Sidonie A.M.KAGNISSODE; Sika DOSSIM; Syntyche DEVATCHAGNI; Wembo A. HALATOKO
EPI_ISL_1293351, EPI_ISL_1303031, EPI_ISL_1321604, EPI_ISL_1406144, EPI_ISL_1406177, EPI_ISL_1445275, EPI_ISL_1499727, EPI_ISL_1500086, EPI_ISL_1500151, EPI_ISL_1500948, EPI_ISL_1501268, EPI_ISL_2278873, EPI_ISL_2278896, EPI_ISL_2279498			
see above	Institut National d'Hygiène (INH)	Unité Mixte Internationale TransVIHMI (UMI 233 IRD - U1175 INSERM - Université de Montpellier)IRD (Institut de recherche pour le développement)	Abla A. KONOU; Adodo SADJI; Ahidjo AYOUBA; Akoélé SILIADIN; Alassane OURO-MEDELI; Amivi EHLAN; Améyo DORKENOO; Anoumou DAGNRA; Christelle BUTEL; Déléma MABA; Eric DELAPORTE; Issaka Maman; Kokou TEGUENI; Laetitia SERRANO; Martine PEETERS; Messanh DOUFFAN; Mireille PRINCE-DAVID; Mounerou SALOU; Sidonie A.M.KAGNISSODE; Sika DOSSIM; Wembo A. HALATOKO
EPI_ISL_2245779, EPI_ISL_2245780, EPI_ISL_2245781, EPI_ISL_2245783, EPI_ISL_2245784, EPI_ISL_2245786, EPI_ISL_2245787, EPI_ISL_2245788, EPI_ISL_2245789, EPI_ISL_2245790, EPI_ISL_2245791, EPI_ISL_2245792, EPI_ISL_2245793, EPI_ISL_2245794, EPI_ISL_2245795, EPI_ISL_2245796, EPI_ISL_2245797, EPI_ISL_2245798, EPI_ISL_2245799, EPI_ISL_2245800, EPI_ISL_2245801, EPI_ISL_2245802,			

EPI_ISL_2245803, EPI_ISL_2245804, EPI_ISL_2245805, EPI_ISL_2245806, EPI_ISL_2245807, EPI_ISL_2245808, EPI_ISL_2245809, EPI_ISL_2245810, EPI_ISL_2245811, EPI_ISL_2245812, EPI_ISL_2245813, EPI_ISL_2245814, EPI_ISL_2245815, EPI_ISL_2245816, EPI_ISL_2245817, EPI_ISL_2245818, EPI_ISL_2245819, EPI_ISL_2245820, EPI_ISL_2245821, EPI_ISL_2245822, EPI_ISL_2245823, EPI_ISL_2245824, EPI_ISL_2245825, EPI_ISL_2245826, EPI_ISL_2245827, EPI_ISL_2245828, EPI_ISL_2245829, EPI_ISL_2245830, EPI_ISL_2245832, EPI_ISL_2245833, EPI_ISL_2245834, EPI_ISL_2245835, EPI_ISL_2245836, EPI_ISL_2245838, EPI_ISL_2245839, EPI_ISL_2245840, EPI_ISL_2245841, EPI_ISL_2245842, EPI_ISL_2245844, EPI_ISL_2245845, EPI_ISL_2245846, EPI_ISL_2245847, EPI_ISL_2245848, EPI_ISL_2245850, EPI_ISL_2245851, EPI_ISL_2245852, EPI_ISL_2245853, EPI_ISL_2245854, EPI_ISL_2245855, EPI_ISL_2245857, EPI_ISL_2245858, EPI_ISL_2245859, EPI_ISL_2245861, EPI_ISL_2245862, EPI_ISL_2245863, EPI_ISL_2245864, EPI_ISL_2245865, EPI_ISL_2245866, EPI_ISL_2245868, EPI_ISL_2245869, EPI_ISL_2245870, EPI_ISL_2245871, EPI_ISL_2245873, EPI_ISL_2245874, EPI_ISL_2245875, EPI_ISL_2245876, EPI_ISL_2245877, EPI_ISL_2245878, EPI_ISL_2245880, EPI_ISL_2245881, EPI_ISL_2245882, EPI_ISL_2245883, EPI_ISL_2245885, EPI_ISL_2245886, EPI_ISL_2245887, EPI_ISL_2245888, EPI_ISL_2245889, EPI_ISL_2245891, EPI_ISL_2245892, EPI_ISL_2245893, EPI_ISL_2245894, EPI_ISL_2245895, EPI_ISL_2245897, EPI_ISL_2245898, EPI_ISL_2245899, EPI_ISL_2245923, EPI_ISL_2245924, EPI_ISL_2245925, EPI_ISL_2245926, EPI_ISL_2245928, EPI_ISL_2245929, EPI_ISL_2245931, EPI_ISL_2245932, EPI_ISL_2245933, EPI_ISL_2245934, EPI_ISL_2245935, EPI_ISL_2245936, EPI_ISL_2245938, EPI_ISL_2245939, EPI_ISL_2245940, EPI_ISL_2245941, EPI_ISL_2245942, EPI_ISL_2245943, EPI_ISL_2245945, EPI_ISL_2245946, EPI_ISL_2245947, EPI_ISL_2245948, EPI_ISL_2245950, EPI_ISL_2245951, EPI_ISL_2245952, EPI_ISL_2245953, EPI_ISL_2245954, EPI_ISL_2245955, EPI_ISL_2245957, EPI_ISL_2245958, EPI_ISL_2245959, EPI_ISL_2245960, EPI_ISL_2245961, EPI_ISL_2245963, EPI_ISL_2245964, EPI_ISL_2245965, EPI_ISL_2245966, EPI_ISL_2245967, EPI_ISL_2245969, EPI_ISL_2245970, EPI_ISL_2245971, EPI_ISL_2245972, EPI_ISL_2245973, EPI_ISL_2245974, EPI_ISL_2245976, EPI_ISL_2245977			
see above	Institut Pasteur de Guinée	Institut Pateur de Dakar	Dia Ndongo; Diagne Moussa Moïse; Diallo Amadou; Diop Mamadou; Faye Ousmane; Grayo Solene; Loucoubar Cheikh; Maimouna Mbanne; Mbengue Safietou Sankhe; Mohamed Kane; Ndiaye Ndack; Sall Amadou Alpha; Tordo Noel
EPI_ISL_1633465, EPI_ISL_1633466, EPI_ISL_1633467, EPI_ISL_1827950	Institute for Health Research, Epidemiological Surveillance and Training (IRESSEF)	Abbott	Adbou Padane; Ambroise Ahouidi; Aminata Dia; Aminata Mboup; Ana Olivo; Anna julienne selbe Ndiaye; Barbara Harris; Cyrille Diedhiou; Gavin Cloherty; Mary Rodgers; Moustapha Mbow; Nafissatou Leye; Ndeye Diabou Diagne; Papa Alassane Diaw; Souleymane Mboup; Todd Meyer
EPI_ISL_1822602, EPI_ISL_1822603, EPI_ISL_1822604, EPI_ISL_1827855, EPI_ISL_1827856, EPI_ISL_1827857, EPI_ISL_1827858, EPI_ISL_1827859, EPI_ISL_1827860, EPI_ISL_1827861, EPI_ISL_1827862, EPI_ISL_1827863, EPI_ISL_1827864, EPI_ISL_1827865, EPI_ISL_1827866, EPI_ISL_1827867, EPI_ISL_1827868, EPI_ISL_1827869, EPI_ISL_1827870, EPI_ISL_1827871, EPI_ISL_1827872, EPI_ISL_1827873, EPI_ISL_1827874, EPI_ISL_1827875, EPI_ISL_1827876, EPI_ISL_1827877, EPI_ISL_1827878, EPI_ISL_1827879, EPI_ISL_1827880, EPI_ISL_1827881, EPI_ISL_1827882, EPI_ISL_1827883, EPI_ISL_1827884, EPI_ISL_1827885, EPI_ISL_1827886, EPI_ISL_1827887, EPI_ISL_1827888, EPI_ISL_1827889, EPI_ISL_1827890, EPI_ISL_1827891, EPI_ISL_1827892, EPI_ISL_1827893, EPI_ISL_1827894, EPI_ISL_1827895, EPI_ISL_1827896, EPI_ISL_1827897, EPI_ISL_1827898, EPI_ISL_1827899, EPI_ISL_1827900, EPI_ISL_1827901, EPI_ISL_1827902, EPI_ISL_1827903, EPI_ISL_1827904, EPI_ISL_1827905, EPI_ISL_1827906, EPI_ISL_1827907, EPI_ISL_1827908, EPI_ISL_1827909, EPI_ISL_1827910, EPI_ISL_1827911, EPI_ISL_1827912, EPI_ISL_1827913, EPI_ISL_1827914, EPI_ISL_1827915, EPI_ISL_1827916, EPI_ISL_1827917, EPI_ISL_1827918, EPI_ISL_1827919, EPI_ISL_1827920, EPI_ISL_1827921, EPI_ISL_1827922, EPI_ISL_1827923, EPI_ISL_1827924, EPI_ISL_1827925, EPI_ISL_1827926, EPI_ISL_1827927, EPI_ISL_1827928, EPI_ISL_1827929, EPI_ISL_1827930, EPI_ISL_1827931, EPI_ISL_1827932, EPI_ISL_1827933, EPI_ISL_1827934, EPI_ISL_1827935, EPI_ISL_1827936, EPI_ISL_1827937, EPI_ISL_1827938, EPI_ISL_1827939, EPI_ISL_1827940, EPI_ISL_1827941, EPI_ISL_1827942, EPI_ISL_1827943, EPI_ISL_1827944, EPI_ISL_1827945, EPI_ISL_1827946, EPI_ISL_1827947, EPI_ISL_1827948, EPI_ISL_1827949			
see above	Institute for Health Research, Epidemiological Surveillance and Training (IRESSEF)	Abbott Laboratories	Adbou Padane; Ambroise Ahouidi; Aminata Dia; Aminata Mboup; Ana Olivo; Anna julienne selbe Ndiaye; Barbara Harris; Cyrille Diedhiou; Gavin Cloherty; Mary Rodgers; Moustapha Mbow; Nafissatou Leye; Ndeye Diabou Diagne; Papa Alassane Diaw; Souleymane Mboup; Todd Meyer
EPI_ISL_2492550, EPI_ISL_2492555, EPI_ISL_2492559, EPI_ISL_2492571, EPI_ISL_2492572, EPI_ISL_2492574, EPI_ISL_2492576, EPI_ISL_2492585, EPI_ISL_2492594, EPI_ISL_2492597, EPI_ISL_2492598, EPI_ISL_2492599, EPI_ISL_2492603, EPI_ISL_2492611, EPI_ISL_2492621, EPI_ISL_2492635, EPI_ISL_2492646, EPI_ISL_2492649, EPI_ISL_2492668, EPI_ISL_2492674, EPI_ISL_2492695, EPI_ISL_2492702, EPI_ISL_2492713, EPI_ISL_2492714, EPI_ISL_2492731, EPI_ISL_2492744, EPI_ISL_2492752, EPI_ISL_2492756, EPI_ISL_2492759, EPI_ISL_2492761, EPI_ISL_2492762, EPI_ISL_2492766, EPI_ISL_2492767, EPI_ISL_2492769, EPI_ISL_2492776, EPI_ISL_2492777, EPI_ISL_2492782, EPI_ISL_2492785, EPI_ISL_2492790, EPI_ISL_2492791, EPI_ISL_2492792, EPI_ISL_2492797, EPI_ISL_2492807, EPI_ISL_2492810, EPI_ISL_2492812, EPI_ISL_2492814, EPI_ISL_2492819, EPI_ISL_2492821, EPI_ISL_2492826, EPI_ISL_2492827, EPI_ISL_2492829, EPI_ISL_2492831, EPI_ISL_2492835, EPI_ISL_2492841, EPI_ISL_2492846, EPI_ISL_2492850, EPI_ISL_2492851, EPI_ISL_2492852, EPI_ISL_2492854, EPI_ISL_2492859, EPI_ISL_2492864, EPI_ISL_2492869, EPI_ISL_2492871, EPI_ISL_2492872, EPI_ISL_2492881, EPI_ISL_2492889, EPI_ISL_2492897, EPI_ISL_2492901, EPI_ISL_2492902, EPI_ISL_2492904, EPI_ISL_2492907, EPI_ISL_2492909, EPI_ISL_2492911, EPI_ISL_2492914, EPI_ISL_2492921, EPI_ISL_2492922, EPI_ISL_2492924, EPI_ISL_2492934, EPI_ISL_2492942, EPI_ISL_2492943, EPI_ISL_2492948, EPI_ISL_2492949, EPI_ISL_2492950, EPI_ISL_2492951, EPI_ISL_2492956, EPI_ISL_2492967, EPI_ISL_2492980, EPI_ISL_2492982, EPI_ISL_2493012, EPI_ISL_2493013, EPI_ISL_2493018, EPI_ISL_2493020, EPI_ISL_2493036, EPI_ISL_2493037, EPI_ISL_2493074, EPI_ISL_2493076, EPI_ISL_2493083, EPI_ISL_2493084, EPI_ISL_2493085, EPI_ISL_2493086, EPI_ISL_2493091, EPI_ISL_2493092, EPI_ISL_2493093, EPI_ISL_2493094, EPI_ISL_2493095, EPI_ISL_2685948, EPI_ISL_2685949, EPI_ISL_2685950, EPI_ISL_2685951, EPI_ISL_2685952, EPI_ISL_2685953, EPI_ISL_2685954, EPI_ISL_2685955, EPI_ISL_2685956, EPI_ISL_2685957, EPI_ISL_2685958, EPI_ISL_2685959, EPI_ISL_2685960, EPI_ISL_2685961, EPI_ISL_2685962, EPI_ISL_2685963, EPI_ISL_2685964, EPI_ISL_2685965, EPI_ISL_2685966, EPI_ISL_2685967, EPI_ISL_2685968, EPI_ISL_2685969, EPI_ISL_2685970, EPI_ISL_2685971, EPI_ISL_2685972, EPI_ISL_2685973, EPI_ISL_2685974, EPI_ISL_2685975, EPI_ISL_2685976, EPI_ISL_2685977, EPI_ISL_2685978, EPI_ISL_2685979, EPI_ISL_2685980, EPI_ISL_2685981, EPI_ISL_2685982, EPI_ISL_2685983, EPI_ISL_2685984, EPI_ISL_2685985, EPI_ISL_2685986, EPI_ISL_2685987, EPI_ISL_2685988, EPI_ISL_2685989, EPI_ISL_2685990, EPI_ISL_2685991, EPI_ISL_2685992, EPI_ISL_2685993, EPI_ISL_2685994, EPI_ISL_2685995, EPI_ISL_2685996, EPI_ISL_2685997, EPI_ISL_2685998, EPI_ISL_2685999, EPI_ISL_2686000, EPI_ISL_2686001, EPI_ISL_2686002, EPI_ISL_2686003, EPI_ISL_2686004, EPI_ISL_2686005, EPI_ISL_2686006			
see above	Instituto Nacional de Investigação em Saúde	CERI, Centre for Epidemic Response and Innvoation, Stellenbosch University and KRISP, KZN Research Innovation and Sequencing Platform, UKZN.	Afonso P; David K; Emmanuel SJ; Freitas RH; Giandhari J; Inglês L; Lutucuta S; Miranda J; Morais J; Mufinda M; Naidoo Y; Neto Z; Paulo A Carralero RR Paixão JP; Pereira A; Pillay S; Tegally H; Wilkinson E; de Oliveira T
EPI_ISL_1347878, EPI_ISL_1347879, EPI_ISL_1347880, EPI_ISL_1347881, EPI_ISL_1347882, EPI_ISL_1347883, EPI_ISL_1347884, EPI_ISL_1347885, EPI_ISL_1347886, EPI_ISL_1347887, EPI_ISL_1347888, EPI_ISL_1347889, EPI_ISL_1347890, EPI_ISL_1347891, EPI_ISL_1347892, EPI_ISL_1347893, EPI_ISL_1347894, EPI_ISL_1347895, EPI_ISL_1347896, EPI_ISL_1347897, EPI_ISL_1347898, EPI_ISL_1347899, EPI_ISL_1347900, EPI_ISL_1347901, EPI_ISL_1347902, EPI_ISL_1347903, EPI_ISL_1347904, EPI_ISL_1347905, EPI_ISL_1347906, EPI_ISL_1347907, EPI_ISL_1347908, EPI_ISL_1347909, EPI_ISL_1347910, EPI_ISL_1347911, EPI_ISL_1347912, EPI_ISL_1347913, EPI_ISL_1347914, EPI_ISL_1347915, EPI_ISL_1347916, EPI_ISL_1347917, EPI_ISL_1347918, EPI_ISL_1347919, EPI_ISL_1347920, EPI_ISL_1347921, EPI_ISL_1347922, EPI_ISL_1347923, EPI_ISL_1347924, EPI_ISL_1347925, EPI_ISL_1347926, EPI_ISL_1347927, EPI_ISL_1347928, EPI_ISL_1347929, EPI_ISL_1347930, EPI_ISL_1347931, EPI_ISL_1347932, EPI_ISL_1347933, EPI_ISL_1347934, EPI_ISL_1347935, EPI_ISL_1347936, EPI_ISL_1347937, EPI_ISL_1347938, EPI_ISL_1347939, EPI_ISL_1347940, EPI_ISL_1347941, EPI_ISL_1347942, EPI_ISL_1347943, EPI_ISL_1347944, EPI_ISL_1347945, EPI_ISL_1347946, EPI_ISL_1347947, EPI_ISL_1347948, EPI_ISL_1545272, EPI_ISL_1545273, EPI_ISL_1545274, EPI_ISL_1545275, EPI_ISL_1545276, EPI_ISL_1545277, EPI_ISL_1545278, EPI_ISL_1545279, EPI_ISL_1545280, EPI_ISL_1545281, EPI_ISL_1545282, EPI_ISL_1545283, EPI_ISL_1545284, EPI_ISL_1545285, EPI_ISL_1545286, EPI_ISL_1545287, EPI_ISL_1545288, EPI_ISL_1545289, EPI_ISL_1545290, EPI_ISL_1545291, EPI_ISL_1545292, EPI_ISL_1545293, EPI_ISL_1545294, EPI_ISL_1545295, EPI_ISL_1545296, EPI_ISL_1545297, EPI_ISL_1545298, EPI_ISL_1545299, EPI_ISL_1545300, EPI_ISL_1545321, EPI_ISL_1545322, EPI_ISL_1545323, EPI_ISL_1545324, EPI_ISL_1545325, EPI_ISL_1545326, EPI_ISL_1545327, EPI_ISL_1545328, EPI_ISL_1545329, EPI_ISL_1545330, EPI_ISL_1545331, EPI_ISL_1545332, EPI_ISL_1545333, EPI_ISL_1545334, EPI_ISL_1545335, EPI_ISL_1545336, EPI_ISL_1545337, EPI_ISL_1545338, EPI_ISL_1545339, EPI_ISL_1545340, EPI_ISL_1545341, EPI_ISL_1545342, EPI_ISL_1545343, EPI_ISL_1545344, EPI_ISL_1545345, EPI_ISL_1545346, EPI_ISL_1545347, EPI_ISL_1545348, EPI_ISL_1545349, EPI_ISL_1545350, EPI_ISL_1545351, EPI_ISL_1545352, EPI_ISL_1545353, EPI_ISL_1545354, EPI_ISL_1545355, EPI_ISL_1545356, EPI_ISL_1545357, EPI_ISL_1545358, EPI_ISL_1545359, EPI_ISL_1545360, EPI_ISL_1545361, EPI_ISL_1545362, EPI_ISL_1545363, EPI_ISL_1545364, EPI_ISL_1545365, EPI_ISL_1545366, EPI_ISL_1545367, EPI_ISL_1545368, EPI_ISL_1545369, EPI_ISL_1545370, EPI_ISL_1545371, EPI_ISL_1545372, EPI_ISL_1545373, EPI_ISL_1545374, EPI_ISL_1545375, EPI_ISL_1545376, EPI_ISL_1545377, EPI_ISL_1545378, EPI_ISL_1545379, EPI_ISL_1545380, EPI_ISL_1545381, EPI_ISL_1545382, EPI_ISL_1545383, EPI_ISL_1545384, EPI_ISL_1545385, EPI_ISL_1545386, EPI_ISL_1545387, EPI_ISL_1545388, EPI_ISL_1545389, EPI_ISL_2609477, EPI_ISL_2609478, EPI_ISL_2609531, EPI_ISL_2609532, EPI_ISL_2609542, EPI_ISL_2609546, EPI_ISL_2609547, EPI_ISL_2609551			
see above	Instituto Nacional de Investigação em Saúde	KRISP, KZN Research Innovation and Sequencing Platform	Afonso P; David K; Emmanuel SJ; Freitas RH; Giandhari J; Inglês L; Lutucuta S; Miranda J; Morais J; Mufinda M; Naidoo Y; Neto Z; Paulo A Carralero RR Paixão JP; Pereira A; Pillay S; Tegally H; Wilkinson E; de Oliveira T
EPI_ISL_2492927	Instituto Nacional de Saude (INS), Mozambique	CERI, Centre for Epidemic Response and Innvoation, Stellenbosch University and KRISP, KZN Research Innovation and Sequencing Platform, UKZN.	Emmanuel S; Giandhari J; Nadia Sitoe; Nalia Ismael; Nedio Mabunda; Paulo Arnaldo; Pillay S; Tegally H; Wilkinson E; de Oliveira T
EPI_ISL_2396890, EPI_ISL_2396891, EPI_ISL_2396892, EPI_ISL_2396893, EPI_ISL_2396894, EPI_ISL_2396895, EPI_ISL_2396896, EPI_ISL_2396897, EPI_ISL_2396898, EPI_ISL_2396899, EPI_ISL_2396900, EPI_ISL_2396901, EPI_ISL_2396902, EPI_ISL_2396903, EPI_ISL_2396904, EPI_ISL_2396905, EPI_ISL_2396906, EPI_ISL_2396907, EPI_ISL_2396908, EPI_ISL_2396909, EPI_ISL_2396910, EPI_ISL_2396911, EPI_ISL_2396912, EPI_ISL_2396913, EPI_ISL_2396914, EPI_ISL_2396915, EPI_ISL_2396916, EPI_ISL_2396917, EPI_ISL_2396918, EPI_ISL_2396919, EPI_ISL_2396920, EPI_ISL_2396921, EPI_ISL_2396922, EPI_ISL_2396923, EPI_ISL_2396924, EPI_ISL_2396925, EPI_ISL_2396926, EPI_ISL_2396927, EPI_ISL_2396928, EPI_ISL_2396929, EPI_ISL_2396930, EPI_ISL_2396931, EPI_ISL_2396932, EPI_ISL_2396933, EPI_ISL_2396934, EPI_ISL_2396935, EPI_ISL_2396936, EPI_ISL_2396937, EPI_ISL_2396938, EPI_ISL_2396939, EPI_ISL_2396940, EPI_ISL_2396941, EPI_ISL_2396942, EPI_ISL_2396943, EPI_ISL_2396944, EPI_ISL_2396945, EPI_ISL_2396946, EPI_ISL_2396947, EPI_ISL_2396948, EPI_ISL_2396949, EPI_ISL_2396950, EPI_ISL_2396951, EPI_ISL_2396952, EPI_ISL_2396953, EPI_ISL_2396954, EPI_ISL_2396955, EPI_ISL_2396956, EPI_ISL_2396957, EPI_ISL_2396958, EPI_ISL_2396959, EPI_ISL_2396960, EPI_ISL_2396961, EPI_ISL_2396962, EPI_ISL_2396963, EPI_ISL_2396964, EPI_ISL_2396965, EPI_ISL_2396966, EPI_ISL_2396967, EPI_ISL_2396968, EPI_ISL_2396969, EPI_ISL_2396970, EPI_ISL_2396971, EPI_ISL_2396972, EPI_ISL_2396973, EPI_ISL_2396974, EPI_ISL_2396975, EPI_ISL_2396976, EPI_ISL_2396977, EPI_ISL_2396978, EPI_ISL_2396979, EPI_ISL_2396980, EPI_ISL_2396981, EPI_ISL_2396982, EPI_ISL_2396983, EPI_ISL_2396984, EPI_ISL_2396985, EPI_ISL_2396986, EPI_ISL_2396987, EPI_ISL_2396988, EPI_ISL_2396989, EPI_ISL_2396990, EPI_ISL_2396991, EPI_ISL_2396992, EPI_ISL_2396993, EPI_ISL_2396994, EPI_ISL_2396995, EPI_ISL_2396996, EPI_ISL_2396997, EPI_ISL_2396998, EPI_ISL_2396999			
see above	Instituto Nacional de Saude (INS), Mozambique	KRISP, KZN Research Innovation and Sequencing Platform	Emmanuel S; Giandhari J; Nadia Sitoe; Nalia Ismael; Nedio Mabunda; Paulo Arnaldo; Pillay S; Tegally H; Wilkinson E; de Oliveira T
EPI_ISL_1652049, EPI_ISL_1652050, EPI_ISL_1652051, EPI_ISL_1652052, EPI_ISL_1652053, EPI_ISL_1652054, EPI_ISL_1652055, EPI_ISL_1652056, EPI_ISL_1652057, EPI_ISL_1652058, EPI_ISL_1652059, EPI_ISL_1652060, EPI_ISL_1652061, EPI_ISL_1652062, EPI_ISL_1652063, EPI_ISL_1652064, EPI_ISL_1652065, EPI_ISL_1652066, EPI_ISL_1652067, EPI_ISL_1652068, EPI_ISL_1652069, EPI_ISL_1652070, EPI_ISL_1652071, EPI_ISL_1652072, EPI_ISL_1652073, EPI_ISL_1655961, EPI_ISL_1655962, EPI_ISL_1655963			
see above	Integrated Biorepository of H3Africa Uganda – IBRH3AU	Molecular Biology Laboratory	Ashaba Fred Katabazi; Bernard Ssentalo Bagaya; David Patrick Kateete; Edgar Kigozi; Emmanuel Nasinghe; Eric Katagirya; Gerald Mboowa; Lwanga Newton; Misaki Wayengera; Moses Joloba; Moses Luutu; Nsubuga Gideon; Rogers Kamulegeya; Samuel Kirimunda; Sarah Stanley; Savannah Mwesigwa
EPI_ISL_1697386, EPI_ISL_1697387, EPI_ISL_1697388, EPI_ISL_1697390, EPI_ISL_1697391, EPI_ISL_1697392, EPI_ISL_1697393, EPI_ISL_1697394, EPI_ISL_1697395, EPI_ISL_1697396			
see above	Iressef / LBV	Iressef Genomics lab	Abdou PADANE; Adjiratou Aissatou BA; Alioune TINE; Ambroise AHOUIDI; Aminata DIA; Aminata MBOUP; Anna Julienne Selbe NDIAYE; Assane DIENG; Astou Gaye GAYE; Barada CISSE; Birahim Piere NDIAYE; Gora LO; Khadim GUEYE; Makhtar Camara; Mengue FALL; Moustapha MBOW; Nafisatou LEYE; Ndeye Coumba Toure KANE; OUMY DIOP; Papa Alassane DIAW; Pauline SENE; SADA DIALLO; Serigne Mbaye Lo NDIAYE; Souleymane MBOUP; Yacine DIA
EPI_ISL_1261701, EPI_ISL_1261702, EPI_ISL_1261703, EPI_ISL_1261704, EPI_ISL_1261705, EPI_ISL_1261706, EPI_ISL_1261707, EPI_ISL_1261708, EPI_ISL_1261709, EPI_ISL_1261710, EPI_ISL_1261711, EPI_ISL_1261712, EPI_ISL_1261713, EPI_ISL_1261714, EPI_ISL_1261715, EPI_ISL_1261716, EPI_ISL_1261717, EPI_ISL_1261718, EPI_ISL_1261719, EPI_ISL_1261720, EPI_ISL_1261721, EPI_ISL_1261722, EPI_ISL_1261723, EPI_ISL_1261724, EPI_ISL_1261725, EPI_ISL_1261726, EPI_ISL_1261727, EPI_ISL_1261728, EPI_ISL_1261729, EPI_ISL_1261730, EPI_ISL_1261731, EPI_ISL_1261732, EPI_ISL_1261733, EPI_ISL_1261734, EPI_ISL_1261735, EPI_ISL_1261736, EPI_ISL_1261737, EPI_ISL_1261738, EPI_ISL_1261739, EPI_ISL_1261740, EPI_ISL_1261741, EPI_ISL_1261742, EPI_ISL_1261743, EPI_ISL_1261744, EPI_ISL_1261745, EPI_ISL_1261746, EPI_ISL_1261747, EPI_ISL_1261748, EPI_ISL_1261749, EPI_ISL_1261750, EPI_ISL_1261751, EPI_ISL_1261752, EPI_ISL_1261753, EPI_ISL_1261754, EPI_ISL_1261755, EPI_ISL_1261756, EPI_ISL_1261757, EPI_ISL_1261758, EPI_ISL_1261759, EPI_ISL_1261760, EPI_ISL_1261761, EPI_ISL_1261762, EPI_ISL_1261763, EPI_ISL_1261764, EPI_ISL_1261765, EPI_ISL_1261766, EPI_ISL_1261767, EPI_ISL_1261768, EPI_ISL_1261769, EPI_ISL_1261770, EPI_ISL_1261771, EPI_ISL_1261772, EPI_ISL_1261773, EPI_ISL_1261774, EPI_ISL_1910385			
see above	Iressef Genomics lab	IRESSEF	Abdou PADANE; Abdoulie KANTEH; Abdul Karim SESAY; Ambroise AHOUIDI; Aminata DIA; Aminata MBOUP; Astou Gaye GAYE; Barada CISSE; Birahim Piere NDIAYE; Gora LO; Khadim GUEYE; Moustapha MBOW; Nafisatou LEYE; Ndeye Coumba Toure KANE; Papa Alassane DIAW; Souleymane MBOUP; Yacine DIA
EPI_ISL_2779373	Isebania Sub County Hospital	USAMRD-A, Basic Science Laboratory	Alan Lemtudo; Beth Mutai; Brian Andika; Carol Kifude; Clement Masakwe; Eric Muthanje; Esther Omuseni; Faith Sigei; Gathii Kimita; George Awinda; John Waitumbi; Josphat Nyataya; Rachel Githii; Rehema Liyai; Stephen Ochola
EPI_ISL_2779353, EPI_ISL_2779365, EPI_ISL_2779366, EPI_ISL_2779367, EPI_ISL_2779368, EPI_ISL_2779369, EPI_ISL_2779370, EPI_ISL_2779378, EPI_ISL_2779379, EPI_ISL_2779380, EPI_ISL_2779381, EPI_ISL_2779382, EPI_ISL_2779383, EPI_ISL_2779384, EPI_ISL_2779385, EPI_ISL_2779386			
see above	Jaramogi Oginga Odinga Teaching and Referral Hospital	USAMRD-A, Basic Science Laboratory	Alan Lemtudo; Beth Mutai; Brian Andika; Carol Kifude; Clement Masakwe; Eric Muthanje; Esther Omuseni; Faith Sigei; Gathii Kimita; George Awinda; John Waitumbi; Josphat Nyataya; Rachel Githii; Rehema Liyai; Stephen Ochola
EPI_ISL_2271952, EPI_ISL_2271953, EPI_ISL_2271955, EPI_ISL_2271956, EPI_ISL_2271957, EPI_ISL_2271964, EPI_ISL_2271965, EPI_ISL_2271971, EPI_ISL_2271977, EPI_ISL_2271978, EPI_ISL_2271981, EPI_ISL_2271984, EPI_ISL_2271986, EPI_ISL_2271987, EPI_ISL_2271988, EPI_ISL_2271989, EPI_ISL_2271994, EPI_ISL_2272000, EPI_ISL_2272004, EPI_ISL_2272018			
see above	Joubert Clinic	National Institute for Communicable Diseases of the National Health Laboratory Service	Amoako DG; Bhiman JN; Ismail A; Mahlangu B; Mohale T; Ntuli N; Scheepers C
EPI_ISL_2779455, EPI_ISL_2779456, EPI_ISL_2779457, EPI_ISL_2779458, EPI_ISL_2779459, EPI_ISL_2779460, EPI_ISL_2779461, EPI_ISL_2779462, EPI_ISL_2779463, EPI_ISL_2779464, EPI_ISL_2779465, EPI_ISL_2779466, EPI_ISL_2779544, EPI_ISL_2779546, EPI_ISL_2779547, EPI_ISL_2779548			
see above	KCRH KEMRI/DEID Surveillance Site	USAMRD-A, Basic Science Laboratory	Alan Lemtudo; Beth Mutai; Brian Andika; Carol Kifude; Clement Masakwe; Eric Muthanje; Esther Omuseni; Faith Sigei; Gathii Kimita; George Awinda; John Waitumbi; Josphat Nyataya; Rachel Githii; Rehema Liyai; Stephen Ochola
EPI_ISL_2348622, EPI_ISL_2348623, EPI_ISL_2348624, EPI_ISL_2348625, EPI_ISL_2348626, EPI_ISL_2348627, EPI_ISL_2348628, EPI_ISL_2348629, EPI_ISL_2348630, EPI_ISL_2348631, EPI_ISL_2361364, EPI_ISL_2361365			
see above	KEMRI	KEMRI & ICGEB	Alessandro Marcello; Emanuele Orsini; Eric A Lelo; Janet Majanja; Samwel L Symekher; Silvanos M Opanda; Wadegu Meshack; Wallace D Bulimo
EPI_ISL_1610600, EPI_ISL_1610645	KEMRI Center for Biotechnology Research and Development	KEMRI-Wellcome Trust Research Programme,Kilifi	Githinji G.; Matoke D.; Mburu M.W.; Mohamed K.S.; Thiongo K.; de Laurent Z.
EPI_ISL_2602765, EPI_ISL_2602766, EPI_ISL_2602767, EPI_ISL_2602768, EPI_ISL_2602769, EPI_ISL_2602770, EPI_ISL_2602771, EPI_ISL_2602772, EPI_ISL_2602773, EPI_ISL_2602774, EPI_ISL_2602775, EPI_ISL_2602776, EPI_ISL_2602777, EPI_ISL_2602778, EPI_ISL_2602779, EPI_ISL_2602780, EPI_ISL_2602781, EPI_ISL_2602782, EPI_ISL_2602783, EPI_ISL_2602784, EPI_ISL_2602785, EPI_ISL_2602786, EPI_ISL_2602787, EPI_ISL_2602788, EPI_ISL_2602789, EPI_ISL_2602790, EPI_ISL_2602791, EPI_ISL_2602792, EPI_ISL_2602793, EPI_ISL_2602794, EPI_ISL_2602795, EPI_ISL_2602796, EPI_ISL_2602797, EPI_ISL_2602798, EPI_ISL_2602799, EPI_ISL_2602800, EPI_ISL_2602801, EPI_ISL_2602802, EPI_ISL_2602803, EPI_ISL_2602804, EPI_ISL_2602805, EPI_ISL_2602806, EPI_ISL_2602807, EPI_ISL_2602808, EPI_ISL_2602809, EPI_ISL_2602810, EPI_ISL_2602811, EPI_ISL_2602812, EPI_ISL_2602813, EPI_ISL_2602814, EPI_ISL_2602815, EPI_ISL_2602816, EPI_ISL_2602817, EPI_ISL_2602818, EPI_ISL_2602819, EPI_ISL_2602820, EPI_ISL_2602821, EPI_ISL_2602822, EPI_ISL_2602823, EPI_ISL_2602824, EPI_ISL_2602825, EPI_ISL_2602826, EPI_ISL_2602827, EPI_ISL_2602828, EPI_ISL_2602829, EPI_ISL_2602830, EPI_ISL_2602831, EPI_ISL_2602833, EPI_ISL_2602834, EPI_ISL_2602835, EPI_ISL_2602836, EPI_ISL_2602837, EPI_ISL_2602838, EPI_ISL_2602839, EPI_ISL_2602840, EPI_ISL_2602841, EPI_ISL_2602846, EPI_ISL_2602856, EPI_ISL_2602860, EPI_ISL_2602865, EPI_ISL_2602869, EPI_ISL_2602874, EPI_ISL_2602877, EPI_ISL_2602883, EPI_ISL_2602887, EPI_ISL_2602891, EPI_ISL_2602894, EPI_ISL_2602895, EPI_ISL_2602896, EPI_ISL_2602897, EPI_ISL_2602898, EPI_ISL_2602899, EPI_ISL_2602900, EPI_ISL_2602901, EPI_ISL_2602902, EPI_ISL_2602903, EPI_ISL_2602904, EPI_ISL_2602905, EPI_ISL_2602906, EPI_ISL_2602907, EPI_ISL_2602908, EPI_ISL_2602909, EPI_ISL_2602910, EPI_ISL_2602911, EPI_ISL_2602912, EPI_ISL_2602913, EPI_ISL_2602914, EPI_ISL_2602915, EPI_ISL_2602916, EPI_ISL_2602917, EPI_ISL_2602918, EPI_ISL_2602919, EPI_ISL_2602920, EPI_ISL_2602921, EPI_ISL_2602922, EPI_ISL_2602923, EPI_ISL_2602924, EPI_ISL_2602925, EPI_ISL_2602926, EPI_ISL_2602927, EPI_ISL_2602928, EPI_ISL_2602929, EPI_ISL_2602930, EPI_ISL_2602931, EPI_ISL_2602932, EPI_ISL_2602933, EPI_ISL_2602934, EPI_ISL_2602935, EPI_ISL_2602936, EPI_ISL_2602937, EPI_ISL_2602938, EPI_ISL_2602939, EPI_ISL_2602940, EPI_ISL_2602941, EPI_ISL_2602942, EPI_ISL_2602943, EPI_ISL_2602944, EPI_ISL_2602945, EPI_ISL_2602946, EPI_ISL_2602947, EPI_ISL_2602948, EPI_ISL_2602949, EPI_ISL_2602950, EPI_ISL_2602951, EPI_ISL_2602952			
see above	KEMRI-CGHR	KEMRI-Wellcome Trust Research Programme,Kilifi	; Githinji G.; Matoke D.; Mburu M.W.; Mohamed K.S.; Onyango C.; de Laurent Z.
EPI_ISL_1440075, EPI_ISL_1440078, EPI_ISL_1440081, EPI_ISL_1440084, EPI_ISL_1440087, EPI_ISL_1440090, EPI_ISL_1440093, EPI_ISL_1440096, EPI_ISL_1440099, EPI_ISL_1440102, EPI_ISL_1440106, EPI_ISL_1440109, EPI_ISL_1440112, EPI_ISL_1440115, EPI_ISL_1440118, EPI_ISL_1440119, EPI_ISL_1440122, EPI_ISL_1440125, EPI_ISL_1440128, EPI_ISL_2602994, EPI_ISL_2603002, EPI_ISL_2603004, EPI_ISL_2603005, EPI_ISL_2603006, EPI_ISL_2603007, EPI_ISL_2603008, EPI_ISL_2603010, EPI_ISL_2603011, EPI_ISL_2603013, EPI_ISL_2603015, EPI_ISL_2603016, EPI_ISL_2603017, EPI_ISL_2603018, EPI_ISL_2603021, EPI_ISL_2603023, EPI_ISL_2603024, EPI_ISL_2603029, EPI_ISL_2603031, EPI_ISL_3049376, EPI_ISL_3049377, EPI_ISL_3049378, EPI_ISL_3049379, EPI_ISL_3049380, EPI_ISL_3049381, EPI_ISL_3049382, EPI_ISL_3049383, EPI_ISL_3049384, EPI_ISL_3049385, EPI_ISL_3049386, EPI_ISL_3049387, EPI_ISL_3049388, EPI_ISL_3049389, EPI_ISL_3049390, EPI_ISL_3049391, EPI_ISL_3049392, EPI_ISL_3049393, EPI_ISL_3049394, EPI_ISL_3049395, EPI_ISL_3049396, EPI_ISL_3049397, EPI_ISL_3049398, EPI_ISL_3049399, EPI_ISL_3049400, EPI_ISL_3049401, EPI_ISL_3049402, EPI_ISL_3049403, EPI_ISL_3049404, EPI_ISL_3049405, EPI_ISL_3049406, EPI_ISL_3049408, EPI_ISL_3049409, EPI_ISL_3049410, EPI_ISL_3049411, EPI_ISL_3049412, EPI_ISL_3049413, EPI_ISL_3049414, EPI_ISL_3049415, EPI_ISL_3049416, EPI_ISL_3049417, EPI_ISL_3049418, EPI_ISL_3049419, EPI_ISL_3049442, EPI_ISL_3049443, EPI_ISL_3049444, EPI_ISL_3049445, EPI_ISL_3049446, EPI_ISL_3049447, EPI_ISL_3049448, EPI_ISL_3049449, EPI_ISL_3049450, EPI_ISL_3049451, EPI_ISL_3049452, EPI_ISL_3049453, EPI_ISL_3049454, EPI_ISL_3049553, EPI_ISL_3049651, EPI_ISL_3049652, EPI_ISL_3049720, EPI_ISL_3049721, EPI_ISL_3049722, EPI_ISL_3049723, EPI_ISL_3049724, EPI_ISL_3049725, EPI_ISL_3049726, EPI_ISL_3049727, EPI_ISL_3049728, EPI_ISL_3049729, EPI_ISL_3049730, EPI_ISL_3049731, EPI_ISL_3049754, EPI_ISL_3049755			
see above	KEMRI-Wellcome Trust Research Programme,Kilifi	KEMRI-Wellcome Trust Research Programme,Kilifi	; Githinji G.; Matoke D.; Mburu M.W.; Mohamed K.S.; Onyango C.; Thiongo K.; de Laurent Z.; deLaurent Z.
EPI_ISL_1323658, EPI_ISL_1323659, EPI_ISL_1323660, EPI_ISL_1323661, EPI_ISL_1323662, EPI_ISL_1323663	KEMRI-Wellcome Trust Research Programme/KEMRI-CGMR-C Kilifi	KEMRI-Wellcome Trust Research Programme/KEMRI-CGMR-C Kilifi	Githinji G.
EPI_ISL_2779283, EPI_ISL_2779301, EPI_ISL_2779302, EPI_ISL_2779374, EPI_ISL_2779375	KEMRI/DEID Surveillance Site (Lamu County Hospital)	USAMRD-A, Basic Science Laboratory	Alan Lemtudo; Beth Mutai; Brian Andika; Carol Kifude; Clement Masakwe; Eric Muthanje; Esther Omuseni; Faith Sigei; Gathii Kimita; George Awinda; John Waitumbi; Josphat Nyataya; Rachel Githii; Rehema Liyai; Stephen Ochola
EPI_ISL_2779354	KEMRI/DEID Surveillance Site (Marigat Sub County Hospital)	USAMRD-A, Basic Science Laboratory	Alan Lemtudo; Beth Mutai; Brian Andika; Carol Kifude; Clement Masakwe; Eric Muthanje; Esther Omuseni; Faith Sigei; Gathii Kimita; George Awinda; John Waitumbi; Josphat Nyataya; Rachel Githii; Rehema Liyai; Stephen Ochola

EPI_ISL_2779355, EPI_ISL_2779356, EPI_ISL_2779357, EPI_ISL_2779358, EPI_ISL_2779359, EPI_ISL_2779360, EPI_ISL_2779361, EPI_ISL_2779362, EPI_ISL_2779363, EPI_ISL_2779364, EPI_ISL_2779376, EPI_ISL_2779377			
see above	KEMRI/DEID Surveillance Site (Mtongwe Navy Base Hospital)	USAMRD-A, Basic Science Laboratory	Alan Lemtudo; Beth Mutai; Brian Andika; Carol Kifude; Clement Masakwe; Eric Muthanje; Esther Omuseni; Faith Sigei; Gathii Kimita; George Awinda; John Waitumbi; Josphat Nyataya; Rachel Githii; Rehema Liyai; Stephen Ochola
EPI_ISL_2086933, EPI_ISL_2086934, EPI_ISL_2086935, EPI_ISL_2086936, EPI_ISL_2086937, EPI_ISL_2086938	KIMBERLEY LABORATORY	National Institute for Communicable Diseases of the National Health Laboratory Service	Amoako DG; Bhiman JN; Ismail A; Mahlangu B; Mohale T; Ntuli N; Scheepers C
EPI_ISL_2375933	Khayelethu Clinic wc KLC	NHLS/UCT	Arash Iranzadeh; Bruna Galvao; Carolyn Williamson; Deelan Doolabh; Diana Hardie; Innocent Mudau; Kruger Marais; Lynn Tyers; Marvin Hsiao; Stephen Korsman
EPI_ISL_1534360, EPI_ISL_2375944, EPI_ISL_2621097	Knysna CDC wc WLC	NHLS/UCT	Arash Iranzadeh; Bruna Galvao; Carolyn Williamson; Deelan Doolabh; Diana Hardie; Emmanuel SJ; Gert Marais; Innocent Mudau; Kruger Marais; Lynn Tyers; Marvin Hsiao; Stephen Korsman; Tegally H; de Oliveira T
EPI_ISL_1534385, EPI_ISL_1534388, EPI_ISL_1534403, EPI_ISL_1817680, EPI_ISL_2375945, EPI_ISL_2375951, EPI_ISL_2621082, EPI_ISL_2802130			
see above	Knysna Hospital wc KNY	NHLS/UCT	Arash Iranzadeh; Bruna Galvao; Carolyn Williamson; Deelan Doolabh; Diana Hardie; Emmanuel SJ; Gert Marais; Innocent Mudau; Kruger Marais; Lynn Tyers; Marvin Hsiao; Stephen Korsman; Tegally H; de Oliveira T
EPI_ISL_2140670, EPI_ISL_2375946	Kranshoek Clinic wc KSH	NHLS/UCT	Arash Iranzadeh; Bruna Galvao; Carolyn Williamson; Deelan Doolabh; Diana Hardie; Innocent Mudau; Kruger Marais; Lynn Tyers; Marvin Hsiao; Stephen Korsman
EPI_ISL_2140659, EPI_ISL_2140673	Kwanokuthula CDC wc KWA	NHLS/UCT	Arash Iranzadeh; Bruna Galvao; Carolyn Williamson; Deelan Doolabh; Diana Hardie; Innocent Mudau; Kruger Marais; Lynn Tyers; Marvin Hsiao; Stephen Korsman
EPI_ISL_2779525	LAB-BMC	USAMRD-A, Basic Science Laboratory	Alan Lemtudo; Beth Mutai; Brian Andika; Carol Kifude; Clement Masakwe; Eric Muthanje; Esther Omuseni; Faith Sigei; Gathii Kimita; George Awinda; John Waitumbi; Josphat Nyataya; Rachel Githii; Rehema Liyai; Stephen Ochola
EPI_ISL_2433705, EPI_ISL_2433706, EPI_ISL_2433707, EPI_ISL_2433708, EPI_ISL_2433709, EPI_ISL_2433710, EPI_ISL_2433711, EPI_ISL_2433712, EPI_ISL_2433713, EPI_ISL_2433714, EPI_ISL_2433715, EPI_ISL_2433716, EPI_ISL_2433717, EPI_ISL_2433718, EPI_ISL_2433719, EPI_ISL_2433720, EPI_ISL_2433721, EPI_ISL_2433722, EPI_ISL_2433723, EPI_ISL_2433724, EPI_ISL_2433777, EPI_ISL_2433778, EPI_ISL_2433779, EPI_ISL_2433780, EPI_ISL_2433781, EPI_ISL_2433782, EPI_ISL_2433783, EPI_ISL_2433784, EPI_ISL_2433785, EPI_ISL_2433786, EPI_ISL_2433787, EPI_ISL_2433788, EPI_ISL_2433789, EPI_ISL_2433790, EPI_ISL_2433791, EPI_ISL_2433792, EPI_ISL_2433793, EPI_ISL_2433794, EPI_ISL_2433795, EPI_ISL_2433850, EPI_ISL_2433851, EPI_ISL_2433852, EPI_ISL_2433853, EPI_ISL_2433854, EPI_ISL_2433855, EPI_ISL_2433856, EPI_ISL_2433857, EPI_ISL_2433858, EPI_ISL_2433859, EPI_ISL_2433860, EPI_ISL_2433861, EPI_ISL_2433862, EPI_ISL_2433863, EPI_ISL_2433864, EPI_ISL_2433865, EPI_ISL_2433866, EPI_ISL_2433867, EPI_ISL_2433868, EPI_ISL_2433869, EPI_ISL_2433870, EPI_ISL_2433871, EPI_ISL_2433872, EPI_ISL_2433873, EPI_ISL_2433874, EPI_ISL_2433875, EPI_ISL_2433876, EPI_ISL_2433877, EPI_ISL_2433878, EPI_ISL_2433879, EPI_ISL_2433880, EPI_ISL_2433881, EPI_ISL_2433882, EPI_ISL_2433883, EPI_ISL_2433884, EPI_ISL_2433885, EPI_ISL_2433886, EPI_ISL_2433887, EPI_ISL_2433888, EPI_ISL_2433889, EPI_ISL_2433890, EPI_ISL_2433891, EPI_ISL_2433892, EPI_ISL_2433893, EPI_ISL_2433895, EPI_ISL_2433896, EPI_ISL_2433897, EPI_ISL_2433898, EPI_ISL_2433899, EPI_ISL_2433900, EPI_ISL_2433901, EPI_ISL_2433903, EPI_ISL_2433904, EPI_ISL_2433905, EPI_ISL_2433906, EPI_ISL_2433907, EPI_ISL_2433908, EPI_ISL_2433909, EPI_ISL_2433910, EPI_ISL_2433911, EPI_ISL_2433912, EPI_ISL_2433913, EPI_ISL_2433914, EPI_ISL_2433915, EPI_ISL_2433916, EPI_ISL_2433917, EPI_ISL_2433918, EPI_ISL_2433919, EPI_ISL_2433920, EPI_ISL_2433922, EPI_ISL_2433923, EPI_ISL_2433924, EPI_ISL_2433925, EPI_ISL_2443995, EPI_ISL_2443996			
see above	LANCET, LABORATORIES	National Institute for Communicable Diseases of the National Health Laboratory Service	Amoako DG; Bhiman JN; Ismail A; Mahlangu B; Mohale T; Ntuli N; Scheepers C
EPI_ISL_1502112, EPI_ISL_1502567, EPI_ISL_1508821, EPI_ISL_1508849, EPI_ISL_1508890, EPI_ISL_1508894, EPI_ISL_1508896, EPI_ISL_1508897, EPI_ISL_1508916, EPI_ISL_1508941, EPI_ISL_1508944, EPI_ISL_1508945, EPI_ISL_1508947, EPI_ISL_1508949, EPI_ISL_1508956, EPI_ISL_1508957, EPI_ISL_1508959, EPI_ISL_1508961, EPI_ISL_1508962, EPI_ISL_1508989, EPI_ISL_1508990, EPI_ISL_1508991, EPI_ISL_1509096, EPI_ISL_1509098, EPI_ISL_1509100, EPI_ISL_1509102, EPI_ISL_1509273, EPI_ISL_1509638, EPI_ISL_1524650, EPI_ISL_1524700, EPI_ISL_1524703, EPI_ISL_1524708, EPI_ISL_1524713, EPI_ISL_1524717, EPI_ISL_1524720, EPI_ISL_1524723, EPI_ISL_1548504, EPI_ISL_1575296, EPI_ISL_1575297, EPI_ISL_1575298, EPI_ISL_1575299, EPI_ISL_1575310, EPI_ISL_1575436, EPI_ISL_1575441, EPI_ISL_1575442, EPI_ISL_1575443, EPI_ISL_1575444, EPI_ISL_1575479, EPI_ISL_1585941, EPI_ISL_1586897, EPI_ISL_1586899, EPI_ISL_1586900, EPI_ISL_1586901, EPI_ISL_1586902, EPI_ISL_1586903, EPI_ISL_1586904, EPI_ISL_1586905, EPI_ISL_1586906, EPI_ISL_1586907, EPI_ISL_1586954, EPI_ISL_1587256, EPI_ISL_1587257, EPI_ISL_1587448, EPI_ISL_1587449, EPI_ISL_1654210, EPI_ISL_1675094, EPI_ISL_1675141, EPI_ISL_1797819, EPI_ISL_1798543, EPI_ISL_1799071, EPI_ISL_1799444, EPI_ISL_1800014, EPI_ISL_1810378, EPI_ISL_1810830, EPI_ISL_1810924, EPI_ISL_1810927, EPI_ISL_1810928, EPI_ISL_1810929, EPI_ISL_2276075, EPI_ISL_2276077, EPI_ISL_2276078, EPI_ISL_2276079, EPI_ISL_2276080, EPI_ISL_2276119			
see above	Laboratoire Biolim/FSS/UL	Unité Mixte Internationale TransVIHMI (UMI 233 IRD - U1175 INSERM - Université de Montpellier) IRD (Institut de recherche pour le développement)	Abla A. KONOU; Adodo SADJI; Ahidjo AYOUBA; Akoélé SILIADIN; Alassane OURO-MEDELI; Amivi EHLAN; Améyo DORKENOO; Anoumou DAGNRA; Christelle BUTEL; Déléma MABA; Eric DELAPORTE; Issaka Maman; Kokou TEGUENI; Koku AGBODEKA; Laetitia SERRANO; Martine PEETERS; Messanh DOUFFAN; Mireille PRINCE-DAVID; Mounerou SALOU; Sidonie A.M.KAGNISSODE; Sika DOSSIM; Syntiche DEVATCHAGNI Adodo SADJI; Syntyche DEVATCHAGNI; Wembo A. HALATOKO
EPI_ISL_2835002, EPI_ISL_2835003, EPI_ISL_2835004	Laboratoire Central Analyse Médicale mohammedia	Functional Genomic Platform_UATRS_CNRST_Rabat	Amina Barkat; Belayachi Lamiae; Elmostafa El fahime; Hajjaji Mohammed; Hasnae Benkirane; Hassan Aguenaou; Jaafar Heikel; Marouane Melloul; Mouhcine Gardoul; Mouhssine Hemlali; Nadia Touil; Oumaima Ait Si Mohammed; Sanaa Alaoui-Amine; Taha Chouati Omar Askander
EPI_ISL_2318037, EPI_ISL_2318899	Laboratoire Central de Virologie	Laboratoire de Biotechnologie	Abdelomunim Essabbar; Amal Zouaki; Ghizlane EL Amin; Hakima Kabbaj; Lahcen Belyamani and Azeddine Ibrahimi; Mouna Ouadghiri; Myriam Seffar; Saaïd Amzazi; Tarik Aanniz
EPI_ISL_2686235, EPI_ISL_2686236, EPI_ISL_2686237, EPI_ISL_2686238, EPI_ISL_2686239, EPI_ISL_2686240, EPI_ISL_2686241, EPI_ISL_2686242, EPI_ISL_2686243, EPI_ISL_2686244, EPI_ISL_2686245, EPI_ISL_2686246, EPI_ISL_2686247, EPI_ISL_2686248, EPI_ISL_2686249, EPI_ISL_2686250, EPI_ISL_2686251, EPI_ISL_2686252, EPI_ISL_2686253, EPI_ISL_2686254, EPI_ISL_2686255, EPI_ISL_2686256, EPI_ISL_2686257, EPI_ISL_2686258, EPI_ISL_2686259, EPI_ISL_2686260, EPI_ISL_2686261, EPI_ISL_2686262, EPI_ISL_2686263, EPI_ISL_2686264, EPI_ISL_2686265, EPI_ISL_2686266, EPI_ISL_2686267, EPI_ISL_2686268, EPI_ISL_2686269, EPI_ISL_2686270, EPI_ISL_2686271, EPI_ISL_2686272			
see above	Laboratoire National de Référence pour les Fièvres Hémorragiques Virales, Centre Muraz	Centre Muraz	Ange Badjo; Arsène Zongo; Essia Belarbi; Fabian Leendertz; Grit Schubert; Jasmin Schlotterbeck; Saïdou Ouedraogo; Soumeya Ouangraoua; Thérèse Kagone
EPI_ISL_2137841, EPI_ISL_2137842, EPI_ISL_2137843, EPI_ISL_3129931, EPI_ISL_3129932	Laboratoire Professeur Daniel GAHOUMA (LPDG)	Centre de Recherches Médicales de Lambaréné (CERMEL)	Ayong More; Bertrand Lell; Bertrand Lell and Ayola Akim Adegnika; Davy Leger Mouangala; Elvyre Mbongo-Nkama; Emilio Skarwan; Georgelin Nguema Ondo; Guy Stéphane Padzys; Gédéon Prince Manouana; Jean Bernard Lekana-Douki; Joël-Fleury Djoba Siawaya; Joël-Fleury Djoba Siawaya and Ayola Akim Adegnika; Kevine Zang Ella; Ludovic Mewono; Moustapha Nzamba Maloum; Noé Patrick Mbondoukwe; Rodrigue Bikangui; Rodrigue Mintsa Nguema; Sam O'neilla Oye Bingono; Samira Zoa Assoumou; Sandrine Zeh Nfor; Srinivas reddy Pallerla; Steffen Borrmann; Thirumalaisamy P. Velavan
EPI_ISL_1760554, EPI_ISL_1760555, EPI_ISL_1936375, EPI_ISL_1936376, EPI_ISL_1936377, EPI_ISL_1936378, EPI_ISL_1936379, EPI_ISL_1936380, EPI_ISL_1936381, EPI_ISL_1936382, EPI_ISL_1936383, EPI_ISL_1936384, EPI_ISL_1936385, EPI_ISL_1936386, EPI_ISL_1936387, EPI_ISL_1936388, EPI_ISL_1936389, EPI_ISL_1936390, EPI_ISL_1936391, EPI_ISL_1936392, EPI_ISL_1936393, EPI_ISL_1936394, EPI_ISL_1936395, EPI_ISL_1936396, EPI_ISL_1936397, EPI_ISL_1936398, EPI_ISL_1936399, EPI_ISL_1936400, EPI_ISL_1936401, EPI_ISL_1936402, EPI_ISL_1936403, EPI_ISL_1936404, EPI_ISL_1936405, EPI_ISL_1936406, EPI_ISL_1936407, EPI_ISL_1936408, EPI_ISL_1936409, EPI_ISL_1936410, EPI_ISL_1936411, EPI_ISL_1936412, EPI_ISL_1936413, EPI_ISL_1936414, EPI_ISL_1936415, EPI_ISL_1936416, EPI_ISL_1936417, EPI_ISL_1936418, EPI_ISL_1936419, EPI_ISL_1936420, EPI_ISL_1936421, EPI_ISL_1936422, EPI_ISL_1936423, EPI_ISL_1936424, EPI_ISL_1936425, EPI_ISL_1936426, EPI_ISL_1936427, EPI_ISL_1936428, EPI_ISL_1936429, EPI_ISL_1936430, EPI_ISL_1936431, EPI_ISL_1936432, EPI_ISL_1936433, EPI_ISL_1936434, EPI_ISL_1936435, EPI_ISL_1936436, EPI_ISL_1936437, EPI_ISL_1936438, EPI_ISL_1936439, EPI_ISL_1936440, EPI_ISL_1936441, EPI_ISL_1936442, EPI_ISL_1936443, EPI_ISL_1936444, EPI_ISL_1936445, EPI_ISL_1936446, EPI_ISL_1936447, EPI_ISL_1936448, EPI_ISL_1936449, EPI_ISL_1936450, EPI_ISL_1936451, EPI_ISL_1936452, EPI_ISL_1936453, EPI_ISL_1936454, EPI_ISL_1936455, EPI_ISL_1936464			
see above	Laboratoire Professeur Daniel GAHOUMA (LPDG)	Centre de recherches médicales de Lambaréné (CERMEL)	Ayola A. Adegnika; Ayong Moure; Bertrand Lell; Bénédicte Ndeboko; Emilio Skarwan; Georgelin Nguema Ondo; Gédéon P. Manouana; Haruka Abe; Jiro Yasuda; Joel Fleury Djoba Siawaya; Rodrigue Bikangui; Rotimi Myrabelle Avome Houechenou; Samira Zoa-Assoumou; Yuri Ushijima
EPI_ISL_1810933, EPI_ISL_1810934, EPI_ISL_1810935, EPI_ISL_1810936, EPI_ISL_1810937, EPI_ISL_1810938, EPI_ISL_1810944, EPI_ISL_1810947, EPI_ISL_1810948, EPI_ISL_1810949, EPI_ISL_1811221, EPI_ISL_1811222, EPI_ISL_2316770			
see above	Laboratoire central de Virologie	Laboratoire de Biotechnologie	Abdelomunim Essabbar; Amal Zouaki; Ghizlane EL Amin; Hakima Kabbaj; Lahcen Belyamani and Azeddine Ibrahimi; Mouna Ouadghiri; Myriam Seffar; Saaïd Amzazi; Tarik Aanniz
EPI_ISL_2178983, EPI_ISL_2178984, EPI_ISL_2178985, EPI_ISL_2178986, EPI_ISL_2178987, EPI_ISL_2178988, EPI_ISL_2178989, EPI_ISL_2178990, EPI_ISL_2178991, EPI_ISL_2178992, EPI_ISL_2178993, EPI_ISL_2178994, EPI_ISL_2179040, EPI_ISL_2179041, EPI_ISL_2179042, EPI_ISL_2179043, EPI_ISL_2179044, EPI_ISL_2179045, EPI_ISL_2179046, EPI_ISL_2179047, EPI_ISL_2179048, EPI_ISL_2179049, EPI_ISL_2179050, EPI_ISL_2179051, EPI_ISL_2227197, EPI_ISL_2227198, EPI_ISL_2227199, EPI_ISL_2227200, EPI_ISL_2227201, EPI_ISL_2227202, EPI_ISL_2227203			
see above	Laboratoire d'analyse biomédicale du camp Ouezzin Coulibaly	Centre Muraz	Abdoul-Salam Ouedraogo; Amariane Koné; Armel Poda; Arsène Zongo; Essia Belarbi; Fabian Leendertz; Grit Schubert; Halidou Tinto; Soumeya Ouangraoua; Thérèse Kagoné; Yacouba Sawadogo; Zekiba Tarnagda
EPI_ISL_2686342, EPI_ISL_2686343, EPI_ISL_2686344, EPI_ISL_2686345, EPI_ISL_2686346, EPI_ISL_2686347, EPI_ISL_2686348, EPI_ISL_2686349, EPI_ISL_2686350, EPI_ISL_2686351, EPI_ISL_2686352, EPI_ISL_2686353, EPI_ISL_2686354, EPI_ISL_2686355, EPI_ISL_2686356, EPI_ISL_2686357			
see above	Laboratoire de Biologie Clinique, Centre Muraz	Centre Muraz	Amariane M. M. Koné; Ange Badjo; Arsène Zongo; Essia Belarbi; Fabian Leendertz; Grit Schubert; Jasmin Schlotterbeck; Soumeya Ouangraoua; Thérèse Kagone
EPI_ISL_2820509, EPI_ISL_2820514	Laboratoire de Diagnostic, Centre de Soins 1, Caisse Nationale de Sécurité Sociale (CNSS) de Djibouti	IHU Méditerranée Infection	Anthony Levasseur; Christian A. Devaux.; Christian Devaux.; Didier Raoult; Idil Salah abdillahi; Ikram Omar Osman; Iman Abdillahi Hassan; Jeremy Delerce; Linda Houhamdi; Ludivine Brechar; Mohamed Houmed Aboubaker; Philippe Colson; Pierre-Edouard Fournier; Zeinab Ali Waberi
EPI_ISL_2820520	Laboratoire de Diagnostic, Centre de Soins 1, Caisse Nationale de Sécurité Sociale (CNSS) de Djibouti,	IHU Méditerranée Infection.	Anthony Levasseur; Christian A. Devaux.; Didier Raoult; Idil Salah abdillahi; Ikram Omar Osman; Iman Abdillahi Hassan; Jeremy Delerce; Linda Houhamdi; Ludivine Brechar; Mohamed Houmed Aboubaker; Philippe Colson; Pierre-Edouard Fournier; Zeinab Ali Waberi
EPI_ISL_2820547, EPI_ISL_2820621, EPI_ISL_2820626, EPI_ISL_2820756, EPI_ISL_2820757	Laboratoire de Diagnostic, Centre de Soins 1, Caisse Nationale de Sécurité Sociale (CNSS) de Djibouti.	IHU Méditerranée Infection.	Anthony Levasseur; Christian A. Devaux.; Didier Raoult; Idil Salah abdillahi; Ikram Omar Osman; Iman Abdillahi Hassan; Jeremy Delerce; Linda Houhamdi; Ludivine Brechar; Mohamed Houmed Aboubaker; Philippe Colson; Pierre-Edouard Fournier; Zeinab Ali Waberi
EPI_ISL_2142707, EPI_ISL_2142708, EPI_ISL_2142709, EPI_ISL_2142710, EPI_ISL_2142711, EPI_ISL_2142712, EPI_ISL_2142713, EPI_ISL_2142714, EPI_ISL_2142715, EPI_ISL_2142716, EPI_ISL_2142717, EPI_ISL_2142718, EPI_ISL_2142719, EPI_ISL_2142720, EPI_ISL_2142721, EPI_ISL_2142722, EPI_ISL_2142723, EPI_ISL_2142724, EPI_ISL_2142725, EPI_ISL_2142726, EPI_ISL_2142727, EPI_ISL_2142728, EPI_ISL_2142729, EPI_ISL_2142730, EPI_ISL_2142731, EPI_ISL_2142732, EPI_ISL_2142733, EPI_ISL_2142734, EPI_ISL_2142735, EPI_ISL_2142736, EPI_ISL_2142737, EPI_ISL_2142738, EPI_ISL_2142739, EPI_ISL_2142740, EPI_ISL_2142741, EPI_ISL_2142742, EPI_ISL_2142743			
see above	Laboratoire de Microbiologie CHU Souro Sanou	Centre Muraz	Abdoul-Salam Ouedraogo; Amariane Koné; Armel Poda; Arsène Zongo; Essia Belarbi; Fabian Leendertz; Grit Schubert; Halidou Tinto; Soumeya Ouangraoua; Thérèse Kagoné; Yacouba Sawadogo; Zekiba Tarnagda
EPI_ISL_2289087, EPI_ISL_2289088, EPI_ISL_2289089, EPI_ISL_2289090, EPI_ISL_2289091, EPI_ISL_2289092, EPI_ISL_2289093, EPI_ISL_2289094, EPI_ISL_2289095, EPI_ISL_2289096, EPI_ISL_2289097, EPI_ISL_2289098, EPI_ISL_2289099, EPI_ISL_2289100, EPI_ISL_2289101, EPI_ISL_2289102, EPI_ISL_2289103, EPI_ISL_2289104, EPI_ISL_2289105, EPI_ISL_2289106, EPI_ISL_2289107, EPI_ISL_2289108, EPI_ISL_2289109, EPI_ISL_2289110, EPI_ISL_2289111, EPI_ISL_2289112, EPI_ISL_2289113, EPI_ISL_2289114, EPI_ISL_2289115, EPI_ISL_2289116, EPI_ISL_2289117, EPI_ISL_2289118, EPI_ISL_2289119, EPI_ISL_2289120, EPI_ISL_2289121, EPI_ISL_2289122, EPI_ISL_2289123, EPI_ISL_2289124, EPI_ISL_2289125, EPI_ISL_2289126, EPI_ISL_2289127, EPI_ISL_2289128, EPI_ISL_2289129, EPI_ISL_2289130			
see above	Laboratoire de Microbiologie CHU Souro Sanou	Laboratoire bacteriologie virologie CHUSS	Abdoul-Salam Ouedraogo; Abdoulie Kanteh; Abdul Sesay; Annette Erhart; Armel Poda; François Kiemdé; Halidou Tinto; Mariama Kujabi; Yacouba Sawadogo
EPI_ISL_3200860, EPI_ISL_3204262	Laboratoire de Recherche et d'Analyses Médicales de la Gendarmerie Royale	Laboratoire de Recherche et d'Analyses Médicales de la Gendarmerie Royale	Amal SOUIRI; Sanaâ LEMRISS; Saâd EL KABBAJ
EPI_ISL_1257893, EPI_ISL_1790074, EPI_ISL_1790075, EPI_ISL_1790076	Laboratoire de Référence de la TB Douala	Institut Pasteur de Dakar	Carniel Elisabeth; Dia Ndongo; Diagne Moussa Moïse; Diallo Amadou; Diop Mamadou; Elisabeth Carniel; Faye Ousmane; Loucoubar Cheikh; Ndiaye Ndack; Njouom Richard; Sall Amadou Alpha; Sankhe Safietou
EPI_ISL_2932532, EPI_ISL_2932533, EPI_ISL_2932534, EPI_ISL_2932535, EPI_ISL_2932536, EPI_ISL_2932537, EPI_ISL_2932538, EPI_ISL_2932539, EPI_ISL_2932540, EPI_ISL_2932541, EPI_ISL_2932542, EPI_ISL_2932543, EPI_ISL_2932544, EPI_ISL_2932545, EPI_ISL_2932546, EPI_ISL_2932547, EPI_ISL_2932548, EPI_ISL_2932549, EPI_ISL_2932550, EPI_ISL_2932551, EPI_ISL_2932552, EPI_ISL_2932553, EPI_ISL_2932554, EPI_ISL_2932555, EPI_ISL_2932556, EPI_ISL_2932557, EPI_ISL_2932558, EPI_ISL_2932559, EPI_ISL_2932560, EPI_ISL_2932561, EPI_ISL_2932562, EPI_ISL_2932563, EPI_ISL_2932564, EPI_ISL_2932565, EPI_ISL_2932566			
see above	Laboratoire des Fièvres Hémorragiques Virales du Benin	Institut für Virologie - Institute of Virology - Charite	Andres Moreira-Soto; Anges Yadouleton; Anna-Lena Sander; Benjamin Hounkpatin and Jan Felix Drexler; Carine Tchibozo; Christian Drosten; Dossou Ange; Eclou Sedjro; Edmilson F de Oliveira Filho; Gildas Hounkanrin; Hinson Fidelia; Keke K. René; Mamoudou Harouna Djingarey; Melchior A. Joël Aïssi; Michael Nagel; Olfert Landt; Praise Adewumi; Salifou Sourakatou; Victor Max Corman; Wendy Karen Jo; Yvette Badou
EPI_ISL_1672551, EPI_ISL_1672552, EPI_ISL_1672553, EPI_ISL_1672554, EPI_ISL_1672555, EPI_ISL_1672556, EPI_ISL_1672557, EPI_ISL_1673311, EPI_ISL_1673312, EPI_ISL_1673313, EPI_ISL_1673314, EPI_ISL_1673315, EPI_ISL_1673316, EPI_ISL_1673317, EPI_ISL_1673318, EPI_ISL_1673319, EPI_ISL_1673320, EPI_ISL_1673321, EPI_ISL_1673322, EPI_ISL_1673323, EPI_ISL_1673324, EPI_ISL_1673325, EPI_ISL_1673326, EPI_ISL_1673327, EPI_ISL_1673328, EPI_ISL_1673329, EPI_ISL_1673330, EPI_ISL_1673331, EPI_ISL_1700674, EPI_ISL_1700676, EPI_ISL_1700677, EPI_ISL_1700678, EPI_ISL_1700679, EPI_ISL_1700680, EPI_ISL_1700681, EPI_ISL_1700682, EPI_ISL_1700683, EPI_ISL_1700684, EPI_ISL_1700685, EPI_ISL_1700686, EPI_ISL_1753020, EPI_ISL_1753021, EPI_ISL_1753022, EPI_ISL_1753023, EPI_ISL_1753024, EPI_ISL_1753025, EPI_ISL_1989326, EPI_ISL_1989331, EPI_ISL_1989335, EPI_ISL_1989339, EPI_ISL_2002669, EPI_ISL_2002670, EPI_ISL_2002671, EPI_ISL_2002672, EPI_ISL_2002673, EPI_ISL_2002674, EPI_ISL_2002675, EPI_ISL_2002676, EPI_ISL_2002677, EPI_ISL_2002678, EPI_ISL_2002679, EPI_ISL_2002680, EPI_ISL_2002681, EPI_ISL_2002682, EPI_ISL_2002683, EPI_ISL_2002684, EPI_ISL_2002685, EPI_ISL_2002686, EPI_ISL_2002687			
see above	Laboratorio de Investigaciones de Baney	Swiss Tropical and Public Health Institute	Bonifacio Manguire Nlavo; Carlos Cortes; Claudia Daubenberger; Diosdado Odjama Nseng Ada; Elizabeth Nyakarungu; Guillermo Garcia; Maximilian Mpina; Mitoha Ondo O Ayekaba; Philip Wonder Phiri; Philipp Wagner; Salome Hosch; Tobias Schindler; Yahya Maidane
EPI_ISL_2150976, EPI_ISL_2150977, EPI_ISL_2150978, EPI_ISL_2150979, EPI_ISL_2150980, EPI_ISL_2150981, EPI_ISL_2150982, EPI_ISL_2150983, EPI_ISL_2150984, EPI_ISL_2150985, EPI_ISL_2150986, EPI_ISL_2150987, EPI_ISL_2150988, EPI_ISL_2150989, EPI_ISL_2150990, EPI_ISL_2150991, EPI_ISL_2150992, EPI_ISL_2150993, EPI_ISL_2150994, EPI_ISL_2150995, EPI_ISL_2150996, EPI_ISL_2150997, EPI_ISL_2150998, EPI_ISL_2150999, EPI_ISL_2158597, EPI_ISL_2158598, EPI_ISL_2158599, EPI_ISL_2158600, EPI_ISL_2158601, EPI_ISL_2158602, EPI_ISL_2158603, EPI_ISL_2158604			
see above	Laboratório de Biologia Molecular Jean Piaget	MRCG at LSHTM, Genomics lab	Abdoulie Kanteh; Abdul Karim Sesay; Adul Candé; Aicha Balde; Aladje Balde; Bakary Sanyang; Bubacar Delgado Pinto Embaló; Dabiri Damilari; Edmira Maria da Costa; Erica Luis Maria Magalhães; Faatu Cassama; Mariama Kujabi; Milanca Agostinho Cá; Paulina Joãozinho da Costa Jarra Manneh; Rei José Pereira; Rui Inndi; Sainabou Laye Ndure; Simão Tchuda Bióté
EPI_ISL_1817687	Ladismith (Alan Blyth) Hosp wc ABH	NHLS/UCT	Arash Iranzadeh; Bruna Galvao; Carolyn Williamson; Deelan Doolabh; Diana Hardie; Innocent Mudau; Kruger Marais; Lynn Tyers; Marvin Hsiao; Stephen Korsman
EPI_ISL_1534246	Ladismith (Nissenville) Clinic wc LAF	NHLS/UCT	Arash Iranzadeh; Bruna Galvao; Carolyn Williamson; Deelan Doolabh; Diana Hardie; Innocent Mudau; Kruger Marais; Lynn Tyers; Marvin Hsiao; Stephen Korsman
EPI_ISL_2779489, EPI_ISL_2779490	Lanet hospital	USAMRD-A, Basic Science Laboratory	Alan Lemtudo; Beth Mutai; Brian Andika; Carol Kifude; Clement Masakwe; Eric Muthanje; Esther Omuseni; Faith Sigei; Gathii Kimita; George Awinda; John Waitumbi; Josphat Nyataya; Rachel Githii; Rehema Liyai; Stephen Ochola
EPI_ISL_1534272, EPI_ISL_1534370,	Lentegeur Hospital wc LGH	NHLS/UCT	Arash Iranzadeh; Bruna Galvao; Carolyn Williamson; Deelan Doolabh; Diana Hardie; Emmanuel SJ; Innocent Mudau; Kruger Marais; Lynn Tyers; Marvin Hsiao; Stephen Korsman; Tegally H; de Oliveira T

EPI_ISL_1534441			
EPI_ISL_1827699, EPI_ISL_1827701, EPI_ISL_1827702, EPI_ISL_1827703	Lobamba	National Institute for Communicable Diseases of the National Health Laboratory Service	Amoako DG; Bhiman JN; Ismail A; Mahlangu B; Maphalala GP; Mohale T; Ntuli N; Scheepers C
EPI_ISL_2779450, EPI_ISL_2779451, EPI_ISL_2779452, EPI_ISL_2779481, EPI_ISL_2779482, EPI_ISL_2779486, EPI_ISL_2779536, EPI_ISL_2779537, EPI_ISL_2779538, EPI_ISL_2779539, EPI_ISL_2779540, EPI_ISL_2779541			
see above	MOH-HQ	USAMRD-A, Basic Science Laboratory	Alan Lemtudo; Beth Mutai; Brian Andika; Carol Kifude; Clement Masakwe; Eric Muthanje; Esther Omuseni; Faith Sigei; Gathii Kimita; George Awinda; John Waitumbi; Josphat Nyataya; Rachel Githii; Rehema Liyai; Stephen Ochola
EPI_ISL_2779522, EPI_ISL_2779523	MOH-HQS	USAMRD-A, Basic Science Laboratory	Alan Lemtudo; Beth Mutai; Brian Andika; Carol Kifude; Clement Masakwe; Eric Muthanje; Esther Omuseni; Faith Sigei; Gathii Kimita; George Awinda; John Waitumbi; Josphat Nyataya; Rachel Githii; Rehema Liyai; Stephen Ochola
EPI_ISL_2779350, EPI_ISL_2779351, EPI_ISL_2779398, EPI_ISL_2779399, EPI_ISL_2779434, EPI_ISL_2779435, EPI_ISL_2779436, EPI_ISL_2779437, EPI_ISL_2779438, EPI_ISL_2779439, EPI_ISL_2779440, EPI_ISL_2779441, EPI_ISL_2779442, EPI_ISL_2779443, EPI_ISL_2779444, EPI_ISL_2779445, EPI_ISL_2779446, EPI_ISL_2779447, EPI_ISL_2779448, EPI_ISL_2779453, EPI_ISL_2779454, EPI_ISL_2779467, EPI_ISL_2779468, EPI_ISL_2779469, EPI_ISL_2779470, EPI_ISL_2779471, EPI_ISL_2779472, EPI_ISL_2779473, EPI_ISL_2779475, EPI_ISL_2779476, EPI_ISL_2779477, EPI_ISL_2779478, EPI_ISL_2779479, EPI_ISL_2779480, EPI_ISL_2779491, EPI_ISL_2779549, EPI_ISL_2779550			
see above	MOH-K	USAMRD-A, Basic Science Laboratory	Alan Lemtudo; Beth Mutai; Brian Andika; Carol Kifude; Clement Masakwe; Eric Muthanje; Esther Omuseni; Faith Sigei; Gathii Kimita; George Awinda; John Waitumbi; Josphat Nyataya; Rachel Githii; Rehema Liyai; Stephen Ochola
EPI_ISL_2779526, EPI_ISL_2779527, EPI_ISL_2779528, EPI_ISL_2779529, EPI_ISL_2779530, EPI_ISL_2779531, EPI_ISL_2779532, EPI_ISL_2779533, EPI_ISL_2779534			
see above	MOH-Q	USAMRD-A, Basic Science Laboratory	Alan Lemtudo; Beth Mutai; Brian Andika; Carol Kifude; Clement Masakwe; Eric Muthanje; Esther Omuseni; Faith Sigei; Gathii Kimita; George Awinda; John Waitumbi; Josphat Nyataya; Rachel Githii; Rehema Liyai; Stephen Ochola
EPI_ISL_2346385, EPI_ISL_2346386, EPI_ISL_2346387, EPI_ISL_2346388, EPI_ISL_2346389, EPI_ISL_2346390, EPI_ISL_2346391, EPI_ISL_2346392, EPI_ISL_2346393, EPI_ISL_2346394, EPI_ISL_2346395, EPI_ISL_2346396, EPI_ISL_2346397, EPI_ISL_2346398, EPI_ISL_2346404, EPI_ISL_2346410, EPI_ISL_2346411			
see above	MRC/UVRI & LSHTM Uganda Research Unit, Central Public Health Laboratories	MRC/UVRI & LSHTM Uganda Research Unit, Central Public Health Laboratories	Dan Lule Bugembe; Isaac Sseeewanyana; Matthew Cotten; My V.T. Phan; Patrick Semanda; Pontiano Kaleebu; Susan Nabadda
EPI_ISL_1469313, EPI_ISL_1469314, EPI_ISL_1469315, EPI_ISL_1469316, EPI_ISL_1469317, EPI_ISL_1469318, EPI_ISL_1469319, EPI_ISL_1469320, EPI_ISL_1469321, EPI_ISL_1469322, EPI_ISL_1469323, EPI_ISL_1469324, EPI_ISL_1469325, EPI_ISL_1469326, EPI_ISL_1469327, EPI_ISL_1469328, EPI_ISL_1469329, EPI_ISL_1469330, EPI_ISL_1469331, EPI_ISL_1469332, EPI_ISL_1469333, EPI_ISL_1469334, EPI_ISL_1469335, EPI_ISL_1469336, EPI_ISL_1469337, EPI_ISL_1469338, EPI_ISL_1469339, EPI_ISL_1469340, EPI_ISL_1469341, EPI_ISL_1469342, EPI_ISL_1469343, EPI_ISL_1469344, EPI_ISL_1469345, EPI_ISL_1469346, EPI_ISL_1469347, EPI_ISL_1469348, EPI_ISL_1469349, EPI_ISL_1469350, EPI_ISL_1469351, EPI_ISL_1469352, EPI_ISL_1469353, EPI_ISL_1469354, EPI_ISL_1469355, EPI_ISL_1469356, EPI_ISL_1469357, EPI_ISL_1469358, EPI_ISL_1469359, EPI_ISL_1469360, EPI_ISL_1469361, EPI_ISL_1469362, EPI_ISL_1469363, EPI_ISL_1469364, EPI_ISL_1469365, EPI_ISL_1469366, EPI_ISL_1469367, EPI_ISL_1469368, EPI_ISL_1469369, EPI_ISL_1469370, EPI_ISL_1469371, EPI_ISL_1469372, EPI_ISL_1469373, EPI_ISL_1469374, EPI_ISL_1469375, EPI_ISL_1469376, EPI_ISL_1469377, EPI_ISL_1469378, EPI_ISL_1469379, EPI_ISL_1469380, EPI_ISL_1469381, EPI_ISL_1469382, EPI_ISL_1469383, EPI_ISL_1469384, EPI_ISL_1469385, EPI_ISL_1469386, EPI_ISL_1469387, EPI_ISL_1469388, EPI_ISL_1469389, EPI_ISL_1469390, EPI_ISL_1469391, EPI_ISL_1469392, EPI_ISL_1469393, EPI_ISL_1469394, EPI_ISL_1469395, EPI_ISL_1469396, EPI_ISL_1469397, EPI_ISL_1469398, EPI_ISL_1469399, EPI_ISL_1469400, EPI_ISL_1469401, EPI_ISL_1469402, EPI_ISL_1469403, EPI_ISL_1469404, EPI_ISL_1469405, EPI_ISL_1469406, EPI_ISL_1469407, EPI_ISL_1469408, EPI_ISL_1469409, EPI_ISL_1469410, EPI_ISL_1469411, EPI_ISL_1469412, EPI_ISL_1469413, EPI_ISL_1469414, EPI_ISL_1469415, EPI_ISL_1469416, EPI_ISL_1469417, EPI_ISL_1469418, EPI_ISL_1469419, EPI_ISL_1469420, EPI_ISL_1469421, EPI_ISL_1469422, EPI_ISL_1469423, EPI_ISL_1469424, EPI_ISL_1469425, EPI_ISL_1469426, EPI_ISL_1469427, EPI_ISL_1469428, EPI_ISL_1469429, EPI_ISL_1469430, EPI_ISL_1469431, EPI_ISL_1469432			
see above	MRC/UVRI & LSHTM Uganda Research Unit	Where sequence data have been generated and submitted to GISAID	Dan Lule Bugembe; Isaac Sseeewanyana; Matthew Cotten; My V.T. Phan; Patrick Semanda; Pontiano Kaleebu; Susan Nabadda
EPI_ISL_2348511	MRC/UVRI & LSHTM Uganda Research Unit, Central Public Health Laboratories	MRC/UVRI & LSHTM Uganda Research Unit, Central Public Health Laboratories	Dan Lule Bugembe; Isaac Sseeewanyana; Matthew Cotten; My V.T. Phan; Patrick Semanda; Pontiano Kaleebu; Susan Nabadda
EPI_ISL_3152064, EPI_ISL_3152066, EPI_ISL_3152069, EPI_ISL_3152070, EPI_ISL_3152071, EPI_ISL_3152072, EPI_ISL_3152073, EPI_ISL_3152074, EPI_ISL_3152075, EPI_ISL_3152076, EPI_ISL_3152077, EPI_ISL_3152078, EPI_ISL_3152079, EPI_ISL_3152080, EPI_ISL_3152081, EPI_ISL_3152083, EPI_ISL_3152084, EPI_ISL_3152085, EPI_ISL_3152086, EPI_ISL_3152087, EPI_ISL_3152088, EPI_ISL_3152089, EPI_ISL_3152091, EPI_ISL_3152092, EPI_ISL_3152093, EPI_ISL_3152094, EPI_ISL_3152097, EPI_ISL_3152098, EPI_ISL_3152099, EPI_ISL_3152100, EPI_ISL_3152101, EPI_ISL_3152102, EPI_ISL_3152103, EPI_ISL_3152104, EPI_ISL_3152105, EPI_ISL_3152106, EPI_ISL_3152107			
see above	MRCG	LBV Le Dantec	; Abdoulie Kante; Abdul Karim Cesay; Adjiratou Aissatou BA; Aminata Sileymane Thiam; Anna julienne Selbe NDiaye; Assane Dieng; Awa Ba-Diallo; Dianke Samaté; Gora Lo; Halimatou Diop Ndiaye; Jarra Manneh; Khadim Gueye; Makhtar Camara; Mbengué Fall; Moustapha Sakho; Oumy DIOP; Pasacaline Manga; Pauline Yacine Sene; Sada Diallo; Serigne Saliou Niane; ousseynou Gueye
EPI_ISL_1620193, EPI_ISL_1731545, EPI_ISL_1731548, EPI_ISL_1731551, EPI_ISL_1731554, EPI_ISL_1731557, EPI_ISL_1731560, EPI_ISL_2142748, EPI_ISL_2941575, EPI_ISL_2958637, EPI_ISL_2958638, EPI_ISL_2958639, EPI_ISL_2958640, EPI_ISL_2958641, EPI_ISL_2958648, EPI_ISL_2958649, EPI_ISL_2958650, EPI_ISL_2958651, EPI_ISL_2958652, EPI_ISL_2958653, EPI_ISL_2958654, EPI_ISL_2958655			
see above	MRCG at LSHTM Genomics lab	MRCG at LSHTM Genomics lab	Abdoulie Kanteh; Abdul Karim sesay; Bakary Sanyang; Dabiri Damilari; Damiri Damilari; Jarra Manneh; Mariama Kujabi; Sainabou laye Ndure
EPI_ISL_1936296, EPI_ISL_1936297, EPI_ISL_1936298, EPI_ISL_1936299, EPI_ISL_1936300, EPI_ISL_1936301, EPI_ISL_1936302, EPI_ISL_1936303, EPI_ISL_1936304, EPI_ISL_1936305, EPI_ISL_1936306, EPI_ISL_1936307, EPI_ISL_1936308, EPI_ISL_1936309, EPI_ISL_1936310, EPI_ISL_1936311, EPI_ISL_1936312, EPI_ISL_1936313, EPI_ISL_1936314, EPI_ISL_1936315, EPI_ISL_1936316, EPI_ISL_1936317, EPI_ISL_1936318, EPI_ISL_1936319, EPI_ISL_1936320, EPI_ISL_1936321, EPI_ISL_1936322, EPI_ISL_1936323, EPI_ISL_1936324, EPI_ISL_1936325, EPI_ISL_1936326, EPI_ISL_1936327, EPI_ISL_1936328, EPI_ISL_1936329, EPI_ISL_1936330, EPI_ISL_1936331, EPI_ISL_1936332, EPI_ISL_1936333, EPI_ISL_1936334, EPI_ISL_1936335, EPI_ISL_1936336, EPI_ISL_1936337, EPI_ISL_1936338, EPI_ISL_1936339, EPI_ISL_1936340, EPI_ISL_1936341, EPI_ISL_1936342, EPI_ISL_1936343, EPI_ISL_1936344, EPI_ISL_1936345, EPI_ISL_1936346, EPI_ISL_1936347, EPI_ISL_1936348, EPI_ISL_1936349, EPI_ISL_1936350, EPI_ISL_1936351, EPI_ISL_1936352, EPI_ISL_1936353, EPI_ISL_1936354, EPI_ISL_1936355, EPI_ISL_1936356, EPI_ISL_1936357, EPI_ISL_1936358, EPI_ISL_1936359, EPI_ISL_1936360, EPI_ISL_1936361, EPI_ISL_1936362, EPI_ISL_1936363, EPI_ISL_1936364, EPI_ISL_1936365, EPI_ISL_1936366, EPI_ISL_1936367, EPI_ISL_1936368, EPI_ISL_1936369, EPI_ISL_1936370, EPI_ISL_1936371, EPI_ISL_1936372, EPI_ISL_1936373, EPI_ISL_1936374			
see above	Main Chemical Laboratories Egypt Army	Main Chemical Laboratories Egypt Army	Laila Elsawy; Mohamed Seadawy; Mostfa Yakout; Ola Elroby; Sherine Helmy
EPI_ISL_2779292	Malaba border point	USAMRD-A, Basic Science Laboratory	Alan Lemtudo; Beth Mutai; Brian Andika; Carol Kifude; Clement Masakwe; Eric Muthanje; Esther Omuseni; Faith Sigei; Gathii Kimita; George Awinda; John Waitumbi; Josphat Nyataya; Rachel Githii; Rehema Liyai; Stephen Ochola
EPI_ISL_2709478, EPI_ISL_2709479, EPI_ISL_2709480, EPI_ISL_2709481, EPI_ISL_2709482, EPI_ISL_2709483, EPI_ISL_2709484, EPI_ISL_2709485, EPI_ISL_2709486, EPI_ISL_2709487, EPI_ISL_2709488, EPI_ISL_2709490			
see above	Malawi Liverpool Wellcome Trust CLinical Research Program	Malawi Liverpool Wellcome Trust CLinical Research Program	Catherine Anscombe; Jonathan Rigby; Kayla Barnes; Nicholas Feasey; Oscar Kanjerwa; Phillip Ashton
EPI_ISL_1827698, EPI_ISL_1827700, EPI_ISL_1827704	Manzana	National Institute for Communicable Diseases of the National Health Laboratory Service	Amoako DG; Bhiman JN; Ismail A; Mahlangu B; Maphalala GP; Mohale T; Ntuli N; Scheepers C
EPI_ISL_2271966, EPI_ISL_2271967, EPI_ISL_2271968, EPI_ISL_2271979, EPI_ISL_2271980	Matikwana Hospital	National Institute for Communicable Diseases of the National Health Laboratory Service	Amoako DG; Bhiman JN; Ismail A; Mahlangu B; Mohale T; Ntuli N; Scheepers C
EPI_ISL_3462061, EPI_ISL_3525475, EPI_ISL_3525476	Microbiology, Russian Research Anti-plague Institute 'Microbe'	Microbiology, Russian Research Anti-plague Institute 'Microbe'	A.A.; A.D.; A.V.; E.A.; E.V.; Fedorov; Katyshev; Kazorina; Keita, M.; Keita, S.; Krasnov; Kritsky; Kutyrev; Naryshkina; S.A.; Shcherbakova; Sosedova; V.V.; Y.M.
EPI_ISL_2802161	Mitchells Plain CHC wc MHC	NHLS/UCT	Arash Iranzadeh; Bruna Galvao; Carolyn Williamson; Deelan Doolabh; Diana Hardie; Gert Marais; Innocent Mudau; Lynn Tyers; Marvin Hsiao; Stephen Korsman
EPI_ISL_2271954, EPI_ISL_2271992, EPI_ISL_2271993, EPI_ISL_2272007, EPI_ISL_2272011, EPI_ISL_2272014	Mitchells Plain Hospital	National Institute for Communicable Diseases of the National Health Laboratory Service	Amoako DG; Bhiman JN; Ismail A; Mahlangu B; Mohale T; Ntuli N; Scheepers C
EPI_ISL_1534321, EPI_ISL_1534426, EPI_ISL_1534427, EPI_ISL_1534435, EPI_ISL_1534447, EPI_ISL_1817736, EPI_ISL_2140656, EPI_ISL_2140664, EPI_ISL_2140682, EPI_ISL_2375959, EPI_ISL_2375976, EPI_ISL_2375977, EPI_ISL_2375978, EPI_ISL_2621069, EPI_ISL_2621071, EPI_ISL_2621106, EPI_ISL_2802149, EPI_ISL_3207526			
see above	Mitchells Plain Hospital wc MPH	NHLS/UCT	Arash Iranzadeh; Bruna Galvao; Carolyn Williamson; Deelan Doolabh; Diana Hardie; Emmanuel SJ; Gert Marais; Innocent Mudau; Kruger Marais; Lynn Tyers; Marvin Hsiao; Rageema Joseph; Stephen Korsman; Tegally H; de Oliveira T
EPI_ISL_1365022, EPI_ISL_1365023, EPI_ISL_1365024, EPI_ISL_1365025, EPI_ISL_1365026, EPI_ISL_1365027, EPI_ISL_1365028, EPI_ISL_1365029, EPI_ISL_1365030, EPI_ISL_1365031, EPI_ISL_1365032, EPI_ISL_1365033, EPI_ISL_1365034, EPI_ISL_1365035, EPI_ISL_1367677, EPI_ISL_1367678, EPI_ISL_1367680, EPI_ISL_1367682, EPI_ISL_1367683, EPI_ISL_1367685, EPI_ISL_1367686, EPI_ISL_1367688, EPI_ISL_1367689, EPI_ISL_1367691, EPI_ISL_1367692, EPI_ISL_1367694, EPI_ISL_1367695, EPI_ISL_1367697, EPI_ISL_1367698, EPI_ISL_1367700, EPI_ISL_1367701, EPI_ISL_1367702, EPI_ISL_1367704, EPI_ISL_1663649, EPI_ISL_1663650, EPI_ISL_1663651, EPI_ISL_1663652, EPI_ISL_1663653, EPI_ISL_1663654, EPI_ISL_1663655, EPI_ISL_1663656, EPI_ISL_1663657, EPI_ISL_1663658, EPI_ISL_1663659, EPI_ISL_1663660, EPI_ISL_1663661, EPI_ISL_1663662, EPI_ISL_1663663, EPI_ISL_1663664, EPI_ISL_1663665, EPI_ISL_1663666, EPI_ISL_1663667, EPI_ISL_1663668, EPI_ISL_1663669, EPI_ISL_1663670, EPI_ISL_1663671, EPI_ISL_1663672, EPI_ISL_1663673, EPI_ISL_1663675, EPI_ISL_1663676			
see above	Molecular diagnostic unit for viral haemorrhagic fevers and emerging viruses, Bouaké CHU Laboratory	Molecular diagnostic unit for viral haemorrhagic fevers and emerging viruses, Bouaké CHU Laboratory	Adjaratou Traoré; Bamba Fatoumata Touré; Chantal Akoua-Koffi; Coulibaly Mbegnan; Diané Bamourou; Essia Belarbi; Etilé Anoh; Fabian Leendertz; Grit Schubert; Kra Ouffoué; Monemo Pacome; Oby Wayoro; Safiatou Karidioula; Soundélé Maïté
EPI_ISL_1662578, EPI_ISL_1662579, EPI_ISL_1662580, EPI_ISL_1662581, EPI_ISL_1662582, EPI_ISL_1662583, EPI_ISL_1662584, EPI_ISL_1662585, EPI_ISL_1662586, EPI_ISL_1662587, EPI_ISL_1662588, EPI_ISL_1662589, EPI_ISL_1662590, EPI_ISL_1662591, EPI_ISL_1662592			
see above	Molecular diagnostic unit for viral haemorrhagic fevers and emerging viruses, Bouaké CHU Laboratory	Project group Epidemology of Highly Pathogenic Microorganisms, Robert Koch-Institute	Adjaratou Traoré; Bamba Fatoumata Touré; Chantal Akoua-Koffi; Coulibaly Mbegnan; Diané Bamourou; Essia Belarbi; Etilé Anoh; Fabian Leendertz; Grit Schubert; Kra Ouffoué; Monemo Pacome; Safiatou Karidioula; Soundélé Maïté
EPI_ISL_1534245, EPI_ISL_1534267, EPI_ISL_2375949	Mowbray Maternity Hospital wc MMH	NHLS/UCT	Arash Iranzadeh; Bruna Galvao; Carolyn Williamson; Deelan Doolabh; Diana Hardie; Innocent Mudau; Kruger Marais; Lynn Tyers; Marvin Hsiao; Stephen Korsman
EPI_ISL_2133083, EPI_ISL_2133084, EPI_ISL_2133085, EPI_ISL_2133086, EPI_ISL_2133087, EPI_ISL_2133088, EPI_ISL_2133089, EPI_ISL_2133090, EPI_ISL_2133091, EPI_ISL_2133092			
see above	NCDC	National Reference Laboratory, Nigeria Centre for Disease Control	Anthony Ahumibe; Catherine Okoi; Chimaobi Chukwu; Dr Chikwe Ihekweazu; Dr Ndodo Nnaemeka; Dr Omoare Adesuyi; Grace Esebanmen; Kingsley Madubuike; Naidoo Dhamari; Nwando Mba; Olusola Anuoluwapo Akanbi
EPI_ISL_2770476	NHLS Charlotte Maxeke Johannesburg Academic Hospital and the University of the Witwatersrand	KRISP, KZN Research Innovation and Sequencing Platform	Bulelani Manene; Florette Treurnicht; Giandhari Jennifer; Kathleen Subramoney; Naidoo Yeshnee; Pillay Sureshnee; San James; Tegally Houriiyah; Tshabuila Derek; Wilkinson Eduan; Yajna Ramphal; de Oliveira Tulio
EPI_ISL_1272300, EPI_ISL_1272301, EPI_ISL_1272302, EPI_ISL_1272303, EPI_ISL_1272304, EPI_ISL_1272305, EPI_ISL_1272306, EPI_ISL_1272307, EPI_ISL_1272308, EPI_ISL_1272309, EPI_ISL_1818854, EPI_ISL_1818855, EPI_ISL_1818856, EPI_ISL_1818857, EPI_ISL_1818858, EPI_ISL_1818859, EPI_ISL_1818860, EPI_ISL_1818861, EPI_ISL_1818862, EPI_ISL_1818863, EPI_ISL_1818864, EPI_ISL_1818865, EPI_ISL_1818866, EPI_ISL_1818867, EPI_ISL_1818868, EPI_ISL_1818869, EPI_ISL_1818870, EPI_ISL_1818871, EPI_ISL_1818872, EPI_ISL_1818873, EPI_ISL_1818874, EPI_ISL_1818875, EPI_ISL_1818876, EPI_ISL_1818877, EPI_ISL_1818878, EPI_ISL_1818879, EPI_ISL_1818880, EPI_ISL_1818881, EPI_ISL_1818882, EPI_ISL_1818883, EPI_ISL_1818884, EPI_ISL_1818885, EPI_ISL_1818886, EPI_ISL_1818887, EPI_ISL_1818888, EPI_ISL_1818889, EPI_ISL_1818890, EPI_ISL_1818891, EPI_ISL_1818892, EPI_ISL_1818893, EPI_ISL_1818894, EPI_ISL_1818895, EPI_ISL_1818896, EPI_ISL_1818897, EPI_ISL_1818898, EPI_ISL_1818899, EPI_ISL_1818900, EPI_ISL_1818901, EPI_ISL_1818902, EPI_ISL_1818903, EPI_ISL_1818904, EPI_ISL_1818905, EPI_ISL_1818906, EPI_ISL_1818907, EPI_ISL_1818908, EPI_ISL_1818909, EPI_ISL_1818910, EPI_ISL_1818911, EPI_ISL_1818912, EPI_ISL_1818913, EPI_ISL_1818914, EPI_ISL_1818915, EPI_ISL_1818916, EPI_ISL_1818917, EPI_ISL_1818918, EPI_ISL_1818919, EPI_ISL_1818920, EPI_ISL_1818921, EPI_ISL_1818922, EPI_ISL_1818923, EPI_ISL_1818924, EPI_ISL_1818925, EPI_ISL_1818926, EPI_ISL_1818927, EPI_ISL_1818928, EPI_ISL_1818929, EPI_ISL_1818930, EPI_ISL_1818931, EPI_ISL_1818932, EPI_ISL_1818933, EPI_ISL_1818934, EPI_ISL_1818935, EPI_ISL_1818936, EPI_ISL_1818937			
see above	NHLS Universitas Academic	UFS Virology	D Goedhals; Emmanuel Ogunbayo; MM Nyaga; MT Mogotsi; P Nthiga; PA Bester; T de Oliveira
EPI_ISL_2397307	NHLS-IALCH	KRISP, KZN Research Innovation and Sequencing Platform	Bernstein M; COMMIT-KZN Team; Cele S; Giandhari J; Gosnell BI; Hanekom W; Karim F; Khan K; Lessells RJ; Manickchund N; Mlisana K; Moosa MYS; Msomi N; Pillay S; Ramphal U; San JE; Sigal A; Singh L; Tegally H; Wilkinson E; de Oliveira T
EPI_ISL_2494622	NHLS_VIRO	KRISP, KZn Research Innovation and Sequencing Platform	Baillie Vicky; Giandhari Jennifer; Madhi Shabir; Naidoo Yeshnee; Pillay Sureshnee; San James; Tegally Houriiyah; Tshabuila Derek; Wilkinson Eduan; de Oliveira Tulio; du Plessis Jeanine
EPI_ISL_2492624, EPI_ISL_2492688, EPI_ISL_2492711, EPI_ISL_2493053, EPI_ISL_2617150, EPI_ISL_2617160, EPI_ISL_2617165, EPI_ISL_2617169, EPI_ISL_2686007, EPI_ISL_2686008, EPI_ISL_2686009, EPI_ISL_2686010, EPI_ISL_2686011, EPI_ISL_2686012, EPI_ISL_2686013, EPI_ISL_2686014, EPI_ISL_2686020, EPI_ISL_2686021, EPI_ISL_2686022, EPI_ISL_2686023, EPI_ISL_2686024, EPI_ISL_2686025, EPI_ISL_2686026, EPI_ISL_2686027			
see above	National HIV Reference Laboratory, Ministry of Health, Public Health Institute of Malawi	CERI, Centre for Epidemic Response and Innvoation, Stellenbosch University and KRISP, KZN Research Innovation and Sequencing Platform, UKZN.	Auld A; Chilima B; Chiwaula M; Emmanuel SJ; Giandhari J; Kaba M; Kampira E; Kasambara W; Kim L; Lessells R; Maida A; Mvula B; Mwangomba W; Naidoo Y; Panja L; Pillay S; Tegally H; Wadonda N; Wilkinson E; de Oliveira T
EPI_ISL_1407095, EPI_ISL_1407096, EPI_ISL_1407097, EPI_ISL_1407098, EPI_ISL_1407099, EPI_ISL_1407100, EPI_ISL_1407101, EPI_ISL_1407102, EPI_ISL_1407103, EPI_ISL_1407104, EPI_ISL_1407105, EPI_ISL_1407106, EPI_ISL_1407107, EPI_ISL_1407108, EPI_ISL_1407109, EPI_ISL_1407110, EPI_ISL_1407111, EPI_ISL_1407112, EPI_ISL_1407113, EPI_ISL_1407114, EPI_ISL_1407115, EPI_ISL_1407116, EPI_ISL_1407117, EPI_ISL_1407118, EPI_ISL_1407119, EPI_ISL_1407120, EPI_ISL_1407121, EPI_ISL_1407122, EPI_ISL_1407123, EPI_ISL_1407124, EPI_ISL_1407125, EPI_ISL_1407126, EPI_ISL_1407127, EPI_ISL_1407128, EPI_ISL_1407129, EPI_ISL_1407130, EPI_ISL_1407131, EPI_ISL_1407132, EPI_ISL_1407133, EPI_ISL_1407134, EPI_ISL_1407135, EPI_ISL_1407136, EPI_ISL_1407137, EPI_ISL_1407138, EPI_ISL_1407139, EPI_ISL_1407140, EPI_ISL_1407141, EPI_ISL_1407142, EPI_ISL_1407143, EPI_ISL_1407144, EPI_ISL_1407145, EPI_ISL_1407146, EPI_ISL_1407147, EPI_ISL_1407148, EPI_ISL_1407149, EPI_ISL_1407150, EPI_ISL_1407151, EPI_ISL_1407152, EPI_ISL_1407153, EPI_ISL_1407154, EPI_ISL_1407155, EPI_ISL_1407156, EPI_ISL_1407157, EPI_ISL_1407158, EPI_ISL_1407159, EPI_ISL_1407160, EPI_ISL_1407161, EPI_ISL_1407162, EPI_ISL_1407163, EPI_ISL_1407164, EPI_ISL_1407165, EPI_ISL_1407166, EPI_ISL_1407167, EPI_ISL_1407168, EPI_ISL_1407169, EPI_ISL_1407170, EPI_ISL_1407171, EPI_ISL_1407172, EPI_ISL_1407173, EPI_ISL_1407174, EPI_ISL_1407175, EPI_ISL_1407176, EPI_ISL_1407177, EPI_ISL_1407178, EPI_ISL_1407179, EPI_ISL_1407180, EPI_ISL_1407181, EPI_ISL_1407182, EPI_ISL_1407183, EPI_ISL_1407184, EPI_ISL_1407185, EPI_ISL_1407186, EPI_ISL_1407187, EPI_ISL_1407188, EPI_ISL_1407189, EPI_ISL_1407190, EPI_ISL_1407191, EPI_ISL_1407192, EPI_ISL_1407193, EPI_ISL_1407194, EPI_ISL_1407195, EPI_ISL_1407196, EPI_ISL_1407197, EPI_ISL_1407198, EPI_ISL_1407199, EPI_ISL_1407200, EPI_ISL_1407201, EPI_ISL_1407202, EPI_ISL_1407203, EPI_ISL_1407204, EPI_ISL_1407205, EPI_ISL_1407206, EPI_ISL_1407207, EPI_ISL_1407208, EPI_ISL_1407209, EPI_ISL_1407210, EPI_ISL_1407211, EPI_ISL_1407212, EPI_ISL_1407213, EPI_ISL_1407214, EPI_ISL_1407215, EPI_ISL_1407216, EPI_ISL_1407217, EPI_ISL_1407218, EPI_ISL_1407219, EPI_ISL_1407220, EPI_ISL_1407221, EPI_ISL_1407222, EPI_ISL_1407223, EPI_ISL_1407224, EPI_ISL_1407225, EPI_ISL_1407226, EPI_ISL_1407227, EPI_ISL_1407228, EPI_ISL_1407229, EPI_ISL_1407230, EPI_ISL_1407231, EPI_ISL_1407232, EPI_ISL_1407233, EPI_ISL_1407234, EPI_ISL_1407235, EPI_ISL_1407236, EPI_ISL_1407237, EPI_ISL_1407238, EPI_ISL_1407239, EPI_ISL_1407240, EPI_ISL_1407241, EPI_ISL_1407242, EPI_ISL_1407243, EPI_ISL_1407244, EPI_ISL_1407245, EPI_ISL_1407246, EPI_ISL_1407247, EPI_ISL_1407248, EPI_ISL_1407249, EPI_ISL_1407250, EPI_ISL_1407251, EPI_ISL_1407252, EPI_ISL_1407253, EPI_ISL_2494714, EPI_ISL_2494720, EPI_ISL_2494794, EPI_ISL_2494822, EPI_ISL_2494844, EPI_ISL_2494943, EPI_ISL_2495036, EPI_ISL_2609564, EPI_ISL_2609565, EPI_ISL_2609566, EPI_ISL_2609567, EPI_ISL_2609568, EPI_ISL_2609569, EPI_ISL_2609570, EPI_ISL_2609571, EPI_ISL_2609572, EPI_ISL_2609573,			

EPI_ISL_2609574, EPI_ISL_2609575, EPI_ISL_2609576, EPI_ISL_2609577, EPI_ISL_2609578, EPI_ISL_2609579, EPI_ISL_2609580, EPI_ISL_2609581, EPI_ISL_2609582, EPI_ISL_2609583, EPI_ISL_2609584, EPI_ISL_2609585, EPI_ISL_2609586, EPI_ISL_2609587, EPI_ISL_2609588, EPI_ISL_2609589, EPI_ISL_2609590, EPI_ISL_2609591, EPI_ISL_2609592, EPI_ISL_2609596, EPI_ISL_2609597, EPI_ISL_2609598, EPI_ISL_2609599, EPI_ISL_2609600, EPI_ISL_2609601, EPI_ISL_2609602, EPI_ISL_2609603, EPI_ISL_2609604, EPI_ISL_2609605, EPI_ISL_2609606, EPI_ISL_2609607, EPI_ISL_2609608			
see above	National HIV Reference Laboratory, Ministry of Health, Public Health Institute of Malawi	KRISP, KZN Research Innovation and Sequencing Platform	Auld A; Chilima B; Chiwaula M; Emmanuel SJ; Giandhari J; Kaba M; Kampira E; Kasambara W; Kim L; Lessells R; Maida A; Mvula B; Mwangomba W; Naidoo Y; Panja L; Pillay S; Tegally H; Wadonda N; Wilkinson E; de Oliveira T
EPI_ISL_1677699, EPI_ISL_1677700, EPI_ISL_1677701, EPI_ISL_1677702, EPI_ISL_1677703, EPI_ISL_1677704, EPI_ISL_1677705, EPI_ISL_1677706, EPI_ISL_1677707, EPI_ISL_1677708, EPI_ISL_1677709, EPI_ISL_1677710, EPI_ISL_1677711, EPI_ISL_1677712, EPI_ISL_1677713, EPI_ISL_1677714, EPI_ISL_1677715, EPI_ISL_1677716, EPI_ISL_1677717, EPI_ISL_1677718, EPI_ISL_1677719, EPI_ISL_1677720, EPI_ISL_1677721, EPI_ISL_1677722, EPI_ISL_1677723, EPI_ISL_1677724, EPI_ISL_1677725, EPI_ISL_1677726, EPI_ISL_1677727, EPI_ISL_1677728, EPI_ISL_1677729, EPI_ISL_1677730, EPI_ISL_1677731, EPI_ISL_1677732, EPI_ISL_1677733, EPI_ISL_1677734, EPI_ISL_1677735, EPI_ISL_1677736, EPI_ISL_1677737, EPI_ISL_1677738			
see above	National Health Laboratory	Botswana Institute for Technology Research and Innovation	Dineo Emang Tshiamo. Tefelo Thela; Gape Nyepetsi; Kefentse Arnold Tumedi; Madisa Mine; Maitshwarelo Ignatius Matsheka; Malebogo Kebabonye; Thongbotho Mphoyakgosi
EPI_ISL_1366754, EPI_ISL_1366755, EPI_ISL_1366756, EPI_ISL_1366757, EPI_ISL_1366758, EPI_ISL_1366759, EPI_ISL_1366760, EPI_ISL_1366761, EPI_ISL_1366762, EPI_ISL_1366763, EPI_ISL_1366764, EPI_ISL_1366765, EPI_ISL_1366766, EPI_ISL_1366767, EPI_ISL_1366768, EPI_ISL_1366769, EPI_ISL_1366770, EPI_ISL_1366771, EPI_ISL_1366772, EPI_ISL_1366773, EPI_ISL_1366774, EPI_ISL_1366775, EPI_ISL_1366776, EPI_ISL_1366777, EPI_ISL_1366778, EPI_ISL_1366779, EPI_ISL_1366780, EPI_ISL_1366781, EPI_ISL_1366782, EPI_ISL_1366783, EPI_ISL_1366784, EPI_ISL_1366785, EPI_ISL_1366786, EPI_ISL_1366787, EPI_ISL_1366788, EPI_ISL_1366789, EPI_ISL_1366790, EPI_ISL_1366791, EPI_ISL_1366792, EPI_ISL_1366793, EPI_ISL_1366794, EPI_ISL_1366795, EPI_ISL_1366796, EPI_ISL_1366797, EPI_ISL_1366798, EPI_ISL_1366799, EPI_ISL_1366800, EPI_ISL_1366801, EPI_ISL_1366802, EPI_ISL_1366803, EPI_ISL_1366804, EPI_ISL_1366805, EPI_ISL_1366806, EPI_ISL_1366807, EPI_ISL_1366808, EPI_ISL_1366809, EPI_ISL_1366810, EPI_ISL_1366811, EPI_ISL_1366812, EPI_ISL_1366813, EPI_ISL_1366814, EPI_ISL_1366815, EPI_ISL_1366816, EPI_ISL_1366817, EPI_ISL_1366818, EPI_ISL_1366819, EPI_ISL_1366820, EPI_ISL_1366821, EPI_ISL_1366822, EPI_ISL_1366823, EPI_ISL_1366824, EPI_ISL_1366825, EPI_ISL_1366826, EPI_ISL_1366827, EPI_ISL_1366828, EPI_ISL_1366829, EPI_ISL_1366830, EPI_ISL_1366831, EPI_ISL_1366832, EPI_ISL_1366833, EPI_ISL_1366834, EPI_ISL_1366835, EPI_ISL_1366836, EPI_ISL_1366837, EPI_ISL_1366838, EPI_ISL_1366839, EPI_ISL_1366840, EPI_ISL_1366841, EPI_ISL_1366842, EPI_ISL_1366843, EPI_ISL_1366844, EPI_ISL_1366845, EPI_ISL_1366846, EPI_ISL_1366847, EPI_ISL_1366848, EPI_ISL_1366849, EPI_ISL_1366850, EPI_ISL_1366851, EPI_ISL_1366852, EPI_ISL_1366853, EPI_ISL_1366854, EPI_ISL_1366855, EPI_ISL_1366856, EPI_ISL_1366857, EPI_ISL_1366858, EPI_ISL_1366859, EPI_ISL_1366860, EPI_ISL_1366861, EPI_ISL_1366862, EPI_ISL_1366863, EPI_ISL_1366864, EPI_ISL_1366865, EPI_ISL_1366866, EPI_ISL_1366867, EPI_ISL_1366868, EPI_ISL_1366869, EPI_ISL_1366870, EPI_ISL_1366871, EPI_ISL_1366872, EPI_ISL_1366873, EPI_ISL_1366874, EPI_ISL_1366875, EPI_ISL_1366876, EPI_ISL_1366877, EPI_ISL_1366878, EPI_ISL_1366879, EPI_ISL_1366880, EPI_ISL_1366881, EPI_ISL_1366882, EPI_ISL_1366883, EPI_ISL_1366884, EPI_ISL_1366885, EPI_ISL_1366886, EPI_ISL_1366887, EPI_ISL_1366888, EPI_ISL_1550913, EPI_ISL_1550921, EPI_ISL_1550924, EPI_ISL_1550930, EPI_ISL_1550932, EPI_ISL_1550938, EPI_ISL_1550939, EPI_ISL_1550941, EPI_ISL_1550942, EPI_ISL_1817782, EPI_ISL_1817783, EPI_ISL_1817855, EPI_ISL_1817856, EPI_ISL_1817857, EPI_ISL_1817864, EPI_ISL_2397050, EPI_ISL_2397062, EPI_ISL_2397111, EPI_ISL_2397118, EPI_ISL_2397122, EPI_ISL_2397126, EPI_ISL_2397140, EPI_ISL_2397142, EPI_ISL_2397147, EPI_ISL_2397151, EPI_ISL_2494801, EPI_ISL_2494808, EPI_ISL_2494812, EPI_ISL_2494815, EPI_ISL_2494821, EPI_ISL_2494824, EPI_ISL_2494835, EPI_ISL_2494836, EPI_ISL_2494843, EPI_ISL_2494857, EPI_ISL_2494866, EPI_ISL_2494897			
see above	National Health Laboratory Service, South Africa	KRISP, KZN Research Innovation and Sequencing Platform	Emmanuel SJ; Giandhari J; Khan S; Laguda-Akingba O; Lessells R; Mdlalose K; Naidoo Y; Pillay S; Ramphal U; Tegally H; Wilkinson E; York D; de Oliveira T
EPI_ISL_1371906, EPI_ISL_1371907, EPI_ISL_1371908, EPI_ISL_1371909, EPI_ISL_1371910, EPI_ISL_1371911, EPI_ISL_1371912, EPI_ISL_1371913, EPI_ISL_1371914, EPI_ISL_1371915, EPI_ISL_1371916, EPI_ISL_1371917, EPI_ISL_1371918, EPI_ISL_1371919, EPI_ISL_1371920, EPI_ISL_1371921, EPI_ISL_1371922, EPI_ISL_1371923, EPI_ISL_1371924, EPI_ISL_1371925, EPI_ISL_1371926, EPI_ISL_1371927, EPI_ISL_1371928, EPI_ISL_1371929, EPI_ISL_1371930, EPI_ISL_1371931, EPI_ISL_1371932, EPI_ISL_1371933, EPI_ISL_1371934, EPI_ISL_1371935, EPI_ISL_1371936, EPI_ISL_1371937, EPI_ISL_1371938, EPI_ISL_1371939, EPI_ISL_1371940, EPI_ISL_1371941, EPI_ISL_1371942, EPI_ISL_1371943, EPI_ISL_1371944, EPI_ISL_1371945, EPI_ISL_1371946, EPI_ISL_1371947, EPI_ISL_1371948, EPI_ISL_1371949, EPI_ISL_1371950, EPI_ISL_1371951, EPI_ISL_1371952, EPI_ISL_1371953, EPI_ISL_1371954, EPI_ISL_1371955, EPI_ISL_1371956, EPI_ISL_1371957, EPI_ISL_1371958, EPI_ISL_1371959, EPI_ISL_1371960, EPI_ISL_1371961, EPI_ISL_1371962, EPI_ISL_1371963, EPI_ISL_1371964, EPI_ISL_1371965, EPI_ISL_1371966, EPI_ISL_1371967, EPI_ISL_1371968, EPI_ISL_1371969, EPI_ISL_1371970, EPI_ISL_1371971, EPI_ISL_1371972, EPI_ISL_1371973, EPI_ISL_1371974, EPI_ISL_1371975, EPI_ISL_1371976, EPI_ISL_1371977, EPI_ISL_1371978, EPI_ISL_1371979, EPI_ISL_1371980, EPI_ISL_1371981, EPI_ISL_1371982, EPI_ISL_1371983, EPI_ISL_1371984, EPI_ISL_1371985, EPI_ISL_1371986, EPI_ISL_1371987, EPI_ISL_1371988, EPI_ISL_1371989, EPI_ISL_1371990, EPI_ISL_1371991, EPI_ISL_1371992, EPI_ISL_1371993, EPI_ISL_1371994, EPI_ISL_1371995, EPI_ISL_1371996, EPI_ISL_1371997, EPI_ISL_1371998, EPI_ISL_1371999, EPI_ISL_1372000, EPI_ISL_1372001, EPI_ISL_1372002, EPI_ISL_1372003, EPI_ISL_1372004, EPI_ISL_1372005, EPI_ISL_1372006			
see above	National Health Laboratory Service, South Africa	KRISP, KZn Research Innovation and Sequencing Platform	Emmanuel SJ; Giandhari J; Khan S; Lessells R; Maslo C; Mdlalose K; Pillay S; Sitharam L; Tegally H; Wilkinson E; York D; de Oliveira T
EPI_ISL_1305818, EPI_ISL_1363124, EPI_ISL_1363125, EPI_ISL_1363126, EPI_ISL_1363127, EPI_ISL_1363128, EPI_ISL_1363129, EPI_ISL_1363130, EPI_ISL_1363131, EPI_ISL_1363132, EPI_ISL_1363133, EPI_ISL_1363134, EPI_ISL_1363135, EPI_ISL_1363136, EPI_ISL_1363137, EPI_ISL_1363138, EPI_ISL_1363139, EPI_ISL_1363140, EPI_ISL_1363141, EPI_ISL_1363142, EPI_ISL_1363143, EPI_ISL_1363144, EPI_ISL_1363145, EPI_ISL_1363146, EPI_ISL_1363147, EPI_ISL_1363148, EPI_ISL_1363149, EPI_ISL_1363150, EPI_ISL_1372562, EPI_ISL_1372563, EPI_ISL_1373589, EPI_ISL_1373590, EPI_ISL_1373591, EPI_ISL_1373592, EPI_ISL_1373593, EPI_ISL_1373594, EPI_ISL_1373595, EPI_ISL_1373596, EPI_ISL_1373597, EPI_ISL_1373598, EPI_ISL_1373599, EPI_ISL_1373600, EPI_ISL_1373601			
see above	National Health Laboratory Services	National Health Laboratory Services	Arshad Ismail; Florette Treurnicht; Kathleen Subramoney; Mushal Ali; Thabo Mohale
EPI_ISL_2535783, EPI_ISL_2535800, EPI_ISL_2535801, EPI_ISL_2535802, EPI_ISL_2535803, EPI_ISL_2535804, EPI_ISL_2535805, EPI_ISL_2535806, EPI_ISL_2535807, EPI_ISL_2535808, EPI_ISL_2535809, EPI_ISL_2535810, EPI_ISL_2535811, EPI_ISL_2535812, EPI_ISL_2535813, EPI_ISL_2535814, EPI_ISL_2535815, EPI_ISL_2535816, EPI_ISL_2535817, EPI_ISL_2535818, EPI_ISL_2535819, EPI_ISL_2535820, EPI_ISL_2535821, EPI_ISL_2535822, EPI_ISL_2535823, EPI_ISL_2535824, EPI_ISL_2535825, EPI_ISL_2535826, EPI_ISL_2535827, EPI_ISL_2535828, EPI_ISL_2535829, EPI_ISL_2535830, EPI_ISL_2535831, EPI_ISL_2535832, EPI_ISL_2535833, EPI_ISL_2535834, EPI_ISL_2535835, EPI_ISL_2535836, EPI_ISL_2535837			
see above	National Influenza Centre	National Influenza Centre	; Benjamiin B. Lindsey; Benjamin H. Foulkes; Dennis Laryea; Ernest Asiedu; Franklin Asiedu-Bekoe; Gordon Awandare; Ivy A. Asante; Joseph Oliver-Commey; Joyce Ngoi; Linda Boatemaa; Lorreta Kwasah; Mathew D. Parker; Michael Marks; Mildred Adusei-Poku; Sharon Hsu; Thushan I de Silva; William K. Ampofo
EPI_ISL_2433725, EPI_ISL_2433726, EPI_ISL_2433727, EPI_ISL_2433728, EPI_ISL_2433729, EPI_ISL_2433730, EPI_ISL_2433731, EPI_ISL_2433732, EPI_ISL_2433733, EPI_ISL_2433734, EPI_ISL_2433735, EPI_ISL_2433736, EPI_ISL_2433737, EPI_ISL_2433738, EPI_ISL_2433739, EPI_ISL_2433740, EPI_ISL_2433741, EPI_ISL_2433742, EPI_ISL_2433743, EPI_ISL_2433744, EPI_ISL_2433745, EPI_ISL_2433746, EPI_ISL_2433747, EPI_ISL_2433748, EPI_ISL_2433749, EPI_ISL_2433750, EPI_ISL_2433751, EPI_ISL_2433752, EPI_ISL_2433753, EPI_ISL_2433754, EPI_ISL_2433755, EPI_ISL_2433756, EPI_ISL_2433757, EPI_ISL_2433758, EPI_ISL_2433759, EPI_ISL_2433760, EPI_ISL_2433761, EPI_ISL_2433762, EPI_ISL_2433763, EPI_ISL_2433764, EPI_ISL_2433765, EPI_ISL_2433766, EPI_ISL_2433767, EPI_ISL_2433768, EPI_ISL_2433769, EPI_ISL_2433770, EPI_ISL_2433771, EPI_ISL_2433772, EPI_ISL_2433773, EPI_ISL_2433774, EPI_ISL_2433775, EPI_ISL_2433776, EPI_ISL_2433796, EPI_ISL_2433797, EPI_ISL_2433798, EPI_ISL_2433799, EPI_ISL_2433800, EPI_ISL_2433801, EPI_ISL_2433802, EPI_ISL_2433803, EPI_ISL_2433804, EPI_ISL_2433805, EPI_ISL_2433806, EPI_ISL_2433807, EPI_ISL_2433808, EPI_ISL_2433809, EPI_ISL_2433810, EPI_ISL_2433811, EPI_ISL_2433812, EPI_ISL_2433813, EPI_ISL_2433814, EPI_ISL_2433815, EPI_ISL_2433816, EPI_ISL_2433817, EPI_ISL_2433818, EPI_ISL_2433819, EPI_ISL_2433820, EPI_ISL_2433821, EPI_ISL_2433822, EPI_ISL_2433823, EPI_ISL_2433824, EPI_ISL_2433825, EPI_ISL_2433826, EPI_ISL_2433827, EPI_ISL_2433828, EPI_ISL_2433829, EPI_ISL_2433830, EPI_ISL_2433831, EPI_ISL_2433832, EPI_ISL_2433833, EPI_ISL_2433834, EPI_ISL_2433835, EPI_ISL_2433836, EPI_ISL_2433837, EPI_ISL_2433838, EPI_ISL_2433839, EPI_ISL_2433840, EPI_ISL_2433841, EPI_ISL_2433842, EPI_ISL_2433843, EPI_ISL_2433844, EPI_ISL_2433845, EPI_ISL_2433846, EPI_ISL_2433847, EPI_ISL_2433848, EPI_ISL_2433849, EPI_ISL_2443997, EPI_ISL_2443998, EPI_ISL_2443999, EPI_ISL_2444000, EPI_ISL_2444001, EPI_ISL_2444002, EPI_ISL_2444003, EPI_ISL_2444004, EPI_ISL_2444005, EPI_ISL_2444006, EPI_ISL_2444007, EPI_ISL_2444008, EPI_ISL_2444009, EPI_ISL_2444010, EPI_ISL_2444011, EPI_ISL_2444012, EPI_ISL_2444013, EPI_ISL_2444014, EPI_ISL_2444015, EPI_ISL_2444016, EPI_ISL_2444017, EPI_ISL_2444018, EPI_ISL_2444019, EPI_ISL_2444020, EPI_ISL_2444021, EPI_ISL_2444022, EPI_ISL_2444023, EPI_ISL_2444024, EPI_ISL_2444026, EPI_ISL_2444027, EPI_ISL_2444028, EPI_ISL_2444029, EPI_ISL_2444030, EPI_ISL_2444031, EPI_ISL_2444032, EPI_ISL_2444033, EPI_ISL_2444034, EPI_ISL_2444035, EPI_ISL_2444036, EPI_ISL_2444037, EPI_ISL_2444038, EPI_ISL_2444039, EPI_ISL_2444040, EPI_ISL_2444041, EPI_ISL_2444042, EPI_ISL_2444043, EPI_ISL_2444044, EPI_ISL_2444045, EPI_ISL_2444046, EPI_ISL_2444047, EPI_ISL_2444048, EPI_ISL_2444049, EPI_ISL_2444050, EPI_ISL_2444051, EPI_ISL_2444052, EPI_ISL_2444053, EPI_ISL_2444054, EPI_ISL_2444055, EPI_ISL_2444056, EPI_ISL_2444057, EPI_ISL_2444058, EPI_ISL_2444059, EPI_ISL_2444060, EPI_ISL_2444061, EPI_ISL_2444062, EPI_ISL_2444063, EPI_ISL_2444064, EPI_ISL_2444065, EPI_ISL_2444066, EPI_ISL_2444067, EPI_ISL_2444068, EPI_ISL_2444069, EPI_ISL_2444070, EPI_ISL_2444071, EPI_ISL_2444072, EPI_ISL_2444073, EPI_ISL_2444074, EPI_ISL_2444075, EPI_ISL_2444076, EPI_ISL_2444077, EPI_ISL_2444078, EPI_ISL_2444079, EPI_ISL_2444080, EPI_ISL_2444081, EPI_ISL_2444082, EPI_ISL_2444083, EPI_ISL_2444084, EPI_ISL_2444085, EPI_ISL_2444086, EPI_ISL_2444087, EPI_ISL_2444088, EPI_ISL_2444089, EPI_ISL_2444090, EPI_ISL_2444091, EPI_ISL_2444092, EPI_ISL_2444093, EPI_ISL_2447827, EPI_ISL_2447828, EPI_ISL_2447829, EPI_ISL_2447830, EPI_ISL_2447875, EPI_ISL_2447876, EPI_ISL_2447877, EPI_ISL_2447879, EPI_ISL_2447880, EPI_ISL_2447883, EPI_ISL_2447884, EPI_ISL_2447886, EPI_ISL_2447887, EPI_ISL_2447888, EPI_ISL_2447889, EPI_ISL_2447891, EPI_ISL_2447892, EPI_ISL_2447895			
see above	National Institute for Communicable Diseases of the National Health Laboratory Service	National Institute for Communicable Diseases of the National Health Laboratory Service	Amoako DG; Bhiman JN; Ismail A; Mahlangu B; Mohale T; Ntuli N; Scheepers C
EPI_ISL_1827555, EPI_ISL_1827556, EPI_ISL_1827557, EPI_ISL_1827558, EPI_ISL_1827559, EPI_ISL_1827560, EPI_ISL_1827561, EPI_ISL_1827562, EPI_ISL_1827563, EPI_ISL_1827564, EPI_ISL_1827565, EPI_ISL_1827566, EPI_ISL_1827567, EPI_ISL_1827568, EPI_ISL_1827569, EPI_ISL_1827570, EPI_ISL_1827571, EPI_ISL_1827572, EPI_ISL_1827573, EPI_ISL_1827574, EPI_ISL_1827575, EPI_ISL_1827576, EPI_ISL_1827577, EPI_ISL_1827578, EPI_ISL_1827579, EPI_ISL_1827580, EPI_ISL_1827581, EPI_ISL_1827582, EPI_ISL_1827583, EPI_ISL_1827584, EPI_ISL_1827585, EPI_ISL_1827586, EPI_ISL_1827587, EPI_ISL_1827588, EPI_ISL_1827589, EPI_ISL_1827590, EPI_ISL_1827591, EPI_ISL_1827592, EPI_ISL_1827593, EPI_ISL_1827594, EPI_ISL_1827595, EPI_ISL_1827596, EPI_ISL_1827597, EPI_ISL_1827598, EPI_ISL_1827599, EPI_ISL_1827600, EPI_ISL_1827601, EPI_ISL_1827602, EPI_ISL_1827603, EPI_ISL_1827604, EPI_ISL_1827605, EPI_ISL_1827606, EPI_ISL_1827607, EPI_ISL_1827608, EPI_ISL_1827609, EPI_ISL_1827610, EPI_ISL_1827611, EPI_ISL_1827612, EPI_ISL_1827613, EPI_ISL_1827614, EPI_ISL_1827615, EPI_ISL_1827616, EPI_ISL_1827618, EPI_ISL_1827620, EPI_ISL_1827621, EPI_ISL_1827622, EPI_ISL_1827623, EPI_ISL_1827624, EPI_ISL_1827625, EPI_ISL_1827626, EPI_ISL_1827627, EPI_ISL_1827628, EPI_ISL_1827629, EPI_ISL_1827630, EPI_ISL_1827631, EPI_ISL_1827632, EPI_ISL_1827633, EPI_ISL_1914786, EPI_ISL_1914787, EPI_ISL_1914788, EPI_ISL_1914789, EPI_ISL_1914790, EPI_ISL_1914791, EPI_ISL_1914792, EPI_ISL_1914793, EPI_ISL_1914794, EPI_ISL_1914795, EPI_ISL_1914796, EPI_ISL_1914797, EPI_ISL_1914798, EPI_ISL_1914799, EPI_ISL_1914800, EPI_ISL_1914801, EPI_ISL_1914802, EPI_ISL_1914803, EPI_ISL_1914804, EPI_ISL_1914805, EPI_ISL_1914806, EPI_ISL_1914807, EPI_ISL_1914808, EPI_ISL_1914809, EPI_ISL_1914810, EPI_ISL_1914811, EPI_ISL_1914812, EPI_ISL_1914813, EPI_ISL_1914814, EPI_ISL_1914815, EPI_ISL_1914816, EPI_ISL_1914817, EPI_ISL_1914818, EPI_ISL_1914819, EPI_ISL_1914820, EPI_ISL_1914821, EPI_ISL_1914822, EPI_ISL_1914823, EPI_ISL_1914824, EPI_ISL_1914825, EPI_ISL_1914826, EPI_ISL_1914827, EPI_ISL_1914828, EPI_ISL_1914829, EPI_ISL_1914830, EPI_ISL_1914831, EPI_ISL_1914832, EPI_ISL_1914833, EPI_ISL_1914834, EPI_ISL_1914835, EPI_ISL_1914836, EPI_ISL_1914837, EPI_ISL_1914838, EPI_ISL_1914839, EPI_ISL_1914840, EPI_ISL_1914841, EPI_ISL_1914842, EPI_ISL_1914843, EPI_ISL_1914845, EPI_ISL_1914848, EPI_ISL_1914849, EPI_ISL_1914850, EPI_ISL_1914851, EPI_ISL_1914852, EPI_ISL_1914853, EPI_ISL_1914854, EPI_ISL_1914855, EPI_ISL_1914856, EPI_ISL_1914857, EPI_ISL_1914858, EPI_ISL_1914859, EPI_ISL_1914860, EPI_ISL_1914861, EPI_ISL_1914862, EPI_ISL_1914863, EPI_ISL_1914864, EPI_ISL_1914865, EPI_ISL_1914866, EPI_ISL_1914867, EPI_ISL_1914868, EPI_ISL_1914869, EPI_ISL_1914870, EPI_ISL_1914871, EPI_ISL_1914872, EPI_ISL_1914873, EPI_ISL_1914874, EPI_ISL_1914875, EPI_ISL_1914876, EPI_ISL_1914877, EPI_ISL_1914878, EPI_ISL_1914879, EPI_ISL_1914880, EPI_ISL_1914881, EPI_ISL_1914882, EPI_ISL_1914883, EPI_ISL_1914884, EPI_ISL_1914885, EPI_ISL_1914886, EPI_ISL_1914887, EPI_ISL_1914888, EPI_ISL_1914889, EPI_ISL_1914890, EPI_ISL_1914891, EPI_ISL_1914892, EPI_ISL_1914893, EPI_ISL_1914894, EPI_ISL_1914895, EPI_ISL_1914896, EPI_ISL_1914897, EPI_ISL_1914898, EPI_ISL_1914899, EPI_ISL_1914900, EPI_ISL_1914901, EPI_ISL_1914902, EPI_ISL_1914903, EPI_ISL_1914904, EPI_ISL_1914905, EPI_ISL_1914906, EPI_ISL_1914907, EPI_ISL_1914908, EPI_ISL_1914909, EPI_ISL_1914910, EPI_ISL_1914911, EPI_ISL_1914912, EPI_ISL_1914913, EPI_ISL_1914914, EPI_ISL_1914915, EPI_ISL_1914916, EPI_ISL_1914917, EPI_ISL_1914918, EPI_ISL_1914919, EPI_ISL_1914920, EPI_ISL_1914921, EPI_ISL_1914922, EPI_ISL_1914923, EPI_ISL_1914924, EPI_ISL_1914925, EPI_ISL_1914926, EPI_ISL_1914927			
see above	National Institute for Communicable Diseases,National Health Laboratory Services, Gauteng, South Africa	National Institute for Communicable Diseases of the National Health Laboratory Service	Amoako DG; Bhiman JN; Ismail A; Mahlangu B; Mohale T; Ntuli N; Scheepers C
EPI_ISL_2107093, EPI_ISL_2107094, EPI_ISL_2107095, EPI_ISL_2107096, EPI_ISL_2107097, EPI_ISL_2107098, EPI_ISL_2107099, EPI_ISL_2107100, EPI_ISL_2107101, EPI_ISL_2107102, EPI_ISL_2107103, EPI_ISL_2107104, EPI_ISL_2107105, EPI_ISL_2107106, EPI_ISL_2107107, EPI_ISL_2308252, EPI_ISL_2308255, EPI_ISL_2308256, EPI_ISL_2308257, EPI_ISL_2308258, EPI_ISL_2308259, EPI_ISL_2308260, EPI_ISL_2308261, EPI_ISL_2308262			
see above	National Institute of Health (NIH) - Federal Government of Somalia	African Centre of Excellence for Genomics of Infectious Diseases (ACEGID), Redeemer's University	A.T.; Abechi; Ajogbasile; Akano; C.A.; C.T.; Eromon; F.V.; Folarin, O.; Happi; I.B.; J.N.; J.U.; K.O.; Kayode; Nosamiefan, I.; Oguzie; Olawoye; Olumade; Oluniyi; P.E.; P.S.; T.J.; Ugwu; Uwanibe
EPI_ISL_2492547, EPI_ISL_2492548, EPI_ISL_2492561, EPI_ISL_2492562, EPI_ISL_2492564, EPI_ISL_2492565, EPI_ISL_2492569, EPI_ISL_2492577, EPI_ISL_2492578, EPI_ISL_2492579, EPI_ISL_2492581, EPI_ISL_2492582, EPI_ISL_2492586, EPI_ISL_2492589, EPI_ISL_2492590, EPI_ISL_2492593, EPI_ISL_2492595, EPI_ISL_2492596, EPI_ISL_2492600, EPI_ISL_2492601, EPI_ISL_2492602, EPI_ISL_2492605, EPI_ISL_2492606, EPI_ISL_2492608, EPI_ISL_2492610, EPI_ISL_2492613, EPI_ISL_2492614, EPI_ISL_2492615, EPI_ISL_2492616, EPI_ISL_2492617, EPI_ISL_2492618, EPI_ISL_2492619, EPI_ISL_2492625, EPI_ISL_2492628, EPI_ISL_2492629, EPI_ISL_2492634, EPI_ISL_2492636, EPI_ISL_2492639, EPI_ISL_2492640, EPI_ISL_2492647, EPI_ISL_2492650, EPI_ISL_2492651, EPI_ISL_2492652, EPI_ISL_2492654, EPI_ISL_2492655, EPI_ISL_2492657, EPI_ISL_2492658, EPI_ISL_2492659, EPI_ISL_2492660, EPI_ISL_2492661, EPI_ISL_2492662, EPI_ISL_2492665, EPI_ISL_2492666, EPI_ISL_2492667, EPI_ISL_2492669, EPI_ISL_2492670, EPI_ISL_2492671, EPI_ISL_2492672, EPI_ISL_2492673, EPI_ISL_2492675, EPI_ISL_2492677, EPI_ISL_2492679, EPI_ISL_2492681, EPI_ISL_2492683, EPI_ISL_2492685, EPI_ISL_2492691, EPI_ISL_2492693, EPI_ISL_2492694, EPI_ISL_2492698, EPI_ISL_2492701, EPI_ISL_2492703, EPI_ISL_2492704, EPI_ISL_2492705, EPI_ISL_2492707, EPI_ISL_2492708, EPI_ISL_2492709, EPI_ISL_2492712, EPI_ISL_2492715, EPI_ISL_2492717, EPI_ISL_2492718, EPI_ISL_2492719, EPI_ISL_2492720, EPI_ISL_2492721, EPI_ISL_2492722, EPI_ISL_2492724, EPI_ISL_2492725, EPI_ISL_2492726, EPI_ISL_2492730, EPI_ISL_2492732, EPI_ISL_2492733, EPI_ISL_2492735, EPI_ISL_2492741, EPI_ISL_2492745, EPI_ISL_2492746, EPI_ISL_2492748, EPI_ISL_2492749, EPI_ISL_2492760, EPI_ISL_2492765, EPI_ISL_2492768, EPI_ISL_2492772, EPI_ISL_2492775, EPI_ISL_2492783, EPI_ISL_2492793, EPI_ISL_2492795, EPI_ISL_2492796, EPI_ISL_2492802, EPI_ISL_2492823, EPI_ISL_2492836, EPI_ISL_2492856, EPI_ISL_2492860, EPI_ISL_2492866, EPI_ISL_2492873, EPI_ISL_2492875, EPI_ISL_2492876, EPI_ISL_2492880, EPI_ISL_2492883, EPI_ISL_2492886, EPI_ISL_2492891, EPI_ISL_2492896, EPI_ISL_2492906, EPI_ISL_2492908, EPI_ISL_2492913, EPI_ISL_2492916, EPI_ISL_2492917, EPI_ISL_2492918, EPI_ISL_2492919, EPI_ISL_2492920, EPI_ISL_2492930, EPI_ISL_2492933, EPI_ISL_2492959, EPI_ISL_2492979, EPI_ISL_2492986, EPI_ISL_2492989, EPI_ISL_2492995, EPI_ISL_2493004, EPI_ISL_2493006, EPI_ISL_2493019, EPI_ISL_2493021, EPI_ISL_2493038, EPI_ISL_2493042, EPI_ISL_2493043, EPI_ISL_2493044, EPI_ISL_2493045, EPI_ISL_2493046, EPI_ISL_2493047, EPI_ISL_2493048, EPI_ISL_2493058, EPI_ISL_2493059, EPI_ISL_2493060, EPI_ISL_2493061, EPI_ISL_2493062, EPI_ISL_2493063, EPI_ISL_2493064, EPI_ISL_2493065, EPI_ISL_2493066, EPI_ISL_2493088, EPI_ISL_2493089, EPI_ISL_2493090, EPI_ISL_2493096, EPI_ISL_2493097			
see above	National Microbiology Reference Laboratory, Ministry of Health, Harare, Zimbabwe	CERI, Centre for Epidemic Response and Innvoation, Stellenbosch University and KRISP, KZN Research Innovation and Sequencing Platform, UKZN.	Agnes Juru; Air Comodor Dr J. Chimedza; Charles Nyagupe; Dr Raiva Simbi; Emmanuel SJ; Giandhari J; Hlanai Gumbo; Kenneth Maeka; Naidoo Y; Pillay S; Tapfumanei Mashe; Tatenda Takawira; Tegally H; Wilkinson E; de Oliveira T
EPI_ISL_1715191, EPI_ISL_1715192, EPI_ISL_1715193, EPI_ISL_1715195, EPI_ISL_1715196, EPI_ISL_1715197, EPI_ISL_1715198, EPI_ISL_1715199, EPI_ISL_1715200			
see above	National Public Health Laboratory, Cameroon	African Centre of Excellence for Genomics of Infectious Diseases, Redeemer's University	I.B. et al; Olawoye
EPI_ISL_2450770, EPI_ISL_2450771, EPI_ISL_2450772, EPI_ISL_2450773, EPI_ISL_2450774, EPI_ISL_2450775, EPI_ISL_2450776, EPI_ISL_2450777, EPI_ISL_2450778, EPI_ISL_2450779, EPI_ISL_2450780, EPI_ISL_2450781, EPI_ISL_2450782, EPI_ISL_2450783, EPI_ISL_2450784, EPI_ISL_2450785, EPI_ISL_2450799, EPI_ISL_2450803, EPI_ISL_2450804, EPI_ISL_2450805			
see above	National Public Health Laboratory, Ministry of Health, Ministry of Health, Republic of South Sudan	South Sudan Ministry of Health, WHO South Sudan, MRC/UVRI & LSHTM Uganda Research Unit	Abe G. Abias; Dan Lule Bugembe; Dennis Kenyi Lodiongo; James Ayei; John Rumunu; Joseph Francis Wamala; Juma John HM; Lul Lojok Deng; Matthew Cotten; My V.T. Phan; Pontiano Kaleebu; Richard Lino Loro Lako; Sudhir Bunga
EPI_ISL_1498123, EPI_ISL_1498124, EPI_ISL_1498125	National Reference Laboratory	National Reference Laboratory	Enatha Mukantwari; Jeanne d'Arc Umuringa; Umuringa Jeanne d'Arc; Yvan Butera
EPI_ISL_1273391, EPI_ISL_1273392, EPI_ISL_1273393, EPI_ISL_1273394, EPI_ISL_1273395, EPI_ISL_1273396, EPI_ISL_1273397, EPI_ISL_1273398, EPI_ISL_1273399, EPI_ISL_1273400, EPI_ISL_1273401, EPI_ISL_1273402, EPI_ISL_1273403, EPI_ISL_1273404, EPI_ISL_1273405, EPI_ISL_1273406, EPI_ISL_1273407, EPI_ISL_1273408			
see above	National Reference Laboratory - Ministry of Health Maseru Lesotho	National Institute for Communicable Diseases of the National Health Laboratory Service	Amoako DG; Banda R; Bhiman JN; Gorova V; Ismail A; Mahlangu B; Mathabo M; Mohale T; Mooko M; Ntuli N; Scheepers C
EPI_ISL_1629712, EPI_ISL_1629713, EPI_ISL_1629714	National Reference Laboratory, NCDC, Gaduwa, Abuja, Nigeria	National Reference Laboratory, NCDC, Gaduwa, Abuja, Nigeria	Anthony Ahumibe; Chimaobi Chukwu; Dr Chikwe Ihekweazu; Dr Grace Esebanmen; Dr Ndodo Nnaemeka; Dr Omoare Adesuyi; Nwando Mba; Olusola Anuoluwapo Akanbi
EPI_ISL_2693103, EPI_ISL_2693104, EPI_ISL_2693105, EPI_ISL_2821075, EPI_ISL_2821081, EPI_ISL_2821257, EPI_ISL_2821326, EPI_ISL_2821509, EPI_ISL_2821510, EPI_ISL_2821669, EPI_ISL_2824987, EPI_ISL_2825066, EPI_ISL_2825070			
see above	National Reference Laboratory, Nigeria Centre for Disease Control	National Reference Laboratory, Nigeria Centre for Disease Control	Anthony Ahumibe; Catherine Okoi; Chimaobi Chukwu; Dr Chikwe Ihekweazu; Dr Ndodo Nnaemeka; Dr Omoare Adesuyi; Grace Esebanmen; Nwando Mba; Olusola Anuoluwapo Akanbi
EPI_ISL_2821076	National Reference Laboratory, Nigeria Centre for Disease Control	National Reference Laboratory, Nigeria Centre for Disease Control Durumi III, Gaduwa, Abuja, Nigeria	Anthony Ahumibe; Catherine Okoi; Chimaobi Chukwu; Dr Chikwe Ihekweazu; Dr Ndodo Nnaemeka; Dr Omoare Adesuyi; Grace Esebanmen; Nwando Mba; Olusola Anuoluwapo Akanbi
EPI_ISL_1576832	National reference Laboratory, NCDC, Gaduwa, Abuja	National reference Laboratory, NCDC, Gaduwa, Abuja	Anthony Ahumibe; Chimaobi Chukwu; Dr Chikwe Ihekweazu; Dr Ndodo Nnaemeka; Dr Omoare Adesuyi; Nwando Mba; Olusola Anuoluwapo Akanbi
EPI_ISL_1627088, EPI_ISL_1627093, EPI_ISL_1627094, EPI_ISL_1627095, EPI_ISL_1627096	National reference Laboratory, NCDC, Gaduwa, Abuja, Nigeria	National reference Laboratory, NCDC, Gaduwa, Abuja, Nigeria	Adama Ahmad; Akinpelu Afolabi; Anthony Ahumibe; Badaru Sikiru; Catherine Okoi; Celestina Obiekea; Chimaobi Chukwu; Dr Chikwe Ihekweazu; Dr Grace Esebanmen; Dr Ndodo Nnaemeka; Dr Omoare Adesuyi; Kingsley Njoku; Nwando Mba; Olusola Anuoluwapo Akanbi
EPI_ISL_2533830, EPI_ISL_2533831, EPI_ISL_2533832, EPI_ISL_2533834, EPI_ISL_2533835, EPI_ISL_2533836, EPI_ISL_2533850, EPI_ISL_2533851, EPI_ISL_2533883, EPI_ISL_2858618, EPI_ISL_2858623, EPI_ISL_2858624, EPI_ISL_2858625, EPI_ISL_2858626, EPI_ISL_2858627, EPI_ISL_2858628, EPI_ISL_2858629, EPI_ISL_2858630, EPI_ISL_2858631, EPI_ISL_2858632, EPI_ISL_2858633, EPI_ISL_2858634, EPI_ISL_2858635, EPI_ISL_2858636, EPI_ISL_2858637, EPI_ISL_2858638, EPI_ISL_2858640, EPI_ISL_2858641, EPI_ISL_2858642, EPI_ISL_2858643, EPI_ISL_2858644, EPI_ISL_2858645, EPI_ISL_2858646, EPI_ISL_2858647, EPI_ISL_2858648, EPI_ISL_2858669, EPI_ISL_2858670, EPI_ISL_2858671, EPI_ISL_2858674, EPI_ISL_2858675, EPI_ISL_2858677, EPI_ISL_2858678, EPI_ISL_2858679, EPI_ISL_2858680, EPI_ISL_2858681, EPI_ISL_2858682, EPI_ISL_2858683, EPI_ISL_2858687, EPI_ISL_2860827, EPI_ISL_2860828, EPI_ISL_2860829, EPI_ISL_2860830, EPI_ISL_2860831, EPI_ISL_2860832, EPI_ISL_2860833, EPI_ISL_2860834, EPI_ISL_2860889			

see above	Naval Medical Research Unit No. 3	Naval Medical Research Center Biological Defense Research Directorate	Andrea E. Luquette; Andrew J. Bennett; Bishwo N. Adhikari; Catherine E. Arnold; Chaselynn M. Watters; Emily K. Stefanov; Francisco Malagon; Kyle A. Long; Logan J. Voegtly; Luis A. Estrella; Michael V. Deschenes; Regina Z. Cer; Stephen M. Eggan; and Kimberly A. Bishop-Lilly
EPI_ISL_1534438, EPI_ISL_2140669, EPI_ISL_2375950	New Horizon Clinic wc NZC	NHLS/UCT	Arash Iranzadeh; Bruna Galvao; Carolyn Williamson; Deelan Doolabh; Diana Hardie; Emmanuel SJ; Innocent Mudau; Kruger Marais; Lynn Tyers; Marvin Hsiao; Stephen Korsman; Tegally H; de Oliveira T
EPI_ISL_1381741, EPI_ISL_1381742, EPI_ISL_1381743, EPI_ISL_1381744, EPI_ISL_1381745, EPI_ISL_1381746, EPI_ISL_1381747, EPI_ISL_1381748, EPI_ISL_1381813, EPI_ISL_1381814, EPI_ISL_1381815, EPI_ISL_1381832			
see above	Nigeria Centre For Disease Control	National reference Laboratory, NCDC, Gaduwa, Abuja	Anthony Ahumibe; Catherine Okoi; Chimaobi Chukwu; Dr Chikwe Ihekweazu; Dr Ndodo Nnaemeka; Dr Omoare Adesuyi; Naidoo Dhamari; Nwando Mba; Olusola Anuoluwapo Akanbi
EPI_ISL_2565886, EPI_ISL_2565887, EPI_ISL_2565888, EPI_ISL_2565889, EPI_ISL_2565890, EPI_ISL_2565891, EPI_ISL_2565892, EPI_ISL_2565893			
see above	Nigeria Centre for Disease Control (NCDC)	Africa Centre for Excellence for Genomics of Infectious Diseases (ACEGID), Redeemer's University	A.T.; Abechi; Ajogbasile; Akano; C.A.; C.T.; Eromon; F.V.; Folarin, O.; Happi; I.B.; J.N.; J.U.; K.O.; Kayode; Nosamiefan, I.; Oguzie; Olawoye; Olumade; Oluniyi; P.E.; P.S.; T.J.; Ugwu; Uwanibe
EPI_ISL_1970547, EPI_ISL_1970549, EPI_ISL_1970550, EPI_ISL_1970551, EPI_ISL_1970552, EPI_ISL_1970553, EPI_ISL_1970554, EPI_ISL_1970555, EPI_ISL_1970556, EPI_ISL_1970558, EPI_ISL_1970559, EPI_ISL_1970560, EPI_ISL_1970561, EPI_ISL_1970562, EPI_ISL_1970563			
see above	Nigeria Centre for Disease Control (NCDC)	African Centre for Excellence for Genomics of Infectious Diseases (ACEGID), Redeemer's University	A.T.; Abechi; Ajogbasile; Akano; C.A.; C.T.; Eromon; F.V.; Folarin, O.; Happi; I.B.; J.N.; J.U.; K.O.; Kayode; Oguzie; Olawoye; Olumade; Oluniyi; P.E.; P.S.; T.J.; Ugwu; Uwanibe
EPI_ISL_2240750, EPI_ISL_2240751, EPI_ISL_2240752, EPI_ISL_2240760, EPI_ISL_2240761, EPI_ISL_2240762, EPI_ISL_2240763, EPI_ISL_2240764, EPI_ISL_2240765, EPI_ISL_2240766, EPI_ISL_2240767, EPI_ISL_2240768, EPI_ISL_2240769, EPI_ISL_2240770, EPI_ISL_2240771, EPI_ISL_2240772, EPI_ISL_2240773, EPI_ISL_2240774, EPI_ISL_2240775, EPI_ISL_2240776, EPI_ISL_2240777, EPI_ISL_2240778, EPI_ISL_2240779, EPI_ISL_2240780, EPI_ISL_2285314, EPI_ISL_2285315, EPI_ISL_2285318, EPI_ISL_2285320, EPI_ISL_2285322, EPI_ISL_2285323, EPI_ISL_2285324, EPI_ISL_2285325, EPI_ISL_2285326, EPI_ISL_2308268, EPI_ISL_2308269			
see above	Nigeria Centre for Disease Control (NCDC)	African Centre of Excellence for Genomics of Infectious Diseases (ACEGID), Redeemer's University	A.T.; Abechi; Ajogbasile; Akano; C.A.; C.T.; Eromon; F.V.; Folarin, O.; Happi; I.B.; J.N.; J.U.; K.O.; Kayode; Nosamiefan, I.; Oguzie; Olawoye; Olumade; Oluniyi; P.E.; P.S.; T.J.; Ugwu; Uwanibe
EPI_ISL_1715364, EPI_ISL_1715365, EPI_ISL_1715366, EPI_ISL_1715367, EPI_ISL_1715368, EPI_ISL_1715369, EPI_ISL_1715370, EPI_ISL_1715371, EPI_ISL_1715372, EPI_ISL_1715373, EPI_ISL_1715374, EPI_ISL_1715375, EPI_ISL_1715376, EPI_ISL_1715377, EPI_ISL_1715378, EPI_ISL_1715379, EPI_ISL_1715380, EPI_ISL_1715381, EPI_ISL_1715382, EPI_ISL_1715383, EPI_ISL_1715384, EPI_ISL_1715385, EPI_ISL_1715386, EPI_ISL_1715387, EPI_ISL_1715388, EPI_ISL_1715389, EPI_ISL_1715390, EPI_ISL_1715391, EPI_ISL_1715392, EPI_ISL_1715393, EPI_ISL_1715394, EPI_ISL_1715395, EPI_ISL_1715396, EPI_ISL_1715397, EPI_ISL_1715398, EPI_ISL_1715399, EPI_ISL_1715400, EPI_ISL_1715401, EPI_ISL_1715402, EPI_ISL_1715403, EPI_ISL_1715404, EPI_ISL_1715405, EPI_ISL_1715406, EPI_ISL_1715407, EPI_ISL_1715408, EPI_ISL_1715409, EPI_ISL_1715410, EPI_ISL_1715411, EPI_ISL_1715412, EPI_ISL_1715413, EPI_ISL_1715414, EPI_ISL_1715415, EPI_ISL_1715416, EPI_ISL_1715417			
see above	Nigerian Centre for Disease Control (NCDC)	African Centre of Excellence for Genomics of Infectious Diseases, Redeemer's University	I.B. et al; Olawoye
EPI_ISL_2361908, EPI_ISL_2361909, EPI_ISL_2361910, EPI_ISL_2361911, EPI_ISL_2361912, EPI_ISL_2361913, EPI_ISL_2361914, EPI_ISL_2361915, EPI_ISL_2376383, EPI_ISL_2376384, EPI_ISL_2376385, EPI_ISL_2376386			
see above	Noguchi Memorial Institute for Medical Research, University of Ghana, Legon, Ghana	Institute of Tropical Medicine, Universitätsklinikum Tübingen, Germany	Abraham Kwabena Anang; Bright Adu; Dorothy Yeboah-Manu; Hilda Opoku Frempong; John Kofi Odoom; Joseph Humphrey Kofi Bonney; Joyce Appiah-Kubi; Keren Okyerebea Attiku; Le Thi Kieu Linh; Quaneeta Mohktar; Sivaramakrishna Rachakonda; Srinivas-reddy Pallerla; Thirumalaisamy P Velavan
EPI_ISL_1300662, EPI_ISL_1300663, EPI_ISL_1301734, EPI_ISL_1301735, EPI_ISL_1301736, EPI_ISL_1301737, EPI_ISL_1301738, EPI_ISL_1301739, EPI_ISL_1301740, EPI_ISL_1301741, EPI_ISL_1301742, EPI_ISL_1301743, EPI_ISL_1301744, EPI_ISL_1301745, EPI_ISL_1301746, EPI_ISL_1301747, EPI_ISL_1301748, EPI_ISL_1301749, EPI_ISL_1301750, EPI_ISL_1301751, EPI_ISL_1301752, EPI_ISL_1301753, EPI_ISL_1301754, EPI_ISL_1301755, EPI_ISL_1301756, EPI_ISL_1301757, EPI_ISL_1301758, EPI_ISL_1301759, EPI_ISL_1301760, EPI_ISL_1301761, EPI_ISL_1301762, EPI_ISL_1301763, EPI_ISL_1301764, EPI_ISL_1301765, EPI_ISL_1301766, EPI_ISL_1301767, EPI_ISL_1301768, EPI_ISL_1301769, EPI_ISL_1301770, EPI_ISL_1301771, EPI_ISL_1301772, EPI_ISL_1301773, EPI_ISL_1301774, EPI_ISL_1301775, EPI_ISL_1301776, EPI_ISL_1301777, EPI_ISL_1301778, EPI_ISL_1301779, EPI_ISL_1301780, EPI_ISL_1301781, EPI_ISL_1301782, EPI_ISL_1301783, EPI_ISL_1301784, EPI_ISL_1301785, EPI_ISL_1301786, EPI_ISL_1301787, EPI_ISL_1301788, EPI_ISL_1301789, EPI_ISL_1301790, EPI_ISL_1301791, EPI_ISL_1301792, EPI_ISL_1301793, EPI_ISL_1301794, EPI_ISL_1301795, EPI_ISL_1301796, EPI_ISL_1301797, EPI_ISL_1301798, EPI_ISL_1301799, EPI_ISL_1301800, EPI_ISL_1301801, EPI_ISL_1301802, EPI_ISL_1301803, EPI_ISL_1302666, EPI_ISL_1302667, EPI_ISL_1302675, EPI_ISL_1302676, EPI_ISL_1302677, EPI_ISL_1302678, EPI_ISL_1302679, EPI_ISL_1302680, EPI_ISL_1302681, EPI_ISL_2362488, EPI_ISL_2362489, EPI_ISL_2362491, EPI_ISL_2362492, EPI_ISL_2362493, EPI_ISL_2362494, EPI_ISL_2362495, EPI_ISL_2362496, EPI_ISL_2362497, EPI_ISL_2362498, EPI_ISL_2362499, EPI_ISL_2362500, EPI_ISL_2362501, EPI_ISL_2362502, EPI_ISL_2362503, EPI_ISL_2362504, EPI_ISL_2362505, EPI_ISL_2362506, EPI_ISL_2362507, EPI_ISL_2362508, EPI_ISL_2362509, EPI_ISL_2362510, EPI_ISL_2362511, EPI_ISL_2362512, EPI_ISL_2362513, EPI_ISL_2362514, EPI_ISL_2362517, EPI_ISL_2365913, EPI_ISL_2365915, EPI_ISL_2521991, EPI_ISL_2522090, EPI_ISL_2522091			
see above	Nucleic Acid Testing, National Reference Laboratory	GIGA Medical Genomics	Bouchra Boujemla; Esperence Umumararungu; Jacob Souopgui; Keith Durkin; Léon Mutesa; Maria Artesi; Marie-Pierre Hayette; Nathalie Renotte; Patrick Tuyisenge; Robert Rutayisire; Sabin Nsanzimana; Swaibu Gatare; Sébastien Bontems; Vincent Bours; Yvan Butera
EPI_ISL_2241621, EPI_ISL_2372325, EPI_ISL_2372327, EPI_ISL_2372329, EPI_ISL_2372340, EPI_ISL_2372348, EPI_ISL_2372352, EPI_ISL_2372354, EPI_ISL_2372358, EPI_ISL_2372361, EPI_ISL_2372363, EPI_ISL_2372365, EPI_ISL_2372371, EPI_ISL_2372373, EPI_ISL_2372375, EPI_ISL_2372377, EPI_ISL_2372379, EPI_ISL_2372381, EPI_ISL_2372383, EPI_ISL_2372387, EPI_ISL_2372390, EPI_ISL_2372400, EPI_ISL_2372402			
see above	Nyangabwe Hospital HIV Reference Laboratory	Botswana Harvard HIV Reference Laboratory	Boitumelo Zuze; Botshelo Radibe; David Lawrence; Dorcas Maruapula; Joseph Makhema; Keoratile Ntshambiwa; Kgomotso Moruisi; Kwana Lechiile; Legodile Kooepile; Madisa Mine; Mosepele Mosepele; Ontlametse T. Bareng; Pamela Smith-Lawrence; Roger Shapiro; Shahin Lockman; Sikhulile Moyo; Sikhulile Wonderful T. Choga; Simani Gaseitsiwe; Thongbotho Mphoyakgosi; Wonderful T. Choga
EPI_ISL_1534271	Ocean View CDC wc OCE	NHLS/UCT	Arash Iranzadeh; Bruna Galvao; Carolyn Williamson; Deelan Doolabh; Diana Hardie; Innocent Mudau; Kruger Marais; Lynn Tyers; Marvin Hsiao; Stephen Korsman
EPI_ISL_1534277, EPI_ISL_1534293, EPI_ISL_1534344, EPI_ISL_1534378, EPI_ISL_1534456, EPI_ISL_2140665, EPI_ISL_2375943, EPI_ISL_2621083, EPI_ISL_2621089			
see above	Oudtshoorn Hospital wc OUD	NHLS/UCT	Arash Iranzadeh; Bruna Galvao; Carolyn Williamson; Deelan Doolabh; Diana Hardie; Emmanuel SJ; Gert Marais; Innocent Mudau; Kruger Marais; Lynn Tyers; Marvin Hsiao; Stephen Korsman; Tegally H; de Oliveira T
EPI_ISL_2140667, EPI_ISL_2621090	Oudtshoorn Sick Bay (SANDF)	NHLS/UCT	Arash Iranzadeh; Bruna Galvao; Carolyn Williamson; Deelan Doolabh; Diana Hardie; Gert Marais; Innocent Mudau; Kruger Marais; Lynn Tyers; Marvin Hsiao; Stephen Korsman
EPI_ISL_1301887, EPI_ISL_1301899, EPI_ISL_1301900	Outre mer	National Reference Center for Viruses of Respiratory Infections, Institut Pasteur, Paris	Angela Brisebarre; Camille Capel; Combe Patrice; Etienne Simon-Lorière; Louise Lefrançois; Marion Barbet; Maud Vanpeene; Méline Bizard; Sylvie Behillil; Sylvie van der Werf; Vincent Enouf
EPI_ISL_2086970	PATH CARE	National Institute for Communicable Diseases of the National Health Laboratory Service	Amoako DG; Bhiman JN; Ismail A; Mahlangu B; Mohale T; Ntuli N; Scheepers C
EPI_ISL_2285253, EPI_ISL_2285254, EPI_ISL_2285255, EPI_ISL_2285256, EPI_ISL_2285257, EPI_ISL_2285258, EPI_ISL_2285259, EPI_ISL_2285260, EPI_ISL_2285261, EPI_ISL_2285262, EPI_ISL_2285263, EPI_ISL_2285264, EPI_ISL_2285265, EPI_ISL_2285266, EPI_ISL_2285267, EPI_ISL_2285268, EPI_ISL_2285269, EPI_ISL_2285270, EPI_ISL_2442364, EPI_ISL_2442365, EPI_ISL_2582702, EPI_ISL_2582704, EPI_ISL_2582707, EPI_ISL_2582710, EPI_ISL_2582713, EPI_ISL_2582717, EPI_ISL_2582720			
see above	PORT ELIZABETH LABORATORY	National Institute for Communicable Diseases of the National Health Laboratory Service	Amoako DG; Bhiman JN; Everatt J; Ismail A; Mahlangu B; Mnguni A; Mohale T; Ntuli N; Scheepers C
EPI_ISL_2779535	PSTC	USAMRD-A, Basic Science Laboratory	Alan Lemtudo; Beth Mutai; Brian Andika; Carol Kifude; Clement Masakwe; Eric Muthanje; Esther Omuseni; Faith Sigei; Gathii Kimita; George Awinda; John Waitumbi; Josphat Nyataya; Rachel Githii; Rehema Liyai; Stephen Ochola
EPI_ISL_1534367, EPI_ISL_1534425, EPI_ISL_1534432, EPI_ISL_1534434, EPI_ISL_1534445, EPI_ISL_1534458, EPI_ISL_2375937, EPI_ISL_2375938, EPI_ISL_2375942, EPI_ISL_2621077, EPI_ISL_2802109, EPI_ISL_2802159			
see above	Pacaltsdorp Clinic wc PAC	NHLS/UCT	Arash Iranzadeh; Bruna Galvao; Carolyn Williamson; Deelan Doolabh; Diana Hardie; Emmanuel SJ; Gert Marais; Innocent Mudau; Kruger Marais; Lynn Tyers; Marvin Hsiao; Stephen Korsman; Tegally H; de Oliveira T
EPI_ISL_2241612, EPI_ISL_2241613, EPI_ISL_2241614, EPI_ISL_2241617	Palapye Primary Hospital Laboratory	Botswana Harvard HIV Reference Laboratory	Boitumelo Zuze; Botshelo Radibe; David Lawrence; Dorcas Maruapula; Joseph Makhema; Kwana Lechiile; Legodile Kooepile; Mosepele Mosepele; Roger Shapiro; Shahin Lockman; Sikhulile Wonderful T. Choga; Simani Gaseitsiwe; Thongbotho Mphoyakgosi
EPI_ISL_2035941, EPI_ISL_2035942, EPI_ISL_2035943, EPI_ISL_2035944	Pasteur Institute - Laboratory of Clinical Virology	Pasteur Institute - Laboratory of Clinical Virology	Anissa Chouikha; Henda Triki; Kais Ghedira; Mariem Gdoura; Sondos Haddad; Wasfi Fares
EPI_ISL_1534424, EPI_ISL_2621080, EPI_ISL_2802129	Plettenberg Bay Clinic wc PLC	NHLS/UCT	Arash Iranzadeh; Bruna Galvao; Carolyn Williamson; Deelan Doolabh; Diana Hardie; Emmanuel SJ; Gert Marais; Innocent Mudau; Kruger Marais; Lynn Tyers; Marvin Hsiao; Stephen Korsman; Tegally H; de Oliveira T
EPI_ISL_2447789, EPI_ISL_2447790, EPI_ISL_2447791, EPI_ISL_2447792, EPI_ISL_2447793, EPI_ISL_2447794, EPI_ISL_2447795, EPI_ISL_2447796, EPI_ISL_2447797, EPI_ISL_2447798, EPI_ISL_2447799, EPI_ISL_2447800, EPI_ISL_2447801, EPI_ISL_2447802, EPI_ISL_2447803, EPI_ISL_2447804, EPI_ISL_2447805, EPI_ISL_2447806, EPI_ISL_2447807, EPI_ISL_2447808, EPI_ISL_2447809, EPI_ISL_2447810, EPI_ISL_2447811, EPI_ISL_2447812, EPI_ISL_2447813, EPI_ISL_2447814, EPI_ISL_2447815, EPI_ISL_2447816, EPI_ISL_2447817, EPI_ISL_2447818, EPI_ISL_2447819, EPI_ISL_2447820, EPI_ISL_2447821, EPI_ISL_2447822, EPI_ISL_2447823			
see above	Port Elizabeth Laboratory	National Institute for Communicable Diseases of the National Health Laboratory Service	Amoako DG; Bhiman JN; Ismail A; Mahlangu B; Mohale T; Ntuli N; Scheepers C
EPI_ISL_2779317, EPI_ISL_2779418, EPI_ISL_2779419, EPI_ISL_2779420, EPI_ISL_2779421, EPI_ISL_2779424, EPI_ISL_2779425, EPI_ISL_2779426, EPI_ISL_2779427, EPI_ISL_2779428, EPI_ISL_2779429, EPI_ISL_2779430			
see above	Port Health Clinic, Busia border point	USAMRD-A, Basic Science Laboratory	Alan Lemtudo; Beth Mutai; Brian Andika; Carol Kifude; Clement Masakwe; Eric Muthanje; Esther Omuseni; Faith Sigei; Gathii Kimita; George Awinda; John Waitumbi; Josphat Nyataya; Rachel Githii; Rehema Liyai; Stephen Ochola
EPI_ISL_2779318, EPI_ISL_2779492, EPI_ISL_2779493, EPI_ISL_2779494, EPI_ISL_2779495, EPI_ISL_2779496, EPI_ISL_2779497, EPI_ISL_2779498, EPI_ISL_2779499, EPI_ISL_2779500, EPI_ISL_2779501, EPI_ISL_2779502, EPI_ISL_2779503, EPI_ISL_2779504, EPI_ISL_2779505, EPI_ISL_2779506, EPI_ISL_2779507, EPI_ISL_2779508, EPI_ISL_2779545			
see above	Port Health Clinic, Malaba border point	USAMRD-A, Basic Science Laboratory	Alan Lemtudo; Beth Mutai; Brian Andika; Carol Kifude; Clement Masakwe; Eric Muthanje; Esther Omuseni; Faith Sigei; Gathii Kimita; George Awinda; John Waitumbi; Josphat Nyataya; Rachel Githii; Rehema Liyai; Stephen Ochola
EPI_ISL_2860636, EPI_ISL_2860637, EPI_ISL_2860638	Public Health Reference Laboratory	Erasmus Medical Center	Anne van der Linden; Annemiek van der Eijk; Bas Oude Munnink; Corine GeurtsvanKessel; David Nieuwenhuijse; Emmanuelle Munger; Irina Chestakova; Marion Koopmans; Marjan Boter; Omar Elahmer-Abdulla Bashein- Rahma Algheriani-Ahlam Alarif; Reina Sikkema; Richard Molenkamp; on behalf of the Dutch national COVID-19 response team.
EPI_ISL_1628700, EPI_ISL_1628701, EPI_ISL_1628702, EPI_ISL_1628703, EPI_ISL_1628704, EPI_ISL_1628705, EPI_ISL_1628706, EPI_ISL_1628707, EPI_ISL_1628708, EPI_ISL_1628728, EPI_ISL_1628729, EPI_ISL_1628731, EPI_ISL_1628732, EPI_ISL_1628744, EPI_ISL_1628752, EPI_ISL_1628764, EPI_ISL_1628765, EPI_ISL_1628767, EPI_ISL_1628768, EPI_ISL_1628774, EPI_ISL_1628778, EPI_ISL_1628779, EPI_ISL_1628780, EPI_ISL_1628781, EPI_ISL_1628782, EPI_ISL_1628816, EPI_ISL_1628817, EPI_ISL_1628825, EPI_ISL_1628849, EPI_ISL_1628850, EPI_ISL_1628861, EPI_ISL_1628862, EPI_ISL_1628863, EPI_ISL_1628864, EPI_ISL_1628865, EPI_ISL_1628866, EPI_ISL_1628867, EPI_ISL_1628868, EPI_ISL_1628869, EPI_ISL_1628870, EPI_ISL_1628871, EPI_ISL_1628892, EPI_ISL_1628893, EPI_ISL_1628894, EPI_ISL_1628895, EPI_ISL_1628896, EPI_ISL_1628897, EPI_ISL_1628898, EPI_ISL_1628899, EPI_ISL_1628900, EPI_ISL_1628901, EPI_ISL_1628902, EPI_ISL_1628903, EPI_ISL_1628904, EPI_ISL_1628905, EPI_ISL_1628906, EPI_ISL_1628907, EPI_ISL_1628908, EPI_ISL_1628909, EPI_ISL_1628910, EPI_ISL_1628911, EPI_ISL_1628912, EPI_ISL_1628918, EPI_ISL_1629189, EPI_ISL_1629190, EPI_ISL_1629191, EPI_ISL_1629192, EPI_ISL_1629193, EPI_ISL_1629194, EPI_ISL_1629195, EPI_ISL_1629196, EPI_ISL_1629197, EPI_ISL_1629198, EPI_ISL_1629199, EPI_ISL_1629200, EPI_ISL_1629201, EPI_ISL_1629202, EPI_ISL_1629203, EPI_ISL_1629204, EPI_ISL_1629205, EPI_ISL_1629206, EPI_ISL_1629207, EPI_ISL_1629208, EPI_ISL_1629341, EPI_ISL_1629342, EPI_ISL_1629343, EPI_ISL_1629344, EPI_ISL_1629345, EPI_ISL_1629346, EPI_ISL_1629347, EPI_ISL_1629348, EPI_ISL_1629349, EPI_ISL_1629399, EPI_ISL_1629400, EPI_ISL_1629401, EPI_ISL_1629402, EPI_ISL_1629403, EPI_ISL_1629404, EPI_ISL_1629405, EPI_ISL_1629406, EPI_ISL_1629407, EPI_ISL_1629408, EPI_ISL_1629409, EPI_ISL_1629410, EPI_ISL_1629411, EPI_ISL_1629412, EPI_ISL_1629437, EPI_ISL_1629473, EPI_ISL_1629474, EPI_ISL_2090732, EPI_ISL_2090739, EPI_ISL_2090743, EPI_ISL_2090755, EPI_ISL_2090756, EPI_ISL_2090757, EPI_ISL_2090763, EPI_ISL_3153700			
see above	REUNILAB	UMR PIMIT	Dr Camille Lebarbenchon; Dr David A Wilkinson; Dr Patrick Mavingui; Magali Turpin; Marie-Alice Simbi
EPI_ISL_2382649, EPI_ISL_2382650, EPI_ISL_2382651, EPI_ISL_2382652, EPI_ISL_2382653, EPI_ISL_2382654	ROB FERREIRA HOSPITAL	National Institute for Communicable Diseases of the National Health Laboratory Service	Amoako DG; Bhiman JN; Ismail A; Mahlangu B; Mohale T; Ntuli N; Scheepers C
EPI_ISL_2086931, EPI_ISL_2086932, EPI_ISL_2086939, EPI_ISL_2086940, EPI_ISL_2086941, EPI_ISL_2086942, EPI_ISL_2086943, EPI_ISL_2086944, EPI_ISL_2086945, EPI_ISL_2086946, EPI_ISL_2086947, EPI_ISL_2086948, EPI_ISL_2086949, EPI_ISL_2086951, EPI_ISL_2086952, EPI_ISL_2086953, EPI_ISL_2086954, EPI_ISL_2086955, EPI_ISL_2086956, EPI_ISL_2086957, EPI_ISL_2086958, EPI_ISL_2086959, EPI_ISL_2086960, EPI_ISL_2086961, EPI_ISL_2086962, EPI_ISL_2086963, EPI_ISL_2086964, EPI_ISL_2086965, EPI_ISL_2086966, EPI_ISL_2086967, EPI_ISL_2153477, EPI_ISL_2153478, EPI_ISL_2153479, EPI_ISL_2284673, EPI_ISL_2284674, EPI_ISL_2284675, EPI_ISL_2284676, EPI_ISL_2284677, EPI_ISL_2284678, EPI_ISL_2284679, EPI_ISL_2284680, EPI_ISL_2284681, EPI_ISL_2284682, EPI_ISL_2284683, EPI_ISL_2284684, EPI_ISL_2284685, EPI_ISL_2284686, EPI_ISL_2284687, EPI_ISL_2284688, EPI_ISL_2284689, EPI_ISL_2284690, EPI_ISL_2284691, EPI_ISL_2285335, EPI_ISL_2285336, EPI_ISL_2442370, EPI_ISL_2442371, EPI_ISL_2442372, EPI_ISL_2442374, EPI_ISL_2442375, EPI_ISL_2442376, EPI_ISL_2442377, EPI_ISL_2442378, EPI_ISL_2442379, EPI_ISL_2442380, EPI_ISL_2442381, EPI_ISL_2442382, EPI_ISL_2444094, EPI_ISL_2444095, EPI_ISL_2444096, EPI_ISL_2444097, EPI_ISL_2444098, EPI_ISL_2444099, EPI_ISL_2444100, EPI_ISL_2444101, EPI_ISL_2444102, EPI_ISL_2444103, EPI_ISL_2444104, EPI_ISL_2444105, EPI_ISL_2444106, EPI_ISL_2444107, EPI_ISL_2444108, EPI_ISL_2444109, EPI_ISL_2444110, EPI_ISL_2444111, EPI_ISL_2444112, EPI_ISL_2444113, EPI_ISL_2444114, EPI_ISL_2444115, EPI_ISL_2444116, EPI_ISL_2444117, EPI_ISL_2444118, EPI_ISL_2444119, EPI_ISL_2444120, EPI_ISL_2444121, EPI_ISL_2444122, EPI_ISL_2444123, EPI_ISL_2444124, EPI_ISL_2444125, EPI_ISL_2444126, EPI_ISL_2444127, EPI_ISL_2444128, EPI_ISL_2444129, EPI_ISL_2444130, EPI_ISL_2444131, EPI_ISL_2444132, EPI_ISL_2444133, EPI_ISL_2444134, EPI_ISL_2444135, EPI_ISL_2444136, EPI_ISL_2454663, EPI_ISL_2582639, EPI_ISL_2582643, EPI_ISL_2582647, EPI_ISL_2582651, EPI_ISL_2582655, EPI_ISL_2582659, EPI_ISL_2582662, EPI_ISL_2582665, EPI_ISL_2582668, EPI_ISL_2582671, EPI_ISL_2582674, EPI_ISL_2582677, EPI_ISL_2582680, EPI_ISL_2582684, EPI_ISL_2582687, EPI_ISL_2582690, EPI_ISL_2582693, EPI_ISL_2582695, EPI_ISL_2582698, EPI_ISL_2662475			
see above	ROB FERREIRA LABORATORY	National Institute for Communicable Diseases of the National Health Laboratory Service	Amoako DG; Bhiman JN; Everatt J; Ismail A; Mahlangu B; Mnguni A; Mohale T; Ntuli N; Scheepers C
EPI_ISL_2348632, EPI_ISL_2348641, EPI_ISL_2348642, EPI_ISL_2348646	Rakai Health Sciences Program	MRC/UVRI & LSHTM Uganda Research Unit	Charles Ssuuna; Dan Lule Bugembe; Matthew Cotten; My V.T. Phan; Pontiano Kaleebu; Ronald Moses Galiwango; Steven J Reynolds
EPI_ISL_1534342, EPI_ISL_1534343, EPI_ISL_1534443	Red Cross Children's Hospital wc RXH	NHLS/UCT	Arash Iranzadeh; Bruna Galvao; Carolyn Williamson; Deelan Doolabh; Diana Hardie; Emmanuel SJ; Innocent Mudau; Kruger Marais; Lynn Tyers; Marvin Hsiao; Stephen Korsman; Tegally H; de Oliveira T
EPI_ISL_2272013	Red Cross Hospital	National Institute for Communicable Diseases of the	Amoako DG; Bhiman JN; Ismail A; Mahlangu B; Mohale T; Ntuli N; Scheepers C

		National Health Laboratory Service	
EPI_ISL_2110836, EPI_ISL_2110838, EPI_ISL_2110840, EPI_ISL_2110841, EPI_ISL_2110843, EPI_ISL_2110845, EPI_ISL_2110846, EPI_ISL_2110847, EPI_ISL_2110848, EPI_ISL_2110850, EPI_ISL_2110851, EPI_ISL_2110853, EPI_ISL_2110854, EPI_ISL_2110856, EPI_ISL_2110858, EPI_ISL_2110859			
see above	Reitor da Universidade Jean Piaget Guine-Bissau	MRCG at LSHTM, Genomics lab	Abdoulie Kanteh; Abdul Karim Sesay; Adul Candé; Aicha Balde; Aladje Balde; Bakary Sanyang; Bubacar Delgado Pinto Embaló; Dabiri Damilari; Edmira Maria da Costa; Erica Luis Maria Magalhães; Faatu Cassama; Mariama Kujabi; Milanca Agostinho Cá; Paulina Joãozinho da Costa Jarra Manneh; Rei José Pereira; Rui Inndi; Sainabou Laye Ndure; Simão Tchuda Bióté
EPI_ISL_1534375	Retreat CHC wc RHC	NHLS/UCT	Arash Iranzadeh; Bruna Galvao; Carolyn Williamson; Deelan Doolabh; Diana Hardie; Emmanuel SJ; Innocent Mudau; Kruger Marais; Lynn Tyers; Marvin Hsiao; Stephen Korsman; Tegally H; de Oliveira T
EPI_ISL_1534332, EPI_ISL_1534333, EPI_ISL_1534422	Riversdale Clinic wc RAV	NHLS/UCT	Arash Iranzadeh; Bruna Galvao; Carolyn Williamson; Deelan Doolabh; Diana Hardie; Emmanuel SJ; Innocent Mudau; Kruger Marais; Lynn Tyers; Marvin Hsiao; Stephen Korsman; Tegally H; de Oliveira T
EPI_ISL_2285240, EPI_ISL_2285241, EPI_ISL_2285242, EPI_ISL_2285243, EPI_ISL_2285244, EPI_ISL_2285248	Rob Ferreira	National Institute for Communicable Diseases of the National Health Laboratory Service	Amoako DG; Bhiman JN; Ismail A; Mahlangu B; Mohale T; Ntuli N; Scheepers C
EPI_ISL_2447825, EPI_ISL_2447831, EPI_ISL_2447832, EPI_ISL_2447836, EPI_ISL_2447838, EPI_ISL_2447840, EPI_ISL_2447841, EPI_ISL_2447842, EPI_ISL_2447843, EPI_ISL_2447844, EPI_ISL_2447846, EPI_ISL_2447847, EPI_ISL_2447848, EPI_ISL_2447849, EPI_ISL_2447850, EPI_ISL_2447851, EPI_ISL_2447852, EPI_ISL_2447853, EPI_ISL_2447854, EPI_ISL_2447855, EPI_ISL_2447856, EPI_ISL_2447857, EPI_ISL_2447858, EPI_ISL_2447859, EPI_ISL_2447861, EPI_ISL_2447862, EPI_ISL_2447863, EPI_ISL_2447865, EPI_ISL_2447866, EPI_ISL_2447867, EPI_ISL_2447868, EPI_ISL_2447869, EPI_ISL_2447870, EPI_ISL_2447871, EPI_ISL_2447872, EPI_ISL_2447873			
see above	Rob Ferreira Laboratory	National Institute for Communicable Diseases of the National Health Laboratory Service	Amoako DG; Bhiman JN; Ismail A; Mahlangu B; Mohale T; Ntuli N; Scheepers C
EPI_ISL_1628725, EPI_ISL_1628726, EPI_ISL_1628727, EPI_ISL_1628730, EPI_ISL_1628733, EPI_ISL_1628734, EPI_ISL_1628735, EPI_ISL_1628745, EPI_ISL_1628746, EPI_ISL_1629398, EPI_ISL_2090744, EPI_ISL_2090754, EPI_ISL_3153873, EPI_ISL_3154090, EPI_ISL_3154091			
see above	SAINT BENOIT	UMR PIMIT	Dr Camille Lebarbenchon; Dr David A Wilkinson; Dr Patrick Mavingui; Magali Turpin; Marie-Alice Simbi
EPI_ISL_2086909, EPI_ISL_2086914, EPI_ISL_2086916, EPI_ISL_2086917, EPI_ISL_2086918, EPI_ISL_2086919, EPI_ISL_2086920, EPI_ISL_2086925, EPI_ISL_2086926, EPI_ISL_2086927, EPI_ISL_2086928, EPI_ISL_2086929, EPI_ISL_2086930			
see above	Sebokeng Lab	National Institute for Communicable Diseases of the National Health Laboratory Service	Amoako DG; Bhiman JN; Ismail A; Mahlangu B; Mohale T; Ntuli N; Scheepers C
EPI_ISL_1534339, EPI_ISL_1534356, EPI_ISL_2621121	Sedgefield Clinic wc SGE	NHLS/UCT	Arash Iranzadeh; Bruna Galvao; Carolyn Williamson; Deelan Doolabh; Diana Hardie; Emmanuel SJ; Gert Marais; Innocent Mudau; Kruger Marais; Lynn Tyers; Marvin Hsiao; Stephen Korsman; Tegally H; de Oliveira T
EPI_ISL_2928025	South Sudan Ministry of Health, WHO South Sudan, MRC/UVRI & LSHTM Uganda Research Unit	MRC/UVRI & LSHTM Uganda Research Unit, South Sudan Ministry of Health, WHO South Sudan	Abe G. Abias; Dan Lule Bugembe; Dennis Kenyi Lodiongo; James Ayei; John Rumunu; Joseph Francis Wamala; Juma John HM; Lul Lojok Deng; Matthew Cotten; My V.T. Phan; Pontiano Kaleebu; Richard Lino Loro Lako; Sudhir Bunga
EPI_ISL_2662726, EPI_ISL_2662727, EPI_ISL_2662728, EPI_ISL_2662729, EPI_ISL_2716623, EPI_ISL_2716624, EPI_ISL_2716625, EPI_ISL_2716626, EPI_ISL_2716628, EPI_ISL_2716629, EPI_ISL_2716630, EPI_ISL_2716631, EPI_ISL_2716632, EPI_ISL_2716633, EPI_ISL_2716634, EPI_ISL_2716635, EPI_ISL_2716637, EPI_ISL_2716638, EPI_ISL_2716639			
see above	State Key Laboratory of Pathogen and Biosecurity, Beijing Institute of Microbiology and Epidemiology	State Key Laboratory of Pathogen and Biosecurity, Beijing Institute of Microbiology and Epidemiology	Campbell, A.; Campbell, J.; Gevao; Harding, D.; Lin, L.; Liu, Y.; P.F.; P.S.; Rogers, J.; Sahr; Wurie, I.; Zhang, J.
EPI_ISL_1534280, EPI_ISL_1534301, EPI_ISL_1534311, EPI_ISL_1534335, EPI_ISL_1534368, EPI_ISL_1534417, EPI_ISL_1534428			
see above	Still Bay Sat Clinic wc SFV	NHLS/UCT	Arash Iranzadeh; Bruna Galvao; Carolyn Williamson; Deelan Doolabh; Diana Hardie; Emmanuel SJ; Innocent Mudau; Kruger Marais; Lynn Tyers; Marvin Hsiao; Stephen Korsman; Tegally H; de Oliveira T
EPI_ISL_2153480, EPI_ISL_2153481, EPI_ISL_2153482, EPI_ISL_2662486, EPI_ISL_2662487, EPI_ISL_2662488, EPI_ISL_2662489, EPI_ISL_2662490, EPI_ISL_2662491			
see above	TAMBO MEMORIAL LABORATORY	National Institute for Communicable Diseases of the National Health Laboratory Service	Amoako DG; Bhiman JN; Everatt J; Ismail A; Mahlangu B; Mnguni A; Mohale T; Ntuli N; Scheepers C
EPI_ISL_2086950, EPI_ISL_2285271, EPI_ISL_2285272, EPI_ISL_2285273, EPI_ISL_2285275, EPI_ISL_2285276, EPI_ISL_2285277, EPI_ISL_2285278, EPI_ISL_2285279, EPI_ISL_2285281, EPI_ISL_2285282, EPI_ISL_2285283, EPI_ISL_2285284, EPI_ISL_2285285, EPI_ISL_2285286, EPI_ISL_2285287, EPI_ISL_2285288, EPI_ISL_2285289, EPI_ISL_2285290, EPI_ISL_2285291, EPI_ISL_2285292, EPI_ISL_2285293, EPI_ISL_2285294, EPI_ISL_2285295, EPI_ISL_2285296, EPI_ISL_2285297, EPI_ISL_2285298, EPI_ISL_2285299, EPI_ISL_2285300, EPI_ISL_2285301, EPI_ISL_2285302, EPI_ISL_2285303, EPI_ISL_2285304, EPI_ISL_2285305, EPI_ISL_2285306, EPI_ISL_2285307, EPI_ISL_2285308, EPI_ISL_2285309, EPI_ISL_2285310, EPI_ISL_2285311, EPI_ISL_2285312, EPI_ISL_2285313			
see above	TSHWANE ACADEMIC LABORATORY	National Institute for Communicable Diseases of the National Health Laboratory Service	Amoako DG; Bhiman JN; Ismail A; Mahlangu B; Mohale T; Ntuli N; Scheepers C
EPI_ISL_2140654, EPI_ISL_2140661, EPI_ISL_2140662, EPI_ISL_2375936, EPI_ISL_2375939, EPI_ISL_2375940, EPI_ISL_2375941, EPI_ISL_2621074, EPI_ISL_2621078, EPI_ISL_2621081, EPI_ISL_2621088, EPI_ISL_2621094			
see above	Thembalethu CDC wc THC	NHLS/UCT	Arash Iranzadeh; Bruna Galvao; Carolyn Williamson; Deelan Doolabh; Diana Hardie; Gert Marais; Innocent Mudau; Kruger Marais; Lynn Tyers; Marvin Hsiao; Stephen Korsman
EPI_ISL_2271958, EPI_ISL_2271959, EPI_ISL_2271960, EPI_ISL_2271961, EPI_ISL_2271962, EPI_ISL_2271963, EPI_ISL_2271969, EPI_ISL_2271975, EPI_ISL_2271985, EPI_ISL_2272009, EPI_ISL_2272012			
see above	Tshepong Hospital	National Institute for Communicable Diseases of the National Health Laboratory Service	Amoako DG; Bhiman JN; Ismail A; Mahlangu B; Mohale T; Ntuli N; Scheepers C
EPI_ISL_2447824, EPI_ISL_2447833	Tshepong Laboratory	National Institute for Communicable Diseases of the National Health Laboratory Service	Amoako DG; Bhiman JN; Ismail A; Mahlangu B; Mohale T; Ntuli N; Scheepers C
EPI_ISL_2447874	Tshwane Academic Laboratory	National Institute for Communicable Diseases of the National Health Laboratory Service	Amoako DG; Bhiman JN; Ismail A; Mahlangu B; Mohale T; Ntuli N; Scheepers C
EPI_ISL_1324121, EPI_ISL_1324122, EPI_ISL_1324123, EPI_ISL_1324124, EPI_ISL_1324125, EPI_ISL_1324126, EPI_ISL_1324127, EPI_ISL_1324128, EPI_ISL_1324129, EPI_ISL_1324130, EPI_ISL_1324131			
see above	UW Virology Lab	UW Virology Lab	Alexander Greninger; Hong Xie; Keith R Jerome; Lasata Shrestha; Margaret Mills; Meei-Li Huang; Michelle Lin; Noah Baker; Pavitra Roychoudhury; Saraswathi Sathees; Sean Ellis; Shah Mohamed Bakhash
EPI_ISL_1534269, EPI_ISL_1534372	Uniondale Hospital wc UDH	NHLS/UCT	Arash Iranzadeh; Bruna Galvao; Carolyn Williamson; Deelan Doolabh; Diana Hardie; Emmanuel SJ; Innocent Mudau; Kruger Marais; Lynn Tyers; Marvin Hsiao; Stephen Korsman; Tegally H; de Oliveira T
EPI_ISL_2864611, EPI_ISL_2864613, EPI_ISL_2864615, EPI_ISL_2864619, EPI_ISL_2864621, EPI_ISL_2864623, EPI_ISL_2864628, EPI_ISL_2864636, EPI_ISL_2864638, EPI_ISL_2864642, EPI_ISL_2864644, EPI_ISL_2864646, EPI_ISL_2864648, EPI_ISL_2864650, EPI_ISL_2864652, EPI_ISL_2864657, EPI_ISL_2864659, EPI_ISL_2864661, EPI_ISL_2864663, EPI_ISL_2864665, EPI_ISL_2864669, EPI_ISL_2864671, EPI_ISL_2864673, EPI_ISL_2864676, EPI_ISL_2864684			
see above	University of Health and Allied Sciences (UHAS) COVID-19 Testing and Research Centre	University of Health and Allied Sciences (UHAS) COVID-19 Testing and Research Centre	John O. Gyapong and the UHAS COVID-19 Lab Team; Jones Gyamfi; Kwabena O. Duedu; Reuben Ayivor-Djanie
EPI_ISL_2241625, EPI_ISL_2241756, EPI_ISL_2241981, EPI_ISL_2242183, EPI_ISL_2242506, EPI_ISL_2242809, EPI_ISL_2243020, EPI_ISL_2243223, EPI_ISL_2243408			
see above	University of Maiduguri Teaching Hospital	International Centre for Genetic Engineering and Biotechnology (ICGEB) and ARGO Open Lab for Genome Sequencing	Alessandro Marcello; Bamidele S Oderinde; Danilo Licastro; Emanuele Orsini; Galadima Gadzama; Marycelin M Baba; Monilade Akinola; Simeone Dal Monego; Zara Wuduri
EPI_ISL_2243550	University of Maiduguri Teaching Hospital, P.M.B. 1414	International Centre for Genetic Engineering and Biotechnology (ICGEB) and ARGO Open Lab for Genome Sequencing	Alessandro Marcello; Bamidele S Oderinde; Danilo Licastro; Emanuele Orsini; Galadima Gadzama; Marycelin M Baba; Monilade Akinola; Simeone Dal Monego; Zara Wuduri
EPI_ISL_2803312, EPI_ISL_2803313, EPI_ISL_2803314, EPI_ISL_2803315, EPI_ISL_2803316, EPI_ISL_2803317, EPI_ISL_2803318, EPI_ISL_2803319, EPI_ISL_2803320, EPI_ISL_2803321, EPI_ISL_2803322, EPI_ISL_2803323, EPI_ISL_2803324, EPI_ISL_2803325, EPI_ISL_2803326, EPI_ISL_2803327, EPI_ISL_2803328, EPI_ISL_2803329, EPI_ISL_2803330, EPI_ISL_2803331, EPI_ISL_2803332, EPI_ISL_2803333, EPI_ISL_2803334, EPI_ISL_2803335, EPI_ISL_2803336, EPI_ISL_2803337, EPI_ISL_2803338, EPI_ISL_2803339, EPI_ISL_2803340, EPI_ISL_2803341, EPI_ISL_2803342, EPI_ISL_2803343, EPI_ISL_2803344, EPI_ISL_2803345, EPI_ISL_2803346, EPI_ISL_2803347, EPI_ISL_2803348, EPI_ISL_2803349, EPI_ISL_2803350, EPI_ISL_2803351, EPI_ISL_2803352, EPI_ISL_2803353, EPI_ISL_2803354, EPI_ISL_2803355, EPI_ISL_2803356, EPI_ISL_2803357, EPI_ISL_2803358, EPI_ISL_2803359, EPI_ISL_2803360, EPI_ISL_2803361, EPI_ISL_2803362, EPI_ISL_2803363, EPI_ISL_2803364, EPI_ISL_2803365, EPI_ISL_2803366, EPI_ISL_2803367, EPI_ISL_2803368, EPI_ISL_2803369, EPI_ISL_2803370, EPI_ISL_2803371, EPI_ISL_2803372, EPI_ISL_2803373, EPI_ISL_2803374, EPI_ISL_2803375, EPI_ISL_2803376, EPI_ISL_2803377, EPI_ISL_2803378, EPI_ISL_2803379, EPI_ISL_2803380, EPI_ISL_2803381, EPI_ISL_2803382, EPI_ISL_2803383, EPI_ISL_2803384, EPI_ISL_2803385, EPI_ISL_2803386, EPI_ISL_2803387, EPI_ISL_2803388, EPI_ISL_2803389, EPI_ISL_2803390, EPI_ISL_2803391, EPI_ISL_2803392, EPI_ISL_2803393, EPI_ISL_2803394, EPI_ISL_2803395, EPI_ISL_2803396, EPI_ISL_2803397, EPI_ISL_2803398, EPI_ISL_2803399, EPI_ISL_2803400, EPI_ISL_2803401, EPI_ISL_2803402, EPI_ISL_2803403, EPI_ISL_2803404, EPI_ISL_2803405, EPI_ISL_2803406, EPI_ISL_2803407, EPI_ISL_2803408, EPI_ISL_2803409, EPI_ISL_2803410, EPI_ISL_2803411, EPI_ISL_2803412, EPI_ISL_2803413, EPI_ISL_2803414, EPI_ISL_2803415, EPI_ISL_2803416, EPI_ISL_2803417, EPI_ISL_2803418, EPI_ISL_2803419, EPI_ISL_2803420, EPI_ISL_2803421, EPI_ISL_2803422, EPI_ISL_2803423, EPI_ISL_2803424, EPI_ISL_2803425, EPI_ISL_2803426, EPI_ISL_2803427, EPI_ISL_2803428, EPI_ISL_2803429, EPI_ISL_2803430, EPI_ISL_2803431, EPI_ISL_2803432, EPI_ISL_2803433, EPI_ISL_2803434, EPI_ISL_2803435, EPI_ISL_2803436, EPI_ISL_2803437, EPI_ISL_2803438, EPI_ISL_2803439, EPI_ISL_2803440, EPI_ISL_2803441, EPI_ISL_2803442, EPI_ISL_2803443, EPI_ISL_2803444, EPI_ISL_2803445, EPI_ISL_2803446, EPI_ISL_2803447, EPI_ISL_2803448, EPI_ISL_2803449, EPI_ISL_2803452, EPI_ISL_2803453, EPI_ISL_2803454, EPI_ISL_2803455, EPI_ISL_2803456, EPI_ISL_2803457, EPI_ISL_2803458, EPI_ISL_2803459, EPI_ISL_2803460, EPI_ISL_2803461, EPI_ISL_2803462, EPI_ISL_2803463, EPI_ISL_2803464, EPI_ISL_2803465, EPI_ISL_2803466, EPI_ISL_2803467, EPI_ISL_2803468, EPI_ISL_2803469, EPI_ISL_2803470, EPI_ISL_2803471, EPI_ISL_2803472, EPI_ISL_2803473, EPI_ISL_2803474, EPI_ISL_2803475, EPI_ISL_2803476, EPI_ISL_2803477, EPI_ISL_2803478, EPI_ISL_2803479, EPI_ISL_2803480, EPI_ISL_2803481, EPI_ISL_2803482, EPI_ISL_2803483, EPI_ISL_2803484, EPI_ISL_2803485, EPI_ISL_2803486, EPI_ISL_2803487, EPI_ISL_2803488, EPI_ISL_2803489, EPI_ISL_2803490, EPI_ISL_2803491, EPI_ISL_2803492, EPI_ISL_2803493, EPI_ISL_2803494, EPI_ISL_2803495, EPI_ISL_2803496, EPI_ISL_2803497, EPI_ISL_2803498, EPI_ISL_2803499, EPI_ISL_2803500, EPI_ISL_2803501, EPI_ISL_2803502, EPI_ISL_2803503, EPI_ISL_2803504, EPI_ISL_2803505, EPI_ISL_2803506, EPI_ISL_2803507, EPI_ISL_2803508, EPI_ISL_2803509, EPI_ISL_2803510, EPI_ISL_2803511, EPI_ISL_2803512, EPI_ISL_2803513, EPI_ISL_2803514, EPI_ISL_2803515, EPI_ISL_2803516, EPI_ISL_2803517, EPI_ISL_2803518, EPI_ISL_2803519, EPI_ISL_2803520, EPI_ISL_2803521, EPI_ISL_2803522, EPI_ISL_2803523, EPI_ISL_2803524, EPI_ISL_2803525, EPI_ISL_2803526, EPI_ISL_2803527, EPI_ISL_2803528, EPI_ISL_2803529, EPI_ISL_2803530, EPI_ISL_2803531, EPI_ISL_2803532, EPI_ISL_2803533, EPI_ISL_2803534, EPI_ISL_2803535, EPI_ISL_2803536, EPI_ISL_2803537, EPI_ISL_2803538, EPI_ISL_2803539, EPI_ISL_2803540, EPI_ISL_2803541, EPI_ISL_2803542, EPI_ISL_2803543, EPI_ISL_2803544, EPI_ISL_2803545, EPI_ISL_2803546, EPI_ISL_2803547, EPI_ISL_2803548, EPI_ISL_2803549, EPI_ISL_2803550, EPI_ISL_2803551, EPI_ISL_2803552, EPI_ISL_2803553, EPI_ISL_2803554, EPI_ISL_2803555, EPI_ISL_2803556, EPI_ISL_2803557, EPI_ISL_2803558, EPI_ISL_2803559, EPI_ISL_2803560, EPI_ISL_2803561, EPI_ISL_2803562, EPI_ISL_2803563, EPI_ISL_2803564, EPI_ISL_2803565, EPI_ISL_2803566, EPI_ISL_2803567, EPI_ISL_2803568, EPI_ISL_2803569, EPI_ISL_2803570, EPI_ISL_2803571, EPI_ISL_2803572, EPI_ISL_2803573, EPI_ISL_2803574, EPI_ISL_2803575, EPI_ISL_2803576, EPI_ISL_2803577, EPI_ISL_2803578, EPI_ISL_2803579, EPI_ISL_2803580, EPI_ISL_2803581, EPI_ISL_2803582, EPI_ISL_2803583, EPI_ISL_2803584, EPI_ISL_2803585, EPI_ISL_2803586, EPI_ISL_2803587, EPI_ISL_2803588, EPI_ISL_2803589, EPI_ISL_2803590, EPI_ISL_2803591, EPI_ISL_2803592, EPI_ISL_2803593, EPI_ISL_2803594, EPI_ISL_2803595, EPI_ISL_2803596, EPI_ISL_2803597, EPI_ISL_2803598, EPI_ISL_2803599, EPI_ISL_2803600, EPI_ISL_2803601, EPI_ISL_2803602, EPI_ISL_2803603, EPI_ISL_2803604, EPI_ISL_2803605, EPI_ISL_2803606, EPI_ISL_2803607, EPI_ISL_2803608, EPI_ISL_2803609, EPI_ISL_2803610, EPI_ISL_2803611, EPI_ISL_2803612, EPI_ISL_2803613, EPI_ISL_2803614, EPI_ISL_2803615, EPI_ISL_2803616, EPI_ISL_2803618, EPI_ISL_2803619, EPI_ISL_2803620, EPI_ISL_2803621, EPI_ISL_2803622, EPI_ISL_2803623, EPI_ISL_2803624, EPI_ISL_2803625, EPI_ISL_2803626, EPI_ISL_2803627, EPI_ISL_2803628, EPI_ISL_2803633, EPI_ISL_2803634, EPI_ISL_2803640, EPI_ISL_2803641, EPI_ISL_2803642, EPI_ISL_2803643, EPI_ISL_2803644, EPI_ISL_2803647, EPI_ISL_2803648, EPI_ISL_2803649, EPI_ISL_2803650, EPI_ISL_2803651, EPI_ISL_2803652, EPI_ISL_2803653, EPI_ISL_2803654, EPI_ISL_2803655, EPI_ISL_2803656, EPI_ISL_2803657, EPI_ISL_2803658, EPI_ISL_2803659, EPI_ISL_2803660			
see above	University of Zambia, School of Veterinary Medicine	UNZAVET and PATH	Daniel Bridges; Mulenga Mwenda-Chimfwembe; Ngonda Saasa; ZNPHI and ZGSC
EPI_ISL_2382656, EPI_ISL_2382657, EPI_ISL_2382658, EPI_ISL_2382659, EPI_ISL_2382660, EPI_ISL_2382661, EPI_ISL_2382662			
see above	VERMAAK	National Institute for Communicable Diseases of the National Health Laboratory Service	Amoako DG; Bhiman JN; Ismail A; Mahlangu B; Mohale T; Ntuli N; Scheepers C
EPI_ISL_2662476, EPI_ISL_2662477, EPI_ISL_2662478, EPI_ISL_2662479, EPI_ISL_2662480, EPI_ISL_2662481, EPI_ISL_2662482, EPI_ISL_2662483, EPI_ISL_2662484, EPI_ISL_2662485, EPI_ISL_2662529, EPI_ISL_2662530, EPI_ISL_2662531, EPI_ISL_2662532, EPI_ISL_2662533, EPI_ISL_2662534, EPI_ISL_2662535, EPI_ISL_2662536, EPI_ISL_2662537			
see above	VERMAAK LABORATORY	National Institute for Communicable Diseases of the National Health Laboratory Service	Amoako DG; Bhiman JN; Everatt J; Ismail A; Mahlangu B; Mnguni A; Mohale T; Ntuli N; Scheepers C
EPI_ISL_2494710, EPI_ISL_2494711, EPI_ISL_2494712, EPI_ISL_2494713, EPI_ISL_2494715, EPI_ISL_2494716, EPI_ISL_2494717, EPI_ISL_2494718, EPI_ISL_2494719, EPI_ISL_2494721, EPI_ISL_2494722, EPI_ISL_2494723, EPI_ISL_2494724, EPI_ISL_2494725, EPI_ISL_2494726, EPI_ISL_2494727, EPI_ISL_2494728, EPI_ISL_2494730, EPI_ISL_2494731, EPI_ISL_2494732, EPI_ISL_2494733, EPI_ISL_2494734, EPI_ISL_2494735, EPI_ISL_2494736, EPI_ISL_2494737, EPI_ISL_2494738, EPI_ISL_2494740, EPI_ISL_2494741, EPI_ISL_2494742, EPI_ISL_2494744, EPI_ISL_2494745, EPI_ISL_2494746, EPI_ISL_2494747, EPI_ISL_2494748, EPI_ISL_2494749, EPI_ISL_2494750, EPI_ISL_2494751, EPI_ISL_2494752, EPI_ISL_2494754, EPI_ISL_2494756, EPI_ISL_2494757, EPI_ISL_2494758, EPI_ISL_2494759, EPI_ISL_2494760, EPI_ISL_2494761, EPI_ISL_2494762, EPI_ISL_2494763, EPI_ISL_2494764, EPI_ISL_2494765, EPI_ISL_2494766, EPI_ISL_2494767, EPI_ISL_2494768, EPI_ISL_2494769, EPI_ISL_2494776, EPI_ISL_2494777, EPI_ISL_2494783, EPI_ISL_2494788, EPI_ISL_2494831, EPI_ISL_2494859, EPI_ISL_2494864, EPI_ISL_2494874, EPI_ISL_2494898, EPI_ISL_2494901, EPI_ISL_2494902, EPI_ISL_2494903, EPI_ISL_2494912, EPI_ISL_2494913, EPI_ISL_2494915, EPI_ISL_2494916, EPI_ISL_2494917, EPI_ISL_2494918, EPI_ISL_2494919, EPI_ISL_2494920, EPI_ISL_2494921, EPI_ISL_2494922, EPI_ISL_2494925, EPI_ISL_2494926, EPI_ISL_2494928, EPI_ISL_2494929, EPI_ISL_2494932, EPI_ISL_2494933, EPI_ISL_2494934, EPI_ISL_2494938, EPI_ISL_2494941, EPI_ISL_2494942, EPI_ISL_2494946, EPI_ISL_2494947, EPI_ISL_2494949, EPI_ISL_2494950, EPI_ISL_2494952, EPI_ISL_2494953, EPI_ISL_2494955, EPI_ISL_2494956, EPI_ISL_2494957, EPI_ISL_2494958, EPI_ISL_2494962, EPI_ISL_2494963, EPI_ISL_2494964, EPI_ISL_2494967, EPI_ISL_2494968, EPI_ISL_2494969, EPI_ISL_2494970, EPI_ISL_2494971, EPI_ISL_2494973, EPI_ISL_2494974, EPI_ISL_2494976, EPI_ISL_2494977, EPI_ISL_2494979, EPI_ISL_2494980, EPI_ISL_2494981, EPI_ISL_2494982, EPI_ISL_2494983, EPI_ISL_2494984, EPI_ISL_2494985, EPI_ISL_2494986, EPI_ISL_2494987, EPI_ISL_2494988, EPI_ISL_2494989, EPI_ISL_2494990, EPI_ISL_2494991, EPI_ISL_2494992, EPI_ISL_2494993, EPI_ISL_2494994, EPI_ISL_2494995, EPI_ISL_2494996, EPI_ISL_2494997, EPI_ISL_2494999, EPI_ISL_2495000, EPI_ISL_2495001, EPI_ISL_2495002, EPI_ISL_2495003, EPI_ISL_2495004, EPI_ISL_2495005, EPI_ISL_2495006, EPI_ISL_2495007, EPI_ISL_2495008, EPI_ISL_2495009, EPI_ISL_2495010, EPI_ISL_2495011, EPI_ISL_2495012, EPI_ISL_2495013, EPI_ISL_2495015, EPI_ISL_2495016, EPI_ISL_2495017, EPI_ISL_2495018, EPI_ISL_2495019, EPI_ISL_2495020, EPI_ISL_2495021, EPI_ISL_2495022, EPI_ISL_2495024, EPI_ISL_2495025, EPI_ISL_2495026, EPI_ISL_2495027, EPI_ISL_2495029, EPI_ISL_2495030, EPI_ISL_2495031, EPI_ISL_2495033, EPI_ISL_2495054, EPI_ISL_2495056			

see above	Vaccines and Infectious Diseases Analytics Research Unit (VIDA)	KRISP, KZN Research Innovation and Sequencing Platform	Baillie Vicky; Giandhari Jennifer; Madhi Shabir; Naidoo Yeshnee; Pillay Sureshnee; San James; Tegally Houriiyah; Wilkinson Eduan; de Oliveira Tulio; du Plessis Jeanine
EPI_ISL_2360343, EPI_ISL_2360344, EPI_ISL_2360345, EPI_ISL_2360346, EPI_ISL_2360347, EPI_ISL_2360348, EPI_ISL_2360349, EPI_ISL_2360350, EPI_ISL_2360351, EPI_ISL_2360352, EPI_ISL_2360353, EPI_ISL_2360354, EPI_ISL_2360355, EPI_ISL_2360356, EPI_ISL_2360357, EPI_ISL_2360358, EPI_ISL_2360359, EPI_ISL_2360360, EPI_ISL_2360361, EPI_ISL_2360362, EPI_ISL_2360363, EPI_ISL_2360364, EPI_ISL_2360365, EPI_ISL_2360366, EPI_ISL_2360367, EPI_ISL_2360368, EPI_ISL_2360369, EPI_ISL_2360370, EPI_ISL_2360371, EPI_ISL_2360372, EPI_ISL_2360373, EPI_ISL_2360374, EPI_ISL_2360375, EPI_ISL_2360376, EPI_ISL_2360377, EPI_ISL_2360378, EPI_ISL_2360379, EPI_ISL_2360380, EPI_ISL_2360381, EPI_ISL_2360382, EPI_ISL_2360383, EPI_ISL_2360384, EPI_ISL_2360385, EPI_ISL_2360386, EPI_ISL_2360387, EPI_ISL_2360388, EPI_ISL_2360389, EPI_ISL_2360390, EPI_ISL_2360391, EPI_ISL_2360392, EPI_ISL_2360393, EPI_ISL_2360394, EPI_ISL_2360395, EPI_ISL_2360396, EPI_ISL_2360397, EPI_ISL_2360398, EPI_ISL_2360399, EPI_ISL_2360400, EPI_ISL_2360401, EPI_ISL_2360402, EPI_ISL_2360403, EPI_ISL_2360404, EPI_ISL_2360405, EPI_ISL_2360406, EPI_ISL_2360407, EPI_ISL_2360408, EPI_ISL_2360409, EPI_ISL_2360410, EPI_ISL_2360411, EPI_ISL_2360412, EPI_ISL_2360413, EPI_ISL_2360414, EPI_ISL_2360415, EPI_ISL_2360416, EPI_ISL_2360417, EPI_ISL_2360418, EPI_ISL_2360419, EPI_ISL_2360420, EPI_ISL_2360421, EPI_ISL_2360422, EPI_ISL_2360423, EPI_ISL_2360424, EPI_ISL_2360425, EPI_ISL_2360426, EPI_ISL_2360427, EPI_ISL_2360428, EPI_ISL_2360429, EPI_ISL_2360430, EPI_ISL_2360431, EPI_ISL_2360432, EPI_ISL_2360433, EPI_ISL_2360434, EPI_ISL_2360435, EPI_ISL_2360436, EPI_ISL_2360437, EPI_ISL_2360438, EPI_ISL_2360439, EPI_ISL_2360440, EPI_ISL_2360441, EPI_ISL_2360442, EPI_ISL_2360443, EPI_ISL_2360444, EPI_ISL_2360445, EPI_ISL_2360446, EPI_ISL_2360447, EPI_ISL_2360448, EPI_ISL_2360449, EPI_ISL_2360450, EPI_ISL_2360451, EPI_ISL_2360452, EPI_ISL_2360453, EPI_ISL_2360454, EPI_ISL_2360455, EPI_ISL_2360456, EPI_ISL_2360457, EPI_ISL_2360458, EPI_ISL_2360459, EPI_ISL_2360460, EPI_ISL_2360461, EPI_ISL_2360462, EPI_ISL_2360463, EPI_ISL_2360464, EPI_ISL_2360465, EPI_ISL_2360466, EPI_ISL_2360467, EPI_ISL_2360468, EPI_ISL_2360469, EPI_ISL_2360470, EPI_ISL_2360471, EPI_ISL_2360472, EPI_ISL_2360473, EPI_ISL_2360474, EPI_ISL_2360475, EPI_ISL_2360476, EPI_ISL_2360477, EPI_ISL_2360478, EPI_ISL_2360479, EPI_ISL_2360480, EPI_ISL_2360481, EPI_ISL_2360482, EPI_ISL_2360483, EPI_ISL_2360484, EPI_ISL_2360485, EPI_ISL_2360486, EPI_ISL_2360487, EPI_ISL_2360488, EPI_ISL_2360489, EPI_ISL_2360490, EPI_ISL_2360491, EPI_ISL_2360492, EPI_ISL_2360493, EPI_ISL_2360494, EPI_ISL_2360495, EPI_ISL_2360496, EPI_ISL_2360497, EPI_ISL_2360498, EPI_ISL_2360499, EPI_ISL_2360500, EPI_ISL_2360501, EPI_ISL_2360502, EPI_ISL_2360503, EPI_ISL_2360504, EPI_ISL_2360505, EPI_ISL_2360506, EPI_ISL_2360507, EPI_ISL_2360508, EPI_ISL_2360509, EPI_ISL_2360510, EPI_ISL_2360511, EPI_ISL_2360512, EPI_ISL_2360513, EPI_ISL_2360514, EPI_ISL_2360515, EPI_ISL_2360516, EPI_ISL_2360517, EPI_ISL_2360518, EPI_ISL_2360519, EPI_ISL_2360520, EPI_ISL_2360521, EPI_ISL_2360522, EPI_ISL_2360523, EPI_ISL_2360524, EPI_ISL_2360525, EPI_ISL_2360526, EPI_ISL_2360527, EPI_ISL_2360528, EPI_ISL_2360529, EPI_ISL_2360530, EPI_ISL_2360531, EPI_ISL_2360532, EPI_ISL_2360533, EPI_ISL_2360534, EPI_ISL_2360535, EPI_ISL_2360536, EPI_ISL_2360537, EPI_ISL_2360538, EPI_ISL_2360539, EPI_ISL_2360540, EPI_ISL_2360541, EPI_ISL_2360542, EPI_ISL_2360543, EPI_ISL_2360544, EPI_ISL_2360545, EPI_ISL_2360546, EPI_ISL_2360547, EPI_ISL_2360548, EPI_ISL_2360549, EPI_ISL_2360550, EPI_ISL_2360551, EPI_ISL_2360552, EPI_ISL_2360553, EPI_ISL_2360554, EPI_ISL_2360555, EPI_ISL_2360556, EPI_ISL_2360557, EPI_ISL_2360558, EPI_ISL_2360559, EPI_ISL_2360560, EPI_ISL_2360561, EPI_ISL_2360562, EPI_ISL_2360563, EPI_ISL_2360564, EPI_ISL_2360565, EPI_ISL_2360566, EPI_ISL_2360567, EPI_ISL_2360568, EPI_ISL_2360569, EPI_ISL_2360570, EPI_ISL_2360571, EPI_ISL_2360572, EPI_ISL_2360573, EPI_ISL_2360574, EPI_ISL_2360575, EPI_ISL_2360576, EPI_ISL_2360577, EPI_ISL_2360578, EPI_ISL_2360579, EPI_ISL_2360580, EPI_ISL_2360581, EPI_ISL_2360582, EPI_ISL_2360583, EPI_ISL_2360584, EPI_ISL_2360585, EPI_ISL_2360586, EPI_ISL_2360587, EPI_ISL_2360588, EPI_ISL_2360589, EPI_ISL_2360590, EPI_ISL_2360591, EPI_ISL_2360592, EPI_ISL_2360593, EPI_ISL_2360594, EPI_ISL_2360595, EPI_ISL_2360596, EPI_ISL_2360597, EPI_ISL_2360598, EPI_ISL_2360599, EPI_ISL_2360600, EPI_ISL_2360601, EPI_ISL_2360602, EPI_ISL_2360603, EPI_ISL_2360604, EPI_ISL_2360605, EPI_ISL_2360606, EPI_ISL_2360607, EPI_ISL_2360608, EPI_ISL_2360609, EPI_ISL_2360610, EPI_ISL_2360611, EPI_ISL_2360612, EPI_ISL_2360613, EPI_ISL_2360614, EPI_ISL_2360615, EPI_ISL_2360616, EPI_ISL_2360617, EPI_ISL_2360618, EPI_ISL_2360619, EPI_ISL_2360620, EPI_ISL_2360621, EPI_ISL_2360622, EPI_ISL_2360623, EPI_ISL_2360624, EPI_ISL_2360625, EPI_ISL_2360626, EPI_ISL_2360627, EPI_ISL_2360628, EPI_ISL_2360629, EPI_ISL_2360630, EPI_ISL_2360631, EPI_ISL_2360632, EPI_ISL_2360633, EPI_ISL_2360634, EPI_ISL_2360635, EPI_ISL_2360636, EPI_ISL_2360637, EPI_ISL_2360638, EPI_ISL_2360639, EPI_ISL_2360640, EPI_ISL_2360641, EPI_ISL_2360642, EPI_ISL_2360643, EPI_ISL_2360644, EPI_ISL_2360645, EPI_ISL_2360646, EPI_ISL_2360647, EPI_ISL_2360648, EPI_ISL_2360649, EPI_ISL_2360650, EPI_ISL_2360651, EPI_ISL_2360652, EPI_ISL_2360653, EPI_ISL_2360654, EPI_ISL_2360655, EPI_ISL_2360656, EPI_ISL_2360657, EPI_ISL_2360658, EPI_ISL_2360659, EPI_ISL_2360660, EPI_ISL_2360661, EPI_ISL_2360662, EPI_ISL_2360663, EPI_ISL_2360664, EPI_ISL_2360665, EPI_ISL_2360666, EPI_ISL_2360667, EPI_ISL_2360668, EPI_ISL_2360669, EPI_ISL_2360670, EPI_ISL_2360671, EPI_ISL_2360672, EPI_ISL_2360673, EPI_ISL_2360674, EPI_ISL_2360675, EPI_ISL_2360676, EPI_ISL_2360677, EPI_ISL_2360678, EPI_ISL_2360679, EPI_ISL_2360680, EPI_ISL_2360681, EPI_ISL_2360682, EPI_ISL_2360683, EPI_ISL_2360684, EPI_ISL_2360685, EPI_ISL_2360686, EPI_ISL_2360687, EPI_ISL_2360688, EPI_ISL_2360689, EPI_ISL_2360690, EPI_ISL_2360691, EPI_ISL_2360692, EPI_ISL_2360693, EPI_ISL_2360694, EPI_ISL_2360695, EPI_ISL_2360696, EPI_ISL_2360697, EPI_ISL_2360698, EPI_ISL_2360699, EPI_ISL_2360700, EPI_ISL_2360701, EPI_ISL_2360702, EPI_ISL_2360703, EPI_ISL_2360704, EPI_ISL_2360705, EPI_ISL_2360706, EPI_ISL_2360707, EPI_ISL_2360708, EPI_ISL_2360709, EPI_ISL_2360710, EPI_ISL_2360711, EPI_ISL_2360712, EPI_ISL_2360713, EPI_ISL_2360714, EPI_ISL_2360715, EPI_ISL_2360716, EPI_ISL_2360717, EPI_ISL_2360718, EPI_ISL_2360719, EPI_ISL_2360720, EPI_ISL_2360721, EPI_ISL_2360722, EPI_ISL_2360723, EPI_ISL_2360724, EPI_ISL_2360725, EPI_ISL_2360726, EPI_ISL_2360727, EPI_ISL_2360728, EPI_ISL_2360729, EPI_ISL_2360730, EPI_ISL_2360731, EPI_ISL_2360732, EPI_ISL_2360733, EPI_ISL_2360734, EPI_ISL_2360735, EPI_ISL_2360736, EPI_ISL_2360737, EPI_ISL_2360738, EPI_ISL_2360739, EPI_ISL_2360740, EPI_ISL_2360741, EPI_ISL_2360742, EPI_ISL_2360743, EPI_ISL_2360744, EPI_ISL_2360745, EPI_ISL_2360746, EPI_ISL_2360747, EPI_ISL_2360748, EPI_ISL_2360749, EPI_ISL_2360750, EPI_ISL_2360751, EPI_ISL_2360752, EPI_ISL_2360753, EPI_ISL_2360754, EPI_ISL_2360755, EPI_ISL_2360756, EPI_ISL_2360757, EPI_ISL_2360758, EPI_ISL_2360759, EPI_ISL_2360760, EPI_ISL_2360761, EPI_ISL_2360762, EPI_ISL_2360763, EPI_ISL_2360764, EPI_ISL_2360765, EPI_ISL_2360766, EPI_ISL_2360767, EPI_ISL_2360768, EPI_ISL_2360769, EPI_ISL_2360770, EPI_ISL_2360771, EPI_ISL_2360772, EPI_ISL_2360773, EPI_ISL_2360774, EPI_ISL_2360775, EPI_ISL_2360776, EPI_ISL_2360777, EPI_ISL_2360778, EPI_ISL_2360779, EPI_ISL_2360780, EPI_ISL_2360781, EPI_ISL_2360782, EPI_ISL_2360783, EPI_ISL_2360784, EPI_ISL_2360785, EPI_ISL_2360786, EPI_ISL_2360787, EPI_ISL_2360788, EPI_ISL_2360789, EPI_ISL_2360790, EPI_ISL_2360791, EPI_ISL_2360792, EPI_ISL_2360793, EPI_ISL_2360794, EPI_ISL_2360795, EPI_ISL_2360796, EPI_ISL_2360797, EPI_ISL_2360798, EPI_ISL_2360799, EPI_ISL_2360800, EPI_ISL_2360801, EPI_ISL_2360802, EPI_ISL_2360803, EPI_ISL_2360804, EPI_ISL_2360805, EPI_ISL_2360806, EPI_ISL_2360807, EPI_ISL_2360808, EPI_ISL_2360809, EPI_ISL_2360810, EPI_ISL_2360811, EPI_ISL_2360812, EPI_ISL_2360813, EPI_ISL_2360814, EPI_ISL_2360815, EPI_ISL_2360816, EPI_ISL_2360817, EPI_ISL_2360818, EPI_ISL_2360819, EPI_ISL_2360820, EPI_ISL_2360821, EPI_ISL_2360822, EPI_ISL_2360823, EPI_ISL_2360824, EPI_ISL_2360825, EPI_ISL_2360826, EPI_ISL_2360827, EPI_ISL_2360828, EPI_ISL_2360829, EPI_ISL_2360830, EPI_ISL_2360831, EPI_ISL_2360832, EPI_ISL_2360833, EPI_ISL_2360834, EPI_ISL_2360835, EPI_ISL_2360836, EPI_ISL_2360837, EPI_ISL_2360838, EPI_ISL_2360839, EPI_ISL_2360840, EPI_ISL_2360841, EPI_ISL_2360842, EPI_ISL_2360843, EPI_ISL_2360844, EPI_ISL_2360845, EPI_ISL_2360846, EPI_ISL_2360847, EPI_ISL_2360848, EPI_ISL_2360849, EPI_ISL_2360850, EPI_ISL_2360851, EPI_ISL_2360852, EPI_ISL_2360853, EPI_ISL_2360854, EPI_ISL_2360855, EPI_ISL_2360856, EPI_ISL_2360857, EPI_ISL_2360858, EPI_ISL_2360859, EPI_ISL_2360860, EPI_ISL_2360861, EPI_ISL_2360862, EPI_ISL_2360863, EPI_ISL_2360864, EPI_ISL_2360865, EPI_ISL_2360866, EPI_ISL_2360867, EPI_ISL_2360868, EPI_ISL_2360869, EPI_ISL_2360870, EPI_ISL_2360871, EPI_ISL_2360872, EPI_ISL_2360873, EPI_ISL_2360874, EPI_ISL_2360875, EPI_ISL_2360876, EPI_ISL_2360877, EPI_ISL_2360878, EPI_ISL_2360879, EPI_ISL_2360880, EPI_ISL_2360881, EPI_ISL_2360882, EPI_ISL_2360883, EPI_ISL_2360884, EPI_ISL_2360885, EPI_ISL_2360886, EPI_ISL_2360887, EPI_ISL_2360888, EPI_ISL_2360889, EPI_ISL_2360890, EPI_ISL_2360891, EPI_ISL_2360892, EPI_ISL_2360893, EPI_ISL_2360894, EPI_ISL_2360895, EPI_ISL_2360896, EPI_ISL_2360897, EPI_ISL_2360898, EPI_ISL_2360899, EPI_ISL_2360900, EPI_ISL_2360901, EPI_ISL_2360902, EPI_ISL_2360903, EPI_ISL_2360904, EPI_ISL_2360905, EPI_ISL_2360906, EPI_ISL_2360907, EPI_ISL_2360908, EPI_ISL_2360909, EPI_ISL_2360910, EPI_ISL_2360911, EPI_ISL_2360912, EPI_ISL_2360913, EPI_ISL_2360914, EPI_ISL_2360915, EPI_ISL_2360916, EPI_ISL_2360917, EPI_ISL_2360918, EPI_ISL_2360919, EPI_ISL_2360920, EPI_ISL_2360921, EPI_ISL_2360922, EPI_ISL_2360923, EPI_ISL_2360924, EPI_ISL_2360925, EPI_ISL_2360926, EPI_ISL_2360927, EPI_ISL_2360928, EPI_ISL_2360929, EPI_ISL_2360930, EPI_ISL_2360931, EPI_ISL_2360932, EPI_ISL_2360933, EPI_ISL_2360934, EPI_ISL_2360935, EPI_ISL_2360936, EPI_ISL_2360937, EPI_ISL_2360938, EPI_ISL_2360939, EPI_ISL_2360940, EPI_ISL_2360941, EPI_ISL_2360942, EPI_ISL_2360943, EPI_ISL_2360944, EPI_ISL_2360945, EPI_ISL_2360946, EPI_ISL_2360947, EPI_ISL_2360948, EPI_ISL_2360949, EPI_ISL_2360950, EPI_ISL_2360951, EPI_ISL_2360952, EPI_ISL_2360953, EPI_ISL_2360954, EPI_ISL_2360955, EPI_ISL_2360956, EPI_ISL_2360957, EPI_ISL_2360958, EPI_ISL_2360959, EPI_ISL_2360960, EPI_ISL_2360961, EPI_ISL_2360962, EPI_ISL_2360963, EPI_ISL_2360964, EPI_ISL_2360965, EPI_ISL_2360966, EPI_ISL_2360967, EPI_ISL_2360968, EPI_ISL_2360969, EPI_ISL_2360970, EPI_ISL_2360971, EPI_ISL_2360972, EPI_ISL_2360973, EPI_ISL_2360974, EPI_ISL_2360975, EPI_ISL_2360976, EPI_ISL_2360977, EPI_ISL_2360978, EPI_ISL_2360979, EPI_ISL_2360980, EPI_ISL_2360981, EPI_ISL_2360982, EPI_ISL_2360983, EPI_ISL_2360984, EPI_ISL_2360985, EPI_ISL_2360986, EPI_ISL_2360987, EPI_ISL_2360988, EPI_ISL_2360989, EPI_ISL_2360990, EPI_ISL_2360991, EPI_ISL_2360992, EPI_ISL_2360993, EPI_ISL_2360994, EPI_ISL_2360995, EPI_ISL_2360996, EPI_ISL_2360997, EPI_ISL_2360998, EPI_ISL_2360999, EPI_ISL_2361000, EPI_ISL_2361001, EPI_ISL_2361002, EPI_ISL_2361003, EPI_ISL_2361004, EPI_ISL_2361005, EPI_ISL_2361006, EPI_ISL_2361007, EPI_ISL_2361008, EPI_ISL_2361009, EPI_ISL_2361010, EPI_ISL_2361011, EPI_ISL_2361012, EPI_ISL_2361013, EPI_ISL_2361014, EPI_ISL_2361015, EPI_ISL_2361016, EPI_ISL_2361017, EPI_ISL_2361018, EPI_ISL_2361019, EPI_ISL_2361020, EPI_ISL_2361021, EPI_ISL_2361022, EPI_ISL_2361023, EPI_ISL_2361024, EPI_ISL_2361025, EPI_ISL_2361026, EPI_ISL_2361027, EPI_ISL_2361028, EPI_ISL_2361029, EPI_ISL_2361030, EPI_ISL_2361031, EPI_ISL_2361032, EPI_ISL_2361033, EPI_ISL_2361034, EPI_ISL_2361035, EPI_ISL_2361036, EPI_ISL_2361037, EPI_ISL_2361038, EPI_ISL_2361039, EPI_ISL_2361040, EPI_ISL_2361041, EPI_ISL_2361042, EPI_ISL_2361043, EPI_ISL_2361044, EPI_ISL_2361045, EPI_ISL_2361046, EPI_ISL_2361047, EPI_ISL_2361048, EPI_ISL_2361049, EPI_ISL_2361050, EPI_ISL_2361051, EPI_ISL_2361052, EPI_ISL_2361053, EPI_ISL_2361054, EPI_ISL_2361055, EPI_ISL_2361056, EPI_ISL_2361057, EPI_ISL_2361058, EPI_ISL_2361059, EPI_ISL_2361060, EPI_ISL_2361061, EPI_ISL_2361062, EPI_ISL_2361063, EPI_ISL_2361064, EPI_ISL_2361065, EPI_ISL_2361066, EPI_ISL_2361067, EPI_ISL_2361068, EPI_ISL_2361069, EPI_ISL_2361070, EPI_ISL_2361071, EPI_ISL_2361072, EPI_ISL_2361073, EPI_ISL_2361074, EPI_ISL_2361075, EPI_ISL_2361076, EPI_ISL_2361077, EPI_ISL_2361078, EPI_ISL_2361079, EPI_ISL_2361080, EPI_ISL_2361081, EPI_ISL_2361082, EPI_ISL_2361083, EPI_ISL_2361084, EPI_ISL_2361085, EPI_ISL_2361086, EPI_ISL_2361087, EPI_ISL_2361088, EPI_ISL_2494354, EPI_ISL_2494355, EPI_ISL_2494356, EPI_ISL_2494357, EPI_ISL_2494358, EPI_ISL_2494359, EPI_ISL_2494360, EPI_ISL_2494361, EPI_ISL_2494362, EPI_ISL_2494364, EPI_ISL_2494365, EPI_ISL_2494366, EPI_ISL_2494367, EPI_ISL_2494369, EPI_ISL_2494370, EPI_ISL_2494371, EPI_ISL_2494372, EPI_ISL_2494373, EPI_ISL_2494374, EPI_ISL_2494375, EPI_ISL_2494377, EPI_ISL_2494378, EPI_ISL_2494379, EPI_ISL_2494380, EPI_ISL_2494381, EPI_ISL_2494382, EPI_ISL_2494383, EPI_ISL_2494384, EPI_ISL_2494385, EPI_ISL_2494386, EPI_ISL_2494388, EPI_ISL_2494389, EPI_ISL_2494391, EPI_ISL_2494392, EPI_ISL_2494393, EPI_ISL_2494394, EPI_ISL_2494395, EPI_ISL_2494396, EPI_ISL_2494398, EPI_ISL_2494399, EPI_ISL_2494400, EPI_ISL_2494401, EPI_ISL_2494402, EPI_ISL_2494404, EPI_ISL_2494405, EPI_ISL_2494406, EPI_ISL_2494407, EPI_ISL_2494408, EPI_ISL_2494409, EPI_ISL_2494410, EPI_ISL_2494411, EPI_ISL_2494412, EPI_ISL_2494413, EPI_ISL_2494414, EPI_ISL_2494415, EPI_ISL_2494416, EPI_ISL_2494418, EPI_ISL_2494419, EPI_ISL_2494420, EPI_ISL_2494423, EPI_ISL_2494425, EPI_ISL_2494426, EPI_ISL_2494429, EPI_ISL_2494430, EPI_ISL_2494432, EPI_ISL_2494433, EPI_ISL_2494434, EPI_ISL_2494435, EPI_ISL_2494436, EPI_ISL_2494438, EPI_ISL_2494439, EPI_ISL_2494442, EPI_ISL_2494443, EPI_ISL_2494444, EPI_ISL_2494448, EPI_ISL_2494452, EPI_ISL_2494456, EPI_ISL_2494460, EPI_ISL_2494461, EPI_ISL_2494466, EPI_ISL_2494467, EPI_ISL_2494468, EPI_ISL_2494471, EPI_ISL_2494472, EPI_ISL_2494473, EPI_ISL_2494477, EPI_ISL_2494478, EPI_ISL_2494479, EPI_ISL_2494481, EPI_ISL_2494482, EPI_ISL_2494483, EPI_ISL_2494484, EPI_ISL_2494485, EPI_ISL_2494486, EPI_ISL_2494487, EPI_ISL_2494488, EPI_ISL_2494489, EPI_ISL_2494494, EPI_ISL_2494495, EPI_ISL_2494496, EPI_ISL_2494497, EPI_ISL_2494498, EPI_ISL_2494501, EPI_ISL_2494512, EPI_ISL_2494513, EPI_ISL_2494518, EPI_ISL_2494519, EPI_ISL_2494520, EPI_ISL_2494521, EPI_ISL_2494522, EPI_ISL_2494524, EPI_ISL_2494526, EPI_ISL_2494527, EPI_ISL_2494528, EPI_ISL_2494529, EPI_ISL_2494530, EPI_ISL_2494533, EPI_ISL_2494534, EPI_ISL_2494535, EPI_ISL_2494537, EPI_ISL_2494538, EPI_ISL_2494539, EPI_ISL_2494540, EPI_ISL_2494543, EPI_ISL_2494544, EPI_ISL_2494545, EPI_ISL_2494546, EPI_ISL_2494547, EPI_ISL_2494548, EPI_ISL_2494549, EPI_ISL_2494550, EPI_ISL_2494551, EPI_ISL_2494552, EPI_ISL_2494553, EPI_ISL_2494554, EPI_ISL_2494557, EPI_ISL_2494558, EPI_ISL_2494559, EPI_ISL_2494560, EPI_ISL_2494561, EPI_ISL_2494562, EPI_ISL_2494563, EPI_ISL_2494564, EPI_ISL_2494565, EPI_ISL_2494566, EPI_ISL_2494567, EPI_ISL_2494568, EPI_ISL_2494570, EPI_ISL_2494571, EPI_ISL_2494572, EPI_ISL_2494573, EPI_ISL_2494574, EPI_ISL_2494575, EPI_ISL_2494576, EPI_ISL_2494577, EPI_ISL_2494578, EPI_ISL_2494580, EPI_ISL_2494581, EPI_ISL_2494582, EPI_ISL_2494583, EPI_ISL_2494584, EPI_ISL_2494585, EPI_ISL_2494586, EPI_ISL_2494587, EPI_ISL_2494588, EPI_ISL_2494589, EPI_ISL_2494590, EPI_ISL_2494591, EPI_ISL_2494592, EPI_ISL_2494593, EPI_ISL_2494594, EPI_ISL_2494595, EPI_ISL_2494596, EPI_ISL_2494597, EPI_ISL_2494598, EPI_ISL_2494599, EPI_ISL_2494600, EPI_ISL_2494601, EPI_ISL_2494602, EPI_ISL_2494603, EPI_ISL_2494604, EPI_ISL_2494605, EPI_ISL_2494609, EPI_ISL_2494610, EPI_ISL_2494614, EPI_ISL_2494616, EPI_ISL_2494627, EPI_ISL_2494628, EPI_ISL_2494629, EPI_ISL_2494630, EPI_ISL_2494631			
see above	Vaccines and Infectious Diseases Analytics Research Unit (VIDA)	KRISP, KZn Research Innovation and Sequencing Platform	Baillie Vicky; Giandhari Jennifer; Madhi Shabir; Naidoo Yeshnee; Pillay Sureshnee; San James; Tegally Houriiyah; Tshabuila Derek; Wilkinson Eduan; de Oliveira Tulio; du Plessis Jeanine
EPI_ISL_1534381	Valkenberg Hospital wc VBH	NHLS/UCT	Arash Iranzadeh; Bruna Galvao; Carolyn Williamson; Deelan Doolabh; Diana Hardie; Emmanuel SJ; Innocent Mudau; Kruger Marais; Lynn Tyers; Marvin Hsiao; Stephen Korsman; Tegally H; de Oliveira T
EPI_ISL_1534361	Van Wyksdorp Sat Clinic wc VWS	NHLS/UCT	Arash Iranzadeh; Bruna Galvao; Carolyn Williamson; Deelan Doolabh; Diana Hardie; Emmanuel SJ; Innocent Mudau; Kruger Marais; Lynn Tyers; Marvin Hsiao; Stephen Korsman; Tegally H; de Oliveira T
EPI_ISL_1534354, EPI_ISL_1534409	Vanguard CHC wc VGC	NHLS/UCT	Arash Iranzadeh; Bruna Galvao; Carolyn Williamson; Deelan Doolabh; Diana Hardie; Emmanuel SJ; Innocent Mudau; Kruger Marais; Lynn Tyers; Marvin Hsiao; Stephen Korsman; Tegally H; de Oliveira T
EPI_ISL_2582723, EPI_ISL_2582726, EPI_ISL_2582730, EPI_ISL_2582733, EPI_ISL_2582736, EPI_ISL_2582739, EPI_ISL_2582742, EPI_ISL_2582744, EPI_ISL_2582746, EPI_ISL_2582748, EPI_ISL_2582749, EPI_ISL_2582752, EPI_ISL_2582754, EPI_ISL_2582757, EPI_ISL_2582761, EPI_ISL_2582765, EPI_ISL_2582769, EPI_ISL_2582772, EPI_ISL_2582774, EPI_ISL_2582776, EPI_ISL_2582781, EPI_ISL_2582786, EPI_ISL_2582790, EPI_ISL_2582792, EPI_ISL_2582794, EPI_ISL_2582797, EPI_ISL_2582800, EPI_ISL_2582803, EPI_ISL_2582806			
see above	Vermaak & Partners Laboratory	National Institute for Communicable Diseases of the National Health Laboratory Service	Amoako DG; Bhiman JN; Everatt J; Ismail A; Mahlangu B; Mnguni A; Mohale T; Ntuli N; Scheepers C
EPI_ISL_1534261, EPI_ISL_1534263, EPI_ISL_1534265, EPI_ISL_1534295, EPI_ISL_1534300, EPI_ISL_1534305, EPI_ISL_1534337, EPI_ISL_1534353, EPI_ISL_1534386, EPI_ISL_1534420, EPI_ISL_1534430, EPI_ISL_1817674, EPI_ISL_1817690, EPI_ISL_1817692, EPI_ISL_2140668, EPI_ISL_2375981, EPI_ISL_2621101, EPI_ISL_2621104, EPI_ISL_2802143, EPI_ISL_2802144, EPI_ISL_2802145, EPI_ISL_2802146, EPI_ISL_2802162, EPI_ISL_2802163, EPI_ISL_2802168			
see above	Victoria Hospital wc VHW	NHLS/UCT	Arash Iranzadeh; Bruna Galvao; Carolyn Williamson; Deelan Doolabh; Diana Hardie; Emmanuel SJ; Gert Marais; Innocent Mudau; Kruger Marais; Lynn Tyers; Marvin Hsiao; Stephen Korsman; Tegally H; de Oliveira T
EPI_ISL_1623756, EPI_ISL_1623757, EPI_ISL_1623758, EPI_ISL_1785562, EPI_ISL_1785563, EPI_ISL_1785564, EPI_ISL_1785565, EPI_ISL_1785574, EPI_ISL_2982479, EPI_ISL_2982480, EPI_ISL_2982481, EPI_ISL_2982482, EPI_ISL_2982483, EPI_ISL_2982484, EPI_ISL_2982485, EPI_ISL_2982486, EPI_ISL_2982487, EPI_ISL_2982488, EPI_ISL_2982489, EPI_ISL_2982490, EPI_ISL_2982491, EPI_ISL_2982492, EPI_ISL_2982493, EPI_ISL_2982494, EPI_ISL_2982495, EPI_ISL_2982496, EPI_ISL_2982497, EPI_ISL_2982498, EPI_ISL_2982499, EPI_ISL_2982500, EPI_ISL_2982501, EPI_ISL_2982502, EPI_ISL_2982503, EPI_ISL_2982504, EPI_ISL_2982505, EPI_ISL_2982506, EPI_ISL_2982507, EPI_ISL_2982508, EPI_ISL_2982509, EPI_ISL_2982510, EPI_ISL_2982511, EPI_ISL_2982512, EPI_ISL_2982513, EPI_ISL_2982514, EPI_ISL_2982515, EPI_ISL_2982516, EPI_ISL_2982517, EPI_ISL_2982518, EPI_ISL_2982519, EPI_ISL_2982520, EPI_ISL_2982521, EPI_ISL_2982522, EPI_ISL_2982523, EPI_ISL_2982524, EPI_ISL_2982525, EPI_ISL_2982526, EPI_ISL_2982527, EPI_ISL_2982528, EPI_ISL_2982529, EPI_ISL_2982530, EPI_ISL_2982531, EPI_ISL_2982532, EPI_ISL_2982533, EPI_ISL_2982534, EPI_ISL_2982535, EPI_ISL_2982536, EPI_ISL_2982537, EPI_ISL_2982538, EPI_ISL_2982539, EPI_ISL_2982540, EPI_ISL_2982541, EPI_ISL_2982542, EPI_ISL_2982543, EPI_ISL_2982544, EPI_ISL_2982545, EPI_ISL_2982546, EPI_ISL_2982547, EPI_ISL_2982548, EPI_ISL_2982549, EPI_ISL_2982550, EPI_ISL_2982551, EPI_ISL_2982552, EPI_ISL_2982553, EPI_ISL_2982554, EPI_ISL_2982555, EPI_ISL_2982556, EPI_ISL_2982557, EPI_ISL_2982558, EPI_ISL_2982559, EPI_ISL_2982560, EPI_ISL_2982561, EPI_ISL_2982562, EPI_ISL_2982563, EPI_ISL_2982564, EPI_ISL_2982565, EPI_ISL_2982566, EPI_ISL_2982567, EPI_ISL_2982568, EPI_ISL_2982569, EPI_ISL_2982570, EPI_ISL_2982571, EPI_ISL_2982572, EPI_ISL_2982573, EPI_ISL_2982574, EPI_ISL_2982575, EPI_ISL_2982576, EPI_ISL_2982577, EPI_ISL_2982578, EPI_ISL_2982579, EPI_ISL_2982580, EPI_ISL_2982581, EPI_ISL_2982582, EPI_ISL_2982583, EPI_ISL_2982584, EPI_ISL_2982585, EPI_ISL_2982586, EPI_ISL_2982587, EPI_ISL_2982588, EPI_ISL_2982589, EPI_ISL_2982590, EPI_ISL_2982591, EPI_ISL_2982592, EPI_ISL_2982593, EPI_ISL_2982594, EPI_ISL_2982595, EPI_ISL_2982596, EPI_ISL_2982597, EPI_ISL_2982598, EPI_ISL_2982599, EPI_ISL_2982600, EPI_ISL_2982601, EPI_ISL_2982602, EPI_ISL_2982603, EPI_ISL_2982604, EPI_ISL_2982605, EPI_ISL_2982606, EPI_ISL_2982607, EPI_ISL_2982608, EPI_ISL_2982609, EPI_ISL_2982610, EPI_ISL_2982611, EPI_ISL_2982612, EPI_ISL_2982613, EPI_ISL_2982614, EPI_ISL_2982615, EPI_ISL_2982616, EPI_ISL_2982617, EPI_ISL_2982618, EPI_ISL_2982619, EPI_ISL_2982620, EPI_ISL_2982621, EPI_ISL_2982622, EPI_ISL_2982623, EPI_ISL_2982624, EPI_ISL_2982625, EPI_ISL_2982626, EPI_ISL_2982627, EPI_ISL_3133634, EPI_ISL_3133635, EPI_ISL_3133636, EPI_ISL_3133637, EPI_ISL_3133638, EPI_ISL_3133639, EPI_ISL_3133640, EPI_ISL_3133641, EPI_ISL_3133642, EPI_ISL_3133645, EPI_ISL_3133654, EPI_ISL_3133655, EPI_ISL_3133656, EPI_ISL_3133657, EPI_ISL_3133658, EPI_ISL_3133659, EPI_ISL_3133660, EPI_ISL_3133661, EPI_ISL_3133662, EPI_ISL_3133663			
see above	Viral Respiratory Lab, National Institute for Biomedical Research (INRB)	Pathogen Sequencing Lab, National Institute for Biomedical Research (INRB)	Allison Black; Amuri Aziza; Andrew Rambaut; Catherine Pratt; Eddy Kinganda-Lusamaki; Edith Nkwembe; Emmanuel Lokilo Lofiko; Francisca Muyembe Mawete; Gabriel Kabamba; Ian Goodfellow; James Hadfield; Jean Claude Makangara; Jean-Jacques Muyembe Tamfum; Josh Quick; Kristian Andersen; Matthias Pauthner; Michael Wiley; Nick Loman; Placide Mbala-Kingebeni; Raphaël Lumembe; Steve Ahuka-Mundeke; Trevor Bedford
EPI_ISL_2499902, EPI_ISL_2499903, EPI_ISL_2499904, EPI_ISL_2499905, EPI_ISL_2499906, EPI_ISL_2499907, EPI_ISL_2499908, EPI_ISL_2499909, EPI_ISL_2499910, EPI_ISL_2499911, EPI_ISL_2499912, EPI_ISL_2499913, EPI_ISL_2499914, EPI_ISL_2499915, EPI_ISL_2499916, EPI_ISL_2499917, EPI_ISL_2532755			
see above	Virology Department, Central Health Laboratory	The Francis Crick Institute	Bahadoor BS; Crawford M; Daniels RS; Goldstone R; Harvey R; Manraj SS; Nicod J; Patel H; Ramuth M; Sonoo J
EPI_ISL_1711641, EPI_ISL_1711642, EPI_ISL_1711643, EPI_ISL_1711644, EPI_ISL_1711645, EPI_ISL_1711646, EPI_ISL_1711659, EPI_ISL_1711660, EPI_ISL_1711661, EPI_ISL_1711662, EPI_ISL_1711663, EPI_ISL_1711664, EPI_ISL_1711665, EPI_ISL_1711666, EPI_ISL_1711667, EPI_ISL_1711668			
see above	Virology Laboratory of Praia	Institut Pasteur de Dakar	Dia Ndongo; Diagne Moussa Moïse; Diallo Amadou; Diop Mamadou; Faye Ousmane; Loucoubar Cheikh; Ndiaye Ndack; Sall Amadou Alpha; Sanders Kevin; Sankhe Safietou; da Luz Lima Mendonça Maria
EPI_ISL_1711647, EPI_ISL_1711648, EPI_ISL_1711649, EPI_ISL_1711650, EPI_ISL_1711651, EPI_ISL_1711652, EPI_ISL_1711653, EPI_ISL_1711654, EPI_ISL_1711655, EPI_ISL_1711656, EPI_ISL_1711657, EPI_ISL_1711658			
see above	Virology Laboratory of São Vicente	Institut Pasteur de Dakar	Dia Ndongo; Diagne Moussa Moïse; Diallo Amadou; Diop Mamadou; Faye Ousmane; Loucoubar Cheikh; Nadia Rodrigues; Ndiaye Ndack; Sall Amadou Alpha; Sanders Kevin; Sankhe Safietou; da Luz Lima Mendonça Maria
EPI_ISL_1660225, EPI_ISL_1660226, EPI_ISL_1660227, EPI_ISL_1660228, EPI_ISL_1660229, EPI_ISL_1660230, EPI_ISL_1660231, EPI_ISL_1660232, EPI_ISL_1660233, EPI_ISL_1660234, EPI_ISL_1660235, EPI_ISL_1660236, EPI_ISL_1660237, EPI_ISL_1660238, EPI_ISL_1660239, EPI_ISL_1660240, EPI_ISL_1660241, EPI_ISL_1660242, EPI_ISL_1660243, EPI_ISL_1660244, EPI_ISL_1660245, EPI_ISL_1660246, EPI_ISL_1660247, EPI_ISL_1660248, EPI_ISL_1660249, EPI_ISL_1660250, EPI_ISL_1660251, EPI_ISL_1660252, EPI_ISL_1660253, EPI_ISL_1660254, EPI_ISL_1660255, EPI_ISL_1660256, EPI_ISL_1660257, EPI_ISL_1660258, EPI_ISL_1660259, EPI_ISL_1660260, EPI_ISL_1660261, EPI_ISL_1660262, EPI_ISL_1660263, EPI_ISL_1660264, EPI_ISL_1660265, EPI_ISL_1660266, EPI_ISL_1660267, EPI_ISL_1660268, EPI_ISL_1660269, EPI_ISL_1660270, EPI_ISL_1660271, EPI_ISL_1660272, EPI_ISL_1660273, EPI_ISL_1660274, EPI_ISL_1660275, EPI_ISL_1660276, EPI_ISL_1660277, EPI_ISL_1660278, EPI_ISL_1660279, EPI_ISL_1660280, EPI_ISL_1660281, EPI_ISL_1660282, EPI_ISL_1660283, EPI_ISL_1660284, EPI_ISL_1660285, EPI_ISL_1660286, EPI_ISL_1660287, EPI_ISL_1660291, EPI_ISL_1660292, EPI_ISL_1660293, EPI_ISL_1660294, EPI_ISL_1660295, EPI_ISL_1660296, EPI_ISL_1660297, EPI_ISL_1660298, EPI_ISL_1660299, EPI_ISL_1660300, EPI_ISL_1660301, EPI_ISL_1660302, EPI_ISL_1660303, EPI_ISL_1660304, EPI_ISL_1660305, EPI_ISL_1660306, EPI_ISL_1660307, EPI_ISL_1660308, EPI_ISL_1660309, EPI_ISL_1660310, EPI_ISL_1660311, EPI_ISL_1660312, EPI_ISL_1660313, EPI_ISL_1660314, EPI_ISL_1660315, EPI_ISL_1660316, EPI_ISL_1660317, EPI_ISL_1660318, EPI_ISL_1660319, EPI_ISL_1660320, EPI_ISL_1660321, EPI_ISL_1660322, EPI_ISL_1660323, EPI_ISL_1660324, EPI_ISL_1660325, EPI_ISL_1660326, EPI_ISL_1660327, EPI_ISL_1660328, EPI_ISL_1660329, EPI_ISL_1660330, EPI_ISL_1660331, EPI_ISL_1660332, EPI_ISL_1660333, EPI_ISL_1660334, EPI_ISL_1660335, EPI_ISL_1660336, EPI_ISL_1672387, EPI_ISL_1672388, EPI_ISL_1672389, EPI_ISL_1672390			
see above	Virology Unit, Institut Pasteur de Madagascar	Virology Unit, Institut Pasteur de Madagascar	Angela Brisebarre; Camille Capel; Cara E. Brook; Christian Ranaivoson; Christophe Malabat; Corinne Maufrais; Cristina M. Tato; Emmanuelle Permal; Etienne Simon-Lorière; Frédéric Lemoine; Helisoa Razafimanjato; Jean-Michel Heraud; Joseph L. DeRisi; Louise Lefrançois; Marion Barbet; Maud Vanpeene; Michelle Tan; Méline Bizard; Norosoa Razanajatovo; Philippe Dussart; Soa Fy Andriamandimby; Sylvie Behillil; Sylvie van der Werf; Tsiry Randriambolamanantsoa; Vida Ahyong; Vincent Enouf; Vololoniaina Raharinosy
EPI_ISL_3115358, EPI_ISL_3115359, EPI_ISL_3115360	Virology laboratory, University of Maiduguri Teaching Hospital	Africa Centre for Excellence for Genomics of Infectious Diseases (ACEGID), Redeemer's University	A.T.; Abechi; Ajogbasile; Akano; C.A.; C.T.; Eromon; F.V.; Folarin, O.; Happi; I.B.; J.N.; J.U.; K.O.; Kayode; Nosamiefan, I.; Oguzie; Olawoye; Olumade; Oluniyi; P.E.; P.S.; T.J.; Ugwu; Uwanibe
EPI_ISL_2836849, EPI_ISL_2836850, EPI_ISL_2836851, EPI_ISL_2836852, EPI_ISL_2836854, EPI_ISL_2836855, EPI_ISL_2836856, EPI_ISL_2836857, EPI_ISL_2836859, EPI_ISL_2836860, EPI_ISL_2836863, EPI_ISL_2836865, EPI_ISL_2836866, EPI_ISL_2836867, EPI_ISL_2836869, EPI_ISL_2836870, EPI_ISL_2836873, EPI_ISL_2836874, EPI_ISL_2836876, EPI_ISL_2836877, EPI_ISL_2836878, EPI_ISL_2836884, EPI_ISL_2836892, EPI_ISL_2836893, EPI_ISL_2836896, EPI_ISL_2836899, EPI_ISL_2968022			
see above	WACCBIP, University of Ghana, Accra, Ghana	WACCBIP, University of Ghana, Volta Road, Legon-Accra, Ghana	Bright K. Yemi; Collins M. Morang'a; Deborah N. A. Mettle; Dominic S. Y. Amuzu; Dominic S.Y. Amuzu; Evelyn B. Quansah; Evelyn Y. Bonney; Frederick Tei-Maya; Israel Osei-Wusu; Ivy A. Asante; Joe K. Mutungi; John K. Odoom; Joseph H.K. Bonney; Joyce M. Ngoi; Lawrence Ofori-Boadu; Lucas N. Amenga-Etego and Gordon A. Awandare; Mildred Adusei-Poku; Nicaise T. Ndam; Oliver Commey; Peter K Quashie; Philip M. Soglo; Samirah Saiid; Samuel Armoo; Vincent Appiah; Violette V. M'cormack; William K. Ampofo; Yaw Bediako
EPI_ISL_2422495, EPI_ISL_2422496, EPI_ISL_2422497, EPI_ISL_2422498, EPI_ISL_2422499, EPI_ISL_2422500, EPI_ISL_2422501, EPI_ISL_2422502, EPI_ISL_2422503, EPI_ISL_2422504, EPI_ISL_2422505, EPI_ISL_2422506, EPI_ISL_2422507, EPI_ISL_2422508, EPI_ISL_2422509, EPI_ISL_2422510, EPI_ISL_2422511, EPI_ISL_2422512, EPI_ISL_2422513, EPI_ISL_2422514, EPI_ISL_2422515, EPI_ISL_2422516, EPI_ISL_2422517, EPI_ISL_2422518, EPI_ISL_2422519, EPI_ISL_2422520, EPI_ISL_2422521, EPI_ISL_2422522, EPI_ISL_2422523, EPI_ISL_2422524, EPI_ISL_2422525, EPI_ISL_2422526, EPI_ISL_2422527, EPI_ISL_2422528, EPI_ISL_2422529, EPI_ISL_2422530, EPI_ISL_2422531, EPI_ISL_2422532, EPI_ISL_2422533, EPI_ISL_2422534, EPI_ISL_2422535, EPI_ISL_2422536, EPI_ISL_2422537, EPI_ISL_2422538, EPI_ISL_2422539, EPI_ISL_2422559, EPI_ISL_2422597, EPI_ISL_2422598, EPI_ISL_2422603, EPI_ISL_2422607, EPI_ISL_2422608, EPI_ISL_2422611, EPI_ISL_2422617, EPI_ISL_2422618, EPI_ISL_2422622, EPI_ISL_2422624, EPI_ISL_2422628, EPI_ISL_2422630			
see above	West African Centre for Cell Biology of Infectious Pathogen, University of Ghana, Legon	WACCBIP, University of Ghana, Volta Road, Legon, Accra	Collins M. Morang'a; Dominic S. Y. Amuzu; Dominic S.Y. Amuzu; Edward Danso Fenteng; Emmanuel Kudjo; Evelyn B. Quansah; Evelyn Y Bonney; Frederick Tei-Maya; Ivy A Asante; Joe K. Mutungi; John K. Odoom; Joseph HK Bonney; Joyce M. Ngoi; Lawrence Ofori-Boadu; Lucas N. Amenga-Etego and Gordon A Awandare; Lucas N. Amenga-Etego and Gordon A. Awandare; Mildred Adusei-Poku; Nicaise T. Ndam; Patrick Tetteh Ababio; Peter K Quashie; Peter K. Quashie; Philip M Soglo; Philip M. Soglo; Samirah Saiid; Theophilus Odoom; Vincent Appiah; Violet MCormack; Violette M'cormack; William K. Ampofo; Yaw Bediako
EPI_ISL_1255106, EPI_ISL_1255107, EPI_ISL_1255108, EPI_ISL_1255109, EPI_ISL_1255110, EPI_ISL_1255111, EPI_ISL_1255112, EPI_ISL_1255113, EPI_ISL_1255114, EPI_ISL_1255115, EPI_ISL_1255116, EPI_ISL_1255117, EPI_ISL_1255118, EPI_ISL_1255119, EPI_ISL_1255120, EPI_ISL_1255121, EPI_ISL_1255122, EPI_ISL_1255123, EPI_ISL_1255124, EPI_ISL_1255125, EPI_ISL_1255126, EPI_ISL_1255127, EPI_ISL_1255128, EPI_ISL_1255129, EPI_ISL_1255130, EPI_ISL_1255131, EPI_ISL_1255132, EPI_ISL_1255133, EPI_ISL_1255134, EPI_ISL_1255135, EPI_ISL_1255136, EPI_ISL_1255137, EPI_ISL_1255138, EPI_ISL_1255139, EPI_ISL_1255140, EPI_ISL_1255141, EPI_ISL_1255142, EPI_ISL_1255143, EPI_ISL_1255144, EPI_ISL_1255145, EPI_ISL_1255146, EPI_ISL_1255147, EPI_ISL_1255148, EPI_ISL_1255149, EPI_ISL_1255150, EPI_ISL_1255151, EPI_ISL_1255152, EPI_ISL_1255153, EPI_ISL_1255154, EPI_ISL_1255155, EPI_ISL_1255156, EPI_ISL_1255157, EPI_ISL_1255158, EPI_ISL_1255159, EPI_ISL_1255160, EPI_ISL_1255161, EPI_ISL_1255162, EPI_ISL_1255163, EPI_ISL_1255164, EPI_ISL_1255165, EPI_ISL_1255166, EPI_ISL_1255167, EPI_ISL_1255168, EPI_ISL_1255169, EPI_ISL_1255170, EPI_ISL_1255171,			

EPI_ISL_1255172, EPI_ISL_1255173, EPI_ISL_1255174, EPI_ISL_1255175, EPI_ISL_1255176, EPI_ISL_1255177, EPI_ISL_1255178, EPI_ISL_1255179, EPI_ISL_1255180, EPI_ISL_1255181, EPI_ISL_1255182, EPI_ISL_1255183, EPI_ISL_1255184, EPI_ISL_1255185, EPI_ISL_1255186, EPI_ISL_1255187, EPI_ISL_1255188, EPI_ISL_1255189, EPI_ISL_1255190, EPI_ISL_1255191, EPI_ISL_1255192, EPI_ISL_1255193, EPI_ISL_1255194, EPI_ISL_1255195, EPI_ISL_1255196, EPI_ISL_1255197, EPI_ISL_1255198, EPI_ISL_1255199, EPI_ISL_1255200, EPI_ISL_1255201, EPI_ISL_1255202, EPI_ISL_1255203, EPI_ISL_1255204, EPI_ISL_1255205, EPI_ISL_1255206, EPI_ISL_1255207, EPI_ISL_1255208, EPI_ISL_1255209, EPI_ISL_1255210, EPI_ISL_1255211, EPI_ISL_1255212, EPI_ISL_1255213, EPI_ISL_1255214, EPI_ISL_1255215, EPI_ISL_1255216, EPI_ISL_1255217, EPI_ISL_1255218, EPI_ISL_1255219, EPI_ISL_1255220, EPI_ISL_1255221, EPI_ISL_1255222, EPI_ISL_1255223, EPI_ISL_1255224, EPI_ISL_1255225, EPI_ISL_1255226, EPI_ISL_1255227, EPI_ISL_1255228, EPI_ISL_1255229, EPI_ISL_1255230, EPI_ISL_1255231, EPI_ISL_1255232, EPI_ISL_1255233, EPI_ISL_1255234, EPI_ISL_1255235, EPI_ISL_1255236, EPI_ISL_1255237, EPI_ISL_1255238, EPI_ISL_1255239, EPI_ISL_1255240, EPI_ISL_1255241, EPI_ISL_1255242, EPI_ISL_1255243, EPI_ISL_1255244, EPI_ISL_1255245, EPI_ISL_1255246, EPI_ISL_1255247, EPI_ISL_1255248, EPI_ISL_1255249, EPI_ISL_1255250, EPI_ISL_1255251, EPI_ISL_1255252, EPI_ISL_1255253, EPI_ISL_1255254, EPI_ISL_1255255, EPI_ISL_1255256, EPI_ISL_1255257, EPI_ISL_1255258, EPI_ISL_1255259, EPI_ISL_1255260, EPI_ISL_1255261, EPI_ISL_1255262, EPI_ISL_1255263, EPI_ISL_1255264, EPI_ISL_1255265, EPI_ISL_1255266, EPI_ISL_1255267, EPI_ISL_1255268, EPI_ISL_1255269, EPI_ISL_1255270, EPI_ISL_1255271, EPI_ISL_1255272, EPI_ISL_1255273, EPI_ISL_1255274, EPI_ISL_1255275, EPI_ISL_1255276, EPI_ISL_1255277			
see above	West African Centre for Cell Biology of Infectious Pathogens (WACCBIP), University of Ghana, Accra, Ghana	West African Centre for Cell Biology of Infectious Pathogens (WACCBIP), University of Ghana, Volta Road, Legon-Accra, Ghana	; Abdoulaye B Diallo; Abdul-Karim Abass; Aisha Mohammed; Benjamin Demah Nuertey; Collins M. Morang'a; Dam Kenneth Mibut; Dominic S.Y. Amuzu; Emmanuella Amoako4; Evelyn B. Quansah; Frederick Kumi-Ansah; Frederick Tei-Maya; Gordon A Awandare; Joyce M. Ngoi; Kesego Tapela; Lucas N. Amenga-Etego; Nelson Kibinge; Oliver D Boakye5; Peter K Quashie; Philip M. Soglo; Samirah Saiid; Samuel Kaba Akoriyea; Theophilus Odoom; Vanessa Magnussen; Vincent Appiah; Yaw Bediako
EPI_ISL_1255105	West African Centre for Cell Biology of Infectious Pathogens (WACCBIP), University of Ghana, Volta Road, Legon-Accra, Ghana	West African Centre for Cell Biology of Infectious Pathogens (WACCBIP), University of Ghana, Volta Road, Legon-Accra, Ghana	; Abdoulaye B Diallo; Abdul-Karim Abass; Aisha Mohammed; Benjamin Demah Nuertey; Collins M. Morang'a; Dam Kenneth Mibut; Dominic S.Y. Amuzu; Emmanuella Amoako4; Evelyn B. Quansah; Frederick Kumi-Ansah; Frederick Tei-Maya; Gordon A Awandare; Joyce M. Ngoi; Kesego Tapela; Lucas N. Amenga-Etego; Nelson Kibinge; Oliver D Boakye5; Peter K Quashie; Philip M. Soglo; Samirah Saiid; Samuel Kaba Akoriyea; Theophilus Odoom; Vanessa Magnussen; Vincent Appiah; Yaw Bediako
EPI_ISL_2727239, EPI_ISL_2727240, EPI_ISL_2727241, EPI_ISL_2727242, EPI_ISL_2727243, EPI_ISL_2727244, EPI_ISL_2727245, EPI_ISL_2727246, EPI_ISL_2727247, EPI_ISL_2727248, EPI_ISL_2727249, EPI_ISL_2727250, EPI_ISL_2727251, EPI_ISL_2727252, EPI_ISL_2727253, EPI_ISL_2727254, EPI_ISL_2727255, EPI_ISL_2727256, EPI_ISL_2727257, EPI_ISL_2727258, EPI_ISL_2727259, EPI_ISL_2727260, EPI_ISL_2727261, EPI_ISL_2727262, EPI_ISL_2727263, EPI_ISL_2727264, EPI_ISL_2727265, EPI_ISL_2727266, EPI_ISL_2727267, EPI_ISL_2727268, EPI_ISL_2727269, EPI_ISL_2727270, EPI_ISL_2727271, EPI_ISL_2727272, EPI_ISL_2727273, EPI_ISL_2727274, EPI_ISL_2727275, EPI_ISL_2727276, EPI_ISL_2727283, EPI_ISL_2727285, EPI_ISL_2727286, EPI_ISL_2727287, EPI_ISL_2727288, EPI_ISL_2727289, EPI_ISL_2727290			
see above	ZARV/NHLS, Department Medical Virology, University of Pretoria	KRISP, KZN Research Innovation and Sequencing Platform	Adriano Mendes; Amy Strydom; Emmanuel SJ; Giandhari J; Lessells R; Micheala Davids; Naidoo Y; Pillay S; Ramphal U; Sim Mayaphi and Marietjie Venter; Tegally H; Wilkinson E; de Oliveira T
EPI_ISL_2716621, EPI_ISL_2716622	aboratoire National de Référence pour les Fièvres Hémorragiques Virales, Centre Muraz	Centre Muraz	Ange Badjo; Arsène Zongo; Essia Belarbi; Fabian Leendertz; Grit Schubert; Jasmin Schlotterbeck; Saïdou Ouedraogo; Soumeya Ouangraoua; Thérèse Kagone

We gratefully acknowledge the following Authors from the Originating laboratories responsible for obtaining the specimens, as well as the Submitting laboratories where the genome data were generated and shared via GISAID, on which this research is based.

All Submitters of data may be contacted directly via www.gisaid.org

Authors are sorted alphabetically.

Accession ID	Originating Laboratory	Submitting Laboratory	Authors
EPI_ISL_5425707	Addis Ababa University	CERI, Centre for Epidemic Response and Innvoation, Stellenbosch University and KRISP, KZN Research Innovation and Sequencing Platform, UKZN.	Abay Sisay; Abraham Tesfaye; Adey Feleke Desta; Giandhari Jennifer; Naidoo Yeshnee; Pillay Sureshnee; San James; Tegally Houriiyah; Tshiabuila Derek; Wilkinson Eduan; Yajna Ramphal; de Oliveira Tulio
EPI_ISL_5510378, EPI_ISL_5510383, EPI_ISL_5510407, EPI_ISL_5510411	Baragwanath	National Institute for Communicable Diseases of the National Health Laboratory Service	Amoako DG; Bhiman JN; Everatt J; Ismail A; Mahlangu B; Mnguni A; Mohale T; Ntuli N; Scheepers C
EPI_ISL_4026291, EPI_ISL_4026292, EPI_ISL_4026293, EPI_ISL_4026294, EPI_ISL_4026295, EPI_ISL_4026297, EPI_ISL_4026299, EPI_ISL_4026300, EPI_ISL_4026301, EPI_ISL_4026302, EPI_ISL_4026303, EPI_ISL_4026304, EPI_ISL_4026305, EPI_ISL_4026306, EPI_ISL_4026307, EPI_ISL_4026308, EPI_ISL_4026309, EPI_ISL_4026310, EPI_ISL_4026311, EPI_ISL_4026312, EPI_ISL_4026313, EPI_ISL_4026314, EPI_ISL_4026315, EPI_ISL_4026316, EPI_ISL_4026319, EPI_ISL_4026321, EPI_ISL_4026322, EPI_ISL_4026323, EPI_ISL_4026324, EPI_ISL_4026325, EPI_ISL_4026326, EPI_ISL_4026327, EPI_ISL_4026328, EPI_ISL_4026329, EPI_ISL_4026330, EPI_ISL_4026331, EPI_ISL_4026332, EPI_ISL_4026333, EPI_ISL_4026334, EPI_ISL_4026335, EPI_ISL_4026336, EPI_ISL_4026337, EPI_ISL_4026338, EPI_ISL_4026339, EPI_ISL_4026340, EPI_ISL_4026341, EPI_ISL_4026342, EPI_ISL_4026343, EPI_ISL_4026344, EPI_ISL_4026345, EPI_ISL_4026346, EPI_ISL_4026347, EPI_ISL_4026348, EPI_ISL_4026349, EPI_ISL_4026350, EPI_ISL_4026351, EPI_ISL_4026352, EPI_ISL_4026353, EPI_ISL_4026354, EPI_ISL_4026355, EPI_ISL_4026356, EPI_ISL_4026357, EPI_ISL_4026358, EPI_ISL_4026359, EPI_ISL_4026360, EPI_ISL_4026361, EPI_ISL_4026362, EPI_ISL_4026363, EPI_ISL_4026364, EPI_ISL_4026365, EPI_ISL_4026366, EPI_ISL_4026367, EPI_ISL_4026368, EPI_ISL_4026369, EPI_ISL_4026370, EPI_ISL_4026371, EPI_ISL_4026372, EPI_ISL_4026373, EPI_ISL_4026374, EPI_ISL_4026375, EPI_ISL_4026378, EPI_ISL_4026379, EPI_ISL_4026380, EPI_ISL_4026381, EPI_ISL_4026382, EPI_ISL_4026384, EPI_ISL_4026385, EPI_ISL_4026386, EPI_ISL_4026387, EPI_ISL_4026388, EPI_ISL_4026389, EPI_ISL_4026390, EPI_ISL_4026391, EPI_ISL_4026392, EPI_ISL_4026393, EPI_ISL_4026394, EPI_ISL_4026395, EPI_ISL_4026396, EPI_ISL_4026397, EPI_ISL_4026398, EPI_ISL_4026399, EPI_ISL_4026400, EPI_ISL_4026401, EPI_ISL_4026402, EPI_ISL_4026403, EPI_ISL_4026404, EPI_ISL_4026405, EPI_ISL_4026406, EPI_ISL_4026407, EPI_ISL_4026408, EPI_ISL_4026409, EPI_ISL_4026410, EPI_ISL_4026411, EPI_ISL_4026412, EPI_ISL_4026413, EPI_ISL_4026414, EPI_ISL_4026415, EPI_ISL_4026416, EPI_ISL_4026417, EPI_ISL_4026418, EPI_ISL_4026420, EPI_ISL_4026421, EPI_ISL_4026422, EPI_ISL_4026423, EPI_ISL_4026425, EPI_ISL_4026426, EPI_ISL_4026427, EPI_ISL_4026428, EPI_ISL_4026429, EPI_ISL_4026430, EPI_ISL_4026435, EPI_ISL_4026436, EPI_ISL_4026438, EPI_ISL_4026439, EPI_ISL_4026440, EPI_ISL_4026441, EPI_ISL_4026442, EPI_ISL_4026443, EPI_ISL_4026444, EPI_ISL_4026445, EPI_ISL_4026446, EPI_ISL_4026447, EPI_ISL_4026448, EPI_ISL_4026449, EPI_ISL_4026450, EPI_ISL_4026451, EPI_ISL_4026452, EPI_ISL_4026455, EPI_ISL_4026456, EPI_ISL_4026457, EPI_ISL_4026458, EPI_ISL_4026459, EPI_ISL_4026460, EPI_ISL_4026461, EPI_ISL_4026462, EPI_ISL_4026463, EPI_ISL_4026464, EPI_ISL_4026465, EPI_ISL_4026466, EPI_ISL_4026467, EPI_ISL_4026468, EPI_ISL_4026470, EPI_ISL_4026471, EPI_ISL_4026472, EPI_ISL_4026473, EPI_ISL_4026474, EPI_ISL_4026475, EPI_ISL_4026476, EPI_ISL_4026477, EPI_ISL_4026479, EPI_ISL_4026480, EPI_ISL_4026481, EPI_ISL_4026482, EPI_ISL_4026483, EPI_ISL_4026484, EPI_ISL_4026485, EPI_ISL_4026486, EPI_ISL_4026487, EPI_ISL_4026488, EPI_ISL_4026489, EPI_ISL_4026490, EPI_ISL_4026491, EPI_ISL_4026492, EPI_ISL_4026493, EPI_ISL_4026494, EPI_ISL_4026495, EPI_ISL_4026496, EPI_ISL_4026497, EPI_ISL_4026498, EPI_ISL_4026499, EPI_ISL_4026500, EPI_ISL_4026501, EPI_ISL_4026502, EPI_ISL_4026503, EPI_ISL_4026504, EPI_ISL_4026505, EPI_ISL_4026506, EPI_ISL_4026507, EPI_ISL_4026508, EPI_ISL_4026509, EPI_ISL_4026510, EPI_ISL_4026595			
see above	Biorepository and Clinical Virology Laboratory	Northwestern University - Center for Pathogen Genomics and Microbial Evolution	Adeola A. Fowotade; Babafemi O. Taiwo; Egon A. Ozer; Ewean C. Omoruyi; Johnson A. Adeniji; Judd F. Hultquist; Lacy M. Simons; Olubusuyi M. Adewumi; Ramon Lorenzo-Redondo
EPI_ISL_5099366	Bioscience Research InstituteIbn Sina UniversityKhartoum, Sudan	Bioscience Research InstituteIbn Sina UniversityKhartoum, Sudan	Charles Kayuki; Elwaleed M. Elamin; Khabab A. Elhag; Maowia M. Mukhtar; Mona O.A. Alkarim; Samira M. Fageer; Zeinab S. Imam
EPI_ISL_5067391	Bioscience Research institute	Bioscience Research institute	Charles Kayuki; Elwaleed M. Elamin; Maowia M. Mukhtar; Mona O.A.Awadelkareem Khaba.A. Elhag; Sameira M. Fageer; Sara A.I Latif; Tomader. A.M.Ibrahim; Zeinab S. Imam
EPI_ISL_5510365, EPI_ISL_5510443	Braamfontein Central	National Institute for Communicable Diseases of the National Health Laboratory Service	Amoako DG; Bhiman JN; Everatt J; Ismail A; Mahlangu B; Mnguni A; Mohale T; Ntuli N; Scheepers C
EPI_ISL_3804019, EPI_ISL_3804020, EPI_ISL_3804021, EPI_ISL_3804022, EPI_ISL_3804023, EPI_ISL_3804025, EPI_ISL_3804026, EPI_ISL_3804030, EPI_ISL_3804031, EPI_ISL_3804032, EPI_ISL_3804033, EPI_ISL_3804034, EPI_ISL_3804035, EPI_ISL_3804036, EPI_ISL_3804056, EPI_ISL_3804057, EPI_ISL_3804058, EPI_ISL_3804059, EPI_ISL_3804060, EPI_ISL_3804061, EPI_ISL_3804062, EPI_ISL_3804063, EPI_ISL_3804064, EPI_ISL_3804066, EPI_ISL_3804067, EPI_ISL_3804068, EPI_ISL_3804258, EPI_ISL_3804260, EPI_ISL_3804262, EPI_ISL_3804264, EPI_ISL_3804268, EPI_ISL_3804270, EPI_ISL_3804272, EPI_ISL_3804274, EPI_ISL_3804276, EPI_ISL_3804278, EPI_ISL_3804280, EPI_ISL_3804282, EPI_ISL_3804284, EPI_ISL_3804286, EPI_ISL_3804288, EPI_ISL_3804289, EPI_ISL_3804291, EPI_ISL_3804293			
see above	CERMES	Institut Pasteur de Dakar	Adamou Lagare; Dhwani Batra; Dia Ndongo; Diagne Moussa Moïse; Diallo Amadou; Faye Ousmane; Justin Lee; Loucoubar Cheikh; Ndiaye Ndack; Sall Amadou Alpha; Sankhe Safietou
EPI_ISL_3262210	CPHL,MOH,EGYPT	CPHL	Abd Monaem Adel; Amelia nagiub; Dalia Ramadan; Galal Mahmoud; Mohamed A Ali; Mohamed Hassany; Mohamed Kamal; Nancy el guindy; Rabeh ..R. El-Shesheny; Ramy Galal; Shymaa s. Ahmed; Wael H. Roshdy; salma sayed
EPI_ISL_3274158, EPI_ISL_3274160, EPI_ISL_3274162, EPI_ISL_3274163	CPHL/MOH/EGYPT	CPHL/MOH/EGYPT	Wael H. Roshdy/ Mohamed Kamal / Shymaa s. Ahmed/ Ramy Galal/Nancy el guindy/ Amel nagiub/yasser el hady/salma sayed/ Abd Monaem Adel/Galal Mahmoud/Dalia Ramadan/Rabeh ..R. El/Shesheny/ Mohamed A Ali/Mohamed Hassany; Wael H. Roshdy/ Mohamed Kamal / Shymaa s. Ahmed/ Ramy Galal/Nancy el guindy/ Amelia nagiub/salma sayed/ Abd Monaem Adel/Galal Mahmoud/Dalia Ramadan/Rabeh ..R. El/Shesheny/ Mohamed A Ali/Mohamed Hassany
EPI_ISL_4255144, EPI_ISL_4255150, EPI_ISL_4255151, EPI_ISL_4255152, EPI_ISL_4255153, EPI_ISL_4255154, EPI_ISL_4255155, EPI_ISL_4255156, EPI_ISL_4255157, EPI_ISL_4255158, EPI_ISL_4255159			
see above	Centre Hospitalier Universitaire Yalgado OUEDRAOGO	Africa Centre for Excellence for Genomics of Infectious Diseases (ACEGID), Redeemer's University	A.T.; Abechi; Ajogbasile; Akano; C.A.; C.T.; Eromon; F.V.; Folarin, O.; Happi; I.B.; J.N.; J.U.; K.O.; Kayode; Nosamiefan, I.; Oguzie; Olawoye; Olumade; Oluniyi; P.E.; P.S.; T.J.; Ugwu; Uwanibe
EPI_ISL_4255126, EPI_ISL_4255127, EPI_ISL_4255128, EPI_ISL_4255129, EPI_ISL_4255130, EPI_ISL_4255131, EPI_ISL_4255133, EPI_ISL_4255134, EPI_ISL_4255135, EPI_ISL_4255136, EPI_ISL_4255137, EPI_ISL_4255138, EPI_ISL_4255139, EPI_ISL_4255140, EPI_ISL_4255141, EPI_ISL_4255143			
see above	Centre Hospitalier Universitaire de Bogodogo	Africa Centre for Excellence for Genomics of Infectious Diseases (ACEGID), Redeemer's University	A.T.; Abechi; Ajogbasile; Akano; C.A.; C.T.; Eromon; F.V.; Folarin, O.; Happi; I.B.; J.N.; J.U.; K.O.; Kayode; Nosamiefan, I.; Oguzie; Olawoye; Olumade; Oluniyi; P.E.; P.S.; T.J.; Ugwu; Uwanibe
EPI_ISL_4255006, EPI_ISL_4255007, EPI_ISL_4255008, EPI_ISL_4255009, EPI_ISL_4255010, EPI_ISL_4255011, EPI_ISL_4255012, EPI_ISL_4255014, EPI_ISL_4255016, EPI_ISL_4255017, EPI_ISL_4255018, EPI_ISL_4255019, EPI_ISL_4255023, EPI_ISL_4255024, EPI_ISL_4255025, EPI_ISL_4255026, EPI_ISL_4255027, EPI_ISL_4255028, EPI_ISL_4255029, EPI_ISL_4255030, EPI_ISL_4255031, EPI_ISL_4255032, EPI_ISL_4255033			
see above	Centre Hospitalier Universitaire de Tengandogo	Africa Centre for Excellence for Genomics of Infectious Diseases (ACEGID), Redeemer's University	A.T.; Abechi; Ajogbasile; Akano; C.A.; C.T.; Eromon; F.V.; Folarin, O.; Happi; I.B.; J.N.; J.U.; K.O.; Kayode; Nosamiefan, I.; Oguzie; Olawoye; Olumade; Oluniyi; P.E.; P.S.; T.J.; Ugwu; Uwanibe
EPI_ISL_4255070, EPI_ISL_4255071, EPI_ISL_4255072, EPI_ISL_4255073, EPI_ISL_4255074, EPI_ISL_4255075, EPI_ISL_4255076, EPI_ISL_4255077, EPI_ISL_4255078, EPI_ISL_4255079, EPI_ISL_4255080, EPI_ISL_4255081, EPI_ISL_4255082, EPI_ISL_4255083, EPI_ISL_4255084, EPI_ISL_4255085, EPI_ISL_4255086, EPI_ISL_4255087, EPI_ISL_4255088, EPI_ISL_4255089, EPI_ISL_4255090, EPI_ISL_4255160, EPI_ISL_4255161, EPI_ISL_4255162, EPI_ISL_4255163, EPI_ISL_4255164, EPI_ISL_4255165			
see above	Centre MURAZ	Africa Centre for Excellence for Genomics of Infectious Diseases (ACEGID), Redeemer's University	A.T.; Abechi; Ajogbasile; Akano; C.A.; C.T.; Eromon; F.V.; Folarin, O.; Happi; I.B.; J.N.; J.U.; K.O.; Kayode; Nosamiefan, I.; Oguzie; Olawoye; Olumade; Oluniyi; P.E.; P.S.; T.J.; Ugwu; Uwanibe
EPI_ISL_3246522, EPI_ISL_3246523, EPI_ISL_3246524, EPI_ISL_3246525, EPI_ISL_3246526, EPI_ISL_3246527, EPI_ISL_3246528, EPI_ISL_3246529, EPI_ISL_3246531, EPI_ISL_3246532			
see above	Centro de Investigação em Saúde de Manhiça (CISM) & ISGlobal, Institut de Salut Global de Barcelona	Instituto de Biomedicina de València - CSIC	Alfredo Mayor; Arsenia Massinga; Inacio Mandomando; Irving Cancino; Iñaki Comas; Manoli Torres Puente; Santiago Jiménez-Serrano
EPI_ISL_3957789	Conville CDC wc CVC	NHLS/UCT	Arash Iranzadeh; Bruna Galvao; Carolyn Williamson; Deelan Doolabh; Diana Hardie; Gert Marais; Innocent Mudau; Lynn Tyers; Marvin Hsiao; Rageema Joseph; Stephen Korsman
EPI_ISL_3342336, EPI_ISL_3342338, EPI_ISL_3342339, EPI_ISL_3342340, EPI_ISL_3342342, EPI_ISL_3342344, EPI_ISL_3342346, EPI_ISL_3342347, EPI_ISL_3342348, EPI_ISL_3411472			
see above	DGM Laboratory	National Institute for Communicable Diseases of the National Health Laboratory Service	Amoako DG; Bhiman JN; Everatt J; Ismail A; Mahlangu B; Mnguni A; Mohale T; Ntuli N; Scheepers C
EPI_ISL_3342385, EPI_ISL_3342402, EPI_ISL_3342411, EPI_ISL_3342414	De Aar Laboratory	National Institute for Communicable Diseases of the National Health Laboratory Service	Amoako DG; Bhiman JN; Everatt J; Ismail A; Mahlangu B; Mnguni A; Mohale T; Ntuli N; Scheepers C
EPI_ISL_5797285, EPI_ISL_5797286, EPI_ISL_5797287, EPI_ISL_5797288, EPI_ISL_5797289, EPI_ISL_5797290, EPI_ISL_5797291, EPI_ISL_5797292, EPI_ISL_5797293, EPI_ISL_5797294, EPI_ISL_5797295, EPI_ISL_5797296, EPI_ISL_5797297, EPI_ISL_5797298, EPI_ISL_5797299, EPI_ISL_5797300, EPI_ISL_5797301, EPI_ISL_5797302, EPI_ISL_5797303, EPI_ISL_5797304, EPI_ISL_5797305, EPI_ISL_5797306, EPI_ISL_5797307, EPI_ISL_5797308, EPI_ISL_5797309, EPI_ISL_5797310, EPI_ISL_5797311, EPI_ISL_5797312, EPI_ISL_5797313, EPI_ISL_5797314, EPI_ISL_5797315, EPI_ISL_5797316, EPI_ISL_5797317, EPI_ISL_5797318, EPI_ISL_5797319, EPI_ISL_5797320, EPI_ISL_5797321, EPI_ISL_5797322, EPI_ISL_5797323, EPI_ISL_5797324, EPI_ISL_5797325, EPI_ISL_5797326, EPI_ISL_5797327, EPI_ISL_5797328, EPI_ISL_5797329, EPI_ISL_5797330, EPI_ISL_5797331, EPI_ISL_5797332, EPI_ISL_5797333, EPI_ISL_5797334, EPI_ISL_5797335, EPI_ISL_5797336, EPI_ISL_5797337, EPI_ISL_5797338, EPI_ISL_5797339, EPI_ISL_5797340, EPI_ISL_5797341, EPI_ISL_5797342, EPI_ISL_5797343, EPI_ISL_5797344, EPI_ISL_5797346, EPI_ISL_5797347, EPI_ISL_5797348, EPI_ISL_5797349, EPI_ISL_5797350, EPI_ISL_5797351, EPI_ISL_5797352, EPI_ISL_5797363, EPI_ISL_5797364, EPI_ISL_5797365, EPI_ISL_5797366, EPI_ISL_5797367, EPI_ISL_5797368, EPI_ISL_5797369, EPI_ISL_5797385, EPI_ISL_5797386, EPI_ISL_5797387, EPI_ISL_5797442, EPI_ISL_5797443, EPI_ISL_5797444, EPI_ISL_5797445, EPI_ISL_5797446, EPI_ISL_5797447, EPI_ISL_5797448, EPI_ISL_5797449, EPI_ISL_5797450, EPI_ISL_5797451, EPI_ISL_5797452			
see above	Epidemiology and Demography, KEMRI-Wellcome Trust Research Programme	Epidemiology and Demography, KEMRI-Wellcome Trust Research Programme	A.W.; Agoti; C.N.; D.J.; Githinji, G.; I.L.; J.M.; K.S.; Lambisia; M.W.; Makori, T.; Mburu; Mohammed; Morobe; Ndwiga, L.; Nokes; Ochola-Oyier; Ongera, E.; Onyango, C.; de Laurent, Z.
EPI_ISL_3957788, EPI_ISL_4884453, EPI_ISL_4884455, EPI_ISL_5195382	Groote Schuur Hospital wc GSH	NHLS/UCT	Arash Iranzadeh; Bruna Galvao; Carolyn Williamson; Deelan Doolabh; Diana Hardie; Gert Marais; Innocent Mudau; Lynn Tyers; Marvin Hsiao; Rageema Joseph; Stephen Korsman
EPI_ISL_5054116	IHU Mediterranean Infection	IRESSEF	Abdou Padane; Ambroise Ahouidi; Aminata Mbaye; Aminata Mboup; Cheikh Sokhna; Didier Raoult; Florence Fenollar; Hubert Bassène; Mamadou Beye Cheikh Ibrahima Lo; Masse Sambou; Moustapha Mbow; Nafissatou Leye; Philippe Colson; Philippe LAVRARD MEYER; Sokhna Ndongo; Souleymane Mboup and Pierre-Edouard Fournier; Yacine Amet Dia
EPI_ISL_5054148, EPI_ISL_5054153, EPI_ISL_5054172	IRESSEF	IHU Mediterranean Infection	Abdou Padane; Ambroise Ahouidi; Aminata Mbaye; Aminata Mboup; Cheikh Sokhna; Didier Raoult; Florence Fenollar; Hubert Bassène; Mamadou Beye Cheikh Ibrahima Lo; Masse Sambou; Moustapha Mbow; Nafissatou Leye; Philippe Colson; Philippe LAVRARD MEYER; Sokhna Ndongo; Souleymane Mboup and Pierre-Edouard Fournier; Yacine Amet Dia
EPI_ISL_5054155, EPI_ISL_5054176	IRESSEF	IUH Mediterranean Infection	Abdou Padane; Ambroise Ahouidi; Aminata Mbaye; Aminata Mboup; Cheikh Sokhna; Didier Raoult; Florence Fenollar; Hubert Bassène; Mamadou Beye Cheikh Ibrahima Lo; Masse Sambou; Moustapha Mbow; Nafissatou Leye; Philippe Colson; Philippe LAVRARD MEYER; Sokhna Ndongo; Souleymane Mboup and Pierre-Edouard Fournier; Yacine Amet Dia
EPI_ISL_4251927, EPI_ISL_4251928, EPI_ISL_4251929, EPI_ISL_4251930, EPI_ISL_4251931, EPI_ISL_4251932, EPI_ISL_4251933, EPI_ISL_4251934, EPI_ISL_4251935, EPI_ISL_4251936, EPI_ISL_4251937, EPI_ISL_4251938, EPI_ISL_4251939, EPI_ISL_4251940, EPI_ISL_4251941, EPI_ISL_4251942, EPI_ISL_4251943, EPI_ISL_4251944, EPI_ISL_4251945, EPI_ISL_4251946, EPI_ISL_4251947, EPI_ISL_4251948, EPI_ISL_4251949, EPI_ISL_4251950, EPI_ISL_4251951, EPI_ISL_4251952, EPI_ISL_4251953, EPI_ISL_4251954, EPI_ISL_4251955, EPI_ISL_4251956, EPI_ISL_4251960, EPI_ISL_4251962, EPI_ISL_4251963, EPI_ISL_4251964, EPI_ISL_4251965, EPI_ISL_4251966, EPI_ISL_4251967, EPI_ISL_4251968, EPI_ISL_4251969, EPI_ISL_4251970, EPI_ISL_4251971, EPI_ISL_4251972, EPI_ISL_4251985, EPI_ISL_4251986, EPI_ISL_4251991, EPI_ISL_4251992, EPI_ISL_4251993, EPI_ISL_4251998, EPI_ISL_4252000, EPI_ISL_4252001, EPI_ISL_4252002, EPI_ISL_4252003, EPI_ISL_4252004, EPI_ISL_4252005, EPI_ISL_4252006, EPI_ISL_4252007, EPI_ISL_4252008, EPI_ISL_4252011, EPI_ISL_4252013, EPI_ISL_4252014, EPI_ISL_4252016, EPI_ISL_4252017, EPI_ISL_4252018, EPI_ISL_4252019, EPI_ISL_4252020, EPI_ISL_4252021, EPI_ISL_4252022, EPI_ISL_4252023, EPI_ISL_4252024, EPI_ISL_4252025, EPI_ISL_4252026, EPI_ISL_4252027, EPI_ISL_4252029, EPI_ISL_4252030, EPI_ISL_4252031, EPI_ISL_4252032, EPI_ISL_4252033, EPI_ISL_4252036, EPI_ISL_4252040, EPI_ISL_4252041, EPI_ISL_4252042, EPI_ISL_4252044, EPI_ISL_4252046, EPI_ISL_4252047, EPI_ISL_4252048, EPI_ISL_4252049, EPI_ISL_4252051, EPI_ISL_4252052, EPI_ISL_4252053, EPI_ISL_4252054, EPI_ISL_4252055, EPI_ISL_4252056, EPI_ISL_4252057, EPI_ISL_4252058, EPI_ISL_4252059, EPI_ISL_4252061, EPI_ISL_4252062, EPI_ISL_4252065, EPI_ISL_4252066, EPI_ISL_4252067, EPI_ISL_4252068, EPI_ISL_4252070, EPI_ISL_4252071, EPI_ISL_4252075, EPI_ISL_4252077, EPI_ISL_4252081, EPI_ISL_4252083, EPI_ISL_4252084, EPI_ISL_4252085, EPI_ISL_4252088, EPI_ISL_4252089, EPI_ISL_4252090, EPI_ISL_4252091, EPI_ISL_4252093, EPI_ISL_4252094, EPI_ISL_4252095, EPI_ISL_4252096, EPI_ISL_4252097, EPI_ISL_4252098, EPI_ISL_4252099, EPI_ISL_4252100, EPI_ISL_4252102, EPI_ISL_4252103, EPI_ISL_4252105, EPI_ISL_4252106, EPI_ISL_4252108, EPI_ISL_4252109, EPI_ISL_4252110, EPI_ISL_4252111, EPI_ISL_4252112, EPI_ISL_4252113, EPI_ISL_4252115, EPI_ISL_4252116, EPI_ISL_4252118, EPI_ISL_4252119, EPI_ISL_4252120, EPI_ISL_4252123, EPI_ISL_4252124, EPI_ISL_4252128, EPI_ISL_4252130, EPI_ISL_4252133, EPI_ISL_4252135, EPI_ISL_4252136, EPI_ISL_4252138, EPI_ISL_4252139, EPI_ISL_4252140, EPI_ISL_4252141, EPI_ISL_4252144, EPI_ISL_4252145, EPI_ISL_4252146, EPI_ISL_4252147, EPI_ISL_4252150, EPI_ISL_4252151, EPI_ISL_4252152, EPI_ISL_4252156, EPI_ISL_4252157, EPI_ISL_4252159, EPI_ISL_4252160, EPI_ISL_4252161, EPI_ISL_4252164, EPI_ISL_4252167, EPI_ISL_4252169, EPI_ISL_4252170, EPI_ISL_4252171, EPI_ISL_4252172, EPI_ISL_4252173, EPI_ISL_4252175, EPI_ISL_4252176, EPI_ISL_4252177, EPI_ISL_4252178, EPI_ISL_4252179, EPI_ISL_4252181, EPI_ISL_4252185, EPI_ISL_4252187, EPI_ISL_4252188, EPI_ISL_4252190, EPI_ISL_4252195, EPI_ISL_4252196, EPI_ISL_4252197, EPI_ISL_4252198, EPI_ISL_4252199, EPI_ISL_4252200, EPI_ISL_4252201, EPI_ISL_4252202, EPI_ISL_4252203, EPI_ISL_4252205, EPI_ISL_4252206, EPI_ISL_4252207, EPI_ISL_4252208, EPI_ISL_4252212, EPI_ISL_4252213, EPI_ISL_4252237, EPI_ISL_4252240, EPI_ISL_4252253, EPI_ISL_4252254, EPI_ISL_4252257, EPI_ISL_4252260, EPI_ISL_4252261, EPI_ISL_4252265, EPI_ISL_4252267, EPI_ISL_4252271, EPI_ISL_4252272, EPI_ISL_4252275, EPI_ISL_4252276, EPI_ISL_4252277, EPI_ISL_4252282, EPI_ISL_4252283, EPI_ISL_4252284, EPI_ISL_4252286, EPI_ISL_4252289, EPI_ISL_4252290, EPI_ISL_4252291, EPI_ISL_4252292, EPI_ISL_4252294, EPI_ISL_4252295, EPI_ISL_4252296, EPI_ISL_4252297, EPI_ISL_4252301, EPI_ISL_4252303, EPI_ISL_4252304, EPI_ISL_4252305, EPI_ISL_4252310, EPI_ISL_4252311, EPI_ISL_4252313, EPI_ISL_4252314, EPI_ISL_4252316, EPI_ISL_4252317, EPI_ISL_4252318, EPI_ISL_4252319, EPI_ISL_4252320, EPI_ISL_4252321, EPI_ISL_4252322, EPI_ISL_4252323, EPI_ISL_4252324, EPI_ISL_4252325, EPI_ISL_4252326, EPI_ISL_4252327, EPI_ISL_4252328, EPI_ISL_4252329, EPI_ISL_4252330, EPI_ISL_4252331, EPI_ISL_4252340, EPI_ISL_4252346, EPI_ISL_4252347, EPI_ISL_4252350, EPI_ISL_4252352, EPI_ISL_4252358, EPI_ISL_4252361, EPI_ISL_4252364, EPI_ISL_4252365, EPI_ISL_4252366, EPI_ISL_4252370, EPI_ISL_4252372, EPI_ISL_4252373, EPI_ISL_4252374, EPI_ISL_4252375, EPI_ISL_4252376, EPI_ISL_4252377, EPI_ISL_4252379, EPI_ISL_4252380, EPI_ISL_4252382, EPI_ISL_4252383, EPI_ISL_4252385, EPI_ISL_4252387, EPI_ISL_4252389, EPI_ISL_4252390, EPI_ISL_4252391, EPI_ISL_4252392, EPI_ISL_4252394, EPI_ISL_4252396, EPI_ISL_4252397, EPI_ISL_4252398, EPI_ISL_4252400, EPI_ISL_4252401, EPI_ISL_4252403, EPI_ISL_4252405, EPI_ISL_4252406, EPI_ISL_4252407, EPI_ISL_4252409, EPI_ISL_4252410, EPI_ISL_4252411, EPI_ISL_4252412, EPI_ISL_4252413, EPI_ISL_4252414, EPI_ISL_4252415, EPI_ISL_4252416, EPI_ISL_4252417, EPI_ISL_4252419, EPI_ISL_4252420, EPI_ISL_4252421, EPI_ISL_4252423, EPI_ISL_4252424, EPI_ISL_4252426, EPI_ISL_4252427, EPI_ISL_4252428, EPI_ISL_4252429, EPI_ISL_4252430, EPI_ISL_4252432, EPI_ISL_4252434, EPI_ISL_4252435, EPI_ISL_4252436, EPI_ISL_4252438, EPI_ISL_4252441, EPI_ISL_4252443, EPI_ISL_4252446, EPI_ISL_4252448, EPI_ISL_4252449, EPI_ISL_4252451, EPI_ISL_4252452, EPI_ISL_4252453, EPI_ISL_4252454, EPI_ISL_4252456, EPI_ISL_4252458, EPI_ISL_4252459, EPI_ISL_4252460, EPI_ISL_4252461, EPI_ISL_4252462, EPI_ISL_4252464, EPI_ISL_4252466, EPI_ISL_4252468, EPI_ISL_4252469, EPI_ISL_4252470, EPI_ISL_4252471, EPI_ISL_4252472, EPI_ISL_4252473, EPI_ISL_4252476, EPI_ISL_4252477, EPI_ISL_4252478, EPI_ISL_4252479, EPI_ISL_4252480, EPI_ISL_4252481, EPI_ISL_4252483, EPI_ISL_4252484, EPI_ISL_4252485, EPI_ISL_4252486, EPI_ISL_4252489, EPI_ISL_4252490, EPI_ISL_4252491, EPI_ISL_4252492, EPI_ISL_4252493, EPI_ISL_4252494, EPI_ISL_4252495, EPI_ISL_4252496, EPI_ISL_4252497, EPI_ISL_4252498, EPI_ISL_4252499, EPI_ISL_4252500, EPI_ISL_4252501, EPI_ISL_4252506, EPI_ISL_4252507, EPI_ISL_4252509, EPI_ISL_4252512, EPI_ISL_4252513, EPI_ISL_4252516, EPI_ISL_4252517, EPI_ISL_4252518, EPI_ISL_4252520, EPI_ISL_4252521, EPI_ISL_4252522, EPI_ISL_4252523, EPI_ISL_4252524, EPI_ISL_4252526, EPI_ISL_4252527, EPI_ISL_4252528, EPI_ISL_4252529, EPI_ISL_4252530, EPI_ISL_4252531, EPI_ISL_4252532, EPI_ISL_4252533, EPI_ISL_4252534, EPI_ISL_4252536, EPI_ISL_4252537, EPI_ISL_4252546, EPI_ISL_4252547, EPI_ISL_4252549, EPI_ISL_4252550, EPI_ISL_4252552, EPI_ISL_4252553			

see above	International Livestock Research Institute	International Livestock Research Institute	Collins Muli; Daniel Ouso; Edward Kiritu; Edward O. Abworo; Gilbert Kibet; Patrick Amoth; Paul Dobi; Samuel O. Oyola; Shebbar Osiany; Sonal P. Henson; Vishvanath Nene
EPI_ISL_4276972	Iressef Genomics lab	IRESSEF	Abdou PADANE; Ambroise AHOUIDI; Aminata DIA; Aminata MBOUP; Astou Gaye GAYE; Barada CISSE; Birahim Piere NDIAYE; Diabou Diagne; Gora LO; Khadim GUEYE; Moustapha MBOW; Nafisatou LEYE; Ndeye Coumba Toure KANE; Papa Alassane DIAW; Samba Ndiour; Seni Ndiaye; Souleymane MBOUP; Yacine DIA
EPI_ISL_4178268, EPI_ISL_4178270, EPI_ISL_4178271, EPI_ISL_4178273, EPI_ISL_4178276, EPI_ISL_4178278, EPI_ISL_4178281, EPI_ISL_4178285, EPI_ISL_4178286, EPI_ISL_4178288, EPI_ISL_4178292, EPI_ISL_4178294, EPI_ISL_4178295, EPI_ISL_4178301, EPI_ISL_4178308, EPI_ISL_4178310, EPI_ISL_4178313, EPI_ISL_4178315, EPI_ISL_4178319, EPI_ISL_4178320, EPI_ISL_4178326, EPI_ISL_4178328, EPI_ISL_4178329, EPI_ISL_4178331, EPI_ISL_4178333, EPI_ISL_4178335, EPI_ISL_4178338, EPI_ISL_4178341, EPI_ISL_4178344, EPI_ISL_4178346, EPI_ISL_4178348, EPI_ISL_4178350, EPI_ISL_4178351, EPI_ISL_4178353, EPI_ISL_4178357, EPI_ISL_4178359, EPI_ISL_4178360, EPI_ISL_4178362, EPI_ISL_4178364, EPI_ISL_4178368, EPI_ISL_4178369, EPI_ISL_4178371, EPI_ISL_4178373, EPI_ISL_4178376, EPI_ISL_4178378, EPI_ISL_4178380, EPI_ISL_4178382, EPI_ISL_4178383, EPI_ISL_4178385, EPI_ISL_4178387, EPI_ISL_4178389, EPI_ISL_4178390, EPI_ISL_4178392, EPI_ISL_4178394			
see above	Irrua Specialist Teaching Hospital	Africa Centre for Excellence for Genomics of Infectious Diseases (ACEGID), Redeemer's University	A.T.; Abechi; Ajogbasile; Akano; C.A.; C.T.; Eromon; F.V.; Folarin, O.; Happi; I.B.; J.N.; J.U.; K.O.; Kayode; Nosamiefan, I.; Oguzie; Olawoye; Olumade; Oluniyi; P.E.; P.S.; T.J.; Ugwu; Uwanibe
EPI_ISL_3342332, EPI_ISL_3342333, EPI_ISL_3342334, EPI_ISL_3342335, EPI_ISL_3342337, EPI_ISL_3342341, EPI_ISL_3342343, EPI_ISL_3342345, EPI_ISL_3342349			
see above	Jubilee laboratory	National Institute for Communicable Diseases of the National Health Laboratory Service	Amoako DG; Bhiman JN; Everatt J; Ismail A; Mahlangu B; Mnguni A; Mohale T; Ntuli N; Scheepers C
EPI_ISL_5416274	KOPANONG LABORATORY	National Institute for Communicable Diseases of the National Health Laboratory Service	Amoako DG; Bhiman JN; Everatt J; Ismail A; Mahlangu B; Mnguni A; Mohale T; Ntuli N; Scheepers C
EPI_ISL_5510310	Kimberley	National Institute for Communicable Diseases of the National Health Laboratory Service	Amoako DG; Bhiman JN; Everatt J; Ismail A; Mahlangu B; Mnguni A; Mohale T; Ntuli N; Scheepers C
EPI_ISL_3342352, EPI_ISL_3342355, EPI_ISL_3342357, EPI_ISL_3342367, EPI_ISL_3342379, EPI_ISL_3342390, EPI_ISL_3342391, EPI_ISL_3342393, EPI_ISL_3342399, EPI_ISL_3342400, EPI_ISL_3342401, EPI_ISL_3342405, EPI_ISL_3342406, EPI_ISL_3342408, EPI_ISL_3342412, EPI_ISL_3342416, EPI_ISL_3342417, EPI_ISL_3342419, EPI_ISL_3342421			
see above	Kimberley laboratory	National Institute for Communicable Diseases of the National Health Laboratory Service	Amoako DG; Bhiman JN; Everatt J; Ismail A; Mahlangu B; Mnguni A; Mohale T; Ntuli N; Scheepers C
EPI_ISL_5510438	Kopanong	National Institute for Communicable Diseases of the National Health Laboratory Service	Amoako DG; Bhiman JN; Everatt J; Ismail A; Mahlangu B; Mnguni A; Mohale T; Ntuli N; Scheepers C
EPI_ISL_4877371, EPI_ISL_4877372, EPI_ISL_4877373	LBV Le DANTEC	LBV Le DANTEC	Adjiratou Aissatou BA; Aminata Sileymane Thiam; Anna julienne Selbe NDiaye; Assane Dieng; Awa Ba-Diallo; Dianke Samaté; Gora Lo; Halimatou Diop Ndiaye; Khadim GUEYE; Makhtar Camara; Mbengué Fall; Moustapha Sakho; Oumy DIOP; Pascaline Manga; Pauline Yacine Sene; Sada Diallo; Samba NDOUR; Serigne Saliou Niane; ousseynou Gueye
EPI_ISL_5123542, EPI_ISL_5123575	Laboratoire Biolife	Laboratoire de Biotechnologie	Afaf Alaoui; Amina Benouda; Bouchra Belfquih; Hanae Dakka; Lahcen belyamani; Mohamed Chenaoui; Mohammed Walid Chemao Elfihri; Mouna Ouadghiri; Otmane Touzani; Saaid Amzazi and Azeddine Ibrahimi; Tarik Aanniz
EPI_ISL_4255048, EPI_ISL_4255049, EPI_ISL_4255050, EPI_ISL_4255051, EPI_ISL_4255052, EPI_ISL_4255053, EPI_ISL_4255054, EPI_ISL_4255055, EPI_ISL_4255056, EPI_ISL_4255057, EPI_ISL_4255058, EPI_ISL_4255059, EPI_ISL_4255060, EPI_ISL_4255061, EPI_ISL_4255062, EPI_ISL_4255063, EPI_ISL_4255064, EPI_ISL_4255065, EPI_ISL_4255066, EPI_ISL_4255067, EPI_ISL_4255068, EPI_ISL_4255069			
see above	Laboratoire National de Santé Publique	Africa Centre for Excellence for Genomics of Infectious Diseases (ACEGID), Redeemer's University	A.T.; Abechi; Ajogbasile; Akano; C.A.; C.T.; Eromon; F.V.; Folarin, O.; Happi; I.B.; J.N.; J.U.; K.O.; Kayode; Nosamiefan, I.; Oguzie; Olawoye; Olumade; Oluniyi; P.E.; P.S.; T.J.; Ugwu; Uwanibe
EPI_ISL_3506226, EPI_ISL_3506356, EPI_ISL_3506508, EPI_ISL_3506722, EPI_ISL_3506968, EPI_ISL_3506969, EPI_ISL_3507055, EPI_ISL_3507233			
see above	Laboratoire Professeur Daniel GAHOUMA	Centre de Recherches Médicales de Lambaréné	; Abe Haruka; Ayola Akim Adegnika and Bertrand Lel; Ayola Akim Adegnika and Bertrand Lell; Ayong More; Davy Leger Mouangala; Elvyre Mbongo-Nkama; Emilio Skarwan; Georgelin Nguema Ondo; Guy Stéphane Padzys; Gédéon Prince Manouana; Jean Bernard Lekana-Douki; Jiro Yasuda; Joël-Fleury Djoba Siawaya; Kevine Zang Ella; Ludovic Mewono; Maradona Agbanrin Daouda; Moustapha Nzamba Maloum; Nadine N'dilimabaka; Noé Patrick Mbondoukwe; Paulin N Essone; Rodrigue Bikangui; Rodrigue Mintsa Nguema; Sam O'neilla Oye Bingono; Samira Zoa Assoumou; Sandrine Zeh Nfor; Sonia Etenna Lekana-Douki; Srinivas reddy Pallerla; Yuri Ushijima
EPI_ISL_5061472	Laboratoire central de Virologie	Laboratoire de Biotechnologie	Lahcen Belyamani and Azeddine Ibrahimi; Mouna Ouadghiri; Saaïd Amzazi; Safae El Mazouri; Tarik Aanniz
EPI_ISL_4299860	Laboratoire de Recherche et d'Analyses Medicales de la Gendarmerie Royale	Laboratoire de Recherche et d'Analyses Medicales de la Gendarmerie Royale	Amal SOUIRI; Hajar LEMRISS; Mohamed LABIOUI; Nabil LEMZAOUI; Sanaâ LEMRISS; Saâd EL KABBAJ
EPI_ISL_4299861	Laboratoire de Recherche et d'Analyses Medicales de la Gendarmerie Royale	Laboratoire de Recherche et d'Analyses Médicales de la Gendarmerie Royale	Amal SOUIRI; Hajar LEMRISS; Mohamed LABIOUI; Nabil LEMZAOUI; Sanaâ LEMRISS; Saâd EL KABBAJ
EPI_ISL_3250687, EPI_ISL_4300209	Laboratoire de Recherche et d'Analyses Médicales de la Gendarmerie Royale	Laboratoire de Recherche et d'Analyses Médicales de la Gendarmerie Royale	Amal SOUIRI; Hajar LEMRISS; Mohamed LABIOUI; Nabil LEMZAOUI; Sanaâ LEMRISS; Saâd EL KABBAJ
EPI_ISL_4567011, EPI_ISL_4567013, EPI_ISL_4567015, EPI_ISL_4567016, EPI_ISL_4567018, EPI_ISL_4567020, EPI_ISL_4567022, EPI_ISL_4567023, EPI_ISL_4567025, EPI_ISL_4567027, EPI_ISL_4567029, EPI_ISL_4567031, EPI_ISL_4567032, EPI_ISL_4567034, EPI_ISL_4567036, EPI_ISL_4567038, EPI_ISL_4567039, EPI_ISL_4567041, EPI_ISL_4567043, EPI_ISL_4567045, EPI_ISL_4567046, EPI_ISL_4567048, EPI_ISL_4567050, EPI_ISL_4567052, EPI_ISL_4567054, EPI_ISL_4567056, EPI_ISL_4567058, EPI_ISL_4567059, EPI_ISL_4567061, EPI_ISL_4567063, EPI_ISL_4567065, EPI_ISL_4567067, EPI_ISL_4567069, EPI_ISL_4567071, EPI_ISL_4567072, EPI_ISL_4567074, EPI_ISL_4567076, EPI_ISL_4567078, EPI_ISL_4567080, EPI_ISL_4567081, EPI_ISL_4567083, EPI_ISL_4567085, EPI_ISL_4567087, EPI_ISL_4567089, EPI_ISL_4567090, EPI_ISL_4567092, EPI_ISL_4567094, EPI_ISL_4567096, EPI_ISL_4567098, EPI_ISL_4567099, EPI_ISL_4567101, EPI_ISL_4567103, EPI_ISL_4567105, EPI_ISL_4567107, EPI_ISL_4567108, EPI_ISL_4567110, EPI_ISL_4567112, EPI_ISL_4567114, EPI_ISL_4567116, EPI_ISL_4567118, EPI_ISL_4567120, EPI_ISL_4567122, EPI_ISL_4567124, EPI_ISL_4567126, EPI_ISL_4567128, EPI_ISL_4567130, EPI_ISL_4567132, EPI_ISL_4567133, EPI_ISL_4567135, EPI_ISL_4567137, EPI_ISL_4567139, EPI_ISL_4567141, EPI_ISL_4567143, EPI_ISL_4567144			
see above	Laboratoire des Fièvres Hémorragiques Virales du Benin	Institut für Virologie - Institute of Virology - Charite	Andres Moreira-Soto; Anges Yadouleton; Anna-Lena Sander; Ben Wulf; Benjamin Hounkpatin and Jan Felix Drexler; Carine Tchibozo; Christian Drosten; Clement G. Kakai; Dossou Ange; Edmilson F. de Oliveira Filho; Fattah Al Onifade; Gildas Hounkanrin; Keke K. René; Mamoudou Harouna Djingarey; Melchior A. Joël Aïssi; Michael Nagel; Petas Akogbeto; Praise Adewumi; Ramalia Chabi Nari; Raoul Saizonou; Rodrigue K. Kohoun; Sonia V. Bedié; Sourakatou Salifou
EPI_ISL_5416409	MAFIKENG LABORATORY	National Institute for Communicable Diseases of the National Health Laboratory Service	Amoako DG; Bhiman JN; Everatt J; Ismail A; Mahlangu B; Mnguni A; Mohale T; Ntuli N; Scheepers C
EPI_ISL_5641065	MRCG at LSHTM Genomics lab	MRCG at LSHTM Genomics lab	Abdoulie Kanteh; Abdul Karim sesay; Bakary Sanyang; Dabiri Damilari; Jarra Manneh; Mariama Kujabi
EPI_ISL_3770646, EPI_ISL_3770647, EPI_ISL_3770648, EPI_ISL_3770649, EPI_ISL_3770650, EPI_ISL_3770651	Malawi Liverpool Wellcome Trust Clinical Research Program	Malawi Liverpool Wellcome Trust CLinical Research Program	Ben Morton; Catherine Anscombe; Kondwani Jambo; Philip Ashton; Sam Lissauer
EPI_ISL_3342331	Mmametlhake laboratory	National Institute for Communicable Diseases of the National Health Laboratory Service	Amoako DG; Bhiman JN; Everatt J; Ismail A; Mahlangu B; Mnguni A; Mohale T; Ntuli N; Scheepers C
EPI_ISL_3545627, EPI_ISL_3545628, EPI_ISL_3545629, EPI_ISL_3545630, EPI_ISL_3545631, EPI_ISL_3545632, EPI_ISL_3545633, EPI_ISL_3545634, EPI_ISL_3545635, EPI_ISL_3545636, EPI_ISL_3545637, EPI_ISL_3545641, EPI_ISL_3545643, EPI_ISL_3545644, EPI_ISL_3545645, EPI_ISL_3545646, EPI_ISL_3545647			
see above	Molecular diagnostic unit for viral haemorrhagic fevers and emerging viruses, Bouake CHU Laboratory	Molecular diagnostic unit for viral haemorrhagic fevers and emerging viruses, Bouake CHU Laboratory	Adjaratou Traore; Bamba Fatoumata Toure; Chantal Akoua-Koffi; Coulibaly Mbegnan; Diane Bamourou; Essia Belarbi; Etile Anoh; Fabian Leendertz; Grit Schubert; Kra Ouffoue; Monemo Pacome; Oby Wayoro; Safiatou Karidioula; Soundele Maïte
EPI_ISL_5736811	Multidisciplinary Research Laboratory, Osun State	Central Research Laboratory	Akinde; Ayorinde Babatunde James; Azuka Patrick Okwuraiwe; Babatunde Lawal Salako; Bamidele Iwalokun; Chika Kingsley Onwuamah; Folorunsho; Grace Oni; Josiah Ayoola Isong; Joy Ayoola; Muinah Adenike Fowora; Ngozi Mirabel Otuonye; Nyam Itse Yusuf; Ojo; Olabisi; Phasha-Muchemenye Mmatshepho; Sola Ajibaye; Sunday Babatunde; Waidi Sule
EPI_ISL_5536462, EPI_ISL_5536463, EPI_ISL_5599480, EPI_ISL_5599487, EPI_ISL_5599496, EPI_ISL_5687044, EPI_ISL_5687045, EPI_ISL_5687046, EPI_ISL_5687047, EPI_ISL_5687048, EPI_ISL_5687049, EPI_ISL_5687050, EPI_ISL_5687051, EPI_ISL_5687052, EPI_ISL_5687053, EPI_ISL_5687054, EPI_ISL_5736276, EPI_ISL_5736282, EPI_ISL_5736285, EPI_ISL_5736289, EPI_ISL_5736302, EPI_ISL_5736307, EPI_ISL_5736314, EPI_ISL_5736583			
see above	Multidisciplinary Reserch Lab, Osun State University	Central Research Laboratory	Akinde; Ayorinde Babatunde James; Azuka Patrick Okwuraiwe; Babatunde Lawal Salako; Bamidele Iwalokun; Chika Kingsley Onwuamah; Folorunsho; Grace Oni; Josiah Ayoola Isong; Joy Ayoola; Muinah Adenike Fowora; Ngozi Mirabel Otuonye; Nyam Itse Yusuf; Ojo; Olabisi; Phasha-Muchemenye Mmatshepho; Richard Adegbola; Sola Ajibaye; Sunday Babatunde; Waidi Sule
EPI_ISL_4004796	NIC, Viral Respiratory Unit	Virology Departement	Aicha Bensalem; Aissam Hachid; Fawzi Derrar; Fayez Khardine; Fetouma Doudou; Mohamed Amine Beloufa
EPI_ISL_5510277, EPI_ISL_5510364	Natalspruit	National Institute for Communicable Diseases of the National Health Laboratory Service	Amoako DG; Bhiman JN; Everatt J; Ismail A; Mahlangu B; Mnguni A; Mohale T; Ntuli N; Scheepers C
EPI_ISL_4078218, EPI_ISL_4078219, EPI_ISL_4078220, EPI_ISL_4078221, EPI_ISL_4078222, EPI_ISL_4078223, EPI_ISL_4078224, EPI_ISL_4078225, EPI_ISL_4078226, EPI_ISL_4078227, EPI_ISL_4078228, EPI_ISL_4078229, EPI_ISL_4078230, EPI_ISL_4078231, EPI_ISL_4078232, EPI_ISL_4078233, EPI_ISL_4078234, EPI_ISL_4078235, EPI_ISL_4078236, EPI_ISL_4078237, EPI_ISL_4078238, EPI_ISL_4078239, EPI_ISL_4078240, EPI_ISL_4078241, EPI_ISL_4078242, EPI_ISL_4078243, EPI_ISL_4078244, EPI_ISL_4078245, EPI_ISL_4078246, EPI_ISL_4078247, EPI_ISL_4078248, EPI_ISL_4078249, EPI_ISL_4078250, EPI_ISL_4078251, EPI_ISL_4533450			
see above	National Health Laboratory Services, Virology	National Health Laboratory Services, Virology	Florette Treurnicht; Kathleen Subramoney
EPI_ISL_4602072, EPI_ISL_4602073, EPI_ISL_4602074, EPI_ISL_4602075, EPI_ISL_4602076, EPI_ISL_4602077, EPI_ISL_4602078, EPI_ISL_4602079, EPI_ISL_4602080, EPI_ISL_4602081, EPI_ISL_4602082, EPI_ISL_4602083, EPI_ISL_4602084, EPI_ISL_4602085			
see above	National Influenza Centre	National Influenza Centre	; Benjamiin B. Lindsey; Benjamin H. Foulkes; Dennis Laryea; Ernest Asiedu; Franklin Asiedu-Bekoe; Gordon Awandare; Ivy A. Asante; Joseph Oliver-Commey; Joyce Ngoi; Linda Boatemaa; Lorreta Kwasah; Mathew D. Parker; Michael Marks; Mildred Adusei-Poku; Sharon Hsu; Thushan I de Silva; William K. Ampofo
EPI_ISL_4891518, EPI_ISL_4891607, EPI_ISL_4891630	National Institute For Communicable Diseases Of The National Health Laboratory Service	National Institute for Communicable Diseases of the National Health Laboratory Service	Amoako DG; Bhiman JN; Everatt J; Ismail A; Mahlangu B; Mnguni A; Mohale T; Ntuli N; Scheepers C
EPI_ISL_4739527, EPI_ISL_4739528, EPI_ISL_4739529, EPI_ISL_4739530, EPI_ISL_4739531, EPI_ISL_4739532, EPI_ISL_4739533, EPI_ISL_4739534, EPI_ISL_4739535, EPI_ISL_4739536, EPI_ISL_4739567, EPI_ISL_4739569, EPI_ISL_4739580, EPI_ISL_4739581, EPI_ISL_4739584, EPI_ISL_4739587, EPI_ISL_4739592, EPI_ISL_4739598, EPI_ISL_4739600, EPI_ISL_4739601, EPI_ISL_4739603, EPI_ISL_4739614, EPI_ISL_4739620, EPI_ISL_4739622, EPI_ISL_4739627, EPI_ISL_4739628			
see above	National Public Health Laboratory (NPHL)	National Institute for Communicable Diseases of the National Health Laboratory Service	Adba Alfatih AlEmam; Amira suliaman wadi; Bhiman JN; Elamin Abualas; Elham Rizgalla; Everatt J; Hatem ahmed babeker Tamadur tag elser; Ismail A; Mahlangu B; Mnguni A; Mohale T; Mohamed Hassan Abdoelraheem Amoako DG; Muntaser elTayeb Ibrahim; Ntuli N; Shahinaz.A. Bedri; Yousif Rabih Makki
EPI_ISL_3845304, EPI_ISL_3845307, EPI_ISL_3845310, EPI_ISL_3845313, EPI_ISL_3845316, EPI_ISL_3845319, EPI_ISL_3845328, EPI_ISL_3845345, EPI_ISL_3845348, EPI_ISL_3845350, EPI_ISL_3845355, EPI_ISL_3845359, EPI_ISL_3845360, EPI_ISL_3845362, EPI_ISL_3845369, EPI_ISL_3845395, EPI_ISL_3845397, EPI_ISL_3845401, EPI_ISL_3845417, EPI_ISL_3869526, EPI_ISL_3869527, EPI_ISL_3869528, EPI_ISL_3869529, EPI_ISL_3869530, EPI_ISL_3869531, EPI_ISL_3869532, EPI_ISL_3869533, EPI_ISL_3869534, EPI_ISL_3869535, EPI_ISL_3869536, EPI_ISL_3869537, EPI_ISL_3920316, EPI_ISL_3920321, EPI_ISL_3920332, EPI_ISL_3920333, EPI_ISL_3920338, EPI_ISL_3920339, EPI_ISL_3920340, EPI_ISL_3920363, EPI_ISL_3920364, EPI_ISL_3920367, EPI_ISL_3920368, EPI_ISL_3920371, EPI_ISL_3920372, EPI_ISL_3920373, EPI_ISL_3920374, EPI_ISL_3920378, EPI_ISL_3920383, EPI_ISL_3920384, EPI_ISL_3920388, EPI_ISL_3920389, EPI_ISL_3920426, EPI_ISL_3920427, EPI_ISL_3920428, EPI_ISL_3920429, EPI_ISL_3920430, EPI_ISL_3920431, EPI_ISL_3920432, EPI_ISL_3920433, EPI_ISL_3920434, EPI_ISL_3920435			
see above	Naval Medical Research Unit No. 3	Naval Medical Research Center Biological Defense Research Directorate	Andrea E. Luquette; Andrew J. Bennett; Catherine E. Arnold; Chaselynn M. Watters; Emily K. Stefanov; Francisco Malagon; Kyle A. Long; Lindsay A. Glang; Logan J. Voegtly; Luis A. Estrella; Michael V. Deschenes; Regina Z. Cer; Robin H. Miller; Stephen M. Eggan; and Kimberly A. Bishop-Lilly
EPI_ISL_5510412, EPI_ISL_5510429	Ndlovu Research Centre	National Institute for Communicable Diseases of the National Health Laboratory Service	Amoako DG; Bhiman JN; Everatt J; Ismail A; Mahlangu B; Mnguni A; Mohale T; Ntuli N; Scheepers C
EPI_ISL_5510409	ORT Mobile lab	National Institute for Communicable Diseases of the National Health Laboratory Service	Amoako DG; Bhiman JN; Everatt J; Ismail A; Mahlangu B; Mnguni A; Mohale T; Ntuli N; Scheepers C

EPI_ISL_5367954, EPI_ISL_5368391, EPI_ISL_5368981, EPI_ISL_5369154	Osun State University, Multidisciplinary Research Lab	Nigerian Institute of Medical Research Central Research Laboratory	Akinde; Ayorinde Babatunde James; Azuka Patrick Okwuraiwe; Babatunde Lawal Salako; Bamidele Iwalokun; Chika Kingsley Onwuamah; Folorunsho; Grace Oni; Josiah Ayoola Isong; Joy Ayoola; Muinah Adenike Fowora; Ngozi Mirabel Otuonye; Nyam Itse Yusuf; Ojo; Olabisi; Phasha-Muchemenye Mmatshepho; Sola Ajibaye; Sunday Babatunde; Waidi Sule
EPI_ISL_3957790, EPI_ISL_3957791	Oudtshoorn Hospital wc OUD	NHLS/UCT	Arash Iranzadeh; Bruna Galvao; Carolyn Williamson; Deelan Doolabh; Diana Hardie; Gert Marais; Innocent Mudau; Lynn Tyers; Marvin Hsiao; Rageema Joseph; Stephen Korsman
EPI_ISL_5510288, EPI_ISL_5510289, EPI_ISL_5510290, EPI_ISL_5510291, EPI_ISL_5510371, EPI_ISL_5510418, EPI_ISL_5510446, EPI_ISL_5510463, EPI_ISL_5510464, EPI_ISL_5510466, EPI_ISL_5510472, EPI_ISL_5510475, EPI_ISL_5510483, EPI_ISL_5510485			
see above	Pathcare/Vermaak Centurion	National Institute for Communicable Diseases of the National Health Laboratory Service	Amoako DG; Bhiman JN; Everatt J; Ismail A; Mahlangu B; Mnguni A; Mohale T; Ntuli N; Scheepers C
EPI_ISL_3507268, EPI_ISL_3800206, EPI_ISL_3800565, EPI_ISL_3800653, EPI_ISL_3800654, EPI_ISL_3800655, EPI_ISL_3800656, EPI_ISL_3800657, EPI_ISL_3800658			
see above	Pfizer, Inc	Pfizer, Inc	Huiqing Si; Kena Swanson and the C4591001 study team; Kwok Lee; Paul Liberator; Qi Yang; Zhenghui Li
EPI_ISL_5477704	Rob Ferreira	National Institute for Communicable Diseases of the National Health Laboratory Service	Amoako DG; Bhiman JN; Everatt J; Ismail A; Mahlangu B; Mnguni A; Mohale T; Ntuli N; Scheepers C
EPI_ISL_4768104	Rwanda National Joint Taskforce COVID-24	Africa Centre for Excellence for Genomics of Infectious Diseases (ACEGID), Redeemer's University	A.N.; A.T.; Abechi; Ahmed, M.; Ajogbasile; Akano; Alice Kabanda; Arlene Uwituze; Ayo-Ale, B.; Ayoade, F.; C.A.; C.T.; Chukwu, G.; Dieudonne Mutangana; Enatha Mukantwari; Eromon; Esperance Umumararungu; F.V.; Folarin, O.; Happi; I.B.; Izuwayo Gerard; J.N.; J.U.; K.O.; Kayode; Leon Mutesa; Murebwayire Clarisse; Nosamiefan, I.; Oguzie; Okolie, J.; Olawoye; Olumade; Oluniyi; Ope-Ewe, O.; P.E.; P.S.; Philip, C.; Robert Rutayisire; Sindayiheba Reuben; Sobajo, T.; T.J.; Ugwu; Umuringa Jeanne d'Arc; Uwanibe; Yvan Butera
EPI_ISL_4768101	Rwanda National Joint Taskforce COVID-34	Africa Centre for Excellence for Genomics of Infectious Diseases (ACEGID), Redeemer's University	A.N.; A.T.; Abechi; Ahmed, M.; Ajogbasile; Akano; Alice Kabanda; Arlene Uwituze; Ayo-Ale, B.; Ayoade, F.; C.A.; C.T.; Chukwu, G.; Dieudonne Mutangana; Enatha Mukantwari; Eromon; Esperance Umumararungu; F.V.; Folarin, O.; Happi; I.B.; Izuwayo Gerard; J.N.; J.U.; K.O.; Kayode; Leon Mutesa; Murebwayire Clarisse; Nosamiefan, I.; Oguzie; Okolie, J.; Olawoye; Olumade; Oluniyi; Ope-Ewe, O.; P.E.; P.S.; Philip, C.; Robert Rutayisire; Sindayiheba Reuben; Sobajo, T.; T.J.; Ugwu; Umuringa Jeanne d'Arc; Uwanibe; Yvan Butera
EPI_ISL_4768107	Rwanda National Joint Taskforce COVID-36	Africa Centre for Excellence for Genomics of Infectious Diseases (ACEGID), Redeemer's University	A.N.; A.T.; Abechi; Ahmed, M.; Ajogbasile; Akano; Alice Kabanda; Arlene Uwituze; Ayo-Ale, B.; Ayoade, F.; C.A.; C.T.; Chukwu, G.; Dieudonne Mutangana; Enatha Mukantwari; Eromon; Esperance Umumararungu; F.V.; Folarin, O.; Happi; I.B.; Izuwayo Gerard; J.N.; J.U.; K.O.; Kayode; Leon Mutesa; Murebwayire Clarisse; Nosamiefan, I.; Oguzie; Okolie, J.; Olawoye; Olumade; Oluniyi; Ope-Ewe, O.; P.E.; P.S.; Philip, C.; Robert Rutayisire; Sindayiheba Reuben; Sobajo, T.; T.J.; Ugwu; Umuringa Jeanne d'Arc; Uwanibe; Yvan Butera
EPI_ISL_4768098	Rwanda National Joint Taskforce COVID-37	Africa Centre for Excellence for Genomics of Infectious Diseases (ACEGID), Redeemer's University	A.N.; A.T.; Abechi; Ahmed, M.; Ajogbasile; Akano; Alice Kabanda; Arlene Uwituze; Ayo-Ale, B.; Ayoade, F.; C.A.; C.T.; Chukwu, G.; Dieudonne Mutangana; Enatha Mukantwari; Eromon; Esperance Umumararungu; F.V.; Folarin, O.; Happi; I.B.; Izuwayo Gerard; J.N.; J.U.; K.O.; Kayode; Leon Mutesa; Murebwayire Clarisse; Nosamiefan, I.; Oguzie; Okolie, J.; Olawoye; Olumade; Oluniyi; Ope-Ewe, O.; P.E.; P.S.; Philip, C.; Robert Rutayisire; Sindayiheba Reuben; Sobajo, T.; T.J.; Ugwu; Umuringa Jeanne d'Arc; Uwanibe; Yvan Butera
EPI_ISL_4768119	Rwanda National Joint Taskforce COVID-51	Africa Centre for Excellence for Genomics of Infectious Diseases (ACEGID), Redeemer's University	A.N.; A.T.; Abechi; Ahmed, M.; Ajogbasile; Akano; Alice Kabanda; Arlene Uwituze; Ayo-Ale, B.; Ayoade, F.; C.A.; C.T.; Chukwu, G.; Dieudonne Mutangana; Enatha Mukantwari; Eromon; Esperance Umumararungu; F.V.; Folarin, O.; Happi; I.B.; Izuwayo Gerard; J.N.; J.U.; K.O.; Kayode; Leon Mutesa; Murebwayire Clarisse; Nosamiefan, I.; Oguzie; Okolie, J.; Olawoye; Olumade; Oluniyi; Ope-Ewe, O.; P.E.; P.S.; Philip, C.; Robert Rutayisire; Sindayiheba Reuben; Sobajo, T.; T.J.; Ugwu; Umuringa Jeanne d'Arc; Uwanibe; Yvan Butera
EPI_ISL_4768081	Rwanda National Joint Taskforce COVID-53	Africa Centre for Excellence for Genomics of Infectious Diseases (ACEGID), Redeemer's University	A.N.; A.T.; Abechi; Ahmed, M.; Ajogbasile; Akano; Alice Kabanda; Arlene Uwituze; Ayo-Ale, B.; Ayoade, F.; C.A.; C.T.; Chukwu, G.; Dieudonne Mutangana; Enatha Mukantwari; Eromon; Esperance Umumararungu; F.V.; Folarin, O.; Happi; I.B.; Izuwayo Gerard; J.N.; J.U.; K.O.; Kayode; Leon Mutesa; Murebwayire Clarisse; Nosamiefan, I.; Oguzie; Okolie, J.; Olawoye; Olumade; Oluniyi; Ope-Ewe, O.; P.E.; P.S.; Philip, C.; Robert Rutayisire; Sindayiheba Reuben; Sobajo, T.; T.J.; Ugwu; Umuringa Jeanne d'Arc; Uwanibe; Yvan Butera
EPI_ISL_4768090	Rwanda National Joint Taskforce COVID-59	Africa Centre for Excellence for Genomics of Infectious Diseases (ACEGID), Redeemer's University	A.N.; A.T.; Abechi; Ahmed, M.; Ajogbasile; Akano; Alice Kabanda; Arlene Uwituze; Ayo-Ale, B.; Ayoade, F.; C.A.; C.T.; Chukwu, G.; Dieudonne Mutangana; Enatha Mukantwari; Eromon; Esperance Umumararungu; F.V.; Folarin, O.; Happi; I.B.; Izuwayo Gerard; J.N.; J.U.; K.O.; Kayode; Leon Mutesa; Murebwayire Clarisse; Nosamiefan, I.; Oguzie; Okolie, J.; Olawoye; Olumade; Oluniyi; Ope-Ewe, O.; P.E.; P.S.; Philip, C.; Robert Rutayisire; Sindayiheba Reuben; Sobajo, T.; T.J.; Ugwu; Umuringa Jeanne d'Arc; Uwanibe; Yvan Butera
EPI_ISL_4768056	Rwanda National Joint Taskforce COVID-62	Africa Centre for Excellence for Genomics of Infectious Diseases (ACEGID), Redeemer's University	A.N.; A.T.; Abechi; Ahmed, M.; Ajogbasile; Akano; Alice Kabanda; Arlene Uwituze; Ayo-Ale, B.; Ayoade, F.; C.A.; C.T.; Chukwu, G.; Dieudonne Mutangana; Enatha Mukantwari; Eromon; Esperance Umumararungu; F.V.; Folarin, O.; Happi; I.B.; Izuwayo Gerard; J.N.; J.U.; K.O.; Kayode; Leon Mutesa; Murebwayire Clarisse; Nosamiefan, I.; Oguzie; Okolie, J.; Olawoye; Olumade; Oluniyi; Ope-Ewe, O.; P.E.; P.S.; Philip, C.; Robert Rutayisire; Sindayiheba Reuben; Sobajo, T.; T.J.; Ugwu; Umuringa Jeanne d'Arc; Uwanibe; Yvan Butera
EPI_ISL_4274017, EPI_ISL_4274021, EPI_ISL_4274022, EPI_ISL_4274024, EPI_ISL_4274030, EPI_ISL_4274034, EPI_ISL_4274036, EPI_ISL_4274038, EPI_ISL_4274040, EPI_ISL_4274042, EPI_ISL_4274045, EPI_ISL_4274047, EPI_ISL_4274049			
see above	Service des Maladies Infectieuses	Northwestern University - Center for Pathogen Genomics and Microbial Evolution	Egon A. Ozer; Judd F. Hultquist; Lacy M. Simons; Mamadou S. Sow; Mamoudou Maiga; Ramon Lorenzo-Redondo
EPI_ISL_4880527, EPI_ISL_4880528, EPI_ISL_4880529, EPI_ISL_4880530, EPI_ISL_4880531, EPI_ISL_4880532, EPI_ISL_4880533, EPI_ISL_4880534, EPI_ISL_4880535, EPI_ISL_4880536, EPI_ISL_4880540, EPI_ISL_4880612			
see above	Seychelles Public Health Laboratory	KEMRI-Wellcome Trust Research Programme,Kilifi	Agoti C.; Brigitte Pool; Githinji G.; Jude Gedeon; Lambisia A. Nokes D J.; Leon Biscornet.; Mburu M.W.; Meggy Louange; Mohamed K.S.; Morobe J.; Ndwiga L. Makori T.; Ochola I.; Ongera E.; de Laurent Z.
EPI_ISL_3546349	South Sudan Ministry of Health, WHO South Sudan, MRC/UVRI & LSHTM Uganda Research Unit	MRC/UVRI & LSHTM Uganda Research Unit, South Sudan Ministry of Health, WHO South Sudan	Abe G. Abias; Dan Lule Bugembe; Dennis Kenyi Lodiongo; James Ayei; John Rumunu; Joseph Francis Wamala; Juma John HM; Lul Lojok Deng; Matthew Cotten; My V.T. Phan; Pontiano Kaleebu; Richard Lino Loro Lako; Sudhir Bunga
EPI_ISL_5510390	Tembisa	National Institute for Communicable Diseases of the National Health Laboratory Service	Amoako DG; Bhiman JN; Everatt J; Ismail A; Mahlangu B; Mnguni A; Mohale T; Ntuli N; Scheepers C
EPI_ISL_4884452	Thembalethu CDC wc THC	NHLS/UCT	Arash Iranzadeh; Bruna Galvao; Carolyn Williamson; Deelan Doolabh; Diana Hardie; Gert Marais; Innocent Mudau; Lynn Tyers; Marvin Hsiao; Rageema Joseph; Stephen Korsman
EPI_ISL_4884454	Toekomsrus Clinic wc TKS	NHLS/UCT	Arash Iranzadeh; Bruna Galvao; Carolyn Williamson; Deelan Doolabh; Diana Hardie; Gert Marais; Innocent Mudau; Lynn Tyers; Marvin Hsiao; Rageema Joseph; Stephen Korsman
EPI_ISL_3342350, EPI_ISL_3342351, EPI_ISL_3342353, EPI_ISL_3342354, EPI_ISL_3342359, EPI_ISL_3342360, EPI_ISL_3342365, EPI_ISL_3342369, EPI_ISL_3342370, EPI_ISL_3342376, EPI_ISL_3342378, EPI_ISL_3342380, EPI_ISL_3342382, EPI_ISL_3342383, EPI_ISL_3342386, EPI_ISL_3342388, EPI_ISL_3342389, EPI_ISL_3342404, EPI_ISL_3342407, EPI_ISL_3342409, EPI_ISL_3342413, EPI_ISL_3342415, EPI_ISL_3342418, EPI_ISL_3342420, EPI_ISL_3411470			
see above	Tshwaragano laboratory	National Institute for Communicable Diseases of the National Health Laboratory Service	Amoako DG; Bhiman JN; Everatt J; Ismail A; Mahlangu B; Mnguni A; Mohale T; Ntuli N; Scheepers C
EPI_ISL_4273939, EPI_ISL_4273941, EPI_ISL_4273945	University Clinical Research Center (UCRC)	Northwestern University - Center for Pathogen Genomics and Microbial Evolution	Almoustapha I. Maiga; Anou M. Somboro; Egon A. Ozer; Ibrahima Guindo; Judd F. Hultquist; Lacy M. Simons; Mamoudou Maiga; Ramon Lorenzo-Redondo
EPI_ISL_4513512, EPI_ISL_4513513, EPI_ISL_4513514, EPI_ISL_4513516, EPI_ISL_4513517, EPI_ISL_4513518, EPI_ISL_4513519, EPI_ISL_4513520, EPI_ISL_4513521, EPI_ISL_4513522, EPI_ISL_4513523, EPI_ISL_4513524, EPI_ISL_4513525, EPI_ISL_4513526, EPI_ISL_4513527, EPI_ISL_4513685			
see above	University of Zambia, School of Veterinary Medicine	UNZAVET and PATH	Daniel Bridges; Mulenga Mwenda-Chimfwembe; Ngonda Saasa; ZNPHI and ZGSC
EPI_ISL_3342356, EPI_ISL_3342358, EPI_ISL_3342361, EPI_ISL_3342362, EPI_ISL_3342363, EPI_ISL_3342364, EPI_ISL_3342366, EPI_ISL_3342368, EPI_ISL_3342371, EPI_ISL_3342372, EPI_ISL_3342373, EPI_ISL_3342374, EPI_ISL_3342375, EPI_ISL_3342377, EPI_ISL_3342381, EPI_ISL_3342384, EPI_ISL_3342387, EPI_ISL_3342392, EPI_ISL_3342394, EPI_ISL_3342395, EPI_ISL_3342396, EPI_ISL_3342397, EPI_ISL_3342398, EPI_ISL_3342403, EPI_ISL_3342410, EPI_ISL_3411471			
see above	Upington laboratory	National Institute for Communicable Diseases of the National Health Laboratory Service	Amoako DG; Bhiman JN; Everatt J; Ismail A; Mahlangu B; Mnguni A; Mohale T; Ntuli N; Scheepers C
EPI_ISL_3716349, EPI_ISL_3716350, EPI_ISL_3716351, EPI_ISL_3716352, EPI_ISL_3716353, EPI_ISL_3716354, EPI_ISL_3716355, EPI_ISL_3716356, EPI_ISL_3716357, EPI_ISL_3716358, EPI_ISL_3716359, EPI_ISL_3716360, EPI_ISL_3716361, EPI_ISL_3716362, EPI_ISL_3716363, EPI_ISL_3716364, EPI_ISL_3716365, EPI_ISL_3716366, EPI_ISL_3716367, EPI_ISL_3716368, EPI_ISL_3716369, EPI_ISL_3716370, EPI_ISL_3716371, EPI_ISL_3716372, EPI_ISL_3716373, EPI_ISL_3716374, EPI_ISL_3716375, EPI_ISL_3716376, EPI_ISL_3716377, EPI_ISL_3716378, EPI_ISL_3716379, EPI_ISL_3716380, EPI_ISL_3716381, EPI_ISL_3716382, EPI_ISL_3716383, EPI_ISL_3716384, EPI_ISL_3716385, EPI_ISL_3716386, EPI_ISL_3716387, EPI_ISL_3716388, EPI_ISL_3716389, EPI_ISL_3716390, EPI_ISL_3716391, EPI_ISL_3716392, EPI_ISL_3716393, EPI_ISL_3716394, EPI_ISL_3716395, EPI_ISL_3716396, EPI_ISL_3716397, EPI_ISL_3716398, EPI_ISL_3716399, EPI_ISL_3716400, EPI_ISL_3716401, EPI_ISL_3716402, EPI_ISL_3716403, EPI_ISL_3716404, EPI_ISL_3716405, EPI_ISL_3716406, EPI_ISL_3716407, EPI_ISL_3716408, EPI_ISL_3716409, EPI_ISL_3716410, EPI_ISL_3716411, EPI_ISL_3716412, EPI_ISL_3716413, EPI_ISL_3716414, EPI_ISL_3716415, EPI_ISL_3716416, EPI_ISL_3716417, EPI_ISL_3716418, EPI_ISL_3716419, EPI_ISL_3716420, EPI_ISL_3716421, EPI_ISL_3716422, EPI_ISL_3716423, EPI_ISL_3716424, EPI_ISL_3716425, EPI_ISL_3716426, EPI_ISL_3716427, EPI_ISL_3716428, EPI_ISL_3716429, EPI_ISL_3716430, EPI_ISL_3716431, EPI_ISL_3716432, EPI_ISL_3716433, EPI_ISL_3716434, EPI_ISL_3716435, EPI_ISL_3716436, EPI_ISL_3716437, EPI_ISL_3716438, EPI_ISL_3716439, EPI_ISL_3716440, EPI_ISL_3716441, EPI_ISL_3716442, EPI_ISL_3716443, EPI_ISL_3716444, EPI_ISL_3716445, EPI_ISL_3716446, EPI_ISL_3716447, EPI_ISL_3716448, EPI_ISL_3716449, EPI_ISL_3716450, EPI_ISL_3716451, EPI_ISL_3716452, EPI_ISL_3716453, EPI_ISL_3716454, EPI_ISL_3716455, EPI_ISL_3716456, EPI_ISL_3716457, EPI_ISL_3716458, EPI_ISL_3716459, EPI_ISL_3716460, EPI_ISL_3716461, EPI_ISL_3716462, EPI_ISL_3716463, EPI_ISL_3716464, EPI_ISL_3716465, EPI_ISL_3716466, EPI_ISL_3716467, EPI_ISL_3716468, EPI_ISL_3716469, EPI_ISL_3716470, EPI_ISL_3716471, EPI_ISL_3716472, EPI_ISL_3716473, EPI_ISL_3716474, EPI_ISL_3716475, EPI_ISL_3716476, EPI_ISL_3716477, EPI_ISL_3716478, EPI_ISL_3716479, EPI_ISL_3716480, EPI_ISL_3716481, EPI_ISL_3716482, EPI_ISL_3716483, EPI_ISL_3716484, EPI_ISL_3716485, EPI_ISL_3716486, EPI_ISL_3716487, EPI_ISL_3716488, EPI_ISL_3716489, EPI_ISL_3716490, EPI_ISL_3716491, EPI_ISL_3716492, EPI_ISL_3716493, EPI_ISL_3716494, EPI_ISL_3716495, EPI_ISL_3716496, EPI_ISL_3716497, EPI_ISL_3716498, EPI_ISL_3716499, EPI_ISL_3716500, EPI_ISL_3716501, EPI_ISL_3716502, EPI_ISL_3716503, EPI_ISL_3716504, EPI_ISL_3716505, EPI_ISL_3716506, EPI_ISL_3716507, EPI_ISL_3716508, EPI_ISL_3716509, EPI_ISL_3716510, EPI_ISL_3716511, EPI_ISL_3716512, EPI_ISL_3716513, EPI_ISL_3716514, EPI_ISL_3716515, EPI_ISL_3716516, EPI_ISL_3716517, EPI_ISL_3716518, EPI_ISL_3716519, EPI_ISL_3716520, EPI_ISL_3716521, EPI_ISL_3716522, EPI_ISL_3716523, EPI_ISL_3716524, EPI_ISL_3716525, EPI_ISL_3716526, EPI_ISL_3716527, EPI_ISL_3716528, EPI_ISL_3716529, EPI_ISL_3716531, EPI_ISL_3716532, EPI_ISL_3716533, EPI_ISL_3716534, EPI_ISL_3716535, EPI_ISL_3716536, EPI_ISL_3716537, EPI_ISL_3716538, EPI_ISL_3716539, EPI_ISL_3716540, EPI_ISL_3716541, EPI_ISL_3716542, EPI_ISL_3716543, EPI_ISL_3716544, EPI_ISL_3716545, EPI_ISL_3716546, EPI_ISL_3716547, EPI_ISL_3716548, EPI_ISL_3716549, EPI_ISL_3716550, EPI_ISL_3716551, EPI_ISL_3716552, EPI_ISL_3716553, EPI_ISL_3716554, EPI_ISL_3716555, EPI_ISL_3716556, EPI_ISL_3716557, EPI_ISL_3716558, EPI_ISL_3716559, EPI_ISL_3716560, EPI_ISL_3716561, EPI_ISL_3716562, EPI_ISL_3716563, EPI_ISL_3716564, EPI_ISL_3716565, EPI_ISL_3716566, EPI_ISL_3716567, EPI_ISL_3716568, EPI_ISL_3716569, EPI_ISL_3716570, EPI_ISL_3716571, EPI_ISL_3716572, EPI_ISL_3716573, EPI_ISL_3716574, EPI_ISL_3716575, EPI_ISL_3716576, EPI_ISL_3716577, EPI_ISL_3716578, EPI_ISL_3716579, EPI_ISL_3716580, EPI_ISL_3716581, EPI_ISL_3716582, EPI_ISL_3716583, EPI_ISL_3716584, EPI_ISL_3716585, EPI_ISL_3716586, EPI_ISL_3716587, EPI_ISL_3716588, EPI_ISL_3716589, EPI_ISL_3716590, EPI_ISL_3716591, EPI_ISL_3716592, EPI_ISL_3716593, EPI_ISL_3716594, EPI_ISL_3716595, EPI_ISL_3716596, EPI_ISL_3716597, EPI_ISL_3716598, EPI_ISL_3716599, EPI_ISL_3716600, EPI_ISL_3716601, EPI_ISL_3716602, EPI_ISL_3716603, EPI_ISL_3716604, EPI_ISL_3716605, EPI_ISL_3716606, EPI_ISL_3716607, EPI_ISL_3716608, EPI_ISL_3716609, EPI_ISL_3716610, EPI_ISL_3716611, EPI_ISL_3716612, EPI_ISL_3716613, EPI_ISL_3716614, EPI_ISL_3716615, EPI_ISL_3716616, EPI_ISL_3716617, EPI_ISL_3716618, EPI_ISL_3716619, EPI_ISL_3716620, EPI_ISL_3716621, EPI_ISL_3716622, EPI_ISL_3716623, EPI_ISL_3716624, EPI_ISL_3716625, EPI_ISL_3716626, EPI_ISL_3716627, EPI_ISL_3716628, EPI_ISL_3716629, EPI_ISL_3716630, EPI_ISL_3716631, EPI_ISL_3716632, EPI_ISL_3716633, EPI_ISL_3716634, EPI_ISL_3716635, EPI_ISL_3716636, EPI_ISL_3716637, EPI_ISL_3716638, EPI_ISL_3716639, EPI_ISL_3716640, EPI_ISL_3716641, EPI_ISL_3716642, EPI_ISL_3716643, EPI_ISL_3716644, EPI_ISL_3716645, EPI_ISL_3716646, EPI_ISL_3716647, EPI_ISL_3716648, EPI_ISL_3716649, EPI_ISL_3716650, EPI_ISL_3716651, EPI_ISL_3716652, EPI_ISL_3716653, EPI_ISL_3716654, EPI_ISL_3716655, EPI_ISL_3716656, EPI_ISL_3716657, EPI_ISL_3716658, EPI_ISL_3716659, EPI_ISL_3716660, EPI_ISL_3716661, EPI_ISL_3716662, EPI_ISL_3716663, EPI_ISL_3716664, EPI_ISL_3716665, EPI_ISL_3716666, EPI_ISL_3716667, EPI_ISL_3716668, EPI_ISL_3716669, EPI_ISL_3716670, EPI_ISL_3716671, EPI_ISL_3716672, EPI_ISL_3716673, EPI_ISL_3716674, EPI_ISL_3716675, EPI_ISL_3716676, EPI_ISL_3716677, EPI_ISL_3716678, EPI_ISL_3716679, EPI_ISL_3716680, EPI_ISL_3716681, EPI_ISL_3716682, EPI_ISL_3716683, EPI_ISL_3716684, EPI_ISL_3716685, EPI_ISL_3716686, EPI_ISL_3716687, EPI_ISL_3716688, EPI_ISL_3716689, EPI_ISL_3716690, EPI_ISL_3716691, EPI_ISL_3716692, EPI_ISL_3716693, EPI_ISL_3716694, EPI_ISL_3716695, EPI_ISL_3716696, EPI_ISL_3716697, EPI_ISL_3716698, EPI_ISL_3716699, EPI_ISL_3716700, EPI_ISL_3716701, EPI_ISL_3716702, EPI_ISL_3716703, EPI_ISL_3716704, EPI_ISL_3716705, EPI_ISL_3716706, EPI_ISL_3716707, EPI_ISL_3716708, EPI_ISL_3716709, EPI_ISL_3716710, EPI_ISL_3716711, EPI_ISL_3716828, EPI_ISL_3716829, EPI_ISL_3716830, EPI_ISL_3716831, EPI_ISL_3716832, EPI_ISL_3716833, EPI_ISL_3716834, EPI_ISL_3716835, EPI_ISL_3716836, EPI_ISL_3716837, EPI_ISL_3716838, EPI_ISL_3716839, EPI_ISL_3716840, EPI_ISL_3716841, EPI_ISL_3716842, EPI_ISL_3716843, EPI_ISL_3716844			
see above	Virology Unit, Institut Pasteur de Madagascar	Institut Pateur de Dakar	Cara E. Brook; Christian Ranaivoson; Dia Ndongo; Diagne Moussa Moïse; Diallo Amadou; Faye Ousmane; Helisoa Razafimanjato; Jean-Michel Heraud; Loucoubar Cheikh; Ndiaye Ndack; Norosoa Razanajatovo; Philippe Dussart; Sall Amadou Alpha; Sankhe Safietou; Soa Fy Andriamandimby; Tsiry Randriambolamanantsoa; Vololoniaina Raharinosy
EPI_ISL_4411129, EPI_ISL_4411130	WACCBIP, University of Ghana	WACCBIP, University of Ghana	Abdul-Karim Abass; Bright K. Yemi; Collins M. Morang'a; Deborah N. A. Mettle; Dennis Adu-Gyasi; Dominic S. Y. Amuzu; Edward Danso Fenteng; Emmanuel Kudjo; Emmanuella Amoako; Evelyn B. Quansah; Frederick Tei-Maya; Israel Osei-Wusu; Joe K. Mutungi; Joyce M. Ngoi; Kwado Poku Asante; Lawrence Ofori-Boadu; Lucas N. Amenga-Etego; Michael Owus; Nicaise T. Ndam; Oliver Commey; Oliver D. Boakye; Patrick Tetteh Ababio; Paul Owusu-Oduro; Peter K. Quashie; Philip M. Soglo; Richard Odame Phillips; Samirah Saiid; Samuel Armoo; Samuel Kaba Akoriyea; Sylvester Dassah; Theophilus Odoom; Victor Asoala; Vincent Appiah; Violette V. Mâ€™cormack; William K. Ampofo; Yaw Bediako; and Gordon A. Awandare
