# Supplementary material for: Transmission networks of SARS-CoV-2 in Coastal Kenya during the first two waves: A retrospective genomic study
Source: eLife. 2022 Jun 14;11:e71703. doi: 10.7554/eLife.71703 (PMC9282859; doi:10.7554/eLife.71703)
Supplement: Transparent reporting form [file elife-71703-transrepform1.docx]

***eLife’s* transparent reporting form**

We encourage authors to provide detailed information *within their submission* to facilitate the interpretation and replication of experiments. Authors can upload supporting documentation to indicate the use of appropriate reporting guidelines for health-related research (see [EQUATOR Network](http://www.equator-network.org/%20)), life science research (see the [BioSharing Information Resource](https://biosharing.org/)), or the [ARRIVE guidelines](http://www.plosbiology.org/article/info:doi/10.1371/journal.pbio.1000412) for reporting work involving animal research. Where applicable, authors should refer to any relevant reporting standards documents in this form.

If you have any questions, please consult our Journal Policies and/or contact us: [editorial@elifesciences.org](mailto:editorial@elifesciences.org).

**Sample-size estimation**

- You should state whether an appropriate sample size was computed when the study was being designed
- You should state the statistical method of sample size computation and any required assumptions
- If no explicit power analysis was used, you should describe how you decided what sample (replicate) size (number) to use

This was an observational study with a primary objective of describing SARS-CoV-2 genomic surveillance data to inform public health response. There was no formal sample size calculation exercise undertaken.

In the results section, under the sub-section “**COVID-19 waves in Coastal Kenya and sequencing at KWTRP**” we detail on the total number of SARS-CoV-2 identified as positive and that ~18% were genome sequenced.

In Figure 2C, we detail the monthly proportion of samples sequenced from the total positive tests recorded in our laboratory.

The choice of samples to sequence and the number sequenced was informed by:

(a) Diagnostic RT-PCR threshold <30.0 i.e. likely to have high virus titer predicted hence high likelihood of genomic sequencing.

(b) Early in the pandemic we aimed to sequence all identified positive samples but during the wave one and wave two period we aimed to sequence >=10% of the positive samples identified across the six counties.

The decision to sequence ~ 10% of the positives was in part informed by our local sequencing capacity.

Please outline where this information can be found within the submission (e.g., sections or figure legends), or explain why this information doesn’t apply to your submission:

**Replicates**

- You should report how often each experiment was performed
- You should include a definition of biological versus technical replication
- The data obtained should be provided and sufficient information should be provided to indicate the number of independent biological and/or technical replicates
- If you encountered any outliers, you should describe how these were handled
- Criteria for exclusion/inclusion of data should be clearly stated
- High-throughput sequence data should be uploaded before submission, with a private link for reviewers provided (these are available from both GEO and ArrayExpress)

Please outline where this information can be found within the submission (e.g., sections or figure legends), or explain why this information doesn’t apply to your submission:

The diagnostic RT-PCR and sequencing processes always included positive and negative controls for quality control assessment and assessment of run validity.

Sequences reported are based on an assembly pipeline that only accepts nucleotide calls where read depth exceeded ×20.

Only sequences with coverage of >80% of the total SARS-CoV-2 genome length were included in the current study.

During building of time-resolved phylogenetic trees using Tree-Time program, any outlier sequences from the molecular clock-like evolution were removed from the analysis.

The coastal Kenya genomes have been uploaded to the GISAID database (accession numbers provided in the manuscript) and the process of submission to GenBank database is currently ongoing.

We performed replicated discrete phylogeographic analyses based on random subset of genomic sequences. Each subset was obtained by subsampling available Kenyan genomic sequences according to the COVID-19 incidence recorded in each sampled county during the study period.

This information has been provided under the material and methods section of the manuscript under the subsections “SARS-CoV-2 genome assembly”, “Phylogenetic analysis”, “import/export analysis” AND PHYLOG.

**Statistical reporting**

- Statistical analysis methods should be described and justified
- Raw data should be presented in figures whenever informative to do so (typically when N per group is less than 10)
- For each experiment, you should identify the statistical tests used, exact values of N, definitions of center, methods of multiple test correction, and dispersion and precision measures (e.g., mean, median, SD, SEM, confidence intervals; and, for the major substantive results, a measure of effect size (e.g., Pearson's r, Cohen's d)
- Report exact p-values wherever possible alongside the summary statistics and 95% confidence intervals. These should be reported for all key questions and not only when the p-value is less than 0.05.

Please outline where this information can be found within the submission (e.g., sections or figure legends), or explain why this information doesn’t apply to your submission:

Statistical analysis was undertaken in R statistical software.

Summary statistics were calculated and provided for various groups and these included proportions, means, median and ranges where applicable.

The linearity of the relationship between sampling date and accumulation of nucleotide changes was assessed using the lm function in R and a correlation co-efficient.

Comparison of proportions was using Chi-squared test or Fisher’s exact test as appropriate.

(For large datasets, or papers with a very large number of statistical tests, you may upload a single table file with tests, Ns, etc., with reference to sections in the manuscript.)

**Group allocation**

- Indicate how samples were allocated into experimental groups (in the case of clinical studies, please specify allocation to treatment method); if randomization was used, please also state if restricted randomization was applied
- Indicate if masking was used during group allocation, data collection and/or data analysis

Please outline where this information can be found within the submission (e.g., sections or figure legends), or explain why this information doesn’t apply to your submission:

This was an observational study, therefore no randomization.

The genome sequences were assigned into the various groups based on the Pango lineage identified, county of sampling or time of sampling (introductions phase, wave wone and wave two).

The details are provided in the in the “Study period and population” and “Lineage assignment and calling of amino acid changes” sub-sections under materials and methods section.

**Additional data files (“source data”)**

- We encourage you to upload relevant additional data files, such as numerical data that are represented as a graph in a figure, or as a summary table
- Where provided, these should be in the most useful format, and they can be uploaded as “Source data” files linked to a main figure or table
- Include model definition files including the full list of parameters used
- Include code used for data analysis (e.g., R, MatLab)
- Avoid stating that data files are “available upon request”

Please indicate the figures or tables for which source data files have been provided:

The source data files for the figures have been provided.

These scripts used in our analysis are publicly available via the Harvard dataverse link below:

<https://doi.org/10.7910/DVN/4ZZYIM>
